# Effects of Low Nighttime Temperature on Fatty Acid Content in Developing Seeds from *Brassica napus* L. Based on RNA-Seq and Metabolome

**DOI:** 10.3390/plants12020325

**Published:** 2023-01-10

**Authors:** Chao Mi, Chao Sun, Yuting Yuan, Fei Li, Qian Wang, Haiping Zhu, Shuijin Hua, Liangbin Lin

**Affiliations:** 1 College of Agronomy and Biotechnology, Yunnan Agricultural University, Kunming 650201, China; 2 Agricultural Research Institute, Tibet Academy of Agriculture and Animal Husbandry Sciences, Lhasa 850032, China; 3 Yunnan Key Laboratory for Wild Plant Resources, Department of Economic Plants and Biotechnology, Kunming Institute of Botany, Chinese Academy of Sciences, Kunming 650204, China; 4 Horticultural Research Institute, Yunnan Academy of Agricultural Sciences, Kunming 650200, China; 5 Food Crops Research Institute, Yunnan Academy of Agricultural Sciences, Kunming 650200, China; 6 Institute of Crop and Nuclear Technology Utilization, Zhejiang Academy of Agricultural Sciences, 17, Hangzhou 310021, China

**Keywords:** *Brassica napus* L., seeds, nighttime temperature, fatty acid content, RNA-seq, metabolome, *cis*-acting element involved in low-temperature responsiveness, oleic acid, erucic acid, *HB1*, *KCS*

## Abstract

*Brassica napus* L. is a vital plant oil resource worldwide. The fatty acid biosynthesis and oil accumulation in its seeds are controlled by several genetic and environmental factors, including daytime and nighttime temperatures. We analyzed changes in oleic and erucic acid content in two double haploid (DH) lines, DH0729, a weakly temperature-sensitive line, and DH0815, a strongly temperature-sensitive line, derived from *B. napus* plants grown at different altitudes (1600, 1800, 2000, 2200, and 2400 m a.s.l., 28.85° N, 112.35° E) and nighttime temperatures (20/18, 20/16, 20/13 and 20/10 °C, daytime/nighttime temperature). Based on medium- and long-chain fatty acid metabolites, the total oleic acid content 35 and 43 days after flowering was significantly lower in low nighttime temperature (LNT, 20/13 °C) plants than in high nighttime temperature (HNT, 20/18 °C) plants (HNT: 58–62%; LNT: 49–54%; an average decrease of 9%), and the total erucic acid content was significantly lower in HNT than in LNT plants (HNT: 1–2%; LNT: 8–13%; an average increase of 10%). An RNA-seq analysis showed that the expression levels of *SAD* (LOC106366808), *ECR* (LOC106396280), *KCS* (LOC106419344), *KAR* (LOC106367337), *HB1*(LOC106430193), and *DOF5* (LOC111211868) in STSL seeds increased under LNT conditions. In STSL seeds, a base mutation in the *cis*-acting element involved in low-temperature responsiveness (LTR), the *HB1* and *KCS* promoter caused loss of sensitivity to low temperatures, whereas that of the *KCS* promoter caused increased sensitivity to low temperatures.

## 1. Introduction

Plant oil is an important food component that plays a vital role in human health and can serve as a renewable resource of biodiesel and industrial raw materials [1,2]. *Brassica napus* L. (*B. napus*, 2*n* = 38) is widely planted worldwide and is one of the major sources of edible vegetable oil, owing to a high yield, high oil content, and good quality [3,4]. Triacylglycerols (TAGs) are the main constituent of rapeseed oil, and the oil content and fatty acid composition provide the basis of its edible and economic value [5]. Therefore, it is of great scientific significance to study regulatory mechanisms involved in oil accumulation in *B. napus* developing seeds.

Oil accumulation in seeds is controlled by both genetic and environmental factors (temperature, light, latitude, altitude, etc.). Genetic factors commonly predominate, but environmental factors cannot not be completely ignored [6,7]. For example, temperature plays an important role in the maturation stage. Excessively high temperature affects oil accumulation in seeds, resulting in lower yield and oil content. While low temperatures can improve yield and oil content, excessively low temperatures prolong the whole growth period, affecting overall crop development [6]. A suitable growth temperature not only promotes a high yield but also facilitates oil accumulation, thereby improving the nutritional value of rapeseed. *B. napus* was planted in agroecological areas at different altitudes [Nanjing (altitude: 8.9 m), Xining (altitude: 2261.2 m), and Lhasa (altitude: 3658 m)]. The oil content was assessed as a function of altitude 35 days after flowering (DAF). With increasing altitude, the oil content increased, and the expression of oil accumulation-related genes was significantly upregulated in seeds [8]. High daytime temperature was not conducive to oil accumulation in seeds, increasing the saturated fatty acid (SFA) content, whereas low daytime temperatures promoted oil accumulation in seeds, increased the content of unsaturated fatty acid (UFA), and promoted fatty acid composition in mature seeds [6]. Fatty acid biosynthesis can be divided into three stages. Firstly, the initial substrate acetyl-CoA is used to synthesize the fatty acids of C16–C18 chains in plastids (de novo biosynthesis and modification of fatty acids). Secondly, transport to the endoplasmic reticulum allows for carbon chain elongation and fatty acid desaturation (carbon chain elongation and fatty acid desaturation). Finally, fatty acids and glycerol are processed into TAGs and storage oils (TAG synthesis) [9]. Fatty acid biosynthesis is controlled by fatty acid metabolism genes (*ACC*, *KAS II*, *HAD*, *EAR*, *KAR*, *ACP*, *SAD*, *FAD2*, *FAD3*, *LPAAT*, *DGAT*, *PDAT*, *FAE1*, *STERO*, *OBO*, *OBP*, and so on) [10,11,12,13,14,15] and transcription factors (TFs; WRI1, LEC1, LEC2, FUS3, L1L, DOF11, DOF4, and DOF5), and it regulates oil accumulation [16,17,18,19,20]. In sunflower (*Helianthus annuus* L.) mutants CAS-6 and CAS-14, maximum oleic and linoleic acid content values were obtained at daytime/nighttime temperatures of 39/24 °C and 20/10 °C, respectively [21].

Plant hormones regulate seed development and storage compound (oil, protein, and carbohydrate) accumulation. Gibberellin negatively regulates lipid accumulation by upregulating the expression of GDSL-type lipase genes in seeds. The GDSL family genes SFAR1–SFAR5 act downstream of the GA signaling pathway to reduce lipid storage in *Arabidopsis* seeds [22]. During variable temperature development (25/9 °C) genes related to fatty acid biosynthesis and GA metabolism are significantly upregulated in high oil content seeds (*B. napus*) [5]. Two lines of *B. napus* were planted in different agroecological areas (Wuhan and Chongqing), and the degree of increase in oil content was significantly higher in high oil content seeds compared with low oil content seeds. RNA-seq analysis showed that differentially expressed genes (DEGs) related to photosynthesis, carbohydrates, lipids, phytohormones, transporters, and TAG and fatty acid synthesis tend to be upregulated in a high oil content line. For example, GA biosynthesis-related genes (*BnGA1*, *BnGA3*, *BnGA4*) were upregulated and DELLA genes were downregulated in a high oil content line, indicating that high temperature activated the GA signaling pathway in seeds [2]. Abscisic acid induces the expression of the fatty acid elongase gene (*FAE1*) and promotes long-chain fatty acid and TAG accumulation [23]. These studies showed the effect of oil accumulation or plant hormone regulation in developing and mature seeds at high temperatures; however, there are few reports of nighttime temperature-regulated fatty acid composition in *B. napus* seeds. In 2019–2020, two double haploid (DH) lines, *B. napus* L. (DH0815 and DH0729) were planted at different altitudes (1600, 1800, 2000, 2200, 2400 m a.s.l., 28.85° N, 112.35° E, Laowodi Mountain, Yuanjiang County, Yunnan Province, China), and the degree of change in oleic and erucic acid content in mature seeds was significantly greater in DH0815 than in DH0729, but the results were not published. Under the same agroecological conditions, an increase in altitude leads to a change in nighttime temperature; hence, we obtained a strongly temperature-sensitive line DH0815 (STSL) and a weakly temperature-sensitive line (WTSL) DH0729. In this study, we used these DH lines to study the effects of different nighttime temperatures on fatty acid metabolites while also exploring changes in DEGs in developing seeds based on RNA-seq data. The results can provide a theoretical basis for planting area distribution and breeding of *B. napus* plants with a high unsaturated fatty acid content.

## 2. Results

### 2.1. Effect of Different Nighttime Temperatures on the Fatty Acid Content of Seeds

According to tests at different attitudes (Tables S1 and S2), the content of seven fatty acids in the WTSL and STSL changed with an increase in altitude, and the coefficient of variation (CV) for the MUFA (oleic and erucic acid content) content was greater than for the SFA content. The highest CVs for the oleic and erucic acid content were 3.11 and 28.52 in the WTSL, and 9.09 and 48.98 in the STSL, respectively. Based on these results, 20/18, 20/16, 20/13, and 20/10 °C were used in tests of different nighttime temperatures.

**Figure 1:**
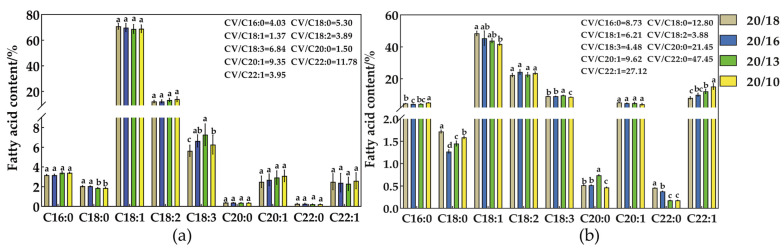
(**a**) Fatty acid content at different nighttime temperatures (WTSL); (**b**) fatty acid content at different nighttime temperatures (STSL). CV/C16:0, CV/C18:0, CV/C18:1, CV/C18:2, CV/C18:3, CV/C20:0, CV/C20:1, CV/C22:0, and CV/C22:1 represent coefficients of variation (CV) for palmitic acid, stearic acid, oleic acid, linoleic acid, linolenic acid, eicosenoic acid, arachidic acid, docosanoic acid, and erucic acid content in DH0729 and DH0815 in the mature period. CV = SD/Mean × 100%. Lowercase letters were deemed to be statistically significant at *p* ≤ 0.05.

The effect of different nighttime temperatures on the fatty acid content of seeds is shown in Figure 1. The WTSL and STSL showed different responses to nighttime temperatures. Other than for linoleic acid, the CVs of the content of eight fatty acids in the WTSL were lower than those in the STSL. In the WTSL, the contents of docosanoic and arachidic acids had the highest CVs of 11.78 and 9.35, respectively, whereas oleic acid content had the lowest CV of 1.35. In contrast, in the STSL, the content of SFAs (palmitic, stearic, arachidic, and docosanoic acids) increased slightly, while that of oleic acid decreased more definitively with decreasing nighttime temperature. At the same time, the contents of linoleic, linolenic, and erucic acids increased with decreased nighttime temperatures. Five fatty acids (palmitic, oleic, linoleic, linolenic, and erucic acid) accounted for more than 90% of the total fatty acids in *B. napus* seeds. With a decrease in nighttime temperature, the oleic acid content decreased from 48.48% to 41.85%, and erucic acid increased from 8.02 % to 15.21% in the STSL; however, in the WTSL, the corresponding content changes were not significant, being only 2% and 5%, respectively.

With a decrease in nighttime temperature, the development period of *B. napus* increased (Table 1). The highest CVs from full flowering to final flowering and from bolting to the beginning of flowering were 25.46 (WTSL) and 36.58 (STSL), respectively, with the development periods increasing to 10 d (WTSL) and 6 d (STSL) and 25 d (WTSL) and 7 d (STSL), respectively. The development periods of CVs from beginning of flowering to full flowering and from final flowering to maturation increased to 6 d (WTSL) and 46 d (STSL) and 7 d and 14 d, respectively. The whole period increased to 39 d (WTSL) and 54 d (STSL). Considering the changes in fatty acid content and growth period, 20/13 °C was selected as the best temperature condition that did not affect the field production rhythm.

### 2.2. Qualitative and Quantitative Analyses of Medium- and Long-Chain Fatty Acid Metabolites in Seeds at Different Developmental Stages at Different Nighttime Temperatures

**Figure 2:**
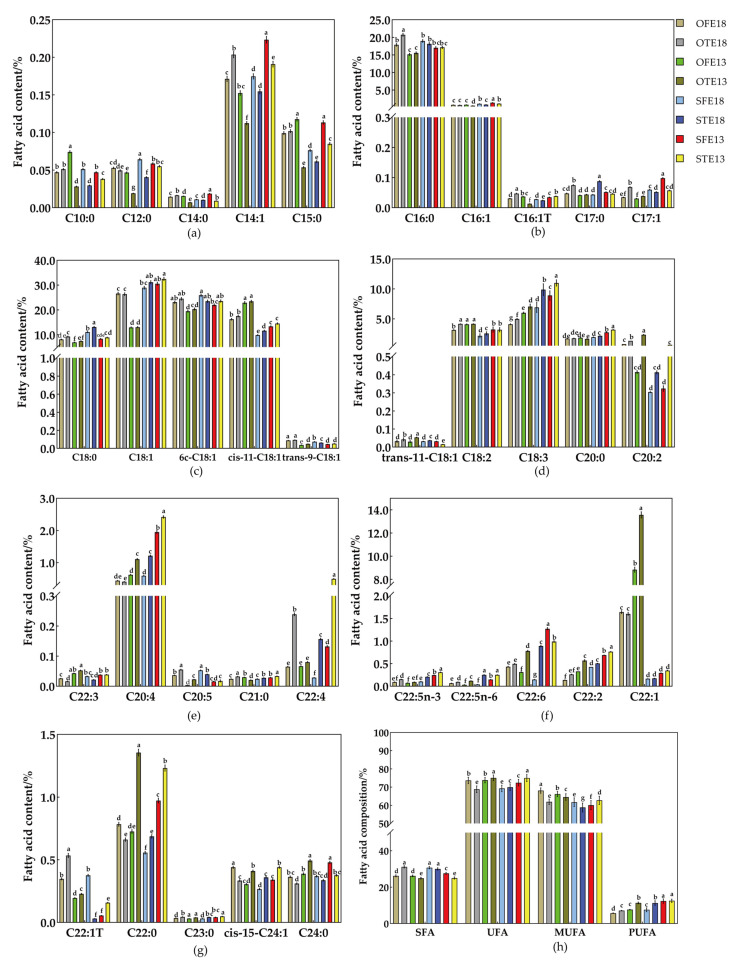
Qualitative and quantitative analyses of medium- and long-chain fatty acids. (**a**–**g**) Content of 35 medium- and long-chain fatty acids in the WTSL and STSL during different developmental stages. (**h**) SFA, UFA, MUFA, and PUFA content in the WTSL and STSL at different nighttime temperatures. OFE18 and OTE18 represent the STSL seeds at the HNT in 35 and 43 DAF, OFE13 and OTE13 represent the STSL seeds at the LNT in 35 and 43 DAF, SFE18 and STE18 represent the WTSL seeds at the HNT in 35 and 43 DAF, and SFE13 and STE13 represent the WTSL seeds at the LNT in 35 and 43 DAF, respectively. C10:0, C12:0, C14:1, C14:0, C15:0, C16:1T, C16:1, C16:0, C17:1, C17:0, C18:2, C18:3, C18:1, 6c-C18:1, cis-11-C18:1, trans-9-C18:1, trans-11-C18:1, C18:0, C20:4, C20:5, C22:3, C20:2, C20:0, C21:0, C22:6, C22:5, C22:5, C22:4, C22:2, C22:1, C22:1T, C22:0, C23:0, cis-15-C24:1, and C24:0 represent capric, lauric, tetradecenoic, myristic, pentadecanoic, palmitelaidic, palmitoleic, palmitic, cis-10-heptadecenoic, heptadecanoic, oleic, linolenic, 6c-octadecenoc, cis-11-octasecenoic, elaidic, trans-11-octasecenoic, stearic, arachidonic, eicosapentaenoic, cis-13,16,19-docosatrienoic, eicosa-11,14-dienoic, arachic, henicosanoic, docosahexaenoic, cis-7,10,13,16-docosatetraenoic, cis-13,16-docosadienoic, erucic, brassidic, behenic, tricosanoic, nervonic, and lignoceric acids, respectively. SFA, UFA, MUFA, and PUFA represent total saturated, unsaturated, monounsaturated, and polyunsaturated fatty acids, respectively. One-way analysis of variance (ANOVA) and Tukey’s post-hoc test were applied to evaluate the significance of fatty acid content changes at different nighttime temperatures. Lowercase letters were deemed to be statistically significant at *p* ≤ 0.05.

A total of 35 fatty acid metabolites were detected in the WTSL and STSL seeds at 35 and 43 DAF, respectively. Fatty acid responses to different nighttime temperatures are shown in Figure 2, Figures S1 and S2. The nighttime temperature sensitivity of the STSL plants was significantly different to that of the WTSL plants. In the STSL, other than for capric, arachic, behenic, and lignoceric acids, the content of SFAs (lauric, myristic, pentadecanoic, palmitic, heptadecanoic, stearic, and henicosanoic acids) (Figure 2a–g) at the high nighttime temperature (HNT, 18 °C) was significantly higher than at the low nighttime temperature (LNT, 13 °C) in the same development period (35 and 43 DAF), and the total SFA (Figure 2h) content at the HNT was significantly higher than at the LNT. The total UFA (Figure 2h) content at the HNT was significantly or insignificantly higher than at the LNT. Monounsaturated fatty acid (MUFA) and polyunsaturated fatty acid (PUFA) content (Figure 2h) at the HNT were significantly higher than at the LNT. Six major fatty acids (palmitic, stearic, oleic, linoleic, linolenic, and erucic acids) accounted for more than 91% (Figure S3a–h) of the total fatty acid content. In the STSL seeds, the oleic acid content at the HNT (58–62%) were clearly higher than at the LNT (49–54%), while the erucic acid content showed the opposite trend (HNT: 1–2%, LNT: 8–13%).

However, in the WTSL seeds, the effect of different nighttime temperatures on the content of 35 fatty acids was smaller than in the STSL seeds (Figure 2a–h). Except for four fatty acids (capric, palmitic, eicosa-11,14-dienoic, and DPAn-6 acids) at the HNT, where the content at the HNT was not significantly different to that at the LNT, five fatty acids (lauric, heptadecanoic, elaidic, trans-11-octadecenoic, and eicosapentaenoic acids) had significantly higher contents at the HNT than at the LNT. The contents of other fatty acids (tetradecenoic, myristic, pentadecanoic, palmitelaidic, palmitoleic, cis-10-heptadecenoic, vaccenic, arachidonic, cis-13,16,19-docosatrienoic, arachic, henicosanoic, docosahexaenoic, DPAn-3, cis-7,10,13,16-docosatetraenoic, cis-13,16-docosadienoate, brassidic, nervonic, and lignoceric acids) were significantly lower at the HNT than at the LNT. The SFA content at the HNT was significantly higher than at the LNT, whereas the PUFA content at the HNT was significantly lower than that at the LNT. The contents of UFAs and MUFAs were not significantly different. Six fatty acids made up more than 93% of the total fatty acid content (Figure S3a–g). The content of SFAs (palmitic and stearic acids) was significantly higher at the HNT than at the LNT, whereas the content of UFAs (linoleic and linolenic acids) was significantly lower at the HNT than at the LNT. The contents of oleic and erucic acids were not significantly different.

In the development stages (35 and 43 DAF, at the same nighttime temperature), the contents of SFAs, UFAs, and MUFAs at 35 DAF were not significantly different to that at 43 DAF; however, the PUFA content at 43 DAF was significantly higher than at 35 DAF. In conclusion, the fatty acid content of the STSL seeds was more significantly affected by nighttime temperature than the WTSL. The content of SFAs and MUFAs were higher (oleic acid content increased) at the HNT than at the LNT, but that of PUFAs was lower.

### 2.3. Transcriptome Analysis of Seeds of at Different Developmental Stages at Different Nighttime Temperatures

**Figure 3:**
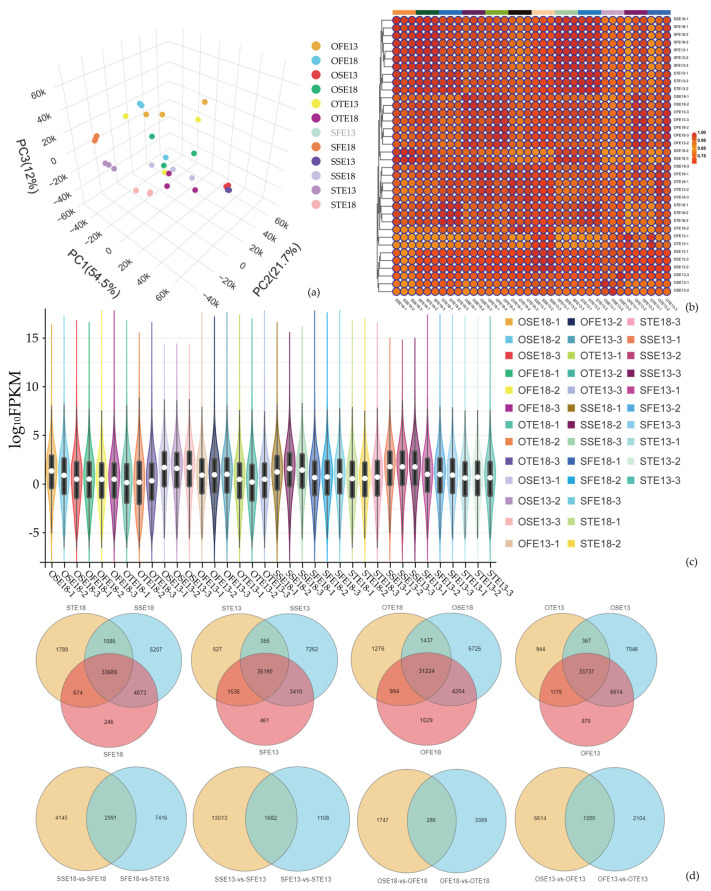
(**a**) Three-dimensional principal component analysis (PCA) score chart of RNA-seq data for all quality control (QC) samples. (**b**) Correlation heat map. Pearson’s correlation coefficient (R^2^) > 0.8 between the three biological replicate samples. (**c**) Venn diagram of differentially expressed genes (DEGs). Abscissa indicates different samples. Ordinate indicates the logarithmic values of the sample expression FPKM. (**d**) Venn diagram of differentially expressed genes (DEGs).

The WTSL and STSL seeds at 27, 35, and 43 DAF were used for RNA-seq at the HNT and the LNT, with three biological replicates each. After filtering the original data, determining the sequencing error rate, and establishing the GC content distribution, 292.49 Gb of clean data were obtained. More than six Gb of clean reads and Q30 > 91% (Table S3) were obtained for each sample. The proportion of the total mapped showed at least 91% similarity with the *B. napus* genome, and the proportion of unique mapped reads was >75%. Thus, the obtained RNA-seq data were accurate and met the requirements for subsequent analysis. A principal component analysis (PCA) analysis was performed on the WTSL and STSL seeds, and the three-dimensional PCA showed good consistency between the three biological replicates (Figure 3a and Figures S4–S7). The fragments per kilobase of transcript per million fragments mapped (FPKM) violins of the sample identity are shown in Figure 3c. The dispersion in the distribution of expression levels for each sample was small. The FPKM range of gene expression was 10^−5^–10^10^, and overall gene expression levels were high. The correlation heatmap (Figure 3b) shows high biological reproducibility for each sample. The correlation between biological replicates in the WTSL and STSL seeds was >0.75. The normalized FPKM expression in the differential gene set was extracted, and a hierarchical cluster analysis was performed. The results showed significant differences in gene expression before nighttime temperature treatment in this experiment (Figure 3c). Thus, these results supported further search into differentially expressed genes (DEGs).

**Figure 4:**
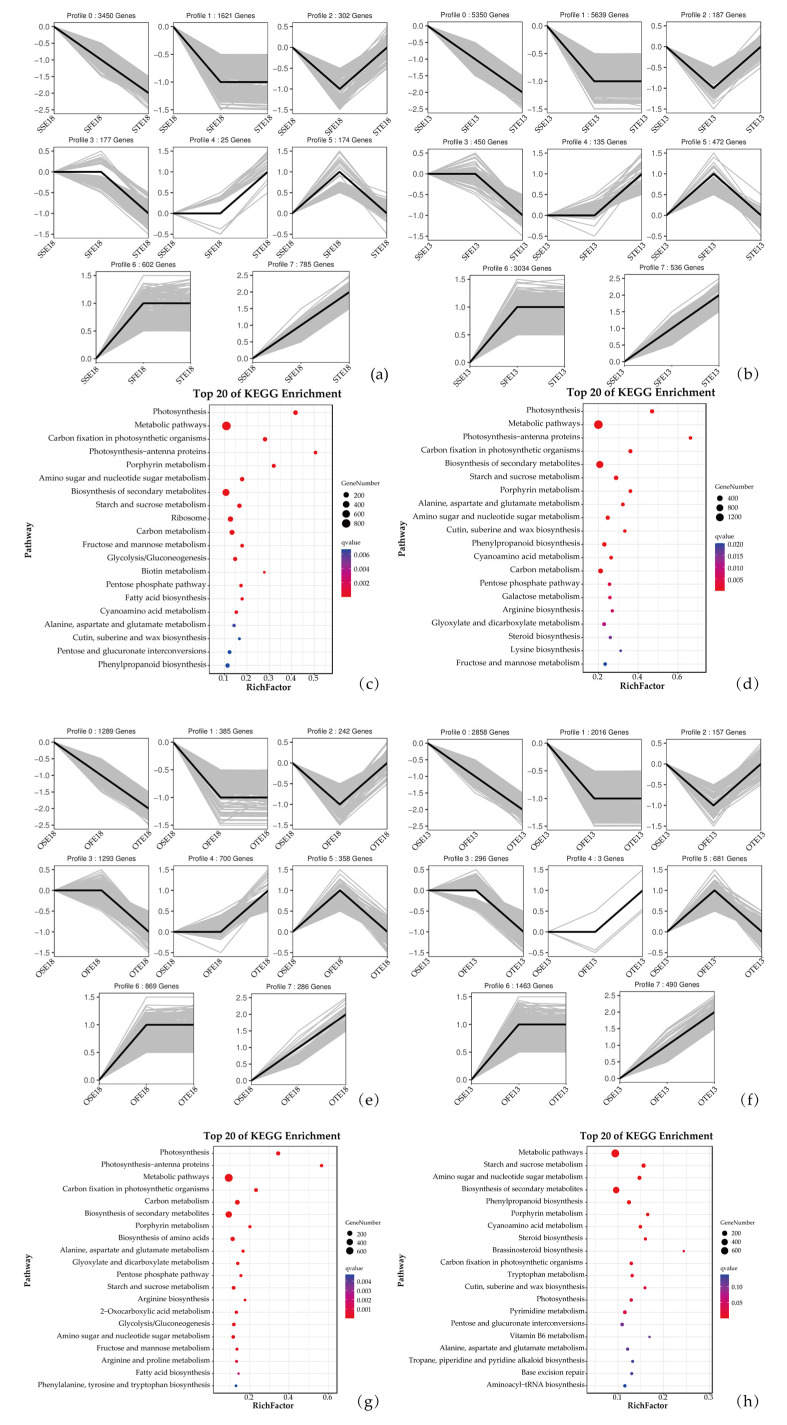
Expression profiles for differentially expressed genes for different nighttime temperature treatments with annotations from the Kyoto Encyclopedia of Genes and Genomes. (**a**,**b**,**e**,**f**) eight representative expression patterns were screened for eight clusters; (**c**,**d**,**g**,**h**) enrichment scatter diagram. Black lines were deemed to the trends of genes expression of STSL and WTSL at different nighttime temperatures.

At different development stages of the WTSL and STSL, DEGs were analyzed using DESeq2, and the total upregulated and downregulated DEGs per group were enumerated (Table 2 and Tables S4–S7). A Venn chart reflected the number of differences between the WTSL and STSL seeds at different nighttime temperatures, and the number of DEGs at the same development stage (Figure 3d). At the HNT, the numbers of total DEGs in the WTSL were 44,054 (27 DAF), 38,436 (35 DAF), and 37,228 (43 DAF); the numbers of specific DEGs were 5208 (27 DAF), 246 (35 DAF), and 1780 (43 DAF); the number of common DEGs was 33,689. The numbers of total DEGs in the STSL were 44,054 (27 DAF), 38,436 (35 DAF), and 37,228 (43 DAF); the numbers of specific DEGs were 5275 (27 DAF), 270 (35 DAF), and 1276 (43 DAF); the number of common DEGs was 31,224. In the WTSL plants grown at the LNT, the numbers of total DEGs were 40,597 (27 DAF), 46,217 (35 DAF), and 37,608 (43 DAF); the numbers of specific DEGs were 7262 (27 DAF), 461 (35 DAF), and 527 (43 DAF); and the number of common DEGs was 35,190. In the STSL plants grown at the LNT, the numbers of total DEGs were 47,764 (27 DAF), 42,396 (35 DAF), and 32,223 (43 DAF); the numbers of specific DEGs were 7024 (27 DAF), 870 (35 DAF), and 944 (43 DAF); and the number of common DEGs was 33,737. Compared with plants grown at the LNT, the total numbers of DEGs in the WTSL plants grown at the HNT were 6862 (27 DAF, 4213 up- and 2649 downregulated), 270 (35 DAF, 199 up- and 71 downregulated), and 4036 (43 DAF, 1095 up- and 2031 downregulated), and the total numbers of DEGs in the STSL plants were 5804 (3042 up- and 2762 downregulated), 1339 (1210 up- and 819 downregulated), and 1534 (1000 up- and 534 downregulated). Combined with the medium- and long-chain fatty acid metabolism, the trend analysis of the RNA-seq data was conducted according to these fatty acid content changes at 35–43 DAF at different nighttime temperatures in the WTSL and STSL seeds, and eight DEG trend profiles (0–7) similar to the trend in variation trend of fatty acids were obtained (Figure 4a,b,e,f). Therefore, the nighttime temperature was more reliable for screening DEGs related to fatty acid synthesis.

### 2.4. Gene Ontology (GO) Functional Annotation and Kyoto Encyclopedia of Genes and Genomes (KEGG) Enrichment Analysis for DEGs at Different Nighttime Temperatures

A GO functional annotation (Figure S8 and Tables S8–S11) and a KEGG enrichment (Figure 4c,d,g,h) analysis were performed on eight trend profiles (0–7) screened according to changes in 35 medium- and long-chain fatty acid metabolites at different development stages to reveal molecular mechanisms relevant to fatty acid accumulations in *B. napus* seeds at different nighttime temperatures. The GO database was used to classify the DEGs into biological processes, molecular functions, and cellular components. At the HNT, the WTSL seeds were enriched for the GO terms GO:0051301 (cell division), GO:0000281 (mitotic cytokinesis), GO:0046983 (protein dimerization activity), GO:0048037 (cofactor binding), GO:0009579 (thylakoid), and GO:0031976 (plastid thylakoid) (Figure S8a), while the STSL seeds were enriched for GO:1990066 (energy quenching), GO:0045229 (external encapsulating structure organization), GO:0046906 (tetrapyrrole binding), GO:0070546 (L-phenylalanine aminotransferase activity), GO:0042575 (DNA polymerase complex), and GO:0031301 (integral component of organelle membrane) (Figure S8b). At the LNT, the WTSL seeds were enriched for GO:1903047 (mitotic cell cycle process), GO:0000281 (mitotic cytokinesis cell), GO:0048037 (cofactor binding), GO:0003824 (catalytic activity), GO:0032993 (protein-DNA complex), and GO:0031976 (plastid thylakoid) (Figure S8c), and the STSL seeds were enriched for GO:0007049 (cell cycle), GO:0000281 (mitotic cytokinesis), GO:0046983 (protein dimerization activity), GO:0005515 (protein binding), GO:0032993 (protein-DNA complex), and GO:0000793 (condensed chromosome) (Figure S8d).

The KEGG enrichment analysis output was plotted as a scatter diagram (Figure 4c,d,g,h and Tables S12–S15), in which the enrichment degree of KEGG was determined based on the gene percentage, *p*-value, and the number of genes in a given pathway. The top20 KEGG enrichment analysis (screening pathway entries with more than two DEGs, each entry sorted by −log10 *p*-value) is shown in Figure 4. At the HNT and LNT, the pathways related to fatty acid metabolism (ko00061: fatty acid biosynthesis; ko00073: cutin, suberine, and wax biosynthesis) were displayed in KEGG top20 terms. In addition, the carbon metabolism processes (ko00020: Citrate cycle; ko00051: fructose and mannose metabolism; ko00010: glycolysis/gluconeogenesis; ko00630: glyoxylate and dicarboxylate metabolism) that provide substrates for fatty acid metabolism were also noted in the KEGG top20 terms.

### 2.5. DEGs in Pathways Involved in and/or Related to Fatty Acid Metabolism Affected by Different Nighttime Temperatures

To understand the effect of different nighttime temperatures on fatty acid metabolism, we focused on DEGs in the pathways that were directly involved in and/or related by fatty acid metabolism. The number and ID of DEGs up- and downregulated by nighttime temperature are shown in Table 3 and Table 4, and Tables S12–S15. At the HNT, the numbers of SE18 vs. SFE18, SFE18 vs. STE18, OSE18 vs. OFE18, and OFE18 vs. OTE18 detected were 165 up- and 785 downregulated, 429 up- and 1190 downregulated, 130 up- and 67 downregulated, and 134 up- and 500 downregulated DEGs, respectively. At the LNT, the numbers of SSE13 vs. SFE13, SFE13 vs. STE13, OSE13 vs. OFE13, and OFE13 vs. OTE13 detected were 368 up- and 1153 downregulated, 85 up- and 324 downregulated, 234 up- and 500 downregulated, and 215 up- and 234 downregulated DEGs, respectively. Compared with plants grown at the HNT, the numbers of SSE18 vs. SSE13, SFE18 vs. SFE13, and STE18 vs. STE13 detected in LNT plants were 389 up- and 261 downregulated, 196 up- and 174 downregulated, and 339 up- and 350 downregulated DEGs, respectively. The numbers of OSE18 vs. OSE13, OFE18 vs. OFE13, and OTE18 vs. OTE13 detected were 312 up- and 337 downregulated, 116 up- and 117 downregulated, and 178 up- and 84 downregulated DEGs, respectively. At the same nighttime temperature, the STSL and WTSL seeds had significantly different numbers of DEGs related to fatty acid synthesis, with the WTSL seeds showing a significantly lower number of fatty acid-related DEGs compared with the STSL seeds (Table 3 and Table 4). A large number of DEGs were found to be involved in fatty acid metabolism and other related metabolic processes (ABC transporters, alpha-linolenic acid metabolism, arachidonic acid metabolism, biosynthesis of unsaturated fatty acids, cutin, suberine, and wax biosynthesis, fatty acid biosynthesis, fatty acid elongation, fatty acid degradation, and fatty acid metabolism). At the same time, the number of DEGs related to the formation of the fatty acid carbon skeleton (carbon metabolism, citrate cycle (TCA cycle), fructose and mannose metabolism, glycolysis/gluconeogenesis, starch and sucrose metabolism, pyruvate metabolism, and oxidative phosphorylation) was also lower. The number of DEGs in plant hormone signal transduction and circadian rhythm plants was also similar.

### 2.6. TFs Related to Metabolism DEGs at Different Nighttime Temperatures

**Figure 5:**
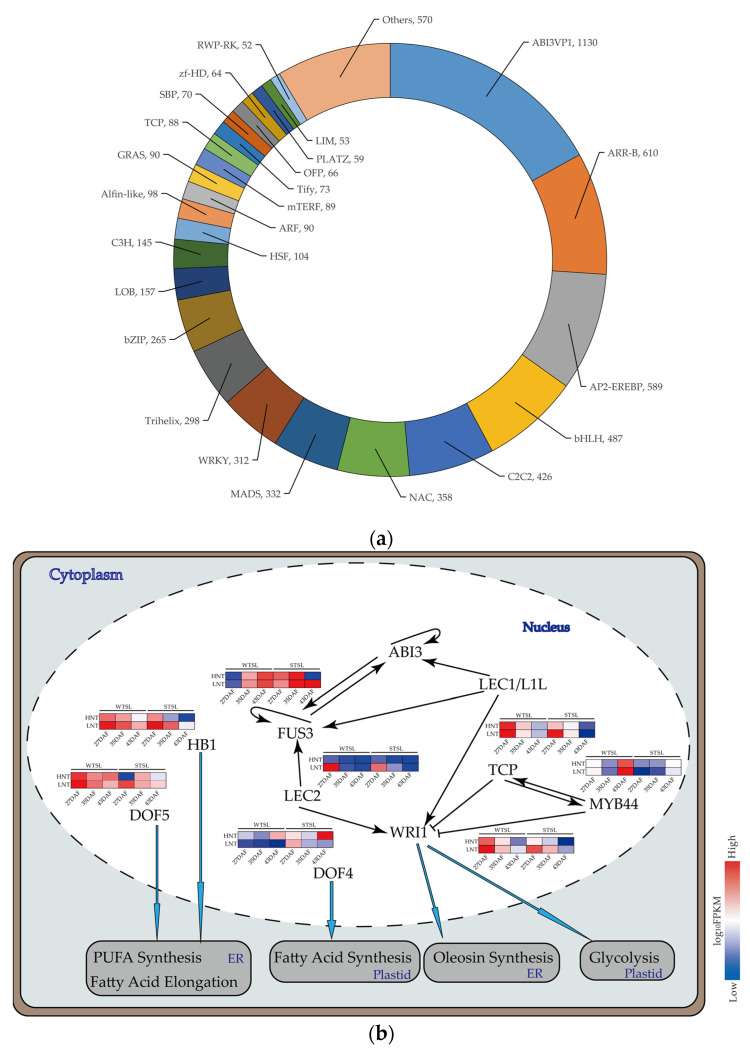
(**a**) Differentially expressed transcription factors (TFs) in different samples. (**b**) Pathways for involvement of TFs in *B. napus* seeds at different nighttime temperatures. ER represents endoplasmic reticulum.

Combined with the GO functional annotation top20 terms, GO:0001071 (nucleic acid-binding transcription factor activity) was enriched in both developing seeds of the WTSL and STSL. Several TFs have been reported to be involved in fatty acid synthesis in *B. napus* seeds [16,23,24]. In this study, 669 differentially expressed TFs were identified in the WTSL and STSL developing seeds (Figure 5a), of which 113 belonged to the ABI3VP3 family of TFs. *ABI3* (LOC106367461), *LEC2* (LOC106356333), *WRI1* (LOC106412050), *DOF5* (LOC106436817), *DOF4* (LOC106436165), *MYB44* (LOC106390324), *TCP* (LOC106383098), and *HB1* (LOC106430193), involved in fatty acid biosynthesis and oil accumulation, were detected in both the WTSL and STSL developing seeds (Figure 5b), and these TFs played vital roles in fatty acid biosynthesis and oil accumulation. At the HNT, the expression level of *WRI1* was higher in the STSL seeds than in the WTSL seeds; however, at the LNT, the expression level was the reverse of that at the HNT. The expression levels of *DOF4* and *ABI3VP1* were not correlated with the nighttime temperature, and their expression levels were higher in the WTSL than in the STSL, whereas the relative expression levels of *TCP* and *MYB44* were opposite to those of *DOF4* and *ABI3VP1*, being higher in the STSL seeds than in the WTSL seeds. The expression levels of *HB1* and *DOF5* in the WTSL and STSL seeds grown at the LNT were higher than in seeds grown at the LNT, and these expression level differences were higher in the STSL than in the WTSL seeds. The changes in the expression levels of nine TFs at the HNT and LNT occurred in parallel with the changes in the oil and fatty acid content of seeds, and expression of these TFs may regulate fatty acid metabolism and oil accumulation in *B. napus* developing seeds at both the HNT and LNT. *DOF5* positively regulates fatty acid elongation and PUFA synthesis [25,26,27], while *HB21* negatively regulates the expression of *SAD*, *FAD2*, and *FAD3* [28]. *HB1* in *B. napus* is homologous to *HB21* in *Arabidopsis thaliana*. The differences in the expression levels of *DOF5* and *HB1* parallel differences in fatty acid content in *B. napus* plants grown at different nighttime temperatures.

### 2.7. RNA-Seq Analyses of Fatty Acid Metabolism at Different Nighttime Temperatures

**Figure 6:**
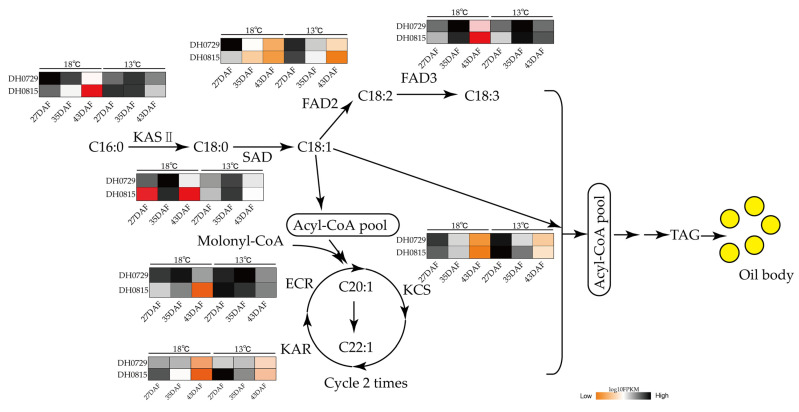
Effects of nighttime temperature on fatty acid metabolism in *Brassica napus* developing seeds.

Based on changes in medium- and long-chain fatty acid metabolites and DEGs related to fatty acid biosynthesis, we mapped the metabolic network from palmitic acid to erucic acid in developing seeds of *B. napus* (Figure 6). At the HNT, the expression levels of *KAS II* (LOC106387251), *SAD* (LOC106372205), *FAD2* (LOC106424191), *FAD3* (LOC106439274), *ECR* (LOC106396280), *KAR* (LOC10649448), and *KCS* (LOC106419344) were higher in the WTSL plants than in the STSL plants. However, at LNT, the expression levels of *KAS II*, *FAD3*, *KCS*, *KAR*, and *ECR* in the STSL were lower than in the WTSL plants. Other genes had similar expression levels at the HNT and LNT. At the HNT and LNT, the expression levels of *KAS II*, *FAD2*, *FAD3*, *ECR*, *KAR*, and *KCS* were slightly higher in the WTSL plants than in the STSL plants, although they were not significantly different, and this is the reason for the slightly increased fatty acid content of plants grown at the LNT. In the STSL plants, although the expression levels of *FAD2* and *FAD3* were only slightly and not significantly higher, the expression levels of *SAD*, *ECR*, *KAR*, and *KCS* were significantly lower. At the LNT, the expression level of *SAD* in the STSL plants was higher than at the HNT, but DEGs related to erucic acid biosynthesis were also upregulated, most of which are used in the synthesis of UFAs (linoleic, linolenic, and erucic acids), especially erucic acid, leading to a decrease in oleic acid content and an increase in erucic acid content. The expression levels of *DOF5* and *HB1* in plants grown at the LNT were increased, and this positively regulated fatty acid chain elongation and promoted accumulation of UFAs, including erucic acid.

### 2.8. Prediction of Cis-Acting Elements in DEG Promoters Using plantCARE

**Figure 7:**
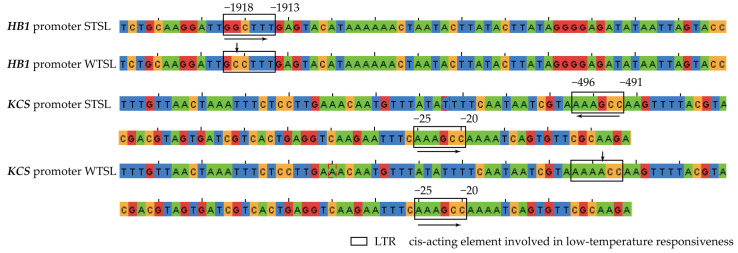
Prediction of cis-acting elements in *HB1* and *KCS* promoter.

In *A. thaliana* mutants, *HB21* plays an important role in oil accumulation and fatty acid elongation. *HB1* is the homologous gene in *B. napus*. *KCS* is the main regulatory gene for the elongation of the C18:1-ACP carbon chain to form erucic acid in seeds and thus is heavily involved in changes in the erucic acid content of seeds. The expression of *HB1* (LOC106430193) and *KCS* (LOC106419344) in seeds grown at the LNT was upregulated as determined by RNA-seq, indicating that these genes are regulated by nighttime temperature. Based on these results, the promoter sequences of the four genes were obtained from the *B. napus* genome database, and their promoter cis-acting elements were predicted by plantCARE. These gene promoters were found to have cis-acting elements involved in LTR. The promoter sequences of *HB1* and *KCS* were cloned from the STSL and WTSL plants (Figure 7, Files S1–S4). In the *HB1* promoter, the LTR element has a base mutation (G-C mutation, −1917 bp) in the WTSL that causes loss of sensitivity to low temperatures (Files S1 and S2). In the *KCS* promoter, a base mutation (A-G mutation, −493 bp) increased the sensitivity of the LTR elements to low temperatures (Files S3 and S4). Therefore, LTR sequence mutations in the WTSL seeds make these genes immune from LNT enhanced sensitivity. As a result, the erucic acid content in the WTSL was not significantly different at the LNT.

RNA-seq analysis and qRT-PCR were performed on randomly selected DEGs to determine the authenticity and reliability of the transcriptome data as well as on DEGs related to fatty acid metabolism and encoding TFs based on qRT-PCR. The RT-qPCR and RNA-seq results were consistent for nine (LOC106436817, LOC106436165, LOC106356333, LOC106372205, LOC106434448, LOC106439274, LOC106439448, LOC106446788, and LOC106387251) of the 13 validated genes. Hence, transcriptome sequencing was reliable (Figure S9).

## 3. Discussion

Temperature is one of the most important factors determining plant distribution. Earlier studies mainly focused on the effects of temperature on yield, phenotype, and abiotic stress in crops [28,29,30]. However, in recent years, the research focus has changed to the influence of temperature on crop quality [31]. Extreme nighttime temperatures lead to changes in cell membrane composition and lipid component (PUFA) storage, which in turn lead to adaptation in plants [5]. PUFAs are synthesized by the fatty acid desaturases FAD2 and FAD3, being dehydrogenated by FAD2 to form linoleic acid, which in turn is dehydrogenated by FAD3 to form linolenic acid. Temperature affects fatty acid saturation through substrate availability [32,33]. Zhou et al. reported that a HNT significantly affected oil content and fatty acid composition in *B. napus* L. seeds by increasing the ratio of stearic acid to oleic acid, result in a decreasing in oil content by nearly 20% [5]. Fatty acid composition is greatly influenced by nighttime temperature and affects oil accumulation and fatty acid metabolism in seeds. Fu et al. showed that when *B. napus* seeds were planted in a high-altitude agroecological area, the oil content increased by 7% and the fatty acid composition also changed, while the expression of 362–443 genes related to carbohydrate metabolism was increased, with *sucrose synthase*, *pyruvate kinase*, and *6-phosphogluconate dehydrogenase* showing the largest changes in expression [8]. In this study, based on the changes in the oleic and erucic acid content in the WTSL and STSL seeds planted at different altitudes, appropriate nighttime temperatures were manually simulated at the same daytime temperature to reveal the effect of nighttime temperature on fatty acid metabolism in *B. napus* seeds. With the decrease in nighttime temperature in the STSL, the oleic acid, MUFA, and UFA content decreased, and the erucic acid and PUFA content increased, while the linoleic and linolenic acid content slightly increased. However, in the WTSL seeds grown at a lower nighttime temperature, the oleic acid, erucic acid, linoleic acid, and linolenic acid contents slightly increased, the PUFA and MUFA content increased, the UFA content decreased, and the PUFA content remained unchanged.

Fatty acid biosynthesis and oil accumulation are regulated by interaction of genetic and environmental factors in *B. napus* seeds [34]. Oleic acid is formed by stearic acid desaturation catalyzed by stearoyl-ACP desaturase (SAD), the key enzyme in oleic acid biosynthesis. Oleic acid is then desaturated twice to form first linoleic acid and then linolenic acids. The activities of these enzymes determine the speed and extent of fatty acid synthesis in seeds [35,36]. After two cycles of carbon chain elongation, oleoyl-CoA is converted into erucoyl-CoA. The enzymes 3-ketoacyl-CoA synthase (KCS), enoyl-CoA reductase (ECR), 3-ketoacyl-CoA reductase (KCR), and 3-hydroxyacyl-CoA dehydrase (HCD) are involved in fatty acid elongation and are key enzymes in erucic acid biosynthesis [37,38,39]. TFs, such as WRI1 [40], LEC1 [41], LEC2 [42], ABI3 [43], DOF11 [44], DOF4 [44], and HB21 [45], are key to fatty acid metabolism and positively regulate glycolysis, fatty acid biosynthesis and elongation, oil accumulation, MUFA biosynthesis, and oil-body assembly. However, other TFs, such as MYB89, MYB67, and TCP, are negative regulators of fatty acid biosynthesis. In *Arabidopsis* seeds, the overexpression of these TFs led to a decrease in oil content [45,46]. In this study, the expression of *SAD*, *KAS*, *ECR*, and *KCS* were upregulated in the STSL, and this may also be responsible for the increased erucic acid content at the LNT. However, the expression level of *SAD* and the oleic acid content in the WTSL was higher than in the STSL. In the tung tree (*Vernicia fordii*), *HB21* negatively regulates the expression of *SAD*, *FAD2*, and *FAD3* [45]. In this study, the expression level for *HB21* was consistent with those of *SAD*, *FAD2*, and *FAD3*, slightly different from what was found in the tung tree, which may be due to the differences in molecular functions of the same molecules in different species. In soybeans [47], DOF11 and DOF4 can activate the *acetyl CoA carboxylase* and *long-chain-acyl CoA synthetase* genes by binding to cis-DNA elements (A/TAAAG or CTTTA/T) in their promoter regions, thereby the expression of upregulated genes is involved in fatty acid elongation and oil accumulation. This in turn leads to an increase in the total fatty acid and oil content. In the present study, the expression of *DOF11* and *DOF4* was upregulated in plants grown at the HNT, and the expression of significant genes was similar to that of *SAD*, *ECR*, *KCS*, and *KAR*. Although the expression of *SAD*, *ECR*, *KCS,* and *KAR* was upregulated, these genes were unable to compensate for oleic acid consumption during the synthesis of erucic acid, causing the oleic acid content to decrease and the erucic acid content to increase. We cloned the *HB1* (LOC106430193) and *KCS* (LOC106419344) promoter sequences and found that the cis-acting regulation of LTR sequences was affected by a base mutation, leading to a loss of function, which in turn reduced the sensitivity response to the LNT in the WTSL. Therefore, base mutations in LTR elements can be selected from the promoter regions of *HB1* and *KCS* to maintain the oleic acid content and to increase erucic acid content in *B. napus* seeds. In cultivation, the oleic acid content of *B. napus* plants could be increased by planting them in agroecological areas with smaller daytime/nighttime differences, for example low altitude areas. However, to obtain a high content of erucic acid, an important fatty acid in industrial production, rapeseed can be planted in agroecological areas with larger daytime/nighttime temperature differences, for example high altitude areas.

## 4. Materials and Methods

### 4.1. Plant Growth and Seed Collection

Two DH lines of *B. napus*, DH0729 (WTSL) and DH0815 (STSL), were obtained from Yunnan Agricultural University (Panlong District, Kunming, China) and used in this study for tests at different altitudes (major climate and environmental factors are shown in Table S1, results in Table S2). During 2020–2021, the WTSL and STSL were first planted in a field pot experiment of Yunnan Agricultural University then transferred to a greenhouse with automatic temperature and sunlight control (Kunming Institute of Botany, Chinese Academy of Sciences, Kunming, China) during the bolting period. These DH lines were subjected to different temperatures between day and night, and the treatment groups were as follows: 20/18 (±0.5 °C, daytime/nighttime temperature), 20/16, 20/13, and 20/10 °C; day 14 h, night 10 h. At the early florescence stage, flower blooming was marked using red wool on different days. After maturation, the fatty acid content of seeds was determined using GC-MS. Based on the results, 20/18 (HNT) and 20/13 °C (LNT) were used in the next step of testing. During 2021–2022, the HNT and LNT were used in a study of nighttime temperature and metabolomics and RNA-seq analyses. After the bolting period, materials were transferred to a greenhouse with automatic temperature and sunlight control. From 27 DAF, flower blooming was marked by red wool every 7 days. The optimal sampling times were 27, 35, and 43 DAF at night [16,48], and seeds were sampled in 3 biological replicates from 3 plants. Each biological replicate was divided into 2 samples, which were used for metabolomics and RNA-seq analyses. Five biological and technical replicates were completed for each treatment, with 10 plants per replicate. Here, SSE18, SFE18, STE18, SSE13, SFE13, and STE13 represent 27, 35, and 43 DAF WTSL seeds in HNT and LNT; OSE18, OFE18, OTE18, OSE13, OFE13, and OTE13 represent 27, 35, and 43 DAF STSL seeds in HNT and LNT.

### 4.2. Fatty Acid Composition Analysis

During the maturation stage, the fatty acid composition of the day and night treatment groups of seeds was determined at different altitudes. Lipid extraction was carried out according to a previously described method [49,50] with slight modifications. A mixed sample of high-temperature thermally permissive lipase (100 mg) was transferred into a 15-mL centrifuge tube after grinding. Subsequently, 2 mL of a 5% methanol solution of hydrochloric acid, 3 mL of chloroform/methanol solution (*v*/*v* 1:1), and 1 L of methyl decanoate internal standard were added. The mixture was placed in a water bath at 90 °C for 1 h. Next, 1 mL of n-hexane was added to the centrifuge tube at room temperature. After extraction by oscillation for 2 min, the sample was allowed to stand for 1 h until the layers separated. The supernatant was brought to 1 mL with *n*-hexane, filtered through a 0.45-μm filter membrane, and tested.

A Thermo Fisher Trace 1310 ISQ (Thermo Fisher Scientific, Waltham, MA, USA) gas chromatography–mass spectrometry (GC–MS) instrument was used to analyze the fatty acid composition and content of the supernatant. The column temperature was programmed as follows: 80 °C for 1 min; increase at a rate of approximately 10 °C/min up to 200 °C, increase at a rate of approximately 5 °C/min to 250 °C, increase at rate of 2 °C/min to 270 °C, hold for 3 min. The inlet temperature was 290 °C. Splitless injection was used, and the process was repeated 3 times. The identities of fatty acids were determined according to the qualitative analysis based on the retention time in the sample, and the relative content of each fatty acid was calculated using a peak area normalization method.

### 4.3. Metabolite Profiling by GC–MS

Seeds collected at 35 and 43 DAF were used for fatty acid extraction. Fatty acids were extracted from powdered seed material at 90 °C using 5 mL acetyl chloride-methanol as the solvent, ands held for 4 h at 250 rpm. Following the ISO 5509-2000 method, fatty acid methyl esters were analyzed using a Thermo Fisher Trace 1300 gas chromatograph (Thermo Fisher Scientific, Waltham, MA, USA)-ISQ 7000 Mass Spectrometry (chromatographic column: DB-5ms, 60 m × 0.25 mm × 0.25 µm). The internal standard was 5 mg/mL C19:0 methyl nonadecanoic acid (Sigma-Aldrich, Darmstadt, Germany). Three biological replicates were used for the seeds sampled at 35 and 43 DAF. Based on the concentrations of the fatty acid standards used, the peak areas for the sample and the internal standard, the sample volume, the content of fatty acid in the sample can be calculated. Fatty acid detection and statistical data recovery were conducted by Guangzhou Genedenovo Biotechnology Co., Ltd. (Gene Denovo, Shenzhen, China).

The saturated fatty acid (SFA), unsaturated fatty acid (UFA), monounsaturated fatty acid (MUFA), and polyunsaturated fatty acid (PUFA) contents were calculated as follows:

SFA = C10:0 + C12:0 + C14:0 + C15:0 + C16:0 + C17:0 + C18:0 + C20:0 + C21:0 + C22:0 + C23:0 + C24:0

UFA = C14:1 + C16:1 + C16:1T + C17:1 + C18:1 + 6c-C18:1 + cis-11-C18:1 + trans-9-C18:1 + trans-11-C18:1 + C18:2 + C18:3 + C20:2 + C22:3 + C20:4 + C20:5 + C22:4 + C22:5n – 3 + C22:5n – 6 + C22:6 + C22:2 + C22:1 + C22:1T + cis-15-C24:1

MUFA = C14:1 + C16:1 + C16:1T + C17:1 + C18:1 + 6c-C18:1 + cis-11-C18:1 + trans-9-C18:1 + trans-11-C18:1 + C22:1 + C22:1T + cis-15-C24:1

PUFA = C18:2 + C18:3 + C20:2 + C22:3 + C20:4 + C20:5 + C22:2 + C22:4 + C22:5n − 3 + C22:5n – 6 + C22:6

### 4.4. RNA Extraction, Illumina Sequencings, and Data Analysis

Total RNA was extracted from approximately 0.3 g seeds using TRIzol reagent kits (Invitrogen, Carlsbad, CA, USA), and RNA quality was assessed using an Agilent 2100 Bioanalyzer (Agilent Technologies Inc., Palo Alto, CA, USA) under enrichment with Oligo(dT) beads according to the manufacturer’s instructions. The cDNA library (total cDNA ≥ 0.1 μg) was prepared using the NEBNext Ultra RNA Library Prep Kit for Illumina (NEB #7530, New England Biolabs, Springfield, MA, USA), and sent for Illumina Novaseq6000 sequencing at Gene Denovo Biotechnology Co., to obtain 100 bp paired-end sequencing reads. To get high-quality, clean reads, the reads were further filtered by Fastp v. 0.19.3 (https://github.com/OpenGene/fastp, accessed on 15 July 2022), and unknown nucleotides (N) and low-quality reads were removed. An index of *B. napus* genome (ncbi_GCF_000686985.2) [51] was built and paired-end clean reads were mapped to the reference genome using HISAT-3N (rapid and accurate alignment of nucleotide conversion sequencing reads with HISAT-3N) to obtain location data or genes as well as unique sample sequence feature information to obtain mapped data. Based on the *B. napus* genome, mapped reads from each sample were assembled using StringTie v1.3.4, and gene expression levels were quantified and compared with featureCounts v.1.6.2 in R. An FPKM (fragment per kilobase of transcript per million mapped reads) value was calculated to quantify expression abundance and variations using RNA-seq and expectation maximization (RSEM) software that provides accurate transcript quantification from RNA-Seq data with or without a reference genome). Differential expression between the 2 groups was assessed using the DESeq2 (https://bioconductor.org/packages/release/bioc/html/DESeq2.html, accessed on 27 July 2022) software. Genes with a false discovery rate (FDR) ≤ 0.05 and |log2 fold change| ≥ 1 were considered DEGs. DEGs were functionally annotated using the KEGG (Kyoto Encyclopedia of Genes and Genomes, https://www.kegg.jp/, accessed on 26 August 2022), GO (Gene Ontology, http://geneontology.org/, accessed on 27 August 2022), and KOG (Cluster of Orthologous Groups for Enkaryotic Complete Genomes) databases using Blast 2.2.28+.

### 4.5. Quantitative Real-Time PCR (qRT-PCR)

To verify the reliability of genes encoding differentially expressed proteins, genes were subjected to qRT-PCR, which was performed in a 96-well plate on a CFX96 Touch Real-Time PCR system (Bio-Rad, Hercules, CA, USA). The *BnActin7* gene was used as an internal standard for qRT-PCR. The thermal conditions were 95 °C for 10 s, followed by 40 cycles of 95 °C for 10 s, 60 °C for 30 s, and 72 °C for 15 s. Subsequently, gene-specific primers (https://www.ncbi.nlm.nih.gov/tools/primer-blast, accessed on 10 October 2022) were designed based on the results of multiple sequence alignments (Table S16). The relative expression levels of selected genes encoding DEGs were calculated using the 2^−∆∆CT^ method.

### 4.6. Cloning of Cis-Acting Elements in HB1 and KCS Promoters and Cis-Acting Elements of Promoters Using plantCARE

The promoter sequences of *HB1* and *KCS* were obtained from *the B. napus* genomic data using SPDEv1.2 [52]. Cis-acting elements of these promoters were predicted using plantCARE (http://bioinformatics.psb.ugent.be/webtools/plantcare/html/, accessed on 23 October 2022). According to the reported nucleotide sequence of the *HB1* (LOC106430193) and *KCS* (LOC106419344) promoters in the *B. napus* L. genome, the Primer Premier 6.0 software was used to design primers based on the highest homology to *B. napus* L. sequence (Table S17) to amplify the promoter sequence. The cDNAs of the WTSL and STSL were used as templates for RT-PCR, with the following PCR amplification program: pre-denaturation at 95 °C for 10 min, denaturation at 95 °C for 30 s, annealing at 50 °C for 30 s, 20 cycles of extension at 72 °C for 60 s, and 30 cycles of extension at 72 °C for 10 min. The RT-PCR products were stored at 4 °C for further analysis. Subsequently, the RT-PCR products were purified and sent to Sangon Biotech (Sangon Biotech (Shanghai) Co., Ltd., Shanghai, China) for sequencing.

### 4.7. Statistical Analysis

Microsoft Office 2019 (Microsoft, Washington, DC, USA) and GraphPad Prism 8.0 (GraphPad Software Inc., San Diego, CA, USA) were used for the statistical analyses and data plotting for the fatty acid composition of seeds. Heatmap analysis and PCA were performed using the Omicshare tools (https://www.omicshare.com/, accessed on 10 October 2022).

## 5. Conclusions

In this study, two DH lines (DH0729, WTSL) and (DH0815, STSL) of *B. napus* were planted at five different altitudes. In the STSL, the oleic and erucic acid content in mature seeds showed significant changes with altitude; the higher the altitude, the lower the oleic acid content and the higher the erucic acid content. However, similar changes in fatty acid content were not obvious in the WTSL. A temperature-controlled chamber was used to establish different nighttime temperature gradients (daytime/nighttime temperature, 20/18, 20/16, 20/13, and 20/10 °C) combined with the differing growth period; 20/13 °C was used in this study. The nighttime temperature tests were conducted using precision temperature-controlled rooms at the HNT and LNT. The content of 35 medium- and long-chain fatty acid metabolites was determined and RNA-seq analysis was carried out. The total oleic acid content (oleic acid, 6c-octadecenoic acid, cis-11-octasecenoic acid, elaidic acid, and trans-11-octasecenoic acid) at 35 and 43 DAF was significantly lower in the LNT plants than in the HNT plants (58–62%, LNT: 49–54%, an average decrease of 9%), and the total erucic acid content (erucic and brassidic acids, an average increase of 1–2%) was significantly lower at the HNT than at the LNT (8–13%), representing an average increase of 10%. At the LNT, the saturated fatty acid (SFA) content decreased, the polyunsaturated fatty acid (PUFA) content increased, and the monounsaturated fatty acid (MUFA) content decreased in the WTSL. RNA-seq analysis showed that the expression levels of *SAD* (LOC106366808), *ECR* (LOC106396280), *KCS* (LOC106419344), and *KAR* (LOC106367337) in the STSL seeds increased with increasing temperatures. Expression levels for these genes in the WTSL were slightly increased. While the expression level of *SAD* in the STSL seeds increased, the synthesis of erucic acid increased sharply, but the oleic acid content decreased. Transcription factor genes *HB1* (LOC106430193) and *DOF1* (LOC111211868), which are related to oil accumulation and fatty acid synthesis, were upregulated. The *KCS* and *HB1* promoters were cloned, and cis-acting elements involved in low-temperature responsiveness (LTR) were predicted to exist in the *KCS* and *HB1* promoters in both the STSL and WTSL. The *KCS* and *HB1* LTR elements showed sequence differences between the WTSL and the STSL. The altered promoter sequence increased the effects of an LTR element in the STSL, and this may be responsible for the sensitivity to nighttime temperatures in the STSL. Conversely, the LTR for these gene promoters in the WTSL renders it insensitive to nighttime temperatures.

## Figures and Tables

**Table 1:** Effects of development period at different nighttime temperatures.

	Daytime/Nighttime Temperature/°C	Sowing to Emergence	Emergence to Bolting	Bolting to Beginning of Flowering	Beginning of Flowering to Full Flowering Stage	Full Flowering to the Final Flowering	Final Flowering to Maturing	Whole Growth Period
WTSL	20/18	5.00 ± 1.00 a	82.00 ± 1.00 a	5.33 ± 0.58 c	8.00 ± 1.00 c	14.00 ± 1.00 c	35.00 ± 0.58 c	147.33 ± 4.73 d
	20/16	4.67 ± 0.58 a	82.33 ± 1.15 a	7.00 ± 1.00 c	9.00 ± 1.00 c	15.00 ± 1.00 c	36.67 ± 1.53 c	154.67 ± 3.21 c
	20/13	4.67 ± 0.58 a	82.00 ± 1.00 a	10.00 ± 1.00 b	11.00 ± 1.00 b	20.00 ± 1.00 b	45.00 ± 1.00 b	172.67 ± 2.08 b
	20/10	4.67 ± 0.58 a	82.00 ± 1.00 a	12.00 ± 1.00 a	14.00 ± 1.00 a	24.00 ± 1.00 a	51.33 ± 0.58 a	188.00 ± 3.00 a
	Mean	4.75	82.08	8.58	10.50	18.25	42.00	165.67
	SD	0.17	0.17	2.99	2.65	4.65	7.61	18.30
	CV/%	3.51	0.20	34.79	25.20	25.46	18.11	11.05
STSL	20/18	4.67 ± 0.58 a	81.00 ± 1.00 a	7.00 ± 1.00 c	7.00 ± 1.00 d	15.00 ± 1.00 d	32.33 ± 0.58 d	149.00 ± 3.46 d
	20/16	4.67 ± 0.58 a	80.00 ± 1.00 a	8.00 ± 1.00 c	9.33 ± 0.58 c	18.00 ± 1.00 c	36.67 ± 1.15 c	156.33 ± 0.58 c
	20/13	5.00 ± 1.00 a	81.00 ± 1.00 a	11.00 ± 1.00 b	12.33 ± 0.58 b	24.33 ± 0.58 b	42.00 ± 1.00 b	175.00 ± 1.73 b
	20/10	4.67 ± 0.58 a	82.00 ± 1.00 a	14.33 ± 0.58 a	14.00 ± 1.00 a	40.00 ± 1.00 a	46.33 ± 1.53 a	203.00 ± 1.73 a
	Mean	4.75	81.00	10.00	10.50	24.25	39.33	170.83
	SD	0.17	0.82	3.16	3.11	11.15	6.12	24.08
	CV/%	3.51	1.01	31.62	29.61	45.97	15.55	14.09

One-way analysis of variance (ANOVA) and Tukey’s post-hoc test were applied to evaluate the significance of differences in the development period as a function of nighttime temperature. Lowercase letters were deemed to be statistically significant at *p* ≤ 0.05.

**Table 2:** Differentially expressed genes (DEGs) in the different treatment groups.

Group Comparisons	Total No. of Significantly DEGs	Total No. of Significantly Upregulated DEGs	Total No. of Significantly Downregulated DEGs
SSE18 vs. SFE18	7136	1604	5532
SFE18 vs. STE18	10,407	4296	6111
SSE13 vs. SFE13	14,695	3928	10,767
SFE13 vs. STE13	2790	795	1995
SSE18 vs. SSE13	6862	4213	2649
SFE18 vs. SFE13	270	199	71
STE18 vs. STE13	4036	1905	2131
OSE18 vs. OFE18	2033	1230	803
OFE18 vs. OTE18	3675	1166	2509
OSE13 vs. OFE13	7964	2823	5141
OFE13 vs. OTE13	3454	1460	1994
OSE18 vs. OSE13	5804	3042	2762
OFE18 vs. OFE13	1939	1120	819
OTE18 vs. OTE13	1534	1000	534

**Table 3:** Differentially expressed genes in pathways involved in and/or related to fatty acid metabolism from the WTSL at the LNT and HNT.

Pathway	SSE18 vs. SFE18		SFE18 vs. STE18		SSE13 vs. SFE13		SFE13 vs. STE13		SSE18 vs. SSE13		SFE18 vs. SFE13		STE18 vs. STE13	
	Up	Down	Up	Down	Up	Down	Up	Down	Up	Down	Up	Down	Up	Down
ABC transporters	2	6	4	10	4	16	2	2	3	0	0	0	0	2
Alpha-linolenic acid metabolism	6	8	10	29	3	15	5	4	14	6	3	1	7	12
Arachidonic acid metabolism	0	6	4	8	0	12	0	0	6	1	1	0	5	1
Biosynthesis of unsaturated fatty acids	1	4	8	17	3	4	1	0	1	7	0	0	1	6
Carbon metabolism	11	134	29	180	38	159	6	63	33	25	0	1	68	37
Circadian rhythm—plant	1	4	2	8	3	6	0	4	7	4	0	0	1	2
Citrate cycle (TCA cycle)	2	13	9	26	10	16	1	1	4	7	0	0	7	6
Cutin, suberine, and wax biosynthesis	20	1	20	14	33	8	8	0	4	15	2	0	0	11
Fatty acid biosynthesis	2	28	10	38	2	19	0	1	4	7	0	0	5	9
Fatty acid degradation	9	10	13	29	14	15	5	1	6	2	1	0	3	16
Fatty acid elongation	2	14	5	29	5	30	1	4	10	5	0	0	4	5
Fatty acid metabolism	3	30	29	34	7	27	1	1	7	9	0	0	5	14
Fructose and mannose metabolism	1	44	20	30	5	46	1	15	13	5	2	1	12	6
Glycerolipid metabolism	11	18	20	31	21	41	3	6	14	11	3	0	6	9
Glycerophospholipid metabolism	6	12	22	29	20	44	4	4	11	11	2	0	9	11
Glycolysis/Gluconeogenesis	8	63	17	75	15	73	4	17	15	19	1	2	22	16
Glyoxylate and dicarboxylate metabolism	5	39	27	65	18	50	1	22	14	7	1	0	26	22
Indole alkaloid biosynthesis	2	3	3	7	3	6	1	4	4	1	1	0	1	0
Linoleic acid metabolism	0	1	2	8	1	3	1	0	6	1	1	0	4	1
Oxidative phosphorylation	5	30	5	26	9	49	3	11	10	4	1	1	6	5
Phosphatidylinositol signaling system	5	9	3	20	7	35	4	5	11	4	0	0	6	2
Photosynthesis	1	109	25	100	5	71	1	89	1	7	0	1	68	2
Plant hormone signal transduction	18	52	25	150	55	119	10	17	65	43	3	1	12	55
Propanoate metabolism	0	10	2	20	2	11	0	1	3	0	0	0	4	1
Protein processing in endoplasmic reticulum	6	29	20	62	10	100	8	8	37	5	1	1	5	40
Pyruvate metabolism	5	21	24	32	21	31	2	4	9	5	1	0	9	12
Starch and sucrose metabolism	19	74	39	95	26	121	10	34	65	25	7	0	28	20
Tryptophan metabolism	14	13	32	18	28	26	2	6	12	25	2	0	15	27
Total	165	785	429	1190	368	1153	85	324	389	261	33	8	339	350

**Table 4:** Differentially expressed genes in pathways involved in and/or related to fatty acid metabolism from the STSL at the LNT and HNT.

Pathway	OSE18 vs. OFE18		OFE18 vs. OTE18		OSE13 vs. OFE13		OFE13 vs. OTE13		OSE18 vs. OSE13		OFE18 vs. OFE13		OTE18 vs. OTE13	
	Up	Down	Up	Down	Up	Down	Up	Down	Up	Down	Up	Down	Up	Down
ABC transporters	1	0	1	1	1	4	2	3	2	1	1	1	0	3
Alpha-linolenic acid metabolism	3	1	2	7	6	7	5	4	4	6	1	2	9	3
Arachidonic acid metabolism	1	1	2	4	1	6	0	2	2	0	1	1	0	1
Biosynthesis of unsaturated fatty acids	5	0	0	10	1	0	0	2	0	2	0	0	3	1
Carbon metabolism	20	9	6	57	15	76	16	50	40	36	20	10	21	11
Circadian rhythm—plant	2	0	4	4	7	4	0	0	5	5	1	0	1	0
Citrate cycle (TCA cycle)	5	1	4	14	7	11	5	0	7	14	0	4	6	3
Cutin, suberine, and wax biosynthesis	0	0	0	2	19	1	28	1	3	28	2	3	7	0
Fatty acid biosynthesis	5	1	2	18	1	0	5	4	5	4	1	7	6	0
Fatty acid degradation	2	0	4	5	4	1	12	2	4	11	1	4	8	2
Fatty acid elongation	2	1	1	7	2	13	8	5	12	10	2	3	8	0
Fatty acid metabolism	6	1	3	26	3	3	5	4	4	7	4	1	9	1
Fructose and mannose metabolism	2	3	3	20	1	21	2	9	10	4	5	9	5	4
Glycerolipid metabolism	8	0	4	9	10	19	15	4	16	14	2	8	8	4
Glycerophospholipid metabolism	3	1	8	8	13	17	19	5	11	14	2	7	9	2
Glycolysis/Gluconeogenesis	8	0	8	41	7	29	9	11	14	13	6	6	10	5
Glyoxylate and dicarboxylate metabolism	3	6	11	32	9	28	12	16	13	21	6	6	11	5
Indole alkaloid biosynthesis	0	1	0	3	1	2	0	2	1	1	1	0	1	0
Linoleic acid metabolism	1	0	0	2	3	3	1	1	3	3	0	2	3	1
Oxidative phosphorylation	9	1	2	14	3	36	2	7	25	4	3	3	1	1
Phosphatidylinositol signaling system	4	4	2	4	9	6	1	3	11	5	9	0	1	0
Photosynthesis	3	16	0	78	1	33	2	66	6	5	18	2	21	0
Plant hormone signal transduction	6	12	12	23	45	47	25	34	24	43	14	8	8	5
Propanoate metabolism	3	1	0	7	0	3	2	1	5	3	2	2	1	0
Protein processing in endoplasmic reticulum	12	2	23	31	18	40	6	5	22	13	3	4	4	14
Pyruvate metabolism	6	2	8	16	6	9	11	1	9	19	2	7	5	4
Starch and sucrose metabolism	6	3	12	45	22	65	12	19	44	25	8	11	10	6
Tryptophan metabolism	4	0	12	12	19	16	10	4	10	26	1	6	2	8
Total	130	67	134	500	234	500	215	265	312	337	116	117	178	84

## Data Availability

Not applicable.

## Supplementary Materials

The following supporting information can be downloaded at: https://www.mdpi.com/article/10.3390/plants12020325/s1. Figure S1: Relative retention time and intensity of 35 and 43 DAF medium- and long-chain fatty acid metabolites of WTSL and STSL in LNT. Figure S2: Relative retention time and intensity of 35 and 43 DAF medium- and long-chain fatty acid metabolites of WTSL and STSL in HNT. Figure S3: Analyses of major fatty acid contents based on medium- and long-chain fatty acid metabolites. Figure S4: Principal component analysis (PCA) score chart of the RNA-seq data of STSL in LNT. Figure S5: Principal component analysis (PCA) score chart of the RNA-seq data of STSL in HNT. Figure S6: Principal component analysis (PCA) score chart of the RNA-seq data of WTSL in LNT. Figure S7: Principal component analysis (PCA) score chart of the RNA-seq data of WTSL in HNT. Figure S8: GO functional annotation of DEGs of in 27, 35 and 43 DAF seeds in WTSL and STSL in LNT and HNT. Figure S9: Comparison between qRT-PCR and RNA-seq results for DEGs caused by LNT and HNT. Table S1: Major climate and environmental factors during growth period. Table S2: Effect of different altitudes on oil content and fatty acid composition in *B. napus* L. Table S3: Reads number based on the RNA-Seq data. Table S4: Analysis of DEGs from WTSL in HNT. Table S5: Analysis of DEGs from WTSL in LNT. Table S6: Analysis of DEGs from STSL in HNT. Table S7: Analysis of DEGs from STSL in LNT. Table S8: GO functional annotation on SSE18 vs. SFE18 vs. STE18. Table S9: GO functional annotation on SSE13 vs. SFE13 vs. STE13. Table S10: GO functional annotation on OSE18 vs. OFE18 vs. OTE18. Table S11: GO functional annotation on OSE13 vs. OFE13 vs. OTE13. Table S12: KEGG enrichment on WTSL in HNT. Table S13: KEGG enrichment in WTSL in LNT. Table S14: KEGG enrichment on STSL in HNT. Table S15: KEGG enrichment in STSL in LNT. Table S16: The primer design of genes. Table S17: The primer design of *HB1* and *KCS* promoters. File S1: Predicted LTR elements in *HB1* promoter from STSL by plantCare. File S2: Predicted LTR elements in *HB1* promoter from WTSL by plantCare. File S3: Predicted LTR elements in *KCS* promoter from STSL by plantCare. File S4: Predicted the LTR elements in *KCS* promoter from WTSL by plantCare.
