# Supplementary material for: Effects of Low Nighttime Temperature on Fatty Acid Content in Developing Seeds from Brassica napus L. Based on RNA-Seq and Metabolome
Source: Plants (Basel). 2023 Jan 10;12(2):325. doi: 10.3390/plants12020325 (PMC9862530; doi:10.3390/plants12020325)
Supplement: Supplementary file 1 [file plants-12-00325-s001.zip › Figure S2.pdf]

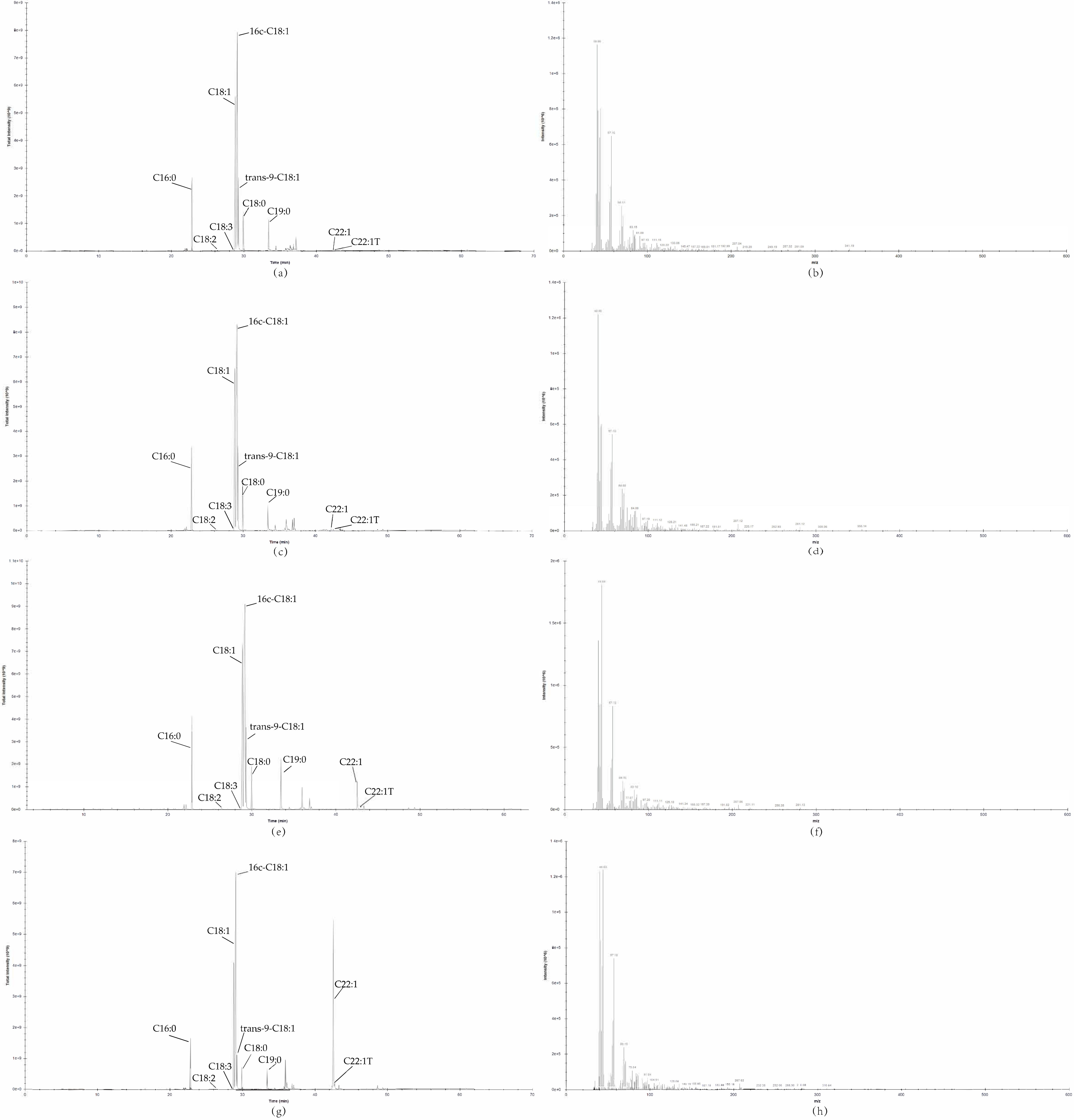

Figure S2. (a, c) and (e, g) Relative retention time of 35 and 43DAF medium- and long-chain fatty acid metabolites of WTSL and STSL in LNT. (b, d) and (f, h) Intensity of 35 and 43 DAF medium- and long-chain fatty acid meta-bolites of WTSL of STSL in LNT.
