# Supplementary material for: Effects of Low Nighttime Temperature on Fatty Acid Content in Developing Seeds from Brassica napus L. Based on RNA-Seq and Metabolome
Source: Plants (Basel). 2023 Jan 10;12(2):325. doi: 10.3390/plants12020325 (PMC9862530; doi:10.3390/plants12020325)
Supplement: Supplementary file 1 [file plants-12-00325-s001.zip › Figure S9.pdf]

RNA-seq data

	STSL			WTSL		
	OSE18vs.OSE13	OFE18vs.OFE13	OTE18vs.OTE13	SSE18vs.SSE13	SFE18vs.SFE13	STE18vs.STE13
LOC106436817	0.72	2.20	1.97	0.18	0.43	1.67
LOC106436165	1.53	1.78	2.73	0.56	0.87	0.61
LOC106390324	-1.79	0.04	-1.60	0.09	-0.62	-0.73
LOC106356333	1.86	3.50	3.10	2.25	0.78	1.62
LOC106383445	1.65	1.85	1.34	0.58	0.82	1.69
LOC106372205	1.07	0.54	1.33	-0.16	0.18	0.72
LOC106434448	1.73	1.59	1.38	0.53	0.18	0.77
LOC106439274	1.16	0.37	1.64	0.10	0.81	0.82
LOC106439448	1.08	0.83	1.27	0.02	0.04	0.89
LOC106446788	1.92	1.35	1.03	0.74	0.29	0.44
LOC106396280	0.80	0.35	1.59	0.08	0.23	0.73
LOC106387251	1.03	1.55	2.02	0.75	0.45	0.02

qRT-PCR data

	STSL			WTSL		
	OSE18vs.OSE13	OFE18vs.OFE13	OTE18vs.OTE13	SSE18vs.SSE13	SFE18vs.SFE13	STE18vs.STE13
LOC106436817	2.64	1.68	1.61	-0.56	0.86	1.42
LOC106436165	1.07	4.35	2.27	1.16	1.19	0.18
LOC106390324	1.03	1.12	1.52	-1.19	1.23	0.36
LOC106356333	7.03	1.07	2.78	-1.78	1.00	4.16
LOC106383445	0.15	0.59	1.00	-0.53	8.41	0.20
LOC106372205	7.02	2.00	3.75	-1.78	1.12	1.11
LOC106434448	1.48	8.09	1.01	-0.23	0.13	1.12
LOC106439274	1.32	0.33	0.73	-0.82	1.39	2.55
LOC106439448	1.47	5.92	0.89	0.30	0.10	0.51
LOC106446788	7.72	1.31	6.58	0.72	1.04	0.59
LOC106396280	1.73	1.02	0.80	-0.27	1.13	2.25
LOC106387251	4.44	13.68	1.85	0.27	1.36	1.28

Figure S9. Comparison between qRT-PCR and RNA-seq results for DEGs caused by LNT and HNT.
