# Supplementary material for: Effects of Low Nighttime Temperature on Fatty Acid Content in Developing Seeds from Brassica napus L. Based on RNA-Seq and Metabolome
Source: Plants (Basel). 2023 Jan 10;12(2):325. doi: 10.3390/plants12020325 (PMC9862530; doi:10.3390/plants12020325)
Supplement: Supplementary file 1 [file plants-12-00325-s001.zip › File S1.html]

Content-Type: text/html; charset=ISO-8859-1


PlantCARE


Webmaster Firefox specific output  
To save the result:
click on the frame with the right mouse button and save the source code as a text file with extension .html  
REFERENCE:PlantCARE: a database of plant cis-acting regulatory elements and a portal to tools for in silico analysis of promoter sequences.  
Lescot, M., Déhais, P., Moreau, Y., De Moor, B., Rouzé ,P.,and Rombauts, S.  
Nucleic Acids Res., Database issue(2002), 30(1):325-327.   


---

>PlantCARE\_25378   
+ ATAAAGTTGT CGTGATTTCT TGCCCTTTAT TTTTTATTTA TATTAACATA TATTTTTAAA AAAAAATTAA   
  
  
+ AGACGTTCCT AACCGAAACT CATGTATTTT TTGATTATGA ATATGAATAT CCCCTCTATA TTAATCATGG   
  
  
+ AGCATTACAA CATGTTTTCG TAGCCATATG TCATCACGAG AATGATTTTT AGAATTGTTA GAAAAATAAA   
  
  
+ TTGATTCATA TAAACATATA CTATGTTTTT TATTAAACTA ACTATCAAAT TAATTAATAG TGTACAAAAA   
  
  
+ AATATTTTTT TCTTTCCTTA AATAAAAACT ACGGAATTAC CTAATATGGC TAACATATAT ATGACAATTA   
  
  
+ ATGATTATGA ATAATACATA TTTGATAAAA AAATTTCTAA CCTCTCTCTT TTTTGTTTAA TTTTATATTA   
  
  
+ TTAAAGGAAA TTTAACAATC ACATTAATCA TATAATAAAA ACAATTAGAT TTTTTCTTAT ATGTTATATT   
  
  
+ TTGAATTTTT AAAAACGACT ATAAATTACT AAAAATGATA AGAGTCCCAC ATTAAAAAAT TTGTGATCAA   
  
  
+ CCGTTTAACT TTTTTTTTAG TTCAAGCAAG ATACAAATGA TCATATATCT GATATAGACG TGGGCGTTCG   
  
  
+ GATACACGTT CGGGTTTGTA TCAGATATTT CAGTATAAAG GTATAGAACC CGTTCGGGTA TTTCTACACT   
  
  
+ CCGAGTCGGG TTCGGGTTCG GATATTTTGG ATCGGGTTCG AATATTTAAA TTTTGAAGAA AAAAAGAAAT   
  
  
+ TATTCACTGT TTAAGTTTTT TATATTTAAA TATATCTTAA CTTAACTGAT TTTTTTTAGT TTTTAAAAGA   
  
  
+ TTAAAATATT AATATGTTTG GAGATAAAAC TTTAAAAATA GAAAGACACT AATTTAGTTT TTGTTTTGAA   
  
  
+ AATTTAGATG CAACTTTTGT TAATGCAAGA AACAAGAACT TGATATGTAT TTTAAGTGAG TAACAAATGA   
  
  
+ TTTTGTCTAT AGTTATATGT ATATTATCTA ATTTTGAGTA ATAAGAATCA TTAATATAAA TATTTTGAAT   
  
  
+ AAAATTAGAT AGATAAACTA TAAATATAGA GTTAAGTATA CTTATGTTTG GTTATCTTCG AATATTACCC   
  
  
+ GTTCGGATAT ATTATCTGAA CTGGTGAAAT AAGTAATATG TTTTGTTGTT TTAATTAGAT AATTTTTAGA   
  
  
+ CCGAGCTTGT GAATATATAC TAGACAAACA TTTATATTTC GAGTCTGCAC TTATATTCTA TAAGAGCTTG   
  
  
+ ATATATTAGA TTTGAACACT AACCTGTTAA TATAGTTTGC CGGTGATTTT TTTTCAAAAT TTTGATTCTT   
  
  
+ AGATATGTAT ATGGAGTAAA ACTAATTTTT ACAGATGCCC ATTTTTTTAA TTGACACTTA TGTAATTAAC   
  
  
+ TGAATTCATA AAACAAGGTT TTTTAAAAAA ATTTAACTCA TATCAATGAA ACAAAGACGA GAACGAAAGC   
  
  
+ ACAATTCTAT GGAAATGGAA AATGAAGTCA CTTATGGAGA TTCAATAGTA AGCAAATCGA GAGCAGAAAA   
  
  
+ TCTAATCTCC TTTCGTCATT ATACAATCAA TATTGCCTAT TTGGTTTTAG TGATTTGTTT CAGCCGCAAA   
  
  
+ ACTTAATTTT CTTTGGTGCA TATGAAATCT TAAAAAGAAT TAAAATAAGA TATAATACGT TAACTCTTCA   
  
  
+ ACAACATGAT ATATTTAAGA TACCAATATT TGTATTCATC ATATAAAAAT TGTAGTGTTG CAAAATATTA   
  
  
+ AAATTATTTC ATAAATAAAC ATTATTATAA GAACTGACTC CGCGGATTAT CATATGGTAT AGATTACAGA   
  
  
+ GTGGGTGGGT TTAAATAATT TCCCCGACAC ATTATACTTT TAGAAGAGCT ATTAGCTCCA AATTATTTAA   
  
  
+ ACAATGTTCT AAAGAGCAAA AAAATCAATG TTTTGGATTT TGATCCGACC GAGAGGACTT GTCCGACCAT   
  
  
+ TCCATATTAA AATGTTCCTA GTTCTGATTA GCTAATACCA   

- TATTTCAACA GCACTAAAGA ACGGGAAATA AAAAATAAAT ATAATTGTAT ATAAAAATTT TTTTTTAATT   
  
  
- TCTGCAAGGA TTGGCTTTGA GTACATAAAA AACTAATACT TATACTTATA GGGGAGATAT AATTAGTACC   
  
  
- TCGTAATGTT GTACAAAAGC ATCGGTATAC AGTAGTGCTC TTACTAAAAA TCTTAACAAT CTTTTTATTT   
  
  
- AACTAAGTAT ATTTGTATAT GATACAAAAA ATAATTTGAT TGATAGTTTA ATTAATTATC ACATGTTTTT   
  
  
- TTATAAAAAA AGAAAGGAAT TTATTTTTGA TGCCTTAATG GATTATACCG ATTGTATATA TACTGTTAAT   
  
  
- TACTAATACT TATTATGTAT AAACTATTTT TTTAAAGATT GGAGAGAGAA AAAACAAATT AAAATATAAT   
  
  
- AATTTCCTTT AAATTGTTAG TGTAATTAGT ATATTATTTT TGTTAATCTA AAAAAGAATA TACAATATAA   
  
  
- AACTTAAAAA TTTTTGCTGA TATTTAATGA TTTTTACTAT TCTCAGGGTG TAATTTTTTA AACACTAGTT   
  
  
- GGCAAATTGA AAAAAAAATC AAGTTCGTTC TATGTTTACT AGTATATAGA CTATATCTGC ACCCGCAAGC   
  
  
- CTATGTGCAA GCCCAAACAT AGTCTATAAA GTCATATTTC CATATCTTGG GCAAGCCCAT AAAGATGTGA   
  
  
- GGCTCAGCCC AAGCCCAAGC CTATAAAACC TAGCCCAAGC TTATAAATTT AAAACTTCTT TTTTTCTTTA   
  
  
- ATAAGTGACA AATTCAAAAA ATATAAATTT ATATAGAATT GAATTGACTA AAAAAAATCA AAAATTTTCT   
  
  
- AATTTTATAA TTATACAAAC CTCTATTTTG AAATTTTTAT CTTTCTGTGA TTAAATCAAA AACAAAACTT   
  
  
- TTAAATCTAC GTTGAAAACA ATTACGTTCT TTGTTCTTGA ACTATACATA AAATTCACTC ATTGTTTACT   
  
  
- AAAACAGATA TCAATATACA TATAATAGAT TAAAACTCAT TATTCTTAGT AATTATATTT ATAAAACTTA   
  
  
- TTTTAATCTA TCTATTTGAT ATTTATATCT CAATTCATAT GAATACAAAC CAATAGAAGC TTATAATGGG   
  
  
- CAAGCCTATA TAATAGACTT GACCACTTTA TTCATTATAC AAAACAACAA AATTAATCTA TTAAAAATCT   
  
  
- GGCTCGAACA CTTATATATG ATCTGTTTGT AAATATAAAG CTCAGACGTG AATATAAGAT ATTCTCGAAC   
  
  
- TATATAATCT AAACTTGTGA TTGGACAATT ATATCAAACG GCCACTAAAA AAAAGTTTTA AAACTAAGAA   
  
  
- TCTATACATA TACCTCATTT TGATTAAAAA TGTCTACGGG TAAAAAAATT AACTGTGAAT ACATTAATTG   
  
  
- ACTTAAGTAT TTTGTTCCAA AAAATTTTTT TAAATTGAGT ATAGTTACTT TGTTTCTGCT CTTGCTTTCG   
  
  
- TGTTAAGATA CCTTTACCTT TTACTTCAGT GAATACCTCT AAGTTATCAT TCGTTTAGCT CTCGTCTTTT   
  
  
- AGATTAGAGG AAAGCAGTAA TATGTTAGTT ATAACGGATA AACCAAAATC ACTAAACAAA GTCGGCGTTT   
  
  
- TGAATTAAAA GAAACCACGT ATACTTTAGA ATTTTTCTTA ATTTTATTCT ATATTATGCA ATTGAGAAGT   
  
  
- TGTTGTACTA TATAAATTCT ATGGTTATAA ACATAAGTAG TATATTTTTA ACATCACAAC GTTTTATAAT   
  
  
- TTTAATAAAG TATTTATTTG TAATAATATT CTTGACTGAG GCGCCTAATA GTATACCATA TCTAATGTCT   
  
  
- CACCCACCCA AATTTATTAA AGGGGCTGTG TAATATGAAA ATCTTCTCGA TAATCGAGGT TTAATAAATT   
  
  
- TGTTACAAGA TTTCTCGTTT TTTTAGTTAC AAAACCTAAA ACTAGGCTGG CTCTCCTGAA CAGGCTGGTA   
  
  
- AGGTATAATT TTACAAGGAT CAAGACTAAT CGATTATGGT

  
  
Motifs Found  

+   

| Site Name | Organism | Position | Strand | Matrix score. | sequence | function |
| --- | --- | --- | --- | --- | --- | --- |
|  | organism | 860 | - | 4 | motif\_sequence | short\_function |
|  | organism | 1674 | + | 4 | motif\_sequence | short\_function |
|  | organism | 1506 | - | 4 | motif\_sequence | short\_function |
|  | organism | 1894 | + | 4 | motif\_sequence | short\_function |
|  | organism | 1104 | + | 4 | motif\_sequence | short\_function |
|  | organism | 1863 | - | 4 | motif\_sequence | short\_function |
|  | organism | 1545 | + | 4 | motif\_sequence | short\_function |
|  | organism | 1972 | + | 4 | motif\_sequence | short\_function |

>PlantCARE\_25378   
+ ATAAAGTTGT CGTGATTTCT TGCCCTTTAT TTTTTATTTA TATTAACATA TATTTTTAAA AAAAAATTAA   
  
  
+ AGACGTTCCT AACCGAAACT CATGTATTTT TTGATTATGA ATATGAATAT CCCCTCTATA TTAATCATGG   
  
  
+ AGCATTACAA CATGTTTTCG TAGCCATATG TCATCACGAG AATGATTTTT AGAATTGTTA GAAAAATAAA   
  
  
+ TTGATTCATA TAAACATATA CTATGTTTTT TATTAAACTA ACTATCAAAT TAATTAATAG TGTACAAAAA   
  
  
+ AATATTTTTT TCTTTCCTTA AATAAAAACT ACGGAATTAC CTAATATGGC TAACATATAT ATGACAATTA   
  
  
+ ATGATTATGA ATAATACATA TTTGATAAAA AAATTTCTAA CCTCTCTCTT TTTTGTTTAA TTTTATATTA   
  
  
+ TTAAAGGAAA TTTAACAATC ACATTAATCA TATAATAAAA ACAATTAGAT TTTTTCTTAT ATGTTATATT   
  
  
+ TTGAATTTTT AAAAACGACT ATAAATTACT AAAAATGATA AGAGTCCCAC ATTAAAAAAT TTGTGATCAA   
  
  
+ CCGTTTAACT TTTTTTTTAG TTCAAGCAAG ATACAAATGA TCATATATCT GATATAGACG TGGGCGTTCG   
  
  
+ GATACACGTT CGGGTTTGTA TCAGATATTT CAGTATAAAG GTATAGAACC CGTTCGGGTA TTTCTACACT   
  
  
+ CCGAGTCGGG TTCGGGTTCG GATATTTTGG ATCGGGTTCG AATATTTAAA TTTTGAAGAA AAAAAGAAAT   
  
  
+ TATTCACTGT TTAAGTTTTT TATATTTAAA TATATCTTAA CTTAACTGAT TTTTTTTAGT TTTTAAAAGA   
  
  
+ TTAAAATATT AATATGTTTG GAGATAAAAC TTTAAAAATA GAAAGACACT AATTTAGTTT TTGTTTTGAA   
  
  
+ AATTTAGATG CAACTTTTGT TAATGCAAGA AACAAGAACT TGATATGTAT TTTAAGTGAG TAACAAATGA   
  
  
+ TTTTGTCTAT AGTTATATGT ATATTATCTA ATTTTGAGTA ATAAGAATCA TTAATATAAA TATTTTGAAT   
  
  
+ AAAATTAGAT AGATAAACTA TAAATATAGA GTTAAGTATA CTTATGTTTG GTTATCTTCG AATATTACCC   
  
  
+ GTTCGGATAT ATTATCTGAA CTGGTGAAAT AAGTAATATG TTTTGTTGTT TTAATTAGAT AATTTTTAGA   
  
  
+ CCGAGCTTGT GAATATATAC TAGACAAACA TTTATATTTC GAGTCTGCAC TTATATTCTA TAAGAGCTTG   
  
  
+ ATATATTAGA TTTGAACACT AACCTGTTAA TATAGTTTGC CGGTGATTTT TTTTCAAAAT TTTGATTCTT   
  
  
+ AGATATGTAT ATGGAGTAAA ACTAATTTTT ACAGATGCCC ATTTTTTTAA TTGACACTTA TGTAATTAAC   
  
  
+ TGAATTCATA AAACAAGGTT TTTTAAAAAA ATTTAACTCA TATCAATGAA ACAAAGACGA GAACGAAAGC   
  
  
+ ACAATTCTAT GGAAATGGAA AATGAAGTCA CTTATGGAGA TTCAATAGTA AGCAAATCGA GAGCAGAAAA   
  
  
+ TCTAATCTCC TTTCGTCATT ATACAATCAA TATTGCCTAT TTGGTTTTAG TGATTTGTTT CAGCCGCAAA   
  
  
+ ACTTAATTTT CTTTGGTGCA TATGAAATCT TAAAAAGAAT TAAAATAAGA TATAATACGT TAACTCTTCA   
  
  
+ ACAACATGAT ATATTTAAGA TACCAATATT TGTATTCATC ATATAAAAAT TGTAGTGTTG CAAAATATTA   
  
  
+ AAATTATTTC ATAAATAAAC ATTATTATAA GAACTGACTC CGCGGATTAT CATATGGTAT AGATTACAGA   
  
  
+ GTGGGTGGGT TTAAATAATT TCCCCGACAC ATTATACTTT TAGAAGAGCT ATTAGCTCCA AATTATTTAA   
  
  
+ ACAATGTTCT AAAGAGCAAA AAAATCAATG TTTTGGATTT TGATCCGACC GAGAGGACTT GTCCGACCAT   
  
  
+ TCCATATTAA AATGTTCCTA GTTCTGATTA GCTAATACCA   

- TATTTCAACA GCACTAAAGA ACGGGAAATA AAAAATAAAT ATAATTGTAT ATAAAAATTT TTTTTTAATT   
  
  
- TCTGCAAGGA TTGGCTTTGA GTACATAAAA AACTAATACT TATACTTATA GGGGAGATAT AATTAGTACC   
  
  
- TCGTAATGTT GTACAAAAGC ATCGGTATAC AGTAGTGCTC TTACTAAAAA TCTTAACAAT CTTTTTATTT   
  
  
- AACTAAGTAT ATTTGTATAT GATACAAAAA ATAATTTGAT TGATAGTTTA ATTAATTATC ACATGTTTTT   
  
  
- TTATAAAAAA AGAAAGGAAT TTATTTTTGA TGCCTTAATG GATTATACCG ATTGTATATA TACTGTTAAT   
  
  
- TACTAATACT TATTATGTAT AAACTATTTT TTTAAAGATT GGAGAGAGAA AAAACAAATT AAAATATAAT   
  
  
- AATTTCCTTT AAATTGTTAG TGTAATTAGT ATATTATTTT TGTTAATCTA AAAAAGAATA TACAATATAA   
  
  
- AACTTAAAAA TTTTTGCTGA TATTTAATGA TTTTTACTAT TCTCAGGGTG TAATTTTTTA AACACTAGTT   
  
  
- GGCAAATTGA AAAAAAAATC AAGTTCGTTC TATGTTTACT AGTATATAGA CTATATCTGC ACCCGCAAGC   
  
  
- CTATGTGCAA GCCCAAACAT AGTCTATAAA GTCATATTTC CATATCTTGG GCAAGCCCAT AAAGATGTGA   
  
  
- GGCTCAGCCC AAGCCCAAGC CTATAAAACC TAGCCCAAGC TTATAAATTT AAAACTTCTT TTTTTCTTTA   
  
  
- ATAAGTGACA AATTCAAAAA ATATAAATTT ATATAGAATT GAATTGACTA AAAAAAATCA AAAATTTTCT   
  
  
- AATTTTATAA TTATACAAAC CTCTATTTTG AAATTTTTAT CTTTCTGTGA TTAAATCAAA AACAAAACTT   
  
  
- TTAAATCTAC GTTGAAAACA ATTACGTTCT TTGTTCTTGA ACTATACATA AAATTCACTC ATTGTTTACT   
  
  
- AAAACAGATA TCAATATACA TATAATAGAT TAAAACTCAT TATTCTTAGT AATTATATTT ATAAAACTTA   
  
  
- TTTTAATCTA TCTATTTGAT ATTTATATCT CAATTCATAT GAATACAAAC CAATAGAAGC TTATAATGGG   
  
  
- CAAGCCTATA TAATAGACTT GACCACTTTA TTCATTATAC AAAACAACAA AATTAATCTA TTAAAAATCT   
  
  
- GGCTCGAACA CTTATATATG ATCTGTTTGT AAATATAAAG CTCAGACGTG AATATAAGAT ATTCTCGAAC   
  
  
- TATATAATCT AAACTTGTGA TTGGACAATT ATATCAAACG GCCACTAAAA AAAAGTTTTA AAACTAAGAA   
  
  
- TCTATACATA TACCTCATTT TGATTAAAAA TGTCTACGGG TAAAAAAATT AACTGTGAAT ACATTAATTG   
  
  
- ACTTAAGTAT TTTGTTCCAA AAAATTTTTT TAAATTGAGT ATAGTTACTT TGTTTCTGCT CTTGCTTTCG   
  
  
- TGTTAAGATA CCTTTACCTT TTACTTCAGT GAATACCTCT AAGTTATCAT TCGTTTAGCT CTCGTCTTTT   
  
  
- AGATTAGAGG AAAGCAGTAA TATGTTAGTT ATAACGGATA AACCAAAATC ACTAAACAAA GTCGGCGTTT   
  
  
- TGAATTAAAA GAAACCACGT ATACTTTAGA ATTTTTCTTA ATTTTATTCT ATATTATGCA ATTGAGAAGT   
  
  
- TGTTGTACTA TATAAATTCT ATGGTTATAA ACATAAGTAG TATATTTTTA ACATCACAAC GTTTTATAAT   
  
  
- TTTAATAAAG TATTTATTTG TAATAATATT CTTGACTGAG GCGCCTAATA GTATACCATA TCTAATGTCT   
  
  
- CACCCACCCA AATTTATTAA AGGGGCTGTG TAATATGAAA ATCTTCTCGA TAATCGAGGT TTAATAAATT   
  
  
- TGTTACAAGA TTTCTCGTTT TTTTAGTTAC AAAACCTAAA ACTAGGCTGG CTCTCCTGAA CAGGCTGGTA   
  
  
- AGGTATAATT TTACAAGGAT CAAGACTAAT CGATTATGGT

+     AAGAA-motif

| Site Name | Organism | Position | Strand | Matrix score. | sequence | function |
| --- | --- | --- | --- | --- | --- | --- |
| AAGAA-motif | Avena sativa | 290 | - | 7 | GAAAGAA |  |

>PlantCARE\_25378   
+ ATAAAGTTGT CGTGATTTCT TGCCCTTTAT TTTTTATTTA TATTAACATA TATTTTTAAA AAAAAATTAA   
  
  
+ AGACGTTCCT AACCGAAACT CATGTATTTT TTGATTATGA ATATGAATAT CCCCTCTATA TTAATCATGG   
  
  
+ AGCATTACAA CATGTTTTCG TAGCCATATG TCATCACGAG AATGATTTTT AGAATTGTTA GAAAAATAAA   
  
  
+ TTGATTCATA TAAACATATA CTATGTTTTT TATTAAACTA ACTATCAAAT TAATTAATAG TGTACAAAAA   
  
  
+ AATATTTTTT TCTTTCCTTA AATAAAAACT ACGGAATTAC CTAATATGGC TAACATATAT ATGACAATTA   
  
  
+ ATGATTATGA ATAATACATA TTTGATAAAA AAATTTCTAA CCTCTCTCTT TTTTGTTTAA TTTTATATTA   
  
  
+ TTAAAGGAAA TTTAACAATC ACATTAATCA TATAATAAAA ACAATTAGAT TTTTTCTTAT ATGTTATATT   
  
  
+ TTGAATTTTT AAAAACGACT ATAAATTACT AAAAATGATA AGAGTCCCAC ATTAAAAAAT TTGTGATCAA   
  
  
+ CCGTTTAACT TTTTTTTTAG TTCAAGCAAG ATACAAATGA TCATATATCT GATATAGACG TGGGCGTTCG   
  
  
+ GATACACGTT CGGGTTTGTA TCAGATATTT CAGTATAAAG GTATAGAACC CGTTCGGGTA TTTCTACACT   
  
  
+ CCGAGTCGGG TTCGGGTTCG GATATTTTGG ATCGGGTTCG AATATTTAAA TTTTGAAGAA AAAAAGAAAT   
  
  
+ TATTCACTGT TTAAGTTTTT TATATTTAAA TATATCTTAA CTTAACTGAT TTTTTTTAGT TTTTAAAAGA   
  
  
+ TTAAAATATT AATATGTTTG GAGATAAAAC TTTAAAAATA GAAAGACACT AATTTAGTTT TTGTTTTGAA   
  
  
+ AATTTAGATG CAACTTTTGT TAATGCAAGA AACAAGAACT TGATATGTAT TTTAAGTGAG TAACAAATGA   
  
  
+ TTTTGTCTAT AGTTATATGT ATATTATCTA ATTTTGAGTA ATAAGAATCA TTAATATAAA TATTTTGAAT   
  
  
+ AAAATTAGAT AGATAAACTA TAAATATAGA GTTAAGTATA CTTATGTTTG GTTATCTTCG AATATTACCC   
  
  
+ GTTCGGATAT ATTATCTGAA CTGGTGAAAT AAGTAATATG TTTTGTTGTT TTAATTAGAT AATTTTTAGA   
  
  
+ CCGAGCTTGT GAATATATAC TAGACAAACA TTTATATTTC GAGTCTGCAC TTATATTCTA TAAGAGCTTG   
  
  
+ ATATATTAGA TTTGAACACT AACCTGTTAA TATAGTTTGC CGGTGATTTT TTTTCAAAAT TTTGATTCTT   
  
  
+ AGATATGTAT ATGGAGTAAA ACTAATTTTT ACAGATGCCC ATTTTTTTAA TTGACACTTA TGTAATTAAC   
  
  
+ TGAATTCATA AAACAAGGTT TTTTAAAAAA ATTTAACTCA TATCAATGAA ACAAAGACGA GAACGAAAGC   
  
  
+ ACAATTCTAT GGAAATGGAA AATGAAGTCA CTTATGGAGA TTCAATAGTA AGCAAATCGA GAGCAGAAAA   
  
  
+ TCTAATCTCC TTTCGTCATT ATACAATCAA TATTGCCTAT TTGGTTTTAG TGATTTGTTT CAGCCGCAAA   
  
  
+ ACTTAATTTT CTTTGGTGCA TATGAAATCT TAAAAAGAAT TAAAATAAGA TATAATACGT TAACTCTTCA   
  
  
+ ACAACATGAT ATATTTAAGA TACCAATATT TGTATTCATC ATATAAAAAT TGTAGTGTTG CAAAATATTA   
  
  
+ AAATTATTTC ATAAATAAAC ATTATTATAA GAACTGACTC CGCGGATTAT CATATGGTAT AGATTACAGA   
  
  
+ GTGGGTGGGT TTAAATAATT TCCCCGACAC ATTATACTTT TAGAAGAGCT ATTAGCTCCA AATTATTTAA   
  
  
+ ACAATGTTCT AAAGAGCAAA AAAATCAATG TTTTGGATTT TGATCCGACC GAGAGGACTT GTCCGACCAT   
  
  
+ TCCATATTAA AATGTTCCTA GTTCTGATTA GCTAATACCA   

- TATTTCAACA GCACTAAAGA ACGGGAAATA AAAAATAAAT ATAATTGTAT ATAAAAATTT TTTTTTAATT   
  
  
- TCTGCAAGGA TTGGCTTTGA GTACATAAAA AACTAATACT TATACTTATA GGGGAGATAT AATTAGTACC   
  
  
- TCGTAATGTT GTACAAAAGC ATCGGTATAC AGTAGTGCTC TTACTAAAAA TCTTAACAAT CTTTTTATTT   
  
  
- AACTAAGTAT ATTTGTATAT GATACAAAAA ATAATTTGAT TGATAGTTTA ATTAATTATC ACATGTTTTT   
  
  
- TTATAAAAAA AGAAAGGAAT TTATTTTTGA TGCCTTAATG GATTATACCG ATTGTATATA TACTGTTAAT   
  
  
- TACTAATACT TATTATGTAT AAACTATTTT TTTAAAGATT GGAGAGAGAA AAAACAAATT AAAATATAAT   
  
  
- AATTTCCTTT AAATTGTTAG TGTAATTAGT ATATTATTTT TGTTAATCTA AAAAAGAATA TACAATATAA   
  
  
- AACTTAAAAA TTTTTGCTGA TATTTAATGA TTTTTACTAT TCTCAGGGTG TAATTTTTTA AACACTAGTT   
  
  
- GGCAAATTGA AAAAAAAATC AAGTTCGTTC TATGTTTACT AGTATATAGA CTATATCTGC ACCCGCAAGC   
  
  
- CTATGTGCAA GCCCAAACAT AGTCTATAAA GTCATATTTC CATATCTTGG GCAAGCCCAT AAAGATGTGA   
  
  
- GGCTCAGCCC AAGCCCAAGC CTATAAAACC TAGCCCAAGC TTATAAATTT AAAACTTCTT TTTTTCTTTA   
  
  
- ATAAGTGACA AATTCAAAAA ATATAAATTT ATATAGAATT GAATTGACTA AAAAAAATCA AAAATTTTCT   
  
  
- AATTTTATAA TTATACAAAC CTCTATTTTG AAATTTTTAT CTTTCTGTGA TTAAATCAAA AACAAAACTT   
  
  
- TTAAATCTAC GTTGAAAACA ATTACGTTCT TTGTTCTTGA ACTATACATA AAATTCACTC ATTGTTTACT   
  
  
- AAAACAGATA TCAATATACA TATAATAGAT TAAAACTCAT TATTCTTAGT AATTATATTT ATAAAACTTA   
  
  
- TTTTAATCTA TCTATTTGAT ATTTATATCT CAATTCATAT GAATACAAAC CAATAGAAGC TTATAATGGG   
  
  
- CAAGCCTATA TAATAGACTT GACCACTTTA TTCATTATAC AAAACAACAA AATTAATCTA TTAAAAATCT   
  
  
- GGCTCGAACA CTTATATATG ATCTGTTTGT AAATATAAAG CTCAGACGTG AATATAAGAT ATTCTCGAAC   
  
  
- TATATAATCT AAACTTGTGA TTGGACAATT ATATCAAACG GCCACTAAAA AAAAGTTTTA AAACTAAGAA   
  
  
- TCTATACATA TACCTCATTT TGATTAAAAA TGTCTACGGG TAAAAAAATT AACTGTGAAT ACATTAATTG   
  
  
- ACTTAAGTAT TTTGTTCCAA AAAATTTTTT TAAATTGAGT ATAGTTACTT TGTTTCTGCT CTTGCTTTCG   
  
  
- TGTTAAGATA CCTTTACCTT TTACTTCAGT GAATACCTCT AAGTTATCAT TCGTTTAGCT CTCGTCTTTT   
  
  
- AGATTAGAGG AAAGCAGTAA TATGTTAGTT ATAACGGATA AACCAAAATC ACTAAACAAA GTCGGCGTTT   
  
  
- TGAATTAAAA GAAACCACGT ATACTTTAGA ATTTTTCTTA ATTTTATTCT ATATTATGCA ATTGAGAAGT   
  
  
- TGTTGTACTA TATAAATTCT ATGGTTATAA ACATAAGTAG TATATTTTTA ACATCACAAC GTTTTATAAT   
  
  
- TTTAATAAAG TATTTATTTG TAATAATATT CTTGACTGAG GCGCCTAATA GTATACCATA TCTAATGTCT   
  
  
- CACCCACCCA AATTTATTAA AGGGGCTGTG TAATATGAAA ATCTTCTCGA TAATCGAGGT TTAATAAATT   
  
  
- TGTTACAAGA TTTCTCGTTT TTTTAGTTAC AAAACCTAAA ACTAGGCTGG CTCTCCTGAA CAGGCTGGTA   
  
  
- AGGTATAATT TTACAAGGAT CAAGACTAAT CGATTATGGT

+     ABRE

| Site Name | Organism | Position | Strand | Matrix score. | sequence | function |
| --- | --- | --- | --- | --- | --- | --- |
| ABRE | Arabidopsis thaliana | 618 | + | 5 | ACGTG | cis-acting element involved in the abscisic acid responsiveness |
| ABRE | Arabidopsis thaliana | 635 | - | 5 | ACGTG | cis-acting element involved in the abscisic acid responsiveness |

>PlantCARE\_25378   
+ ATAAAGTTGT CGTGATTTCT TGCCCTTTAT TTTTTATTTA TATTAACATA TATTTTTAAA AAAAAATTAA   
  
  
+ AGACGTTCCT AACCGAAACT CATGTATTTT TTGATTATGA ATATGAATAT CCCCTCTATA TTAATCATGG   
  
  
+ AGCATTACAA CATGTTTTCG TAGCCATATG TCATCACGAG AATGATTTTT AGAATTGTTA GAAAAATAAA   
  
  
+ TTGATTCATA TAAACATATA CTATGTTTTT TATTAAACTA ACTATCAAAT TAATTAATAG TGTACAAAAA   
  
  
+ AATATTTTTT TCTTTCCTTA AATAAAAACT ACGGAATTAC CTAATATGGC TAACATATAT ATGACAATTA   
  
  
+ ATGATTATGA ATAATACATA TTTGATAAAA AAATTTCTAA CCTCTCTCTT TTTTGTTTAA TTTTATATTA   
  
  
+ TTAAAGGAAA TTTAACAATC ACATTAATCA TATAATAAAA ACAATTAGAT TTTTTCTTAT ATGTTATATT   
  
  
+ TTGAATTTTT AAAAACGACT ATAAATTACT AAAAATGATA AGAGTCCCAC ATTAAAAAAT TTGTGATCAA   
  
  
+ CCGTTTAACT TTTTTTTTAG TTCAAGCAAG ATACAAATGA TCATATATCT GATATAGACG TGGGCGTTCG   
  
  
+ GATACACGTT CGGGTTTGTA TCAGATATTT CAGTATAAAG GTATAGAACC CGTTCGGGTA TTTCTACACT   
  
  
+ CCGAGTCGGG TTCGGGTTCG GATATTTTGG ATCGGGTTCG AATATTTAAA TTTTGAAGAA AAAAAGAAAT   
  
  
+ TATTCACTGT TTAAGTTTTT TATATTTAAA TATATCTTAA CTTAACTGAT TTTTTTTAGT TTTTAAAAGA   
  
  
+ TTAAAATATT AATATGTTTG GAGATAAAAC TTTAAAAATA GAAAGACACT AATTTAGTTT TTGTTTTGAA   
  
  
+ AATTTAGATG CAACTTTTGT TAATGCAAGA AACAAGAACT TGATATGTAT TTTAAGTGAG TAACAAATGA   
  
  
+ TTTTGTCTAT AGTTATATGT ATATTATCTA ATTTTGAGTA ATAAGAATCA TTAATATAAA TATTTTGAAT   
  
  
+ AAAATTAGAT AGATAAACTA TAAATATAGA GTTAAGTATA CTTATGTTTG GTTATCTTCG AATATTACCC   
  
  
+ GTTCGGATAT ATTATCTGAA CTGGTGAAAT AAGTAATATG TTTTGTTGTT TTAATTAGAT AATTTTTAGA   
  
  
+ CCGAGCTTGT GAATATATAC TAGACAAACA TTTATATTTC GAGTCTGCAC TTATATTCTA TAAGAGCTTG   
  
  
+ ATATATTAGA TTTGAACACT AACCTGTTAA TATAGTTTGC CGGTGATTTT TTTTCAAAAT TTTGATTCTT   
  
  
+ AGATATGTAT ATGGAGTAAA ACTAATTTTT ACAGATGCCC ATTTTTTTAA TTGACACTTA TGTAATTAAC   
  
  
+ TGAATTCATA AAACAAGGTT TTTTAAAAAA ATTTAACTCA TATCAATGAA ACAAAGACGA GAACGAAAGC   
  
  
+ ACAATTCTAT GGAAATGGAA AATGAAGTCA CTTATGGAGA TTCAATAGTA AGCAAATCGA GAGCAGAAAA   
  
  
+ TCTAATCTCC TTTCGTCATT ATACAATCAA TATTGCCTAT TTGGTTTTAG TGATTTGTTT CAGCCGCAAA   
  
  
+ ACTTAATTTT CTTTGGTGCA TATGAAATCT TAAAAAGAAT TAAAATAAGA TATAATACGT TAACTCTTCA   
  
  
+ ACAACATGAT ATATTTAAGA TACCAATATT TGTATTCATC ATATAAAAAT TGTAGTGTTG CAAAATATTA   
  
  
+ AAATTATTTC ATAAATAAAC ATTATTATAA GAACTGACTC CGCGGATTAT CATATGGTAT AGATTACAGA   
  
  
+ GTGGGTGGGT TTAAATAATT TCCCCGACAC ATTATACTTT TAGAAGAGCT ATTAGCTCCA AATTATTTAA   
  
  
+ ACAATGTTCT AAAGAGCAAA AAAATCAATG TTTTGGATTT TGATCCGACC GAGAGGACTT GTCCGACCAT   
  
  
+ TCCATATTAA AATGTTCCTA GTTCTGATTA GCTAATACCA   

- TATTTCAACA GCACTAAAGA ACGGGAAATA AAAAATAAAT ATAATTGTAT ATAAAAATTT TTTTTTAATT   
  
  
- TCTGCAAGGA TTGGCTTTGA GTACATAAAA AACTAATACT TATACTTATA GGGGAGATAT AATTAGTACC   
  
  
- TCGTAATGTT GTACAAAAGC ATCGGTATAC AGTAGTGCTC TTACTAAAAA TCTTAACAAT CTTTTTATTT   
  
  
- AACTAAGTAT ATTTGTATAT GATACAAAAA ATAATTTGAT TGATAGTTTA ATTAATTATC ACATGTTTTT   
  
  
- TTATAAAAAA AGAAAGGAAT TTATTTTTGA TGCCTTAATG GATTATACCG ATTGTATATA TACTGTTAAT   
  
  
- TACTAATACT TATTATGTAT AAACTATTTT TTTAAAGATT GGAGAGAGAA AAAACAAATT AAAATATAAT   
  
  
- AATTTCCTTT AAATTGTTAG TGTAATTAGT ATATTATTTT TGTTAATCTA AAAAAGAATA TACAATATAA   
  
  
- AACTTAAAAA TTTTTGCTGA TATTTAATGA TTTTTACTAT TCTCAGGGTG TAATTTTTTA AACACTAGTT   
  
  
- GGCAAATTGA AAAAAAAATC AAGTTCGTTC TATGTTTACT AGTATATAGA CTATATCTGC ACCCGCAAGC   
  
  
- CTATGTGCAA GCCCAAACAT AGTCTATAAA GTCATATTTC CATATCTTGG GCAAGCCCAT AAAGATGTGA   
  
  
- GGCTCAGCCC AAGCCCAAGC CTATAAAACC TAGCCCAAGC TTATAAATTT AAAACTTCTT TTTTTCTTTA   
  
  
- ATAAGTGACA AATTCAAAAA ATATAAATTT ATATAGAATT GAATTGACTA AAAAAAATCA AAAATTTTCT   
  
  
- AATTTTATAA TTATACAAAC CTCTATTTTG AAATTTTTAT CTTTCTGTGA TTAAATCAAA AACAAAACTT   
  
  
- TTAAATCTAC GTTGAAAACA ATTACGTTCT TTGTTCTTGA ACTATACATA AAATTCACTC ATTGTTTACT   
  
  
- AAAACAGATA TCAATATACA TATAATAGAT TAAAACTCAT TATTCTTAGT AATTATATTT ATAAAACTTA   
  
  
- TTTTAATCTA TCTATTTGAT ATTTATATCT CAATTCATAT GAATACAAAC CAATAGAAGC TTATAATGGG   
  
  
- CAAGCCTATA TAATAGACTT GACCACTTTA TTCATTATAC AAAACAACAA AATTAATCTA TTAAAAATCT   
  
  
- GGCTCGAACA CTTATATATG ATCTGTTTGT AAATATAAAG CTCAGACGTG AATATAAGAT ATTCTCGAAC   
  
  
- TATATAATCT AAACTTGTGA TTGGACAATT ATATCAAACG GCCACTAAAA AAAAGTTTTA AAACTAAGAA   
  
  
- TCTATACATA TACCTCATTT TGATTAAAAA TGTCTACGGG TAAAAAAATT AACTGTGAAT ACATTAATTG   
  
  
- ACTTAAGTAT TTTGTTCCAA AAAATTTTTT TAAATTGAGT ATAGTTACTT TGTTTCTGCT CTTGCTTTCG   
  
  
- TGTTAAGATA CCTTTACCTT TTACTTCAGT GAATACCTCT AAGTTATCAT TCGTTTAGCT CTCGTCTTTT   
  
  
- AGATTAGAGG AAAGCAGTAA TATGTTAGTT ATAACGGATA AACCAAAATC ACTAAACAAA GTCGGCGTTT   
  
  
- TGAATTAAAA GAAACCACGT ATACTTTAGA ATTTTTCTTA ATTTTATTCT ATATTATGCA ATTGAGAAGT   
  
  
- TGTTGTACTA TATAAATTCT ATGGTTATAA ACATAAGTAG TATATTTTTA ACATCACAAC GTTTTATAAT   
  
  
- TTTAATAAAG TATTTATTTG TAATAATATT CTTGACTGAG GCGCCTAATA GTATACCATA TCTAATGTCT   
  
  
- CACCCACCCA AATTTATTAA AGGGGCTGTG TAATATGAAA ATCTTCTCGA TAATCGAGGT TTAATAAATT   
  
  
- TGTTACAAGA TTTCTCGTTT TTTTAGTTAC AAAACCTAAA ACTAGGCTGG CTCTCCTGAA CAGGCTGGTA   
  
  
- AGGTATAATT TTACAAGGAT CAAGACTAAT CGATTATGGT

+     ACE

| Site Name | Organism | Position | Strand | Matrix score. | sequence | function |
| --- | --- | --- | --- | --- | --- | --- |
| ACE | Petroselinum crispum | 1383 | + | 9 | GACACGTATG | cis-acting element involved in light responsiveness |
| ACE | Petroselinum crispum | 1664 | - | 9 | CTAACGTATT | cis-acting element involved in light responsiveness |

>PlantCARE\_25378   
+ ATAAAGTTGT CGTGATTTCT TGCCCTTTAT TTTTTATTTA TATTAACATA TATTTTTAAA AAAAAATTAA   
  
  
+ AGACGTTCCT AACCGAAACT CATGTATTTT TTGATTATGA ATATGAATAT CCCCTCTATA TTAATCATGG   
  
  
+ AGCATTACAA CATGTTTTCG TAGCCATATG TCATCACGAG AATGATTTTT AGAATTGTTA GAAAAATAAA   
  
  
+ TTGATTCATA TAAACATATA CTATGTTTTT TATTAAACTA ACTATCAAAT TAATTAATAG TGTACAAAAA   
  
  
+ AATATTTTTT TCTTTCCTTA AATAAAAACT ACGGAATTAC CTAATATGGC TAACATATAT ATGACAATTA   
  
  
+ ATGATTATGA ATAATACATA TTTGATAAAA AAATTTCTAA CCTCTCTCTT TTTTGTTTAA TTTTATATTA   
  
  
+ TTAAAGGAAA TTTAACAATC ACATTAATCA TATAATAAAA ACAATTAGAT TTTTTCTTAT ATGTTATATT   
  
  
+ TTGAATTTTT AAAAACGACT ATAAATTACT AAAAATGATA AGAGTCCCAC ATTAAAAAAT TTGTGATCAA   
  
  
+ CCGTTTAACT TTTTTTTTAG TTCAAGCAAG ATACAAATGA TCATATATCT GATATAGACG TGGGCGTTCG   
  
  
+ GATACACGTT CGGGTTTGTA TCAGATATTT CAGTATAAAG GTATAGAACC CGTTCGGGTA TTTCTACACT   
  
  
+ CCGAGTCGGG TTCGGGTTCG GATATTTTGG ATCGGGTTCG AATATTTAAA TTTTGAAGAA AAAAAGAAAT   
  
  
+ TATTCACTGT TTAAGTTTTT TATATTTAAA TATATCTTAA CTTAACTGAT TTTTTTTAGT TTTTAAAAGA   
  
  
+ TTAAAATATT AATATGTTTG GAGATAAAAC TTTAAAAATA GAAAGACACT AATTTAGTTT TTGTTTTGAA   
  
  
+ AATTTAGATG CAACTTTTGT TAATGCAAGA AACAAGAACT TGATATGTAT TTTAAGTGAG TAACAAATGA   
  
  
+ TTTTGTCTAT AGTTATATGT ATATTATCTA ATTTTGAGTA ATAAGAATCA TTAATATAAA TATTTTGAAT   
  
  
+ AAAATTAGAT AGATAAACTA TAAATATAGA GTTAAGTATA CTTATGTTTG GTTATCTTCG AATATTACCC   
  
  
+ GTTCGGATAT ATTATCTGAA CTGGTGAAAT AAGTAATATG TTTTGTTGTT TTAATTAGAT AATTTTTAGA   
  
  
+ CCGAGCTTGT GAATATATAC TAGACAAACA TTTATATTTC GAGTCTGCAC TTATATTCTA TAAGAGCTTG   
  
  
+ ATATATTAGA TTTGAACACT AACCTGTTAA TATAGTTTGC CGGTGATTTT TTTTCAAAAT TTTGATTCTT   
  
  
+ AGATATGTAT ATGGAGTAAA ACTAATTTTT ACAGATGCCC ATTTTTTTAA TTGACACTTA TGTAATTAAC   
  
  
+ TGAATTCATA AAACAAGGTT TTTTAAAAAA ATTTAACTCA TATCAATGAA ACAAAGACGA GAACGAAAGC   
  
  
+ ACAATTCTAT GGAAATGGAA AATGAAGTCA CTTATGGAGA TTCAATAGTA AGCAAATCGA GAGCAGAAAA   
  
  
+ TCTAATCTCC TTTCGTCATT ATACAATCAA TATTGCCTAT TTGGTTTTAG TGATTTGTTT CAGCCGCAAA   
  
  
+ ACTTAATTTT CTTTGGTGCA TATGAAATCT TAAAAAGAAT TAAAATAAGA TATAATACGT TAACTCTTCA   
  
  
+ ACAACATGAT ATATTTAAGA TACCAATATT TGTATTCATC ATATAAAAAT TGTAGTGTTG CAAAATATTA   
  
  
+ AAATTATTTC ATAAATAAAC ATTATTATAA GAACTGACTC CGCGGATTAT CATATGGTAT AGATTACAGA   
  
  
+ GTGGGTGGGT TTAAATAATT TCCCCGACAC ATTATACTTT TAGAAGAGCT ATTAGCTCCA AATTATTTAA   
  
  
+ ACAATGTTCT AAAGAGCAAA AAAATCAATG TTTTGGATTT TGATCCGACC GAGAGGACTT GTCCGACCAT   
  
  
+ TCCATATTAA AATGTTCCTA GTTCTGATTA GCTAATACCA   

- TATTTCAACA GCACTAAAGA ACGGGAAATA AAAAATAAAT ATAATTGTAT ATAAAAATTT TTTTTTAATT   
  
  
- TCTGCAAGGA TTGGCTTTGA GTACATAAAA AACTAATACT TATACTTATA GGGGAGATAT AATTAGTACC   
  
  
- TCGTAATGTT GTACAAAAGC ATCGGTATAC AGTAGTGCTC TTACTAAAAA TCTTAACAAT CTTTTTATTT   
  
  
- AACTAAGTAT ATTTGTATAT GATACAAAAA ATAATTTGAT TGATAGTTTA ATTAATTATC ACATGTTTTT   
  
  
- TTATAAAAAA AGAAAGGAAT TTATTTTTGA TGCCTTAATG GATTATACCG ATTGTATATA TACTGTTAAT   
  
  
- TACTAATACT TATTATGTAT AAACTATTTT TTTAAAGATT GGAGAGAGAA AAAACAAATT AAAATATAAT   
  
  
- AATTTCCTTT AAATTGTTAG TGTAATTAGT ATATTATTTT TGTTAATCTA AAAAAGAATA TACAATATAA   
  
  
- AACTTAAAAA TTTTTGCTGA TATTTAATGA TTTTTACTAT TCTCAGGGTG TAATTTTTTA AACACTAGTT   
  
  
- GGCAAATTGA AAAAAAAATC AAGTTCGTTC TATGTTTACT AGTATATAGA CTATATCTGC ACCCGCAAGC   
  
  
- CTATGTGCAA GCCCAAACAT AGTCTATAAA GTCATATTTC CATATCTTGG GCAAGCCCAT AAAGATGTGA   
  
  
- GGCTCAGCCC AAGCCCAAGC CTATAAAACC TAGCCCAAGC TTATAAATTT AAAACTTCTT TTTTTCTTTA   
  
  
- ATAAGTGACA AATTCAAAAA ATATAAATTT ATATAGAATT GAATTGACTA AAAAAAATCA AAAATTTTCT   
  
  
- AATTTTATAA TTATACAAAC CTCTATTTTG AAATTTTTAT CTTTCTGTGA TTAAATCAAA AACAAAACTT   
  
  
- TTAAATCTAC GTTGAAAACA ATTACGTTCT TTGTTCTTGA ACTATACATA AAATTCACTC ATTGTTTACT   
  
  
- AAAACAGATA TCAATATACA TATAATAGAT TAAAACTCAT TATTCTTAGT AATTATATTT ATAAAACTTA   
  
  
- TTTTAATCTA TCTATTTGAT ATTTATATCT CAATTCATAT GAATACAAAC CAATAGAAGC TTATAATGGG   
  
  
- CAAGCCTATA TAATAGACTT GACCACTTTA TTCATTATAC AAAACAACAA AATTAATCTA TTAAAAATCT   
  
  
- GGCTCGAACA CTTATATATG ATCTGTTTGT AAATATAAAG CTCAGACGTG AATATAAGAT ATTCTCGAAC   
  
  
- TATATAATCT AAACTTGTGA TTGGACAATT ATATCAAACG GCCACTAAAA AAAAGTTTTA AAACTAAGAA   
  
  
- TCTATACATA TACCTCATTT TGATTAAAAA TGTCTACGGG TAAAAAAATT AACTGTGAAT ACATTAATTG   
  
  
- ACTTAAGTAT TTTGTTCCAA AAAATTTTTT TAAATTGAGT ATAGTTACTT TGTTTCTGCT CTTGCTTTCG   
  
  
- TGTTAAGATA CCTTTACCTT TTACTTCAGT GAATACCTCT AAGTTATCAT TCGTTTAGCT CTCGTCTTTT   
  
  
- AGATTAGAGG AAAGCAGTAA TATGTTAGTT ATAACGGATA AACCAAAATC ACTAAACAAA GTCGGCGTTT   
  
  
- TGAATTAAAA GAAACCACGT ATACTTTAGA ATTTTTCTTA ATTTTATTCT ATATTATGCA ATTGAGAAGT   
  
  
- TGTTGTACTA TATAAATTCT ATGGTTATAA ACATAAGTAG TATATTTTTA ACATCACAAC GTTTTATAAT   
  
  
- TTTAATAAAG TATTTATTTG TAATAATATT CTTGACTGAG GCGCCTAATA GTATACCATA TCTAATGTCT   
  
  
- CACCCACCCA AATTTATTAA AGGGGCTGTG TAATATGAAA ATCTTCTCGA TAATCGAGGT TTAATAAATT   
  
  
- TGTTACAAGA TTTCTCGTTT TTTTAGTTAC AAAACCTAAA ACTAGGCTGG CTCTCCTGAA CAGGCTGGTA   
  
  
- AGGTATAATT TTACAAGGAT CAAGACTAAT CGATTATGGT

+     AE-box

| Site Name | Organism | Position | Strand | Matrix score. | sequence | function |
| --- | --- | --- | --- | --- | --- | --- |
| AE-box | Arabidopsis thaliana | 938 | + | 8 | AGAAACAA | part of a module for light response |

>PlantCARE\_25378   
+ ATAAAGTTGT CGTGATTTCT TGCCCTTTAT TTTTTATTTA TATTAACATA TATTTTTAAA AAAAAATTAA   
  
  
+ AGACGTTCCT AACCGAAACT CATGTATTTT TTGATTATGA ATATGAATAT CCCCTCTATA TTAATCATGG   
  
  
+ AGCATTACAA CATGTTTTCG TAGCCATATG TCATCACGAG AATGATTTTT AGAATTGTTA GAAAAATAAA   
  
  
+ TTGATTCATA TAAACATATA CTATGTTTTT TATTAAACTA ACTATCAAAT TAATTAATAG TGTACAAAAA   
  
  
+ AATATTTTTT TCTTTCCTTA AATAAAAACT ACGGAATTAC CTAATATGGC TAACATATAT ATGACAATTA   
  
  
+ ATGATTATGA ATAATACATA TTTGATAAAA AAATTTCTAA CCTCTCTCTT TTTTGTTTAA TTTTATATTA   
  
  
+ TTAAAGGAAA TTTAACAATC ACATTAATCA TATAATAAAA ACAATTAGAT TTTTTCTTAT ATGTTATATT   
  
  
+ TTGAATTTTT AAAAACGACT ATAAATTACT AAAAATGATA AGAGTCCCAC ATTAAAAAAT TTGTGATCAA   
  
  
+ CCGTTTAACT TTTTTTTTAG TTCAAGCAAG ATACAAATGA TCATATATCT GATATAGACG TGGGCGTTCG   
  
  
+ GATACACGTT CGGGTTTGTA TCAGATATTT CAGTATAAAG GTATAGAACC CGTTCGGGTA TTTCTACACT   
  
  
+ CCGAGTCGGG TTCGGGTTCG GATATTTTGG ATCGGGTTCG AATATTTAAA TTTTGAAGAA AAAAAGAAAT   
  
  
+ TATTCACTGT TTAAGTTTTT TATATTTAAA TATATCTTAA CTTAACTGAT TTTTTTTAGT TTTTAAAAGA   
  
  
+ TTAAAATATT AATATGTTTG GAGATAAAAC TTTAAAAATA GAAAGACACT AATTTAGTTT TTGTTTTGAA   
  
  
+ AATTTAGATG CAACTTTTGT TAATGCAAGA AACAAGAACT TGATATGTAT TTTAAGTGAG TAACAAATGA   
  
  
+ TTTTGTCTAT AGTTATATGT ATATTATCTA ATTTTGAGTA ATAAGAATCA TTAATATAAA TATTTTGAAT   
  
  
+ AAAATTAGAT AGATAAACTA TAAATATAGA GTTAAGTATA CTTATGTTTG GTTATCTTCG AATATTACCC   
  
  
+ GTTCGGATAT ATTATCTGAA CTGGTGAAAT AAGTAATATG TTTTGTTGTT TTAATTAGAT AATTTTTAGA   
  
  
+ CCGAGCTTGT GAATATATAC TAGACAAACA TTTATATTTC GAGTCTGCAC TTATATTCTA TAAGAGCTTG   
  
  
+ ATATATTAGA TTTGAACACT AACCTGTTAA TATAGTTTGC CGGTGATTTT TTTTCAAAAT TTTGATTCTT   
  
  
+ AGATATGTAT ATGGAGTAAA ACTAATTTTT ACAGATGCCC ATTTTTTTAA TTGACACTTA TGTAATTAAC   
  
  
+ TGAATTCATA AAACAAGGTT TTTTAAAAAA ATTTAACTCA TATCAATGAA ACAAAGACGA GAACGAAAGC   
  
  
+ ACAATTCTAT GGAAATGGAA AATGAAGTCA CTTATGGAGA TTCAATAGTA AGCAAATCGA GAGCAGAAAA   
  
  
+ TCTAATCTCC TTTCGTCATT ATACAATCAA TATTGCCTAT TTGGTTTTAG TGATTTGTTT CAGCCGCAAA   
  
  
+ ACTTAATTTT CTTTGGTGCA TATGAAATCT TAAAAAGAAT TAAAATAAGA TATAATACGT TAACTCTTCA   
  
  
+ ACAACATGAT ATATTTAAGA TACCAATATT TGTATTCATC ATATAAAAAT TGTAGTGTTG CAAAATATTA   
  
  
+ AAATTATTTC ATAAATAAAC ATTATTATAA GAACTGACTC CGCGGATTAT CATATGGTAT AGATTACAGA   
  
  
+ GTGGGTGGGT TTAAATAATT TCCCCGACAC ATTATACTTT TAGAAGAGCT ATTAGCTCCA AATTATTTAA   
  
  
+ ACAATGTTCT AAAGAGCAAA AAAATCAATG TTTTGGATTT TGATCCGACC GAGAGGACTT GTCCGACCAT   
  
  
+ TCCATATTAA AATGTTCCTA GTTCTGATTA GCTAATACCA   

- TATTTCAACA GCACTAAAGA ACGGGAAATA AAAAATAAAT ATAATTGTAT ATAAAAATTT TTTTTTAATT   
  
  
- TCTGCAAGGA TTGGCTTTGA GTACATAAAA AACTAATACT TATACTTATA GGGGAGATAT AATTAGTACC   
  
  
- TCGTAATGTT GTACAAAAGC ATCGGTATAC AGTAGTGCTC TTACTAAAAA TCTTAACAAT CTTTTTATTT   
  
  
- AACTAAGTAT ATTTGTATAT GATACAAAAA ATAATTTGAT TGATAGTTTA ATTAATTATC ACATGTTTTT   
  
  
- TTATAAAAAA AGAAAGGAAT TTATTTTTGA TGCCTTAATG GATTATACCG ATTGTATATA TACTGTTAAT   
  
  
- TACTAATACT TATTATGTAT AAACTATTTT TTTAAAGATT GGAGAGAGAA AAAACAAATT AAAATATAAT   
  
  
- AATTTCCTTT AAATTGTTAG TGTAATTAGT ATATTATTTT TGTTAATCTA AAAAAGAATA TACAATATAA   
  
  
- AACTTAAAAA TTTTTGCTGA TATTTAATGA TTTTTACTAT TCTCAGGGTG TAATTTTTTA AACACTAGTT   
  
  
- GGCAAATTGA AAAAAAAATC AAGTTCGTTC TATGTTTACT AGTATATAGA CTATATCTGC ACCCGCAAGC   
  
  
- CTATGTGCAA GCCCAAACAT AGTCTATAAA GTCATATTTC CATATCTTGG GCAAGCCCAT AAAGATGTGA   
  
  
- GGCTCAGCCC AAGCCCAAGC CTATAAAACC TAGCCCAAGC TTATAAATTT AAAACTTCTT TTTTTCTTTA   
  
  
- ATAAGTGACA AATTCAAAAA ATATAAATTT ATATAGAATT GAATTGACTA AAAAAAATCA AAAATTTTCT   
  
  
- AATTTTATAA TTATACAAAC CTCTATTTTG AAATTTTTAT CTTTCTGTGA TTAAATCAAA AACAAAACTT   
  
  
- TTAAATCTAC GTTGAAAACA ATTACGTTCT TTGTTCTTGA ACTATACATA AAATTCACTC ATTGTTTACT   
  
  
- AAAACAGATA TCAATATACA TATAATAGAT TAAAACTCAT TATTCTTAGT AATTATATTT ATAAAACTTA   
  
  
- TTTTAATCTA TCTATTTGAT ATTTATATCT CAATTCATAT GAATACAAAC CAATAGAAGC TTATAATGGG   
  
  
- CAAGCCTATA TAATAGACTT GACCACTTTA TTCATTATAC AAAACAACAA AATTAATCTA TTAAAAATCT   
  
  
- GGCTCGAACA CTTATATATG ATCTGTTTGT AAATATAAAG CTCAGACGTG AATATAAGAT ATTCTCGAAC   
  
  
- TATATAATCT AAACTTGTGA TTGGACAATT ATATCAAACG GCCACTAAAA AAAAGTTTTA AAACTAAGAA   
  
  
- TCTATACATA TACCTCATTT TGATTAAAAA TGTCTACGGG TAAAAAAATT AACTGTGAAT ACATTAATTG   
  
  
- ACTTAAGTAT TTTGTTCCAA AAAATTTTTT TAAATTGAGT ATAGTTACTT TGTTTCTGCT CTTGCTTTCG   
  
  
- TGTTAAGATA CCTTTACCTT TTACTTCAGT GAATACCTCT AAGTTATCAT TCGTTTAGCT CTCGTCTTTT   
  
  
- AGATTAGAGG AAAGCAGTAA TATGTTAGTT ATAACGGATA AACCAAAATC ACTAAACAAA GTCGGCGTTT   
  
  
- TGAATTAAAA GAAACCACGT ATACTTTAGA ATTTTTCTTA ATTTTATTCT ATATTATGCA ATTGAGAAGT   
  
  
- TGTTGTACTA TATAAATTCT ATGGTTATAA ACATAAGTAG TATATTTTTA ACATCACAAC GTTTTATAAT   
  
  
- TTTAATAAAG TATTTATTTG TAATAATATT CTTGACTGAG GCGCCTAATA GTATACCATA TCTAATGTCT   
  
  
- CACCCACCCA AATTTATTAA AGGGGCTGTG TAATATGAAA ATCTTCTCGA TAATCGAGGT TTAATAAATT   
  
  
- TGTTACAAGA TTTCTCGTTT TTTTAGTTAC AAAACCTAAA ACTAGGCTGG CTCTCCTGAA CAGGCTGGTA   
  
  
- AGGTATAATT TTACAAGGAT CAAGACTAAT CGATTATGGT

+     ARE

| Site Name | Organism | Position | Strand | Matrix score. | sequence | function |
| --- | --- | --- | --- | --- | --- | --- |
| ARE | Zea mays | 1582 | - | 6 | AAACCA | cis-acting regulatory element essential for the anaerobic induction |

>PlantCARE\_25378   
+ ATAAAGTTGT CGTGATTTCT TGCCCTTTAT TTTTTATTTA TATTAACATA TATTTTTAAA AAAAAATTAA   
  
  
+ AGACGTTCCT AACCGAAACT CATGTATTTT TTGATTATGA ATATGAATAT CCCCTCTATA TTAATCATGG   
  
  
+ AGCATTACAA CATGTTTTCG TAGCCATATG TCATCACGAG AATGATTTTT AGAATTGTTA GAAAAATAAA   
  
  
+ TTGATTCATA TAAACATATA CTATGTTTTT TATTAAACTA ACTATCAAAT TAATTAATAG TGTACAAAAA   
  
  
+ AATATTTTTT TCTTTCCTTA AATAAAAACT ACGGAATTAC CTAATATGGC TAACATATAT ATGACAATTA   
  
  
+ ATGATTATGA ATAATACATA TTTGATAAAA AAATTTCTAA CCTCTCTCTT TTTTGTTTAA TTTTATATTA   
  
  
+ TTAAAGGAAA TTTAACAATC ACATTAATCA TATAATAAAA ACAATTAGAT TTTTTCTTAT ATGTTATATT   
  
  
+ TTGAATTTTT AAAAACGACT ATAAATTACT AAAAATGATA AGAGTCCCAC ATTAAAAAAT TTGTGATCAA   
  
  
+ CCGTTTAACT TTTTTTTTAG TTCAAGCAAG ATACAAATGA TCATATATCT GATATAGACG TGGGCGTTCG   
  
  
+ GATACACGTT CGGGTTTGTA TCAGATATTT CAGTATAAAG GTATAGAACC CGTTCGGGTA TTTCTACACT   
  
  
+ CCGAGTCGGG TTCGGGTTCG GATATTTTGG ATCGGGTTCG AATATTTAAA TTTTGAAGAA AAAAAGAAAT   
  
  
+ TATTCACTGT TTAAGTTTTT TATATTTAAA TATATCTTAA CTTAACTGAT TTTTTTTAGT TTTTAAAAGA   
  
  
+ TTAAAATATT AATATGTTTG GAGATAAAAC TTTAAAAATA GAAAGACACT AATTTAGTTT TTGTTTTGAA   
  
  
+ AATTTAGATG CAACTTTTGT TAATGCAAGA AACAAGAACT TGATATGTAT TTTAAGTGAG TAACAAATGA   
  
  
+ TTTTGTCTAT AGTTATATGT ATATTATCTA ATTTTGAGTA ATAAGAATCA TTAATATAAA TATTTTGAAT   
  
  
+ AAAATTAGAT AGATAAACTA TAAATATAGA GTTAAGTATA CTTATGTTTG GTTATCTTCG AATATTACCC   
  
  
+ GTTCGGATAT ATTATCTGAA CTGGTGAAAT AAGTAATATG TTTTGTTGTT TTAATTAGAT AATTTTTAGA   
  
  
+ CCGAGCTTGT GAATATATAC TAGACAAACA TTTATATTTC GAGTCTGCAC TTATATTCTA TAAGAGCTTG   
  
  
+ ATATATTAGA TTTGAACACT AACCTGTTAA TATAGTTTGC CGGTGATTTT TTTTCAAAAT TTTGATTCTT   
  
  
+ AGATATGTAT ATGGAGTAAA ACTAATTTTT ACAGATGCCC ATTTTTTTAA TTGACACTTA TGTAATTAAC   
  
  
+ TGAATTCATA AAACAAGGTT TTTTAAAAAA ATTTAACTCA TATCAATGAA ACAAAGACGA GAACGAAAGC   
  
  
+ ACAATTCTAT GGAAATGGAA AATGAAGTCA CTTATGGAGA TTCAATAGTA AGCAAATCGA GAGCAGAAAA   
  
  
+ TCTAATCTCC TTTCGTCATT ATACAATCAA TATTGCCTAT TTGGTTTTAG TGATTTGTTT CAGCCGCAAA   
  
  
+ ACTTAATTTT CTTTGGTGCA TATGAAATCT TAAAAAGAAT TAAAATAAGA TATAATACGT TAACTCTTCA   
  
  
+ ACAACATGAT ATATTTAAGA TACCAATATT TGTATTCATC ATATAAAAAT TGTAGTGTTG CAAAATATTA   
  
  
+ AAATTATTTC ATAAATAAAC ATTATTATAA GAACTGACTC CGCGGATTAT CATATGGTAT AGATTACAGA   
  
  
+ GTGGGTGGGT TTAAATAATT TCCCCGACAC ATTATACTTT TAGAAGAGCT ATTAGCTCCA AATTATTTAA   
  
  
+ ACAATGTTCT AAAGAGCAAA AAAATCAATG TTTTGGATTT TGATCCGACC GAGAGGACTT GTCCGACCAT   
  
  
+ TCCATATTAA AATGTTCCTA GTTCTGATTA GCTAATACCA   

- TATTTCAACA GCACTAAAGA ACGGGAAATA AAAAATAAAT ATAATTGTAT ATAAAAATTT TTTTTTAATT   
  
  
- TCTGCAAGGA TTGGCTTTGA GTACATAAAA AACTAATACT TATACTTATA GGGGAGATAT AATTAGTACC   
  
  
- TCGTAATGTT GTACAAAAGC ATCGGTATAC AGTAGTGCTC TTACTAAAAA TCTTAACAAT CTTTTTATTT   
  
  
- AACTAAGTAT ATTTGTATAT GATACAAAAA ATAATTTGAT TGATAGTTTA ATTAATTATC ACATGTTTTT   
  
  
- TTATAAAAAA AGAAAGGAAT TTATTTTTGA TGCCTTAATG GATTATACCG ATTGTATATA TACTGTTAAT   
  
  
- TACTAATACT TATTATGTAT AAACTATTTT TTTAAAGATT GGAGAGAGAA AAAACAAATT AAAATATAAT   
  
  
- AATTTCCTTT AAATTGTTAG TGTAATTAGT ATATTATTTT TGTTAATCTA AAAAAGAATA TACAATATAA   
  
  
- AACTTAAAAA TTTTTGCTGA TATTTAATGA TTTTTACTAT TCTCAGGGTG TAATTTTTTA AACACTAGTT   
  
  
- GGCAAATTGA AAAAAAAATC AAGTTCGTTC TATGTTTACT AGTATATAGA CTATATCTGC ACCCGCAAGC   
  
  
- CTATGTGCAA GCCCAAACAT AGTCTATAAA GTCATATTTC CATATCTTGG GCAAGCCCAT AAAGATGTGA   
  
  
- GGCTCAGCCC AAGCCCAAGC CTATAAAACC TAGCCCAAGC TTATAAATTT AAAACTTCTT TTTTTCTTTA   
  
  
- ATAAGTGACA AATTCAAAAA ATATAAATTT ATATAGAATT GAATTGACTA AAAAAAATCA AAAATTTTCT   
  
  
- AATTTTATAA TTATACAAAC CTCTATTTTG AAATTTTTAT CTTTCTGTGA TTAAATCAAA AACAAAACTT   
  
  
- TTAAATCTAC GTTGAAAACA ATTACGTTCT TTGTTCTTGA ACTATACATA AAATTCACTC ATTGTTTACT   
  
  
- AAAACAGATA TCAATATACA TATAATAGAT TAAAACTCAT TATTCTTAGT AATTATATTT ATAAAACTTA   
  
  
- TTTTAATCTA TCTATTTGAT ATTTATATCT CAATTCATAT GAATACAAAC CAATAGAAGC TTATAATGGG   
  
  
- CAAGCCTATA TAATAGACTT GACCACTTTA TTCATTATAC AAAACAACAA AATTAATCTA TTAAAAATCT   
  
  
- GGCTCGAACA CTTATATATG ATCTGTTTGT AAATATAAAG CTCAGACGTG AATATAAGAT ATTCTCGAAC   
  
  
- TATATAATCT AAACTTGTGA TTGGACAATT ATATCAAACG GCCACTAAAA AAAAGTTTTA AAACTAAGAA   
  
  
- TCTATACATA TACCTCATTT TGATTAAAAA TGTCTACGGG TAAAAAAATT AACTGTGAAT ACATTAATTG   
  
  
- ACTTAAGTAT TTTGTTCCAA AAAATTTTTT TAAATTGAGT ATAGTTACTT TGTTTCTGCT CTTGCTTTCG   
  
  
- TGTTAAGATA CCTTTACCTT TTACTTCAGT GAATACCTCT AAGTTATCAT TCGTTTAGCT CTCGTCTTTT   
  
  
- AGATTAGAGG AAAGCAGTAA TATGTTAGTT ATAACGGATA AACCAAAATC ACTAAACAAA GTCGGCGTTT   
  
  
- TGAATTAAAA GAAACCACGT ATACTTTAGA ATTTTTCTTA ATTTTATTCT ATATTATGCA ATTGAGAAGT   
  
  
- TGTTGTACTA TATAAATTCT ATGGTTATAA ACATAAGTAG TATATTTTTA ACATCACAAC GTTTTATAAT   
  
  
- TTTAATAAAG TATTTATTTG TAATAATATT CTTGACTGAG GCGCCTAATA GTATACCATA TCTAATGTCT   
  
  
- CACCCACCCA AATTTATTAA AGGGGCTGTG TAATATGAAA ATCTTCTCGA TAATCGAGGT TTAATAAATT   
  
  
- TGTTACAAGA TTTCTCGTTT TTTTAGTTAC AAAACCTAAA ACTAGGCTGG CTCTCCTGAA CAGGCTGGTA   
  
  
- AGGTATAATT TTACAAGGAT CAAGACTAAT CGATTATGGT

+     ATCT-motif

| Site Name | Organism | Position | Strand | Matrix score. | sequence | function |
| --- | --- | --- | --- | --- | --- | --- |
| ATCT-motif | Pisum sativum | 1539 | + | 9 | AATCTAATCC | part of a conserved DNA module involved in light responsiveness |

>PlantCARE\_25378   
+ ATAAAGTTGT CGTGATTTCT TGCCCTTTAT TTTTTATTTA TATTAACATA TATTTTTAAA AAAAAATTAA   
  
  
+ AGACGTTCCT AACCGAAACT CATGTATTTT TTGATTATGA ATATGAATAT CCCCTCTATA TTAATCATGG   
  
  
+ AGCATTACAA CATGTTTTCG TAGCCATATG TCATCACGAG AATGATTTTT AGAATTGTTA GAAAAATAAA   
  
  
+ TTGATTCATA TAAACATATA CTATGTTTTT TATTAAACTA ACTATCAAAT TAATTAATAG TGTACAAAAA   
  
  
+ AATATTTTTT TCTTTCCTTA AATAAAAACT ACGGAATTAC CTAATATGGC TAACATATAT ATGACAATTA   
  
  
+ ATGATTATGA ATAATACATA TTTGATAAAA AAATTTCTAA CCTCTCTCTT TTTTGTTTAA TTTTATATTA   
  
  
+ TTAAAGGAAA TTTAACAATC ACATTAATCA TATAATAAAA ACAATTAGAT TTTTTCTTAT ATGTTATATT   
  
  
+ TTGAATTTTT AAAAACGACT ATAAATTACT AAAAATGATA AGAGTCCCAC ATTAAAAAAT TTGTGATCAA   
  
  
+ CCGTTTAACT TTTTTTTTAG TTCAAGCAAG ATACAAATGA TCATATATCT GATATAGACG TGGGCGTTCG   
  
  
+ GATACACGTT CGGGTTTGTA TCAGATATTT CAGTATAAAG GTATAGAACC CGTTCGGGTA TTTCTACACT   
  
  
+ CCGAGTCGGG TTCGGGTTCG GATATTTTGG ATCGGGTTCG AATATTTAAA TTTTGAAGAA AAAAAGAAAT   
  
  
+ TATTCACTGT TTAAGTTTTT TATATTTAAA TATATCTTAA CTTAACTGAT TTTTTTTAGT TTTTAAAAGA   
  
  
+ TTAAAATATT AATATGTTTG GAGATAAAAC TTTAAAAATA GAAAGACACT AATTTAGTTT TTGTTTTGAA   
  
  
+ AATTTAGATG CAACTTTTGT TAATGCAAGA AACAAGAACT TGATATGTAT TTTAAGTGAG TAACAAATGA   
  
  
+ TTTTGTCTAT AGTTATATGT ATATTATCTA ATTTTGAGTA ATAAGAATCA TTAATATAAA TATTTTGAAT   
  
  
+ AAAATTAGAT AGATAAACTA TAAATATAGA GTTAAGTATA CTTATGTTTG GTTATCTTCG AATATTACCC   
  
  
+ GTTCGGATAT ATTATCTGAA CTGGTGAAAT AAGTAATATG TTTTGTTGTT TTAATTAGAT AATTTTTAGA   
  
  
+ CCGAGCTTGT GAATATATAC TAGACAAACA TTTATATTTC GAGTCTGCAC TTATATTCTA TAAGAGCTTG   
  
  
+ ATATATTAGA TTTGAACACT AACCTGTTAA TATAGTTTGC CGGTGATTTT TTTTCAAAAT TTTGATTCTT   
  
  
+ AGATATGTAT ATGGAGTAAA ACTAATTTTT ACAGATGCCC ATTTTTTTAA TTGACACTTA TGTAATTAAC   
  
  
+ TGAATTCATA AAACAAGGTT TTTTAAAAAA ATTTAACTCA TATCAATGAA ACAAAGACGA GAACGAAAGC   
  
  
+ ACAATTCTAT GGAAATGGAA AATGAAGTCA CTTATGGAGA TTCAATAGTA AGCAAATCGA GAGCAGAAAA   
  
  
+ TCTAATCTCC TTTCGTCATT ATACAATCAA TATTGCCTAT TTGGTTTTAG TGATTTGTTT CAGCCGCAAA   
  
  
+ ACTTAATTTT CTTTGGTGCA TATGAAATCT TAAAAAGAAT TAAAATAAGA TATAATACGT TAACTCTTCA   
  
  
+ ACAACATGAT ATATTTAAGA TACCAATATT TGTATTCATC ATATAAAAAT TGTAGTGTTG CAAAATATTA   
  
  
+ AAATTATTTC ATAAATAAAC ATTATTATAA GAACTGACTC CGCGGATTAT CATATGGTAT AGATTACAGA   
  
  
+ GTGGGTGGGT TTAAATAATT TCCCCGACAC ATTATACTTT TAGAAGAGCT ATTAGCTCCA AATTATTTAA   
  
  
+ ACAATGTTCT AAAGAGCAAA AAAATCAATG TTTTGGATTT TGATCCGACC GAGAGGACTT GTCCGACCAT   
  
  
+ TCCATATTAA AATGTTCCTA GTTCTGATTA GCTAATACCA   

- TATTTCAACA GCACTAAAGA ACGGGAAATA AAAAATAAAT ATAATTGTAT ATAAAAATTT TTTTTTAATT   
  
  
- TCTGCAAGGA TTGGCTTTGA GTACATAAAA AACTAATACT TATACTTATA GGGGAGATAT AATTAGTACC   
  
  
- TCGTAATGTT GTACAAAAGC ATCGGTATAC AGTAGTGCTC TTACTAAAAA TCTTAACAAT CTTTTTATTT   
  
  
- AACTAAGTAT ATTTGTATAT GATACAAAAA ATAATTTGAT TGATAGTTTA ATTAATTATC ACATGTTTTT   
  
  
- TTATAAAAAA AGAAAGGAAT TTATTTTTGA TGCCTTAATG GATTATACCG ATTGTATATA TACTGTTAAT   
  
  
- TACTAATACT TATTATGTAT AAACTATTTT TTTAAAGATT GGAGAGAGAA AAAACAAATT AAAATATAAT   
  
  
- AATTTCCTTT AAATTGTTAG TGTAATTAGT ATATTATTTT TGTTAATCTA AAAAAGAATA TACAATATAA   
  
  
- AACTTAAAAA TTTTTGCTGA TATTTAATGA TTTTTACTAT TCTCAGGGTG TAATTTTTTA AACACTAGTT   
  
  
- GGCAAATTGA AAAAAAAATC AAGTTCGTTC TATGTTTACT AGTATATAGA CTATATCTGC ACCCGCAAGC   
  
  
- CTATGTGCAA GCCCAAACAT AGTCTATAAA GTCATATTTC CATATCTTGG GCAAGCCCAT AAAGATGTGA   
  
  
- GGCTCAGCCC AAGCCCAAGC CTATAAAACC TAGCCCAAGC TTATAAATTT AAAACTTCTT TTTTTCTTTA   
  
  
- ATAAGTGACA AATTCAAAAA ATATAAATTT ATATAGAATT GAATTGACTA AAAAAAATCA AAAATTTTCT   
  
  
- AATTTTATAA TTATACAAAC CTCTATTTTG AAATTTTTAT CTTTCTGTGA TTAAATCAAA AACAAAACTT   
  
  
- TTAAATCTAC GTTGAAAACA ATTACGTTCT TTGTTCTTGA ACTATACATA AAATTCACTC ATTGTTTACT   
  
  
- AAAACAGATA TCAATATACA TATAATAGAT TAAAACTCAT TATTCTTAGT AATTATATTT ATAAAACTTA   
  
  
- TTTTAATCTA TCTATTTGAT ATTTATATCT CAATTCATAT GAATACAAAC CAATAGAAGC TTATAATGGG   
  
  
- CAAGCCTATA TAATAGACTT GACCACTTTA TTCATTATAC AAAACAACAA AATTAATCTA TTAAAAATCT   
  
  
- GGCTCGAACA CTTATATATG ATCTGTTTGT AAATATAAAG CTCAGACGTG AATATAAGAT ATTCTCGAAC   
  
  
- TATATAATCT AAACTTGTGA TTGGACAATT ATATCAAACG GCCACTAAAA AAAAGTTTTA AAACTAAGAA   
  
  
- TCTATACATA TACCTCATTT TGATTAAAAA TGTCTACGGG TAAAAAAATT AACTGTGAAT ACATTAATTG   
  
  
- ACTTAAGTAT TTTGTTCCAA AAAATTTTTT TAAATTGAGT ATAGTTACTT TGTTTCTGCT CTTGCTTTCG   
  
  
- TGTTAAGATA CCTTTACCTT TTACTTCAGT GAATACCTCT AAGTTATCAT TCGTTTAGCT CTCGTCTTTT   
  
  
- AGATTAGAGG AAAGCAGTAA TATGTTAGTT ATAACGGATA AACCAAAATC ACTAAACAAA GTCGGCGTTT   
  
  
- TGAATTAAAA GAAACCACGT ATACTTTAGA ATTTTTCTTA ATTTTATTCT ATATTATGCA ATTGAGAAGT   
  
  
- TGTTGTACTA TATAAATTCT ATGGTTATAA ACATAAGTAG TATATTTTTA ACATCACAAC GTTTTATAAT   
  
  
- TTTAATAAAG TATTTATTTG TAATAATATT CTTGACTGAG GCGCCTAATA GTATACCATA TCTAATGTCT   
  
  
- CACCCACCCA AATTTATTAA AGGGGCTGTG TAATATGAAA ATCTTCTCGA TAATCGAGGT TTAATAAATT   
  
  
- TGTTACAAGA TTTCTCGTTT TTTTAGTTAC AAAACCTAAA ACTAGGCTGG CTCTCCTGAA CAGGCTGGTA   
  
  
- AGGTATAATT TTACAAGGAT CAAGACTAAT CGATTATGGT

+     AT~TATA-box

| Site Name | Organism | Position | Strand | Matrix score. | sequence | function |
| --- | --- | --- | --- | --- | --- | --- |
| AT~TATA-box | Arabidopsis thaliana | 336 | + | 6 | TATATA |  |
| AT~TATA-box | Arabidopsis thaliana | 1204 | - | 6 | TATATA |  |

>PlantCARE\_25378   
+ ATAAAGTTGT CGTGATTTCT TGCCCTTTAT TTTTTATTTA TATTAACATA TATTTTTAAA AAAAAATTAA   
  
  
+ AGACGTTCCT AACCGAAACT CATGTATTTT TTGATTATGA ATATGAATAT CCCCTCTATA TTAATCATGG   
  
  
+ AGCATTACAA CATGTTTTCG TAGCCATATG TCATCACGAG AATGATTTTT AGAATTGTTA GAAAAATAAA   
  
  
+ TTGATTCATA TAAACATATA CTATGTTTTT TATTAAACTA ACTATCAAAT TAATTAATAG TGTACAAAAA   
  
  
+ AATATTTTTT TCTTTCCTTA AATAAAAACT ACGGAATTAC CTAATATGGC TAACATATAT ATGACAATTA   
  
  
+ ATGATTATGA ATAATACATA TTTGATAAAA AAATTTCTAA CCTCTCTCTT TTTTGTTTAA TTTTATATTA   
  
  
+ TTAAAGGAAA TTTAACAATC ACATTAATCA TATAATAAAA ACAATTAGAT TTTTTCTTAT ATGTTATATT   
  
  
+ TTGAATTTTT AAAAACGACT ATAAATTACT AAAAATGATA AGAGTCCCAC ATTAAAAAAT TTGTGATCAA   
  
  
+ CCGTTTAACT TTTTTTTTAG TTCAAGCAAG ATACAAATGA TCATATATCT GATATAGACG TGGGCGTTCG   
  
  
+ GATACACGTT CGGGTTTGTA TCAGATATTT CAGTATAAAG GTATAGAACC CGTTCGGGTA TTTCTACACT   
  
  
+ CCGAGTCGGG TTCGGGTTCG GATATTTTGG ATCGGGTTCG AATATTTAAA TTTTGAAGAA AAAAAGAAAT   
  
  
+ TATTCACTGT TTAAGTTTTT TATATTTAAA TATATCTTAA CTTAACTGAT TTTTTTTAGT TTTTAAAAGA   
  
  
+ TTAAAATATT AATATGTTTG GAGATAAAAC TTTAAAAATA GAAAGACACT AATTTAGTTT TTGTTTTGAA   
  
  
+ AATTTAGATG CAACTTTTGT TAATGCAAGA AACAAGAACT TGATATGTAT TTTAAGTGAG TAACAAATGA   
  
  
+ TTTTGTCTAT AGTTATATGT ATATTATCTA ATTTTGAGTA ATAAGAATCA TTAATATAAA TATTTTGAAT   
  
  
+ AAAATTAGAT AGATAAACTA TAAATATAGA GTTAAGTATA CTTATGTTTG GTTATCTTCG AATATTACCC   
  
  
+ GTTCGGATAT ATTATCTGAA CTGGTGAAAT AAGTAATATG TTTTGTTGTT TTAATTAGAT AATTTTTAGA   
  
  
+ CCGAGCTTGT GAATATATAC TAGACAAACA TTTATATTTC GAGTCTGCAC TTATATTCTA TAAGAGCTTG   
  
  
+ ATATATTAGA TTTGAACACT AACCTGTTAA TATAGTTTGC CGGTGATTTT TTTTCAAAAT TTTGATTCTT   
  
  
+ AGATATGTAT ATGGAGTAAA ACTAATTTTT ACAGATGCCC ATTTTTTTAA TTGACACTTA TGTAATTAAC   
  
  
+ TGAATTCATA AAACAAGGTT TTTTAAAAAA ATTTAACTCA TATCAATGAA ACAAAGACGA GAACGAAAGC   
  
  
+ ACAATTCTAT GGAAATGGAA AATGAAGTCA CTTATGGAGA TTCAATAGTA AGCAAATCGA GAGCAGAAAA   
  
  
+ TCTAATCTCC TTTCGTCATT ATACAATCAA TATTGCCTAT TTGGTTTTAG TGATTTGTTT CAGCCGCAAA   
  
  
+ ACTTAATTTT CTTTGGTGCA TATGAAATCT TAAAAAGAAT TAAAATAAGA TATAATACGT TAACTCTTCA   
  
  
+ ACAACATGAT ATATTTAAGA TACCAATATT TGTATTCATC ATATAAAAAT TGTAGTGTTG CAAAATATTA   
  
  
+ AAATTATTTC ATAAATAAAC ATTATTATAA GAACTGACTC CGCGGATTAT CATATGGTAT AGATTACAGA   
  
  
+ GTGGGTGGGT TTAAATAATT TCCCCGACAC ATTATACTTT TAGAAGAGCT ATTAGCTCCA AATTATTTAA   
  
  
+ ACAATGTTCT AAAGAGCAAA AAAATCAATG TTTTGGATTT TGATCCGACC GAGAGGACTT GTCCGACCAT   
  
  
+ TCCATATTAA AATGTTCCTA GTTCTGATTA GCTAATACCA   

- TATTTCAACA GCACTAAAGA ACGGGAAATA AAAAATAAAT ATAATTGTAT ATAAAAATTT TTTTTTAATT   
  
  
- TCTGCAAGGA TTGGCTTTGA GTACATAAAA AACTAATACT TATACTTATA GGGGAGATAT AATTAGTACC   
  
  
- TCGTAATGTT GTACAAAAGC ATCGGTATAC AGTAGTGCTC TTACTAAAAA TCTTAACAAT CTTTTTATTT   
  
  
- AACTAAGTAT ATTTGTATAT GATACAAAAA ATAATTTGAT TGATAGTTTA ATTAATTATC ACATGTTTTT   
  
  
- TTATAAAAAA AGAAAGGAAT TTATTTTTGA TGCCTTAATG GATTATACCG ATTGTATATA TACTGTTAAT   
  
  
- TACTAATACT TATTATGTAT AAACTATTTT TTTAAAGATT GGAGAGAGAA AAAACAAATT AAAATATAAT   
  
  
- AATTTCCTTT AAATTGTTAG TGTAATTAGT ATATTATTTT TGTTAATCTA AAAAAGAATA TACAATATAA   
  
  
- AACTTAAAAA TTTTTGCTGA TATTTAATGA TTTTTACTAT TCTCAGGGTG TAATTTTTTA AACACTAGTT   
  
  
- GGCAAATTGA AAAAAAAATC AAGTTCGTTC TATGTTTACT AGTATATAGA CTATATCTGC ACCCGCAAGC   
  
  
- CTATGTGCAA GCCCAAACAT AGTCTATAAA GTCATATTTC CATATCTTGG GCAAGCCCAT AAAGATGTGA   
  
  
- GGCTCAGCCC AAGCCCAAGC CTATAAAACC TAGCCCAAGC TTATAAATTT AAAACTTCTT TTTTTCTTTA   
  
  
- ATAAGTGACA AATTCAAAAA ATATAAATTT ATATAGAATT GAATTGACTA AAAAAAATCA AAAATTTTCT   
  
  
- AATTTTATAA TTATACAAAC CTCTATTTTG AAATTTTTAT CTTTCTGTGA TTAAATCAAA AACAAAACTT   
  
  
- TTAAATCTAC GTTGAAAACA ATTACGTTCT TTGTTCTTGA ACTATACATA AAATTCACTC ATTGTTTACT   
  
  
- AAAACAGATA TCAATATACA TATAATAGAT TAAAACTCAT TATTCTTAGT AATTATATTT ATAAAACTTA   
  
  
- TTTTAATCTA TCTATTTGAT ATTTATATCT CAATTCATAT GAATACAAAC CAATAGAAGC TTATAATGGG   
  
  
- CAAGCCTATA TAATAGACTT GACCACTTTA TTCATTATAC AAAACAACAA AATTAATCTA TTAAAAATCT   
  
  
- GGCTCGAACA CTTATATATG ATCTGTTTGT AAATATAAAG CTCAGACGTG AATATAAGAT ATTCTCGAAC   
  
  
- TATATAATCT AAACTTGTGA TTGGACAATT ATATCAAACG GCCACTAAAA AAAAGTTTTA AAACTAAGAA   
  
  
- TCTATACATA TACCTCATTT TGATTAAAAA TGTCTACGGG TAAAAAAATT AACTGTGAAT ACATTAATTG   
  
  
- ACTTAAGTAT TTTGTTCCAA AAAATTTTTT TAAATTGAGT ATAGTTACTT TGTTTCTGCT CTTGCTTTCG   
  
  
- TGTTAAGATA CCTTTACCTT TTACTTCAGT GAATACCTCT AAGTTATCAT TCGTTTAGCT CTCGTCTTTT   
  
  
- AGATTAGAGG AAAGCAGTAA TATGTTAGTT ATAACGGATA AACCAAAATC ACTAAACAAA GTCGGCGTTT   
  
  
- TGAATTAAAA GAAACCACGT ATACTTTAGA ATTTTTCTTA ATTTTATTCT ATATTATGCA ATTGAGAAGT   
  
  
- TGTTGTACTA TATAAATTCT ATGGTTATAA ACATAAGTAG TATATTTTTA ACATCACAAC GTTTTATAAT   
  
  
- TTTAATAAAG TATTTATTTG TAATAATATT CTTGACTGAG GCGCCTAATA GTATACCATA TCTAATGTCT   
  
  
- CACCCACCCA AATTTATTAA AGGGGCTGTG TAATATGAAA ATCTTCTCGA TAATCGAGGT TTAATAAATT   
  
  
- TGTTACAAGA TTTCTCGTTT TTTTAGTTAC AAAACCTAAA ACTAGGCTGG CTCTCCTGAA CAGGCTGGTA   
  
  
- AGGTATAATT TTACAAGGAT CAAGACTAAT CGATTATGGT

+     Box 4

| Site Name | Organism | Position | Strand | Matrix score. | sequence | function |
| --- | --- | --- | --- | --- | --- | --- |
| Box 4 | Petroselinum crispum | 130 | + | 6 | ATTAAT | part of a conserved DNA module involved in light responsiveness |
| Box 4 | Petroselinum crispum | 848 | + | 6 | ATTAAT | part of a conserved DNA module involved in light responsiveness |
| Box 4 | Petroselinum crispum | 263 | + | 6 | ATTAAT | part of a conserved DNA module involved in light responsiveness |
| Box 4 | Petroselinum crispum | 1030 | - | 6 | ATTAAT | part of a conserved DNA module involved in light responsiveness |
| Box 4 | Petroselinum crispum | 259 | + | 6 | ATTAAT | part of a conserved DNA module involved in light responsiveness |
| Box 4 | Petroselinum crispum | 443 | + | 6 | ATTAAT | part of a conserved DNA module involved in light responsiveness |
| Box 4 | Petroselinum crispum | 347 | + | 6 | ATTAAT | part of a conserved DNA module involved in light responsiveness |

>PlantCARE\_25378   
+ ATAAAGTTGT CGTGATTTCT TGCCCTTTAT TTTTTATTTA TATTAACATA TATTTTTAAA AAAAAATTAA   
  
  
+ AGACGTTCCT AACCGAAACT CATGTATTTT TTGATTATGA ATATGAATAT CCCCTCTATA TTAATCATGG   
  
  
+ AGCATTACAA CATGTTTTCG TAGCCATATG TCATCACGAG AATGATTTTT AGAATTGTTA GAAAAATAAA   
  
  
+ TTGATTCATA TAAACATATA CTATGTTTTT TATTAAACTA ACTATCAAAT TAATTAATAG TGTACAAAAA   
  
  
+ AATATTTTTT TCTTTCCTTA AATAAAAACT ACGGAATTAC CTAATATGGC TAACATATAT ATGACAATTA   
  
  
+ ATGATTATGA ATAATACATA TTTGATAAAA AAATTTCTAA CCTCTCTCTT TTTTGTTTAA TTTTATATTA   
  
  
+ TTAAAGGAAA TTTAACAATC ACATTAATCA TATAATAAAA ACAATTAGAT TTTTTCTTAT ATGTTATATT   
  
  
+ TTGAATTTTT AAAAACGACT ATAAATTACT AAAAATGATA AGAGTCCCAC ATTAAAAAAT TTGTGATCAA   
  
  
+ CCGTTTAACT TTTTTTTTAG TTCAAGCAAG ATACAAATGA TCATATATCT GATATAGACG TGGGCGTTCG   
  
  
+ GATACACGTT CGGGTTTGTA TCAGATATTT CAGTATAAAG GTATAGAACC CGTTCGGGTA TTTCTACACT   
  
  
+ CCGAGTCGGG TTCGGGTTCG GATATTTTGG ATCGGGTTCG AATATTTAAA TTTTGAAGAA AAAAAGAAAT   
  
  
+ TATTCACTGT TTAAGTTTTT TATATTTAAA TATATCTTAA CTTAACTGAT TTTTTTTAGT TTTTAAAAGA   
  
  
+ TTAAAATATT AATATGTTTG GAGATAAAAC TTTAAAAATA GAAAGACACT AATTTAGTTT TTGTTTTGAA   
  
  
+ AATTTAGATG CAACTTTTGT TAATGCAAGA AACAAGAACT TGATATGTAT TTTAAGTGAG TAACAAATGA   
  
  
+ TTTTGTCTAT AGTTATATGT ATATTATCTA ATTTTGAGTA ATAAGAATCA TTAATATAAA TATTTTGAAT   
  
  
+ AAAATTAGAT AGATAAACTA TAAATATAGA GTTAAGTATA CTTATGTTTG GTTATCTTCG AATATTACCC   
  
  
+ GTTCGGATAT ATTATCTGAA CTGGTGAAAT AAGTAATATG TTTTGTTGTT TTAATTAGAT AATTTTTAGA   
  
  
+ CCGAGCTTGT GAATATATAC TAGACAAACA TTTATATTTC GAGTCTGCAC TTATATTCTA TAAGAGCTTG   
  
  
+ ATATATTAGA TTTGAACACT AACCTGTTAA TATAGTTTGC CGGTGATTTT TTTTCAAAAT TTTGATTCTT   
  
  
+ AGATATGTAT ATGGAGTAAA ACTAATTTTT ACAGATGCCC ATTTTTTTAA TTGACACTTA TGTAATTAAC   
  
  
+ TGAATTCATA AAACAAGGTT TTTTAAAAAA ATTTAACTCA TATCAATGAA ACAAAGACGA GAACGAAAGC   
  
  
+ ACAATTCTAT GGAAATGGAA AATGAAGTCA CTTATGGAGA TTCAATAGTA AGCAAATCGA GAGCAGAAAA   
  
  
+ TCTAATCTCC TTTCGTCATT ATACAATCAA TATTGCCTAT TTGGTTTTAG TGATTTGTTT CAGCCGCAAA   
  
  
+ ACTTAATTTT CTTTGGTGCA TATGAAATCT TAAAAAGAAT TAAAATAAGA TATAATACGT TAACTCTTCA   
  
  
+ ACAACATGAT ATATTTAAGA TACCAATATT TGTATTCATC ATATAAAAAT TGTAGTGTTG CAAAATATTA   
  
  
+ AAATTATTTC ATAAATAAAC ATTATTATAA GAACTGACTC CGCGGATTAT CATATGGTAT AGATTACAGA   
  
  
+ GTGGGTGGGT TTAAATAATT TCCCCGACAC ATTATACTTT TAGAAGAGCT ATTAGCTCCA AATTATTTAA   
  
  
+ ACAATGTTCT AAAGAGCAAA AAAATCAATG TTTTGGATTT TGATCCGACC GAGAGGACTT GTCCGACCAT   
  
  
+ TCCATATTAA AATGTTCCTA GTTCTGATTA GCTAATACCA   

- TATTTCAACA GCACTAAAGA ACGGGAAATA AAAAATAAAT ATAATTGTAT ATAAAAATTT TTTTTTAATT   
  
  
- TCTGCAAGGA TTGGCTTTGA GTACATAAAA AACTAATACT TATACTTATA GGGGAGATAT AATTAGTACC   
  
  
- TCGTAATGTT GTACAAAAGC ATCGGTATAC AGTAGTGCTC TTACTAAAAA TCTTAACAAT CTTTTTATTT   
  
  
- AACTAAGTAT ATTTGTATAT GATACAAAAA ATAATTTGAT TGATAGTTTA ATTAATTATC ACATGTTTTT   
  
  
- TTATAAAAAA AGAAAGGAAT TTATTTTTGA TGCCTTAATG GATTATACCG ATTGTATATA TACTGTTAAT   
  
  
- TACTAATACT TATTATGTAT AAACTATTTT TTTAAAGATT GGAGAGAGAA AAAACAAATT AAAATATAAT   
  
  
- AATTTCCTTT AAATTGTTAG TGTAATTAGT ATATTATTTT TGTTAATCTA AAAAAGAATA TACAATATAA   
  
  
- AACTTAAAAA TTTTTGCTGA TATTTAATGA TTTTTACTAT TCTCAGGGTG TAATTTTTTA AACACTAGTT   
  
  
- GGCAAATTGA AAAAAAAATC AAGTTCGTTC TATGTTTACT AGTATATAGA CTATATCTGC ACCCGCAAGC   
  
  
- CTATGTGCAA GCCCAAACAT AGTCTATAAA GTCATATTTC CATATCTTGG GCAAGCCCAT AAAGATGTGA   
  
  
- GGCTCAGCCC AAGCCCAAGC CTATAAAACC TAGCCCAAGC TTATAAATTT AAAACTTCTT TTTTTCTTTA   
  
  
- ATAAGTGACA AATTCAAAAA ATATAAATTT ATATAGAATT GAATTGACTA AAAAAAATCA AAAATTTTCT   
  
  
- AATTTTATAA TTATACAAAC CTCTATTTTG AAATTTTTAT CTTTCTGTGA TTAAATCAAA AACAAAACTT   
  
  
- TTAAATCTAC GTTGAAAACA ATTACGTTCT TTGTTCTTGA ACTATACATA AAATTCACTC ATTGTTTACT   
  
  
- AAAACAGATA TCAATATACA TATAATAGAT TAAAACTCAT TATTCTTAGT AATTATATTT ATAAAACTTA   
  
  
- TTTTAATCTA TCTATTTGAT ATTTATATCT CAATTCATAT GAATACAAAC CAATAGAAGC TTATAATGGG   
  
  
- CAAGCCTATA TAATAGACTT GACCACTTTA TTCATTATAC AAAACAACAA AATTAATCTA TTAAAAATCT   
  
  
- GGCTCGAACA CTTATATATG ATCTGTTTGT AAATATAAAG CTCAGACGTG AATATAAGAT ATTCTCGAAC   
  
  
- TATATAATCT AAACTTGTGA TTGGACAATT ATATCAAACG GCCACTAAAA AAAAGTTTTA AAACTAAGAA   
  
  
- TCTATACATA TACCTCATTT TGATTAAAAA TGTCTACGGG TAAAAAAATT AACTGTGAAT ACATTAATTG   
  
  
- ACTTAAGTAT TTTGTTCCAA AAAATTTTTT TAAATTGAGT ATAGTTACTT TGTTTCTGCT CTTGCTTTCG   
  
  
- TGTTAAGATA CCTTTACCTT TTACTTCAGT GAATACCTCT AAGTTATCAT TCGTTTAGCT CTCGTCTTTT   
  
  
- AGATTAGAGG AAAGCAGTAA TATGTTAGTT ATAACGGATA AACCAAAATC ACTAAACAAA GTCGGCGTTT   
  
  
- TGAATTAAAA GAAACCACGT ATACTTTAGA ATTTTTCTTA ATTTTATTCT ATATTATGCA ATTGAGAAGT   
  
  
- TGTTGTACTA TATAAATTCT ATGGTTATAA ACATAAGTAG TATATTTTTA ACATCACAAC GTTTTATAAT   
  
  
- TTTAATAAAG TATTTATTTG TAATAATATT CTTGACTGAG GCGCCTAATA GTATACCATA TCTAATGTCT   
  
  
- CACCCACCCA AATTTATTAA AGGGGCTGTG TAATATGAAA ATCTTCTCGA TAATCGAGGT TTAATAAATT   
  
  
- TGTTACAAGA TTTCTCGTTT TTTTAGTTAC AAAACCTAAA ACTAGGCTGG CTCTCCTGAA CAGGCTGGTA   
  
  
- AGGTATAATT TTACAAGGAT CAAGACTAAT CGATTATGGT

+     CAAT-box

| Site Name | Organism | Position | Strand | Matrix score. | sequence | function |
| --- | --- | --- | --- | --- | --- | --- |
| CAAT-box | Nicotiana glutinosa | 194 | - | 4 | CAAT |  |
| CAAT-box | Nicotiana glutinosa | 210 | - | 4 | CAAT |  |
| CAAT-box | Pisum sativum | 256 | + | 5 | CAAAT | common cis-acting element in promoter and enhancer regions |
| CAAT-box | Nicotiana glutinosa | 345 | + | 4 | CAAT |  |
| CAAT-box | Pisum sativum | 370 | - | 5 | CAAAT | common cis-acting element in promoter and enhancer regions |
| CAAT-box | Nicotiana glutinosa | 436 | + | 4 | CAAT |  |
| CAAT-box | Nicotiana glutinosa | 462 | + | 4 | CAAT |  |
| CAAT-box | Pisum sativum | 549 | - | 5 | CAAAT | common cis-acting element in promoter and enhancer regions |
| CAAT-box | Pisum sativum | 594 | + | 5 | CAAAT | common cis-acting element in promoter and enhancer regions |
| CAAT-box | Pisum sativum | 974 | + | 5 | CAAAT | common cis-acting element in promoter and enhancer regions |
| CAAT-box | Pisum sativum | 1270 | - | 5 | CAAAT | common cis-acting element in promoter and enhancer regions |
| CAAT-box | Nicotiana glutinosa | 1380 | - | 4 | CAAT |  |
| CAAT-box | Nicotiana glutinosa | 1444 | + | 4 | CAAT |  |
| CAAT-box | Nicotiana glutinosa | 1472 | + | 4 | CAAT |  |
| CAAT-box | Nicotiana glutinosa | 1513 | + | 4 | CAAT |  |
| CAAT-box | Pisum sativum | 1523 | + | 5 | CAAAT | common cis-acting element in promoter and enhancer regions |
| CAAT-box | Nicotiana glutinosa | 1564 | + | 4 | CAAT |  |
| CAAT-box | Nicotiana glutinosa | 1568 | + | 4 | CAAT |  |
| CAAT-box | Nicotiana glutinosa | 1572 | - | 4 | CAAT |  |
| CAAT-box | Pisum sativum | 1579 | - | 5 | CAAAT | common cis-acting element in promoter and enhancer regions |
| CAAT-box | Pisum sativum | 1593 | - | 5 | CAAAT | common cis-acting element in promoter and enhancer regions |
| CAAT-box | Arabidopsis thaliana | 1703 | + | 5 | CCAAT | common cis-acting element in promoter and enhancer regions |
| CAAT-box | Nicotiana glutinosa | 1704 | + | 4 | CAAT |  |
| CAAT-box | Pisum sativum | 1708 | - | 5 | CAAAT | common cis-acting element in promoter and enhancer regions |
| CAAT-box | Nicotiana glutinosa | 1729 | - | 4 | CAAT |  |
| CAAT-box | Pisum sativum | 1879 | + | 5 | CAAAT | common cis-acting element in promoter and enhancer regions |
| CAAT-box | Nicotiana glutinosa | 1892 | + | 4 | CAAT |  |
| CAAT-box | Nicotiana glutinosa | 1916 | + | 4 | CAAT |  |

>PlantCARE\_25378   
+ ATAAAGTTGT CGTGATTTCT TGCCCTTTAT TTTTTATTTA TATTAACATA TATTTTTAAA AAAAAATTAA   
  
  
+ AGACGTTCCT AACCGAAACT CATGTATTTT TTGATTATGA ATATGAATAT CCCCTCTATA TTAATCATGG   
  
  
+ AGCATTACAA CATGTTTTCG TAGCCATATG TCATCACGAG AATGATTTTT AGAATTGTTA GAAAAATAAA   
  
  
+ TTGATTCATA TAAACATATA CTATGTTTTT TATTAAACTA ACTATCAAAT TAATTAATAG TGTACAAAAA   
  
  
+ AATATTTTTT TCTTTCCTTA AATAAAAACT ACGGAATTAC CTAATATGGC TAACATATAT ATGACAATTA   
  
  
+ ATGATTATGA ATAATACATA TTTGATAAAA AAATTTCTAA CCTCTCTCTT TTTTGTTTAA TTTTATATTA   
  
  
+ TTAAAGGAAA TTTAACAATC ACATTAATCA TATAATAAAA ACAATTAGAT TTTTTCTTAT ATGTTATATT   
  
  
+ TTGAATTTTT AAAAACGACT ATAAATTACT AAAAATGATA AGAGTCCCAC ATTAAAAAAT TTGTGATCAA   
  
  
+ CCGTTTAACT TTTTTTTTAG TTCAAGCAAG ATACAAATGA TCATATATCT GATATAGACG TGGGCGTTCG   
  
  
+ GATACACGTT CGGGTTTGTA TCAGATATTT CAGTATAAAG GTATAGAACC CGTTCGGGTA TTTCTACACT   
  
  
+ CCGAGTCGGG TTCGGGTTCG GATATTTTGG ATCGGGTTCG AATATTTAAA TTTTGAAGAA AAAAAGAAAT   
  
  
+ TATTCACTGT TTAAGTTTTT TATATTTAAA TATATCTTAA CTTAACTGAT TTTTTTTAGT TTTTAAAAGA   
  
  
+ TTAAAATATT AATATGTTTG GAGATAAAAC TTTAAAAATA GAAAGACACT AATTTAGTTT TTGTTTTGAA   
  
  
+ AATTTAGATG CAACTTTTGT TAATGCAAGA AACAAGAACT TGATATGTAT TTTAAGTGAG TAACAAATGA   
  
  
+ TTTTGTCTAT AGTTATATGT ATATTATCTA ATTTTGAGTA ATAAGAATCA TTAATATAAA TATTTTGAAT   
  
  
+ AAAATTAGAT AGATAAACTA TAAATATAGA GTTAAGTATA CTTATGTTTG GTTATCTTCG AATATTACCC   
  
  
+ GTTCGGATAT ATTATCTGAA CTGGTGAAAT AAGTAATATG TTTTGTTGTT TTAATTAGAT AATTTTTAGA   
  
  
+ CCGAGCTTGT GAATATATAC TAGACAAACA TTTATATTTC GAGTCTGCAC TTATATTCTA TAAGAGCTTG   
  
  
+ ATATATTAGA TTTGAACACT AACCTGTTAA TATAGTTTGC CGGTGATTTT TTTTCAAAAT TTTGATTCTT   
  
  
+ AGATATGTAT ATGGAGTAAA ACTAATTTTT ACAGATGCCC ATTTTTTTAA TTGACACTTA TGTAATTAAC   
  
  
+ TGAATTCATA AAACAAGGTT TTTTAAAAAA ATTTAACTCA TATCAATGAA ACAAAGACGA GAACGAAAGC   
  
  
+ ACAATTCTAT GGAAATGGAA AATGAAGTCA CTTATGGAGA TTCAATAGTA AGCAAATCGA GAGCAGAAAA   
  
  
+ TCTAATCTCC TTTCGTCATT ATACAATCAA TATTGCCTAT TTGGTTTTAG TGATTTGTTT CAGCCGCAAA   
  
  
+ ACTTAATTTT CTTTGGTGCA TATGAAATCT TAAAAAGAAT TAAAATAAGA TATAATACGT TAACTCTTCA   
  
  
+ ACAACATGAT ATATTTAAGA TACCAATATT TGTATTCATC ATATAAAAAT TGTAGTGTTG CAAAATATTA   
  
  
+ AAATTATTTC ATAAATAAAC ATTATTATAA GAACTGACTC CGCGGATTAT CATATGGTAT AGATTACAGA   
  
  
+ GTGGGTGGGT TTAAATAATT TCCCCGACAC ATTATACTTT TAGAAGAGCT ATTAGCTCCA AATTATTTAA   
  
  
+ ACAATGTTCT AAAGAGCAAA AAAATCAATG TTTTGGATTT TGATCCGACC GAGAGGACTT GTCCGACCAT   
  
  
+ TCCATATTAA AATGTTCCTA GTTCTGATTA GCTAATACCA   

- TATTTCAACA GCACTAAAGA ACGGGAAATA AAAAATAAAT ATAATTGTAT ATAAAAATTT TTTTTTAATT   
  
  
- TCTGCAAGGA TTGGCTTTGA GTACATAAAA AACTAATACT TATACTTATA GGGGAGATAT AATTAGTACC   
  
  
- TCGTAATGTT GTACAAAAGC ATCGGTATAC AGTAGTGCTC TTACTAAAAA TCTTAACAAT CTTTTTATTT   
  
  
- AACTAAGTAT ATTTGTATAT GATACAAAAA ATAATTTGAT TGATAGTTTA ATTAATTATC ACATGTTTTT   
  
  
- TTATAAAAAA AGAAAGGAAT TTATTTTTGA TGCCTTAATG GATTATACCG ATTGTATATA TACTGTTAAT   
  
  
- TACTAATACT TATTATGTAT AAACTATTTT TTTAAAGATT GGAGAGAGAA AAAACAAATT AAAATATAAT   
  
  
- AATTTCCTTT AAATTGTTAG TGTAATTAGT ATATTATTTT TGTTAATCTA AAAAAGAATA TACAATATAA   
  
  
- AACTTAAAAA TTTTTGCTGA TATTTAATGA TTTTTACTAT TCTCAGGGTG TAATTTTTTA AACACTAGTT   
  
  
- GGCAAATTGA AAAAAAAATC AAGTTCGTTC TATGTTTACT AGTATATAGA CTATATCTGC ACCCGCAAGC   
  
  
- CTATGTGCAA GCCCAAACAT AGTCTATAAA GTCATATTTC CATATCTTGG GCAAGCCCAT AAAGATGTGA   
  
  
- GGCTCAGCCC AAGCCCAAGC CTATAAAACC TAGCCCAAGC TTATAAATTT AAAACTTCTT TTTTTCTTTA   
  
  
- ATAAGTGACA AATTCAAAAA ATATAAATTT ATATAGAATT GAATTGACTA AAAAAAATCA AAAATTTTCT   
  
  
- AATTTTATAA TTATACAAAC CTCTATTTTG AAATTTTTAT CTTTCTGTGA TTAAATCAAA AACAAAACTT   
  
  
- TTAAATCTAC GTTGAAAACA ATTACGTTCT TTGTTCTTGA ACTATACATA AAATTCACTC ATTGTTTACT   
  
  
- AAAACAGATA TCAATATACA TATAATAGAT TAAAACTCAT TATTCTTAGT AATTATATTT ATAAAACTTA   
  
  
- TTTTAATCTA TCTATTTGAT ATTTATATCT CAATTCATAT GAATACAAAC CAATAGAAGC TTATAATGGG   
  
  
- CAAGCCTATA TAATAGACTT GACCACTTTA TTCATTATAC AAAACAACAA AATTAATCTA TTAAAAATCT   
  
  
- GGCTCGAACA CTTATATATG ATCTGTTTGT AAATATAAAG CTCAGACGTG AATATAAGAT ATTCTCGAAC   
  
  
- TATATAATCT AAACTTGTGA TTGGACAATT ATATCAAACG GCCACTAAAA AAAAGTTTTA AAACTAAGAA   
  
  
- TCTATACATA TACCTCATTT TGATTAAAAA TGTCTACGGG TAAAAAAATT AACTGTGAAT ACATTAATTG   
  
  
- ACTTAAGTAT TTTGTTCCAA AAAATTTTTT TAAATTGAGT ATAGTTACTT TGTTTCTGCT CTTGCTTTCG   
  
  
- TGTTAAGATA CCTTTACCTT TTACTTCAGT GAATACCTCT AAGTTATCAT TCGTTTAGCT CTCGTCTTTT   
  
  
- AGATTAGAGG AAAGCAGTAA TATGTTAGTT ATAACGGATA AACCAAAATC ACTAAACAAA GTCGGCGTTT   
  
  
- TGAATTAAAA GAAACCACGT ATACTTTAGA ATTTTTCTTA ATTTTATTCT ATATTATGCA ATTGAGAAGT   
  
  
- TGTTGTACTA TATAAATTCT ATGGTTATAA ACATAAGTAG TATATTTTTA ACATCACAAC GTTTTATAAT   
  
  
- TTTAATAAAG TATTTATTTG TAATAATATT CTTGACTGAG GCGCCTAATA GTATACCATA TCTAATGTCT   
  
  
- CACCCACCCA AATTTATTAA AGGGGCTGTG TAATATGAAA ATCTTCTCGA TAATCGAGGT TTAATAAATT   
  
  
- TGTTACAAGA TTTCTCGTTT TTTTAGTTAC AAAACCTAAA ACTAGGCTGG CTCTCCTGAA CAGGCTGGTA   
  
  
- AGGTATAATT TTACAAGGAT CAAGACTAAT CGATTATGGT

+     CGTCA-motif

| Site Name | Organism | Position | Strand | Matrix score. | sequence | function |
| --- | --- | --- | --- | --- | --- | --- |
| CGTCA-motif | Hordeum vulgare | 1554 | + | 5 | CGTCA | cis-acting regulatory element involved in the MeJA-responsiveness |

>PlantCARE\_25378   
+ ATAAAGTTGT CGTGATTTCT TGCCCTTTAT TTTTTATTTA TATTAACATA TATTTTTAAA AAAAAATTAA   
  
  
+ AGACGTTCCT AACCGAAACT CATGTATTTT TTGATTATGA ATATGAATAT CCCCTCTATA TTAATCATGG   
  
  
+ AGCATTACAA CATGTTTTCG TAGCCATATG TCATCACGAG AATGATTTTT AGAATTGTTA GAAAAATAAA   
  
  
+ TTGATTCATA TAAACATATA CTATGTTTTT TATTAAACTA ACTATCAAAT TAATTAATAG TGTACAAAAA   
  
  
+ AATATTTTTT TCTTTCCTTA AATAAAAACT ACGGAATTAC CTAATATGGC TAACATATAT ATGACAATTA   
  
  
+ ATGATTATGA ATAATACATA TTTGATAAAA AAATTTCTAA CCTCTCTCTT TTTTGTTTAA TTTTATATTA   
  
  
+ TTAAAGGAAA TTTAACAATC ACATTAATCA TATAATAAAA ACAATTAGAT TTTTTCTTAT ATGTTATATT   
  
  
+ TTGAATTTTT AAAAACGACT ATAAATTACT AAAAATGATA AGAGTCCCAC ATTAAAAAAT TTGTGATCAA   
  
  
+ CCGTTTAACT TTTTTTTTAG TTCAAGCAAG ATACAAATGA TCATATATCT GATATAGACG TGGGCGTTCG   
  
  
+ GATACACGTT CGGGTTTGTA TCAGATATTT CAGTATAAAG GTATAGAACC CGTTCGGGTA TTTCTACACT   
  
  
+ CCGAGTCGGG TTCGGGTTCG GATATTTTGG ATCGGGTTCG AATATTTAAA TTTTGAAGAA AAAAAGAAAT   
  
  
+ TATTCACTGT TTAAGTTTTT TATATTTAAA TATATCTTAA CTTAACTGAT TTTTTTTAGT TTTTAAAAGA   
  
  
+ TTAAAATATT AATATGTTTG GAGATAAAAC TTTAAAAATA GAAAGACACT AATTTAGTTT TTGTTTTGAA   
  
  
+ AATTTAGATG CAACTTTTGT TAATGCAAGA AACAAGAACT TGATATGTAT TTTAAGTGAG TAACAAATGA   
  
  
+ TTTTGTCTAT AGTTATATGT ATATTATCTA ATTTTGAGTA ATAAGAATCA TTAATATAAA TATTTTGAAT   
  
  
+ AAAATTAGAT AGATAAACTA TAAATATAGA GTTAAGTATA CTTATGTTTG GTTATCTTCG AATATTACCC   
  
  
+ GTTCGGATAT ATTATCTGAA CTGGTGAAAT AAGTAATATG TTTTGTTGTT TTAATTAGAT AATTTTTAGA   
  
  
+ CCGAGCTTGT GAATATATAC TAGACAAACA TTTATATTTC GAGTCTGCAC TTATATTCTA TAAGAGCTTG   
  
  
+ ATATATTAGA TTTGAACACT AACCTGTTAA TATAGTTTGC CGGTGATTTT TTTTCAAAAT TTTGATTCTT   
  
  
+ AGATATGTAT ATGGAGTAAA ACTAATTTTT ACAGATGCCC ATTTTTTTAA TTGACACTTA TGTAATTAAC   
  
  
+ TGAATTCATA AAACAAGGTT TTTTAAAAAA ATTTAACTCA TATCAATGAA ACAAAGACGA GAACGAAAGC   
  
  
+ ACAATTCTAT GGAAATGGAA AATGAAGTCA CTTATGGAGA TTCAATAGTA AGCAAATCGA GAGCAGAAAA   
  
  
+ TCTAATCTCC TTTCGTCATT ATACAATCAA TATTGCCTAT TTGGTTTTAG TGATTTGTTT CAGCCGCAAA   
  
  
+ ACTTAATTTT CTTTGGTGCA TATGAAATCT TAAAAAGAAT TAAAATAAGA TATAATACGT TAACTCTTCA   
  
  
+ ACAACATGAT ATATTTAAGA TACCAATATT TGTATTCATC ATATAAAAAT TGTAGTGTTG CAAAATATTA   
  
  
+ AAATTATTTC ATAAATAAAC ATTATTATAA GAACTGACTC CGCGGATTAT CATATGGTAT AGATTACAGA   
  
  
+ GTGGGTGGGT TTAAATAATT TCCCCGACAC ATTATACTTT TAGAAGAGCT ATTAGCTCCA AATTATTTAA   
  
  
+ ACAATGTTCT AAAGAGCAAA AAAATCAATG TTTTGGATTT TGATCCGACC GAGAGGACTT GTCCGACCAT   
  
  
+ TCCATATTAA AATGTTCCTA GTTCTGATTA GCTAATACCA   

- TATTTCAACA GCACTAAAGA ACGGGAAATA AAAAATAAAT ATAATTGTAT ATAAAAATTT TTTTTTAATT   
  
  
- TCTGCAAGGA TTGGCTTTGA GTACATAAAA AACTAATACT TATACTTATA GGGGAGATAT AATTAGTACC   
  
  
- TCGTAATGTT GTACAAAAGC ATCGGTATAC AGTAGTGCTC TTACTAAAAA TCTTAACAAT CTTTTTATTT   
  
  
- AACTAAGTAT ATTTGTATAT GATACAAAAA ATAATTTGAT TGATAGTTTA ATTAATTATC ACATGTTTTT   
  
  
- TTATAAAAAA AGAAAGGAAT TTATTTTTGA TGCCTTAATG GATTATACCG ATTGTATATA TACTGTTAAT   
  
  
- TACTAATACT TATTATGTAT AAACTATTTT TTTAAAGATT GGAGAGAGAA AAAACAAATT AAAATATAAT   
  
  
- AATTTCCTTT AAATTGTTAG TGTAATTAGT ATATTATTTT TGTTAATCTA AAAAAGAATA TACAATATAA   
  
  
- AACTTAAAAA TTTTTGCTGA TATTTAATGA TTTTTACTAT TCTCAGGGTG TAATTTTTTA AACACTAGTT   
  
  
- GGCAAATTGA AAAAAAAATC AAGTTCGTTC TATGTTTACT AGTATATAGA CTATATCTGC ACCCGCAAGC   
  
  
- CTATGTGCAA GCCCAAACAT AGTCTATAAA GTCATATTTC CATATCTTGG GCAAGCCCAT AAAGATGTGA   
  
  
- GGCTCAGCCC AAGCCCAAGC CTATAAAACC TAGCCCAAGC TTATAAATTT AAAACTTCTT TTTTTCTTTA   
  
  
- ATAAGTGACA AATTCAAAAA ATATAAATTT ATATAGAATT GAATTGACTA AAAAAAATCA AAAATTTTCT   
  
  
- AATTTTATAA TTATACAAAC CTCTATTTTG AAATTTTTAT CTTTCTGTGA TTAAATCAAA AACAAAACTT   
  
  
- TTAAATCTAC GTTGAAAACA ATTACGTTCT TTGTTCTTGA ACTATACATA AAATTCACTC ATTGTTTACT   
  
  
- AAAACAGATA TCAATATACA TATAATAGAT TAAAACTCAT TATTCTTAGT AATTATATTT ATAAAACTTA   
  
  
- TTTTAATCTA TCTATTTGAT ATTTATATCT CAATTCATAT GAATACAAAC CAATAGAAGC TTATAATGGG   
  
  
- CAAGCCTATA TAATAGACTT GACCACTTTA TTCATTATAC AAAACAACAA AATTAATCTA TTAAAAATCT   
  
  
- GGCTCGAACA CTTATATATG ATCTGTTTGT AAATATAAAG CTCAGACGTG AATATAAGAT ATTCTCGAAC   
  
  
- TATATAATCT AAACTTGTGA TTGGACAATT ATATCAAACG GCCACTAAAA AAAAGTTTTA AAACTAAGAA   
  
  
- TCTATACATA TACCTCATTT TGATTAAAAA TGTCTACGGG TAAAAAAATT AACTGTGAAT ACATTAATTG   
  
  
- ACTTAAGTAT TTTGTTCCAA AAAATTTTTT TAAATTGAGT ATAGTTACTT TGTTTCTGCT CTTGCTTTCG   
  
  
- TGTTAAGATA CCTTTACCTT TTACTTCAGT GAATACCTCT AAGTTATCAT TCGTTTAGCT CTCGTCTTTT   
  
  
- AGATTAGAGG AAAGCAGTAA TATGTTAGTT ATAACGGATA AACCAAAATC ACTAAACAAA GTCGGCGTTT   
  
  
- TGAATTAAAA GAAACCACGT ATACTTTAGA ATTTTTCTTA ATTTTATTCT ATATTATGCA ATTGAGAAGT   
  
  
- TGTTGTACTA TATAAATTCT ATGGTTATAA ACATAAGTAG TATATTTTTA ACATCACAAC GTTTTATAAT   
  
  
- TTTAATAAAG TATTTATTTG TAATAATATT CTTGACTGAG GCGCCTAATA GTATACCATA TCTAATGTCT   
  
  
- CACCCACCCA AATTTATTAA AGGGGCTGTG TAATATGAAA ATCTTCTCGA TAATCGAGGT TTAATAAATT   
  
  
- TGTTACAAGA TTTCTCGTTT TTTTAGTTAC AAAACCTAAA ACTAGGCTGG CTCTCCTGAA CAGGCTGGTA   
  
  
- AGGTATAATT TTACAAGGAT CAAGACTAAT CGATTATGGT

+     DRE1

| Site Name | Organism | Position | Strand | Matrix score. | sequence | function |
| --- | --- | --- | --- | --- | --- | --- |
| DRE1 | Zea mays | 1938 | + | 7 | ACCGAGA |  |

>PlantCARE\_25378   
+ ATAAAGTTGT CGTGATTTCT TGCCCTTTAT TTTTTATTTA TATTAACATA TATTTTTAAA AAAAAATTAA   
  
  
+ AGACGTTCCT AACCGAAACT CATGTATTTT TTGATTATGA ATATGAATAT CCCCTCTATA TTAATCATGG   
  
  
+ AGCATTACAA CATGTTTTCG TAGCCATATG TCATCACGAG AATGATTTTT AGAATTGTTA GAAAAATAAA   
  
  
+ TTGATTCATA TAAACATATA CTATGTTTTT TATTAAACTA ACTATCAAAT TAATTAATAG TGTACAAAAA   
  
  
+ AATATTTTTT TCTTTCCTTA AATAAAAACT ACGGAATTAC CTAATATGGC TAACATATAT ATGACAATTA   
  
  
+ ATGATTATGA ATAATACATA TTTGATAAAA AAATTTCTAA CCTCTCTCTT TTTTGTTTAA TTTTATATTA   
  
  
+ TTAAAGGAAA TTTAACAATC ACATTAATCA TATAATAAAA ACAATTAGAT TTTTTCTTAT ATGTTATATT   
  
  
+ TTGAATTTTT AAAAACGACT ATAAATTACT AAAAATGATA AGAGTCCCAC ATTAAAAAAT TTGTGATCAA   
  
  
+ CCGTTTAACT TTTTTTTTAG TTCAAGCAAG ATACAAATGA TCATATATCT GATATAGACG TGGGCGTTCG   
  
  
+ GATACACGTT CGGGTTTGTA TCAGATATTT CAGTATAAAG GTATAGAACC CGTTCGGGTA TTTCTACACT   
  
  
+ CCGAGTCGGG TTCGGGTTCG GATATTTTGG ATCGGGTTCG AATATTTAAA TTTTGAAGAA AAAAAGAAAT   
  
  
+ TATTCACTGT TTAAGTTTTT TATATTTAAA TATATCTTAA CTTAACTGAT TTTTTTTAGT TTTTAAAAGA   
  
  
+ TTAAAATATT AATATGTTTG GAGATAAAAC TTTAAAAATA GAAAGACACT AATTTAGTTT TTGTTTTGAA   
  
  
+ AATTTAGATG CAACTTTTGT TAATGCAAGA AACAAGAACT TGATATGTAT TTTAAGTGAG TAACAAATGA   
  
  
+ TTTTGTCTAT AGTTATATGT ATATTATCTA ATTTTGAGTA ATAAGAATCA TTAATATAAA TATTTTGAAT   
  
  
+ AAAATTAGAT AGATAAACTA TAAATATAGA GTTAAGTATA CTTATGTTTG GTTATCTTCG AATATTACCC   
  
  
+ GTTCGGATAT ATTATCTGAA CTGGTGAAAT AAGTAATATG TTTTGTTGTT TTAATTAGAT AATTTTTAGA   
  
  
+ CCGAGCTTGT GAATATATAC TAGACAAACA TTTATATTTC GAGTCTGCAC TTATATTCTA TAAGAGCTTG   
  
  
+ ATATATTAGA TTTGAACACT AACCTGTTAA TATAGTTTGC CGGTGATTTT TTTTCAAAAT TTTGATTCTT   
  
  
+ AGATATGTAT ATGGAGTAAA ACTAATTTTT ACAGATGCCC ATTTTTTTAA TTGACACTTA TGTAATTAAC   
  
  
+ TGAATTCATA AAACAAGGTT TTTTAAAAAA ATTTAACTCA TATCAATGAA ACAAAGACGA GAACGAAAGC   
  
  
+ ACAATTCTAT GGAAATGGAA AATGAAGTCA CTTATGGAGA TTCAATAGTA AGCAAATCGA GAGCAGAAAA   
  
  
+ TCTAATCTCC TTTCGTCATT ATACAATCAA TATTGCCTAT TTGGTTTTAG TGATTTGTTT CAGCCGCAAA   
  
  
+ ACTTAATTTT CTTTGGTGCA TATGAAATCT TAAAAAGAAT TAAAATAAGA TATAATACGT TAACTCTTCA   
  
  
+ ACAACATGAT ATATTTAAGA TACCAATATT TGTATTCATC ATATAAAAAT TGTAGTGTTG CAAAATATTA   
  
  
+ AAATTATTTC ATAAATAAAC ATTATTATAA GAACTGACTC CGCGGATTAT CATATGGTAT AGATTACAGA   
  
  
+ GTGGGTGGGT TTAAATAATT TCCCCGACAC ATTATACTTT TAGAAGAGCT ATTAGCTCCA AATTATTTAA   
  
  
+ ACAATGTTCT AAAGAGCAAA AAAATCAATG TTTTGGATTT TGATCCGACC GAGAGGACTT GTCCGACCAT   
  
  
+ TCCATATTAA AATGTTCCTA GTTCTGATTA GCTAATACCA   

- TATTTCAACA GCACTAAAGA ACGGGAAATA AAAAATAAAT ATAATTGTAT ATAAAAATTT TTTTTTAATT   
  
  
- TCTGCAAGGA TTGGCTTTGA GTACATAAAA AACTAATACT TATACTTATA GGGGAGATAT AATTAGTACC   
  
  
- TCGTAATGTT GTACAAAAGC ATCGGTATAC AGTAGTGCTC TTACTAAAAA TCTTAACAAT CTTTTTATTT   
  
  
- AACTAAGTAT ATTTGTATAT GATACAAAAA ATAATTTGAT TGATAGTTTA ATTAATTATC ACATGTTTTT   
  
  
- TTATAAAAAA AGAAAGGAAT TTATTTTTGA TGCCTTAATG GATTATACCG ATTGTATATA TACTGTTAAT   
  
  
- TACTAATACT TATTATGTAT AAACTATTTT TTTAAAGATT GGAGAGAGAA AAAACAAATT AAAATATAAT   
  
  
- AATTTCCTTT AAATTGTTAG TGTAATTAGT ATATTATTTT TGTTAATCTA AAAAAGAATA TACAATATAA   
  
  
- AACTTAAAAA TTTTTGCTGA TATTTAATGA TTTTTACTAT TCTCAGGGTG TAATTTTTTA AACACTAGTT   
  
  
- GGCAAATTGA AAAAAAAATC AAGTTCGTTC TATGTTTACT AGTATATAGA CTATATCTGC ACCCGCAAGC   
  
  
- CTATGTGCAA GCCCAAACAT AGTCTATAAA GTCATATTTC CATATCTTGG GCAAGCCCAT AAAGATGTGA   
  
  
- GGCTCAGCCC AAGCCCAAGC CTATAAAACC TAGCCCAAGC TTATAAATTT AAAACTTCTT TTTTTCTTTA   
  
  
- ATAAGTGACA AATTCAAAAA ATATAAATTT ATATAGAATT GAATTGACTA AAAAAAATCA AAAATTTTCT   
  
  
- AATTTTATAA TTATACAAAC CTCTATTTTG AAATTTTTAT CTTTCTGTGA TTAAATCAAA AACAAAACTT   
  
  
- TTAAATCTAC GTTGAAAACA ATTACGTTCT TTGTTCTTGA ACTATACATA AAATTCACTC ATTGTTTACT   
  
  
- AAAACAGATA TCAATATACA TATAATAGAT TAAAACTCAT TATTCTTAGT AATTATATTT ATAAAACTTA   
  
  
- TTTTAATCTA TCTATTTGAT ATTTATATCT CAATTCATAT GAATACAAAC CAATAGAAGC TTATAATGGG   
  
  
- CAAGCCTATA TAATAGACTT GACCACTTTA TTCATTATAC AAAACAACAA AATTAATCTA TTAAAAATCT   
  
  
- GGCTCGAACA CTTATATATG ATCTGTTTGT AAATATAAAG CTCAGACGTG AATATAAGAT ATTCTCGAAC   
  
  
- TATATAATCT AAACTTGTGA TTGGACAATT ATATCAAACG GCCACTAAAA AAAAGTTTTA AAACTAAGAA   
  
  
- TCTATACATA TACCTCATTT TGATTAAAAA TGTCTACGGG TAAAAAAATT AACTGTGAAT ACATTAATTG   
  
  
- ACTTAAGTAT TTTGTTCCAA AAAATTTTTT TAAATTGAGT ATAGTTACTT TGTTTCTGCT CTTGCTTTCG   
  
  
- TGTTAAGATA CCTTTACCTT TTACTTCAGT GAATACCTCT AAGTTATCAT TCGTTTAGCT CTCGTCTTTT   
  
  
- AGATTAGAGG AAAGCAGTAA TATGTTAGTT ATAACGGATA AACCAAAATC ACTAAACAAA GTCGGCGTTT   
  
  
- TGAATTAAAA GAAACCACGT ATACTTTAGA ATTTTTCTTA ATTTTATTCT ATATTATGCA ATTGAGAAGT   
  
  
- TGTTGTACTA TATAAATTCT ATGGTTATAA ACATAAGTAG TATATTTTTA ACATCACAAC GTTTTATAAT   
  
  
- TTTAATAAAG TATTTATTTG TAATAATATT CTTGACTGAG GCGCCTAATA GTATACCATA TCTAATGTCT   
  
  
- CACCCACCCA AATTTATTAA AGGGGCTGTG TAATATGAAA ATCTTCTCGA TAATCGAGGT TTAATAAATT   
  
  
- TGTTACAAGA TTTCTCGTTT TTTTAGTTAC AAAACCTAAA ACTAGGCTGG CTCTCCTGAA CAGGCTGGTA   
  
  
- AGGTATAATT TTACAAGGAT CAAGACTAAT CGATTATGGT

+     ERE

| Site Name | Organism | Position | Strand | Matrix score. | sequence | function |
| --- | --- | --- | --- | --- | --- | --- |
| ERE | Nicotiana glutinos | 1631 | - | 8 | ATTTCATA |  |
| ERE | Nicotiana glutinos | 1756 | + | 8 | ATTTCATA |  |

>PlantCARE\_25378   
+ ATAAAGTTGT CGTGATTTCT TGCCCTTTAT TTTTTATTTA TATTAACATA TATTTTTAAA AAAAAATTAA   
  
  
+ AGACGTTCCT AACCGAAACT CATGTATTTT TTGATTATGA ATATGAATAT CCCCTCTATA TTAATCATGG   
  
  
+ AGCATTACAA CATGTTTTCG TAGCCATATG TCATCACGAG AATGATTTTT AGAATTGTTA GAAAAATAAA   
  
  
+ TTGATTCATA TAAACATATA CTATGTTTTT TATTAAACTA ACTATCAAAT TAATTAATAG TGTACAAAAA   
  
  
+ AATATTTTTT TCTTTCCTTA AATAAAAACT ACGGAATTAC CTAATATGGC TAACATATAT ATGACAATTA   
  
  
+ ATGATTATGA ATAATACATA TTTGATAAAA AAATTTCTAA CCTCTCTCTT TTTTGTTTAA TTTTATATTA   
  
  
+ TTAAAGGAAA TTTAACAATC ACATTAATCA TATAATAAAA ACAATTAGAT TTTTTCTTAT ATGTTATATT   
  
  
+ TTGAATTTTT AAAAACGACT ATAAATTACT AAAAATGATA AGAGTCCCAC ATTAAAAAAT TTGTGATCAA   
  
  
+ CCGTTTAACT TTTTTTTTAG TTCAAGCAAG ATACAAATGA TCATATATCT GATATAGACG TGGGCGTTCG   
  
  
+ GATACACGTT CGGGTTTGTA TCAGATATTT CAGTATAAAG GTATAGAACC CGTTCGGGTA TTTCTACACT   
  
  
+ CCGAGTCGGG TTCGGGTTCG GATATTTTGG ATCGGGTTCG AATATTTAAA TTTTGAAGAA AAAAAGAAAT   
  
  
+ TATTCACTGT TTAAGTTTTT TATATTTAAA TATATCTTAA CTTAACTGAT TTTTTTTAGT TTTTAAAAGA   
  
  
+ TTAAAATATT AATATGTTTG GAGATAAAAC TTTAAAAATA GAAAGACACT AATTTAGTTT TTGTTTTGAA   
  
  
+ AATTTAGATG CAACTTTTGT TAATGCAAGA AACAAGAACT TGATATGTAT TTTAAGTGAG TAACAAATGA   
  
  
+ TTTTGTCTAT AGTTATATGT ATATTATCTA ATTTTGAGTA ATAAGAATCA TTAATATAAA TATTTTGAAT   
  
  
+ AAAATTAGAT AGATAAACTA TAAATATAGA GTTAAGTATA CTTATGTTTG GTTATCTTCG AATATTACCC   
  
  
+ GTTCGGATAT ATTATCTGAA CTGGTGAAAT AAGTAATATG TTTTGTTGTT TTAATTAGAT AATTTTTAGA   
  
  
+ CCGAGCTTGT GAATATATAC TAGACAAACA TTTATATTTC GAGTCTGCAC TTATATTCTA TAAGAGCTTG   
  
  
+ ATATATTAGA TTTGAACACT AACCTGTTAA TATAGTTTGC CGGTGATTTT TTTTCAAAAT TTTGATTCTT   
  
  
+ AGATATGTAT ATGGAGTAAA ACTAATTTTT ACAGATGCCC ATTTTTTTAA TTGACACTTA TGTAATTAAC   
  
  
+ TGAATTCATA AAACAAGGTT TTTTAAAAAA ATTTAACTCA TATCAATGAA ACAAAGACGA GAACGAAAGC   
  
  
+ ACAATTCTAT GGAAATGGAA AATGAAGTCA CTTATGGAGA TTCAATAGTA AGCAAATCGA GAGCAGAAAA   
  
  
+ TCTAATCTCC TTTCGTCATT ATACAATCAA TATTGCCTAT TTGGTTTTAG TGATTTGTTT CAGCCGCAAA   
  
  
+ ACTTAATTTT CTTTGGTGCA TATGAAATCT TAAAAAGAAT TAAAATAAGA TATAATACGT TAACTCTTCA   
  
  
+ ACAACATGAT ATATTTAAGA TACCAATATT TGTATTCATC ATATAAAAAT TGTAGTGTTG CAAAATATTA   
  
  
+ AAATTATTTC ATAAATAAAC ATTATTATAA GAACTGACTC CGCGGATTAT CATATGGTAT AGATTACAGA   
  
  
+ GTGGGTGGGT TTAAATAATT TCCCCGACAC ATTATACTTT TAGAAGAGCT ATTAGCTCCA AATTATTTAA   
  
  
+ ACAATGTTCT AAAGAGCAAA AAAATCAATG TTTTGGATTT TGATCCGACC GAGAGGACTT GTCCGACCAT   
  
  
+ TCCATATTAA AATGTTCCTA GTTCTGATTA GCTAATACCA   

- TATTTCAACA GCACTAAAGA ACGGGAAATA AAAAATAAAT ATAATTGTAT ATAAAAATTT TTTTTTAATT   
  
  
- TCTGCAAGGA TTGGCTTTGA GTACATAAAA AACTAATACT TATACTTATA GGGGAGATAT AATTAGTACC   
  
  
- TCGTAATGTT GTACAAAAGC ATCGGTATAC AGTAGTGCTC TTACTAAAAA TCTTAACAAT CTTTTTATTT   
  
  
- AACTAAGTAT ATTTGTATAT GATACAAAAA ATAATTTGAT TGATAGTTTA ATTAATTATC ACATGTTTTT   
  
  
- TTATAAAAAA AGAAAGGAAT TTATTTTTGA TGCCTTAATG GATTATACCG ATTGTATATA TACTGTTAAT   
  
  
- TACTAATACT TATTATGTAT AAACTATTTT TTTAAAGATT GGAGAGAGAA AAAACAAATT AAAATATAAT   
  
  
- AATTTCCTTT AAATTGTTAG TGTAATTAGT ATATTATTTT TGTTAATCTA AAAAAGAATA TACAATATAA   
  
  
- AACTTAAAAA TTTTTGCTGA TATTTAATGA TTTTTACTAT TCTCAGGGTG TAATTTTTTA AACACTAGTT   
  
  
- GGCAAATTGA AAAAAAAATC AAGTTCGTTC TATGTTTACT AGTATATAGA CTATATCTGC ACCCGCAAGC   
  
  
- CTATGTGCAA GCCCAAACAT AGTCTATAAA GTCATATTTC CATATCTTGG GCAAGCCCAT AAAGATGTGA   
  
  
- GGCTCAGCCC AAGCCCAAGC CTATAAAACC TAGCCCAAGC TTATAAATTT AAAACTTCTT TTTTTCTTTA   
  
  
- ATAAGTGACA AATTCAAAAA ATATAAATTT ATATAGAATT GAATTGACTA AAAAAAATCA AAAATTTTCT   
  
  
- AATTTTATAA TTATACAAAC CTCTATTTTG AAATTTTTAT CTTTCTGTGA TTAAATCAAA AACAAAACTT   
  
  
- TTAAATCTAC GTTGAAAACA ATTACGTTCT TTGTTCTTGA ACTATACATA AAATTCACTC ATTGTTTACT   
  
  
- AAAACAGATA TCAATATACA TATAATAGAT TAAAACTCAT TATTCTTAGT AATTATATTT ATAAAACTTA   
  
  
- TTTTAATCTA TCTATTTGAT ATTTATATCT CAATTCATAT GAATACAAAC CAATAGAAGC TTATAATGGG   
  
  
- CAAGCCTATA TAATAGACTT GACCACTTTA TTCATTATAC AAAACAACAA AATTAATCTA TTAAAAATCT   
  
  
- GGCTCGAACA CTTATATATG ATCTGTTTGT AAATATAAAG CTCAGACGTG AATATAAGAT ATTCTCGAAC   
  
  
- TATATAATCT AAACTTGTGA TTGGACAATT ATATCAAACG GCCACTAAAA AAAAGTTTTA AAACTAAGAA   
  
  
- TCTATACATA TACCTCATTT TGATTAAAAA TGTCTACGGG TAAAAAAATT AACTGTGAAT ACATTAATTG   
  
  
- ACTTAAGTAT TTTGTTCCAA AAAATTTTTT TAAATTGAGT ATAGTTACTT TGTTTCTGCT CTTGCTTTCG   
  
  
- TGTTAAGATA CCTTTACCTT TTACTTCAGT GAATACCTCT AAGTTATCAT TCGTTTAGCT CTCGTCTTTT   
  
  
- AGATTAGAGG AAAGCAGTAA TATGTTAGTT ATAACGGATA AACCAAAATC ACTAAACAAA GTCGGCGTTT   
  
  
- TGAATTAAAA GAAACCACGT ATACTTTAGA ATTTTTCTTA ATTTTATTCT ATATTATGCA ATTGAGAAGT   
  
  
- TGTTGTACTA TATAAATTCT ATGGTTATAA ACATAAGTAG TATATTTTTA ACATCACAAC GTTTTATAAT   
  
  
- TTTAATAAAG TATTTATTTG TAATAATATT CTTGACTGAG GCGCCTAATA GTATACCATA TCTAATGTCT   
  
  
- CACCCACCCA AATTTATTAA AGGGGCTGTG TAATATGAAA ATCTTCTCGA TAATCGAGGT TTAATAAATT   
  
  
- TGTTACAAGA TTTCTCGTTT TTTTAGTTAC AAAACCTAAA ACTAGGCTGG CTCTCCTGAA CAGGCTGGTA   
  
  
- AGGTATAATT TTACAAGGAT CAAGACTAAT CGATTATGGT

+     G-Box

| Site Name | Organism | Position | Strand | Matrix score. | sequence | function |
| --- | --- | --- | --- | --- | --- | --- |
| G-Box | Pisum sativum | 635 | + | 6 | CACGTT | cis-acting regulatory element involved in light responsiveness |

>PlantCARE\_25378   
+ ATAAAGTTGT CGTGATTTCT TGCCCTTTAT TTTTTATTTA TATTAACATA TATTTTTAAA AAAAAATTAA   
  
  
+ AGACGTTCCT AACCGAAACT CATGTATTTT TTGATTATGA ATATGAATAT CCCCTCTATA TTAATCATGG   
  
  
+ AGCATTACAA CATGTTTTCG TAGCCATATG TCATCACGAG AATGATTTTT AGAATTGTTA GAAAAATAAA   
  
  
+ TTGATTCATA TAAACATATA CTATGTTTTT TATTAAACTA ACTATCAAAT TAATTAATAG TGTACAAAAA   
  
  
+ AATATTTTTT TCTTTCCTTA AATAAAAACT ACGGAATTAC CTAATATGGC TAACATATAT ATGACAATTA   
  
  
+ ATGATTATGA ATAATACATA TTTGATAAAA AAATTTCTAA CCTCTCTCTT TTTTGTTTAA TTTTATATTA   
  
  
+ TTAAAGGAAA TTTAACAATC ACATTAATCA TATAATAAAA ACAATTAGAT TTTTTCTTAT ATGTTATATT   
  
  
+ TTGAATTTTT AAAAACGACT ATAAATTACT AAAAATGATA AGAGTCCCAC ATTAAAAAAT TTGTGATCAA   
  
  
+ CCGTTTAACT TTTTTTTTAG TTCAAGCAAG ATACAAATGA TCATATATCT GATATAGACG TGGGCGTTCG   
  
  
+ GATACACGTT CGGGTTTGTA TCAGATATTT CAGTATAAAG GTATAGAACC CGTTCGGGTA TTTCTACACT   
  
  
+ CCGAGTCGGG TTCGGGTTCG GATATTTTGG ATCGGGTTCG AATATTTAAA TTTTGAAGAA AAAAAGAAAT   
  
  
+ TATTCACTGT TTAAGTTTTT TATATTTAAA TATATCTTAA CTTAACTGAT TTTTTTTAGT TTTTAAAAGA   
  
  
+ TTAAAATATT AATATGTTTG GAGATAAAAC TTTAAAAATA GAAAGACACT AATTTAGTTT TTGTTTTGAA   
  
  
+ AATTTAGATG CAACTTTTGT TAATGCAAGA AACAAGAACT TGATATGTAT TTTAAGTGAG TAACAAATGA   
  
  
+ TTTTGTCTAT AGTTATATGT ATATTATCTA ATTTTGAGTA ATAAGAATCA TTAATATAAA TATTTTGAAT   
  
  
+ AAAATTAGAT AGATAAACTA TAAATATAGA GTTAAGTATA CTTATGTTTG GTTATCTTCG AATATTACCC   
  
  
+ GTTCGGATAT ATTATCTGAA CTGGTGAAAT AAGTAATATG TTTTGTTGTT TTAATTAGAT AATTTTTAGA   
  
  
+ CCGAGCTTGT GAATATATAC TAGACAAACA TTTATATTTC GAGTCTGCAC TTATATTCTA TAAGAGCTTG   
  
  
+ ATATATTAGA TTTGAACACT AACCTGTTAA TATAGTTTGC CGGTGATTTT TTTTCAAAAT TTTGATTCTT   
  
  
+ AGATATGTAT ATGGAGTAAA ACTAATTTTT ACAGATGCCC ATTTTTTTAA TTGACACTTA TGTAATTAAC   
  
  
+ TGAATTCATA AAACAAGGTT TTTTAAAAAA ATTTAACTCA TATCAATGAA ACAAAGACGA GAACGAAAGC   
  
  
+ ACAATTCTAT GGAAATGGAA AATGAAGTCA CTTATGGAGA TTCAATAGTA AGCAAATCGA GAGCAGAAAA   
  
  
+ TCTAATCTCC TTTCGTCATT ATACAATCAA TATTGCCTAT TTGGTTTTAG TGATTTGTTT CAGCCGCAAA   
  
  
+ ACTTAATTTT CTTTGGTGCA TATGAAATCT TAAAAAGAAT TAAAATAAGA TATAATACGT TAACTCTTCA   
  
  
+ ACAACATGAT ATATTTAAGA TACCAATATT TGTATTCATC ATATAAAAAT TGTAGTGTTG CAAAATATTA   
  
  
+ AAATTATTTC ATAAATAAAC ATTATTATAA GAACTGACTC CGCGGATTAT CATATGGTAT AGATTACAGA   
  
  
+ GTGGGTGGGT TTAAATAATT TCCCCGACAC ATTATACTTT TAGAAGAGCT ATTAGCTCCA AATTATTTAA   
  
  
+ ACAATGTTCT AAAGAGCAAA AAAATCAATG TTTTGGATTT TGATCCGACC GAGAGGACTT GTCCGACCAT   
  
  
+ TCCATATTAA AATGTTCCTA GTTCTGATTA GCTAATACCA   

- TATTTCAACA GCACTAAAGA ACGGGAAATA AAAAATAAAT ATAATTGTAT ATAAAAATTT TTTTTTAATT   
  
  
- TCTGCAAGGA TTGGCTTTGA GTACATAAAA AACTAATACT TATACTTATA GGGGAGATAT AATTAGTACC   
  
  
- TCGTAATGTT GTACAAAAGC ATCGGTATAC AGTAGTGCTC TTACTAAAAA TCTTAACAAT CTTTTTATTT   
  
  
- AACTAAGTAT ATTTGTATAT GATACAAAAA ATAATTTGAT TGATAGTTTA ATTAATTATC ACATGTTTTT   
  
  
- TTATAAAAAA AGAAAGGAAT TTATTTTTGA TGCCTTAATG GATTATACCG ATTGTATATA TACTGTTAAT   
  
  
- TACTAATACT TATTATGTAT AAACTATTTT TTTAAAGATT GGAGAGAGAA AAAACAAATT AAAATATAAT   
  
  
- AATTTCCTTT AAATTGTTAG TGTAATTAGT ATATTATTTT TGTTAATCTA AAAAAGAATA TACAATATAA   
  
  
- AACTTAAAAA TTTTTGCTGA TATTTAATGA TTTTTACTAT TCTCAGGGTG TAATTTTTTA AACACTAGTT   
  
  
- GGCAAATTGA AAAAAAAATC AAGTTCGTTC TATGTTTACT AGTATATAGA CTATATCTGC ACCCGCAAGC   
  
  
- CTATGTGCAA GCCCAAACAT AGTCTATAAA GTCATATTTC CATATCTTGG GCAAGCCCAT AAAGATGTGA   
  
  
- GGCTCAGCCC AAGCCCAAGC CTATAAAACC TAGCCCAAGC TTATAAATTT AAAACTTCTT TTTTTCTTTA   
  
  
- ATAAGTGACA AATTCAAAAA ATATAAATTT ATATAGAATT GAATTGACTA AAAAAAATCA AAAATTTTCT   
  
  
- AATTTTATAA TTATACAAAC CTCTATTTTG AAATTTTTAT CTTTCTGTGA TTAAATCAAA AACAAAACTT   
  
  
- TTAAATCTAC GTTGAAAACA ATTACGTTCT TTGTTCTTGA ACTATACATA AAATTCACTC ATTGTTTACT   
  
  
- AAAACAGATA TCAATATACA TATAATAGAT TAAAACTCAT TATTCTTAGT AATTATATTT ATAAAACTTA   
  
  
- TTTTAATCTA TCTATTTGAT ATTTATATCT CAATTCATAT GAATACAAAC CAATAGAAGC TTATAATGGG   
  
  
- CAAGCCTATA TAATAGACTT GACCACTTTA TTCATTATAC AAAACAACAA AATTAATCTA TTAAAAATCT   
  
  
- GGCTCGAACA CTTATATATG ATCTGTTTGT AAATATAAAG CTCAGACGTG AATATAAGAT ATTCTCGAAC   
  
  
- TATATAATCT AAACTTGTGA TTGGACAATT ATATCAAACG GCCACTAAAA AAAAGTTTTA AAACTAAGAA   
  
  
- TCTATACATA TACCTCATTT TGATTAAAAA TGTCTACGGG TAAAAAAATT AACTGTGAAT ACATTAATTG   
  
  
- ACTTAAGTAT TTTGTTCCAA AAAATTTTTT TAAATTGAGT ATAGTTACTT TGTTTCTGCT CTTGCTTTCG   
  
  
- TGTTAAGATA CCTTTACCTT TTACTTCAGT GAATACCTCT AAGTTATCAT TCGTTTAGCT CTCGTCTTTT   
  
  
- AGATTAGAGG AAAGCAGTAA TATGTTAGTT ATAACGGATA AACCAAAATC ACTAAACAAA GTCGGCGTTT   
  
  
- TGAATTAAAA GAAACCACGT ATACTTTAGA ATTTTTCTTA ATTTTATTCT ATATTATGCA ATTGAGAAGT   
  
  
- TGTTGTACTA TATAAATTCT ATGGTTATAA ACATAAGTAG TATATTTTTA ACATCACAAC GTTTTATAAT   
  
  
- TTTAATAAAG TATTTATTTG TAATAATATT CTTGACTGAG GCGCCTAATA GTATACCATA TCTAATGTCT   
  
  
- CACCCACCCA AATTTATTAA AGGGGCTGTG TAATATGAAA ATCTTCTCGA TAATCGAGGT TTAATAAATT   
  
  
- TGTTACAAGA TTTCTCGTTT TTTTAGTTAC AAAACCTAAA ACTAGGCTGG CTCTCCTGAA CAGGCTGGTA   
  
  
- AGGTATAATT TTACAAGGAT CAAGACTAAT CGATTATGGT

+     G-box

| Site Name | Organism | Position | Strand | Matrix score. | sequence | function |
| --- | --- | --- | --- | --- | --- | --- |
| G-box | Zea mays | 9 | - | 6 | CACGAC | cis-acting regulatory element involved in light responsiveness |
| G-box | Zea mays | 617 | - | 6 | CACGTC | cis-acting regulatory element involved in light responsiveness |

>PlantCARE\_25378   
+ ATAAAGTTGT CGTGATTTCT TGCCCTTTAT TTTTTATTTA TATTAACATA TATTTTTAAA AAAAAATTAA   
  
  
+ AGACGTTCCT AACCGAAACT CATGTATTTT TTGATTATGA ATATGAATAT CCCCTCTATA TTAATCATGG   
  
  
+ AGCATTACAA CATGTTTTCG TAGCCATATG TCATCACGAG AATGATTTTT AGAATTGTTA GAAAAATAAA   
  
  
+ TTGATTCATA TAAACATATA CTATGTTTTT TATTAAACTA ACTATCAAAT TAATTAATAG TGTACAAAAA   
  
  
+ AATATTTTTT TCTTTCCTTA AATAAAAACT ACGGAATTAC CTAATATGGC TAACATATAT ATGACAATTA   
  
  
+ ATGATTATGA ATAATACATA TTTGATAAAA AAATTTCTAA CCTCTCTCTT TTTTGTTTAA TTTTATATTA   
  
  
+ TTAAAGGAAA TTTAACAATC ACATTAATCA TATAATAAAA ACAATTAGAT TTTTTCTTAT ATGTTATATT   
  
  
+ TTGAATTTTT AAAAACGACT ATAAATTACT AAAAATGATA AGAGTCCCAC ATTAAAAAAT TTGTGATCAA   
  
  
+ CCGTTTAACT TTTTTTTTAG TTCAAGCAAG ATACAAATGA TCATATATCT GATATAGACG TGGGCGTTCG   
  
  
+ GATACACGTT CGGGTTTGTA TCAGATATTT CAGTATAAAG GTATAGAACC CGTTCGGGTA TTTCTACACT   
  
  
+ CCGAGTCGGG TTCGGGTTCG GATATTTTGG ATCGGGTTCG AATATTTAAA TTTTGAAGAA AAAAAGAAAT   
  
  
+ TATTCACTGT TTAAGTTTTT TATATTTAAA TATATCTTAA CTTAACTGAT TTTTTTTAGT TTTTAAAAGA   
  
  
+ TTAAAATATT AATATGTTTG GAGATAAAAC TTTAAAAATA GAAAGACACT AATTTAGTTT TTGTTTTGAA   
  
  
+ AATTTAGATG CAACTTTTGT TAATGCAAGA AACAAGAACT TGATATGTAT TTTAAGTGAG TAACAAATGA   
  
  
+ TTTTGTCTAT AGTTATATGT ATATTATCTA ATTTTGAGTA ATAAGAATCA TTAATATAAA TATTTTGAAT   
  
  
+ AAAATTAGAT AGATAAACTA TAAATATAGA GTTAAGTATA CTTATGTTTG GTTATCTTCG AATATTACCC   
  
  
+ GTTCGGATAT ATTATCTGAA CTGGTGAAAT AAGTAATATG TTTTGTTGTT TTAATTAGAT AATTTTTAGA   
  
  
+ CCGAGCTTGT GAATATATAC TAGACAAACA TTTATATTTC GAGTCTGCAC TTATATTCTA TAAGAGCTTG   
  
  
+ ATATATTAGA TTTGAACACT AACCTGTTAA TATAGTTTGC CGGTGATTTT TTTTCAAAAT TTTGATTCTT   
  
  
+ AGATATGTAT ATGGAGTAAA ACTAATTTTT ACAGATGCCC ATTTTTTTAA TTGACACTTA TGTAATTAAC   
  
  
+ TGAATTCATA AAACAAGGTT TTTTAAAAAA ATTTAACTCA TATCAATGAA ACAAAGACGA GAACGAAAGC   
  
  
+ ACAATTCTAT GGAAATGGAA AATGAAGTCA CTTATGGAGA TTCAATAGTA AGCAAATCGA GAGCAGAAAA   
  
  
+ TCTAATCTCC TTTCGTCATT ATACAATCAA TATTGCCTAT TTGGTTTTAG TGATTTGTTT CAGCCGCAAA   
  
  
+ ACTTAATTTT CTTTGGTGCA TATGAAATCT TAAAAAGAAT TAAAATAAGA TATAATACGT TAACTCTTCA   
  
  
+ ACAACATGAT ATATTTAAGA TACCAATATT TGTATTCATC ATATAAAAAT TGTAGTGTTG CAAAATATTA   
  
  
+ AAATTATTTC ATAAATAAAC ATTATTATAA GAACTGACTC CGCGGATTAT CATATGGTAT AGATTACAGA   
  
  
+ GTGGGTGGGT TTAAATAATT TCCCCGACAC ATTATACTTT TAGAAGAGCT ATTAGCTCCA AATTATTTAA   
  
  
+ ACAATGTTCT AAAGAGCAAA AAAATCAATG TTTTGGATTT TGATCCGACC GAGAGGACTT GTCCGACCAT   
  
  
+ TCCATATTAA AATGTTCCTA GTTCTGATTA GCTAATACCA   

- TATTTCAACA GCACTAAAGA ACGGGAAATA AAAAATAAAT ATAATTGTAT ATAAAAATTT TTTTTTAATT   
  
  
- TCTGCAAGGA TTGGCTTTGA GTACATAAAA AACTAATACT TATACTTATA GGGGAGATAT AATTAGTACC   
  
  
- TCGTAATGTT GTACAAAAGC ATCGGTATAC AGTAGTGCTC TTACTAAAAA TCTTAACAAT CTTTTTATTT   
  
  
- AACTAAGTAT ATTTGTATAT GATACAAAAA ATAATTTGAT TGATAGTTTA ATTAATTATC ACATGTTTTT   
  
  
- TTATAAAAAA AGAAAGGAAT TTATTTTTGA TGCCTTAATG GATTATACCG ATTGTATATA TACTGTTAAT   
  
  
- TACTAATACT TATTATGTAT AAACTATTTT TTTAAAGATT GGAGAGAGAA AAAACAAATT AAAATATAAT   
  
  
- AATTTCCTTT AAATTGTTAG TGTAATTAGT ATATTATTTT TGTTAATCTA AAAAAGAATA TACAATATAA   
  
  
- AACTTAAAAA TTTTTGCTGA TATTTAATGA TTTTTACTAT TCTCAGGGTG TAATTTTTTA AACACTAGTT   
  
  
- GGCAAATTGA AAAAAAAATC AAGTTCGTTC TATGTTTACT AGTATATAGA CTATATCTGC ACCCGCAAGC   
  
  
- CTATGTGCAA GCCCAAACAT AGTCTATAAA GTCATATTTC CATATCTTGG GCAAGCCCAT AAAGATGTGA   
  
  
- GGCTCAGCCC AAGCCCAAGC CTATAAAACC TAGCCCAAGC TTATAAATTT AAAACTTCTT TTTTTCTTTA   
  
  
- ATAAGTGACA AATTCAAAAA ATATAAATTT ATATAGAATT GAATTGACTA AAAAAAATCA AAAATTTTCT   
  
  
- AATTTTATAA TTATACAAAC CTCTATTTTG AAATTTTTAT CTTTCTGTGA TTAAATCAAA AACAAAACTT   
  
  
- TTAAATCTAC GTTGAAAACA ATTACGTTCT TTGTTCTTGA ACTATACATA AAATTCACTC ATTGTTTACT   
  
  
- AAAACAGATA TCAATATACA TATAATAGAT TAAAACTCAT TATTCTTAGT AATTATATTT ATAAAACTTA   
  
  
- TTTTAATCTA TCTATTTGAT ATTTATATCT CAATTCATAT GAATACAAAC CAATAGAAGC TTATAATGGG   
  
  
- CAAGCCTATA TAATAGACTT GACCACTTTA TTCATTATAC AAAACAACAA AATTAATCTA TTAAAAATCT   
  
  
- GGCTCGAACA CTTATATATG ATCTGTTTGT AAATATAAAG CTCAGACGTG AATATAAGAT ATTCTCGAAC   
  
  
- TATATAATCT AAACTTGTGA TTGGACAATT ATATCAAACG GCCACTAAAA AAAAGTTTTA AAACTAAGAA   
  
  
- TCTATACATA TACCTCATTT TGATTAAAAA TGTCTACGGG TAAAAAAATT AACTGTGAAT ACATTAATTG   
  
  
- ACTTAAGTAT TTTGTTCCAA AAAATTTTTT TAAATTGAGT ATAGTTACTT TGTTTCTGCT CTTGCTTTCG   
  
  
- TGTTAAGATA CCTTTACCTT TTACTTCAGT GAATACCTCT AAGTTATCAT TCGTTTAGCT CTCGTCTTTT   
  
  
- AGATTAGAGG AAAGCAGTAA TATGTTAGTT ATAACGGATA AACCAAAATC ACTAAACAAA GTCGGCGTTT   
  
  
- TGAATTAAAA GAAACCACGT ATACTTTAGA ATTTTTCTTA ATTTTATTCT ATATTATGCA ATTGAGAAGT   
  
  
- TGTTGTACTA TATAAATTCT ATGGTTATAA ACATAAGTAG TATATTTTTA ACATCACAAC GTTTTATAAT   
  
  
- TTTAATAAAG TATTTATTTG TAATAATATT CTTGACTGAG GCGCCTAATA GTATACCATA TCTAATGTCT   
  
  
- CACCCACCCA AATTTATTAA AGGGGCTGTG TAATATGAAA ATCTTCTCGA TAATCGAGGT TTAATAAATT   
  
  
- TGTTACAAGA TTTCTCGTTT TTTTAGTTAC AAAACCTAAA ACTAGGCTGG CTCTCCTGAA CAGGCTGGTA   
  
  
- AGGTATAATT TTACAAGGAT CAAGACTAAT CGATTATGGT

+     GA-motif

| Site Name | Organism | Position | Strand | Matrix score. | sequence | function |
| --- | --- | --- | --- | --- | --- | --- |
| GA-motif | Arabidopsis thaliana | 1059 | + | 8 | ATAGATAA | part of a light responsive element |

>PlantCARE\_25378   
+ ATAAAGTTGT CGTGATTTCT TGCCCTTTAT TTTTTATTTA TATTAACATA TATTTTTAAA AAAAAATTAA   
  
  
+ AGACGTTCCT AACCGAAACT CATGTATTTT TTGATTATGA ATATGAATAT CCCCTCTATA TTAATCATGG   
  
  
+ AGCATTACAA CATGTTTTCG TAGCCATATG TCATCACGAG AATGATTTTT AGAATTGTTA GAAAAATAAA   
  
  
+ TTGATTCATA TAAACATATA CTATGTTTTT TATTAAACTA ACTATCAAAT TAATTAATAG TGTACAAAAA   
  
  
+ AATATTTTTT TCTTTCCTTA AATAAAAACT ACGGAATTAC CTAATATGGC TAACATATAT ATGACAATTA   
  
  
+ ATGATTATGA ATAATACATA TTTGATAAAA AAATTTCTAA CCTCTCTCTT TTTTGTTTAA TTTTATATTA   
  
  
+ TTAAAGGAAA TTTAACAATC ACATTAATCA TATAATAAAA ACAATTAGAT TTTTTCTTAT ATGTTATATT   
  
  
+ TTGAATTTTT AAAAACGACT ATAAATTACT AAAAATGATA AGAGTCCCAC ATTAAAAAAT TTGTGATCAA   
  
  
+ CCGTTTAACT TTTTTTTTAG TTCAAGCAAG ATACAAATGA TCATATATCT GATATAGACG TGGGCGTTCG   
  
  
+ GATACACGTT CGGGTTTGTA TCAGATATTT CAGTATAAAG GTATAGAACC CGTTCGGGTA TTTCTACACT   
  
  
+ CCGAGTCGGG TTCGGGTTCG GATATTTTGG ATCGGGTTCG AATATTTAAA TTTTGAAGAA AAAAAGAAAT   
  
  
+ TATTCACTGT TTAAGTTTTT TATATTTAAA TATATCTTAA CTTAACTGAT TTTTTTTAGT TTTTAAAAGA   
  
  
+ TTAAAATATT AATATGTTTG GAGATAAAAC TTTAAAAATA GAAAGACACT AATTTAGTTT TTGTTTTGAA   
  
  
+ AATTTAGATG CAACTTTTGT TAATGCAAGA AACAAGAACT TGATATGTAT TTTAAGTGAG TAACAAATGA   
  
  
+ TTTTGTCTAT AGTTATATGT ATATTATCTA ATTTTGAGTA ATAAGAATCA TTAATATAAA TATTTTGAAT   
  
  
+ AAAATTAGAT AGATAAACTA TAAATATAGA GTTAAGTATA CTTATGTTTG GTTATCTTCG AATATTACCC   
  
  
+ GTTCGGATAT ATTATCTGAA CTGGTGAAAT AAGTAATATG TTTTGTTGTT TTAATTAGAT AATTTTTAGA   
  
  
+ CCGAGCTTGT GAATATATAC TAGACAAACA TTTATATTTC GAGTCTGCAC TTATATTCTA TAAGAGCTTG   
  
  
+ ATATATTAGA TTTGAACACT AACCTGTTAA TATAGTTTGC CGGTGATTTT TTTTCAAAAT TTTGATTCTT   
  
  
+ AGATATGTAT ATGGAGTAAA ACTAATTTTT ACAGATGCCC ATTTTTTTAA TTGACACTTA TGTAATTAAC   
  
  
+ TGAATTCATA AAACAAGGTT TTTTAAAAAA ATTTAACTCA TATCAATGAA ACAAAGACGA GAACGAAAGC   
  
  
+ ACAATTCTAT GGAAATGGAA AATGAAGTCA CTTATGGAGA TTCAATAGTA AGCAAATCGA GAGCAGAAAA   
  
  
+ TCTAATCTCC TTTCGTCATT ATACAATCAA TATTGCCTAT TTGGTTTTAG TGATTTGTTT CAGCCGCAAA   
  
  
+ ACTTAATTTT CTTTGGTGCA TATGAAATCT TAAAAAGAAT TAAAATAAGA TATAATACGT TAACTCTTCA   
  
  
+ ACAACATGAT ATATTTAAGA TACCAATATT TGTATTCATC ATATAAAAAT TGTAGTGTTG CAAAATATTA   
  
  
+ AAATTATTTC ATAAATAAAC ATTATTATAA GAACTGACTC CGCGGATTAT CATATGGTAT AGATTACAGA   
  
  
+ GTGGGTGGGT TTAAATAATT TCCCCGACAC ATTATACTTT TAGAAGAGCT ATTAGCTCCA AATTATTTAA   
  
  
+ ACAATGTTCT AAAGAGCAAA AAAATCAATG TTTTGGATTT TGATCCGACC GAGAGGACTT GTCCGACCAT   
  
  
+ TCCATATTAA AATGTTCCTA GTTCTGATTA GCTAATACCA   

- TATTTCAACA GCACTAAAGA ACGGGAAATA AAAAATAAAT ATAATTGTAT ATAAAAATTT TTTTTTAATT   
  
  
- TCTGCAAGGA TTGGCTTTGA GTACATAAAA AACTAATACT TATACTTATA GGGGAGATAT AATTAGTACC   
  
  
- TCGTAATGTT GTACAAAAGC ATCGGTATAC AGTAGTGCTC TTACTAAAAA TCTTAACAAT CTTTTTATTT   
  
  
- AACTAAGTAT ATTTGTATAT GATACAAAAA ATAATTTGAT TGATAGTTTA ATTAATTATC ACATGTTTTT   
  
  
- TTATAAAAAA AGAAAGGAAT TTATTTTTGA TGCCTTAATG GATTATACCG ATTGTATATA TACTGTTAAT   
  
  
- TACTAATACT TATTATGTAT AAACTATTTT TTTAAAGATT GGAGAGAGAA AAAACAAATT AAAATATAAT   
  
  
- AATTTCCTTT AAATTGTTAG TGTAATTAGT ATATTATTTT TGTTAATCTA AAAAAGAATA TACAATATAA   
  
  
- AACTTAAAAA TTTTTGCTGA TATTTAATGA TTTTTACTAT TCTCAGGGTG TAATTTTTTA AACACTAGTT   
  
  
- GGCAAATTGA AAAAAAAATC AAGTTCGTTC TATGTTTACT AGTATATAGA CTATATCTGC ACCCGCAAGC   
  
  
- CTATGTGCAA GCCCAAACAT AGTCTATAAA GTCATATTTC CATATCTTGG GCAAGCCCAT AAAGATGTGA   
  
  
- GGCTCAGCCC AAGCCCAAGC CTATAAAACC TAGCCCAAGC TTATAAATTT AAAACTTCTT TTTTTCTTTA   
  
  
- ATAAGTGACA AATTCAAAAA ATATAAATTT ATATAGAATT GAATTGACTA AAAAAAATCA AAAATTTTCT   
  
  
- AATTTTATAA TTATACAAAC CTCTATTTTG AAATTTTTAT CTTTCTGTGA TTAAATCAAA AACAAAACTT   
  
  
- TTAAATCTAC GTTGAAAACA ATTACGTTCT TTGTTCTTGA ACTATACATA AAATTCACTC ATTGTTTACT   
  
  
- AAAACAGATA TCAATATACA TATAATAGAT TAAAACTCAT TATTCTTAGT AATTATATTT ATAAAACTTA   
  
  
- TTTTAATCTA TCTATTTGAT ATTTATATCT CAATTCATAT GAATACAAAC CAATAGAAGC TTATAATGGG   
  
  
- CAAGCCTATA TAATAGACTT GACCACTTTA TTCATTATAC AAAACAACAA AATTAATCTA TTAAAAATCT   
  
  
- GGCTCGAACA CTTATATATG ATCTGTTTGT AAATATAAAG CTCAGACGTG AATATAAGAT ATTCTCGAAC   
  
  
- TATATAATCT AAACTTGTGA TTGGACAATT ATATCAAACG GCCACTAAAA AAAAGTTTTA AAACTAAGAA   
  
  
- TCTATACATA TACCTCATTT TGATTAAAAA TGTCTACGGG TAAAAAAATT AACTGTGAAT ACATTAATTG   
  
  
- ACTTAAGTAT TTTGTTCCAA AAAATTTTTT TAAATTGAGT ATAGTTACTT TGTTTCTGCT CTTGCTTTCG   
  
  
- TGTTAAGATA CCTTTACCTT TTACTTCAGT GAATACCTCT AAGTTATCAT TCGTTTAGCT CTCGTCTTTT   
  
  
- AGATTAGAGG AAAGCAGTAA TATGTTAGTT ATAACGGATA AACCAAAATC ACTAAACAAA GTCGGCGTTT   
  
  
- TGAATTAAAA GAAACCACGT ATACTTTAGA ATTTTTCTTA ATTTTATTCT ATATTATGCA ATTGAGAAGT   
  
  
- TGTTGTACTA TATAAATTCT ATGGTTATAA ACATAAGTAG TATATTTTTA ACATCACAAC GTTTTATAAT   
  
  
- TTTAATAAAG TATTTATTTG TAATAATATT CTTGACTGAG GCGCCTAATA GTATACCATA TCTAATGTCT   
  
  
- CACCCACCCA AATTTATTAA AGGGGCTGTG TAATATGAAA ATCTTCTCGA TAATCGAGGT TTAATAAATT   
  
  
- TGTTACAAGA TTTCTCGTTT TTTTAGTTAC AAAACCTAAA ACTAGGCTGG CTCTCCTGAA CAGGCTGGTA   
  
  
- AGGTATAATT TTACAAGGAT CAAGACTAAT CGATTATGGT

+     LTR

| Site Name | Organism | Position | Strand | Matrix score. | sequence | function |
| --- | --- | --- | --- | --- | --- | --- |
| LTR | Hordeum vulgare | 83 | + | 6 | CCGAAA | cis-acting element involved in low-temperature responsiveness |

>PlantCARE\_25378   
+ ATAAAGTTGT CGTGATTTCT TGCCCTTTAT TTTTTATTTA TATTAACATA TATTTTTAAA AAAAAATTAA   
  
  
+ AGACGTTCCT AACCGAAACT CATGTATTTT TTGATTATGA ATATGAATAT CCCCTCTATA TTAATCATGG   
  
  
+ AGCATTACAA CATGTTTTCG TAGCCATATG TCATCACGAG AATGATTTTT AGAATTGTTA GAAAAATAAA   
  
  
+ TTGATTCATA TAAACATATA CTATGTTTTT TATTAAACTA ACTATCAAAT TAATTAATAG TGTACAAAAA   
  
  
+ AATATTTTTT TCTTTCCTTA AATAAAAACT ACGGAATTAC CTAATATGGC TAACATATAT ATGACAATTA   
  
  
+ ATGATTATGA ATAATACATA TTTGATAAAA AAATTTCTAA CCTCTCTCTT TTTTGTTTAA TTTTATATTA   
  
  
+ TTAAAGGAAA TTTAACAATC ACATTAATCA TATAATAAAA ACAATTAGAT TTTTTCTTAT ATGTTATATT   
  
  
+ TTGAATTTTT AAAAACGACT ATAAATTACT AAAAATGATA AGAGTCCCAC ATTAAAAAAT TTGTGATCAA   
  
  
+ CCGTTTAACT TTTTTTTTAG TTCAAGCAAG ATACAAATGA TCATATATCT GATATAGACG TGGGCGTTCG   
  
  
+ GATACACGTT CGGGTTTGTA TCAGATATTT CAGTATAAAG GTATAGAACC CGTTCGGGTA TTTCTACACT   
  
  
+ CCGAGTCGGG TTCGGGTTCG GATATTTTGG ATCGGGTTCG AATATTTAAA TTTTGAAGAA AAAAAGAAAT   
  
  
+ TATTCACTGT TTAAGTTTTT TATATTTAAA TATATCTTAA CTTAACTGAT TTTTTTTAGT TTTTAAAAGA   
  
  
+ TTAAAATATT AATATGTTTG GAGATAAAAC TTTAAAAATA GAAAGACACT AATTTAGTTT TTGTTTTGAA   
  
  
+ AATTTAGATG CAACTTTTGT TAATGCAAGA AACAAGAACT TGATATGTAT TTTAAGTGAG TAACAAATGA   
  
  
+ TTTTGTCTAT AGTTATATGT ATATTATCTA ATTTTGAGTA ATAAGAATCA TTAATATAAA TATTTTGAAT   
  
  
+ AAAATTAGAT AGATAAACTA TAAATATAGA GTTAAGTATA CTTATGTTTG GTTATCTTCG AATATTACCC   
  
  
+ GTTCGGATAT ATTATCTGAA CTGGTGAAAT AAGTAATATG TTTTGTTGTT TTAATTAGAT AATTTTTAGA   
  
  
+ CCGAGCTTGT GAATATATAC TAGACAAACA TTTATATTTC GAGTCTGCAC TTATATTCTA TAAGAGCTTG   
  
  
+ ATATATTAGA TTTGAACACT AACCTGTTAA TATAGTTTGC CGGTGATTTT TTTTCAAAAT TTTGATTCTT   
  
  
+ AGATATGTAT ATGGAGTAAA ACTAATTTTT ACAGATGCCC ATTTTTTTAA TTGACACTTA TGTAATTAAC   
  
  
+ TGAATTCATA AAACAAGGTT TTTTAAAAAA ATTTAACTCA TATCAATGAA ACAAAGACGA GAACGAAAGC   
  
  
+ ACAATTCTAT GGAAATGGAA AATGAAGTCA CTTATGGAGA TTCAATAGTA AGCAAATCGA GAGCAGAAAA   
  
  
+ TCTAATCTCC TTTCGTCATT ATACAATCAA TATTGCCTAT TTGGTTTTAG TGATTTGTTT CAGCCGCAAA   
  
  
+ ACTTAATTTT CTTTGGTGCA TATGAAATCT TAAAAAGAAT TAAAATAAGA TATAATACGT TAACTCTTCA   
  
  
+ ACAACATGAT ATATTTAAGA TACCAATATT TGTATTCATC ATATAAAAAT TGTAGTGTTG CAAAATATTA   
  
  
+ AAATTATTTC ATAAATAAAC ATTATTATAA GAACTGACTC CGCGGATTAT CATATGGTAT AGATTACAGA   
  
  
+ GTGGGTGGGT TTAAATAATT TCCCCGACAC ATTATACTTT TAGAAGAGCT ATTAGCTCCA AATTATTTAA   
  
  
+ ACAATGTTCT AAAGAGCAAA AAAATCAATG TTTTGGATTT TGATCCGACC GAGAGGACTT GTCCGACCAT   
  
  
+ TCCATATTAA AATGTTCCTA GTTCTGATTA GCTAATACCA   

- TATTTCAACA GCACTAAAGA ACGGGAAATA AAAAATAAAT ATAATTGTAT ATAAAAATTT TTTTTTAATT   
  
  
- TCTGCAAGGA TTGGCTTTGA GTACATAAAA AACTAATACT TATACTTATA GGGGAGATAT AATTAGTACC   
  
  
- TCGTAATGTT GTACAAAAGC ATCGGTATAC AGTAGTGCTC TTACTAAAAA TCTTAACAAT CTTTTTATTT   
  
  
- AACTAAGTAT ATTTGTATAT GATACAAAAA ATAATTTGAT TGATAGTTTA ATTAATTATC ACATGTTTTT   
  
  
- TTATAAAAAA AGAAAGGAAT TTATTTTTGA TGCCTTAATG GATTATACCG ATTGTATATA TACTGTTAAT   
  
  
- TACTAATACT TATTATGTAT AAACTATTTT TTTAAAGATT GGAGAGAGAA AAAACAAATT AAAATATAAT   
  
  
- AATTTCCTTT AAATTGTTAG TGTAATTAGT ATATTATTTT TGTTAATCTA AAAAAGAATA TACAATATAA   
  
  
- AACTTAAAAA TTTTTGCTGA TATTTAATGA TTTTTACTAT TCTCAGGGTG TAATTTTTTA AACACTAGTT   
  
  
- GGCAAATTGA AAAAAAAATC AAGTTCGTTC TATGTTTACT AGTATATAGA CTATATCTGC ACCCGCAAGC   
  
  
- CTATGTGCAA GCCCAAACAT AGTCTATAAA GTCATATTTC CATATCTTGG GCAAGCCCAT AAAGATGTGA   
  
  
- GGCTCAGCCC AAGCCCAAGC CTATAAAACC TAGCCCAAGC TTATAAATTT AAAACTTCTT TTTTTCTTTA   
  
  
- ATAAGTGACA AATTCAAAAA ATATAAATTT ATATAGAATT GAATTGACTA AAAAAAATCA AAAATTTTCT   
  
  
- AATTTTATAA TTATACAAAC CTCTATTTTG AAATTTTTAT CTTTCTGTGA TTAAATCAAA AACAAAACTT   
  
  
- TTAAATCTAC GTTGAAAACA ATTACGTTCT TTGTTCTTGA ACTATACATA AAATTCACTC ATTGTTTACT   
  
  
- AAAACAGATA TCAATATACA TATAATAGAT TAAAACTCAT TATTCTTAGT AATTATATTT ATAAAACTTA   
  
  
- TTTTAATCTA TCTATTTGAT ATTTATATCT CAATTCATAT GAATACAAAC CAATAGAAGC TTATAATGGG   
  
  
- CAAGCCTATA TAATAGACTT GACCACTTTA TTCATTATAC AAAACAACAA AATTAATCTA TTAAAAATCT   
  
  
- GGCTCGAACA CTTATATATG ATCTGTTTGT AAATATAAAG CTCAGACGTG AATATAAGAT ATTCTCGAAC   
  
  
- TATATAATCT AAACTTGTGA TTGGACAATT ATATCAAACG GCCACTAAAA AAAAGTTTTA AAACTAAGAA   
  
  
- TCTATACATA TACCTCATTT TGATTAAAAA TGTCTACGGG TAAAAAAATT AACTGTGAAT ACATTAATTG   
  
  
- ACTTAAGTAT TTTGTTCCAA AAAATTTTTT TAAATTGAGT ATAGTTACTT TGTTTCTGCT CTTGCTTTCG   
  
  
- TGTTAAGATA CCTTTACCTT TTACTTCAGT GAATACCTCT AAGTTATCAT TCGTTTAGCT CTCGTCTTTT   
  
  
- AGATTAGAGG AAAGCAGTAA TATGTTAGTT ATAACGGATA AACCAAAATC ACTAAACAAA GTCGGCGTTT   
  
  
- TGAATTAAAA GAAACCACGT ATACTTTAGA ATTTTTCTTA ATTTTATTCT ATATTATGCA ATTGAGAAGT   
  
  
- TGTTGTACTA TATAAATTCT ATGGTTATAA ACATAAGTAG TATATTTTTA ACATCACAAC GTTTTATAAT   
  
  
- TTTAATAAAG TATTTATTTG TAATAATATT CTTGACTGAG GCGCCTAATA GTATACCATA TCTAATGTCT   
  
  
- CACCCACCCA AATTTATTAA AGGGGCTGTG TAATATGAAA ATCTTCTCGA TAATCGAGGT TTAATAAATT   
  
  
- TGTTACAAGA TTTCTCGTTT TTTTAGTTAC AAAACCTAAA ACTAGGCTGG CTCTCCTGAA CAGGCTGGTA   
  
  
- AGGTATAATT TTACAAGGAT CAAGACTAAT CGATTATGGT

+     MYB

| Site Name | Organism | Position | Strand | Matrix score. | sequence | function |
| --- | --- | --- | --- | --- | --- | --- |
| MYB | Arabidopsis thaliana | 1099 | - | 6 | TAACCA |  |

>PlantCARE\_25378   
+ ATAAAGTTGT CGTGATTTCT TGCCCTTTAT TTTTTATTTA TATTAACATA TATTTTTAAA AAAAAATTAA   
  
  
+ AGACGTTCCT AACCGAAACT CATGTATTTT TTGATTATGA ATATGAATAT CCCCTCTATA TTAATCATGG   
  
  
+ AGCATTACAA CATGTTTTCG TAGCCATATG TCATCACGAG AATGATTTTT AGAATTGTTA GAAAAATAAA   
  
  
+ TTGATTCATA TAAACATATA CTATGTTTTT TATTAAACTA ACTATCAAAT TAATTAATAG TGTACAAAAA   
  
  
+ AATATTTTTT TCTTTCCTTA AATAAAAACT ACGGAATTAC CTAATATGGC TAACATATAT ATGACAATTA   
  
  
+ ATGATTATGA ATAATACATA TTTGATAAAA AAATTTCTAA CCTCTCTCTT TTTTGTTTAA TTTTATATTA   
  
  
+ TTAAAGGAAA TTTAACAATC ACATTAATCA TATAATAAAA ACAATTAGAT TTTTTCTTAT ATGTTATATT   
  
  
+ TTGAATTTTT AAAAACGACT ATAAATTACT AAAAATGATA AGAGTCCCAC ATTAAAAAAT TTGTGATCAA   
  
  
+ CCGTTTAACT TTTTTTTTAG TTCAAGCAAG ATACAAATGA TCATATATCT GATATAGACG TGGGCGTTCG   
  
  
+ GATACACGTT CGGGTTTGTA TCAGATATTT CAGTATAAAG GTATAGAACC CGTTCGGGTA TTTCTACACT   
  
  
+ CCGAGTCGGG TTCGGGTTCG GATATTTTGG ATCGGGTTCG AATATTTAAA TTTTGAAGAA AAAAAGAAAT   
  
  
+ TATTCACTGT TTAAGTTTTT TATATTTAAA TATATCTTAA CTTAACTGAT TTTTTTTAGT TTTTAAAAGA   
  
  
+ TTAAAATATT AATATGTTTG GAGATAAAAC TTTAAAAATA GAAAGACACT AATTTAGTTT TTGTTTTGAA   
  
  
+ AATTTAGATG CAACTTTTGT TAATGCAAGA AACAAGAACT TGATATGTAT TTTAAGTGAG TAACAAATGA   
  
  
+ TTTTGTCTAT AGTTATATGT ATATTATCTA ATTTTGAGTA ATAAGAATCA TTAATATAAA TATTTTGAAT   
  
  
+ AAAATTAGAT AGATAAACTA TAAATATAGA GTTAAGTATA CTTATGTTTG GTTATCTTCG AATATTACCC   
  
  
+ GTTCGGATAT ATTATCTGAA CTGGTGAAAT AAGTAATATG TTTTGTTGTT TTAATTAGAT AATTTTTAGA   
  
  
+ CCGAGCTTGT GAATATATAC TAGACAAACA TTTATATTTC GAGTCTGCAC TTATATTCTA TAAGAGCTTG   
  
  
+ ATATATTAGA TTTGAACACT AACCTGTTAA TATAGTTTGC CGGTGATTTT TTTTCAAAAT TTTGATTCTT   
  
  
+ AGATATGTAT ATGGAGTAAA ACTAATTTTT ACAGATGCCC ATTTTTTTAA TTGACACTTA TGTAATTAAC   
  
  
+ TGAATTCATA AAACAAGGTT TTTTAAAAAA ATTTAACTCA TATCAATGAA ACAAAGACGA GAACGAAAGC   
  
  
+ ACAATTCTAT GGAAATGGAA AATGAAGTCA CTTATGGAGA TTCAATAGTA AGCAAATCGA GAGCAGAAAA   
  
  
+ TCTAATCTCC TTTCGTCATT ATACAATCAA TATTGCCTAT TTGGTTTTAG TGATTTGTTT CAGCCGCAAA   
  
  
+ ACTTAATTTT CTTTGGTGCA TATGAAATCT TAAAAAGAAT TAAAATAAGA TATAATACGT TAACTCTTCA   
  
  
+ ACAACATGAT ATATTTAAGA TACCAATATT TGTATTCATC ATATAAAAAT TGTAGTGTTG CAAAATATTA   
  
  
+ AAATTATTTC ATAAATAAAC ATTATTATAA GAACTGACTC CGCGGATTAT CATATGGTAT AGATTACAGA   
  
  
+ GTGGGTGGGT TTAAATAATT TCCCCGACAC ATTATACTTT TAGAAGAGCT ATTAGCTCCA AATTATTTAA   
  
  
+ ACAATGTTCT AAAGAGCAAA AAAATCAATG TTTTGGATTT TGATCCGACC GAGAGGACTT GTCCGACCAT   
  
  
+ TCCATATTAA AATGTTCCTA GTTCTGATTA GCTAATACCA   

- TATTTCAACA GCACTAAAGA ACGGGAAATA AAAAATAAAT ATAATTGTAT ATAAAAATTT TTTTTTAATT   
  
  
- TCTGCAAGGA TTGGCTTTGA GTACATAAAA AACTAATACT TATACTTATA GGGGAGATAT AATTAGTACC   
  
  
- TCGTAATGTT GTACAAAAGC ATCGGTATAC AGTAGTGCTC TTACTAAAAA TCTTAACAAT CTTTTTATTT   
  
  
- AACTAAGTAT ATTTGTATAT GATACAAAAA ATAATTTGAT TGATAGTTTA ATTAATTATC ACATGTTTTT   
  
  
- TTATAAAAAA AGAAAGGAAT TTATTTTTGA TGCCTTAATG GATTATACCG ATTGTATATA TACTGTTAAT   
  
  
- TACTAATACT TATTATGTAT AAACTATTTT TTTAAAGATT GGAGAGAGAA AAAACAAATT AAAATATAAT   
  
  
- AATTTCCTTT AAATTGTTAG TGTAATTAGT ATATTATTTT TGTTAATCTA AAAAAGAATA TACAATATAA   
  
  
- AACTTAAAAA TTTTTGCTGA TATTTAATGA TTTTTACTAT TCTCAGGGTG TAATTTTTTA AACACTAGTT   
  
  
- GGCAAATTGA AAAAAAAATC AAGTTCGTTC TATGTTTACT AGTATATAGA CTATATCTGC ACCCGCAAGC   
  
  
- CTATGTGCAA GCCCAAACAT AGTCTATAAA GTCATATTTC CATATCTTGG GCAAGCCCAT AAAGATGTGA   
  
  
- GGCTCAGCCC AAGCCCAAGC CTATAAAACC TAGCCCAAGC TTATAAATTT AAAACTTCTT TTTTTCTTTA   
  
  
- ATAAGTGACA AATTCAAAAA ATATAAATTT ATATAGAATT GAATTGACTA AAAAAAATCA AAAATTTTCT   
  
  
- AATTTTATAA TTATACAAAC CTCTATTTTG AAATTTTTAT CTTTCTGTGA TTAAATCAAA AACAAAACTT   
  
  
- TTAAATCTAC GTTGAAAACA ATTACGTTCT TTGTTCTTGA ACTATACATA AAATTCACTC ATTGTTTACT   
  
  
- AAAACAGATA TCAATATACA TATAATAGAT TAAAACTCAT TATTCTTAGT AATTATATTT ATAAAACTTA   
  
  
- TTTTAATCTA TCTATTTGAT ATTTATATCT CAATTCATAT GAATACAAAC CAATAGAAGC TTATAATGGG   
  
  
- CAAGCCTATA TAATAGACTT GACCACTTTA TTCATTATAC AAAACAACAA AATTAATCTA TTAAAAATCT   
  
  
- GGCTCGAACA CTTATATATG ATCTGTTTGT AAATATAAAG CTCAGACGTG AATATAAGAT ATTCTCGAAC   
  
  
- TATATAATCT AAACTTGTGA TTGGACAATT ATATCAAACG GCCACTAAAA AAAAGTTTTA AAACTAAGAA   
  
  
- TCTATACATA TACCTCATTT TGATTAAAAA TGTCTACGGG TAAAAAAATT AACTGTGAAT ACATTAATTG   
  
  
- ACTTAAGTAT TTTGTTCCAA AAAATTTTTT TAAATTGAGT ATAGTTACTT TGTTTCTGCT CTTGCTTTCG   
  
  
- TGTTAAGATA CCTTTACCTT TTACTTCAGT GAATACCTCT AAGTTATCAT TCGTTTAGCT CTCGTCTTTT   
  
  
- AGATTAGAGG AAAGCAGTAA TATGTTAGTT ATAACGGATA AACCAAAATC ACTAAACAAA GTCGGCGTTT   
  
  
- TGAATTAAAA GAAACCACGT ATACTTTAGA ATTTTTCTTA ATTTTATTCT ATATTATGCA ATTGAGAAGT   
  
  
- TGTTGTACTA TATAAATTCT ATGGTTATAA ACATAAGTAG TATATTTTTA ACATCACAAC GTTTTATAAT   
  
  
- TTTAATAAAG TATTTATTTG TAATAATATT CTTGACTGAG GCGCCTAATA GTATACCATA TCTAATGTCT   
  
  
- CACCCACCCA AATTTATTAA AGGGGCTGTG TAATATGAAA ATCTTCTCGA TAATCGAGGT TTAATAAATT   
  
  
- TGTTACAAGA TTTCTCGTTT TTTTAGTTAC AAAACCTAAA ACTAGGCTGG CTCTCCTGAA CAGGCTGGTA   
  
  
- AGGTATAATT TTACAAGGAT CAAGACTAAT CGATTATGGT

+     MYB-like sequence

| Site Name | Organism | Position | Strand | Matrix score. | sequence | function |
| --- | --- | --- | --- | --- | --- | --- |
| MYB-like sequence | Arabidopsis thaliana | 1099 | - | 6 | TAACCA |  |

>PlantCARE\_25378   
+ ATAAAGTTGT CGTGATTTCT TGCCCTTTAT TTTTTATTTA TATTAACATA TATTTTTAAA AAAAAATTAA   
  
  
+ AGACGTTCCT AACCGAAACT CATGTATTTT TTGATTATGA ATATGAATAT CCCCTCTATA TTAATCATGG   
  
  
+ AGCATTACAA CATGTTTTCG TAGCCATATG TCATCACGAG AATGATTTTT AGAATTGTTA GAAAAATAAA   
  
  
+ TTGATTCATA TAAACATATA CTATGTTTTT TATTAAACTA ACTATCAAAT TAATTAATAG TGTACAAAAA   
  
  
+ AATATTTTTT TCTTTCCTTA AATAAAAACT ACGGAATTAC CTAATATGGC TAACATATAT ATGACAATTA   
  
  
+ ATGATTATGA ATAATACATA TTTGATAAAA AAATTTCTAA CCTCTCTCTT TTTTGTTTAA TTTTATATTA   
  
  
+ TTAAAGGAAA TTTAACAATC ACATTAATCA TATAATAAAA ACAATTAGAT TTTTTCTTAT ATGTTATATT   
  
  
+ TTGAATTTTT AAAAACGACT ATAAATTACT AAAAATGATA AGAGTCCCAC ATTAAAAAAT TTGTGATCAA   
  
  
+ CCGTTTAACT TTTTTTTTAG TTCAAGCAAG ATACAAATGA TCATATATCT GATATAGACG TGGGCGTTCG   
  
  
+ GATACACGTT CGGGTTTGTA TCAGATATTT CAGTATAAAG GTATAGAACC CGTTCGGGTA TTTCTACACT   
  
  
+ CCGAGTCGGG TTCGGGTTCG GATATTTTGG ATCGGGTTCG AATATTTAAA TTTTGAAGAA AAAAAGAAAT   
  
  
+ TATTCACTGT TTAAGTTTTT TATATTTAAA TATATCTTAA CTTAACTGAT TTTTTTTAGT TTTTAAAAGA   
  
  
+ TTAAAATATT AATATGTTTG GAGATAAAAC TTTAAAAATA GAAAGACACT AATTTAGTTT TTGTTTTGAA   
  
  
+ AATTTAGATG CAACTTTTGT TAATGCAAGA AACAAGAACT TGATATGTAT TTTAAGTGAG TAACAAATGA   
  
  
+ TTTTGTCTAT AGTTATATGT ATATTATCTA ATTTTGAGTA ATAAGAATCA TTAATATAAA TATTTTGAAT   
  
  
+ AAAATTAGAT AGATAAACTA TAAATATAGA GTTAAGTATA CTTATGTTTG GTTATCTTCG AATATTACCC   
  
  
+ GTTCGGATAT ATTATCTGAA CTGGTGAAAT AAGTAATATG TTTTGTTGTT TTAATTAGAT AATTTTTAGA   
  
  
+ CCGAGCTTGT GAATATATAC TAGACAAACA TTTATATTTC GAGTCTGCAC TTATATTCTA TAAGAGCTTG   
  
  
+ ATATATTAGA TTTGAACACT AACCTGTTAA TATAGTTTGC CGGTGATTTT TTTTCAAAAT TTTGATTCTT   
  
  
+ AGATATGTAT ATGGAGTAAA ACTAATTTTT ACAGATGCCC ATTTTTTTAA TTGACACTTA TGTAATTAAC   
  
  
+ TGAATTCATA AAACAAGGTT TTTTAAAAAA ATTTAACTCA TATCAATGAA ACAAAGACGA GAACGAAAGC   
  
  
+ ACAATTCTAT GGAAATGGAA AATGAAGTCA CTTATGGAGA TTCAATAGTA AGCAAATCGA GAGCAGAAAA   
  
  
+ TCTAATCTCC TTTCGTCATT ATACAATCAA TATTGCCTAT TTGGTTTTAG TGATTTGTTT CAGCCGCAAA   
  
  
+ ACTTAATTTT CTTTGGTGCA TATGAAATCT TAAAAAGAAT TAAAATAAGA TATAATACGT TAACTCTTCA   
  
  
+ ACAACATGAT ATATTTAAGA TACCAATATT TGTATTCATC ATATAAAAAT TGTAGTGTTG CAAAATATTA   
  
  
+ AAATTATTTC ATAAATAAAC ATTATTATAA GAACTGACTC CGCGGATTAT CATATGGTAT AGATTACAGA   
  
  
+ GTGGGTGGGT TTAAATAATT TCCCCGACAC ATTATACTTT TAGAAGAGCT ATTAGCTCCA AATTATTTAA   
  
  
+ ACAATGTTCT AAAGAGCAAA AAAATCAATG TTTTGGATTT TGATCCGACC GAGAGGACTT GTCCGACCAT   
  
  
+ TCCATATTAA AATGTTCCTA GTTCTGATTA GCTAATACCA   

- TATTTCAACA GCACTAAAGA ACGGGAAATA AAAAATAAAT ATAATTGTAT ATAAAAATTT TTTTTTAATT   
  
  
- TCTGCAAGGA TTGGCTTTGA GTACATAAAA AACTAATACT TATACTTATA GGGGAGATAT AATTAGTACC   
  
  
- TCGTAATGTT GTACAAAAGC ATCGGTATAC AGTAGTGCTC TTACTAAAAA TCTTAACAAT CTTTTTATTT   
  
  
- AACTAAGTAT ATTTGTATAT GATACAAAAA ATAATTTGAT TGATAGTTTA ATTAATTATC ACATGTTTTT   
  
  
- TTATAAAAAA AGAAAGGAAT TTATTTTTGA TGCCTTAATG GATTATACCG ATTGTATATA TACTGTTAAT   
  
  
- TACTAATACT TATTATGTAT AAACTATTTT TTTAAAGATT GGAGAGAGAA AAAACAAATT AAAATATAAT   
  
  
- AATTTCCTTT AAATTGTTAG TGTAATTAGT ATATTATTTT TGTTAATCTA AAAAAGAATA TACAATATAA   
  
  
- AACTTAAAAA TTTTTGCTGA TATTTAATGA TTTTTACTAT TCTCAGGGTG TAATTTTTTA AACACTAGTT   
  
  
- GGCAAATTGA AAAAAAAATC AAGTTCGTTC TATGTTTACT AGTATATAGA CTATATCTGC ACCCGCAAGC   
  
  
- CTATGTGCAA GCCCAAACAT AGTCTATAAA GTCATATTTC CATATCTTGG GCAAGCCCAT AAAGATGTGA   
  
  
- GGCTCAGCCC AAGCCCAAGC CTATAAAACC TAGCCCAAGC TTATAAATTT AAAACTTCTT TTTTTCTTTA   
  
  
- ATAAGTGACA AATTCAAAAA ATATAAATTT ATATAGAATT GAATTGACTA AAAAAAATCA AAAATTTTCT   
  
  
- AATTTTATAA TTATACAAAC CTCTATTTTG AAATTTTTAT CTTTCTGTGA TTAAATCAAA AACAAAACTT   
  
  
- TTAAATCTAC GTTGAAAACA ATTACGTTCT TTGTTCTTGA ACTATACATA AAATTCACTC ATTGTTTACT   
  
  
- AAAACAGATA TCAATATACA TATAATAGAT TAAAACTCAT TATTCTTAGT AATTATATTT ATAAAACTTA   
  
  
- TTTTAATCTA TCTATTTGAT ATTTATATCT CAATTCATAT GAATACAAAC CAATAGAAGC TTATAATGGG   
  
  
- CAAGCCTATA TAATAGACTT GACCACTTTA TTCATTATAC AAAACAACAA AATTAATCTA TTAAAAATCT   
  
  
- GGCTCGAACA CTTATATATG ATCTGTTTGT AAATATAAAG CTCAGACGTG AATATAAGAT ATTCTCGAAC   
  
  
- TATATAATCT AAACTTGTGA TTGGACAATT ATATCAAACG GCCACTAAAA AAAAGTTTTA AAACTAAGAA   
  
  
- TCTATACATA TACCTCATTT TGATTAAAAA TGTCTACGGG TAAAAAAATT AACTGTGAAT ACATTAATTG   
  
  
- ACTTAAGTAT TTTGTTCCAA AAAATTTTTT TAAATTGAGT ATAGTTACTT TGTTTCTGCT CTTGCTTTCG   
  
  
- TGTTAAGATA CCTTTACCTT TTACTTCAGT GAATACCTCT AAGTTATCAT TCGTTTAGCT CTCGTCTTTT   
  
  
- AGATTAGAGG AAAGCAGTAA TATGTTAGTT ATAACGGATA AACCAAAATC ACTAAACAAA GTCGGCGTTT   
  
  
- TGAATTAAAA GAAACCACGT ATACTTTAGA ATTTTTCTTA ATTTTATTCT ATATTATGCA ATTGAGAAGT   
  
  
- TGTTGTACTA TATAAATTCT ATGGTTATAA ACATAAGTAG TATATTTTTA ACATCACAAC GTTTTATAAT   
  
  
- TTTAATAAAG TATTTATTTG TAATAATATT CTTGACTGAG GCGCCTAATA GTATACCATA TCTAATGTCT   
  
  
- CACCCACCCA AATTTATTAA AGGGGCTGTG TAATATGAAA ATCTTCTCGA TAATCGAGGT TTAATAAATT   
  
  
- TGTTACAAGA TTTCTCGTTT TTTTAGTTAC AAAACCTAAA ACTAGGCTGG CTCTCCTGAA CAGGCTGGTA   
  
  
- AGGTATAATT TTACAAGGAT CAAGACTAAT CGATTATGGT

+     MYC

| Site Name | Organism | Position | Strand | Matrix score. | sequence | function |
| --- | --- | --- | --- | --- | --- | --- |
| MYC | Arabidopsis thaliana | 594 | - | 6 | CATTTG |  |
| MYC | Arabidopsis thaliana | 974 | - | 6 | CATTTG |  |

>PlantCARE\_25378   
+ ATAAAGTTGT CGTGATTTCT TGCCCTTTAT TTTTTATTTA TATTAACATA TATTTTTAAA AAAAAATTAA   
  
  
+ AGACGTTCCT AACCGAAACT CATGTATTTT TTGATTATGA ATATGAATAT CCCCTCTATA TTAATCATGG   
  
  
+ AGCATTACAA CATGTTTTCG TAGCCATATG TCATCACGAG AATGATTTTT AGAATTGTTA GAAAAATAAA   
  
  
+ TTGATTCATA TAAACATATA CTATGTTTTT TATTAAACTA ACTATCAAAT TAATTAATAG TGTACAAAAA   
  
  
+ AATATTTTTT TCTTTCCTTA AATAAAAACT ACGGAATTAC CTAATATGGC TAACATATAT ATGACAATTA   
  
  
+ ATGATTATGA ATAATACATA TTTGATAAAA AAATTTCTAA CCTCTCTCTT TTTTGTTTAA TTTTATATTA   
  
  
+ TTAAAGGAAA TTTAACAATC ACATTAATCA TATAATAAAA ACAATTAGAT TTTTTCTTAT ATGTTATATT   
  
  
+ TTGAATTTTT AAAAACGACT ATAAATTACT AAAAATGATA AGAGTCCCAC ATTAAAAAAT TTGTGATCAA   
  
  
+ CCGTTTAACT TTTTTTTTAG TTCAAGCAAG ATACAAATGA TCATATATCT GATATAGACG TGGGCGTTCG   
  
  
+ GATACACGTT CGGGTTTGTA TCAGATATTT CAGTATAAAG GTATAGAACC CGTTCGGGTA TTTCTACACT   
  
  
+ CCGAGTCGGG TTCGGGTTCG GATATTTTGG ATCGGGTTCG AATATTTAAA TTTTGAAGAA AAAAAGAAAT   
  
  
+ TATTCACTGT TTAAGTTTTT TATATTTAAA TATATCTTAA CTTAACTGAT TTTTTTTAGT TTTTAAAAGA   
  
  
+ TTAAAATATT AATATGTTTG GAGATAAAAC TTTAAAAATA GAAAGACACT AATTTAGTTT TTGTTTTGAA   
  
  
+ AATTTAGATG CAACTTTTGT TAATGCAAGA AACAAGAACT TGATATGTAT TTTAAGTGAG TAACAAATGA   
  
  
+ TTTTGTCTAT AGTTATATGT ATATTATCTA ATTTTGAGTA ATAAGAATCA TTAATATAAA TATTTTGAAT   
  
  
+ AAAATTAGAT AGATAAACTA TAAATATAGA GTTAAGTATA CTTATGTTTG GTTATCTTCG AATATTACCC   
  
  
+ GTTCGGATAT ATTATCTGAA CTGGTGAAAT AAGTAATATG TTTTGTTGTT TTAATTAGAT AATTTTTAGA   
  
  
+ CCGAGCTTGT GAATATATAC TAGACAAACA TTTATATTTC GAGTCTGCAC TTATATTCTA TAAGAGCTTG   
  
  
+ ATATATTAGA TTTGAACACT AACCTGTTAA TATAGTTTGC CGGTGATTTT TTTTCAAAAT TTTGATTCTT   
  
  
+ AGATATGTAT ATGGAGTAAA ACTAATTTTT ACAGATGCCC ATTTTTTTAA TTGACACTTA TGTAATTAAC   
  
  
+ TGAATTCATA AAACAAGGTT TTTTAAAAAA ATTTAACTCA TATCAATGAA ACAAAGACGA GAACGAAAGC   
  
  
+ ACAATTCTAT GGAAATGGAA AATGAAGTCA CTTATGGAGA TTCAATAGTA AGCAAATCGA GAGCAGAAAA   
  
  
+ TCTAATCTCC TTTCGTCATT ATACAATCAA TATTGCCTAT TTGGTTTTAG TGATTTGTTT CAGCCGCAAA   
  
  
+ ACTTAATTTT CTTTGGTGCA TATGAAATCT TAAAAAGAAT TAAAATAAGA TATAATACGT TAACTCTTCA   
  
  
+ ACAACATGAT ATATTTAAGA TACCAATATT TGTATTCATC ATATAAAAAT TGTAGTGTTG CAAAATATTA   
  
  
+ AAATTATTTC ATAAATAAAC ATTATTATAA GAACTGACTC CGCGGATTAT CATATGGTAT AGATTACAGA   
  
  
+ GTGGGTGGGT TTAAATAATT TCCCCGACAC ATTATACTTT TAGAAGAGCT ATTAGCTCCA AATTATTTAA   
  
  
+ ACAATGTTCT AAAGAGCAAA AAAATCAATG TTTTGGATTT TGATCCGACC GAGAGGACTT GTCCGACCAT   
  
  
+ TCCATATTAA AATGTTCCTA GTTCTGATTA GCTAATACCA   

- TATTTCAACA GCACTAAAGA ACGGGAAATA AAAAATAAAT ATAATTGTAT ATAAAAATTT TTTTTTAATT   
  
  
- TCTGCAAGGA TTGGCTTTGA GTACATAAAA AACTAATACT TATACTTATA GGGGAGATAT AATTAGTACC   
  
  
- TCGTAATGTT GTACAAAAGC ATCGGTATAC AGTAGTGCTC TTACTAAAAA TCTTAACAAT CTTTTTATTT   
  
  
- AACTAAGTAT ATTTGTATAT GATACAAAAA ATAATTTGAT TGATAGTTTA ATTAATTATC ACATGTTTTT   
  
  
- TTATAAAAAA AGAAAGGAAT TTATTTTTGA TGCCTTAATG GATTATACCG ATTGTATATA TACTGTTAAT   
  
  
- TACTAATACT TATTATGTAT AAACTATTTT TTTAAAGATT GGAGAGAGAA AAAACAAATT AAAATATAAT   
  
  
- AATTTCCTTT AAATTGTTAG TGTAATTAGT ATATTATTTT TGTTAATCTA AAAAAGAATA TACAATATAA   
  
  
- AACTTAAAAA TTTTTGCTGA TATTTAATGA TTTTTACTAT TCTCAGGGTG TAATTTTTTA AACACTAGTT   
  
  
- GGCAAATTGA AAAAAAAATC AAGTTCGTTC TATGTTTACT AGTATATAGA CTATATCTGC ACCCGCAAGC   
  
  
- CTATGTGCAA GCCCAAACAT AGTCTATAAA GTCATATTTC CATATCTTGG GCAAGCCCAT AAAGATGTGA   
  
  
- GGCTCAGCCC AAGCCCAAGC CTATAAAACC TAGCCCAAGC TTATAAATTT AAAACTTCTT TTTTTCTTTA   
  
  
- ATAAGTGACA AATTCAAAAA ATATAAATTT ATATAGAATT GAATTGACTA AAAAAAATCA AAAATTTTCT   
  
  
- AATTTTATAA TTATACAAAC CTCTATTTTG AAATTTTTAT CTTTCTGTGA TTAAATCAAA AACAAAACTT   
  
  
- TTAAATCTAC GTTGAAAACA ATTACGTTCT TTGTTCTTGA ACTATACATA AAATTCACTC ATTGTTTACT   
  
  
- AAAACAGATA TCAATATACA TATAATAGAT TAAAACTCAT TATTCTTAGT AATTATATTT ATAAAACTTA   
  
  
- TTTTAATCTA TCTATTTGAT ATTTATATCT CAATTCATAT GAATACAAAC CAATAGAAGC TTATAATGGG   
  
  
- CAAGCCTATA TAATAGACTT GACCACTTTA TTCATTATAC AAAACAACAA AATTAATCTA TTAAAAATCT   
  
  
- GGCTCGAACA CTTATATATG ATCTGTTTGT AAATATAAAG CTCAGACGTG AATATAAGAT ATTCTCGAAC   
  
  
- TATATAATCT AAACTTGTGA TTGGACAATT ATATCAAACG GCCACTAAAA AAAAGTTTTA AAACTAAGAA   
  
  
- TCTATACATA TACCTCATTT TGATTAAAAA TGTCTACGGG TAAAAAAATT AACTGTGAAT ACATTAATTG   
  
  
- ACTTAAGTAT TTTGTTCCAA AAAATTTTTT TAAATTGAGT ATAGTTACTT TGTTTCTGCT CTTGCTTTCG   
  
  
- TGTTAAGATA CCTTTACCTT TTACTTCAGT GAATACCTCT AAGTTATCAT TCGTTTAGCT CTCGTCTTTT   
  
  
- AGATTAGAGG AAAGCAGTAA TATGTTAGTT ATAACGGATA AACCAAAATC ACTAAACAAA GTCGGCGTTT   
  
  
- TGAATTAAAA GAAACCACGT ATACTTTAGA ATTTTTCTTA ATTTTATTCT ATATTATGCA ATTGAGAAGT   
  
  
- TGTTGTACTA TATAAATTCT ATGGTTATAA ACATAAGTAG TATATTTTTA ACATCACAAC GTTTTATAAT   
  
  
- TTTAATAAAG TATTTATTTG TAATAATATT CTTGACTGAG GCGCCTAATA GTATACCATA TCTAATGTCT   
  
  
- CACCCACCCA AATTTATTAA AGGGGCTGTG TAATATGAAA ATCTTCTCGA TAATCGAGGT TTAATAAATT   
  
  
- TGTTACAAGA TTTCTCGTTT TTTTAGTTAC AAAACCTAAA ACTAGGCTGG CTCTCCTGAA CAGGCTGGTA   
  
  
- AGGTATAATT TTACAAGGAT CAAGACTAAT CGATTATGGT

+     Myb

| Site Name | Organism | Position | Strand | Matrix score. | sequence | function |
| --- | --- | --- | --- | --- | --- | --- |
| Myb | Arabidopsis thaliana | 813 | + | 6 | TAACTG |  |
| Myb | Arabidopsis thaliana | 1397 | + | 6 | TAACTG |  |

>PlantCARE\_25378   
+ ATAAAGTTGT CGTGATTTCT TGCCCTTTAT TTTTTATTTA TATTAACATA TATTTTTAAA AAAAAATTAA   
  
  
+ AGACGTTCCT AACCGAAACT CATGTATTTT TTGATTATGA ATATGAATAT CCCCTCTATA TTAATCATGG   
  
  
+ AGCATTACAA CATGTTTTCG TAGCCATATG TCATCACGAG AATGATTTTT AGAATTGTTA GAAAAATAAA   
  
  
+ TTGATTCATA TAAACATATA CTATGTTTTT TATTAAACTA ACTATCAAAT TAATTAATAG TGTACAAAAA   
  
  
+ AATATTTTTT TCTTTCCTTA AATAAAAACT ACGGAATTAC CTAATATGGC TAACATATAT ATGACAATTA   
  
  
+ ATGATTATGA ATAATACATA TTTGATAAAA AAATTTCTAA CCTCTCTCTT TTTTGTTTAA TTTTATATTA   
  
  
+ TTAAAGGAAA TTTAACAATC ACATTAATCA TATAATAAAA ACAATTAGAT TTTTTCTTAT ATGTTATATT   
  
  
+ TTGAATTTTT AAAAACGACT ATAAATTACT AAAAATGATA AGAGTCCCAC ATTAAAAAAT TTGTGATCAA   
  
  
+ CCGTTTAACT TTTTTTTTAG TTCAAGCAAG ATACAAATGA TCATATATCT GATATAGACG TGGGCGTTCG   
  
  
+ GATACACGTT CGGGTTTGTA TCAGATATTT CAGTATAAAG GTATAGAACC CGTTCGGGTA TTTCTACACT   
  
  
+ CCGAGTCGGG TTCGGGTTCG GATATTTTGG ATCGGGTTCG AATATTTAAA TTTTGAAGAA AAAAAGAAAT   
  
  
+ TATTCACTGT TTAAGTTTTT TATATTTAAA TATATCTTAA CTTAACTGAT TTTTTTTAGT TTTTAAAAGA   
  
  
+ TTAAAATATT AATATGTTTG GAGATAAAAC TTTAAAAATA GAAAGACACT AATTTAGTTT TTGTTTTGAA   
  
  
+ AATTTAGATG CAACTTTTGT TAATGCAAGA AACAAGAACT TGATATGTAT TTTAAGTGAG TAACAAATGA   
  
  
+ TTTTGTCTAT AGTTATATGT ATATTATCTA ATTTTGAGTA ATAAGAATCA TTAATATAAA TATTTTGAAT   
  
  
+ AAAATTAGAT AGATAAACTA TAAATATAGA GTTAAGTATA CTTATGTTTG GTTATCTTCG AATATTACCC   
  
  
+ GTTCGGATAT ATTATCTGAA CTGGTGAAAT AAGTAATATG TTTTGTTGTT TTAATTAGAT AATTTTTAGA   
  
  
+ CCGAGCTTGT GAATATATAC TAGACAAACA TTTATATTTC GAGTCTGCAC TTATATTCTA TAAGAGCTTG   
  
  
+ ATATATTAGA TTTGAACACT AACCTGTTAA TATAGTTTGC CGGTGATTTT TTTTCAAAAT TTTGATTCTT   
  
  
+ AGATATGTAT ATGGAGTAAA ACTAATTTTT ACAGATGCCC ATTTTTTTAA TTGACACTTA TGTAATTAAC   
  
  
+ TGAATTCATA AAACAAGGTT TTTTAAAAAA ATTTAACTCA TATCAATGAA ACAAAGACGA GAACGAAAGC   
  
  
+ ACAATTCTAT GGAAATGGAA AATGAAGTCA CTTATGGAGA TTCAATAGTA AGCAAATCGA GAGCAGAAAA   
  
  
+ TCTAATCTCC TTTCGTCATT ATACAATCAA TATTGCCTAT TTGGTTTTAG TGATTTGTTT CAGCCGCAAA   
  
  
+ ACTTAATTTT CTTTGGTGCA TATGAAATCT TAAAAAGAAT TAAAATAAGA TATAATACGT TAACTCTTCA   
  
  
+ ACAACATGAT ATATTTAAGA TACCAATATT TGTATTCATC ATATAAAAAT TGTAGTGTTG CAAAATATTA   
  
  
+ AAATTATTTC ATAAATAAAC ATTATTATAA GAACTGACTC CGCGGATTAT CATATGGTAT AGATTACAGA   
  
  
+ GTGGGTGGGT TTAAATAATT TCCCCGACAC ATTATACTTT TAGAAGAGCT ATTAGCTCCA AATTATTTAA   
  
  
+ ACAATGTTCT AAAGAGCAAA AAAATCAATG TTTTGGATTT TGATCCGACC GAGAGGACTT GTCCGACCAT   
  
  
+ TCCATATTAA AATGTTCCTA GTTCTGATTA GCTAATACCA   

- TATTTCAACA GCACTAAAGA ACGGGAAATA AAAAATAAAT ATAATTGTAT ATAAAAATTT TTTTTTAATT   
  
  
- TCTGCAAGGA TTGGCTTTGA GTACATAAAA AACTAATACT TATACTTATA GGGGAGATAT AATTAGTACC   
  
  
- TCGTAATGTT GTACAAAAGC ATCGGTATAC AGTAGTGCTC TTACTAAAAA TCTTAACAAT CTTTTTATTT   
  
  
- AACTAAGTAT ATTTGTATAT GATACAAAAA ATAATTTGAT TGATAGTTTA ATTAATTATC ACATGTTTTT   
  
  
- TTATAAAAAA AGAAAGGAAT TTATTTTTGA TGCCTTAATG GATTATACCG ATTGTATATA TACTGTTAAT   
  
  
- TACTAATACT TATTATGTAT AAACTATTTT TTTAAAGATT GGAGAGAGAA AAAACAAATT AAAATATAAT   
  
  
- AATTTCCTTT AAATTGTTAG TGTAATTAGT ATATTATTTT TGTTAATCTA AAAAAGAATA TACAATATAA   
  
  
- AACTTAAAAA TTTTTGCTGA TATTTAATGA TTTTTACTAT TCTCAGGGTG TAATTTTTTA AACACTAGTT   
  
  
- GGCAAATTGA AAAAAAAATC AAGTTCGTTC TATGTTTACT AGTATATAGA CTATATCTGC ACCCGCAAGC   
  
  
- CTATGTGCAA GCCCAAACAT AGTCTATAAA GTCATATTTC CATATCTTGG GCAAGCCCAT AAAGATGTGA   
  
  
- GGCTCAGCCC AAGCCCAAGC CTATAAAACC TAGCCCAAGC TTATAAATTT AAAACTTCTT TTTTTCTTTA   
  
  
- ATAAGTGACA AATTCAAAAA ATATAAATTT ATATAGAATT GAATTGACTA AAAAAAATCA AAAATTTTCT   
  
  
- AATTTTATAA TTATACAAAC CTCTATTTTG AAATTTTTAT CTTTCTGTGA TTAAATCAAA AACAAAACTT   
  
  
- TTAAATCTAC GTTGAAAACA ATTACGTTCT TTGTTCTTGA ACTATACATA AAATTCACTC ATTGTTTACT   
  
  
- AAAACAGATA TCAATATACA TATAATAGAT TAAAACTCAT TATTCTTAGT AATTATATTT ATAAAACTTA   
  
  
- TTTTAATCTA TCTATTTGAT ATTTATATCT CAATTCATAT GAATACAAAC CAATAGAAGC TTATAATGGG   
  
  
- CAAGCCTATA TAATAGACTT GACCACTTTA TTCATTATAC AAAACAACAA AATTAATCTA TTAAAAATCT   
  
  
- GGCTCGAACA CTTATATATG ATCTGTTTGT AAATATAAAG CTCAGACGTG AATATAAGAT ATTCTCGAAC   
  
  
- TATATAATCT AAACTTGTGA TTGGACAATT ATATCAAACG GCCACTAAAA AAAAGTTTTA AAACTAAGAA   
  
  
- TCTATACATA TACCTCATTT TGATTAAAAA TGTCTACGGG TAAAAAAATT AACTGTGAAT ACATTAATTG   
  
  
- ACTTAAGTAT TTTGTTCCAA AAAATTTTTT TAAATTGAGT ATAGTTACTT TGTTTCTGCT CTTGCTTTCG   
  
  
- TGTTAAGATA CCTTTACCTT TTACTTCAGT GAATACCTCT AAGTTATCAT TCGTTTAGCT CTCGTCTTTT   
  
  
- AGATTAGAGG AAAGCAGTAA TATGTTAGTT ATAACGGATA AACCAAAATC ACTAAACAAA GTCGGCGTTT   
  
  
- TGAATTAAAA GAAACCACGT ATACTTTAGA ATTTTTCTTA ATTTTATTCT ATATTATGCA ATTGAGAAGT   
  
  
- TGTTGTACTA TATAAATTCT ATGGTTATAA ACATAAGTAG TATATTTTTA ACATCACAAC GTTTTATAAT   
  
  
- TTTAATAAAG TATTTATTTG TAATAATATT CTTGACTGAG GCGCCTAATA GTATACCATA TCTAATGTCT   
  
  
- CACCCACCCA AATTTATTAA AGGGGCTGTG TAATATGAAA ATCTTCTCGA TAATCGAGGT TTAATAAATT   
  
  
- TGTTACAAGA TTTCTCGTTT TTTTAGTTAC AAAACCTAAA ACTAGGCTGG CTCTCCTGAA CAGGCTGGTA   
  
  
- AGGTATAATT TTACAAGGAT CAAGACTAAT CGATTATGGT

+     O2-site

| Site Name | Organism | Position | Strand | Matrix score. | sequence | function |
| --- | --- | --- | --- | --- | --- | --- |
| O2-site | Zea mays | 6 | + | 9 | GTTGACGTGA | cis-acting regulatory element involved in zein metabolism regulation |

>PlantCARE\_25378   
+ ATAAAGTTGT CGTGATTTCT TGCCCTTTAT TTTTTATTTA TATTAACATA TATTTTTAAA AAAAAATTAA   
  
  
+ AGACGTTCCT AACCGAAACT CATGTATTTT TTGATTATGA ATATGAATAT CCCCTCTATA TTAATCATGG   
  
  
+ AGCATTACAA CATGTTTTCG TAGCCATATG TCATCACGAG AATGATTTTT AGAATTGTTA GAAAAATAAA   
  
  
+ TTGATTCATA TAAACATATA CTATGTTTTT TATTAAACTA ACTATCAAAT TAATTAATAG TGTACAAAAA   
  
  
+ AATATTTTTT TCTTTCCTTA AATAAAAACT ACGGAATTAC CTAATATGGC TAACATATAT ATGACAATTA   
  
  
+ ATGATTATGA ATAATACATA TTTGATAAAA AAATTTCTAA CCTCTCTCTT TTTTGTTTAA TTTTATATTA   
  
  
+ TTAAAGGAAA TTTAACAATC ACATTAATCA TATAATAAAA ACAATTAGAT TTTTTCTTAT ATGTTATATT   
  
  
+ TTGAATTTTT AAAAACGACT ATAAATTACT AAAAATGATA AGAGTCCCAC ATTAAAAAAT TTGTGATCAA   
  
  
+ CCGTTTAACT TTTTTTTTAG TTCAAGCAAG ATACAAATGA TCATATATCT GATATAGACG TGGGCGTTCG   
  
  
+ GATACACGTT CGGGTTTGTA TCAGATATTT CAGTATAAAG GTATAGAACC CGTTCGGGTA TTTCTACACT   
  
  
+ CCGAGTCGGG TTCGGGTTCG GATATTTTGG ATCGGGTTCG AATATTTAAA TTTTGAAGAA AAAAAGAAAT   
  
  
+ TATTCACTGT TTAAGTTTTT TATATTTAAA TATATCTTAA CTTAACTGAT TTTTTTTAGT TTTTAAAAGA   
  
  
+ TTAAAATATT AATATGTTTG GAGATAAAAC TTTAAAAATA GAAAGACACT AATTTAGTTT TTGTTTTGAA   
  
  
+ AATTTAGATG CAACTTTTGT TAATGCAAGA AACAAGAACT TGATATGTAT TTTAAGTGAG TAACAAATGA   
  
  
+ TTTTGTCTAT AGTTATATGT ATATTATCTA ATTTTGAGTA ATAAGAATCA TTAATATAAA TATTTTGAAT   
  
  
+ AAAATTAGAT AGATAAACTA TAAATATAGA GTTAAGTATA CTTATGTTTG GTTATCTTCG AATATTACCC   
  
  
+ GTTCGGATAT ATTATCTGAA CTGGTGAAAT AAGTAATATG TTTTGTTGTT TTAATTAGAT AATTTTTAGA   
  
  
+ CCGAGCTTGT GAATATATAC TAGACAAACA TTTATATTTC GAGTCTGCAC TTATATTCTA TAAGAGCTTG   
  
  
+ ATATATTAGA TTTGAACACT AACCTGTTAA TATAGTTTGC CGGTGATTTT TTTTCAAAAT TTTGATTCTT   
  
  
+ AGATATGTAT ATGGAGTAAA ACTAATTTTT ACAGATGCCC ATTTTTTTAA TTGACACTTA TGTAATTAAC   
  
  
+ TGAATTCATA AAACAAGGTT TTTTAAAAAA ATTTAACTCA TATCAATGAA ACAAAGACGA GAACGAAAGC   
  
  
+ ACAATTCTAT GGAAATGGAA AATGAAGTCA CTTATGGAGA TTCAATAGTA AGCAAATCGA GAGCAGAAAA   
  
  
+ TCTAATCTCC TTTCGTCATT ATACAATCAA TATTGCCTAT TTGGTTTTAG TGATTTGTTT CAGCCGCAAA   
  
  
+ ACTTAATTTT CTTTGGTGCA TATGAAATCT TAAAAAGAAT TAAAATAAGA TATAATACGT TAACTCTTCA   
  
  
+ ACAACATGAT ATATTTAAGA TACCAATATT TGTATTCATC ATATAAAAAT TGTAGTGTTG CAAAATATTA   
  
  
+ AAATTATTTC ATAAATAAAC ATTATTATAA GAACTGACTC CGCGGATTAT CATATGGTAT AGATTACAGA   
  
  
+ GTGGGTGGGT TTAAATAATT TCCCCGACAC ATTATACTTT TAGAAGAGCT ATTAGCTCCA AATTATTTAA   
  
  
+ ACAATGTTCT AAAGAGCAAA AAAATCAATG TTTTGGATTT TGATCCGACC GAGAGGACTT GTCCGACCAT   
  
  
+ TCCATATTAA AATGTTCCTA GTTCTGATTA GCTAATACCA   

- TATTTCAACA GCACTAAAGA ACGGGAAATA AAAAATAAAT ATAATTGTAT ATAAAAATTT TTTTTTAATT   
  
  
- TCTGCAAGGA TTGGCTTTGA GTACATAAAA AACTAATACT TATACTTATA GGGGAGATAT AATTAGTACC   
  
  
- TCGTAATGTT GTACAAAAGC ATCGGTATAC AGTAGTGCTC TTACTAAAAA TCTTAACAAT CTTTTTATTT   
  
  
- AACTAAGTAT ATTTGTATAT GATACAAAAA ATAATTTGAT TGATAGTTTA ATTAATTATC ACATGTTTTT   
  
  
- TTATAAAAAA AGAAAGGAAT TTATTTTTGA TGCCTTAATG GATTATACCG ATTGTATATA TACTGTTAAT   
  
  
- TACTAATACT TATTATGTAT AAACTATTTT TTTAAAGATT GGAGAGAGAA AAAACAAATT AAAATATAAT   
  
  
- AATTTCCTTT AAATTGTTAG TGTAATTAGT ATATTATTTT TGTTAATCTA AAAAAGAATA TACAATATAA   
  
  
- AACTTAAAAA TTTTTGCTGA TATTTAATGA TTTTTACTAT TCTCAGGGTG TAATTTTTTA AACACTAGTT   
  
  
- GGCAAATTGA AAAAAAAATC AAGTTCGTTC TATGTTTACT AGTATATAGA CTATATCTGC ACCCGCAAGC   
  
  
- CTATGTGCAA GCCCAAACAT AGTCTATAAA GTCATATTTC CATATCTTGG GCAAGCCCAT AAAGATGTGA   
  
  
- GGCTCAGCCC AAGCCCAAGC CTATAAAACC TAGCCCAAGC TTATAAATTT AAAACTTCTT TTTTTCTTTA   
  
  
- ATAAGTGACA AATTCAAAAA ATATAAATTT ATATAGAATT GAATTGACTA AAAAAAATCA AAAATTTTCT   
  
  
- AATTTTATAA TTATACAAAC CTCTATTTTG AAATTTTTAT CTTTCTGTGA TTAAATCAAA AACAAAACTT   
  
  
- TTAAATCTAC GTTGAAAACA ATTACGTTCT TTGTTCTTGA ACTATACATA AAATTCACTC ATTGTTTACT   
  
  
- AAAACAGATA TCAATATACA TATAATAGAT TAAAACTCAT TATTCTTAGT AATTATATTT ATAAAACTTA   
  
  
- TTTTAATCTA TCTATTTGAT ATTTATATCT CAATTCATAT GAATACAAAC CAATAGAAGC TTATAATGGG   
  
  
- CAAGCCTATA TAATAGACTT GACCACTTTA TTCATTATAC AAAACAACAA AATTAATCTA TTAAAAATCT   
  
  
- GGCTCGAACA CTTATATATG ATCTGTTTGT AAATATAAAG CTCAGACGTG AATATAAGAT ATTCTCGAAC   
  
  
- TATATAATCT AAACTTGTGA TTGGACAATT ATATCAAACG GCCACTAAAA AAAAGTTTTA AAACTAAGAA   
  
  
- TCTATACATA TACCTCATTT TGATTAAAAA TGTCTACGGG TAAAAAAATT AACTGTGAAT ACATTAATTG   
  
  
- ACTTAAGTAT TTTGTTCCAA AAAATTTTTT TAAATTGAGT ATAGTTACTT TGTTTCTGCT CTTGCTTTCG   
  
  
- TGTTAAGATA CCTTTACCTT TTACTTCAGT GAATACCTCT AAGTTATCAT TCGTTTAGCT CTCGTCTTTT   
  
  
- AGATTAGAGG AAAGCAGTAA TATGTTAGTT ATAACGGATA AACCAAAATC ACTAAACAAA GTCGGCGTTT   
  
  
- TGAATTAAAA GAAACCACGT ATACTTTAGA ATTTTTCTTA ATTTTATTCT ATATTATGCA ATTGAGAAGT   
  
  
- TGTTGTACTA TATAAATTCT ATGGTTATAA ACATAAGTAG TATATTTTTA ACATCACAAC GTTTTATAAT   
  
  
- TTTAATAAAG TATTTATTTG TAATAATATT CTTGACTGAG GCGCCTAATA GTATACCATA TCTAATGTCT   
  
  
- CACCCACCCA AATTTATTAA AGGGGCTGTG TAATATGAAA ATCTTCTCGA TAATCGAGGT TTAATAAATT   
  
  
- TGTTACAAGA TTTCTCGTTT TTTTAGTTAC AAAACCTAAA ACTAGGCTGG CTCTCCTGAA CAGGCTGGTA   
  
  
- AGGTATAATT TTACAAGGAT CAAGACTAAT CGATTATGGT

+     STRE

| Site Name | Organism | Position | Strand | Matrix score. | sequence | function |
| --- | --- | --- | --- | --- | --- | --- |
| STRE | Arabidopsis thaliana | 121 | - | 5 | AGGGG |  |

>PlantCARE\_25378   
+ ATAAAGTTGT CGTGATTTCT TGCCCTTTAT TTTTTATTTA TATTAACATA TATTTTTAAA AAAAAATTAA   
  
  
+ AGACGTTCCT AACCGAAACT CATGTATTTT TTGATTATGA ATATGAATAT CCCCTCTATA TTAATCATGG   
  
  
+ AGCATTACAA CATGTTTTCG TAGCCATATG TCATCACGAG AATGATTTTT AGAATTGTTA GAAAAATAAA   
  
  
+ TTGATTCATA TAAACATATA CTATGTTTTT TATTAAACTA ACTATCAAAT TAATTAATAG TGTACAAAAA   
  
  
+ AATATTTTTT TCTTTCCTTA AATAAAAACT ACGGAATTAC CTAATATGGC TAACATATAT ATGACAATTA   
  
  
+ ATGATTATGA ATAATACATA TTTGATAAAA AAATTTCTAA CCTCTCTCTT TTTTGTTTAA TTTTATATTA   
  
  
+ TTAAAGGAAA TTTAACAATC ACATTAATCA TATAATAAAA ACAATTAGAT TTTTTCTTAT ATGTTATATT   
  
  
+ TTGAATTTTT AAAAACGACT ATAAATTACT AAAAATGATA AGAGTCCCAC ATTAAAAAAT TTGTGATCAA   
  
  
+ CCGTTTAACT TTTTTTTTAG TTCAAGCAAG ATACAAATGA TCATATATCT GATATAGACG TGGGCGTTCG   
  
  
+ GATACACGTT CGGGTTTGTA TCAGATATTT CAGTATAAAG GTATAGAACC CGTTCGGGTA TTTCTACACT   
  
  
+ CCGAGTCGGG TTCGGGTTCG GATATTTTGG ATCGGGTTCG AATATTTAAA TTTTGAAGAA AAAAAGAAAT   
  
  
+ TATTCACTGT TTAAGTTTTT TATATTTAAA TATATCTTAA CTTAACTGAT TTTTTTTAGT TTTTAAAAGA   
  
  
+ TTAAAATATT AATATGTTTG GAGATAAAAC TTTAAAAATA GAAAGACACT AATTTAGTTT TTGTTTTGAA   
  
  
+ AATTTAGATG CAACTTTTGT TAATGCAAGA AACAAGAACT TGATATGTAT TTTAAGTGAG TAACAAATGA   
  
  
+ TTTTGTCTAT AGTTATATGT ATATTATCTA ATTTTGAGTA ATAAGAATCA TTAATATAAA TATTTTGAAT   
  
  
+ AAAATTAGAT AGATAAACTA TAAATATAGA GTTAAGTATA CTTATGTTTG GTTATCTTCG AATATTACCC   
  
  
+ GTTCGGATAT ATTATCTGAA CTGGTGAAAT AAGTAATATG TTTTGTTGTT TTAATTAGAT AATTTTTAGA   
  
  
+ CCGAGCTTGT GAATATATAC TAGACAAACA TTTATATTTC GAGTCTGCAC TTATATTCTA TAAGAGCTTG   
  
  
+ ATATATTAGA TTTGAACACT AACCTGTTAA TATAGTTTGC CGGTGATTTT TTTTCAAAAT TTTGATTCTT   
  
  
+ AGATATGTAT ATGGAGTAAA ACTAATTTTT ACAGATGCCC ATTTTTTTAA TTGACACTTA TGTAATTAAC   
  
  
+ TGAATTCATA AAACAAGGTT TTTTAAAAAA ATTTAACTCA TATCAATGAA ACAAAGACGA GAACGAAAGC   
  
  
+ ACAATTCTAT GGAAATGGAA AATGAAGTCA CTTATGGAGA TTCAATAGTA AGCAAATCGA GAGCAGAAAA   
  
  
+ TCTAATCTCC TTTCGTCATT ATACAATCAA TATTGCCTAT TTGGTTTTAG TGATTTGTTT CAGCCGCAAA   
  
  
+ ACTTAATTTT CTTTGGTGCA TATGAAATCT TAAAAAGAAT TAAAATAAGA TATAATACGT TAACTCTTCA   
  
  
+ ACAACATGAT ATATTTAAGA TACCAATATT TGTATTCATC ATATAAAAAT TGTAGTGTTG CAAAATATTA   
  
  
+ AAATTATTTC ATAAATAAAC ATTATTATAA GAACTGACTC CGCGGATTAT CATATGGTAT AGATTACAGA   
  
  
+ GTGGGTGGGT TTAAATAATT TCCCCGACAC ATTATACTTT TAGAAGAGCT ATTAGCTCCA AATTATTTAA   
  
  
+ ACAATGTTCT AAAGAGCAAA AAAATCAATG TTTTGGATTT TGATCCGACC GAGAGGACTT GTCCGACCAT   
  
  
+ TCCATATTAA AATGTTCCTA GTTCTGATTA GCTAATACCA   

- TATTTCAACA GCACTAAAGA ACGGGAAATA AAAAATAAAT ATAATTGTAT ATAAAAATTT TTTTTTAATT   
  
  
- TCTGCAAGGA TTGGCTTTGA GTACATAAAA AACTAATACT TATACTTATA GGGGAGATAT AATTAGTACC   
  
  
- TCGTAATGTT GTACAAAAGC ATCGGTATAC AGTAGTGCTC TTACTAAAAA TCTTAACAAT CTTTTTATTT   
  
  
- AACTAAGTAT ATTTGTATAT GATACAAAAA ATAATTTGAT TGATAGTTTA ATTAATTATC ACATGTTTTT   
  
  
- TTATAAAAAA AGAAAGGAAT TTATTTTTGA TGCCTTAATG GATTATACCG ATTGTATATA TACTGTTAAT   
  
  
- TACTAATACT TATTATGTAT AAACTATTTT TTTAAAGATT GGAGAGAGAA AAAACAAATT AAAATATAAT   
  
  
- AATTTCCTTT AAATTGTTAG TGTAATTAGT ATATTATTTT TGTTAATCTA AAAAAGAATA TACAATATAA   
  
  
- AACTTAAAAA TTTTTGCTGA TATTTAATGA TTTTTACTAT TCTCAGGGTG TAATTTTTTA AACACTAGTT   
  
  
- GGCAAATTGA AAAAAAAATC AAGTTCGTTC TATGTTTACT AGTATATAGA CTATATCTGC ACCCGCAAGC   
  
  
- CTATGTGCAA GCCCAAACAT AGTCTATAAA GTCATATTTC CATATCTTGG GCAAGCCCAT AAAGATGTGA   
  
  
- GGCTCAGCCC AAGCCCAAGC CTATAAAACC TAGCCCAAGC TTATAAATTT AAAACTTCTT TTTTTCTTTA   
  
  
- ATAAGTGACA AATTCAAAAA ATATAAATTT ATATAGAATT GAATTGACTA AAAAAAATCA AAAATTTTCT   
  
  
- AATTTTATAA TTATACAAAC CTCTATTTTG AAATTTTTAT CTTTCTGTGA TTAAATCAAA AACAAAACTT   
  
  
- TTAAATCTAC GTTGAAAACA ATTACGTTCT TTGTTCTTGA ACTATACATA AAATTCACTC ATTGTTTACT   
  
  
- AAAACAGATA TCAATATACA TATAATAGAT TAAAACTCAT TATTCTTAGT AATTATATTT ATAAAACTTA   
  
  
- TTTTAATCTA TCTATTTGAT ATTTATATCT CAATTCATAT GAATACAAAC CAATAGAAGC TTATAATGGG   
  
  
- CAAGCCTATA TAATAGACTT GACCACTTTA TTCATTATAC AAAACAACAA AATTAATCTA TTAAAAATCT   
  
  
- GGCTCGAACA CTTATATATG ATCTGTTTGT AAATATAAAG CTCAGACGTG AATATAAGAT ATTCTCGAAC   
  
  
- TATATAATCT AAACTTGTGA TTGGACAATT ATATCAAACG GCCACTAAAA AAAAGTTTTA AAACTAAGAA   
  
  
- TCTATACATA TACCTCATTT TGATTAAAAA TGTCTACGGG TAAAAAAATT AACTGTGAAT ACATTAATTG   
  
  
- ACTTAAGTAT TTTGTTCCAA AAAATTTTTT TAAATTGAGT ATAGTTACTT TGTTTCTGCT CTTGCTTTCG   
  
  
- TGTTAAGATA CCTTTACCTT TTACTTCAGT GAATACCTCT AAGTTATCAT TCGTTTAGCT CTCGTCTTTT   
  
  
- AGATTAGAGG AAAGCAGTAA TATGTTAGTT ATAACGGATA AACCAAAATC ACTAAACAAA GTCGGCGTTT   
  
  
- TGAATTAAAA GAAACCACGT ATACTTTAGA ATTTTTCTTA ATTTTATTCT ATATTATGCA ATTGAGAAGT   
  
  
- TGTTGTACTA TATAAATTCT ATGGTTATAA ACATAAGTAG TATATTTTTA ACATCACAAC GTTTTATAAT   
  
  
- TTTAATAAAG TATTTATTTG TAATAATATT CTTGACTGAG GCGCCTAATA GTATACCATA TCTAATGTCT   
  
  
- CACCCACCCA AATTTATTAA AGGGGCTGTG TAATATGAAA ATCTTCTCGA TAATCGAGGT TTAATAAATT   
  
  
- TGTTACAAGA TTTCTCGTTT TTTTAGTTAC AAAACCTAAA ACTAGGCTGG CTCTCCTGAA CAGGCTGGTA   
  
  
- AGGTATAATT TTACAAGGAT CAAGACTAAT CGATTATGGT

+     TATA

| Site Name | Organism | Position | Strand | Matrix score. | sequence | function |
| --- | --- | --- | --- | --- | --- | --- |
| TATA | Arabidopsis thaliana | 410 | - | 8 | TATAAAAT |  |

>PlantCARE\_25378   
+ ATAAAGTTGT CGTGATTTCT TGCCCTTTAT TTTTTATTTA TATTAACATA TATTTTTAAA AAAAAATTAA   
  
  
+ AGACGTTCCT AACCGAAACT CATGTATTTT TTGATTATGA ATATGAATAT CCCCTCTATA TTAATCATGG   
  
  
+ AGCATTACAA CATGTTTTCG TAGCCATATG TCATCACGAG AATGATTTTT AGAATTGTTA GAAAAATAAA   
  
  
+ TTGATTCATA TAAACATATA CTATGTTTTT TATTAAACTA ACTATCAAAT TAATTAATAG TGTACAAAAA   
  
  
+ AATATTTTTT TCTTTCCTTA AATAAAAACT ACGGAATTAC CTAATATGGC TAACATATAT ATGACAATTA   
  
  
+ ATGATTATGA ATAATACATA TTTGATAAAA AAATTTCTAA CCTCTCTCTT TTTTGTTTAA TTTTATATTA   
  
  
+ TTAAAGGAAA TTTAACAATC ACATTAATCA TATAATAAAA ACAATTAGAT TTTTTCTTAT ATGTTATATT   
  
  
+ TTGAATTTTT AAAAACGACT ATAAATTACT AAAAATGATA AGAGTCCCAC ATTAAAAAAT TTGTGATCAA   
  
  
+ CCGTTTAACT TTTTTTTTAG TTCAAGCAAG ATACAAATGA TCATATATCT GATATAGACG TGGGCGTTCG   
  
  
+ GATACACGTT CGGGTTTGTA TCAGATATTT CAGTATAAAG GTATAGAACC CGTTCGGGTA TTTCTACACT   
  
  
+ CCGAGTCGGG TTCGGGTTCG GATATTTTGG ATCGGGTTCG AATATTTAAA TTTTGAAGAA AAAAAGAAAT   
  
  
+ TATTCACTGT TTAAGTTTTT TATATTTAAA TATATCTTAA CTTAACTGAT TTTTTTTAGT TTTTAAAAGA   
  
  
+ TTAAAATATT AATATGTTTG GAGATAAAAC TTTAAAAATA GAAAGACACT AATTTAGTTT TTGTTTTGAA   
  
  
+ AATTTAGATG CAACTTTTGT TAATGCAAGA AACAAGAACT TGATATGTAT TTTAAGTGAG TAACAAATGA   
  
  
+ TTTTGTCTAT AGTTATATGT ATATTATCTA ATTTTGAGTA ATAAGAATCA TTAATATAAA TATTTTGAAT   
  
  
+ AAAATTAGAT AGATAAACTA TAAATATAGA GTTAAGTATA CTTATGTTTG GTTATCTTCG AATATTACCC   
  
  
+ GTTCGGATAT ATTATCTGAA CTGGTGAAAT AAGTAATATG TTTTGTTGTT TTAATTAGAT AATTTTTAGA   
  
  
+ CCGAGCTTGT GAATATATAC TAGACAAACA TTTATATTTC GAGTCTGCAC TTATATTCTA TAAGAGCTTG   
  
  
+ ATATATTAGA TTTGAACACT AACCTGTTAA TATAGTTTGC CGGTGATTTT TTTTCAAAAT TTTGATTCTT   
  
  
+ AGATATGTAT ATGGAGTAAA ACTAATTTTT ACAGATGCCC ATTTTTTTAA TTGACACTTA TGTAATTAAC   
  
  
+ TGAATTCATA AAACAAGGTT TTTTAAAAAA ATTTAACTCA TATCAATGAA ACAAAGACGA GAACGAAAGC   
  
  
+ ACAATTCTAT GGAAATGGAA AATGAAGTCA CTTATGGAGA TTCAATAGTA AGCAAATCGA GAGCAGAAAA   
  
  
+ TCTAATCTCC TTTCGTCATT ATACAATCAA TATTGCCTAT TTGGTTTTAG TGATTTGTTT CAGCCGCAAA   
  
  
+ ACTTAATTTT CTTTGGTGCA TATGAAATCT TAAAAAGAAT TAAAATAAGA TATAATACGT TAACTCTTCA   
  
  
+ ACAACATGAT ATATTTAAGA TACCAATATT TGTATTCATC ATATAAAAAT TGTAGTGTTG CAAAATATTA   
  
  
+ AAATTATTTC ATAAATAAAC ATTATTATAA GAACTGACTC CGCGGATTAT CATATGGTAT AGATTACAGA   
  
  
+ GTGGGTGGGT TTAAATAATT TCCCCGACAC ATTATACTTT TAGAAGAGCT ATTAGCTCCA AATTATTTAA   
  
  
+ ACAATGTTCT AAAGAGCAAA AAAATCAATG TTTTGGATTT TGATCCGACC GAGAGGACTT GTCCGACCAT   
  
  
+ TCCATATTAA AATGTTCCTA GTTCTGATTA GCTAATACCA   

- TATTTCAACA GCACTAAAGA ACGGGAAATA AAAAATAAAT ATAATTGTAT ATAAAAATTT TTTTTTAATT   
  
  
- TCTGCAAGGA TTGGCTTTGA GTACATAAAA AACTAATACT TATACTTATA GGGGAGATAT AATTAGTACC   
  
  
- TCGTAATGTT GTACAAAAGC ATCGGTATAC AGTAGTGCTC TTACTAAAAA TCTTAACAAT CTTTTTATTT   
  
  
- AACTAAGTAT ATTTGTATAT GATACAAAAA ATAATTTGAT TGATAGTTTA ATTAATTATC ACATGTTTTT   
  
  
- TTATAAAAAA AGAAAGGAAT TTATTTTTGA TGCCTTAATG GATTATACCG ATTGTATATA TACTGTTAAT   
  
  
- TACTAATACT TATTATGTAT AAACTATTTT TTTAAAGATT GGAGAGAGAA AAAACAAATT AAAATATAAT   
  
  
- AATTTCCTTT AAATTGTTAG TGTAATTAGT ATATTATTTT TGTTAATCTA AAAAAGAATA TACAATATAA   
  
  
- AACTTAAAAA TTTTTGCTGA TATTTAATGA TTTTTACTAT TCTCAGGGTG TAATTTTTTA AACACTAGTT   
  
  
- GGCAAATTGA AAAAAAAATC AAGTTCGTTC TATGTTTACT AGTATATAGA CTATATCTGC ACCCGCAAGC   
  
  
- CTATGTGCAA GCCCAAACAT AGTCTATAAA GTCATATTTC CATATCTTGG GCAAGCCCAT AAAGATGTGA   
  
  
- GGCTCAGCCC AAGCCCAAGC CTATAAAACC TAGCCCAAGC TTATAAATTT AAAACTTCTT TTTTTCTTTA   
  
  
- ATAAGTGACA AATTCAAAAA ATATAAATTT ATATAGAATT GAATTGACTA AAAAAAATCA AAAATTTTCT   
  
  
- AATTTTATAA TTATACAAAC CTCTATTTTG AAATTTTTAT CTTTCTGTGA TTAAATCAAA AACAAAACTT   
  
  
- TTAAATCTAC GTTGAAAACA ATTACGTTCT TTGTTCTTGA ACTATACATA AAATTCACTC ATTGTTTACT   
  
  
- AAAACAGATA TCAATATACA TATAATAGAT TAAAACTCAT TATTCTTAGT AATTATATTT ATAAAACTTA   
  
  
- TTTTAATCTA TCTATTTGAT ATTTATATCT CAATTCATAT GAATACAAAC CAATAGAAGC TTATAATGGG   
  
  
- CAAGCCTATA TAATAGACTT GACCACTTTA TTCATTATAC AAAACAACAA AATTAATCTA TTAAAAATCT   
  
  
- GGCTCGAACA CTTATATATG ATCTGTTTGT AAATATAAAG CTCAGACGTG AATATAAGAT ATTCTCGAAC   
  
  
- TATATAATCT AAACTTGTGA TTGGACAATT ATATCAAACG GCCACTAAAA AAAAGTTTTA AAACTAAGAA   
  
  
- TCTATACATA TACCTCATTT TGATTAAAAA TGTCTACGGG TAAAAAAATT AACTGTGAAT ACATTAATTG   
  
  
- ACTTAAGTAT TTTGTTCCAA AAAATTTTTT TAAATTGAGT ATAGTTACTT TGTTTCTGCT CTTGCTTTCG   
  
  
- TGTTAAGATA CCTTTACCTT TTACTTCAGT GAATACCTCT AAGTTATCAT TCGTTTAGCT CTCGTCTTTT   
  
  
- AGATTAGAGG AAAGCAGTAA TATGTTAGTT ATAACGGATA AACCAAAATC ACTAAACAAA GTCGGCGTTT   
  
  
- TGAATTAAAA GAAACCACGT ATACTTTAGA ATTTTTCTTA ATTTTATTCT ATATTATGCA ATTGAGAAGT   
  
  
- TGTTGTACTA TATAAATTCT ATGGTTATAA ACATAAGTAG TATATTTTTA ACATCACAAC GTTTTATAAT   
  
  
- TTTAATAAAG TATTTATTTG TAATAATATT CTTGACTGAG GCGCCTAATA GTATACCATA TCTAATGTCT   
  
  
- CACCCACCCA AATTTATTAA AGGGGCTGTG TAATATGAAA ATCTTCTCGA TAATCGAGGT TTAATAAATT   
  
  
- TGTTACAAGA TTTCTCGTTT TTTTAGTTAC AAAACCTAAA ACTAGGCTGG CTCTCCTGAA CAGGCTGGTA   
  
  
- AGGTATAATT TTACAAGGAT CAAGACTAAT CGATTATGGT

+     TATA-box

| Site Name | Organism | Position | Strand | Matrix score. | sequence | function |
| --- | --- | --- | --- | --- | --- | --- |
| TATA-box | Arabidopsis thaliana | 1559 | - | 5 | TATAA | core promoter element around -30 of transcription start |
| TATA-box | Arabidopsis thaliana | 1722 | - | 4 | TATA | core promoter element around -30 of transcription start |
| TATA-box | Arabidopsis thaliana | 1560 | - | 4 | TATA | core promoter element around -30 of transcription start |
| TATA-box | Brassica napus | 1851 | + | 6 | ATTATA | core promoter element around -30 of transcription start |
| TATA-box | Arabidopsis thaliana | 1338 | - | 4 | TATA | core promoter element around -30 of transcription start |
| TATA-box | Arabidopsis thaliana | 1808 | - | 4 | TATA | core promoter element around -30 of transcription start |
| TATA-box | Arabidopsis thaliana | 1720 | + | 9 | ccTATAAAaa | core promoter element around -30 of transcription start |
| TATA-box | Arabidopsis thaliana | 1853 | - | 4 | TATA | core promoter element around -30 of transcription start |
| TATA-box | Daucus carota | 35 | - | 8 | TATAAATA | core promoter element around -30 of transcription start |
| TATA-box | Arabidopsis thaliana | 1775 | - | 5 | TATAA | core promoter element around -30 of transcription start |
| TATA-box | Brassica oleracea | 1660 | + | 6 | ATATAA | core promoter element around -30 of transcription start |
| TATA-box | Arabidopsis thaliana | 1852 | - | 5 | TATAA | core promoter element around -30 of transcription start |
| TATA-box | Brassica napus | 1558 | + | 6 | ATTATA | core promoter element around -30 of transcription start |
| TATA-box | Arabidopsis thaliana | 1830 | - | 8 | TATTTAAA | core promoter element around -30 of transcription start |
| TATA-box | Brassica juncea | 36 | - | 7 | TATAAAT | core promoter element around -30 of transcription start |
| TATA-box | Arabidopsis thaliana | 1884 | + | 8 | TATTTAAA | core promoter element around -30 of transcription start |
| TATA-box | Helianthus annuus | 37 | - | 6 | TATAAA | core promoter element around -30 of transcription start |
| TATA-box | Brassica napus | 1774 | + | 6 | ATTATA | core promoter element around -30 of transcription start |
| TATA-box | Arabidopsis thaliana | 1661 | - | 4 | TATA | core promoter element around -30 of transcription start |
| TATA-box | Arabidopsis thaliana | 1776 | - | 4 | TATA | core promoter element around -30 of transcription start |
| TATA-box | Arabidopsis thaliana | 38 | - | 5 | TATAA | core promoter element around -30 of transcription start |
| TATA-box | Brassica oleracea | 1721 | + | 6 | ATATAA | core promoter element around -30 of transcription start |
| TATA-box | Arabidopsis thaliana | 39 | + | 4 | TATA | core promoter element around -30 of transcription start |
| TATA-box | Brassica napus | 1689 | - | 6 | ATATAT | core promoter element around -30 of transcription start |
| TATA-box | Brassica napus | 48 | + | 6 | ATATAT | core promoter element around -30 of transcription start |
| TATA-box | Arabidopsis thaliana | 1690 | - | 4 | TATA | core promoter element around -30 of transcription start |
| TATA-box | Arabidopsis thaliana | 49 | + | 4 | TATA | core promoter element around -30 of transcription start |
| TATA-box | Arabidopsis thaliana | 127 | + | 4 | TATA | core promoter element around -30 of transcription start |
| TATA-box | Arabidopsis thaliana | 213 | - | 9 | taTATAAAtc | core promoter element around -30 of transcription start |
| TATA-box | Brassica oleracea | 218 | + | 6 | ATATAA | core promoter element around -30 of transcription start |
| TATA-box | Arabidopsis thaliana | 219 | + | 4 | TATA | core promoter element around -30 of transcription start |
| TATA-box | Arabidopsis thaliana | 227 | + | 4 | TATA | core promoter element around -30 of transcription start |
| TATA-box | Oryza sativa | 273 | + | 7 | TACAAAA | core promoter element around -30 of transcription start |
| TATA-box | Brassica napus | 335 | + | 6 | ATATAT | core promoter element around -30 of transcription start |
| TATA-box | Arabidopsis thaliana | 336 | + | 6 | TATATA | core promoter element around -30 of transcription start |
| TATA-box | Brassica napus | 337 | + | 6 | ATATAT | core promoter element around -30 of transcription start |
| TATA-box | Arabidopsis thaliana | 338 | + | 4 | TATA | core promoter element around -30 of transcription start |
| TATA-box | Pisum sativum | 411 | - | 7 | TATAAAA | core promoter element around -30 of transcription start |
| TATA-box | Helianthus annuus | 412 | - | 6 | TATAAA | core promoter element around -30 of transcription start |
| TATA-box | Arabidopsis thaliana | 413 | - | 5 | TATAA | core promoter element around -30 of transcription start |
| TATA-box | Arabidopsis thaliana | 414 | + | 4 | TATA | core promoter element around -30 of transcription start |
| TATA-box | Brassica oleracea | 450 | + | 6 | ATATAA | core promoter element around -30 of transcription start |
| TATA-box | Arabidopsis thaliana | 451 | + | 4 | TATA | core promoter element around -30 of transcription start |
| TATA-box | Zea mays | 474 | - | 8 | TATAAGAA | core promoter element around -30 of transcription start |
| TATA-box | Arabidopsis thaliana | 477 | - | 5 | TATAA | core promoter element around -30 of transcription start |
| TATA-box | Arabidopsis thaliana | 478 | + | 4 | TATA | core promoter element around -30 of transcription start |
| TATA-box | Arabidopsis thaliana | 484 | - | 5 | TATAA | core promoter element around -30 of transcription start |
| TATA-box | Arabidopsis thaliana | 485 | + | 4 | TATA | core promoter element around -30 of transcription start |
| TATA-box | Arabidopsis thaliana | 510 | + | 4 | TATA | core promoter element around -30 of transcription start |
| TATA-box | Brassica napus | 603 | + | 6 | ATATAT | core promoter element around -30 of transcription start |
| TATA-box | Arabidopsis thaliana | 604 | + | 4 | TATA | core promoter element around -30 of transcription start |
| TATA-box | Arabidopsis thaliana | 613 | + | 4 | TATA | core promoter element around -30 of transcription start |
| TATA-box | Arabidopsis thaliana | 664 | + | 4 | TATA | core promoter element around -30 of transcription start |
| TATA-box | Arabidopsis thaliana | 672 | + | 4 | TATA | core promoter element around -30 of transcription start |
| TATA-box | Arabidopsis thaliana | 743 | + | 8 | TATTTAAA | core promoter element around -30 of transcription start |
| TATA-box | Pisum sativum | 788 | - | 7 | TATAAAA | core promoter element around -30 of transcription start |
| TATA-box | Helianthus annuus | 789 | - | 6 | TATAAA | core promoter element around -30 of transcription start |
| TATA-box | Arabidopsis thaliana | 790 | - | 5 | TATAA | core promoter element around -30 of transcription start |
| TATA-box | Arabidopsis thaliana | 791 | + | 4 | TATA | core promoter element around -30 of transcription start |
| TATA-box | Arabidopsis thaliana | 793 | + | 8 | TATTTAAA | core promoter element around -30 of transcription start |
| TATA-box | Arabidopsis thaliana | 795 | - | 8 | TATTTAAA | core promoter element around -30 of transcription start |
| TATA-box | Brassica napus | 800 | + | 6 | ATATAT | core promoter element around -30 of transcription start |
| TATA-box | Arabidopsis thaliana | 801 | + | 4 | TATA | core promoter element around -30 of transcription start |
| TATA-box | Arabidopsis thaliana | 988 | + | 4 | TATA | core promoter element around -30 of transcription start |
| TATA-box | Arabidopsis thaliana | 993 | - | 5 | TATAA | core promoter element around -30 of transcription start |
| TATA-box | Arabidopsis thaliana | 994 | + | 4 | TATA | core promoter element around -30 of transcription start |
| TATA-box | Helianthus annuus | 998 | - | 6 | TATACA | core promoter element around -30 of transcription start |
| TATA-box | Arabidopsis thaliana | 1000 | - | 4 | TATA | core promoter element around -30 of transcription start |
| TATA-box | Brassica oleracea | 1034 | + | 6 | ATATAA | core promoter element around -30 of transcription start |
| TATA-box | Arabidopsis thaliana | 1035 | - | 4 | TATA | core promoter element around -30 of transcription start |
| TATA-box | Avena sativa | 1066 | - | 12 | TATATTTATATTT | core promoter element around -30 of transcription start |
| TATA-box | Arabidopsis thaliana | 1069 | - | 4 | TATA | core promoter element around -30 of transcription start |
| TATA-box | Arabidopsis thaliana | 1075 | - | 4 | TATA | core promoter element around -30 of transcription start |
| TATA-box | Arabidopsis thaliana | 1087 | - | 4 | TATA | core promoter element around -30 of transcription start |
| TATA-box | Brassica napus | 1127 | - | 6 | ATATAT | core promoter element around -30 of transcription start |
| TATA-box | Arabidopsis thaliana | 1128 | - | 4 | TATA | core promoter element around -30 of transcription start |
| TATA-box | Brassica napus | 1203 | - | 6 | ATATAT | core promoter element around -30 of transcription start |
| TATA-box | Arabidopsis thaliana | 1204 | - | 6 | TATATA | core promoter element around -30 of transcription start |
| TATA-box | Arabidopsis thaliana | 1206 | - | 4 | TATA | core promoter element around -30 of transcription start |
| TATA-box | Brassica juncea | 1220 | - | 7 | TATAAAT | core promoter element around -30 of transcription start |
| TATA-box | Helianthus annuus | 1221 | - | 6 | TATAAA | core promoter element around -30 of transcription start |
| TATA-box | Arabidopsis thaliana | 1222 | - | 5 | TATAA | core promoter element around -30 of transcription start |
| TATA-box | Arabidopsis thaliana | 1223 | - | 4 | TATA | core promoter element around -30 of transcription start |
| TATA-box | Arabidopsis thaliana | 1241 | - | 5 | TATAA | core promoter element around -30 of transcription start |
| TATA-box | Arabidopsis thaliana | 1242 | - | 4 | TATA | core promoter element around -30 of transcription start |
| TATA-box | Arabidopsis thaliana | 1249 | - | 4 | TATA | core promoter element around -30 of transcription start |
| TATA-box | Brassica napus | 1261 | - | 6 | ATATAT | core promoter element around -30 of transcription start |
| TATA-box | Arabidopsis thaliana | 1262 | - | 4 | TATA | core promoter element around -30 of transcription start |
| TATA-box | Arabidopsis thaliana | 1291 | - | 4 | TATA | core promoter element around -30 of transcription start |
| TATA-box | Helianthus annuus | 1336 | - | 6 | TATACA | core promoter element around -30 of transcription start |

>PlantCARE\_25378   
+ ATAAAGTTGT CGTGATTTCT TGCCCTTTAT TTTTTATTTA TATTAACATA TATTTTTAAA AAAAAATTAA   
  
  
+ AGACGTTCCT AACCGAAACT CATGTATTTT TTGATTATGA ATATGAATAT CCCCTCTATA TTAATCATGG   
  
  
+ AGCATTACAA CATGTTTTCG TAGCCATATG TCATCACGAG AATGATTTTT AGAATTGTTA GAAAAATAAA   
  
  
+ TTGATTCATA TAAACATATA CTATGTTTTT TATTAAACTA ACTATCAAAT TAATTAATAG TGTACAAAAA   
  
  
+ AATATTTTTT TCTTTCCTTA AATAAAAACT ACGGAATTAC CTAATATGGC TAACATATAT ATGACAATTA   
  
  
+ ATGATTATGA ATAATACATA TTTGATAAAA AAATTTCTAA CCTCTCTCTT TTTTGTTTAA TTTTATATTA   
  
  
+ TTAAAGGAAA TTTAACAATC ACATTAATCA TATAATAAAA ACAATTAGAT TTTTTCTTAT ATGTTATATT   
  
  
+ TTGAATTTTT AAAAACGACT ATAAATTACT AAAAATGATA AGAGTCCCAC ATTAAAAAAT TTGTGATCAA   
  
  
+ CCGTTTAACT TTTTTTTTAG TTCAAGCAAG ATACAAATGA TCATATATCT GATATAGACG TGGGCGTTCG   
  
  
+ GATACACGTT CGGGTTTGTA TCAGATATTT CAGTATAAAG GTATAGAACC CGTTCGGGTA TTTCTACACT   
  
  
+ CCGAGTCGGG TTCGGGTTCG GATATTTTGG ATCGGGTTCG AATATTTAAA TTTTGAAGAA AAAAAGAAAT   
  
  
+ TATTCACTGT TTAAGTTTTT TATATTTAAA TATATCTTAA CTTAACTGAT TTTTTTTAGT TTTTAAAAGA   
  
  
+ TTAAAATATT AATATGTTTG GAGATAAAAC TTTAAAAATA GAAAGACACT AATTTAGTTT TTGTTTTGAA   
  
  
+ AATTTAGATG CAACTTTTGT TAATGCAAGA AACAAGAACT TGATATGTAT TTTAAGTGAG TAACAAATGA   
  
  
+ TTTTGTCTAT AGTTATATGT ATATTATCTA ATTTTGAGTA ATAAGAATCA TTAATATAAA TATTTTGAAT   
  
  
+ AAAATTAGAT AGATAAACTA TAAATATAGA GTTAAGTATA CTTATGTTTG GTTATCTTCG AATATTACCC   
  
  
+ GTTCGGATAT ATTATCTGAA CTGGTGAAAT AAGTAATATG TTTTGTTGTT TTAATTAGAT AATTTTTAGA   
  
  
+ CCGAGCTTGT GAATATATAC TAGACAAACA TTTATATTTC GAGTCTGCAC TTATATTCTA TAAGAGCTTG   
  
  
+ ATATATTAGA TTTGAACACT AACCTGTTAA TATAGTTTGC CGGTGATTTT TTTTCAAAAT TTTGATTCTT   
  
  
+ AGATATGTAT ATGGAGTAAA ACTAATTTTT ACAGATGCCC ATTTTTTTAA TTGACACTTA TGTAATTAAC   
  
  
+ TGAATTCATA AAACAAGGTT TTTTAAAAAA ATTTAACTCA TATCAATGAA ACAAAGACGA GAACGAAAGC   
  
  
+ ACAATTCTAT GGAAATGGAA AATGAAGTCA CTTATGGAGA TTCAATAGTA AGCAAATCGA GAGCAGAAAA   
  
  
+ TCTAATCTCC TTTCGTCATT ATACAATCAA TATTGCCTAT TTGGTTTTAG TGATTTGTTT CAGCCGCAAA   
  
  
+ ACTTAATTTT CTTTGGTGCA TATGAAATCT TAAAAAGAAT TAAAATAAGA TATAATACGT TAACTCTTCA   
  
  
+ ACAACATGAT ATATTTAAGA TACCAATATT TGTATTCATC ATATAAAAAT TGTAGTGTTG CAAAATATTA   
  
  
+ AAATTATTTC ATAAATAAAC ATTATTATAA GAACTGACTC CGCGGATTAT CATATGGTAT AGATTACAGA   
  
  
+ GTGGGTGGGT TTAAATAATT TCCCCGACAC ATTATACTTT TAGAAGAGCT ATTAGCTCCA AATTATTTAA   
  
  
+ ACAATGTTCT AAAGAGCAAA AAAATCAATG TTTTGGATTT TGATCCGACC GAGAGGACTT GTCCGACCAT   
  
  
+ TCCATATTAA AATGTTCCTA GTTCTGATTA GCTAATACCA   

- TATTTCAACA GCACTAAAGA ACGGGAAATA AAAAATAAAT ATAATTGTAT ATAAAAATTT TTTTTTAATT   
  
  
- TCTGCAAGGA TTGGCTTTGA GTACATAAAA AACTAATACT TATACTTATA GGGGAGATAT AATTAGTACC   
  
  
- TCGTAATGTT GTACAAAAGC ATCGGTATAC AGTAGTGCTC TTACTAAAAA TCTTAACAAT CTTTTTATTT   
  
  
- AACTAAGTAT ATTTGTATAT GATACAAAAA ATAATTTGAT TGATAGTTTA ATTAATTATC ACATGTTTTT   
  
  
- TTATAAAAAA AGAAAGGAAT TTATTTTTGA TGCCTTAATG GATTATACCG ATTGTATATA TACTGTTAAT   
  
  
- TACTAATACT TATTATGTAT AAACTATTTT TTTAAAGATT GGAGAGAGAA AAAACAAATT AAAATATAAT   
  
  
- AATTTCCTTT AAATTGTTAG TGTAATTAGT ATATTATTTT TGTTAATCTA AAAAAGAATA TACAATATAA   
  
  
- AACTTAAAAA TTTTTGCTGA TATTTAATGA TTTTTACTAT TCTCAGGGTG TAATTTTTTA AACACTAGTT   
  
  
- GGCAAATTGA AAAAAAAATC AAGTTCGTTC TATGTTTACT AGTATATAGA CTATATCTGC ACCCGCAAGC   
  
  
- CTATGTGCAA GCCCAAACAT AGTCTATAAA GTCATATTTC CATATCTTGG GCAAGCCCAT AAAGATGTGA   
  
  
- GGCTCAGCCC AAGCCCAAGC CTATAAAACC TAGCCCAAGC TTATAAATTT AAAACTTCTT TTTTTCTTTA   
  
  
- ATAAGTGACA AATTCAAAAA ATATAAATTT ATATAGAATT GAATTGACTA AAAAAAATCA AAAATTTTCT   
  
  
- AATTTTATAA TTATACAAAC CTCTATTTTG AAATTTTTAT CTTTCTGTGA TTAAATCAAA AACAAAACTT   
  
  
- TTAAATCTAC GTTGAAAACA ATTACGTTCT TTGTTCTTGA ACTATACATA AAATTCACTC ATTGTTTACT   
  
  
- AAAACAGATA TCAATATACA TATAATAGAT TAAAACTCAT TATTCTTAGT AATTATATTT ATAAAACTTA   
  
  
- TTTTAATCTA TCTATTTGAT ATTTATATCT CAATTCATAT GAATACAAAC CAATAGAAGC TTATAATGGG   
  
  
- CAAGCCTATA TAATAGACTT GACCACTTTA TTCATTATAC AAAACAACAA AATTAATCTA TTAAAAATCT   
  
  
- GGCTCGAACA CTTATATATG ATCTGTTTGT AAATATAAAG CTCAGACGTG AATATAAGAT ATTCTCGAAC   
  
  
- TATATAATCT AAACTTGTGA TTGGACAATT ATATCAAACG GCCACTAAAA AAAAGTTTTA AAACTAAGAA   
  
  
- TCTATACATA TACCTCATTT TGATTAAAAA TGTCTACGGG TAAAAAAATT AACTGTGAAT ACATTAATTG   
  
  
- ACTTAAGTAT TTTGTTCCAA AAAATTTTTT TAAATTGAGT ATAGTTACTT TGTTTCTGCT CTTGCTTTCG   
  
  
- TGTTAAGATA CCTTTACCTT TTACTTCAGT GAATACCTCT AAGTTATCAT TCGTTTAGCT CTCGTCTTTT   
  
  
- AGATTAGAGG AAAGCAGTAA TATGTTAGTT ATAACGGATA AACCAAAATC ACTAAACAAA GTCGGCGTTT   
  
  
- TGAATTAAAA GAAACCACGT ATACTTTAGA ATTTTTCTTA ATTTTATTCT ATATTATGCA ATTGAGAAGT   
  
  
- TGTTGTACTA TATAAATTCT ATGGTTATAA ACATAAGTAG TATATTTTTA ACATCACAAC GTTTTATAAT   
  
  
- TTTAATAAAG TATTTATTTG TAATAATATT CTTGACTGAG GCGCCTAATA GTATACCATA TCTAATGTCT   
  
  
- CACCCACCCA AATTTATTAA AGGGGCTGTG TAATATGAAA ATCTTCTCGA TAATCGAGGT TTAATAAATT   
  
  
- TGTTACAAGA TTTCTCGTTT TTTTAGTTAC AAAACCTAAA ACTAGGCTGG CTCTCCTGAA CAGGCTGGTA   
  
  
- AGGTATAATT TTACAAGGAT CAAGACTAAT CGATTATGGT

+     TCA

| Site Name | Organism | Position | Strand | Matrix score. | sequence | function |
| --- | --- | --- | --- | --- | --- | --- |
| TCA | Pisum sativum | 107 | - | 9 | TCATCTTCAT |  |

>PlantCARE\_25378   
+ ATAAAGTTGT CGTGATTTCT TGCCCTTTAT TTTTTATTTA TATTAACATA TATTTTTAAA AAAAAATTAA   
  
  
+ AGACGTTCCT AACCGAAACT CATGTATTTT TTGATTATGA ATATGAATAT CCCCTCTATA TTAATCATGG   
  
  
+ AGCATTACAA CATGTTTTCG TAGCCATATG TCATCACGAG AATGATTTTT AGAATTGTTA GAAAAATAAA   
  
  
+ TTGATTCATA TAAACATATA CTATGTTTTT TATTAAACTA ACTATCAAAT TAATTAATAG TGTACAAAAA   
  
  
+ AATATTTTTT TCTTTCCTTA AATAAAAACT ACGGAATTAC CTAATATGGC TAACATATAT ATGACAATTA   
  
  
+ ATGATTATGA ATAATACATA TTTGATAAAA AAATTTCTAA CCTCTCTCTT TTTTGTTTAA TTTTATATTA   
  
  
+ TTAAAGGAAA TTTAACAATC ACATTAATCA TATAATAAAA ACAATTAGAT TTTTTCTTAT ATGTTATATT   
  
  
+ TTGAATTTTT AAAAACGACT ATAAATTACT AAAAATGATA AGAGTCCCAC ATTAAAAAAT TTGTGATCAA   
  
  
+ CCGTTTAACT TTTTTTTTAG TTCAAGCAAG ATACAAATGA TCATATATCT GATATAGACG TGGGCGTTCG   
  
  
+ GATACACGTT CGGGTTTGTA TCAGATATTT CAGTATAAAG GTATAGAACC CGTTCGGGTA TTTCTACACT   
  
  
+ CCGAGTCGGG TTCGGGTTCG GATATTTTGG ATCGGGTTCG AATATTTAAA TTTTGAAGAA AAAAAGAAAT   
  
  
+ TATTCACTGT TTAAGTTTTT TATATTTAAA TATATCTTAA CTTAACTGAT TTTTTTTAGT TTTTAAAAGA   
  
  
+ TTAAAATATT AATATGTTTG GAGATAAAAC TTTAAAAATA GAAAGACACT AATTTAGTTT TTGTTTTGAA   
  
  
+ AATTTAGATG CAACTTTTGT TAATGCAAGA AACAAGAACT TGATATGTAT TTTAAGTGAG TAACAAATGA   
  
  
+ TTTTGTCTAT AGTTATATGT ATATTATCTA ATTTTGAGTA ATAAGAATCA TTAATATAAA TATTTTGAAT   
  
  
+ AAAATTAGAT AGATAAACTA TAAATATAGA GTTAAGTATA CTTATGTTTG GTTATCTTCG AATATTACCC   
  
  
+ GTTCGGATAT ATTATCTGAA CTGGTGAAAT AAGTAATATG TTTTGTTGTT TTAATTAGAT AATTTTTAGA   
  
  
+ CCGAGCTTGT GAATATATAC TAGACAAACA TTTATATTTC GAGTCTGCAC TTATATTCTA TAAGAGCTTG   
  
  
+ ATATATTAGA TTTGAACACT AACCTGTTAA TATAGTTTGC CGGTGATTTT TTTTCAAAAT TTTGATTCTT   
  
  
+ AGATATGTAT ATGGAGTAAA ACTAATTTTT ACAGATGCCC ATTTTTTTAA TTGACACTTA TGTAATTAAC   
  
  
+ TGAATTCATA AAACAAGGTT TTTTAAAAAA ATTTAACTCA TATCAATGAA ACAAAGACGA GAACGAAAGC   
  
  
+ ACAATTCTAT GGAAATGGAA AATGAAGTCA CTTATGGAGA TTCAATAGTA AGCAAATCGA GAGCAGAAAA   
  
  
+ TCTAATCTCC TTTCGTCATT ATACAATCAA TATTGCCTAT TTGGTTTTAG TGATTTGTTT CAGCCGCAAA   
  
  
+ ACTTAATTTT CTTTGGTGCA TATGAAATCT TAAAAAGAAT TAAAATAAGA TATAATACGT TAACTCTTCA   
  
  
+ ACAACATGAT ATATTTAAGA TACCAATATT TGTATTCATC ATATAAAAAT TGTAGTGTTG CAAAATATTA   
  
  
+ AAATTATTTC ATAAATAAAC ATTATTATAA GAACTGACTC CGCGGATTAT CATATGGTAT AGATTACAGA   
  
  
+ GTGGGTGGGT TTAAATAATT TCCCCGACAC ATTATACTTT TAGAAGAGCT ATTAGCTCCA AATTATTTAA   
  
  
+ ACAATGTTCT AAAGAGCAAA AAAATCAATG TTTTGGATTT TGATCCGACC GAGAGGACTT GTCCGACCAT   
  
  
+ TCCATATTAA AATGTTCCTA GTTCTGATTA GCTAATACCA   

- TATTTCAACA GCACTAAAGA ACGGGAAATA AAAAATAAAT ATAATTGTAT ATAAAAATTT TTTTTTAATT   
  
  
- TCTGCAAGGA TTGGCTTTGA GTACATAAAA AACTAATACT TATACTTATA GGGGAGATAT AATTAGTACC   
  
  
- TCGTAATGTT GTACAAAAGC ATCGGTATAC AGTAGTGCTC TTACTAAAAA TCTTAACAAT CTTTTTATTT   
  
  
- AACTAAGTAT ATTTGTATAT GATACAAAAA ATAATTTGAT TGATAGTTTA ATTAATTATC ACATGTTTTT   
  
  
- TTATAAAAAA AGAAAGGAAT TTATTTTTGA TGCCTTAATG GATTATACCG ATTGTATATA TACTGTTAAT   
  
  
- TACTAATACT TATTATGTAT AAACTATTTT TTTAAAGATT GGAGAGAGAA AAAACAAATT AAAATATAAT   
  
  
- AATTTCCTTT AAATTGTTAG TGTAATTAGT ATATTATTTT TGTTAATCTA AAAAAGAATA TACAATATAA   
  
  
- AACTTAAAAA TTTTTGCTGA TATTTAATGA TTTTTACTAT TCTCAGGGTG TAATTTTTTA AACACTAGTT   
  
  
- GGCAAATTGA AAAAAAAATC AAGTTCGTTC TATGTTTACT AGTATATAGA CTATATCTGC ACCCGCAAGC   
  
  
- CTATGTGCAA GCCCAAACAT AGTCTATAAA GTCATATTTC CATATCTTGG GCAAGCCCAT AAAGATGTGA   
  
  
- GGCTCAGCCC AAGCCCAAGC CTATAAAACC TAGCCCAAGC TTATAAATTT AAAACTTCTT TTTTTCTTTA   
  
  
- ATAAGTGACA AATTCAAAAA ATATAAATTT ATATAGAATT GAATTGACTA AAAAAAATCA AAAATTTTCT   
  
  
- AATTTTATAA TTATACAAAC CTCTATTTTG AAATTTTTAT CTTTCTGTGA TTAAATCAAA AACAAAACTT   
  
  
- TTAAATCTAC GTTGAAAACA ATTACGTTCT TTGTTCTTGA ACTATACATA AAATTCACTC ATTGTTTACT   
  
  
- AAAACAGATA TCAATATACA TATAATAGAT TAAAACTCAT TATTCTTAGT AATTATATTT ATAAAACTTA   
  
  
- TTTTAATCTA TCTATTTGAT ATTTATATCT CAATTCATAT GAATACAAAC CAATAGAAGC TTATAATGGG   
  
  
- CAAGCCTATA TAATAGACTT GACCACTTTA TTCATTATAC AAAACAACAA AATTAATCTA TTAAAAATCT   
  
  
- GGCTCGAACA CTTATATATG ATCTGTTTGT AAATATAAAG CTCAGACGTG AATATAAGAT ATTCTCGAAC   
  
  
- TATATAATCT AAACTTGTGA TTGGACAATT ATATCAAACG GCCACTAAAA AAAAGTTTTA AAACTAAGAA   
  
  
- TCTATACATA TACCTCATTT TGATTAAAAA TGTCTACGGG TAAAAAAATT AACTGTGAAT ACATTAATTG   
  
  
- ACTTAAGTAT TTTGTTCCAA AAAATTTTTT TAAATTGAGT ATAGTTACTT TGTTTCTGCT CTTGCTTTCG   
  
  
- TGTTAAGATA CCTTTACCTT TTACTTCAGT GAATACCTCT AAGTTATCAT TCGTTTAGCT CTCGTCTTTT   
  
  
- AGATTAGAGG AAAGCAGTAA TATGTTAGTT ATAACGGATA AACCAAAATC ACTAAACAAA GTCGGCGTTT   
  
  
- TGAATTAAAA GAAACCACGT ATACTTTAGA ATTTTTCTTA ATTTTATTCT ATATTATGCA ATTGAGAAGT   
  
  
- TGTTGTACTA TATAAATTCT ATGGTTATAA ACATAAGTAG TATATTTTTA ACATCACAAC GTTTTATAAT   
  
  
- TTTAATAAAG TATTTATTTG TAATAATATT CTTGACTGAG GCGCCTAATA GTATACCATA TCTAATGTCT   
  
  
- CACCCACCCA AATTTATTAA AGGGGCTGTG TAATATGAAA ATCTTCTCGA TAATCGAGGT TTAATAAATT   
  
  
- TGTTACAAGA TTTCTCGTTT TTTTAGTTAC AAAACCTAAA ACTAGGCTGG CTCTCCTGAA CAGGCTGGTA   
  
  
- AGGTATAATT TTACAAGGAT CAAGACTAAT CGATTATGGT

+     TCA-element

| Site Name | Organism | Position | Strand | Matrix score. | sequence | function |
| --- | --- | --- | --- | --- | --- | --- |
| TCA-element | Nicotiana tabacum | 1369 | + | 9 | CCATCTTTTT | cis-acting element involved in salicylic acid responsiveness |

>PlantCARE\_25378   
+ ATAAAGTTGT CGTGATTTCT TGCCCTTTAT TTTTTATTTA TATTAACATA TATTTTTAAA AAAAAATTAA   
  
  
+ AGACGTTCCT AACCGAAACT CATGTATTTT TTGATTATGA ATATGAATAT CCCCTCTATA TTAATCATGG   
  
  
+ AGCATTACAA CATGTTTTCG TAGCCATATG TCATCACGAG AATGATTTTT AGAATTGTTA GAAAAATAAA   
  
  
+ TTGATTCATA TAAACATATA CTATGTTTTT TATTAAACTA ACTATCAAAT TAATTAATAG TGTACAAAAA   
  
  
+ AATATTTTTT TCTTTCCTTA AATAAAAACT ACGGAATTAC CTAATATGGC TAACATATAT ATGACAATTA   
  
  
+ ATGATTATGA ATAATACATA TTTGATAAAA AAATTTCTAA CCTCTCTCTT TTTTGTTTAA TTTTATATTA   
  
  
+ TTAAAGGAAA TTTAACAATC ACATTAATCA TATAATAAAA ACAATTAGAT TTTTTCTTAT ATGTTATATT   
  
  
+ TTGAATTTTT AAAAACGACT ATAAATTACT AAAAATGATA AGAGTCCCAC ATTAAAAAAT TTGTGATCAA   
  
  
+ CCGTTTAACT TTTTTTTTAG TTCAAGCAAG ATACAAATGA TCATATATCT GATATAGACG TGGGCGTTCG   
  
  
+ GATACACGTT CGGGTTTGTA TCAGATATTT CAGTATAAAG GTATAGAACC CGTTCGGGTA TTTCTACACT   
  
  
+ CCGAGTCGGG TTCGGGTTCG GATATTTTGG ATCGGGTTCG AATATTTAAA TTTTGAAGAA AAAAAGAAAT   
  
  
+ TATTCACTGT TTAAGTTTTT TATATTTAAA TATATCTTAA CTTAACTGAT TTTTTTTAGT TTTTAAAAGA   
  
  
+ TTAAAATATT AATATGTTTG GAGATAAAAC TTTAAAAATA GAAAGACACT AATTTAGTTT TTGTTTTGAA   
  
  
+ AATTTAGATG CAACTTTTGT TAATGCAAGA AACAAGAACT TGATATGTAT TTTAAGTGAG TAACAAATGA   
  
  
+ TTTTGTCTAT AGTTATATGT ATATTATCTA ATTTTGAGTA ATAAGAATCA TTAATATAAA TATTTTGAAT   
  
  
+ AAAATTAGAT AGATAAACTA TAAATATAGA GTTAAGTATA CTTATGTTTG GTTATCTTCG AATATTACCC   
  
  
+ GTTCGGATAT ATTATCTGAA CTGGTGAAAT AAGTAATATG TTTTGTTGTT TTAATTAGAT AATTTTTAGA   
  
  
+ CCGAGCTTGT GAATATATAC TAGACAAACA TTTATATTTC GAGTCTGCAC TTATATTCTA TAAGAGCTTG   
  
  
+ ATATATTAGA TTTGAACACT AACCTGTTAA TATAGTTTGC CGGTGATTTT TTTTCAAAAT TTTGATTCTT   
  
  
+ AGATATGTAT ATGGAGTAAA ACTAATTTTT ACAGATGCCC ATTTTTTTAA TTGACACTTA TGTAATTAAC   
  
  
+ TGAATTCATA AAACAAGGTT TTTTAAAAAA ATTTAACTCA TATCAATGAA ACAAAGACGA GAACGAAAGC   
  
  
+ ACAATTCTAT GGAAATGGAA AATGAAGTCA CTTATGGAGA TTCAATAGTA AGCAAATCGA GAGCAGAAAA   
  
  
+ TCTAATCTCC TTTCGTCATT ATACAATCAA TATTGCCTAT TTGGTTTTAG TGATTTGTTT CAGCCGCAAA   
  
  
+ ACTTAATTTT CTTTGGTGCA TATGAAATCT TAAAAAGAAT TAAAATAAGA TATAATACGT TAACTCTTCA   
  
  
+ ACAACATGAT ATATTTAAGA TACCAATATT TGTATTCATC ATATAAAAAT TGTAGTGTTG CAAAATATTA   
  
  
+ AAATTATTTC ATAAATAAAC ATTATTATAA GAACTGACTC CGCGGATTAT CATATGGTAT AGATTACAGA   
  
  
+ GTGGGTGGGT TTAAATAATT TCCCCGACAC ATTATACTTT TAGAAGAGCT ATTAGCTCCA AATTATTTAA   
  
  
+ ACAATGTTCT AAAGAGCAAA AAAATCAATG TTTTGGATTT TGATCCGACC GAGAGGACTT GTCCGACCAT   
  
  
+ TCCATATTAA AATGTTCCTA GTTCTGATTA GCTAATACCA   

- TATTTCAACA GCACTAAAGA ACGGGAAATA AAAAATAAAT ATAATTGTAT ATAAAAATTT TTTTTTAATT   
  
  
- TCTGCAAGGA TTGGCTTTGA GTACATAAAA AACTAATACT TATACTTATA GGGGAGATAT AATTAGTACC   
  
  
- TCGTAATGTT GTACAAAAGC ATCGGTATAC AGTAGTGCTC TTACTAAAAA TCTTAACAAT CTTTTTATTT   
  
  
- AACTAAGTAT ATTTGTATAT GATACAAAAA ATAATTTGAT TGATAGTTTA ATTAATTATC ACATGTTTTT   
  
  
- TTATAAAAAA AGAAAGGAAT TTATTTTTGA TGCCTTAATG GATTATACCG ATTGTATATA TACTGTTAAT   
  
  
- TACTAATACT TATTATGTAT AAACTATTTT TTTAAAGATT GGAGAGAGAA AAAACAAATT AAAATATAAT   
  
  
- AATTTCCTTT AAATTGTTAG TGTAATTAGT ATATTATTTT TGTTAATCTA AAAAAGAATA TACAATATAA   
  
  
- AACTTAAAAA TTTTTGCTGA TATTTAATGA TTTTTACTAT TCTCAGGGTG TAATTTTTTA AACACTAGTT   
  
  
- GGCAAATTGA AAAAAAAATC AAGTTCGTTC TATGTTTACT AGTATATAGA CTATATCTGC ACCCGCAAGC   
  
  
- CTATGTGCAA GCCCAAACAT AGTCTATAAA GTCATATTTC CATATCTTGG GCAAGCCCAT AAAGATGTGA   
  
  
- GGCTCAGCCC AAGCCCAAGC CTATAAAACC TAGCCCAAGC TTATAAATTT AAAACTTCTT TTTTTCTTTA   
  
  
- ATAAGTGACA AATTCAAAAA ATATAAATTT ATATAGAATT GAATTGACTA AAAAAAATCA AAAATTTTCT   
  
  
- AATTTTATAA TTATACAAAC CTCTATTTTG AAATTTTTAT CTTTCTGTGA TTAAATCAAA AACAAAACTT   
  
  
- TTAAATCTAC GTTGAAAACA ATTACGTTCT TTGTTCTTGA ACTATACATA AAATTCACTC ATTGTTTACT   
  
  
- AAAACAGATA TCAATATACA TATAATAGAT TAAAACTCAT TATTCTTAGT AATTATATTT ATAAAACTTA   
  
  
- TTTTAATCTA TCTATTTGAT ATTTATATCT CAATTCATAT GAATACAAAC CAATAGAAGC TTATAATGGG   
  
  
- CAAGCCTATA TAATAGACTT GACCACTTTA TTCATTATAC AAAACAACAA AATTAATCTA TTAAAAATCT   
  
  
- GGCTCGAACA CTTATATATG ATCTGTTTGT AAATATAAAG CTCAGACGTG AATATAAGAT ATTCTCGAAC   
  
  
- TATATAATCT AAACTTGTGA TTGGACAATT ATATCAAACG GCCACTAAAA AAAAGTTTTA AAACTAAGAA   
  
  
- TCTATACATA TACCTCATTT TGATTAAAAA TGTCTACGGG TAAAAAAATT AACTGTGAAT ACATTAATTG   
  
  
- ACTTAAGTAT TTTGTTCCAA AAAATTTTTT TAAATTGAGT ATAGTTACTT TGTTTCTGCT CTTGCTTTCG   
  
  
- TGTTAAGATA CCTTTACCTT TTACTTCAGT GAATACCTCT AAGTTATCAT TCGTTTAGCT CTCGTCTTTT   
  
  
- AGATTAGAGG AAAGCAGTAA TATGTTAGTT ATAACGGATA AACCAAAATC ACTAAACAAA GTCGGCGTTT   
  
  
- TGAATTAAAA GAAACCACGT ATACTTTAGA ATTTTTCTTA ATTTTATTCT ATATTATGCA ATTGAGAAGT   
  
  
- TGTTGTACTA TATAAATTCT ATGGTTATAA ACATAAGTAG TATATTTTTA ACATCACAAC GTTTTATAAT   
  
  
- TTTAATAAAG TATTTATTTG TAATAATATT CTTGACTGAG GCGCCTAATA GTATACCATA TCTAATGTCT   
  
  
- CACCCACCCA AATTTATTAA AGGGGCTGTG TAATATGAAA ATCTTCTCGA TAATCGAGGT TTAATAAATT   
  
  
- TGTTACAAGA TTTCTCGTTT TTTTAGTTAC AAAACCTAAA ACTAGGCTGG CTCTCCTGAA CAGGCTGGTA   
  
  
- AGGTATAATT TTACAAGGAT CAAGACTAAT CGATTATGGT

+     TGA-element

| Site Name | Organism | Position | Strand | Matrix score. | sequence | function |
| --- | --- | --- | --- | --- | --- | --- |
| TGA-element | Brassica oleracea | 504 | + | 6 | AACGAC | auxin-responsive element |

>PlantCARE\_25378   
+ ATAAAGTTGT CGTGATTTCT TGCCCTTTAT TTTTTATTTA TATTAACATA TATTTTTAAA AAAAAATTAA   
  
  
+ AGACGTTCCT AACCGAAACT CATGTATTTT TTGATTATGA ATATGAATAT CCCCTCTATA TTAATCATGG   
  
  
+ AGCATTACAA CATGTTTTCG TAGCCATATG TCATCACGAG AATGATTTTT AGAATTGTTA GAAAAATAAA   
  
  
+ TTGATTCATA TAAACATATA CTATGTTTTT TATTAAACTA ACTATCAAAT TAATTAATAG TGTACAAAAA   
  
  
+ AATATTTTTT TCTTTCCTTA AATAAAAACT ACGGAATTAC CTAATATGGC TAACATATAT ATGACAATTA   
  
  
+ ATGATTATGA ATAATACATA TTTGATAAAA AAATTTCTAA CCTCTCTCTT TTTTGTTTAA TTTTATATTA   
  
  
+ TTAAAGGAAA TTTAACAATC ACATTAATCA TATAATAAAA ACAATTAGAT TTTTTCTTAT ATGTTATATT   
  
  
+ TTGAATTTTT AAAAACGACT ATAAATTACT AAAAATGATA AGAGTCCCAC ATTAAAAAAT TTGTGATCAA   
  
  
+ CCGTTTAACT TTTTTTTTAG TTCAAGCAAG ATACAAATGA TCATATATCT GATATAGACG TGGGCGTTCG   
  
  
+ GATACACGTT CGGGTTTGTA TCAGATATTT CAGTATAAAG GTATAGAACC CGTTCGGGTA TTTCTACACT   
  
  
+ CCGAGTCGGG TTCGGGTTCG GATATTTTGG ATCGGGTTCG AATATTTAAA TTTTGAAGAA AAAAAGAAAT   
  
  
+ TATTCACTGT TTAAGTTTTT TATATTTAAA TATATCTTAA CTTAACTGAT TTTTTTTAGT TTTTAAAAGA   
  
  
+ TTAAAATATT AATATGTTTG GAGATAAAAC TTTAAAAATA GAAAGACACT AATTTAGTTT TTGTTTTGAA   
  
  
+ AATTTAGATG CAACTTTTGT TAATGCAAGA AACAAGAACT TGATATGTAT TTTAAGTGAG TAACAAATGA   
  
  
+ TTTTGTCTAT AGTTATATGT ATATTATCTA ATTTTGAGTA ATAAGAATCA TTAATATAAA TATTTTGAAT   
  
  
+ AAAATTAGAT AGATAAACTA TAAATATAGA GTTAAGTATA CTTATGTTTG GTTATCTTCG AATATTACCC   
  
  
+ GTTCGGATAT ATTATCTGAA CTGGTGAAAT AAGTAATATG TTTTGTTGTT TTAATTAGAT AATTTTTAGA   
  
  
+ CCGAGCTTGT GAATATATAC TAGACAAACA TTTATATTTC GAGTCTGCAC TTATATTCTA TAAGAGCTTG   
  
  
+ ATATATTAGA TTTGAACACT AACCTGTTAA TATAGTTTGC CGGTGATTTT TTTTCAAAAT TTTGATTCTT   
  
  
+ AGATATGTAT ATGGAGTAAA ACTAATTTTT ACAGATGCCC ATTTTTTTAA TTGACACTTA TGTAATTAAC   
  
  
+ TGAATTCATA AAACAAGGTT TTTTAAAAAA ATTTAACTCA TATCAATGAA ACAAAGACGA GAACGAAAGC   
  
  
+ ACAATTCTAT GGAAATGGAA AATGAAGTCA CTTATGGAGA TTCAATAGTA AGCAAATCGA GAGCAGAAAA   
  
  
+ TCTAATCTCC TTTCGTCATT ATACAATCAA TATTGCCTAT TTGGTTTTAG TGATTTGTTT CAGCCGCAAA   
  
  
+ ACTTAATTTT CTTTGGTGCA TATGAAATCT TAAAAAGAAT TAAAATAAGA TATAATACGT TAACTCTTCA   
  
  
+ ACAACATGAT ATATTTAAGA TACCAATATT TGTATTCATC ATATAAAAAT TGTAGTGTTG CAAAATATTA   
  
  
+ AAATTATTTC ATAAATAAAC ATTATTATAA GAACTGACTC CGCGGATTAT CATATGGTAT AGATTACAGA   
  
  
+ GTGGGTGGGT TTAAATAATT TCCCCGACAC ATTATACTTT TAGAAGAGCT ATTAGCTCCA AATTATTTAA   
  
  
+ ACAATGTTCT AAAGAGCAAA AAAATCAATG TTTTGGATTT TGATCCGACC GAGAGGACTT GTCCGACCAT   
  
  
+ TCCATATTAA AATGTTCCTA GTTCTGATTA GCTAATACCA   

- TATTTCAACA GCACTAAAGA ACGGGAAATA AAAAATAAAT ATAATTGTAT ATAAAAATTT TTTTTTAATT   
  
  
- TCTGCAAGGA TTGGCTTTGA GTACATAAAA AACTAATACT TATACTTATA GGGGAGATAT AATTAGTACC   
  
  
- TCGTAATGTT GTACAAAAGC ATCGGTATAC AGTAGTGCTC TTACTAAAAA TCTTAACAAT CTTTTTATTT   
  
  
- AACTAAGTAT ATTTGTATAT GATACAAAAA ATAATTTGAT TGATAGTTTA ATTAATTATC ACATGTTTTT   
  
  
- TTATAAAAAA AGAAAGGAAT TTATTTTTGA TGCCTTAATG GATTATACCG ATTGTATATA TACTGTTAAT   
  
  
- TACTAATACT TATTATGTAT AAACTATTTT TTTAAAGATT GGAGAGAGAA AAAACAAATT AAAATATAAT   
  
  
- AATTTCCTTT AAATTGTTAG TGTAATTAGT ATATTATTTT TGTTAATCTA AAAAAGAATA TACAATATAA   
  
  
- AACTTAAAAA TTTTTGCTGA TATTTAATGA TTTTTACTAT TCTCAGGGTG TAATTTTTTA AACACTAGTT   
  
  
- GGCAAATTGA AAAAAAAATC AAGTTCGTTC TATGTTTACT AGTATATAGA CTATATCTGC ACCCGCAAGC   
  
  
- CTATGTGCAA GCCCAAACAT AGTCTATAAA GTCATATTTC CATATCTTGG GCAAGCCCAT AAAGATGTGA   
  
  
- GGCTCAGCCC AAGCCCAAGC CTATAAAACC TAGCCCAAGC TTATAAATTT AAAACTTCTT TTTTTCTTTA   
  
  
- ATAAGTGACA AATTCAAAAA ATATAAATTT ATATAGAATT GAATTGACTA AAAAAAATCA AAAATTTTCT   
  
  
- AATTTTATAA TTATACAAAC CTCTATTTTG AAATTTTTAT CTTTCTGTGA TTAAATCAAA AACAAAACTT   
  
  
- TTAAATCTAC GTTGAAAACA ATTACGTTCT TTGTTCTTGA ACTATACATA AAATTCACTC ATTGTTTACT   
  
  
- AAAACAGATA TCAATATACA TATAATAGAT TAAAACTCAT TATTCTTAGT AATTATATTT ATAAAACTTA   
  
  
- TTTTAATCTA TCTATTTGAT ATTTATATCT CAATTCATAT GAATACAAAC CAATAGAAGC TTATAATGGG   
  
  
- CAAGCCTATA TAATAGACTT GACCACTTTA TTCATTATAC AAAACAACAA AATTAATCTA TTAAAAATCT   
  
  
- GGCTCGAACA CTTATATATG ATCTGTTTGT AAATATAAAG CTCAGACGTG AATATAAGAT ATTCTCGAAC   
  
  
- TATATAATCT AAACTTGTGA TTGGACAATT ATATCAAACG GCCACTAAAA AAAAGTTTTA AAACTAAGAA   
  
  
- TCTATACATA TACCTCATTT TGATTAAAAA TGTCTACGGG TAAAAAAATT AACTGTGAAT ACATTAATTG   
  
  
- ACTTAAGTAT TTTGTTCCAA AAAATTTTTT TAAATTGAGT ATAGTTACTT TGTTTCTGCT CTTGCTTTCG   
  
  
- TGTTAAGATA CCTTTACCTT TTACTTCAGT GAATACCTCT AAGTTATCAT TCGTTTAGCT CTCGTCTTTT   
  
  
- AGATTAGAGG AAAGCAGTAA TATGTTAGTT ATAACGGATA AACCAAAATC ACTAAACAAA GTCGGCGTTT   
  
  
- TGAATTAAAA GAAACCACGT ATACTTTAGA ATTTTTCTTA ATTTTATTCT ATATTATGCA ATTGAGAAGT   
  
  
- TGTTGTACTA TATAAATTCT ATGGTTATAA ACATAAGTAG TATATTTTTA ACATCACAAC GTTTTATAAT   
  
  
- TTTAATAAAG TATTTATTTG TAATAATATT CTTGACTGAG GCGCCTAATA GTATACCATA TCTAATGTCT   
  
  
- CACCCACCCA AATTTATTAA AGGGGCTGTG TAATATGAAA ATCTTCTCGA TAATCGAGGT TTAATAAATT   
  
  
- TGTTACAAGA TTTCTCGTTT TTTTAGTTAC AAAACCTAAA ACTAGGCTGG CTCTCCTGAA CAGGCTGGTA   
  
  
- AGGTATAATT TTACAAGGAT CAAGACTAAT CGATTATGGT

+     TGACG-motif

| Site Name | Organism | Position | Strand | Matrix score. | sequence | function |
| --- | --- | --- | --- | --- | --- | --- |
| TGACG-motif | Hordeum vulgare | 1554 | - | 5 | TGACG | cis-acting regulatory element involved in the MeJA-responsiveness |

>PlantCARE\_25378   
+ ATAAAGTTGT CGTGATTTCT TGCCCTTTAT TTTTTATTTA TATTAACATA TATTTTTAAA AAAAAATTAA   
  
  
+ AGACGTTCCT AACCGAAACT CATGTATTTT TTGATTATGA ATATGAATAT CCCCTCTATA TTAATCATGG   
  
  
+ AGCATTACAA CATGTTTTCG TAGCCATATG TCATCACGAG AATGATTTTT AGAATTGTTA GAAAAATAAA   
  
  
+ TTGATTCATA TAAACATATA CTATGTTTTT TATTAAACTA ACTATCAAAT TAATTAATAG TGTACAAAAA   
  
  
+ AATATTTTTT TCTTTCCTTA AATAAAAACT ACGGAATTAC CTAATATGGC TAACATATAT ATGACAATTA   
  
  
+ ATGATTATGA ATAATACATA TTTGATAAAA AAATTTCTAA CCTCTCTCTT TTTTGTTTAA TTTTATATTA   
  
  
+ TTAAAGGAAA TTTAACAATC ACATTAATCA TATAATAAAA ACAATTAGAT TTTTTCTTAT ATGTTATATT   
  
  
+ TTGAATTTTT AAAAACGACT ATAAATTACT AAAAATGATA AGAGTCCCAC ATTAAAAAAT TTGTGATCAA   
  
  
+ CCGTTTAACT TTTTTTTTAG TTCAAGCAAG ATACAAATGA TCATATATCT GATATAGACG TGGGCGTTCG   
  
  
+ GATACACGTT CGGGTTTGTA TCAGATATTT CAGTATAAAG GTATAGAACC CGTTCGGGTA TTTCTACACT   
  
  
+ CCGAGTCGGG TTCGGGTTCG GATATTTTGG ATCGGGTTCG AATATTTAAA TTTTGAAGAA AAAAAGAAAT   
  
  
+ TATTCACTGT TTAAGTTTTT TATATTTAAA TATATCTTAA CTTAACTGAT TTTTTTTAGT TTTTAAAAGA   
  
  
+ TTAAAATATT AATATGTTTG GAGATAAAAC TTTAAAAATA GAAAGACACT AATTTAGTTT TTGTTTTGAA   
  
  
+ AATTTAGATG CAACTTTTGT TAATGCAAGA AACAAGAACT TGATATGTAT TTTAAGTGAG TAACAAATGA   
  
  
+ TTTTGTCTAT AGTTATATGT ATATTATCTA ATTTTGAGTA ATAAGAATCA TTAATATAAA TATTTTGAAT   
  
  
+ AAAATTAGAT AGATAAACTA TAAATATAGA GTTAAGTATA CTTATGTTTG GTTATCTTCG AATATTACCC   
  
  
+ GTTCGGATAT ATTATCTGAA CTGGTGAAAT AAGTAATATG TTTTGTTGTT TTAATTAGAT AATTTTTAGA   
  
  
+ CCGAGCTTGT GAATATATAC TAGACAAACA TTTATATTTC GAGTCTGCAC TTATATTCTA TAAGAGCTTG   
  
  
+ ATATATTAGA TTTGAACACT AACCTGTTAA TATAGTTTGC CGGTGATTTT TTTTCAAAAT TTTGATTCTT   
  
  
+ AGATATGTAT ATGGAGTAAA ACTAATTTTT ACAGATGCCC ATTTTTTTAA TTGACACTTA TGTAATTAAC   
  
  
+ TGAATTCATA AAACAAGGTT TTTTAAAAAA ATTTAACTCA TATCAATGAA ACAAAGACGA GAACGAAAGC   
  
  
+ ACAATTCTAT GGAAATGGAA AATGAAGTCA CTTATGGAGA TTCAATAGTA AGCAAATCGA GAGCAGAAAA   
  
  
+ TCTAATCTCC TTTCGTCATT ATACAATCAA TATTGCCTAT TTGGTTTTAG TGATTTGTTT CAGCCGCAAA   
  
  
+ ACTTAATTTT CTTTGGTGCA TATGAAATCT TAAAAAGAAT TAAAATAAGA TATAATACGT TAACTCTTCA   
  
  
+ ACAACATGAT ATATTTAAGA TACCAATATT TGTATTCATC ATATAAAAAT TGTAGTGTTG CAAAATATTA   
  
  
+ AAATTATTTC ATAAATAAAC ATTATTATAA GAACTGACTC CGCGGATTAT CATATGGTAT AGATTACAGA   
  
  
+ GTGGGTGGGT TTAAATAATT TCCCCGACAC ATTATACTTT TAGAAGAGCT ATTAGCTCCA AATTATTTAA   
  
  
+ ACAATGTTCT AAAGAGCAAA AAAATCAATG TTTTGGATTT TGATCCGACC GAGAGGACTT GTCCGACCAT   
  
  
+ TCCATATTAA AATGTTCCTA GTTCTGATTA GCTAATACCA   

- TATTTCAACA GCACTAAAGA ACGGGAAATA AAAAATAAAT ATAATTGTAT ATAAAAATTT TTTTTTAATT   
  
  
- TCTGCAAGGA TTGGCTTTGA GTACATAAAA AACTAATACT TATACTTATA GGGGAGATAT AATTAGTACC   
  
  
- TCGTAATGTT GTACAAAAGC ATCGGTATAC AGTAGTGCTC TTACTAAAAA TCTTAACAAT CTTTTTATTT   
  
  
- AACTAAGTAT ATTTGTATAT GATACAAAAA ATAATTTGAT TGATAGTTTA ATTAATTATC ACATGTTTTT   
  
  
- TTATAAAAAA AGAAAGGAAT TTATTTTTGA TGCCTTAATG GATTATACCG ATTGTATATA TACTGTTAAT   
  
  
- TACTAATACT TATTATGTAT AAACTATTTT TTTAAAGATT GGAGAGAGAA AAAACAAATT AAAATATAAT   
  
  
- AATTTCCTTT AAATTGTTAG TGTAATTAGT ATATTATTTT TGTTAATCTA AAAAAGAATA TACAATATAA   
  
  
- AACTTAAAAA TTTTTGCTGA TATTTAATGA TTTTTACTAT TCTCAGGGTG TAATTTTTTA AACACTAGTT   
  
  
- GGCAAATTGA AAAAAAAATC AAGTTCGTTC TATGTTTACT AGTATATAGA CTATATCTGC ACCCGCAAGC   
  
  
- CTATGTGCAA GCCCAAACAT AGTCTATAAA GTCATATTTC CATATCTTGG GCAAGCCCAT AAAGATGTGA   
  
  
- GGCTCAGCCC AAGCCCAAGC CTATAAAACC TAGCCCAAGC TTATAAATTT AAAACTTCTT TTTTTCTTTA   
  
  
- ATAAGTGACA AATTCAAAAA ATATAAATTT ATATAGAATT GAATTGACTA AAAAAAATCA AAAATTTTCT   
  
  
- AATTTTATAA TTATACAAAC CTCTATTTTG AAATTTTTAT CTTTCTGTGA TTAAATCAAA AACAAAACTT   
  
  
- TTAAATCTAC GTTGAAAACA ATTACGTTCT TTGTTCTTGA ACTATACATA AAATTCACTC ATTGTTTACT   
  
  
- AAAACAGATA TCAATATACA TATAATAGAT TAAAACTCAT TATTCTTAGT AATTATATTT ATAAAACTTA   
  
  
- TTTTAATCTA TCTATTTGAT ATTTATATCT CAATTCATAT GAATACAAAC CAATAGAAGC TTATAATGGG   
  
  
- CAAGCCTATA TAATAGACTT GACCACTTTA TTCATTATAC AAAACAACAA AATTAATCTA TTAAAAATCT   
  
  
- GGCTCGAACA CTTATATATG ATCTGTTTGT AAATATAAAG CTCAGACGTG AATATAAGAT ATTCTCGAAC   
  
  
- TATATAATCT AAACTTGTGA TTGGACAATT ATATCAAACG GCCACTAAAA AAAAGTTTTA AAACTAAGAA   
  
  
- TCTATACATA TACCTCATTT TGATTAAAAA TGTCTACGGG TAAAAAAATT AACTGTGAAT ACATTAATTG   
  
  
- ACTTAAGTAT TTTGTTCCAA AAAATTTTTT TAAATTGAGT ATAGTTACTT TGTTTCTGCT CTTGCTTTCG   
  
  
- TGTTAAGATA CCTTTACCTT TTACTTCAGT GAATACCTCT AAGTTATCAT TCGTTTAGCT CTCGTCTTTT   
  
  
- AGATTAGAGG AAAGCAGTAA TATGTTAGTT ATAACGGATA AACCAAAATC ACTAAACAAA GTCGGCGTTT   
  
  
- TGAATTAAAA GAAACCACGT ATACTTTAGA ATTTTTCTTA ATTTTATTCT ATATTATGCA ATTGAGAAGT   
  
  
- TGTTGTACTA TATAAATTCT ATGGTTATAA ACATAAGTAG TATATTTTTA ACATCACAAC GTTTTATAAT   
  
  
- TTTAATAAAG TATTTATTTG TAATAATATT CTTGACTGAG GCGCCTAATA GTATACCATA TCTAATGTCT   
  
  
- CACCCACCCA AATTTATTAA AGGGGCTGTG TAATATGAAA ATCTTCTCGA TAATCGAGGT TTAATAAATT   
  
  
- TGTTACAAGA TTTCTCGTTT TTTTAGTTAC AAAACCTAAA ACTAGGCTGG CTCTCCTGAA CAGGCTGGTA   
  
  
- AGGTATAATT TTACAAGGAT CAAGACTAAT CGATTATGGT

+     Unnamed\_\_1

| Site Name | Organism | Position | Strand | Matrix score. | sequence | function |
| --- | --- | --- | --- | --- | --- | --- |
| Unnamed\_\_1 | Zea mays | 619 | + | 5 | CGTGG |  |

>PlantCARE\_25378   
+ ATAAAGTTGT CGTGATTTCT TGCCCTTTAT TTTTTATTTA TATTAACATA TATTTTTAAA AAAAAATTAA   
  
  
+ AGACGTTCCT AACCGAAACT CATGTATTTT TTGATTATGA ATATGAATAT CCCCTCTATA TTAATCATGG   
  
  
+ AGCATTACAA CATGTTTTCG TAGCCATATG TCATCACGAG AATGATTTTT AGAATTGTTA GAAAAATAAA   
  
  
+ TTGATTCATA TAAACATATA CTATGTTTTT TATTAAACTA ACTATCAAAT TAATTAATAG TGTACAAAAA   
  
  
+ AATATTTTTT TCTTTCCTTA AATAAAAACT ACGGAATTAC CTAATATGGC TAACATATAT ATGACAATTA   
  
  
+ ATGATTATGA ATAATACATA TTTGATAAAA AAATTTCTAA CCTCTCTCTT TTTTGTTTAA TTTTATATTA   
  
  
+ TTAAAGGAAA TTTAACAATC ACATTAATCA TATAATAAAA ACAATTAGAT TTTTTCTTAT ATGTTATATT   
  
  
+ TTGAATTTTT AAAAACGACT ATAAATTACT AAAAATGATA AGAGTCCCAC ATTAAAAAAT TTGTGATCAA   
  
  
+ CCGTTTAACT TTTTTTTTAG TTCAAGCAAG ATACAAATGA TCATATATCT GATATAGACG TGGGCGTTCG   
  
  
+ GATACACGTT CGGGTTTGTA TCAGATATTT CAGTATAAAG GTATAGAACC CGTTCGGGTA TTTCTACACT   
  
  
+ CCGAGTCGGG TTCGGGTTCG GATATTTTGG ATCGGGTTCG AATATTTAAA TTTTGAAGAA AAAAAGAAAT   
  
  
+ TATTCACTGT TTAAGTTTTT TATATTTAAA TATATCTTAA CTTAACTGAT TTTTTTTAGT TTTTAAAAGA   
  
  
+ TTAAAATATT AATATGTTTG GAGATAAAAC TTTAAAAATA GAAAGACACT AATTTAGTTT TTGTTTTGAA   
  
  
+ AATTTAGATG CAACTTTTGT TAATGCAAGA AACAAGAACT TGATATGTAT TTTAAGTGAG TAACAAATGA   
  
  
+ TTTTGTCTAT AGTTATATGT ATATTATCTA ATTTTGAGTA ATAAGAATCA TTAATATAAA TATTTTGAAT   
  
  
+ AAAATTAGAT AGATAAACTA TAAATATAGA GTTAAGTATA CTTATGTTTG GTTATCTTCG AATATTACCC   
  
  
+ GTTCGGATAT ATTATCTGAA CTGGTGAAAT AAGTAATATG TTTTGTTGTT TTAATTAGAT AATTTTTAGA   
  
  
+ CCGAGCTTGT GAATATATAC TAGACAAACA TTTATATTTC GAGTCTGCAC TTATATTCTA TAAGAGCTTG   
  
  
+ ATATATTAGA TTTGAACACT AACCTGTTAA TATAGTTTGC CGGTGATTTT TTTTCAAAAT TTTGATTCTT   
  
  
+ AGATATGTAT ATGGAGTAAA ACTAATTTTT ACAGATGCCC ATTTTTTTAA TTGACACTTA TGTAATTAAC   
  
  
+ TGAATTCATA AAACAAGGTT TTTTAAAAAA ATTTAACTCA TATCAATGAA ACAAAGACGA GAACGAAAGC   
  
  
+ ACAATTCTAT GGAAATGGAA AATGAAGTCA CTTATGGAGA TTCAATAGTA AGCAAATCGA GAGCAGAAAA   
  
  
+ TCTAATCTCC TTTCGTCATT ATACAATCAA TATTGCCTAT TTGGTTTTAG TGATTTGTTT CAGCCGCAAA   
  
  
+ ACTTAATTTT CTTTGGTGCA TATGAAATCT TAAAAAGAAT TAAAATAAGA TATAATACGT TAACTCTTCA   
  
  
+ ACAACATGAT ATATTTAAGA TACCAATATT TGTATTCATC ATATAAAAAT TGTAGTGTTG CAAAATATTA   
  
  
+ AAATTATTTC ATAAATAAAC ATTATTATAA GAACTGACTC CGCGGATTAT CATATGGTAT AGATTACAGA   
  
  
+ GTGGGTGGGT TTAAATAATT TCCCCGACAC ATTATACTTT TAGAAGAGCT ATTAGCTCCA AATTATTTAA   
  
  
+ ACAATGTTCT AAAGAGCAAA AAAATCAATG TTTTGGATTT TGATCCGACC GAGAGGACTT GTCCGACCAT   
  
  
+ TCCATATTAA AATGTTCCTA GTTCTGATTA GCTAATACCA   

- TATTTCAACA GCACTAAAGA ACGGGAAATA AAAAATAAAT ATAATTGTAT ATAAAAATTT TTTTTTAATT   
  
  
- TCTGCAAGGA TTGGCTTTGA GTACATAAAA AACTAATACT TATACTTATA GGGGAGATAT AATTAGTACC   
  
  
- TCGTAATGTT GTACAAAAGC ATCGGTATAC AGTAGTGCTC TTACTAAAAA TCTTAACAAT CTTTTTATTT   
  
  
- AACTAAGTAT ATTTGTATAT GATACAAAAA ATAATTTGAT TGATAGTTTA ATTAATTATC ACATGTTTTT   
  
  
- TTATAAAAAA AGAAAGGAAT TTATTTTTGA TGCCTTAATG GATTATACCG ATTGTATATA TACTGTTAAT   
  
  
- TACTAATACT TATTATGTAT AAACTATTTT TTTAAAGATT GGAGAGAGAA AAAACAAATT AAAATATAAT   
  
  
- AATTTCCTTT AAATTGTTAG TGTAATTAGT ATATTATTTT TGTTAATCTA AAAAAGAATA TACAATATAA   
  
  
- AACTTAAAAA TTTTTGCTGA TATTTAATGA TTTTTACTAT TCTCAGGGTG TAATTTTTTA AACACTAGTT   
  
  
- GGCAAATTGA AAAAAAAATC AAGTTCGTTC TATGTTTACT AGTATATAGA CTATATCTGC ACCCGCAAGC   
  
  
- CTATGTGCAA GCCCAAACAT AGTCTATAAA GTCATATTTC CATATCTTGG GCAAGCCCAT AAAGATGTGA   
  
  
- GGCTCAGCCC AAGCCCAAGC CTATAAAACC TAGCCCAAGC TTATAAATTT AAAACTTCTT TTTTTCTTTA   
  
  
- ATAAGTGACA AATTCAAAAA ATATAAATTT ATATAGAATT GAATTGACTA AAAAAAATCA AAAATTTTCT   
  
  
- AATTTTATAA TTATACAAAC CTCTATTTTG AAATTTTTAT CTTTCTGTGA TTAAATCAAA AACAAAACTT   
  
  
- TTAAATCTAC GTTGAAAACA ATTACGTTCT TTGTTCTTGA ACTATACATA AAATTCACTC ATTGTTTACT   
  
  
- AAAACAGATA TCAATATACA TATAATAGAT TAAAACTCAT TATTCTTAGT AATTATATTT ATAAAACTTA   
  
  
- TTTTAATCTA TCTATTTGAT ATTTATATCT CAATTCATAT GAATACAAAC CAATAGAAGC TTATAATGGG   
  
  
- CAAGCCTATA TAATAGACTT GACCACTTTA TTCATTATAC AAAACAACAA AATTAATCTA TTAAAAATCT   
  
  
- GGCTCGAACA CTTATATATG ATCTGTTTGT AAATATAAAG CTCAGACGTG AATATAAGAT ATTCTCGAAC   
  
  
- TATATAATCT AAACTTGTGA TTGGACAATT ATATCAAACG GCCACTAAAA AAAAGTTTTA AAACTAAGAA   
  
  
- TCTATACATA TACCTCATTT TGATTAAAAA TGTCTACGGG TAAAAAAATT AACTGTGAAT ACATTAATTG   
  
  
- ACTTAAGTAT TTTGTTCCAA AAAATTTTTT TAAATTGAGT ATAGTTACTT TGTTTCTGCT CTTGCTTTCG   
  
  
- TGTTAAGATA CCTTTACCTT TTACTTCAGT GAATACCTCT AAGTTATCAT TCGTTTAGCT CTCGTCTTTT   
  
  
- AGATTAGAGG AAAGCAGTAA TATGTTAGTT ATAACGGATA AACCAAAATC ACTAAACAAA GTCGGCGTTT   
  
  
- TGAATTAAAA GAAACCACGT ATACTTTAGA ATTTTTCTTA ATTTTATTCT ATATTATGCA ATTGAGAAGT   
  
  
- TGTTGTACTA TATAAATTCT ATGGTTATAA ACATAAGTAG TATATTTTTA ACATCACAAC GTTTTATAAT   
  
  
- TTTAATAAAG TATTTATTTG TAATAATATT CTTGACTGAG GCGCCTAATA GTATACCATA TCTAATGTCT   
  
  
- CACCCACCCA AATTTATTAA AGGGGCTGTG TAATATGAAA ATCTTCTCGA TAATCGAGGT TTAATAAATT   
  
  
- TGTTACAAGA TTTCTCGTTT TTTTAGTTAC AAAACCTAAA ACTAGGCTGG CTCTCCTGAA CAGGCTGGTA   
  
  
- AGGTATAATT TTACAAGGAT CAAGACTAAT CGATTATGGT

+     Unnamed\_\_4

| Site Name | Organism | Position | Strand | Matrix score. | sequence | function |
| --- | --- | --- | --- | --- | --- | --- |
| Unnamed\_\_4 | Petroselinum hortense | 139 | - | 4 | CTCC |  |
| Unnamed\_\_4 | Petroselinum hortense | 1506 | - | 4 | CTCC |  |
| Unnamed\_\_4 | Petroselinum hortense | 860 | - | 4 | CTCC |  |
| Unnamed\_\_4 | Petroselinum hortense | 1788 | + | 4 | CTCC |  |
| Unnamed\_\_4 | Petroselinum hortense | 699 | + | 4 | CTCC |  |
| Unnamed\_\_4 | Petroselinum hortense | 1547 | + | 4 | CTCC |  |
| Unnamed\_\_4 | Petroselinum hortense | 1343 | - | 4 | CTCC |  |
| Unnamed\_\_4 | Petroselinum hortense | 1876 | + | 4 | CTCC |  |

>PlantCARE\_25378   
+ ATAAAGTTGT CGTGATTTCT TGCCCTTTAT TTTTTATTTA TATTAACATA TATTTTTAAA AAAAAATTAA   
  
  
+ AGACGTTCCT AACCGAAACT CATGTATTTT TTGATTATGA ATATGAATAT CCCCTCTATA TTAATCATGG   
  
  
+ AGCATTACAA CATGTTTTCG TAGCCATATG TCATCACGAG AATGATTTTT AGAATTGTTA GAAAAATAAA   
  
  
+ TTGATTCATA TAAACATATA CTATGTTTTT TATTAAACTA ACTATCAAAT TAATTAATAG TGTACAAAAA   
  
  
+ AATATTTTTT TCTTTCCTTA AATAAAAACT ACGGAATTAC CTAATATGGC TAACATATAT ATGACAATTA   
  
  
+ ATGATTATGA ATAATACATA TTTGATAAAA AAATTTCTAA CCTCTCTCTT TTTTGTTTAA TTTTATATTA   
  
  
+ TTAAAGGAAA TTTAACAATC ACATTAATCA TATAATAAAA ACAATTAGAT TTTTTCTTAT ATGTTATATT   
  
  
+ TTGAATTTTT AAAAACGACT ATAAATTACT AAAAATGATA AGAGTCCCAC ATTAAAAAAT TTGTGATCAA   
  
  
+ CCGTTTAACT TTTTTTTTAG TTCAAGCAAG ATACAAATGA TCATATATCT GATATAGACG TGGGCGTTCG   
  
  
+ GATACACGTT CGGGTTTGTA TCAGATATTT CAGTATAAAG GTATAGAACC CGTTCGGGTA TTTCTACACT   
  
  
+ CCGAGTCGGG TTCGGGTTCG GATATTTTGG ATCGGGTTCG AATATTTAAA TTTTGAAGAA AAAAAGAAAT   
  
  
+ TATTCACTGT TTAAGTTTTT TATATTTAAA TATATCTTAA CTTAACTGAT TTTTTTTAGT TTTTAAAAGA   
  
  
+ TTAAAATATT AATATGTTTG GAGATAAAAC TTTAAAAATA GAAAGACACT AATTTAGTTT TTGTTTTGAA   
  
  
+ AATTTAGATG CAACTTTTGT TAATGCAAGA AACAAGAACT TGATATGTAT TTTAAGTGAG TAACAAATGA   
  
  
+ TTTTGTCTAT AGTTATATGT ATATTATCTA ATTTTGAGTA ATAAGAATCA TTAATATAAA TATTTTGAAT   
  
  
+ AAAATTAGAT AGATAAACTA TAAATATAGA GTTAAGTATA CTTATGTTTG GTTATCTTCG AATATTACCC   
  
  
+ GTTCGGATAT ATTATCTGAA CTGGTGAAAT AAGTAATATG TTTTGTTGTT TTAATTAGAT AATTTTTAGA   
  
  
+ CCGAGCTTGT GAATATATAC TAGACAAACA TTTATATTTC GAGTCTGCAC TTATATTCTA TAAGAGCTTG   
  
  
+ ATATATTAGA TTTGAACACT AACCTGTTAA TATAGTTTGC CGGTGATTTT TTTTCAAAAT TTTGATTCTT   
  
  
+ AGATATGTAT ATGGAGTAAA ACTAATTTTT ACAGATGCCC ATTTTTTTAA TTGACACTTA TGTAATTAAC   
  
  
+ TGAATTCATA AAACAAGGTT TTTTAAAAAA ATTTAACTCA TATCAATGAA ACAAAGACGA GAACGAAAGC   
  
  
+ ACAATTCTAT GGAAATGGAA AATGAAGTCA CTTATGGAGA TTCAATAGTA AGCAAATCGA GAGCAGAAAA   
  
  
+ TCTAATCTCC TTTCGTCATT ATACAATCAA TATTGCCTAT TTGGTTTTAG TGATTTGTTT CAGCCGCAAA   
  
  
+ ACTTAATTTT CTTTGGTGCA TATGAAATCT TAAAAAGAAT TAAAATAAGA TATAATACGT TAACTCTTCA   
  
  
+ ACAACATGAT ATATTTAAGA TACCAATATT TGTATTCATC ATATAAAAAT TGTAGTGTTG CAAAATATTA   
  
  
+ AAATTATTTC ATAAATAAAC ATTATTATAA GAACTGACTC CGCGGATTAT CATATGGTAT AGATTACAGA   
  
  
+ GTGGGTGGGT TTAAATAATT TCCCCGACAC ATTATACTTT TAGAAGAGCT ATTAGCTCCA AATTATTTAA   
  
  
+ ACAATGTTCT AAAGAGCAAA AAAATCAATG TTTTGGATTT TGATCCGACC GAGAGGACTT GTCCGACCAT   
  
  
+ TCCATATTAA AATGTTCCTA GTTCTGATTA GCTAATACCA   

- TATTTCAACA GCACTAAAGA ACGGGAAATA AAAAATAAAT ATAATTGTAT ATAAAAATTT TTTTTTAATT   
  
  
- TCTGCAAGGA TTGGCTTTGA GTACATAAAA AACTAATACT TATACTTATA GGGGAGATAT AATTAGTACC   
  
  
- TCGTAATGTT GTACAAAAGC ATCGGTATAC AGTAGTGCTC TTACTAAAAA TCTTAACAAT CTTTTTATTT   
  
  
- AACTAAGTAT ATTTGTATAT GATACAAAAA ATAATTTGAT TGATAGTTTA ATTAATTATC ACATGTTTTT   
  
  
- TTATAAAAAA AGAAAGGAAT TTATTTTTGA TGCCTTAATG GATTATACCG ATTGTATATA TACTGTTAAT   
  
  
- TACTAATACT TATTATGTAT AAACTATTTT TTTAAAGATT GGAGAGAGAA AAAACAAATT AAAATATAAT   
  
  
- AATTTCCTTT AAATTGTTAG TGTAATTAGT ATATTATTTT TGTTAATCTA AAAAAGAATA TACAATATAA   
  
  
- AACTTAAAAA TTTTTGCTGA TATTTAATGA TTTTTACTAT TCTCAGGGTG TAATTTTTTA AACACTAGTT   
  
  
- GGCAAATTGA AAAAAAAATC AAGTTCGTTC TATGTTTACT AGTATATAGA CTATATCTGC ACCCGCAAGC   
  
  
- CTATGTGCAA GCCCAAACAT AGTCTATAAA GTCATATTTC CATATCTTGG GCAAGCCCAT AAAGATGTGA   
  
  
- GGCTCAGCCC AAGCCCAAGC CTATAAAACC TAGCCCAAGC TTATAAATTT AAAACTTCTT TTTTTCTTTA   
  
  
- ATAAGTGACA AATTCAAAAA ATATAAATTT ATATAGAATT GAATTGACTA AAAAAAATCA AAAATTTTCT   
  
  
- AATTTTATAA TTATACAAAC CTCTATTTTG AAATTTTTAT CTTTCTGTGA TTAAATCAAA AACAAAACTT   
  
  
- TTAAATCTAC GTTGAAAACA ATTACGTTCT TTGTTCTTGA ACTATACATA AAATTCACTC ATTGTTTACT   
  
  
- AAAACAGATA TCAATATACA TATAATAGAT TAAAACTCAT TATTCTTAGT AATTATATTT ATAAAACTTA   
  
  
- TTTTAATCTA TCTATTTGAT ATTTATATCT CAATTCATAT GAATACAAAC CAATAGAAGC TTATAATGGG   
  
  
- CAAGCCTATA TAATAGACTT GACCACTTTA TTCATTATAC AAAACAACAA AATTAATCTA TTAAAAATCT   
  
  
- GGCTCGAACA CTTATATATG ATCTGTTTGT AAATATAAAG CTCAGACGTG AATATAAGAT ATTCTCGAAC   
  
  
- TATATAATCT AAACTTGTGA TTGGACAATT ATATCAAACG GCCACTAAAA AAAAGTTTTA AAACTAAGAA   
  
  
- TCTATACATA TACCTCATTT TGATTAAAAA TGTCTACGGG TAAAAAAATT AACTGTGAAT ACATTAATTG   
  
  
- ACTTAAGTAT TTTGTTCCAA AAAATTTTTT TAAATTGAGT ATAGTTACTT TGTTTCTGCT CTTGCTTTCG   
  
  
- TGTTAAGATA CCTTTACCTT TTACTTCAGT GAATACCTCT AAGTTATCAT TCGTTTAGCT CTCGTCTTTT   
  
  
- AGATTAGAGG AAAGCAGTAA TATGTTAGTT ATAACGGATA AACCAAAATC ACTAAACAAA GTCGGCGTTT   
  
  
- TGAATTAAAA GAAACCACGT ATACTTTAGA ATTTTTCTTA ATTTTATTCT ATATTATGCA ATTGAGAAGT   
  
  
- TGTTGTACTA TATAAATTCT ATGGTTATAA ACATAAGTAG TATATTTTTA ACATCACAAC GTTTTATAAT   
  
  
- TTTAATAAAG TATTTATTTG TAATAATATT CTTGACTGAG GCGCCTAATA GTATACCATA TCTAATGTCT   
  
  
- CACCCACCCA AATTTATTAA AGGGGCTGTG TAATATGAAA ATCTTCTCGA TAATCGAGGT TTAATAAATT   
  
  
- TGTTACAAGA TTTCTCGTTT TTTTAGTTAC AAAACCTAAA ACTAGGCTGG CTCTCCTGAA CAGGCTGGTA   
  
  
- AGGTATAATT TTACAAGGAT CAAGACTAAT CGATTATGGT

+     Unnamed\_\_6

| Site Name | Organism | Position | Strand | Matrix score. | sequence | function |
| --- | --- | --- | --- | --- | --- | --- |
| Unnamed\_\_6 | Zea mays | 1035 | + | 10 | taTAAATATct |  |
| Unnamed\_\_6 | Zea mays | 1331 | - | 10 | taTAAATATct |  |

>PlantCARE\_25378   
+ ATAAAGTTGT CGTGATTTCT TGCCCTTTAT TTTTTATTTA TATTAACATA TATTTTTAAA AAAAAATTAA   
  
  
+ AGACGTTCCT AACCGAAACT CATGTATTTT TTGATTATGA ATATGAATAT CCCCTCTATA TTAATCATGG   
  
  
+ AGCATTACAA CATGTTTTCG TAGCCATATG TCATCACGAG AATGATTTTT AGAATTGTTA GAAAAATAAA   
  
  
+ TTGATTCATA TAAACATATA CTATGTTTTT TATTAAACTA ACTATCAAAT TAATTAATAG TGTACAAAAA   
  
  
+ AATATTTTTT TCTTTCCTTA AATAAAAACT ACGGAATTAC CTAATATGGC TAACATATAT ATGACAATTA   
  
  
+ ATGATTATGA ATAATACATA TTTGATAAAA AAATTTCTAA CCTCTCTCTT TTTTGTTTAA TTTTATATTA   
  
  
+ TTAAAGGAAA TTTAACAATC ACATTAATCA TATAATAAAA ACAATTAGAT TTTTTCTTAT ATGTTATATT   
  
  
+ TTGAATTTTT AAAAACGACT ATAAATTACT AAAAATGATA AGAGTCCCAC ATTAAAAAAT TTGTGATCAA   
  
  
+ CCGTTTAACT TTTTTTTTAG TTCAAGCAAG ATACAAATGA TCATATATCT GATATAGACG TGGGCGTTCG   
  
  
+ GATACACGTT CGGGTTTGTA TCAGATATTT CAGTATAAAG GTATAGAACC CGTTCGGGTA TTTCTACACT   
  
  
+ CCGAGTCGGG TTCGGGTTCG GATATTTTGG ATCGGGTTCG AATATTTAAA TTTTGAAGAA AAAAAGAAAT   
  
  
+ TATTCACTGT TTAAGTTTTT TATATTTAAA TATATCTTAA CTTAACTGAT TTTTTTTAGT TTTTAAAAGA   
  
  
+ TTAAAATATT AATATGTTTG GAGATAAAAC TTTAAAAATA GAAAGACACT AATTTAGTTT TTGTTTTGAA   
  
  
+ AATTTAGATG CAACTTTTGT TAATGCAAGA AACAAGAACT TGATATGTAT TTTAAGTGAG TAACAAATGA   
  
  
+ TTTTGTCTAT AGTTATATGT ATATTATCTA ATTTTGAGTA ATAAGAATCA TTAATATAAA TATTTTGAAT   
  
  
+ AAAATTAGAT AGATAAACTA TAAATATAGA GTTAAGTATA CTTATGTTTG GTTATCTTCG AATATTACCC   
  
  
+ GTTCGGATAT ATTATCTGAA CTGGTGAAAT AAGTAATATG TTTTGTTGTT TTAATTAGAT AATTTTTAGA   
  
  
+ CCGAGCTTGT GAATATATAC TAGACAAACA TTTATATTTC GAGTCTGCAC TTATATTCTA TAAGAGCTTG   
  
  
+ ATATATTAGA TTTGAACACT AACCTGTTAA TATAGTTTGC CGGTGATTTT TTTTCAAAAT TTTGATTCTT   
  
  
+ AGATATGTAT ATGGAGTAAA ACTAATTTTT ACAGATGCCC ATTTTTTTAA TTGACACTTA TGTAATTAAC   
  
  
+ TGAATTCATA AAACAAGGTT TTTTAAAAAA ATTTAACTCA TATCAATGAA ACAAAGACGA GAACGAAAGC   
  
  
+ ACAATTCTAT GGAAATGGAA AATGAAGTCA CTTATGGAGA TTCAATAGTA AGCAAATCGA GAGCAGAAAA   
  
  
+ TCTAATCTCC TTTCGTCATT ATACAATCAA TATTGCCTAT TTGGTTTTAG TGATTTGTTT CAGCCGCAAA   
  
  
+ ACTTAATTTT CTTTGGTGCA TATGAAATCT TAAAAAGAAT TAAAATAAGA TATAATACGT TAACTCTTCA   
  
  
+ ACAACATGAT ATATTTAAGA TACCAATATT TGTATTCATC ATATAAAAAT TGTAGTGTTG CAAAATATTA   
  
  
+ AAATTATTTC ATAAATAAAC ATTATTATAA GAACTGACTC CGCGGATTAT CATATGGTAT AGATTACAGA   
  
  
+ GTGGGTGGGT TTAAATAATT TCCCCGACAC ATTATACTTT TAGAAGAGCT ATTAGCTCCA AATTATTTAA   
  
  
+ ACAATGTTCT AAAGAGCAAA AAAATCAATG TTTTGGATTT TGATCCGACC GAGAGGACTT GTCCGACCAT   
  
  
+ TCCATATTAA AATGTTCCTA GTTCTGATTA GCTAATACCA   

- TATTTCAACA GCACTAAAGA ACGGGAAATA AAAAATAAAT ATAATTGTAT ATAAAAATTT TTTTTTAATT   
  
  
- TCTGCAAGGA TTGGCTTTGA GTACATAAAA AACTAATACT TATACTTATA GGGGAGATAT AATTAGTACC   
  
  
- TCGTAATGTT GTACAAAAGC ATCGGTATAC AGTAGTGCTC TTACTAAAAA TCTTAACAAT CTTTTTATTT   
  
  
- AACTAAGTAT ATTTGTATAT GATACAAAAA ATAATTTGAT TGATAGTTTA ATTAATTATC ACATGTTTTT   
  
  
- TTATAAAAAA AGAAAGGAAT TTATTTTTGA TGCCTTAATG GATTATACCG ATTGTATATA TACTGTTAAT   
  
  
- TACTAATACT TATTATGTAT AAACTATTTT TTTAAAGATT GGAGAGAGAA AAAACAAATT AAAATATAAT   
  
  
- AATTTCCTTT AAATTGTTAG TGTAATTAGT ATATTATTTT TGTTAATCTA AAAAAGAATA TACAATATAA   
  
  
- AACTTAAAAA TTTTTGCTGA TATTTAATGA TTTTTACTAT TCTCAGGGTG TAATTTTTTA AACACTAGTT   
  
  
- GGCAAATTGA AAAAAAAATC AAGTTCGTTC TATGTTTACT AGTATATAGA CTATATCTGC ACCCGCAAGC   
  
  
- CTATGTGCAA GCCCAAACAT AGTCTATAAA GTCATATTTC CATATCTTGG GCAAGCCCAT AAAGATGTGA   
  
  
- GGCTCAGCCC AAGCCCAAGC CTATAAAACC TAGCCCAAGC TTATAAATTT AAAACTTCTT TTTTTCTTTA   
  
  
- ATAAGTGACA AATTCAAAAA ATATAAATTT ATATAGAATT GAATTGACTA AAAAAAATCA AAAATTTTCT   
  
  
- AATTTTATAA TTATACAAAC CTCTATTTTG AAATTTTTAT CTTTCTGTGA TTAAATCAAA AACAAAACTT   
  
  
- TTAAATCTAC GTTGAAAACA ATTACGTTCT TTGTTCTTGA ACTATACATA AAATTCACTC ATTGTTTACT   
  
  
- AAAACAGATA TCAATATACA TATAATAGAT TAAAACTCAT TATTCTTAGT AATTATATTT ATAAAACTTA   
  
  
- TTTTAATCTA TCTATTTGAT ATTTATATCT CAATTCATAT GAATACAAAC CAATAGAAGC TTATAATGGG   
  
  
- CAAGCCTATA TAATAGACTT GACCACTTTA TTCATTATAC AAAACAACAA AATTAATCTA TTAAAAATCT   
  
  
- GGCTCGAACA CTTATATATG ATCTGTTTGT AAATATAAAG CTCAGACGTG AATATAAGAT ATTCTCGAAC   
  
  
- TATATAATCT AAACTTGTGA TTGGACAATT ATATCAAACG GCCACTAAAA AAAAGTTTTA AAACTAAGAA   
  
  
- TCTATACATA TACCTCATTT TGATTAAAAA TGTCTACGGG TAAAAAAATT AACTGTGAAT ACATTAATTG   
  
  
- ACTTAAGTAT TTTGTTCCAA AAAATTTTTT TAAATTGAGT ATAGTTACTT TGTTTCTGCT CTTGCTTTCG   
  
  
- TGTTAAGATA CCTTTACCTT TTACTTCAGT GAATACCTCT AAGTTATCAT TCGTTTAGCT CTCGTCTTTT   
  
  
- AGATTAGAGG AAAGCAGTAA TATGTTAGTT ATAACGGATA AACCAAAATC ACTAAACAAA GTCGGCGTTT   
  
  
- TGAATTAAAA GAAACCACGT ATACTTTAGA ATTTTTCTTA ATTTTATTCT ATATTATGCA ATTGAGAAGT   
  
  
- TGTTGTACTA TATAAATTCT ATGGTTATAA ACATAAGTAG TATATTTTTA ACATCACAAC GTTTTATAAT   
  
  
- TTTAATAAAG TATTTATTTG TAATAATATT CTTGACTGAG GCGCCTAATA GTATACCATA TCTAATGTCT   
  
  
- CACCCACCCA AATTTATTAA AGGGGCTGTG TAATATGAAA ATCTTCTCGA TAATCGAGGT TTAATAAATT   
  
  
- TGTTACAAGA TTTCTCGTTT TTTTAGTTAC AAAACCTAAA ACTAGGCTGG CTCTCCTGAA CAGGCTGGTA   
  
  
- AGGTATAATT TTACAAGGAT CAAGACTAAT CGATTATGGT

+     WUN-motif

| Site Name | Organism | Position | Strand | Matrix score. | sequence | function |
| --- | --- | --- | --- | --- | --- | --- |
| WUN-motif | Brassica oleracea | 425 | - | 9 | AAATTTCCT | wound-responsive element |
| WUN-motif | Nicotiana glutinosa | 513 | + | 9 | AAATTACTA |  |

>PlantCARE\_25378   
+ ATAAAGTTGT CGTGATTTCT TGCCCTTTAT TTTTTATTTA TATTAACATA TATTTTTAAA AAAAAATTAA   
  
  
+ AGACGTTCCT AACCGAAACT CATGTATTTT TTGATTATGA ATATGAATAT CCCCTCTATA TTAATCATGG   
  
  
+ AGCATTACAA CATGTTTTCG TAGCCATATG TCATCACGAG AATGATTTTT AGAATTGTTA GAAAAATAAA   
  
  
+ TTGATTCATA TAAACATATA CTATGTTTTT TATTAAACTA ACTATCAAAT TAATTAATAG TGTACAAAAA   
  
  
+ AATATTTTTT TCTTTCCTTA AATAAAAACT ACGGAATTAC CTAATATGGC TAACATATAT ATGACAATTA   
  
  
+ ATGATTATGA ATAATACATA TTTGATAAAA AAATTTCTAA CCTCTCTCTT TTTTGTTTAA TTTTATATTA   
  
  
+ TTAAAGGAAA TTTAACAATC ACATTAATCA TATAATAAAA ACAATTAGAT TTTTTCTTAT ATGTTATATT   
  
  
+ TTGAATTTTT AAAAACGACT ATAAATTACT AAAAATGATA AGAGTCCCAC ATTAAAAAAT TTGTGATCAA   
  
  
+ CCGTTTAACT TTTTTTTTAG TTCAAGCAAG ATACAAATGA TCATATATCT GATATAGACG TGGGCGTTCG   
  
  
+ GATACACGTT CGGGTTTGTA TCAGATATTT CAGTATAAAG GTATAGAACC CGTTCGGGTA TTTCTACACT   
  
  
+ CCGAGTCGGG TTCGGGTTCG GATATTTTGG ATCGGGTTCG AATATTTAAA TTTTGAAGAA AAAAAGAAAT   
  
  
+ TATTCACTGT TTAAGTTTTT TATATTTAAA TATATCTTAA CTTAACTGAT TTTTTTTAGT TTTTAAAAGA   
  
  
+ TTAAAATATT AATATGTTTG GAGATAAAAC TTTAAAAATA GAAAGACACT AATTTAGTTT TTGTTTTGAA   
  
  
+ AATTTAGATG CAACTTTTGT TAATGCAAGA AACAAGAACT TGATATGTAT TTTAAGTGAG TAACAAATGA   
  
  
+ TTTTGTCTAT AGTTATATGT ATATTATCTA ATTTTGAGTA ATAAGAATCA TTAATATAAA TATTTTGAAT   
  
  
+ AAAATTAGAT AGATAAACTA TAAATATAGA GTTAAGTATA CTTATGTTTG GTTATCTTCG AATATTACCC   
  
  
+ GTTCGGATAT ATTATCTGAA CTGGTGAAAT AAGTAATATG TTTTGTTGTT TTAATTAGAT AATTTTTAGA   
  
  
+ CCGAGCTTGT GAATATATAC TAGACAAACA TTTATATTTC GAGTCTGCAC TTATATTCTA TAAGAGCTTG   
  
  
+ ATATATTAGA TTTGAACACT AACCTGTTAA TATAGTTTGC CGGTGATTTT TTTTCAAAAT TTTGATTCTT   
  
  
+ AGATATGTAT ATGGAGTAAA ACTAATTTTT ACAGATGCCC ATTTTTTTAA TTGACACTTA TGTAATTAAC   
  
  
+ TGAATTCATA AAACAAGGTT TTTTAAAAAA ATTTAACTCA TATCAATGAA ACAAAGACGA GAACGAAAGC   
  
  
+ ACAATTCTAT GGAAATGGAA AATGAAGTCA CTTATGGAGA TTCAATAGTA AGCAAATCGA GAGCAGAAAA   
  
  
+ TCTAATCTCC TTTCGTCATT ATACAATCAA TATTGCCTAT TTGGTTTTAG TGATTTGTTT CAGCCGCAAA   
  
  
+ ACTTAATTTT CTTTGGTGCA TATGAAATCT TAAAAAGAAT TAAAATAAGA TATAATACGT TAACTCTTCA   
  
  
+ ACAACATGAT ATATTTAAGA TACCAATATT TGTATTCATC ATATAAAAAT TGTAGTGTTG CAAAATATTA   
  
  
+ AAATTATTTC ATAAATAAAC ATTATTATAA GAACTGACTC CGCGGATTAT CATATGGTAT AGATTACAGA   
  
  
+ GTGGGTGGGT TTAAATAATT TCCCCGACAC ATTATACTTT TAGAAGAGCT ATTAGCTCCA AATTATTTAA   
  
  
+ ACAATGTTCT AAAGAGCAAA AAAATCAATG TTTTGGATTT TGATCCGACC GAGAGGACTT GTCCGACCAT   
  
  
+ TCCATATTAA AATGTTCCTA GTTCTGATTA GCTAATACCA   

- TATTTCAACA GCACTAAAGA ACGGGAAATA AAAAATAAAT ATAATTGTAT ATAAAAATTT TTTTTTAATT   
  
  
- TCTGCAAGGA TTGGCTTTGA GTACATAAAA AACTAATACT TATACTTATA GGGGAGATAT AATTAGTACC   
  
  
- TCGTAATGTT GTACAAAAGC ATCGGTATAC AGTAGTGCTC TTACTAAAAA TCTTAACAAT CTTTTTATTT   
  
  
- AACTAAGTAT ATTTGTATAT GATACAAAAA ATAATTTGAT TGATAGTTTA ATTAATTATC ACATGTTTTT   
  
  
- TTATAAAAAA AGAAAGGAAT TTATTTTTGA TGCCTTAATG GATTATACCG ATTGTATATA TACTGTTAAT   
  
  
- TACTAATACT TATTATGTAT AAACTATTTT TTTAAAGATT GGAGAGAGAA AAAACAAATT AAAATATAAT   
  
  
- AATTTCCTTT AAATTGTTAG TGTAATTAGT ATATTATTTT TGTTAATCTA AAAAAGAATA TACAATATAA   
  
  
- AACTTAAAAA TTTTTGCTGA TATTTAATGA TTTTTACTAT TCTCAGGGTG TAATTTTTTA AACACTAGTT   
  
  
- GGCAAATTGA AAAAAAAATC AAGTTCGTTC TATGTTTACT AGTATATAGA CTATATCTGC ACCCGCAAGC   
  
  
- CTATGTGCAA GCCCAAACAT AGTCTATAAA GTCATATTTC CATATCTTGG GCAAGCCCAT AAAGATGTGA   
  
  
- GGCTCAGCCC AAGCCCAAGC CTATAAAACC TAGCCCAAGC TTATAAATTT AAAACTTCTT TTTTTCTTTA   
  
  
- ATAAGTGACA AATTCAAAAA ATATAAATTT ATATAGAATT GAATTGACTA AAAAAAATCA AAAATTTTCT   
  
  
- AATTTTATAA TTATACAAAC CTCTATTTTG AAATTTTTAT CTTTCTGTGA TTAAATCAAA AACAAAACTT   
  
  
- TTAAATCTAC GTTGAAAACA ATTACGTTCT TTGTTCTTGA ACTATACATA AAATTCACTC ATTGTTTACT   
  
  
- AAAACAGATA TCAATATACA TATAATAGAT TAAAACTCAT TATTCTTAGT AATTATATTT ATAAAACTTA   
  
  
- TTTTAATCTA TCTATTTGAT ATTTATATCT CAATTCATAT GAATACAAAC CAATAGAAGC TTATAATGGG   
  
  
- CAAGCCTATA TAATAGACTT GACCACTTTA TTCATTATAC AAAACAACAA AATTAATCTA TTAAAAATCT   
  
  
- GGCTCGAACA CTTATATATG ATCTGTTTGT AAATATAAAG CTCAGACGTG AATATAAGAT ATTCTCGAAC   
  
  
- TATATAATCT AAACTTGTGA TTGGACAATT ATATCAAACG GCCACTAAAA AAAAGTTTTA AAACTAAGAA   
  
  
- TCTATACATA TACCTCATTT TGATTAAAAA TGTCTACGGG TAAAAAAATT AACTGTGAAT ACATTAATTG   
  
  
- ACTTAAGTAT TTTGTTCCAA AAAATTTTTT TAAATTGAGT ATAGTTACTT TGTTTCTGCT CTTGCTTTCG   
  
  
- TGTTAAGATA CCTTTACCTT TTACTTCAGT GAATACCTCT AAGTTATCAT TCGTTTAGCT CTCGTCTTTT   
  
  
- AGATTAGAGG AAAGCAGTAA TATGTTAGTT ATAACGGATA AACCAAAATC ACTAAACAAA GTCGGCGTTT   
  
  
- TGAATTAAAA GAAACCACGT ATACTTTAGA ATTTTTCTTA ATTTTATTCT ATATTATGCA ATTGAGAAGT   
  
  
- TGTTGTACTA TATAAATTCT ATGGTTATAA ACATAAGTAG TATATTTTTA ACATCACAAC GTTTTATAAT   
  
  
- TTTAATAAAG TATTTATTTG TAATAATATT CTTGACTGAG GCGCCTAATA GTATACCATA TCTAATGTCT   
  
  
- CACCCACCCA AATTTATTAA AGGGGCTGTG TAATATGAAA ATCTTCTCGA TAATCGAGGT TTAATAAATT   
  
  
- TGTTACAAGA TTTCTCGTTT TTTTAGTTAC AAAACCTAAA ACTAGGCTGG CTCTCCTGAA CAGGCTGGTA   
  
  
- AGGTATAATT TTACAAGGAT CAAGACTAAT CGATTATGGT

+     as-1

| Site Name | Organism | Position | Strand | Matrix score. | sequence | function |
| --- | --- | --- | --- | --- | --- | --- |
| as-1 | Arabidopsis thaliana | 1554 | - | 5 | TGACG |  |

>PlantCARE\_25378   
+ ATAAAGTTGT CGTGATTTCT TGCCCTTTAT TTTTTATTTA TATTAACATA TATTTTTAAA AAAAAATTAA   
  
  
+ AGACGTTCCT AACCGAAACT CATGTATTTT TTGATTATGA ATATGAATAT CCCCTCTATA TTAATCATGG   
  
  
+ AGCATTACAA CATGTTTTCG TAGCCATATG TCATCACGAG AATGATTTTT AGAATTGTTA GAAAAATAAA   
  
  
+ TTGATTCATA TAAACATATA CTATGTTTTT TATTAAACTA ACTATCAAAT TAATTAATAG TGTACAAAAA   
  
  
+ AATATTTTTT TCTTTCCTTA AATAAAAACT ACGGAATTAC CTAATATGGC TAACATATAT ATGACAATTA   
  
  
+ ATGATTATGA ATAATACATA TTTGATAAAA AAATTTCTAA CCTCTCTCTT TTTTGTTTAA TTTTATATTA   
  
  
+ TTAAAGGAAA TTTAACAATC ACATTAATCA TATAATAAAA ACAATTAGAT TTTTTCTTAT ATGTTATATT   
  
  
+ TTGAATTTTT AAAAACGACT ATAAATTACT AAAAATGATA AGAGTCCCAC ATTAAAAAAT TTGTGATCAA   
  
  
+ CCGTTTAACT TTTTTTTTAG TTCAAGCAAG ATACAAATGA TCATATATCT GATATAGACG TGGGCGTTCG   
  
  
+ GATACACGTT CGGGTTTGTA TCAGATATTT CAGTATAAAG GTATAGAACC CGTTCGGGTA TTTCTACACT   
  
  
+ CCGAGTCGGG TTCGGGTTCG GATATTTTGG ATCGGGTTCG AATATTTAAA TTTTGAAGAA AAAAAGAAAT   
  
  
+ TATTCACTGT TTAAGTTTTT TATATTTAAA TATATCTTAA CTTAACTGAT TTTTTTTAGT TTTTAAAAGA   
  
  
+ TTAAAATATT AATATGTTTG GAGATAAAAC TTTAAAAATA GAAAGACACT AATTTAGTTT TTGTTTTGAA   
  
  
+ AATTTAGATG CAACTTTTGT TAATGCAAGA AACAAGAACT TGATATGTAT TTTAAGTGAG TAACAAATGA   
  
  
+ TTTTGTCTAT AGTTATATGT ATATTATCTA ATTTTGAGTA ATAAGAATCA TTAATATAAA TATTTTGAAT   
  
  
+ AAAATTAGAT AGATAAACTA TAAATATAGA GTTAAGTATA CTTATGTTTG GTTATCTTCG AATATTACCC   
  
  
+ GTTCGGATAT ATTATCTGAA CTGGTGAAAT AAGTAATATG TTTTGTTGTT TTAATTAGAT AATTTTTAGA   
  
  
+ CCGAGCTTGT GAATATATAC TAGACAAACA TTTATATTTC GAGTCTGCAC TTATATTCTA TAAGAGCTTG   
  
  
+ ATATATTAGA TTTGAACACT AACCTGTTAA TATAGTTTGC CGGTGATTTT TTTTCAAAAT TTTGATTCTT   
  
  
+ AGATATGTAT ATGGAGTAAA ACTAATTTTT ACAGATGCCC ATTTTTTTAA TTGACACTTA TGTAATTAAC   
  
  
+ TGAATTCATA AAACAAGGTT TTTTAAAAAA ATTTAACTCA TATCAATGAA ACAAAGACGA GAACGAAAGC   
  
  
+ ACAATTCTAT GGAAATGGAA AATGAAGTCA CTTATGGAGA TTCAATAGTA AGCAAATCGA GAGCAGAAAA   
  
  
+ TCTAATCTCC TTTCGTCATT ATACAATCAA TATTGCCTAT TTGGTTTTAG TGATTTGTTT CAGCCGCAAA   
  
  
+ ACTTAATTTT CTTTGGTGCA TATGAAATCT TAAAAAGAAT TAAAATAAGA TATAATACGT TAACTCTTCA   
  
  
+ ACAACATGAT ATATTTAAGA TACCAATATT TGTATTCATC ATATAAAAAT TGTAGTGTTG CAAAATATTA   
  
  
+ AAATTATTTC ATAAATAAAC ATTATTATAA GAACTGACTC CGCGGATTAT CATATGGTAT AGATTACAGA   
  
  
+ GTGGGTGGGT TTAAATAATT TCCCCGACAC ATTATACTTT TAGAAGAGCT ATTAGCTCCA AATTATTTAA   
  
  
+ ACAATGTTCT AAAGAGCAAA AAAATCAATG TTTTGGATTT TGATCCGACC GAGAGGACTT GTCCGACCAT   
  
  
+ TCCATATTAA AATGTTCCTA GTTCTGATTA GCTAATACCA   

- TATTTCAACA GCACTAAAGA ACGGGAAATA AAAAATAAAT ATAATTGTAT ATAAAAATTT TTTTTTAATT   
  
  
- TCTGCAAGGA TTGGCTTTGA GTACATAAAA AACTAATACT TATACTTATA GGGGAGATAT AATTAGTACC   
  
  
- TCGTAATGTT GTACAAAAGC ATCGGTATAC AGTAGTGCTC TTACTAAAAA TCTTAACAAT CTTTTTATTT   
  
  
- AACTAAGTAT ATTTGTATAT GATACAAAAA ATAATTTGAT TGATAGTTTA ATTAATTATC ACATGTTTTT   
  
  
- TTATAAAAAA AGAAAGGAAT TTATTTTTGA TGCCTTAATG GATTATACCG ATTGTATATA TACTGTTAAT   
  
  
- TACTAATACT TATTATGTAT AAACTATTTT TTTAAAGATT GGAGAGAGAA AAAACAAATT AAAATATAAT   
  
  
- AATTTCCTTT AAATTGTTAG TGTAATTAGT ATATTATTTT TGTTAATCTA AAAAAGAATA TACAATATAA   
  
  
- AACTTAAAAA TTTTTGCTGA TATTTAATGA TTTTTACTAT TCTCAGGGTG TAATTTTTTA AACACTAGTT   
  
  
- GGCAAATTGA AAAAAAAATC AAGTTCGTTC TATGTTTACT AGTATATAGA CTATATCTGC ACCCGCAAGC   
  
  
- CTATGTGCAA GCCCAAACAT AGTCTATAAA GTCATATTTC CATATCTTGG GCAAGCCCAT AAAGATGTGA   
  
  
- GGCTCAGCCC AAGCCCAAGC CTATAAAACC TAGCCCAAGC TTATAAATTT AAAACTTCTT TTTTTCTTTA   
  
  
- ATAAGTGACA AATTCAAAAA ATATAAATTT ATATAGAATT GAATTGACTA AAAAAAATCA AAAATTTTCT   
  
  
- AATTTTATAA TTATACAAAC CTCTATTTTG AAATTTTTAT CTTTCTGTGA TTAAATCAAA AACAAAACTT   
  
  
- TTAAATCTAC GTTGAAAACA ATTACGTTCT TTGTTCTTGA ACTATACATA AAATTCACTC ATTGTTTACT   
  
  
- AAAACAGATA TCAATATACA TATAATAGAT TAAAACTCAT TATTCTTAGT AATTATATTT ATAAAACTTA   
  
  
- TTTTAATCTA TCTATTTGAT ATTTATATCT CAATTCATAT GAATACAAAC CAATAGAAGC TTATAATGGG   
  
  
- CAAGCCTATA TAATAGACTT GACCACTTTA TTCATTATAC AAAACAACAA AATTAATCTA TTAAAAATCT   
  
  
- GGCTCGAACA CTTATATATG ATCTGTTTGT AAATATAAAG CTCAGACGTG AATATAAGAT ATTCTCGAAC   
  
  
- TATATAATCT AAACTTGTGA TTGGACAATT ATATCAAACG GCCACTAAAA AAAAGTTTTA AAACTAAGAA   
  
  
- TCTATACATA TACCTCATTT TGATTAAAAA TGTCTACGGG TAAAAAAATT AACTGTGAAT ACATTAATTG   
  
  
- ACTTAAGTAT TTTGTTCCAA AAAATTTTTT TAAATTGAGT ATAGTTACTT TGTTTCTGCT CTTGCTTTCG   
  
  
- TGTTAAGATA CCTTTACCTT TTACTTCAGT GAATACCTCT AAGTTATCAT TCGTTTAGCT CTCGTCTTTT   
  
  
- AGATTAGAGG AAAGCAGTAA TATGTTAGTT ATAACGGATA AACCAAAATC ACTAAACAAA GTCGGCGTTT   
  
  
- TGAATTAAAA GAAACCACGT ATACTTTAGA ATTTTTCTTA ATTTTATTCT ATATTATGCA ATTGAGAAGT   
  
  
- TGTTGTACTA TATAAATTCT ATGGTTATAA ACATAAGTAG TATATTTTTA ACATCACAAC GTTTTATAAT   
  
  
- TTTAATAAAG TATTTATTTG TAATAATATT CTTGACTGAG GCGCCTAATA GTATACCATA TCTAATGTCT   
  
  
- CACCCACCCA AATTTATTAA AGGGGCTGTG TAATATGAAA ATCTTCTCGA TAATCGAGGT TTAATAAATT   
  
  
- TGTTACAAGA TTTCTCGTTT TTTTAGTTAC AAAACCTAAA ACTAGGCTGG CTCTCCTGAA CAGGCTGGTA   
  
  
- AGGTATAATT TTACAAGGAT CAAGACTAAT CGATTATGGT
