# Supplementary material for: Effects of Low Nighttime Temperature on Fatty Acid Content in Developing Seeds from Brassica napus L. Based on RNA-Seq and Metabolome
Source: Plants (Basel). 2023 Jan 10;12(2):325. doi: 10.3390/plants12020325 (PMC9862530; doi:10.3390/plants12020325)
Supplement: Supplementary file 1 [file plants-12-00325-s001.zip › File S2.html]

Content-Type: text/html; charset=ISO-8859-1


PlantCARE


Webmaster Firefox specific output  
To save the result:
click on the frame with the right mouse button and save the source code as a text file with extension .html  
REFERENCE:PlantCARE: a database of plant cis-acting regulatory elements and a portal to tools for in silico analysis of promoter sequences.  
Lescot, M., Déhais, P., Moreau, Y., De Moor, B., Rouzé ,P.,and Rombauts, S.  
Nucleic Acids Res., Database issue(2002), 30(1):325-327.   


---

>PlantCARE\_4926   
+ TAAAGTTGTC GTGATTTCTT GCCCTTTATT TTTTATTTAT ATTAACATAT ATTTTTAAAA AAAAATTAAA   
  
  
+ GACGTTCCTA ACGGAAACTC ATGTATTTTT TGATTATGAA TATGAATATC CCCTCTATAT TAATCATGGA   
  
  
+ GCATTACAAC ATGTTTTCGT AGCCATATGT CATCACGAGA ATGATTTTTA GAATTGTTAG AAAAATAAAT   
  
  
+ TGATTCATAT AAACATATAC TATGTTTTTT ATTAAACTAA CTATCAAATT AATTAATAGT GTACAAAAAA   
  
  
+ ATATTTTTTT CTTTCCTTAA ATAAAAACTA CGGAATTACC TAATATGGCT AACATATATA TGACAATTAA   
  
  
+ TGATTATGAA TAATACATAT TTGATAAAAA AATTTCTAAC CTCTCTCTTT TTTGTTTAAT TTTATATTAT   
  
  
+ TAAAGGAAAT TTAACAATCA CATTAATCAT ATAATAAAAA CAATTAGATT TTTTCTTATA TGTTATATTT   
  
  
+ TGAATTTTTA AAAACGACTA TAAATTACTA AAAATGATAA GAGTCCCACA TTAAAAAATT TGTGATCAAC   
  
  
+ CGTTTAACTT TTTTTTTAGT TCAAGCAAGA TACAAATGAT CATATATCTG ATATAGACGT GGGCGTTCGG   
  
  
+ ATACACGTTC GGGTTTGTAT CAGATATTTC AGTATAAAGG TATAGAACCC GTTCGGGTAT TTCTACACTC   
  
  
+ CGAGTCGGGT TCGGGTTCGG ATATTTTGGA TCGGGTTCGA ATATTTAAAT TTTGAAGAAA AAAAGAAATT   
  
  
+ ATTCACTGTT TAAGTTTTTT ATATTTAAAT ATATCTTAAC TTAACTGATT TTTTTTAGTT TTTAAAAGAT   
  
  
+ TAAAATATTA ATATGTTTGG AGATAAAACT TTAAAAATAG AAAGACACTA ATTTAGTTTT TGTTTTGAAA   
  
  
+ ATTTAGATGC AACTTTTGTT AATGCAAGAA ACAAGAACTT GATATGTATT TTAAGTGAGT AACAAATGAT   
  
  
+ TTTGTCTATA GTTATATGTA TATTATCTAA TTTTGAGTAA TAAGAATCAT TAATATAAAT ATTTTGAATA   
  
  
+ AAATTAGATA GATAAACTAT AAATATAGAG TTAAGTATAC TTATGTTTGG TTATCTTCGA ATATTACCCG   
  
  
+ TTCGGATATA TTATCTGAAC TGGTGAAATA AGTAATATGT TTTGTTGTTT TAATTAGATA ATTTTTAGAC   
  
  
+ CGAGCTTGTG AATATATACT AGACAAACAT TTATATTTCG AGTCTGCACT TATATTCTAT AAGAGCTTGA   
  
  
+ TATATTAGAT TTGAACACTA ACCTGTTAAT ATAGTTTGCC GGTGATTTTT TTTCAAAATT TTGATTCTTA   
  
  
+ GATATGTATA TGGAGTAAAA CTAATTTTTA CAGATGCCCA TTTTTTTAAT TGACACTTAT GTAATTAACT   
  
  
+ GAATTCATAA AACAAGGTTT TTTAAAAAAA TTTAACTCAT ATCAATGAAA CAAAGACGAG AACGAAAGCA   
  
  
+ CAATTCTATG GAAATGGAAA ATGAAGTCAC TTATGGAGAT TCAATAGTAA GCAAATCGAG AGCAGAAAAT   
  
  
+ CTAATCTCCT TTCGTCATTA TACAATCAAT ATTGCCTATT TGGTTTTAGT GATTTGTTTC AGCCGCAAAA   
  
  
+ CTTAATTTTC TTTGGTGCAT ATGAAATCTT AAAAAGAATT AAAATAAGAT ATAATACGTT AACTCTTCAA   
  
  
+ CAACATGATA TATTTAAGAT ACCAATATTT GTATTCATCA TATAAAAATT GTAGTGTTGC AAAATATTAA   
  
  
+ AATTATTTCA TAAATAAACA TTATTATAAG AACTGACTCC GCGGATTATC ATATGGTATA GATTACAGAG   
  
  
+ TGGGTGGGTT TAAATAATTT CCCCGACACA TTATACTTTT AGAAGAGCTA TTAGCTCCAA ATTATTTAAA   
  
  
+ CAATGTTCTA AAGAGCAAAA AAATCAATGT TTTGGATTTT GATCCGACCG AGAGGACTTG TCCGACCATT   
  
  
+ CCATATTAAA ATGTTCCTAG TTCTGATTAG CTAATACCA  

- ATTTCAACAG CACTAAAGAA CGGGAAATAA AAAATAAATA TAATTGTATA TAAAAATTTT TTTTTAATTT   
  
  
- CTGCAAGGAT TGCCTTTGAG TACATAAAAA ACTAATACTT ATACTTATAG GGGAGATATA ATTAGTACCT   
  
  
- CGTAATGTTG TACAAAAGCA TCGGTATACA GTAGTGCTCT TACTAAAAAT CTTAACAATC TTTTTATTTA   
  
  
- ACTAAGTATA TTTGTATATG ATACAAAAAA TAATTTGATT GATAGTTTAA TTAATTATCA CATGTTTTTT   
  
  
- TATAAAAAAA GAAAGGAATT TATTTTTGAT GCCTTAATGG ATTATACCGA TTGTATATAT ACTGTTAATT   
  
  
- ACTAATACTT ATTATGTATA AACTATTTTT TTAAAGATTG GAGAGAGAAA AAACAAATTA AAATATAATA   
  
  
- ATTTCCTTTA AATTGTTAGT GTAATTAGTA TATTATTTTT GTTAATCTAA AAAAGAATAT ACAATATAAA   
  
  
- ACTTAAAAAT TTTTGCTGAT ATTTAATGAT TTTTACTATT CTCAGGGTGT AATTTTTTAA ACACTAGTTG   
  
  
- GCAAATTGAA AAAAAAATCA AGTTCGTTCT ATGTTTACTA GTATATAGAC TATATCTGCA CCCGCAAGCC   
  
  
- TATGTGCAAG CCCAAACATA GTCTATAAAG TCATATTTCC ATATCTTGGG CAAGCCCATA AAGATGTGAG   
  
  
- GCTCAGCCCA AGCCCAAGCC TATAAAACCT AGCCCAAGCT TATAAATTTA AAACTTCTTT TTTTCTTTAA   
  
  
- TAAGTGACAA ATTCAAAAAA TATAAATTTA TATAGAATTG AATTGACTAA AAAAAATCAA AAATTTTCTA   
  
  
- ATTTTATAAT TATACAAACC TCTATTTTGA AATTTTTATC TTTCTGTGAT TAAATCAAAA ACAAAACTTT   
  
  
- TAAATCTACG TTGAAAACAA TTACGTTCTT TGTTCTTGAA CTATACATAA AATTCACTCA TTGTTTACTA   
  
  
- AAACAGATAT CAATATACAT ATAATAGATT AAAACTCATT ATTCTTAGTA ATTATATTTA TAAAACTTAT   
  
  
- TTTAATCTAT CTATTTGATA TTTATATCTC AATTCATATG AATACAAACC AATAGAAGCT TATAATGGGC   
  
  
- AAGCCTATAT AATAGACTTG ACCACTTTAT TCATTATACA AAACAACAAA ATTAATCTAT TAAAAATCTG   
  
  
- GCTCGAACAC TTATATATGA TCTGTTTGTA AATATAAAGC TCAGACGTGA ATATAAGATA TTCTCGAACT   
  
  
- ATATAATCTA AACTTGTGAT TGGACAATTA TATCAAACGG CCACTAAAAA AAAGTTTTAA AACTAAGAAT   
  
  
- CTATACATAT ACCTCATTTT GATTAAAAAT GTCTACGGGT AAAAAAATTA ACTGTGAATA CATTAATTGA   
  
  
- CTTAAGTATT TTGTTCCAAA AAATTTTTTT AAATTGAGTA TAGTTACTTT GTTTCTGCTC TTGCTTTCGT   
  
  
- GTTAAGATAC CTTTACCTTT TACTTCAGTG AATACCTCTA AGTTATCATT CGTTTAGCTC TCGTCTTTTA   
  
  
- GATTAGAGGA AAGCAGTAAT ATGTTAGTTA TAACGGATAA ACCAAAATCA CTAAACAAAG TCGGCGTTTT   
  
  
- GAATTAAAAG AAACCACGTA TACTTTAGAA TTTTTCTTAA TTTTATTCTA TATTATGCAA TTGAGAAGTT   
  
  
- GTTGTACTAT ATAAATTCTA TGGTTATAAA CATAAGTAGT ATATTTTTAA CATCACAACG TTTTATAATT   
  
  
- TTAATAAAGT ATTTATTTGT AATAATATTC TTGACTGAGG CGCCTAATAG TATACCATAT CTAATGTCTC   
  
  
- ACCCACCCAA ATTTATTAAA GGGGCTGTGT AATATGAAAA TCTTCTCGAT AATCGAGGTT TAATAAATTT   
  
  
- GTTACAAGAT TTCTCGTTTT TTTAGTTACA AAACCTAAAA CTAGGCTGGC TCTCCTGAAC AGGCTGGTAA   
  
  
- GGTATAATTT TACAAGGATC AAGACTAATC GATTATGGT

  
  
Motifs Found  

+   

| Site Name | Organism | Position | Strand | Matrix score. | sequence | function |
| --- | --- | --- | --- | --- | --- | --- |
|  | organism | 859 | - | 4 | motif\_sequence | short\_function |
|  | organism | 1673 | + | 4 | motif\_sequence | short\_function |
|  | organism | 1505 | - | 4 | motif\_sequence | short\_function |
|  | organism | 1893 | + | 4 | motif\_sequence | short\_function |
|  | organism | 1103 | + | 4 | motif\_sequence | short\_function |
|  | organism | 1862 | - | 4 | motif\_sequence | short\_function |
|  | organism | 1544 | + | 4 | motif\_sequence | short\_function |
|  | organism | 1971 | + | 4 | motif\_sequence | short\_function |

>PlantCARE\_4926   
+ TAAAGTTGTC GTGATTTCTT GCCCTTTATT TTTTATTTAT ATTAACATAT ATTTTTAAAA AAAAATTAAA   
  
  
+ GACGTTCCTA ACGGAAACTC ATGTATTTTT TGATTATGAA TATGAATATC CCCTCTATAT TAATCATGGA   
  
  
+ GCATTACAAC ATGTTTTCGT AGCCATATGT CATCACGAGA ATGATTTTTA GAATTGTTAG AAAAATAAAT   
  
  
+ TGATTCATAT AAACATATAC TATGTTTTTT ATTAAACTAA CTATCAAATT AATTAATAGT GTACAAAAAA   
  
  
+ ATATTTTTTT CTTTCCTTAA ATAAAAACTA CGGAATTACC TAATATGGCT AACATATATA TGACAATTAA   
  
  
+ TGATTATGAA TAATACATAT TTGATAAAAA AATTTCTAAC CTCTCTCTTT TTTGTTTAAT TTTATATTAT   
  
  
+ TAAAGGAAAT TTAACAATCA CATTAATCAT ATAATAAAAA CAATTAGATT TTTTCTTATA TGTTATATTT   
  
  
+ TGAATTTTTA AAAACGACTA TAAATTACTA AAAATGATAA GAGTCCCACA TTAAAAAATT TGTGATCAAC   
  
  
+ CGTTTAACTT TTTTTTTAGT TCAAGCAAGA TACAAATGAT CATATATCTG ATATAGACGT GGGCGTTCGG   
  
  
+ ATACACGTTC GGGTTTGTAT CAGATATTTC AGTATAAAGG TATAGAACCC GTTCGGGTAT TTCTACACTC   
  
  
+ CGAGTCGGGT TCGGGTTCGG ATATTTTGGA TCGGGTTCGA ATATTTAAAT TTTGAAGAAA AAAAGAAATT   
  
  
+ ATTCACTGTT TAAGTTTTTT ATATTTAAAT ATATCTTAAC TTAACTGATT TTTTTTAGTT TTTAAAAGAT   
  
  
+ TAAAATATTA ATATGTTTGG AGATAAAACT TTAAAAATAG AAAGACACTA ATTTAGTTTT TGTTTTGAAA   
  
  
+ ATTTAGATGC AACTTTTGTT AATGCAAGAA ACAAGAACTT GATATGTATT TTAAGTGAGT AACAAATGAT   
  
  
+ TTTGTCTATA GTTATATGTA TATTATCTAA TTTTGAGTAA TAAGAATCAT TAATATAAAT ATTTTGAATA   
  
  
+ AAATTAGATA GATAAACTAT AAATATAGAG TTAAGTATAC TTATGTTTGG TTATCTTCGA ATATTACCCG   
  
  
+ TTCGGATATA TTATCTGAAC TGGTGAAATA AGTAATATGT TTTGTTGTTT TAATTAGATA ATTTTTAGAC   
  
  
+ CGAGCTTGTG AATATATACT AGACAAACAT TTATATTTCG AGTCTGCACT TATATTCTAT AAGAGCTTGA   
  
  
+ TATATTAGAT TTGAACACTA ACCTGTTAAT ATAGTTTGCC GGTGATTTTT TTTCAAAATT TTGATTCTTA   
  
  
+ GATATGTATA TGGAGTAAAA CTAATTTTTA CAGATGCCCA TTTTTTTAAT TGACACTTAT GTAATTAACT   
  
  
+ GAATTCATAA AACAAGGTTT TTTAAAAAAA TTTAACTCAT ATCAATGAAA CAAAGACGAG AACGAAAGCA   
  
  
+ CAATTCTATG GAAATGGAAA ATGAAGTCAC TTATGGAGAT TCAATAGTAA GCAAATCGAG AGCAGAAAAT   
  
  
+ CTAATCTCCT TTCGTCATTA TACAATCAAT ATTGCCTATT TGGTTTTAGT GATTTGTTTC AGCCGCAAAA   
  
  
+ CTTAATTTTC TTTGGTGCAT ATGAAATCTT AAAAAGAATT AAAATAAGAT ATAATACGTT AACTCTTCAA   
  
  
+ CAACATGATA TATTTAAGAT ACCAATATTT GTATTCATCA TATAAAAATT GTAGTGTTGC AAAATATTAA   
  
  
+ AATTATTTCA TAAATAAACA TTATTATAAG AACTGACTCC GCGGATTATC ATATGGTATA GATTACAGAG   
  
  
+ TGGGTGGGTT TAAATAATTT CCCCGACACA TTATACTTTT AGAAGAGCTA TTAGCTCCAA ATTATTTAAA   
  
  
+ CAATGTTCTA AAGAGCAAAA AAATCAATGT TTTGGATTTT GATCCGACCG AGAGGACTTG TCCGACCATT   
  
  
+ CCATATTAAA ATGTTCCTAG TTCTGATTAG CTAATACCA  

- ATTTCAACAG CACTAAAGAA CGGGAAATAA AAAATAAATA TAATTGTATA TAAAAATTTT TTTTTAATTT   
  
  
- CTGCAAGGAT TGCCTTTGAG TACATAAAAA ACTAATACTT ATACTTATAG GGGAGATATA ATTAGTACCT   
  
  
- CGTAATGTTG TACAAAAGCA TCGGTATACA GTAGTGCTCT TACTAAAAAT CTTAACAATC TTTTTATTTA   
  
  
- ACTAAGTATA TTTGTATATG ATACAAAAAA TAATTTGATT GATAGTTTAA TTAATTATCA CATGTTTTTT   
  
  
- TATAAAAAAA GAAAGGAATT TATTTTTGAT GCCTTAATGG ATTATACCGA TTGTATATAT ACTGTTAATT   
  
  
- ACTAATACTT ATTATGTATA AACTATTTTT TTAAAGATTG GAGAGAGAAA AAACAAATTA AAATATAATA   
  
  
- ATTTCCTTTA AATTGTTAGT GTAATTAGTA TATTATTTTT GTTAATCTAA AAAAGAATAT ACAATATAAA   
  
  
- ACTTAAAAAT TTTTGCTGAT ATTTAATGAT TTTTACTATT CTCAGGGTGT AATTTTTTAA ACACTAGTTG   
  
  
- GCAAATTGAA AAAAAAATCA AGTTCGTTCT ATGTTTACTA GTATATAGAC TATATCTGCA CCCGCAAGCC   
  
  
- TATGTGCAAG CCCAAACATA GTCTATAAAG TCATATTTCC ATATCTTGGG CAAGCCCATA AAGATGTGAG   
  
  
- GCTCAGCCCA AGCCCAAGCC TATAAAACCT AGCCCAAGCT TATAAATTTA AAACTTCTTT TTTTCTTTAA   
  
  
- TAAGTGACAA ATTCAAAAAA TATAAATTTA TATAGAATTG AATTGACTAA AAAAAATCAA AAATTTTCTA   
  
  
- ATTTTATAAT TATACAAACC TCTATTTTGA AATTTTTATC TTTCTGTGAT TAAATCAAAA ACAAAACTTT   
  
  
- TAAATCTACG TTGAAAACAA TTACGTTCTT TGTTCTTGAA CTATACATAA AATTCACTCA TTGTTTACTA   
  
  
- AAACAGATAT CAATATACAT ATAATAGATT AAAACTCATT ATTCTTAGTA ATTATATTTA TAAAACTTAT   
  
  
- TTTAATCTAT CTATTTGATA TTTATATCTC AATTCATATG AATACAAACC AATAGAAGCT TATAATGGGC   
  
  
- AAGCCTATAT AATAGACTTG ACCACTTTAT TCATTATACA AAACAACAAA ATTAATCTAT TAAAAATCTG   
  
  
- GCTCGAACAC TTATATATGA TCTGTTTGTA AATATAAAGC TCAGACGTGA ATATAAGATA TTCTCGAACT   
  
  
- ATATAATCTA AACTTGTGAT TGGACAATTA TATCAAACGG CCACTAAAAA AAAGTTTTAA AACTAAGAAT   
  
  
- CTATACATAT ACCTCATTTT GATTAAAAAT GTCTACGGGT AAAAAAATTA ACTGTGAATA CATTAATTGA   
  
  
- CTTAAGTATT TTGTTCCAAA AAATTTTTTT AAATTGAGTA TAGTTACTTT GTTTCTGCTC TTGCTTTCGT   
  
  
- GTTAAGATAC CTTTACCTTT TACTTCAGTG AATACCTCTA AGTTATCATT CGTTTAGCTC TCGTCTTTTA   
  
  
- GATTAGAGGA AAGCAGTAAT ATGTTAGTTA TAACGGATAA ACCAAAATCA CTAAACAAAG TCGGCGTTTT   
  
  
- GAATTAAAAG AAACCACGTA TACTTTAGAA TTTTTCTTAA TTTTATTCTA TATTATGCAA TTGAGAAGTT   
  
  
- GTTGTACTAT ATAAATTCTA TGGTTATAAA CATAAGTAGT ATATTTTTAA CATCACAACG TTTTATAATT   
  
  
- TTAATAAAGT ATTTATTTGT AATAATATTC TTGACTGAGG CGCCTAATAG TATACCATAT CTAATGTCTC   
  
  
- ACCCACCCAA ATTTATTAAA GGGGCTGTGT AATATGAAAA TCTTCTCGAT AATCGAGGTT TAATAAATTT   
  
  
- GTTACAAGAT TTCTCGTTTT TTTAGTTACA AAACCTAAAA CTAGGCTGGC TCTCCTGAAC AGGCTGGTAA   
  
  
- GGTATAATTT TACAAGGATC AAGACTAATC GATTATGGT

+     AAGAA-motif

| Site Name | Organism | Position | Strand | Matrix score. | sequence | function |
| --- | --- | --- | --- | --- | --- | --- |
| AAGAA-motif | Avena sativa | 289 | - | 7 | GAAAGAA |  |

>PlantCARE\_4926   
+ TAAAGTTGTC GTGATTTCTT GCCCTTTATT TTTTATTTAT ATTAACATAT ATTTTTAAAA AAAAATTAAA   
  
  
+ GACGTTCCTA ACGGAAACTC ATGTATTTTT TGATTATGAA TATGAATATC CCCTCTATAT TAATCATGGA   
  
  
+ GCATTACAAC ATGTTTTCGT AGCCATATGT CATCACGAGA ATGATTTTTA GAATTGTTAG AAAAATAAAT   
  
  
+ TGATTCATAT AAACATATAC TATGTTTTTT ATTAAACTAA CTATCAAATT AATTAATAGT GTACAAAAAA   
  
  
+ ATATTTTTTT CTTTCCTTAA ATAAAAACTA CGGAATTACC TAATATGGCT AACATATATA TGACAATTAA   
  
  
+ TGATTATGAA TAATACATAT TTGATAAAAA AATTTCTAAC CTCTCTCTTT TTTGTTTAAT TTTATATTAT   
  
  
+ TAAAGGAAAT TTAACAATCA CATTAATCAT ATAATAAAAA CAATTAGATT TTTTCTTATA TGTTATATTT   
  
  
+ TGAATTTTTA AAAACGACTA TAAATTACTA AAAATGATAA GAGTCCCACA TTAAAAAATT TGTGATCAAC   
  
  
+ CGTTTAACTT TTTTTTTAGT TCAAGCAAGA TACAAATGAT CATATATCTG ATATAGACGT GGGCGTTCGG   
  
  
+ ATACACGTTC GGGTTTGTAT CAGATATTTC AGTATAAAGG TATAGAACCC GTTCGGGTAT TTCTACACTC   
  
  
+ CGAGTCGGGT TCGGGTTCGG ATATTTTGGA TCGGGTTCGA ATATTTAAAT TTTGAAGAAA AAAAGAAATT   
  
  
+ ATTCACTGTT TAAGTTTTTT ATATTTAAAT ATATCTTAAC TTAACTGATT TTTTTTAGTT TTTAAAAGAT   
  
  
+ TAAAATATTA ATATGTTTGG AGATAAAACT TTAAAAATAG AAAGACACTA ATTTAGTTTT TGTTTTGAAA   
  
  
+ ATTTAGATGC AACTTTTGTT AATGCAAGAA ACAAGAACTT GATATGTATT TTAAGTGAGT AACAAATGAT   
  
  
+ TTTGTCTATA GTTATATGTA TATTATCTAA TTTTGAGTAA TAAGAATCAT TAATATAAAT ATTTTGAATA   
  
  
+ AAATTAGATA GATAAACTAT AAATATAGAG TTAAGTATAC TTATGTTTGG TTATCTTCGA ATATTACCCG   
  
  
+ TTCGGATATA TTATCTGAAC TGGTGAAATA AGTAATATGT TTTGTTGTTT TAATTAGATA ATTTTTAGAC   
  
  
+ CGAGCTTGTG AATATATACT AGACAAACAT TTATATTTCG AGTCTGCACT TATATTCTAT AAGAGCTTGA   
  
  
+ TATATTAGAT TTGAACACTA ACCTGTTAAT ATAGTTTGCC GGTGATTTTT TTTCAAAATT TTGATTCTTA   
  
  
+ GATATGTATA TGGAGTAAAA CTAATTTTTA CAGATGCCCA TTTTTTTAAT TGACACTTAT GTAATTAACT   
  
  
+ GAATTCATAA AACAAGGTTT TTTAAAAAAA TTTAACTCAT ATCAATGAAA CAAAGACGAG AACGAAAGCA   
  
  
+ CAATTCTATG GAAATGGAAA ATGAAGTCAC TTATGGAGAT TCAATAGTAA GCAAATCGAG AGCAGAAAAT   
  
  
+ CTAATCTCCT TTCGTCATTA TACAATCAAT ATTGCCTATT TGGTTTTAGT GATTTGTTTC AGCCGCAAAA   
  
  
+ CTTAATTTTC TTTGGTGCAT ATGAAATCTT AAAAAGAATT AAAATAAGAT ATAATACGTT AACTCTTCAA   
  
  
+ CAACATGATA TATTTAAGAT ACCAATATTT GTATTCATCA TATAAAAATT GTAGTGTTGC AAAATATTAA   
  
  
+ AATTATTTCA TAAATAAACA TTATTATAAG AACTGACTCC GCGGATTATC ATATGGTATA GATTACAGAG   
  
  
+ TGGGTGGGTT TAAATAATTT CCCCGACACA TTATACTTTT AGAAGAGCTA TTAGCTCCAA ATTATTTAAA   
  
  
+ CAATGTTCTA AAGAGCAAAA AAATCAATGT TTTGGATTTT GATCCGACCG AGAGGACTTG TCCGACCATT   
  
  
+ CCATATTAAA ATGTTCCTAG TTCTGATTAG CTAATACCA  

- ATTTCAACAG CACTAAAGAA CGGGAAATAA AAAATAAATA TAATTGTATA TAAAAATTTT TTTTTAATTT   
  
  
- CTGCAAGGAT TGCCTTTGAG TACATAAAAA ACTAATACTT ATACTTATAG GGGAGATATA ATTAGTACCT   
  
  
- CGTAATGTTG TACAAAAGCA TCGGTATACA GTAGTGCTCT TACTAAAAAT CTTAACAATC TTTTTATTTA   
  
  
- ACTAAGTATA TTTGTATATG ATACAAAAAA TAATTTGATT GATAGTTTAA TTAATTATCA CATGTTTTTT   
  
  
- TATAAAAAAA GAAAGGAATT TATTTTTGAT GCCTTAATGG ATTATACCGA TTGTATATAT ACTGTTAATT   
  
  
- ACTAATACTT ATTATGTATA AACTATTTTT TTAAAGATTG GAGAGAGAAA AAACAAATTA AAATATAATA   
  
  
- ATTTCCTTTA AATTGTTAGT GTAATTAGTA TATTATTTTT GTTAATCTAA AAAAGAATAT ACAATATAAA   
  
  
- ACTTAAAAAT TTTTGCTGAT ATTTAATGAT TTTTACTATT CTCAGGGTGT AATTTTTTAA ACACTAGTTG   
  
  
- GCAAATTGAA AAAAAAATCA AGTTCGTTCT ATGTTTACTA GTATATAGAC TATATCTGCA CCCGCAAGCC   
  
  
- TATGTGCAAG CCCAAACATA GTCTATAAAG TCATATTTCC ATATCTTGGG CAAGCCCATA AAGATGTGAG   
  
  
- GCTCAGCCCA AGCCCAAGCC TATAAAACCT AGCCCAAGCT TATAAATTTA AAACTTCTTT TTTTCTTTAA   
  
  
- TAAGTGACAA ATTCAAAAAA TATAAATTTA TATAGAATTG AATTGACTAA AAAAAATCAA AAATTTTCTA   
  
  
- ATTTTATAAT TATACAAACC TCTATTTTGA AATTTTTATC TTTCTGTGAT TAAATCAAAA ACAAAACTTT   
  
  
- TAAATCTACG TTGAAAACAA TTACGTTCTT TGTTCTTGAA CTATACATAA AATTCACTCA TTGTTTACTA   
  
  
- AAACAGATAT CAATATACAT ATAATAGATT AAAACTCATT ATTCTTAGTA ATTATATTTA TAAAACTTAT   
  
  
- TTTAATCTAT CTATTTGATA TTTATATCTC AATTCATATG AATACAAACC AATAGAAGCT TATAATGGGC   
  
  
- AAGCCTATAT AATAGACTTG ACCACTTTAT TCATTATACA AAACAACAAA ATTAATCTAT TAAAAATCTG   
  
  
- GCTCGAACAC TTATATATGA TCTGTTTGTA AATATAAAGC TCAGACGTGA ATATAAGATA TTCTCGAACT   
  
  
- ATATAATCTA AACTTGTGAT TGGACAATTA TATCAAACGG CCACTAAAAA AAAGTTTTAA AACTAAGAAT   
  
  
- CTATACATAT ACCTCATTTT GATTAAAAAT GTCTACGGGT AAAAAAATTA ACTGTGAATA CATTAATTGA   
  
  
- CTTAAGTATT TTGTTCCAAA AAATTTTTTT AAATTGAGTA TAGTTACTTT GTTTCTGCTC TTGCTTTCGT   
  
  
- GTTAAGATAC CTTTACCTTT TACTTCAGTG AATACCTCTA AGTTATCATT CGTTTAGCTC TCGTCTTTTA   
  
  
- GATTAGAGGA AAGCAGTAAT ATGTTAGTTA TAACGGATAA ACCAAAATCA CTAAACAAAG TCGGCGTTTT   
  
  
- GAATTAAAAG AAACCACGTA TACTTTAGAA TTTTTCTTAA TTTTATTCTA TATTATGCAA TTGAGAAGTT   
  
  
- GTTGTACTAT ATAAATTCTA TGGTTATAAA CATAAGTAGT ATATTTTTAA CATCACAACG TTTTATAATT   
  
  
- TTAATAAAGT ATTTATTTGT AATAATATTC TTGACTGAGG CGCCTAATAG TATACCATAT CTAATGTCTC   
  
  
- ACCCACCCAA ATTTATTAAA GGGGCTGTGT AATATGAAAA TCTTCTCGAT AATCGAGGTT TAATAAATTT   
  
  
- GTTACAAGAT TTCTCGTTTT TTTAGTTACA AAACCTAAAA CTAGGCTGGC TCTCCTGAAC AGGCTGGTAA   
  
  
- GGTATAATTT TACAAGGATC AAGACTAATC GATTATGGT

+     ABRE

| Site Name | Organism | Position | Strand | Matrix score. | sequence | function |
| --- | --- | --- | --- | --- | --- | --- |
| ABRE | Arabidopsis thaliana | 617 | + | 5 | ACGTG | cis-acting element involved in the abscisic acid responsiveness |
| ABRE | Arabidopsis thaliana | 634 | - | 5 | ACGTG | cis-acting element involved in the abscisic acid responsiveness |

>PlantCARE\_4926   
+ TAAAGTTGTC GTGATTTCTT GCCCTTTATT TTTTATTTAT ATTAACATAT ATTTTTAAAA AAAAATTAAA   
  
  
+ GACGTTCCTA ACGGAAACTC ATGTATTTTT TGATTATGAA TATGAATATC CCCTCTATAT TAATCATGGA   
  
  
+ GCATTACAAC ATGTTTTCGT AGCCATATGT CATCACGAGA ATGATTTTTA GAATTGTTAG AAAAATAAAT   
  
  
+ TGATTCATAT AAACATATAC TATGTTTTTT ATTAAACTAA CTATCAAATT AATTAATAGT GTACAAAAAA   
  
  
+ ATATTTTTTT CTTTCCTTAA ATAAAAACTA CGGAATTACC TAATATGGCT AACATATATA TGACAATTAA   
  
  
+ TGATTATGAA TAATACATAT TTGATAAAAA AATTTCTAAC CTCTCTCTTT TTTGTTTAAT TTTATATTAT   
  
  
+ TAAAGGAAAT TTAACAATCA CATTAATCAT ATAATAAAAA CAATTAGATT TTTTCTTATA TGTTATATTT   
  
  
+ TGAATTTTTA AAAACGACTA TAAATTACTA AAAATGATAA GAGTCCCACA TTAAAAAATT TGTGATCAAC   
  
  
+ CGTTTAACTT TTTTTTTAGT TCAAGCAAGA TACAAATGAT CATATATCTG ATATAGACGT GGGCGTTCGG   
  
  
+ ATACACGTTC GGGTTTGTAT CAGATATTTC AGTATAAAGG TATAGAACCC GTTCGGGTAT TTCTACACTC   
  
  
+ CGAGTCGGGT TCGGGTTCGG ATATTTTGGA TCGGGTTCGA ATATTTAAAT TTTGAAGAAA AAAAGAAATT   
  
  
+ ATTCACTGTT TAAGTTTTTT ATATTTAAAT ATATCTTAAC TTAACTGATT TTTTTTAGTT TTTAAAAGAT   
  
  
+ TAAAATATTA ATATGTTTGG AGATAAAACT TTAAAAATAG AAAGACACTA ATTTAGTTTT TGTTTTGAAA   
  
  
+ ATTTAGATGC AACTTTTGTT AATGCAAGAA ACAAGAACTT GATATGTATT TTAAGTGAGT AACAAATGAT   
  
  
+ TTTGTCTATA GTTATATGTA TATTATCTAA TTTTGAGTAA TAAGAATCAT TAATATAAAT ATTTTGAATA   
  
  
+ AAATTAGATA GATAAACTAT AAATATAGAG TTAAGTATAC TTATGTTTGG TTATCTTCGA ATATTACCCG   
  
  
+ TTCGGATATA TTATCTGAAC TGGTGAAATA AGTAATATGT TTTGTTGTTT TAATTAGATA ATTTTTAGAC   
  
  
+ CGAGCTTGTG AATATATACT AGACAAACAT TTATATTTCG AGTCTGCACT TATATTCTAT AAGAGCTTGA   
  
  
+ TATATTAGAT TTGAACACTA ACCTGTTAAT ATAGTTTGCC GGTGATTTTT TTTCAAAATT TTGATTCTTA   
  
  
+ GATATGTATA TGGAGTAAAA CTAATTTTTA CAGATGCCCA TTTTTTTAAT TGACACTTAT GTAATTAACT   
  
  
+ GAATTCATAA AACAAGGTTT TTTAAAAAAA TTTAACTCAT ATCAATGAAA CAAAGACGAG AACGAAAGCA   
  
  
+ CAATTCTATG GAAATGGAAA ATGAAGTCAC TTATGGAGAT TCAATAGTAA GCAAATCGAG AGCAGAAAAT   
  
  
+ CTAATCTCCT TTCGTCATTA TACAATCAAT ATTGCCTATT TGGTTTTAGT GATTTGTTTC AGCCGCAAAA   
  
  
+ CTTAATTTTC TTTGGTGCAT ATGAAATCTT AAAAAGAATT AAAATAAGAT ATAATACGTT AACTCTTCAA   
  
  
+ CAACATGATA TATTTAAGAT ACCAATATTT GTATTCATCA TATAAAAATT GTAGTGTTGC AAAATATTAA   
  
  
+ AATTATTTCA TAAATAAACA TTATTATAAG AACTGACTCC GCGGATTATC ATATGGTATA GATTACAGAG   
  
  
+ TGGGTGGGTT TAAATAATTT CCCCGACACA TTATACTTTT AGAAGAGCTA TTAGCTCCAA ATTATTTAAA   
  
  
+ CAATGTTCTA AAGAGCAAAA AAATCAATGT TTTGGATTTT GATCCGACCG AGAGGACTTG TCCGACCATT   
  
  
+ CCATATTAAA ATGTTCCTAG TTCTGATTAG CTAATACCA  

- ATTTCAACAG CACTAAAGAA CGGGAAATAA AAAATAAATA TAATTGTATA TAAAAATTTT TTTTTAATTT   
  
  
- CTGCAAGGAT TGCCTTTGAG TACATAAAAA ACTAATACTT ATACTTATAG GGGAGATATA ATTAGTACCT   
  
  
- CGTAATGTTG TACAAAAGCA TCGGTATACA GTAGTGCTCT TACTAAAAAT CTTAACAATC TTTTTATTTA   
  
  
- ACTAAGTATA TTTGTATATG ATACAAAAAA TAATTTGATT GATAGTTTAA TTAATTATCA CATGTTTTTT   
  
  
- TATAAAAAAA GAAAGGAATT TATTTTTGAT GCCTTAATGG ATTATACCGA TTGTATATAT ACTGTTAATT   
  
  
- ACTAATACTT ATTATGTATA AACTATTTTT TTAAAGATTG GAGAGAGAAA AAACAAATTA AAATATAATA   
  
  
- ATTTCCTTTA AATTGTTAGT GTAATTAGTA TATTATTTTT GTTAATCTAA AAAAGAATAT ACAATATAAA   
  
  
- ACTTAAAAAT TTTTGCTGAT ATTTAATGAT TTTTACTATT CTCAGGGTGT AATTTTTTAA ACACTAGTTG   
  
  
- GCAAATTGAA AAAAAAATCA AGTTCGTTCT ATGTTTACTA GTATATAGAC TATATCTGCA CCCGCAAGCC   
  
  
- TATGTGCAAG CCCAAACATA GTCTATAAAG TCATATTTCC ATATCTTGGG CAAGCCCATA AAGATGTGAG   
  
  
- GCTCAGCCCA AGCCCAAGCC TATAAAACCT AGCCCAAGCT TATAAATTTA AAACTTCTTT TTTTCTTTAA   
  
  
- TAAGTGACAA ATTCAAAAAA TATAAATTTA TATAGAATTG AATTGACTAA AAAAAATCAA AAATTTTCTA   
  
  
- ATTTTATAAT TATACAAACC TCTATTTTGA AATTTTTATC TTTCTGTGAT TAAATCAAAA ACAAAACTTT   
  
  
- TAAATCTACG TTGAAAACAA TTACGTTCTT TGTTCTTGAA CTATACATAA AATTCACTCA TTGTTTACTA   
  
  
- AAACAGATAT CAATATACAT ATAATAGATT AAAACTCATT ATTCTTAGTA ATTATATTTA TAAAACTTAT   
  
  
- TTTAATCTAT CTATTTGATA TTTATATCTC AATTCATATG AATACAAACC AATAGAAGCT TATAATGGGC   
  
  
- AAGCCTATAT AATAGACTTG ACCACTTTAT TCATTATACA AAACAACAAA ATTAATCTAT TAAAAATCTG   
  
  
- GCTCGAACAC TTATATATGA TCTGTTTGTA AATATAAAGC TCAGACGTGA ATATAAGATA TTCTCGAACT   
  
  
- ATATAATCTA AACTTGTGAT TGGACAATTA TATCAAACGG CCACTAAAAA AAAGTTTTAA AACTAAGAAT   
  
  
- CTATACATAT ACCTCATTTT GATTAAAAAT GTCTACGGGT AAAAAAATTA ACTGTGAATA CATTAATTGA   
  
  
- CTTAAGTATT TTGTTCCAAA AAATTTTTTT AAATTGAGTA TAGTTACTTT GTTTCTGCTC TTGCTTTCGT   
  
  
- GTTAAGATAC CTTTACCTTT TACTTCAGTG AATACCTCTA AGTTATCATT CGTTTAGCTC TCGTCTTTTA   
  
  
- GATTAGAGGA AAGCAGTAAT ATGTTAGTTA TAACGGATAA ACCAAAATCA CTAAACAAAG TCGGCGTTTT   
  
  
- GAATTAAAAG AAACCACGTA TACTTTAGAA TTTTTCTTAA TTTTATTCTA TATTATGCAA TTGAGAAGTT   
  
  
- GTTGTACTAT ATAAATTCTA TGGTTATAAA CATAAGTAGT ATATTTTTAA CATCACAACG TTTTATAATT   
  
  
- TTAATAAAGT ATTTATTTGT AATAATATTC TTGACTGAGG CGCCTAATAG TATACCATAT CTAATGTCTC   
  
  
- ACCCACCCAA ATTTATTAAA GGGGCTGTGT AATATGAAAA TCTTCTCGAT AATCGAGGTT TAATAAATTT   
  
  
- GTTACAAGAT TTCTCGTTTT TTTAGTTACA AAACCTAAAA CTAGGCTGGC TCTCCTGAAC AGGCTGGTAA   
  
  
- GGTATAATTT TACAAGGATC AAGACTAATC GATTATGGT

+     ACE

| Site Name | Organism | Position | Strand | Matrix score. | sequence | function |
| --- | --- | --- | --- | --- | --- | --- |
| ACE | Petroselinum crispum | 1382 | + | 9 | GACACGTATG | cis-acting element involved in light responsiveness |
| ACE | Petroselinum crispum | 1663 | - | 9 | CTAACGTATT | cis-acting element involved in light responsiveness |

>PlantCARE\_4926   
+ TAAAGTTGTC GTGATTTCTT GCCCTTTATT TTTTATTTAT ATTAACATAT ATTTTTAAAA AAAAATTAAA   
  
  
+ GACGTTCCTA ACGGAAACTC ATGTATTTTT TGATTATGAA TATGAATATC CCCTCTATAT TAATCATGGA   
  
  
+ GCATTACAAC ATGTTTTCGT AGCCATATGT CATCACGAGA ATGATTTTTA GAATTGTTAG AAAAATAAAT   
  
  
+ TGATTCATAT AAACATATAC TATGTTTTTT ATTAAACTAA CTATCAAATT AATTAATAGT GTACAAAAAA   
  
  
+ ATATTTTTTT CTTTCCTTAA ATAAAAACTA CGGAATTACC TAATATGGCT AACATATATA TGACAATTAA   
  
  
+ TGATTATGAA TAATACATAT TTGATAAAAA AATTTCTAAC CTCTCTCTTT TTTGTTTAAT TTTATATTAT   
  
  
+ TAAAGGAAAT TTAACAATCA CATTAATCAT ATAATAAAAA CAATTAGATT TTTTCTTATA TGTTATATTT   
  
  
+ TGAATTTTTA AAAACGACTA TAAATTACTA AAAATGATAA GAGTCCCACA TTAAAAAATT TGTGATCAAC   
  
  
+ CGTTTAACTT TTTTTTTAGT TCAAGCAAGA TACAAATGAT CATATATCTG ATATAGACGT GGGCGTTCGG   
  
  
+ ATACACGTTC GGGTTTGTAT CAGATATTTC AGTATAAAGG TATAGAACCC GTTCGGGTAT TTCTACACTC   
  
  
+ CGAGTCGGGT TCGGGTTCGG ATATTTTGGA TCGGGTTCGA ATATTTAAAT TTTGAAGAAA AAAAGAAATT   
  
  
+ ATTCACTGTT TAAGTTTTTT ATATTTAAAT ATATCTTAAC TTAACTGATT TTTTTTAGTT TTTAAAAGAT   
  
  
+ TAAAATATTA ATATGTTTGG AGATAAAACT TTAAAAATAG AAAGACACTA ATTTAGTTTT TGTTTTGAAA   
  
  
+ ATTTAGATGC AACTTTTGTT AATGCAAGAA ACAAGAACTT GATATGTATT TTAAGTGAGT AACAAATGAT   
  
  
+ TTTGTCTATA GTTATATGTA TATTATCTAA TTTTGAGTAA TAAGAATCAT TAATATAAAT ATTTTGAATA   
  
  
+ AAATTAGATA GATAAACTAT AAATATAGAG TTAAGTATAC TTATGTTTGG TTATCTTCGA ATATTACCCG   
  
  
+ TTCGGATATA TTATCTGAAC TGGTGAAATA AGTAATATGT TTTGTTGTTT TAATTAGATA ATTTTTAGAC   
  
  
+ CGAGCTTGTG AATATATACT AGACAAACAT TTATATTTCG AGTCTGCACT TATATTCTAT AAGAGCTTGA   
  
  
+ TATATTAGAT TTGAACACTA ACCTGTTAAT ATAGTTTGCC GGTGATTTTT TTTCAAAATT TTGATTCTTA   
  
  
+ GATATGTATA TGGAGTAAAA CTAATTTTTA CAGATGCCCA TTTTTTTAAT TGACACTTAT GTAATTAACT   
  
  
+ GAATTCATAA AACAAGGTTT TTTAAAAAAA TTTAACTCAT ATCAATGAAA CAAAGACGAG AACGAAAGCA   
  
  
+ CAATTCTATG GAAATGGAAA ATGAAGTCAC TTATGGAGAT TCAATAGTAA GCAAATCGAG AGCAGAAAAT   
  
  
+ CTAATCTCCT TTCGTCATTA TACAATCAAT ATTGCCTATT TGGTTTTAGT GATTTGTTTC AGCCGCAAAA   
  
  
+ CTTAATTTTC TTTGGTGCAT ATGAAATCTT AAAAAGAATT AAAATAAGAT ATAATACGTT AACTCTTCAA   
  
  
+ CAACATGATA TATTTAAGAT ACCAATATTT GTATTCATCA TATAAAAATT GTAGTGTTGC AAAATATTAA   
  
  
+ AATTATTTCA TAAATAAACA TTATTATAAG AACTGACTCC GCGGATTATC ATATGGTATA GATTACAGAG   
  
  
+ TGGGTGGGTT TAAATAATTT CCCCGACACA TTATACTTTT AGAAGAGCTA TTAGCTCCAA ATTATTTAAA   
  
  
+ CAATGTTCTA AAGAGCAAAA AAATCAATGT TTTGGATTTT GATCCGACCG AGAGGACTTG TCCGACCATT   
  
  
+ CCATATTAAA ATGTTCCTAG TTCTGATTAG CTAATACCA  

- ATTTCAACAG CACTAAAGAA CGGGAAATAA AAAATAAATA TAATTGTATA TAAAAATTTT TTTTTAATTT   
  
  
- CTGCAAGGAT TGCCTTTGAG TACATAAAAA ACTAATACTT ATACTTATAG GGGAGATATA ATTAGTACCT   
  
  
- CGTAATGTTG TACAAAAGCA TCGGTATACA GTAGTGCTCT TACTAAAAAT CTTAACAATC TTTTTATTTA   
  
  
- ACTAAGTATA TTTGTATATG ATACAAAAAA TAATTTGATT GATAGTTTAA TTAATTATCA CATGTTTTTT   
  
  
- TATAAAAAAA GAAAGGAATT TATTTTTGAT GCCTTAATGG ATTATACCGA TTGTATATAT ACTGTTAATT   
  
  
- ACTAATACTT ATTATGTATA AACTATTTTT TTAAAGATTG GAGAGAGAAA AAACAAATTA AAATATAATA   
  
  
- ATTTCCTTTA AATTGTTAGT GTAATTAGTA TATTATTTTT GTTAATCTAA AAAAGAATAT ACAATATAAA   
  
  
- ACTTAAAAAT TTTTGCTGAT ATTTAATGAT TTTTACTATT CTCAGGGTGT AATTTTTTAA ACACTAGTTG   
  
  
- GCAAATTGAA AAAAAAATCA AGTTCGTTCT ATGTTTACTA GTATATAGAC TATATCTGCA CCCGCAAGCC   
  
  
- TATGTGCAAG CCCAAACATA GTCTATAAAG TCATATTTCC ATATCTTGGG CAAGCCCATA AAGATGTGAG   
  
  
- GCTCAGCCCA AGCCCAAGCC TATAAAACCT AGCCCAAGCT TATAAATTTA AAACTTCTTT TTTTCTTTAA   
  
  
- TAAGTGACAA ATTCAAAAAA TATAAATTTA TATAGAATTG AATTGACTAA AAAAAATCAA AAATTTTCTA   
  
  
- ATTTTATAAT TATACAAACC TCTATTTTGA AATTTTTATC TTTCTGTGAT TAAATCAAAA ACAAAACTTT   
  
  
- TAAATCTACG TTGAAAACAA TTACGTTCTT TGTTCTTGAA CTATACATAA AATTCACTCA TTGTTTACTA   
  
  
- AAACAGATAT CAATATACAT ATAATAGATT AAAACTCATT ATTCTTAGTA ATTATATTTA TAAAACTTAT   
  
  
- TTTAATCTAT CTATTTGATA TTTATATCTC AATTCATATG AATACAAACC AATAGAAGCT TATAATGGGC   
  
  
- AAGCCTATAT AATAGACTTG ACCACTTTAT TCATTATACA AAACAACAAA ATTAATCTAT TAAAAATCTG   
  
  
- GCTCGAACAC TTATATATGA TCTGTTTGTA AATATAAAGC TCAGACGTGA ATATAAGATA TTCTCGAACT   
  
  
- ATATAATCTA AACTTGTGAT TGGACAATTA TATCAAACGG CCACTAAAAA AAAGTTTTAA AACTAAGAAT   
  
  
- CTATACATAT ACCTCATTTT GATTAAAAAT GTCTACGGGT AAAAAAATTA ACTGTGAATA CATTAATTGA   
  
  
- CTTAAGTATT TTGTTCCAAA AAATTTTTTT AAATTGAGTA TAGTTACTTT GTTTCTGCTC TTGCTTTCGT   
  
  
- GTTAAGATAC CTTTACCTTT TACTTCAGTG AATACCTCTA AGTTATCATT CGTTTAGCTC TCGTCTTTTA   
  
  
- GATTAGAGGA AAGCAGTAAT ATGTTAGTTA TAACGGATAA ACCAAAATCA CTAAACAAAG TCGGCGTTTT   
  
  
- GAATTAAAAG AAACCACGTA TACTTTAGAA TTTTTCTTAA TTTTATTCTA TATTATGCAA TTGAGAAGTT   
  
  
- GTTGTACTAT ATAAATTCTA TGGTTATAAA CATAAGTAGT ATATTTTTAA CATCACAACG TTTTATAATT   
  
  
- TTAATAAAGT ATTTATTTGT AATAATATTC TTGACTGAGG CGCCTAATAG TATACCATAT CTAATGTCTC   
  
  
- ACCCACCCAA ATTTATTAAA GGGGCTGTGT AATATGAAAA TCTTCTCGAT AATCGAGGTT TAATAAATTT   
  
  
- GTTACAAGAT TTCTCGTTTT TTTAGTTACA AAACCTAAAA CTAGGCTGGC TCTCCTGAAC AGGCTGGTAA   
  
  
- GGTATAATTT TACAAGGATC AAGACTAATC GATTATGGT

+     AE-box

| Site Name | Organism | Position | Strand | Matrix score. | sequence | function |
| --- | --- | --- | --- | --- | --- | --- |
| AE-box | Arabidopsis thaliana | 937 | + | 8 | AGAAACAA | part of a module for light response |

>PlantCARE\_4926   
+ TAAAGTTGTC GTGATTTCTT GCCCTTTATT TTTTATTTAT ATTAACATAT ATTTTTAAAA AAAAATTAAA   
  
  
+ GACGTTCCTA ACGGAAACTC ATGTATTTTT TGATTATGAA TATGAATATC CCCTCTATAT TAATCATGGA   
  
  
+ GCATTACAAC ATGTTTTCGT AGCCATATGT CATCACGAGA ATGATTTTTA GAATTGTTAG AAAAATAAAT   
  
  
+ TGATTCATAT AAACATATAC TATGTTTTTT ATTAAACTAA CTATCAAATT AATTAATAGT GTACAAAAAA   
  
  
+ ATATTTTTTT CTTTCCTTAA ATAAAAACTA CGGAATTACC TAATATGGCT AACATATATA TGACAATTAA   
  
  
+ TGATTATGAA TAATACATAT TTGATAAAAA AATTTCTAAC CTCTCTCTTT TTTGTTTAAT TTTATATTAT   
  
  
+ TAAAGGAAAT TTAACAATCA CATTAATCAT ATAATAAAAA CAATTAGATT TTTTCTTATA TGTTATATTT   
  
  
+ TGAATTTTTA AAAACGACTA TAAATTACTA AAAATGATAA GAGTCCCACA TTAAAAAATT TGTGATCAAC   
  
  
+ CGTTTAACTT TTTTTTTAGT TCAAGCAAGA TACAAATGAT CATATATCTG ATATAGACGT GGGCGTTCGG   
  
  
+ ATACACGTTC GGGTTTGTAT CAGATATTTC AGTATAAAGG TATAGAACCC GTTCGGGTAT TTCTACACTC   
  
  
+ CGAGTCGGGT TCGGGTTCGG ATATTTTGGA TCGGGTTCGA ATATTTAAAT TTTGAAGAAA AAAAGAAATT   
  
  
+ ATTCACTGTT TAAGTTTTTT ATATTTAAAT ATATCTTAAC TTAACTGATT TTTTTTAGTT TTTAAAAGAT   
  
  
+ TAAAATATTA ATATGTTTGG AGATAAAACT TTAAAAATAG AAAGACACTA ATTTAGTTTT TGTTTTGAAA   
  
  
+ ATTTAGATGC AACTTTTGTT AATGCAAGAA ACAAGAACTT GATATGTATT TTAAGTGAGT AACAAATGAT   
  
  
+ TTTGTCTATA GTTATATGTA TATTATCTAA TTTTGAGTAA TAAGAATCAT TAATATAAAT ATTTTGAATA   
  
  
+ AAATTAGATA GATAAACTAT AAATATAGAG TTAAGTATAC TTATGTTTGG TTATCTTCGA ATATTACCCG   
  
  
+ TTCGGATATA TTATCTGAAC TGGTGAAATA AGTAATATGT TTTGTTGTTT TAATTAGATA ATTTTTAGAC   
  
  
+ CGAGCTTGTG AATATATACT AGACAAACAT TTATATTTCG AGTCTGCACT TATATTCTAT AAGAGCTTGA   
  
  
+ TATATTAGAT TTGAACACTA ACCTGTTAAT ATAGTTTGCC GGTGATTTTT TTTCAAAATT TTGATTCTTA   
  
  
+ GATATGTATA TGGAGTAAAA CTAATTTTTA CAGATGCCCA TTTTTTTAAT TGACACTTAT GTAATTAACT   
  
  
+ GAATTCATAA AACAAGGTTT TTTAAAAAAA TTTAACTCAT ATCAATGAAA CAAAGACGAG AACGAAAGCA   
  
  
+ CAATTCTATG GAAATGGAAA ATGAAGTCAC TTATGGAGAT TCAATAGTAA GCAAATCGAG AGCAGAAAAT   
  
  
+ CTAATCTCCT TTCGTCATTA TACAATCAAT ATTGCCTATT TGGTTTTAGT GATTTGTTTC AGCCGCAAAA   
  
  
+ CTTAATTTTC TTTGGTGCAT ATGAAATCTT AAAAAGAATT AAAATAAGAT ATAATACGTT AACTCTTCAA   
  
  
+ CAACATGATA TATTTAAGAT ACCAATATTT GTATTCATCA TATAAAAATT GTAGTGTTGC AAAATATTAA   
  
  
+ AATTATTTCA TAAATAAACA TTATTATAAG AACTGACTCC GCGGATTATC ATATGGTATA GATTACAGAG   
  
  
+ TGGGTGGGTT TAAATAATTT CCCCGACACA TTATACTTTT AGAAGAGCTA TTAGCTCCAA ATTATTTAAA   
  
  
+ CAATGTTCTA AAGAGCAAAA AAATCAATGT TTTGGATTTT GATCCGACCG AGAGGACTTG TCCGACCATT   
  
  
+ CCATATTAAA ATGTTCCTAG TTCTGATTAG CTAATACCA  

- ATTTCAACAG CACTAAAGAA CGGGAAATAA AAAATAAATA TAATTGTATA TAAAAATTTT TTTTTAATTT   
  
  
- CTGCAAGGAT TGCCTTTGAG TACATAAAAA ACTAATACTT ATACTTATAG GGGAGATATA ATTAGTACCT   
  
  
- CGTAATGTTG TACAAAAGCA TCGGTATACA GTAGTGCTCT TACTAAAAAT CTTAACAATC TTTTTATTTA   
  
  
- ACTAAGTATA TTTGTATATG ATACAAAAAA TAATTTGATT GATAGTTTAA TTAATTATCA CATGTTTTTT   
  
  
- TATAAAAAAA GAAAGGAATT TATTTTTGAT GCCTTAATGG ATTATACCGA TTGTATATAT ACTGTTAATT   
  
  
- ACTAATACTT ATTATGTATA AACTATTTTT TTAAAGATTG GAGAGAGAAA AAACAAATTA AAATATAATA   
  
  
- ATTTCCTTTA AATTGTTAGT GTAATTAGTA TATTATTTTT GTTAATCTAA AAAAGAATAT ACAATATAAA   
  
  
- ACTTAAAAAT TTTTGCTGAT ATTTAATGAT TTTTACTATT CTCAGGGTGT AATTTTTTAA ACACTAGTTG   
  
  
- GCAAATTGAA AAAAAAATCA AGTTCGTTCT ATGTTTACTA GTATATAGAC TATATCTGCA CCCGCAAGCC   
  
  
- TATGTGCAAG CCCAAACATA GTCTATAAAG TCATATTTCC ATATCTTGGG CAAGCCCATA AAGATGTGAG   
  
  
- GCTCAGCCCA AGCCCAAGCC TATAAAACCT AGCCCAAGCT TATAAATTTA AAACTTCTTT TTTTCTTTAA   
  
  
- TAAGTGACAA ATTCAAAAAA TATAAATTTA TATAGAATTG AATTGACTAA AAAAAATCAA AAATTTTCTA   
  
  
- ATTTTATAAT TATACAAACC TCTATTTTGA AATTTTTATC TTTCTGTGAT TAAATCAAAA ACAAAACTTT   
  
  
- TAAATCTACG TTGAAAACAA TTACGTTCTT TGTTCTTGAA CTATACATAA AATTCACTCA TTGTTTACTA   
  
  
- AAACAGATAT CAATATACAT ATAATAGATT AAAACTCATT ATTCTTAGTA ATTATATTTA TAAAACTTAT   
  
  
- TTTAATCTAT CTATTTGATA TTTATATCTC AATTCATATG AATACAAACC AATAGAAGCT TATAATGGGC   
  
  
- AAGCCTATAT AATAGACTTG ACCACTTTAT TCATTATACA AAACAACAAA ATTAATCTAT TAAAAATCTG   
  
  
- GCTCGAACAC TTATATATGA TCTGTTTGTA AATATAAAGC TCAGACGTGA ATATAAGATA TTCTCGAACT   
  
  
- ATATAATCTA AACTTGTGAT TGGACAATTA TATCAAACGG CCACTAAAAA AAAGTTTTAA AACTAAGAAT   
  
  
- CTATACATAT ACCTCATTTT GATTAAAAAT GTCTACGGGT AAAAAAATTA ACTGTGAATA CATTAATTGA   
  
  
- CTTAAGTATT TTGTTCCAAA AAATTTTTTT AAATTGAGTA TAGTTACTTT GTTTCTGCTC TTGCTTTCGT   
  
  
- GTTAAGATAC CTTTACCTTT TACTTCAGTG AATACCTCTA AGTTATCATT CGTTTAGCTC TCGTCTTTTA   
  
  
- GATTAGAGGA AAGCAGTAAT ATGTTAGTTA TAACGGATAA ACCAAAATCA CTAAACAAAG TCGGCGTTTT   
  
  
- GAATTAAAAG AAACCACGTA TACTTTAGAA TTTTTCTTAA TTTTATTCTA TATTATGCAA TTGAGAAGTT   
  
  
- GTTGTACTAT ATAAATTCTA TGGTTATAAA CATAAGTAGT ATATTTTTAA CATCACAACG TTTTATAATT   
  
  
- TTAATAAAGT ATTTATTTGT AATAATATTC TTGACTGAGG CGCCTAATAG TATACCATAT CTAATGTCTC   
  
  
- ACCCACCCAA ATTTATTAAA GGGGCTGTGT AATATGAAAA TCTTCTCGAT AATCGAGGTT TAATAAATTT   
  
  
- GTTACAAGAT TTCTCGTTTT TTTAGTTACA AAACCTAAAA CTAGGCTGGC TCTCCTGAAC AGGCTGGTAA   
  
  
- GGTATAATTT TACAAGGATC AAGACTAATC GATTATGGT

+     ARE

| Site Name | Organism | Position | Strand | Matrix score. | sequence | function |
| --- | --- | --- | --- | --- | --- | --- |
| ARE | Zea mays | 1581 | - | 6 | AAACCA | cis-acting regulatory element essential for the anaerobic induction |

>PlantCARE\_4926   
+ TAAAGTTGTC GTGATTTCTT GCCCTTTATT TTTTATTTAT ATTAACATAT ATTTTTAAAA AAAAATTAAA   
  
  
+ GACGTTCCTA ACGGAAACTC ATGTATTTTT TGATTATGAA TATGAATATC CCCTCTATAT TAATCATGGA   
  
  
+ GCATTACAAC ATGTTTTCGT AGCCATATGT CATCACGAGA ATGATTTTTA GAATTGTTAG AAAAATAAAT   
  
  
+ TGATTCATAT AAACATATAC TATGTTTTTT ATTAAACTAA CTATCAAATT AATTAATAGT GTACAAAAAA   
  
  
+ ATATTTTTTT CTTTCCTTAA ATAAAAACTA CGGAATTACC TAATATGGCT AACATATATA TGACAATTAA   
  
  
+ TGATTATGAA TAATACATAT TTGATAAAAA AATTTCTAAC CTCTCTCTTT TTTGTTTAAT TTTATATTAT   
  
  
+ TAAAGGAAAT TTAACAATCA CATTAATCAT ATAATAAAAA CAATTAGATT TTTTCTTATA TGTTATATTT   
  
  
+ TGAATTTTTA AAAACGACTA TAAATTACTA AAAATGATAA GAGTCCCACA TTAAAAAATT TGTGATCAAC   
  
  
+ CGTTTAACTT TTTTTTTAGT TCAAGCAAGA TACAAATGAT CATATATCTG ATATAGACGT GGGCGTTCGG   
  
  
+ ATACACGTTC GGGTTTGTAT CAGATATTTC AGTATAAAGG TATAGAACCC GTTCGGGTAT TTCTACACTC   
  
  
+ CGAGTCGGGT TCGGGTTCGG ATATTTTGGA TCGGGTTCGA ATATTTAAAT TTTGAAGAAA AAAAGAAATT   
  
  
+ ATTCACTGTT TAAGTTTTTT ATATTTAAAT ATATCTTAAC TTAACTGATT TTTTTTAGTT TTTAAAAGAT   
  
  
+ TAAAATATTA ATATGTTTGG AGATAAAACT TTAAAAATAG AAAGACACTA ATTTAGTTTT TGTTTTGAAA   
  
  
+ ATTTAGATGC AACTTTTGTT AATGCAAGAA ACAAGAACTT GATATGTATT TTAAGTGAGT AACAAATGAT   
  
  
+ TTTGTCTATA GTTATATGTA TATTATCTAA TTTTGAGTAA TAAGAATCAT TAATATAAAT ATTTTGAATA   
  
  
+ AAATTAGATA GATAAACTAT AAATATAGAG TTAAGTATAC TTATGTTTGG TTATCTTCGA ATATTACCCG   
  
  
+ TTCGGATATA TTATCTGAAC TGGTGAAATA AGTAATATGT TTTGTTGTTT TAATTAGATA ATTTTTAGAC   
  
  
+ CGAGCTTGTG AATATATACT AGACAAACAT TTATATTTCG AGTCTGCACT TATATTCTAT AAGAGCTTGA   
  
  
+ TATATTAGAT TTGAACACTA ACCTGTTAAT ATAGTTTGCC GGTGATTTTT TTTCAAAATT TTGATTCTTA   
  
  
+ GATATGTATA TGGAGTAAAA CTAATTTTTA CAGATGCCCA TTTTTTTAAT TGACACTTAT GTAATTAACT   
  
  
+ GAATTCATAA AACAAGGTTT TTTAAAAAAA TTTAACTCAT ATCAATGAAA CAAAGACGAG AACGAAAGCA   
  
  
+ CAATTCTATG GAAATGGAAA ATGAAGTCAC TTATGGAGAT TCAATAGTAA GCAAATCGAG AGCAGAAAAT   
  
  
+ CTAATCTCCT TTCGTCATTA TACAATCAAT ATTGCCTATT TGGTTTTAGT GATTTGTTTC AGCCGCAAAA   
  
  
+ CTTAATTTTC TTTGGTGCAT ATGAAATCTT AAAAAGAATT AAAATAAGAT ATAATACGTT AACTCTTCAA   
  
  
+ CAACATGATA TATTTAAGAT ACCAATATTT GTATTCATCA TATAAAAATT GTAGTGTTGC AAAATATTAA   
  
  
+ AATTATTTCA TAAATAAACA TTATTATAAG AACTGACTCC GCGGATTATC ATATGGTATA GATTACAGAG   
  
  
+ TGGGTGGGTT TAAATAATTT CCCCGACACA TTATACTTTT AGAAGAGCTA TTAGCTCCAA ATTATTTAAA   
  
  
+ CAATGTTCTA AAGAGCAAAA AAATCAATGT TTTGGATTTT GATCCGACCG AGAGGACTTG TCCGACCATT   
  
  
+ CCATATTAAA ATGTTCCTAG TTCTGATTAG CTAATACCA  

- ATTTCAACAG CACTAAAGAA CGGGAAATAA AAAATAAATA TAATTGTATA TAAAAATTTT TTTTTAATTT   
  
  
- CTGCAAGGAT TGCCTTTGAG TACATAAAAA ACTAATACTT ATACTTATAG GGGAGATATA ATTAGTACCT   
  
  
- CGTAATGTTG TACAAAAGCA TCGGTATACA GTAGTGCTCT TACTAAAAAT CTTAACAATC TTTTTATTTA   
  
  
- ACTAAGTATA TTTGTATATG ATACAAAAAA TAATTTGATT GATAGTTTAA TTAATTATCA CATGTTTTTT   
  
  
- TATAAAAAAA GAAAGGAATT TATTTTTGAT GCCTTAATGG ATTATACCGA TTGTATATAT ACTGTTAATT   
  
  
- ACTAATACTT ATTATGTATA AACTATTTTT TTAAAGATTG GAGAGAGAAA AAACAAATTA AAATATAATA   
  
  
- ATTTCCTTTA AATTGTTAGT GTAATTAGTA TATTATTTTT GTTAATCTAA AAAAGAATAT ACAATATAAA   
  
  
- ACTTAAAAAT TTTTGCTGAT ATTTAATGAT TTTTACTATT CTCAGGGTGT AATTTTTTAA ACACTAGTTG   
  
  
- GCAAATTGAA AAAAAAATCA AGTTCGTTCT ATGTTTACTA GTATATAGAC TATATCTGCA CCCGCAAGCC   
  
  
- TATGTGCAAG CCCAAACATA GTCTATAAAG TCATATTTCC ATATCTTGGG CAAGCCCATA AAGATGTGAG   
  
  
- GCTCAGCCCA AGCCCAAGCC TATAAAACCT AGCCCAAGCT TATAAATTTA AAACTTCTTT TTTTCTTTAA   
  
  
- TAAGTGACAA ATTCAAAAAA TATAAATTTA TATAGAATTG AATTGACTAA AAAAAATCAA AAATTTTCTA   
  
  
- ATTTTATAAT TATACAAACC TCTATTTTGA AATTTTTATC TTTCTGTGAT TAAATCAAAA ACAAAACTTT   
  
  
- TAAATCTACG TTGAAAACAA TTACGTTCTT TGTTCTTGAA CTATACATAA AATTCACTCA TTGTTTACTA   
  
  
- AAACAGATAT CAATATACAT ATAATAGATT AAAACTCATT ATTCTTAGTA ATTATATTTA TAAAACTTAT   
  
  
- TTTAATCTAT CTATTTGATA TTTATATCTC AATTCATATG AATACAAACC AATAGAAGCT TATAATGGGC   
  
  
- AAGCCTATAT AATAGACTTG ACCACTTTAT TCATTATACA AAACAACAAA ATTAATCTAT TAAAAATCTG   
  
  
- GCTCGAACAC TTATATATGA TCTGTTTGTA AATATAAAGC TCAGACGTGA ATATAAGATA TTCTCGAACT   
  
  
- ATATAATCTA AACTTGTGAT TGGACAATTA TATCAAACGG CCACTAAAAA AAAGTTTTAA AACTAAGAAT   
  
  
- CTATACATAT ACCTCATTTT GATTAAAAAT GTCTACGGGT AAAAAAATTA ACTGTGAATA CATTAATTGA   
  
  
- CTTAAGTATT TTGTTCCAAA AAATTTTTTT AAATTGAGTA TAGTTACTTT GTTTCTGCTC TTGCTTTCGT   
  
  
- GTTAAGATAC CTTTACCTTT TACTTCAGTG AATACCTCTA AGTTATCATT CGTTTAGCTC TCGTCTTTTA   
  
  
- GATTAGAGGA AAGCAGTAAT ATGTTAGTTA TAACGGATAA ACCAAAATCA CTAAACAAAG TCGGCGTTTT   
  
  
- GAATTAAAAG AAACCACGTA TACTTTAGAA TTTTTCTTAA TTTTATTCTA TATTATGCAA TTGAGAAGTT   
  
  
- GTTGTACTAT ATAAATTCTA TGGTTATAAA CATAAGTAGT ATATTTTTAA CATCACAACG TTTTATAATT   
  
  
- TTAATAAAGT ATTTATTTGT AATAATATTC TTGACTGAGG CGCCTAATAG TATACCATAT CTAATGTCTC   
  
  
- ACCCACCCAA ATTTATTAAA GGGGCTGTGT AATATGAAAA TCTTCTCGAT AATCGAGGTT TAATAAATTT   
  
  
- GTTACAAGAT TTCTCGTTTT TTTAGTTACA AAACCTAAAA CTAGGCTGGC TCTCCTGAAC AGGCTGGTAA   
  
  
- GGTATAATTT TACAAGGATC AAGACTAATC GATTATGGT

+     ATCT-motif

| Site Name | Organism | Position | Strand | Matrix score. | sequence | function |
| --- | --- | --- | --- | --- | --- | --- |
| ATCT-motif | Pisum sativum | 1538 | + | 9 | AATCTAATCC | part of a conserved DNA module involved in light responsiveness |

>PlantCARE\_4926   
+ TAAAGTTGTC GTGATTTCTT GCCCTTTATT TTTTATTTAT ATTAACATAT ATTTTTAAAA AAAAATTAAA   
  
  
+ GACGTTCCTA ACGGAAACTC ATGTATTTTT TGATTATGAA TATGAATATC CCCTCTATAT TAATCATGGA   
  
  
+ GCATTACAAC ATGTTTTCGT AGCCATATGT CATCACGAGA ATGATTTTTA GAATTGTTAG AAAAATAAAT   
  
  
+ TGATTCATAT AAACATATAC TATGTTTTTT ATTAAACTAA CTATCAAATT AATTAATAGT GTACAAAAAA   
  
  
+ ATATTTTTTT CTTTCCTTAA ATAAAAACTA CGGAATTACC TAATATGGCT AACATATATA TGACAATTAA   
  
  
+ TGATTATGAA TAATACATAT TTGATAAAAA AATTTCTAAC CTCTCTCTTT TTTGTTTAAT TTTATATTAT   
  
  
+ TAAAGGAAAT TTAACAATCA CATTAATCAT ATAATAAAAA CAATTAGATT TTTTCTTATA TGTTATATTT   
  
  
+ TGAATTTTTA AAAACGACTA TAAATTACTA AAAATGATAA GAGTCCCACA TTAAAAAATT TGTGATCAAC   
  
  
+ CGTTTAACTT TTTTTTTAGT TCAAGCAAGA TACAAATGAT CATATATCTG ATATAGACGT GGGCGTTCGG   
  
  
+ ATACACGTTC GGGTTTGTAT CAGATATTTC AGTATAAAGG TATAGAACCC GTTCGGGTAT TTCTACACTC   
  
  
+ CGAGTCGGGT TCGGGTTCGG ATATTTTGGA TCGGGTTCGA ATATTTAAAT TTTGAAGAAA AAAAGAAATT   
  
  
+ ATTCACTGTT TAAGTTTTTT ATATTTAAAT ATATCTTAAC TTAACTGATT TTTTTTAGTT TTTAAAAGAT   
  
  
+ TAAAATATTA ATATGTTTGG AGATAAAACT TTAAAAATAG AAAGACACTA ATTTAGTTTT TGTTTTGAAA   
  
  
+ ATTTAGATGC AACTTTTGTT AATGCAAGAA ACAAGAACTT GATATGTATT TTAAGTGAGT AACAAATGAT   
  
  
+ TTTGTCTATA GTTATATGTA TATTATCTAA TTTTGAGTAA TAAGAATCAT TAATATAAAT ATTTTGAATA   
  
  
+ AAATTAGATA GATAAACTAT AAATATAGAG TTAAGTATAC TTATGTTTGG TTATCTTCGA ATATTACCCG   
  
  
+ TTCGGATATA TTATCTGAAC TGGTGAAATA AGTAATATGT TTTGTTGTTT TAATTAGATA ATTTTTAGAC   
  
  
+ CGAGCTTGTG AATATATACT AGACAAACAT TTATATTTCG AGTCTGCACT TATATTCTAT AAGAGCTTGA   
  
  
+ TATATTAGAT TTGAACACTA ACCTGTTAAT ATAGTTTGCC GGTGATTTTT TTTCAAAATT TTGATTCTTA   
  
  
+ GATATGTATA TGGAGTAAAA CTAATTTTTA CAGATGCCCA TTTTTTTAAT TGACACTTAT GTAATTAACT   
  
  
+ GAATTCATAA AACAAGGTTT TTTAAAAAAA TTTAACTCAT ATCAATGAAA CAAAGACGAG AACGAAAGCA   
  
  
+ CAATTCTATG GAAATGGAAA ATGAAGTCAC TTATGGAGAT TCAATAGTAA GCAAATCGAG AGCAGAAAAT   
  
  
+ CTAATCTCCT TTCGTCATTA TACAATCAAT ATTGCCTATT TGGTTTTAGT GATTTGTTTC AGCCGCAAAA   
  
  
+ CTTAATTTTC TTTGGTGCAT ATGAAATCTT AAAAAGAATT AAAATAAGAT ATAATACGTT AACTCTTCAA   
  
  
+ CAACATGATA TATTTAAGAT ACCAATATTT GTATTCATCA TATAAAAATT GTAGTGTTGC AAAATATTAA   
  
  
+ AATTATTTCA TAAATAAACA TTATTATAAG AACTGACTCC GCGGATTATC ATATGGTATA GATTACAGAG   
  
  
+ TGGGTGGGTT TAAATAATTT CCCCGACACA TTATACTTTT AGAAGAGCTA TTAGCTCCAA ATTATTTAAA   
  
  
+ CAATGTTCTA AAGAGCAAAA AAATCAATGT TTTGGATTTT GATCCGACCG AGAGGACTTG TCCGACCATT   
  
  
+ CCATATTAAA ATGTTCCTAG TTCTGATTAG CTAATACCA  

- ATTTCAACAG CACTAAAGAA CGGGAAATAA AAAATAAATA TAATTGTATA TAAAAATTTT TTTTTAATTT   
  
  
- CTGCAAGGAT TGCCTTTGAG TACATAAAAA ACTAATACTT ATACTTATAG GGGAGATATA ATTAGTACCT   
  
  
- CGTAATGTTG TACAAAAGCA TCGGTATACA GTAGTGCTCT TACTAAAAAT CTTAACAATC TTTTTATTTA   
  
  
- ACTAAGTATA TTTGTATATG ATACAAAAAA TAATTTGATT GATAGTTTAA TTAATTATCA CATGTTTTTT   
  
  
- TATAAAAAAA GAAAGGAATT TATTTTTGAT GCCTTAATGG ATTATACCGA TTGTATATAT ACTGTTAATT   
  
  
- ACTAATACTT ATTATGTATA AACTATTTTT TTAAAGATTG GAGAGAGAAA AAACAAATTA AAATATAATA   
  
  
- ATTTCCTTTA AATTGTTAGT GTAATTAGTA TATTATTTTT GTTAATCTAA AAAAGAATAT ACAATATAAA   
  
  
- ACTTAAAAAT TTTTGCTGAT ATTTAATGAT TTTTACTATT CTCAGGGTGT AATTTTTTAA ACACTAGTTG   
  
  
- GCAAATTGAA AAAAAAATCA AGTTCGTTCT ATGTTTACTA GTATATAGAC TATATCTGCA CCCGCAAGCC   
  
  
- TATGTGCAAG CCCAAACATA GTCTATAAAG TCATATTTCC ATATCTTGGG CAAGCCCATA AAGATGTGAG   
  
  
- GCTCAGCCCA AGCCCAAGCC TATAAAACCT AGCCCAAGCT TATAAATTTA AAACTTCTTT TTTTCTTTAA   
  
  
- TAAGTGACAA ATTCAAAAAA TATAAATTTA TATAGAATTG AATTGACTAA AAAAAATCAA AAATTTTCTA   
  
  
- ATTTTATAAT TATACAAACC TCTATTTTGA AATTTTTATC TTTCTGTGAT TAAATCAAAA ACAAAACTTT   
  
  
- TAAATCTACG TTGAAAACAA TTACGTTCTT TGTTCTTGAA CTATACATAA AATTCACTCA TTGTTTACTA   
  
  
- AAACAGATAT CAATATACAT ATAATAGATT AAAACTCATT ATTCTTAGTA ATTATATTTA TAAAACTTAT   
  
  
- TTTAATCTAT CTATTTGATA TTTATATCTC AATTCATATG AATACAAACC AATAGAAGCT TATAATGGGC   
  
  
- AAGCCTATAT AATAGACTTG ACCACTTTAT TCATTATACA AAACAACAAA ATTAATCTAT TAAAAATCTG   
  
  
- GCTCGAACAC TTATATATGA TCTGTTTGTA AATATAAAGC TCAGACGTGA ATATAAGATA TTCTCGAACT   
  
  
- ATATAATCTA AACTTGTGAT TGGACAATTA TATCAAACGG CCACTAAAAA AAAGTTTTAA AACTAAGAAT   
  
  
- CTATACATAT ACCTCATTTT GATTAAAAAT GTCTACGGGT AAAAAAATTA ACTGTGAATA CATTAATTGA   
  
  
- CTTAAGTATT TTGTTCCAAA AAATTTTTTT AAATTGAGTA TAGTTACTTT GTTTCTGCTC TTGCTTTCGT   
  
  
- GTTAAGATAC CTTTACCTTT TACTTCAGTG AATACCTCTA AGTTATCATT CGTTTAGCTC TCGTCTTTTA   
  
  
- GATTAGAGGA AAGCAGTAAT ATGTTAGTTA TAACGGATAA ACCAAAATCA CTAAACAAAG TCGGCGTTTT   
  
  
- GAATTAAAAG AAACCACGTA TACTTTAGAA TTTTTCTTAA TTTTATTCTA TATTATGCAA TTGAGAAGTT   
  
  
- GTTGTACTAT ATAAATTCTA TGGTTATAAA CATAAGTAGT ATATTTTTAA CATCACAACG TTTTATAATT   
  
  
- TTAATAAAGT ATTTATTTGT AATAATATTC TTGACTGAGG CGCCTAATAG TATACCATAT CTAATGTCTC   
  
  
- ACCCACCCAA ATTTATTAAA GGGGCTGTGT AATATGAAAA TCTTCTCGAT AATCGAGGTT TAATAAATTT   
  
  
- GTTACAAGAT TTCTCGTTTT TTTAGTTACA AAACCTAAAA CTAGGCTGGC TCTCCTGAAC AGGCTGGTAA   
  
  
- GGTATAATTT TACAAGGATC AAGACTAATC GATTATGGT

+     AT~TATA-box

| Site Name | Organism | Position | Strand | Matrix score. | sequence | function |
| --- | --- | --- | --- | --- | --- | --- |
| AT~TATA-box | Arabidopsis thaliana | 335 | + | 6 | TATATA |  |
| AT~TATA-box | Arabidopsis thaliana | 1203 | - | 6 | TATATA |  |

>PlantCARE\_4926   
+ TAAAGTTGTC GTGATTTCTT GCCCTTTATT TTTTATTTAT ATTAACATAT ATTTTTAAAA AAAAATTAAA   
  
  
+ GACGTTCCTA ACGGAAACTC ATGTATTTTT TGATTATGAA TATGAATATC CCCTCTATAT TAATCATGGA   
  
  
+ GCATTACAAC ATGTTTTCGT AGCCATATGT CATCACGAGA ATGATTTTTA GAATTGTTAG AAAAATAAAT   
  
  
+ TGATTCATAT AAACATATAC TATGTTTTTT ATTAAACTAA CTATCAAATT AATTAATAGT GTACAAAAAA   
  
  
+ ATATTTTTTT CTTTCCTTAA ATAAAAACTA CGGAATTACC TAATATGGCT AACATATATA TGACAATTAA   
  
  
+ TGATTATGAA TAATACATAT TTGATAAAAA AATTTCTAAC CTCTCTCTTT TTTGTTTAAT TTTATATTAT   
  
  
+ TAAAGGAAAT TTAACAATCA CATTAATCAT ATAATAAAAA CAATTAGATT TTTTCTTATA TGTTATATTT   
  
  
+ TGAATTTTTA AAAACGACTA TAAATTACTA AAAATGATAA GAGTCCCACA TTAAAAAATT TGTGATCAAC   
  
  
+ CGTTTAACTT TTTTTTTAGT TCAAGCAAGA TACAAATGAT CATATATCTG ATATAGACGT GGGCGTTCGG   
  
  
+ ATACACGTTC GGGTTTGTAT CAGATATTTC AGTATAAAGG TATAGAACCC GTTCGGGTAT TTCTACACTC   
  
  
+ CGAGTCGGGT TCGGGTTCGG ATATTTTGGA TCGGGTTCGA ATATTTAAAT TTTGAAGAAA AAAAGAAATT   
  
  
+ ATTCACTGTT TAAGTTTTTT ATATTTAAAT ATATCTTAAC TTAACTGATT TTTTTTAGTT TTTAAAAGAT   
  
  
+ TAAAATATTA ATATGTTTGG AGATAAAACT TTAAAAATAG AAAGACACTA ATTTAGTTTT TGTTTTGAAA   
  
  
+ ATTTAGATGC AACTTTTGTT AATGCAAGAA ACAAGAACTT GATATGTATT TTAAGTGAGT AACAAATGAT   
  
  
+ TTTGTCTATA GTTATATGTA TATTATCTAA TTTTGAGTAA TAAGAATCAT TAATATAAAT ATTTTGAATA   
  
  
+ AAATTAGATA GATAAACTAT AAATATAGAG TTAAGTATAC TTATGTTTGG TTATCTTCGA ATATTACCCG   
  
  
+ TTCGGATATA TTATCTGAAC TGGTGAAATA AGTAATATGT TTTGTTGTTT TAATTAGATA ATTTTTAGAC   
  
  
+ CGAGCTTGTG AATATATACT AGACAAACAT TTATATTTCG AGTCTGCACT TATATTCTAT AAGAGCTTGA   
  
  
+ TATATTAGAT TTGAACACTA ACCTGTTAAT ATAGTTTGCC GGTGATTTTT TTTCAAAATT TTGATTCTTA   
  
  
+ GATATGTATA TGGAGTAAAA CTAATTTTTA CAGATGCCCA TTTTTTTAAT TGACACTTAT GTAATTAACT   
  
  
+ GAATTCATAA AACAAGGTTT TTTAAAAAAA TTTAACTCAT ATCAATGAAA CAAAGACGAG AACGAAAGCA   
  
  
+ CAATTCTATG GAAATGGAAA ATGAAGTCAC TTATGGAGAT TCAATAGTAA GCAAATCGAG AGCAGAAAAT   
  
  
+ CTAATCTCCT TTCGTCATTA TACAATCAAT ATTGCCTATT TGGTTTTAGT GATTTGTTTC AGCCGCAAAA   
  
  
+ CTTAATTTTC TTTGGTGCAT ATGAAATCTT AAAAAGAATT AAAATAAGAT ATAATACGTT AACTCTTCAA   
  
  
+ CAACATGATA TATTTAAGAT ACCAATATTT GTATTCATCA TATAAAAATT GTAGTGTTGC AAAATATTAA   
  
  
+ AATTATTTCA TAAATAAACA TTATTATAAG AACTGACTCC GCGGATTATC ATATGGTATA GATTACAGAG   
  
  
+ TGGGTGGGTT TAAATAATTT CCCCGACACA TTATACTTTT AGAAGAGCTA TTAGCTCCAA ATTATTTAAA   
  
  
+ CAATGTTCTA AAGAGCAAAA AAATCAATGT TTTGGATTTT GATCCGACCG AGAGGACTTG TCCGACCATT   
  
  
+ CCATATTAAA ATGTTCCTAG TTCTGATTAG CTAATACCA  

- ATTTCAACAG CACTAAAGAA CGGGAAATAA AAAATAAATA TAATTGTATA TAAAAATTTT TTTTTAATTT   
  
  
- CTGCAAGGAT TGCCTTTGAG TACATAAAAA ACTAATACTT ATACTTATAG GGGAGATATA ATTAGTACCT   
  
  
- CGTAATGTTG TACAAAAGCA TCGGTATACA GTAGTGCTCT TACTAAAAAT CTTAACAATC TTTTTATTTA   
  
  
- ACTAAGTATA TTTGTATATG ATACAAAAAA TAATTTGATT GATAGTTTAA TTAATTATCA CATGTTTTTT   
  
  
- TATAAAAAAA GAAAGGAATT TATTTTTGAT GCCTTAATGG ATTATACCGA TTGTATATAT ACTGTTAATT   
  
  
- ACTAATACTT ATTATGTATA AACTATTTTT TTAAAGATTG GAGAGAGAAA AAACAAATTA AAATATAATA   
  
  
- ATTTCCTTTA AATTGTTAGT GTAATTAGTA TATTATTTTT GTTAATCTAA AAAAGAATAT ACAATATAAA   
  
  
- ACTTAAAAAT TTTTGCTGAT ATTTAATGAT TTTTACTATT CTCAGGGTGT AATTTTTTAA ACACTAGTTG   
  
  
- GCAAATTGAA AAAAAAATCA AGTTCGTTCT ATGTTTACTA GTATATAGAC TATATCTGCA CCCGCAAGCC   
  
  
- TATGTGCAAG CCCAAACATA GTCTATAAAG TCATATTTCC ATATCTTGGG CAAGCCCATA AAGATGTGAG   
  
  
- GCTCAGCCCA AGCCCAAGCC TATAAAACCT AGCCCAAGCT TATAAATTTA AAACTTCTTT TTTTCTTTAA   
  
  
- TAAGTGACAA ATTCAAAAAA TATAAATTTA TATAGAATTG AATTGACTAA AAAAAATCAA AAATTTTCTA   
  
  
- ATTTTATAAT TATACAAACC TCTATTTTGA AATTTTTATC TTTCTGTGAT TAAATCAAAA ACAAAACTTT   
  
  
- TAAATCTACG TTGAAAACAA TTACGTTCTT TGTTCTTGAA CTATACATAA AATTCACTCA TTGTTTACTA   
  
  
- AAACAGATAT CAATATACAT ATAATAGATT AAAACTCATT ATTCTTAGTA ATTATATTTA TAAAACTTAT   
  
  
- TTTAATCTAT CTATTTGATA TTTATATCTC AATTCATATG AATACAAACC AATAGAAGCT TATAATGGGC   
  
  
- AAGCCTATAT AATAGACTTG ACCACTTTAT TCATTATACA AAACAACAAA ATTAATCTAT TAAAAATCTG   
  
  
- GCTCGAACAC TTATATATGA TCTGTTTGTA AATATAAAGC TCAGACGTGA ATATAAGATA TTCTCGAACT   
  
  
- ATATAATCTA AACTTGTGAT TGGACAATTA TATCAAACGG CCACTAAAAA AAAGTTTTAA AACTAAGAAT   
  
  
- CTATACATAT ACCTCATTTT GATTAAAAAT GTCTACGGGT AAAAAAATTA ACTGTGAATA CATTAATTGA   
  
  
- CTTAAGTATT TTGTTCCAAA AAATTTTTTT AAATTGAGTA TAGTTACTTT GTTTCTGCTC TTGCTTTCGT   
  
  
- GTTAAGATAC CTTTACCTTT TACTTCAGTG AATACCTCTA AGTTATCATT CGTTTAGCTC TCGTCTTTTA   
  
  
- GATTAGAGGA AAGCAGTAAT ATGTTAGTTA TAACGGATAA ACCAAAATCA CTAAACAAAG TCGGCGTTTT   
  
  
- GAATTAAAAG AAACCACGTA TACTTTAGAA TTTTTCTTAA TTTTATTCTA TATTATGCAA TTGAGAAGTT   
  
  
- GTTGTACTAT ATAAATTCTA TGGTTATAAA CATAAGTAGT ATATTTTTAA CATCACAACG TTTTATAATT   
  
  
- TTAATAAAGT ATTTATTTGT AATAATATTC TTGACTGAGG CGCCTAATAG TATACCATAT CTAATGTCTC   
  
  
- ACCCACCCAA ATTTATTAAA GGGGCTGTGT AATATGAAAA TCTTCTCGAT AATCGAGGTT TAATAAATTT   
  
  
- GTTACAAGAT TTCTCGTTTT TTTAGTTACA AAACCTAAAA CTAGGCTGGC TCTCCTGAAC AGGCTGGTAA   
  
  
- GGTATAATTT TACAAGGATC AAGACTAATC GATTATGGT

+     Box 4

| Site Name | Organism | Position | Strand | Matrix score. | sequence | function |
| --- | --- | --- | --- | --- | --- | --- |
| Box 4 | Petroselinum crispum | 129 | + | 6 | ATTAAT | part of a conserved DNA module involved in light responsiveness |
| Box 4 | Petroselinum crispum | 442 | + | 6 | ATTAAT | part of a conserved DNA module involved in light responsiveness |
| Box 4 | Petroselinum crispum | 262 | + | 6 | ATTAAT | part of a conserved DNA module involved in light responsiveness |
| Box 4 | Petroselinum crispum | 1029 | - | 6 | ATTAAT | part of a conserved DNA module involved in light responsiveness |
| Box 4 | Petroselinum crispum | 258 | + | 6 | ATTAAT | part of a conserved DNA module involved in light responsiveness |
| Box 4 | Petroselinum crispum | 847 | + | 6 | ATTAAT | part of a conserved DNA module involved in light responsiveness |
| Box 4 | Petroselinum crispum | 346 | + | 6 | ATTAAT | part of a conserved DNA module involved in light responsiveness |

>PlantCARE\_4926   
+ TAAAGTTGTC GTGATTTCTT GCCCTTTATT TTTTATTTAT ATTAACATAT ATTTTTAAAA AAAAATTAAA   
  
  
+ GACGTTCCTA ACGGAAACTC ATGTATTTTT TGATTATGAA TATGAATATC CCCTCTATAT TAATCATGGA   
  
  
+ GCATTACAAC ATGTTTTCGT AGCCATATGT CATCACGAGA ATGATTTTTA GAATTGTTAG AAAAATAAAT   
  
  
+ TGATTCATAT AAACATATAC TATGTTTTTT ATTAAACTAA CTATCAAATT AATTAATAGT GTACAAAAAA   
  
  
+ ATATTTTTTT CTTTCCTTAA ATAAAAACTA CGGAATTACC TAATATGGCT AACATATATA TGACAATTAA   
  
  
+ TGATTATGAA TAATACATAT TTGATAAAAA AATTTCTAAC CTCTCTCTTT TTTGTTTAAT TTTATATTAT   
  
  
+ TAAAGGAAAT TTAACAATCA CATTAATCAT ATAATAAAAA CAATTAGATT TTTTCTTATA TGTTATATTT   
  
  
+ TGAATTTTTA AAAACGACTA TAAATTACTA AAAATGATAA GAGTCCCACA TTAAAAAATT TGTGATCAAC   
  
  
+ CGTTTAACTT TTTTTTTAGT TCAAGCAAGA TACAAATGAT CATATATCTG ATATAGACGT GGGCGTTCGG   
  
  
+ ATACACGTTC GGGTTTGTAT CAGATATTTC AGTATAAAGG TATAGAACCC GTTCGGGTAT TTCTACACTC   
  
  
+ CGAGTCGGGT TCGGGTTCGG ATATTTTGGA TCGGGTTCGA ATATTTAAAT TTTGAAGAAA AAAAGAAATT   
  
  
+ ATTCACTGTT TAAGTTTTTT ATATTTAAAT ATATCTTAAC TTAACTGATT TTTTTTAGTT TTTAAAAGAT   
  
  
+ TAAAATATTA ATATGTTTGG AGATAAAACT TTAAAAATAG AAAGACACTA ATTTAGTTTT TGTTTTGAAA   
  
  
+ ATTTAGATGC AACTTTTGTT AATGCAAGAA ACAAGAACTT GATATGTATT TTAAGTGAGT AACAAATGAT   
  
  
+ TTTGTCTATA GTTATATGTA TATTATCTAA TTTTGAGTAA TAAGAATCAT TAATATAAAT ATTTTGAATA   
  
  
+ AAATTAGATA GATAAACTAT AAATATAGAG TTAAGTATAC TTATGTTTGG TTATCTTCGA ATATTACCCG   
  
  
+ TTCGGATATA TTATCTGAAC TGGTGAAATA AGTAATATGT TTTGTTGTTT TAATTAGATA ATTTTTAGAC   
  
  
+ CGAGCTTGTG AATATATACT AGACAAACAT TTATATTTCG AGTCTGCACT TATATTCTAT AAGAGCTTGA   
  
  
+ TATATTAGAT TTGAACACTA ACCTGTTAAT ATAGTTTGCC GGTGATTTTT TTTCAAAATT TTGATTCTTA   
  
  
+ GATATGTATA TGGAGTAAAA CTAATTTTTA CAGATGCCCA TTTTTTTAAT TGACACTTAT GTAATTAACT   
  
  
+ GAATTCATAA AACAAGGTTT TTTAAAAAAA TTTAACTCAT ATCAATGAAA CAAAGACGAG AACGAAAGCA   
  
  
+ CAATTCTATG GAAATGGAAA ATGAAGTCAC TTATGGAGAT TCAATAGTAA GCAAATCGAG AGCAGAAAAT   
  
  
+ CTAATCTCCT TTCGTCATTA TACAATCAAT ATTGCCTATT TGGTTTTAGT GATTTGTTTC AGCCGCAAAA   
  
  
+ CTTAATTTTC TTTGGTGCAT ATGAAATCTT AAAAAGAATT AAAATAAGAT ATAATACGTT AACTCTTCAA   
  
  
+ CAACATGATA TATTTAAGAT ACCAATATTT GTATTCATCA TATAAAAATT GTAGTGTTGC AAAATATTAA   
  
  
+ AATTATTTCA TAAATAAACA TTATTATAAG AACTGACTCC GCGGATTATC ATATGGTATA GATTACAGAG   
  
  
+ TGGGTGGGTT TAAATAATTT CCCCGACACA TTATACTTTT AGAAGAGCTA TTAGCTCCAA ATTATTTAAA   
  
  
+ CAATGTTCTA AAGAGCAAAA AAATCAATGT TTTGGATTTT GATCCGACCG AGAGGACTTG TCCGACCATT   
  
  
+ CCATATTAAA ATGTTCCTAG TTCTGATTAG CTAATACCA  

- ATTTCAACAG CACTAAAGAA CGGGAAATAA AAAATAAATA TAATTGTATA TAAAAATTTT TTTTTAATTT   
  
  
- CTGCAAGGAT TGCCTTTGAG TACATAAAAA ACTAATACTT ATACTTATAG GGGAGATATA ATTAGTACCT   
  
  
- CGTAATGTTG TACAAAAGCA TCGGTATACA GTAGTGCTCT TACTAAAAAT CTTAACAATC TTTTTATTTA   
  
  
- ACTAAGTATA TTTGTATATG ATACAAAAAA TAATTTGATT GATAGTTTAA TTAATTATCA CATGTTTTTT   
  
  
- TATAAAAAAA GAAAGGAATT TATTTTTGAT GCCTTAATGG ATTATACCGA TTGTATATAT ACTGTTAATT   
  
  
- ACTAATACTT ATTATGTATA AACTATTTTT TTAAAGATTG GAGAGAGAAA AAACAAATTA AAATATAATA   
  
  
- ATTTCCTTTA AATTGTTAGT GTAATTAGTA TATTATTTTT GTTAATCTAA AAAAGAATAT ACAATATAAA   
  
  
- ACTTAAAAAT TTTTGCTGAT ATTTAATGAT TTTTACTATT CTCAGGGTGT AATTTTTTAA ACACTAGTTG   
  
  
- GCAAATTGAA AAAAAAATCA AGTTCGTTCT ATGTTTACTA GTATATAGAC TATATCTGCA CCCGCAAGCC   
  
  
- TATGTGCAAG CCCAAACATA GTCTATAAAG TCATATTTCC ATATCTTGGG CAAGCCCATA AAGATGTGAG   
  
  
- GCTCAGCCCA AGCCCAAGCC TATAAAACCT AGCCCAAGCT TATAAATTTA AAACTTCTTT TTTTCTTTAA   
  
  
- TAAGTGACAA ATTCAAAAAA TATAAATTTA TATAGAATTG AATTGACTAA AAAAAATCAA AAATTTTCTA   
  
  
- ATTTTATAAT TATACAAACC TCTATTTTGA AATTTTTATC TTTCTGTGAT TAAATCAAAA ACAAAACTTT   
  
  
- TAAATCTACG TTGAAAACAA TTACGTTCTT TGTTCTTGAA CTATACATAA AATTCACTCA TTGTTTACTA   
  
  
- AAACAGATAT CAATATACAT ATAATAGATT AAAACTCATT ATTCTTAGTA ATTATATTTA TAAAACTTAT   
  
  
- TTTAATCTAT CTATTTGATA TTTATATCTC AATTCATATG AATACAAACC AATAGAAGCT TATAATGGGC   
  
  
- AAGCCTATAT AATAGACTTG ACCACTTTAT TCATTATACA AAACAACAAA ATTAATCTAT TAAAAATCTG   
  
  
- GCTCGAACAC TTATATATGA TCTGTTTGTA AATATAAAGC TCAGACGTGA ATATAAGATA TTCTCGAACT   
  
  
- ATATAATCTA AACTTGTGAT TGGACAATTA TATCAAACGG CCACTAAAAA AAAGTTTTAA AACTAAGAAT   
  
  
- CTATACATAT ACCTCATTTT GATTAAAAAT GTCTACGGGT AAAAAAATTA ACTGTGAATA CATTAATTGA   
  
  
- CTTAAGTATT TTGTTCCAAA AAATTTTTTT AAATTGAGTA TAGTTACTTT GTTTCTGCTC TTGCTTTCGT   
  
  
- GTTAAGATAC CTTTACCTTT TACTTCAGTG AATACCTCTA AGTTATCATT CGTTTAGCTC TCGTCTTTTA   
  
  
- GATTAGAGGA AAGCAGTAAT ATGTTAGTTA TAACGGATAA ACCAAAATCA CTAAACAAAG TCGGCGTTTT   
  
  
- GAATTAAAAG AAACCACGTA TACTTTAGAA TTTTTCTTAA TTTTATTCTA TATTATGCAA TTGAGAAGTT   
  
  
- GTTGTACTAT ATAAATTCTA TGGTTATAAA CATAAGTAGT ATATTTTTAA CATCACAACG TTTTATAATT   
  
  
- TTAATAAAGT ATTTATTTGT AATAATATTC TTGACTGAGG CGCCTAATAG TATACCATAT CTAATGTCTC   
  
  
- ACCCACCCAA ATTTATTAAA GGGGCTGTGT AATATGAAAA TCTTCTCGAT AATCGAGGTT TAATAAATTT   
  
  
- GTTACAAGAT TTCTCGTTTT TTTAGTTACA AAACCTAAAA CTAGGCTGGC TCTCCTGAAC AGGCTGGTAA   
  
  
- GGTATAATTT TACAAGGATC AAGACTAATC GATTATGGT

+     CAAT-box

| Site Name | Organism | Position | Strand | Matrix score. | sequence | function |
| --- | --- | --- | --- | --- | --- | --- |
| CAAT-box | Nicotiana glutinosa | 193 | - | 4 | CAAT |  |
| CAAT-box | Nicotiana glutinosa | 209 | - | 4 | CAAT |  |
| CAAT-box | Pisum sativum | 255 | + | 5 | CAAAT | common cis-acting element in promoter and enhancer regions |
| CAAT-box | Nicotiana glutinosa | 344 | + | 4 | CAAT |  |
| CAAT-box | Pisum sativum | 369 | - | 5 | CAAAT | common cis-acting element in promoter and enhancer regions |
| CAAT-box | Nicotiana glutinosa | 435 | + | 4 | CAAT |  |
| CAAT-box | Nicotiana glutinosa | 461 | + | 4 | CAAT |  |
| CAAT-box | Pisum sativum | 548 | - | 5 | CAAAT | common cis-acting element in promoter and enhancer regions |
| CAAT-box | Pisum sativum | 593 | + | 5 | CAAAT | common cis-acting element in promoter and enhancer regions |
| CAAT-box | Pisum sativum | 973 | + | 5 | CAAAT | common cis-acting element in promoter and enhancer regions |
| CAAT-box | Pisum sativum | 1269 | - | 5 | CAAAT | common cis-acting element in promoter and enhancer regions |
| CAAT-box | Nicotiana glutinosa | 1379 | - | 4 | CAAT |  |
| CAAT-box | Nicotiana glutinosa | 1443 | + | 4 | CAAT |  |
| CAAT-box | Nicotiana glutinosa | 1471 | + | 4 | CAAT |  |
| CAAT-box | Nicotiana glutinosa | 1512 | + | 4 | CAAT |  |
| CAAT-box | Pisum sativum | 1522 | + | 5 | CAAAT | common cis-acting element in promoter and enhancer regions |
| CAAT-box | Nicotiana glutinosa | 1563 | + | 4 | CAAT |  |
| CAAT-box | Nicotiana glutinosa | 1567 | + | 4 | CAAT |  |
| CAAT-box | Nicotiana glutinosa | 1571 | - | 4 | CAAT |  |
| CAAT-box | Pisum sativum | 1578 | - | 5 | CAAAT | common cis-acting element in promoter and enhancer regions |
| CAAT-box | Pisum sativum | 1592 | - | 5 | CAAAT | common cis-acting element in promoter and enhancer regions |
| CAAT-box | Arabidopsis thaliana | 1702 | + | 5 | CCAAT | common cis-acting element in promoter and enhancer regions |
| CAAT-box | Nicotiana glutinosa | 1703 | + | 4 | CAAT |  |
| CAAT-box | Pisum sativum | 1707 | - | 5 | CAAAT | common cis-acting element in promoter and enhancer regions |
| CAAT-box | Nicotiana glutinosa | 1728 | - | 4 | CAAT |  |
| CAAT-box | Pisum sativum | 1878 | + | 5 | CAAAT | common cis-acting element in promoter and enhancer regions |
| CAAT-box | Nicotiana glutinosa | 1891 | + | 4 | CAAT |  |
| CAAT-box | Nicotiana glutinosa | 1915 | + | 4 | CAAT |  |

>PlantCARE\_4926   
+ TAAAGTTGTC GTGATTTCTT GCCCTTTATT TTTTATTTAT ATTAACATAT ATTTTTAAAA AAAAATTAAA   
  
  
+ GACGTTCCTA ACGGAAACTC ATGTATTTTT TGATTATGAA TATGAATATC CCCTCTATAT TAATCATGGA   
  
  
+ GCATTACAAC ATGTTTTCGT AGCCATATGT CATCACGAGA ATGATTTTTA GAATTGTTAG AAAAATAAAT   
  
  
+ TGATTCATAT AAACATATAC TATGTTTTTT ATTAAACTAA CTATCAAATT AATTAATAGT GTACAAAAAA   
  
  
+ ATATTTTTTT CTTTCCTTAA ATAAAAACTA CGGAATTACC TAATATGGCT AACATATATA TGACAATTAA   
  
  
+ TGATTATGAA TAATACATAT TTGATAAAAA AATTTCTAAC CTCTCTCTTT TTTGTTTAAT TTTATATTAT   
  
  
+ TAAAGGAAAT TTAACAATCA CATTAATCAT ATAATAAAAA CAATTAGATT TTTTCTTATA TGTTATATTT   
  
  
+ TGAATTTTTA AAAACGACTA TAAATTACTA AAAATGATAA GAGTCCCACA TTAAAAAATT TGTGATCAAC   
  
  
+ CGTTTAACTT TTTTTTTAGT TCAAGCAAGA TACAAATGAT CATATATCTG ATATAGACGT GGGCGTTCGG   
  
  
+ ATACACGTTC GGGTTTGTAT CAGATATTTC AGTATAAAGG TATAGAACCC GTTCGGGTAT TTCTACACTC   
  
  
+ CGAGTCGGGT TCGGGTTCGG ATATTTTGGA TCGGGTTCGA ATATTTAAAT TTTGAAGAAA AAAAGAAATT   
  
  
+ ATTCACTGTT TAAGTTTTTT ATATTTAAAT ATATCTTAAC TTAACTGATT TTTTTTAGTT TTTAAAAGAT   
  
  
+ TAAAATATTA ATATGTTTGG AGATAAAACT TTAAAAATAG AAAGACACTA ATTTAGTTTT TGTTTTGAAA   
  
  
+ ATTTAGATGC AACTTTTGTT AATGCAAGAA ACAAGAACTT GATATGTATT TTAAGTGAGT AACAAATGAT   
  
  
+ TTTGTCTATA GTTATATGTA TATTATCTAA TTTTGAGTAA TAAGAATCAT TAATATAAAT ATTTTGAATA   
  
  
+ AAATTAGATA GATAAACTAT AAATATAGAG TTAAGTATAC TTATGTTTGG TTATCTTCGA ATATTACCCG   
  
  
+ TTCGGATATA TTATCTGAAC TGGTGAAATA AGTAATATGT TTTGTTGTTT TAATTAGATA ATTTTTAGAC   
  
  
+ CGAGCTTGTG AATATATACT AGACAAACAT TTATATTTCG AGTCTGCACT TATATTCTAT AAGAGCTTGA   
  
  
+ TATATTAGAT TTGAACACTA ACCTGTTAAT ATAGTTTGCC GGTGATTTTT TTTCAAAATT TTGATTCTTA   
  
  
+ GATATGTATA TGGAGTAAAA CTAATTTTTA CAGATGCCCA TTTTTTTAAT TGACACTTAT GTAATTAACT   
  
  
+ GAATTCATAA AACAAGGTTT TTTAAAAAAA TTTAACTCAT ATCAATGAAA CAAAGACGAG AACGAAAGCA   
  
  
+ CAATTCTATG GAAATGGAAA ATGAAGTCAC TTATGGAGAT TCAATAGTAA GCAAATCGAG AGCAGAAAAT   
  
  
+ CTAATCTCCT TTCGTCATTA TACAATCAAT ATTGCCTATT TGGTTTTAGT GATTTGTTTC AGCCGCAAAA   
  
  
+ CTTAATTTTC TTTGGTGCAT ATGAAATCTT AAAAAGAATT AAAATAAGAT ATAATACGTT AACTCTTCAA   
  
  
+ CAACATGATA TATTTAAGAT ACCAATATTT GTATTCATCA TATAAAAATT GTAGTGTTGC AAAATATTAA   
  
  
+ AATTATTTCA TAAATAAACA TTATTATAAG AACTGACTCC GCGGATTATC ATATGGTATA GATTACAGAG   
  
  
+ TGGGTGGGTT TAAATAATTT CCCCGACACA TTATACTTTT AGAAGAGCTA TTAGCTCCAA ATTATTTAAA   
  
  
+ CAATGTTCTA AAGAGCAAAA AAATCAATGT TTTGGATTTT GATCCGACCG AGAGGACTTG TCCGACCATT   
  
  
+ CCATATTAAA ATGTTCCTAG TTCTGATTAG CTAATACCA  

- ATTTCAACAG CACTAAAGAA CGGGAAATAA AAAATAAATA TAATTGTATA TAAAAATTTT TTTTTAATTT   
  
  
- CTGCAAGGAT TGCCTTTGAG TACATAAAAA ACTAATACTT ATACTTATAG GGGAGATATA ATTAGTACCT   
  
  
- CGTAATGTTG TACAAAAGCA TCGGTATACA GTAGTGCTCT TACTAAAAAT CTTAACAATC TTTTTATTTA   
  
  
- ACTAAGTATA TTTGTATATG ATACAAAAAA TAATTTGATT GATAGTTTAA TTAATTATCA CATGTTTTTT   
  
  
- TATAAAAAAA GAAAGGAATT TATTTTTGAT GCCTTAATGG ATTATACCGA TTGTATATAT ACTGTTAATT   
  
  
- ACTAATACTT ATTATGTATA AACTATTTTT TTAAAGATTG GAGAGAGAAA AAACAAATTA AAATATAATA   
  
  
- ATTTCCTTTA AATTGTTAGT GTAATTAGTA TATTATTTTT GTTAATCTAA AAAAGAATAT ACAATATAAA   
  
  
- ACTTAAAAAT TTTTGCTGAT ATTTAATGAT TTTTACTATT CTCAGGGTGT AATTTTTTAA ACACTAGTTG   
  
  
- GCAAATTGAA AAAAAAATCA AGTTCGTTCT ATGTTTACTA GTATATAGAC TATATCTGCA CCCGCAAGCC   
  
  
- TATGTGCAAG CCCAAACATA GTCTATAAAG TCATATTTCC ATATCTTGGG CAAGCCCATA AAGATGTGAG   
  
  
- GCTCAGCCCA AGCCCAAGCC TATAAAACCT AGCCCAAGCT TATAAATTTA AAACTTCTTT TTTTCTTTAA   
  
  
- TAAGTGACAA ATTCAAAAAA TATAAATTTA TATAGAATTG AATTGACTAA AAAAAATCAA AAATTTTCTA   
  
  
- ATTTTATAAT TATACAAACC TCTATTTTGA AATTTTTATC TTTCTGTGAT TAAATCAAAA ACAAAACTTT   
  
  
- TAAATCTACG TTGAAAACAA TTACGTTCTT TGTTCTTGAA CTATACATAA AATTCACTCA TTGTTTACTA   
  
  
- AAACAGATAT CAATATACAT ATAATAGATT AAAACTCATT ATTCTTAGTA ATTATATTTA TAAAACTTAT   
  
  
- TTTAATCTAT CTATTTGATA TTTATATCTC AATTCATATG AATACAAACC AATAGAAGCT TATAATGGGC   
  
  
- AAGCCTATAT AATAGACTTG ACCACTTTAT TCATTATACA AAACAACAAA ATTAATCTAT TAAAAATCTG   
  
  
- GCTCGAACAC TTATATATGA TCTGTTTGTA AATATAAAGC TCAGACGTGA ATATAAGATA TTCTCGAACT   
  
  
- ATATAATCTA AACTTGTGAT TGGACAATTA TATCAAACGG CCACTAAAAA AAAGTTTTAA AACTAAGAAT   
  
  
- CTATACATAT ACCTCATTTT GATTAAAAAT GTCTACGGGT AAAAAAATTA ACTGTGAATA CATTAATTGA   
  
  
- CTTAAGTATT TTGTTCCAAA AAATTTTTTT AAATTGAGTA TAGTTACTTT GTTTCTGCTC TTGCTTTCGT   
  
  
- GTTAAGATAC CTTTACCTTT TACTTCAGTG AATACCTCTA AGTTATCATT CGTTTAGCTC TCGTCTTTTA   
  
  
- GATTAGAGGA AAGCAGTAAT ATGTTAGTTA TAACGGATAA ACCAAAATCA CTAAACAAAG TCGGCGTTTT   
  
  
- GAATTAAAAG AAACCACGTA TACTTTAGAA TTTTTCTTAA TTTTATTCTA TATTATGCAA TTGAGAAGTT   
  
  
- GTTGTACTAT ATAAATTCTA TGGTTATAAA CATAAGTAGT ATATTTTTAA CATCACAACG TTTTATAATT   
  
  
- TTAATAAAGT ATTTATTTGT AATAATATTC TTGACTGAGG CGCCTAATAG TATACCATAT CTAATGTCTC   
  
  
- ACCCACCCAA ATTTATTAAA GGGGCTGTGT AATATGAAAA TCTTCTCGAT AATCGAGGTT TAATAAATTT   
  
  
- GTTACAAGAT TTCTCGTTTT TTTAGTTACA AAACCTAAAA CTAGGCTGGC TCTCCTGAAC AGGCTGGTAA   
  
  
- GGTATAATTT TACAAGGATC AAGACTAATC GATTATGGT

+     CGTCA-motif

| Site Name | Organism | Position | Strand | Matrix score. | sequence | function |
| --- | --- | --- | --- | --- | --- | --- |
| CGTCA-motif | Hordeum vulgare | 1553 | + | 5 | CGTCA | cis-acting regulatory element involved in the MeJA-responsiveness |

>PlantCARE\_4926   
+ TAAAGTTGTC GTGATTTCTT GCCCTTTATT TTTTATTTAT ATTAACATAT ATTTTTAAAA AAAAATTAAA   
  
  
+ GACGTTCCTA ACGGAAACTC ATGTATTTTT TGATTATGAA TATGAATATC CCCTCTATAT TAATCATGGA   
  
  
+ GCATTACAAC ATGTTTTCGT AGCCATATGT CATCACGAGA ATGATTTTTA GAATTGTTAG AAAAATAAAT   
  
  
+ TGATTCATAT AAACATATAC TATGTTTTTT ATTAAACTAA CTATCAAATT AATTAATAGT GTACAAAAAA   
  
  
+ ATATTTTTTT CTTTCCTTAA ATAAAAACTA CGGAATTACC TAATATGGCT AACATATATA TGACAATTAA   
  
  
+ TGATTATGAA TAATACATAT TTGATAAAAA AATTTCTAAC CTCTCTCTTT TTTGTTTAAT TTTATATTAT   
  
  
+ TAAAGGAAAT TTAACAATCA CATTAATCAT ATAATAAAAA CAATTAGATT TTTTCTTATA TGTTATATTT   
  
  
+ TGAATTTTTA AAAACGACTA TAAATTACTA AAAATGATAA GAGTCCCACA TTAAAAAATT TGTGATCAAC   
  
  
+ CGTTTAACTT TTTTTTTAGT TCAAGCAAGA TACAAATGAT CATATATCTG ATATAGACGT GGGCGTTCGG   
  
  
+ ATACACGTTC GGGTTTGTAT CAGATATTTC AGTATAAAGG TATAGAACCC GTTCGGGTAT TTCTACACTC   
  
  
+ CGAGTCGGGT TCGGGTTCGG ATATTTTGGA TCGGGTTCGA ATATTTAAAT TTTGAAGAAA AAAAGAAATT   
  
  
+ ATTCACTGTT TAAGTTTTTT ATATTTAAAT ATATCTTAAC TTAACTGATT TTTTTTAGTT TTTAAAAGAT   
  
  
+ TAAAATATTA ATATGTTTGG AGATAAAACT TTAAAAATAG AAAGACACTA ATTTAGTTTT TGTTTTGAAA   
  
  
+ ATTTAGATGC AACTTTTGTT AATGCAAGAA ACAAGAACTT GATATGTATT TTAAGTGAGT AACAAATGAT   
  
  
+ TTTGTCTATA GTTATATGTA TATTATCTAA TTTTGAGTAA TAAGAATCAT TAATATAAAT ATTTTGAATA   
  
  
+ AAATTAGATA GATAAACTAT AAATATAGAG TTAAGTATAC TTATGTTTGG TTATCTTCGA ATATTACCCG   
  
  
+ TTCGGATATA TTATCTGAAC TGGTGAAATA AGTAATATGT TTTGTTGTTT TAATTAGATA ATTTTTAGAC   
  
  
+ CGAGCTTGTG AATATATACT AGACAAACAT TTATATTTCG AGTCTGCACT TATATTCTAT AAGAGCTTGA   
  
  
+ TATATTAGAT TTGAACACTA ACCTGTTAAT ATAGTTTGCC GGTGATTTTT TTTCAAAATT TTGATTCTTA   
  
  
+ GATATGTATA TGGAGTAAAA CTAATTTTTA CAGATGCCCA TTTTTTTAAT TGACACTTAT GTAATTAACT   
  
  
+ GAATTCATAA AACAAGGTTT TTTAAAAAAA TTTAACTCAT ATCAATGAAA CAAAGACGAG AACGAAAGCA   
  
  
+ CAATTCTATG GAAATGGAAA ATGAAGTCAC TTATGGAGAT TCAATAGTAA GCAAATCGAG AGCAGAAAAT   
  
  
+ CTAATCTCCT TTCGTCATTA TACAATCAAT ATTGCCTATT TGGTTTTAGT GATTTGTTTC AGCCGCAAAA   
  
  
+ CTTAATTTTC TTTGGTGCAT ATGAAATCTT AAAAAGAATT AAAATAAGAT ATAATACGTT AACTCTTCAA   
  
  
+ CAACATGATA TATTTAAGAT ACCAATATTT GTATTCATCA TATAAAAATT GTAGTGTTGC AAAATATTAA   
  
  
+ AATTATTTCA TAAATAAACA TTATTATAAG AACTGACTCC GCGGATTATC ATATGGTATA GATTACAGAG   
  
  
+ TGGGTGGGTT TAAATAATTT CCCCGACACA TTATACTTTT AGAAGAGCTA TTAGCTCCAA ATTATTTAAA   
  
  
+ CAATGTTCTA AAGAGCAAAA AAATCAATGT TTTGGATTTT GATCCGACCG AGAGGACTTG TCCGACCATT   
  
  
+ CCATATTAAA ATGTTCCTAG TTCTGATTAG CTAATACCA  

- ATTTCAACAG CACTAAAGAA CGGGAAATAA AAAATAAATA TAATTGTATA TAAAAATTTT TTTTTAATTT   
  
  
- CTGCAAGGAT TGCCTTTGAG TACATAAAAA ACTAATACTT ATACTTATAG GGGAGATATA ATTAGTACCT   
  
  
- CGTAATGTTG TACAAAAGCA TCGGTATACA GTAGTGCTCT TACTAAAAAT CTTAACAATC TTTTTATTTA   
  
  
- ACTAAGTATA TTTGTATATG ATACAAAAAA TAATTTGATT GATAGTTTAA TTAATTATCA CATGTTTTTT   
  
  
- TATAAAAAAA GAAAGGAATT TATTTTTGAT GCCTTAATGG ATTATACCGA TTGTATATAT ACTGTTAATT   
  
  
- ACTAATACTT ATTATGTATA AACTATTTTT TTAAAGATTG GAGAGAGAAA AAACAAATTA AAATATAATA   
  
  
- ATTTCCTTTA AATTGTTAGT GTAATTAGTA TATTATTTTT GTTAATCTAA AAAAGAATAT ACAATATAAA   
  
  
- ACTTAAAAAT TTTTGCTGAT ATTTAATGAT TTTTACTATT CTCAGGGTGT AATTTTTTAA ACACTAGTTG   
  
  
- GCAAATTGAA AAAAAAATCA AGTTCGTTCT ATGTTTACTA GTATATAGAC TATATCTGCA CCCGCAAGCC   
  
  
- TATGTGCAAG CCCAAACATA GTCTATAAAG TCATATTTCC ATATCTTGGG CAAGCCCATA AAGATGTGAG   
  
  
- GCTCAGCCCA AGCCCAAGCC TATAAAACCT AGCCCAAGCT TATAAATTTA AAACTTCTTT TTTTCTTTAA   
  
  
- TAAGTGACAA ATTCAAAAAA TATAAATTTA TATAGAATTG AATTGACTAA AAAAAATCAA AAATTTTCTA   
  
  
- ATTTTATAAT TATACAAACC TCTATTTTGA AATTTTTATC TTTCTGTGAT TAAATCAAAA ACAAAACTTT   
  
  
- TAAATCTACG TTGAAAACAA TTACGTTCTT TGTTCTTGAA CTATACATAA AATTCACTCA TTGTTTACTA   
  
  
- AAACAGATAT CAATATACAT ATAATAGATT AAAACTCATT ATTCTTAGTA ATTATATTTA TAAAACTTAT   
  
  
- TTTAATCTAT CTATTTGATA TTTATATCTC AATTCATATG AATACAAACC AATAGAAGCT TATAATGGGC   
  
  
- AAGCCTATAT AATAGACTTG ACCACTTTAT TCATTATACA AAACAACAAA ATTAATCTAT TAAAAATCTG   
  
  
- GCTCGAACAC TTATATATGA TCTGTTTGTA AATATAAAGC TCAGACGTGA ATATAAGATA TTCTCGAACT   
  
  
- ATATAATCTA AACTTGTGAT TGGACAATTA TATCAAACGG CCACTAAAAA AAAGTTTTAA AACTAAGAAT   
  
  
- CTATACATAT ACCTCATTTT GATTAAAAAT GTCTACGGGT AAAAAAATTA ACTGTGAATA CATTAATTGA   
  
  
- CTTAAGTATT TTGTTCCAAA AAATTTTTTT AAATTGAGTA TAGTTACTTT GTTTCTGCTC TTGCTTTCGT   
  
  
- GTTAAGATAC CTTTACCTTT TACTTCAGTG AATACCTCTA AGTTATCATT CGTTTAGCTC TCGTCTTTTA   
  
  
- GATTAGAGGA AAGCAGTAAT ATGTTAGTTA TAACGGATAA ACCAAAATCA CTAAACAAAG TCGGCGTTTT   
  
  
- GAATTAAAAG AAACCACGTA TACTTTAGAA TTTTTCTTAA TTTTATTCTA TATTATGCAA TTGAGAAGTT   
  
  
- GTTGTACTAT ATAAATTCTA TGGTTATAAA CATAAGTAGT ATATTTTTAA CATCACAACG TTTTATAATT   
  
  
- TTAATAAAGT ATTTATTTGT AATAATATTC TTGACTGAGG CGCCTAATAG TATACCATAT CTAATGTCTC   
  
  
- ACCCACCCAA ATTTATTAAA GGGGCTGTGT AATATGAAAA TCTTCTCGAT AATCGAGGTT TAATAAATTT   
  
  
- GTTACAAGAT TTCTCGTTTT TTTAGTTACA AAACCTAAAA CTAGGCTGGC TCTCCTGAAC AGGCTGGTAA   
  
  
- GGTATAATTT TACAAGGATC AAGACTAATC GATTATGGT

+     DRE1

| Site Name | Organism | Position | Strand | Matrix score. | sequence | function |
| --- | --- | --- | --- | --- | --- | --- |
| DRE1 | Zea mays | 1937 | + | 7 | ACCGAGA |  |

>PlantCARE\_4926   
+ TAAAGTTGTC GTGATTTCTT GCCCTTTATT TTTTATTTAT ATTAACATAT ATTTTTAAAA AAAAATTAAA   
  
  
+ GACGTTCCTA ACGGAAACTC ATGTATTTTT TGATTATGAA TATGAATATC CCCTCTATAT TAATCATGGA   
  
  
+ GCATTACAAC ATGTTTTCGT AGCCATATGT CATCACGAGA ATGATTTTTA GAATTGTTAG AAAAATAAAT   
  
  
+ TGATTCATAT AAACATATAC TATGTTTTTT ATTAAACTAA CTATCAAATT AATTAATAGT GTACAAAAAA   
  
  
+ ATATTTTTTT CTTTCCTTAA ATAAAAACTA CGGAATTACC TAATATGGCT AACATATATA TGACAATTAA   
  
  
+ TGATTATGAA TAATACATAT TTGATAAAAA AATTTCTAAC CTCTCTCTTT TTTGTTTAAT TTTATATTAT   
  
  
+ TAAAGGAAAT TTAACAATCA CATTAATCAT ATAATAAAAA CAATTAGATT TTTTCTTATA TGTTATATTT   
  
  
+ TGAATTTTTA AAAACGACTA TAAATTACTA AAAATGATAA GAGTCCCACA TTAAAAAATT TGTGATCAAC   
  
  
+ CGTTTAACTT TTTTTTTAGT TCAAGCAAGA TACAAATGAT CATATATCTG ATATAGACGT GGGCGTTCGG   
  
  
+ ATACACGTTC GGGTTTGTAT CAGATATTTC AGTATAAAGG TATAGAACCC GTTCGGGTAT TTCTACACTC   
  
  
+ CGAGTCGGGT TCGGGTTCGG ATATTTTGGA TCGGGTTCGA ATATTTAAAT TTTGAAGAAA AAAAGAAATT   
  
  
+ ATTCACTGTT TAAGTTTTTT ATATTTAAAT ATATCTTAAC TTAACTGATT TTTTTTAGTT TTTAAAAGAT   
  
  
+ TAAAATATTA ATATGTTTGG AGATAAAACT TTAAAAATAG AAAGACACTA ATTTAGTTTT TGTTTTGAAA   
  
  
+ ATTTAGATGC AACTTTTGTT AATGCAAGAA ACAAGAACTT GATATGTATT TTAAGTGAGT AACAAATGAT   
  
  
+ TTTGTCTATA GTTATATGTA TATTATCTAA TTTTGAGTAA TAAGAATCAT TAATATAAAT ATTTTGAATA   
  
  
+ AAATTAGATA GATAAACTAT AAATATAGAG TTAAGTATAC TTATGTTTGG TTATCTTCGA ATATTACCCG   
  
  
+ TTCGGATATA TTATCTGAAC TGGTGAAATA AGTAATATGT TTTGTTGTTT TAATTAGATA ATTTTTAGAC   
  
  
+ CGAGCTTGTG AATATATACT AGACAAACAT TTATATTTCG AGTCTGCACT TATATTCTAT AAGAGCTTGA   
  
  
+ TATATTAGAT TTGAACACTA ACCTGTTAAT ATAGTTTGCC GGTGATTTTT TTTCAAAATT TTGATTCTTA   
  
  
+ GATATGTATA TGGAGTAAAA CTAATTTTTA CAGATGCCCA TTTTTTTAAT TGACACTTAT GTAATTAACT   
  
  
+ GAATTCATAA AACAAGGTTT TTTAAAAAAA TTTAACTCAT ATCAATGAAA CAAAGACGAG AACGAAAGCA   
  
  
+ CAATTCTATG GAAATGGAAA ATGAAGTCAC TTATGGAGAT TCAATAGTAA GCAAATCGAG AGCAGAAAAT   
  
  
+ CTAATCTCCT TTCGTCATTA TACAATCAAT ATTGCCTATT TGGTTTTAGT GATTTGTTTC AGCCGCAAAA   
  
  
+ CTTAATTTTC TTTGGTGCAT ATGAAATCTT AAAAAGAATT AAAATAAGAT ATAATACGTT AACTCTTCAA   
  
  
+ CAACATGATA TATTTAAGAT ACCAATATTT GTATTCATCA TATAAAAATT GTAGTGTTGC AAAATATTAA   
  
  
+ AATTATTTCA TAAATAAACA TTATTATAAG AACTGACTCC GCGGATTATC ATATGGTATA GATTACAGAG   
  
  
+ TGGGTGGGTT TAAATAATTT CCCCGACACA TTATACTTTT AGAAGAGCTA TTAGCTCCAA ATTATTTAAA   
  
  
+ CAATGTTCTA AAGAGCAAAA AAATCAATGT TTTGGATTTT GATCCGACCG AGAGGACTTG TCCGACCATT   
  
  
+ CCATATTAAA ATGTTCCTAG TTCTGATTAG CTAATACCA  

- ATTTCAACAG CACTAAAGAA CGGGAAATAA AAAATAAATA TAATTGTATA TAAAAATTTT TTTTTAATTT   
  
  
- CTGCAAGGAT TGCCTTTGAG TACATAAAAA ACTAATACTT ATACTTATAG GGGAGATATA ATTAGTACCT   
  
  
- CGTAATGTTG TACAAAAGCA TCGGTATACA GTAGTGCTCT TACTAAAAAT CTTAACAATC TTTTTATTTA   
  
  
- ACTAAGTATA TTTGTATATG ATACAAAAAA TAATTTGATT GATAGTTTAA TTAATTATCA CATGTTTTTT   
  
  
- TATAAAAAAA GAAAGGAATT TATTTTTGAT GCCTTAATGG ATTATACCGA TTGTATATAT ACTGTTAATT   
  
  
- ACTAATACTT ATTATGTATA AACTATTTTT TTAAAGATTG GAGAGAGAAA AAACAAATTA AAATATAATA   
  
  
- ATTTCCTTTA AATTGTTAGT GTAATTAGTA TATTATTTTT GTTAATCTAA AAAAGAATAT ACAATATAAA   
  
  
- ACTTAAAAAT TTTTGCTGAT ATTTAATGAT TTTTACTATT CTCAGGGTGT AATTTTTTAA ACACTAGTTG   
  
  
- GCAAATTGAA AAAAAAATCA AGTTCGTTCT ATGTTTACTA GTATATAGAC TATATCTGCA CCCGCAAGCC   
  
  
- TATGTGCAAG CCCAAACATA GTCTATAAAG TCATATTTCC ATATCTTGGG CAAGCCCATA AAGATGTGAG   
  
  
- GCTCAGCCCA AGCCCAAGCC TATAAAACCT AGCCCAAGCT TATAAATTTA AAACTTCTTT TTTTCTTTAA   
  
  
- TAAGTGACAA ATTCAAAAAA TATAAATTTA TATAGAATTG AATTGACTAA AAAAAATCAA AAATTTTCTA   
  
  
- ATTTTATAAT TATACAAACC TCTATTTTGA AATTTTTATC TTTCTGTGAT TAAATCAAAA ACAAAACTTT   
  
  
- TAAATCTACG TTGAAAACAA TTACGTTCTT TGTTCTTGAA CTATACATAA AATTCACTCA TTGTTTACTA   
  
  
- AAACAGATAT CAATATACAT ATAATAGATT AAAACTCATT ATTCTTAGTA ATTATATTTA TAAAACTTAT   
  
  
- TTTAATCTAT CTATTTGATA TTTATATCTC AATTCATATG AATACAAACC AATAGAAGCT TATAATGGGC   
  
  
- AAGCCTATAT AATAGACTTG ACCACTTTAT TCATTATACA AAACAACAAA ATTAATCTAT TAAAAATCTG   
  
  
- GCTCGAACAC TTATATATGA TCTGTTTGTA AATATAAAGC TCAGACGTGA ATATAAGATA TTCTCGAACT   
  
  
- ATATAATCTA AACTTGTGAT TGGACAATTA TATCAAACGG CCACTAAAAA AAAGTTTTAA AACTAAGAAT   
  
  
- CTATACATAT ACCTCATTTT GATTAAAAAT GTCTACGGGT AAAAAAATTA ACTGTGAATA CATTAATTGA   
  
  
- CTTAAGTATT TTGTTCCAAA AAATTTTTTT AAATTGAGTA TAGTTACTTT GTTTCTGCTC TTGCTTTCGT   
  
  
- GTTAAGATAC CTTTACCTTT TACTTCAGTG AATACCTCTA AGTTATCATT CGTTTAGCTC TCGTCTTTTA   
  
  
- GATTAGAGGA AAGCAGTAAT ATGTTAGTTA TAACGGATAA ACCAAAATCA CTAAACAAAG TCGGCGTTTT   
  
  
- GAATTAAAAG AAACCACGTA TACTTTAGAA TTTTTCTTAA TTTTATTCTA TATTATGCAA TTGAGAAGTT   
  
  
- GTTGTACTAT ATAAATTCTA TGGTTATAAA CATAAGTAGT ATATTTTTAA CATCACAACG TTTTATAATT   
  
  
- TTAATAAAGT ATTTATTTGT AATAATATTC TTGACTGAGG CGCCTAATAG TATACCATAT CTAATGTCTC   
  
  
- ACCCACCCAA ATTTATTAAA GGGGCTGTGT AATATGAAAA TCTTCTCGAT AATCGAGGTT TAATAAATTT   
  
  
- GTTACAAGAT TTCTCGTTTT TTTAGTTACA AAACCTAAAA CTAGGCTGGC TCTCCTGAAC AGGCTGGTAA   
  
  
- GGTATAATTT TACAAGGATC AAGACTAATC GATTATGGT

+     ERE

| Site Name | Organism | Position | Strand | Matrix score. | sequence | function |
| --- | --- | --- | --- | --- | --- | --- |
| ERE | Nicotiana glutinos | 1630 | - | 8 | ATTTCATA |  |
| ERE | Nicotiana glutinos | 1755 | + | 8 | ATTTCATA |  |

>PlantCARE\_4926   
+ TAAAGTTGTC GTGATTTCTT GCCCTTTATT TTTTATTTAT ATTAACATAT ATTTTTAAAA AAAAATTAAA   
  
  
+ GACGTTCCTA ACGGAAACTC ATGTATTTTT TGATTATGAA TATGAATATC CCCTCTATAT TAATCATGGA   
  
  
+ GCATTACAAC ATGTTTTCGT AGCCATATGT CATCACGAGA ATGATTTTTA GAATTGTTAG AAAAATAAAT   
  
  
+ TGATTCATAT AAACATATAC TATGTTTTTT ATTAAACTAA CTATCAAATT AATTAATAGT GTACAAAAAA   
  
  
+ ATATTTTTTT CTTTCCTTAA ATAAAAACTA CGGAATTACC TAATATGGCT AACATATATA TGACAATTAA   
  
  
+ TGATTATGAA TAATACATAT TTGATAAAAA AATTTCTAAC CTCTCTCTTT TTTGTTTAAT TTTATATTAT   
  
  
+ TAAAGGAAAT TTAACAATCA CATTAATCAT ATAATAAAAA CAATTAGATT TTTTCTTATA TGTTATATTT   
  
  
+ TGAATTTTTA AAAACGACTA TAAATTACTA AAAATGATAA GAGTCCCACA TTAAAAAATT TGTGATCAAC   
  
  
+ CGTTTAACTT TTTTTTTAGT TCAAGCAAGA TACAAATGAT CATATATCTG ATATAGACGT GGGCGTTCGG   
  
  
+ ATACACGTTC GGGTTTGTAT CAGATATTTC AGTATAAAGG TATAGAACCC GTTCGGGTAT TTCTACACTC   
  
  
+ CGAGTCGGGT TCGGGTTCGG ATATTTTGGA TCGGGTTCGA ATATTTAAAT TTTGAAGAAA AAAAGAAATT   
  
  
+ ATTCACTGTT TAAGTTTTTT ATATTTAAAT ATATCTTAAC TTAACTGATT TTTTTTAGTT TTTAAAAGAT   
  
  
+ TAAAATATTA ATATGTTTGG AGATAAAACT TTAAAAATAG AAAGACACTA ATTTAGTTTT TGTTTTGAAA   
  
  
+ ATTTAGATGC AACTTTTGTT AATGCAAGAA ACAAGAACTT GATATGTATT TTAAGTGAGT AACAAATGAT   
  
  
+ TTTGTCTATA GTTATATGTA TATTATCTAA TTTTGAGTAA TAAGAATCAT TAATATAAAT ATTTTGAATA   
  
  
+ AAATTAGATA GATAAACTAT AAATATAGAG TTAAGTATAC TTATGTTTGG TTATCTTCGA ATATTACCCG   
  
  
+ TTCGGATATA TTATCTGAAC TGGTGAAATA AGTAATATGT TTTGTTGTTT TAATTAGATA ATTTTTAGAC   
  
  
+ CGAGCTTGTG AATATATACT AGACAAACAT TTATATTTCG AGTCTGCACT TATATTCTAT AAGAGCTTGA   
  
  
+ TATATTAGAT TTGAACACTA ACCTGTTAAT ATAGTTTGCC GGTGATTTTT TTTCAAAATT TTGATTCTTA   
  
  
+ GATATGTATA TGGAGTAAAA CTAATTTTTA CAGATGCCCA TTTTTTTAAT TGACACTTAT GTAATTAACT   
  
  
+ GAATTCATAA AACAAGGTTT TTTAAAAAAA TTTAACTCAT ATCAATGAAA CAAAGACGAG AACGAAAGCA   
  
  
+ CAATTCTATG GAAATGGAAA ATGAAGTCAC TTATGGAGAT TCAATAGTAA GCAAATCGAG AGCAGAAAAT   
  
  
+ CTAATCTCCT TTCGTCATTA TACAATCAAT ATTGCCTATT TGGTTTTAGT GATTTGTTTC AGCCGCAAAA   
  
  
+ CTTAATTTTC TTTGGTGCAT ATGAAATCTT AAAAAGAATT AAAATAAGAT ATAATACGTT AACTCTTCAA   
  
  
+ CAACATGATA TATTTAAGAT ACCAATATTT GTATTCATCA TATAAAAATT GTAGTGTTGC AAAATATTAA   
  
  
+ AATTATTTCA TAAATAAACA TTATTATAAG AACTGACTCC GCGGATTATC ATATGGTATA GATTACAGAG   
  
  
+ TGGGTGGGTT TAAATAATTT CCCCGACACA TTATACTTTT AGAAGAGCTA TTAGCTCCAA ATTATTTAAA   
  
  
+ CAATGTTCTA AAGAGCAAAA AAATCAATGT TTTGGATTTT GATCCGACCG AGAGGACTTG TCCGACCATT   
  
  
+ CCATATTAAA ATGTTCCTAG TTCTGATTAG CTAATACCA  

- ATTTCAACAG CACTAAAGAA CGGGAAATAA AAAATAAATA TAATTGTATA TAAAAATTTT TTTTTAATTT   
  
  
- CTGCAAGGAT TGCCTTTGAG TACATAAAAA ACTAATACTT ATACTTATAG GGGAGATATA ATTAGTACCT   
  
  
- CGTAATGTTG TACAAAAGCA TCGGTATACA GTAGTGCTCT TACTAAAAAT CTTAACAATC TTTTTATTTA   
  
  
- ACTAAGTATA TTTGTATATG ATACAAAAAA TAATTTGATT GATAGTTTAA TTAATTATCA CATGTTTTTT   
  
  
- TATAAAAAAA GAAAGGAATT TATTTTTGAT GCCTTAATGG ATTATACCGA TTGTATATAT ACTGTTAATT   
  
  
- ACTAATACTT ATTATGTATA AACTATTTTT TTAAAGATTG GAGAGAGAAA AAACAAATTA AAATATAATA   
  
  
- ATTTCCTTTA AATTGTTAGT GTAATTAGTA TATTATTTTT GTTAATCTAA AAAAGAATAT ACAATATAAA   
  
  
- ACTTAAAAAT TTTTGCTGAT ATTTAATGAT TTTTACTATT CTCAGGGTGT AATTTTTTAA ACACTAGTTG   
  
  
- GCAAATTGAA AAAAAAATCA AGTTCGTTCT ATGTTTACTA GTATATAGAC TATATCTGCA CCCGCAAGCC   
  
  
- TATGTGCAAG CCCAAACATA GTCTATAAAG TCATATTTCC ATATCTTGGG CAAGCCCATA AAGATGTGAG   
  
  
- GCTCAGCCCA AGCCCAAGCC TATAAAACCT AGCCCAAGCT TATAAATTTA AAACTTCTTT TTTTCTTTAA   
  
  
- TAAGTGACAA ATTCAAAAAA TATAAATTTA TATAGAATTG AATTGACTAA AAAAAATCAA AAATTTTCTA   
  
  
- ATTTTATAAT TATACAAACC TCTATTTTGA AATTTTTATC TTTCTGTGAT TAAATCAAAA ACAAAACTTT   
  
  
- TAAATCTACG TTGAAAACAA TTACGTTCTT TGTTCTTGAA CTATACATAA AATTCACTCA TTGTTTACTA   
  
  
- AAACAGATAT CAATATACAT ATAATAGATT AAAACTCATT ATTCTTAGTA ATTATATTTA TAAAACTTAT   
  
  
- TTTAATCTAT CTATTTGATA TTTATATCTC AATTCATATG AATACAAACC AATAGAAGCT TATAATGGGC   
  
  
- AAGCCTATAT AATAGACTTG ACCACTTTAT TCATTATACA AAACAACAAA ATTAATCTAT TAAAAATCTG   
  
  
- GCTCGAACAC TTATATATGA TCTGTTTGTA AATATAAAGC TCAGACGTGA ATATAAGATA TTCTCGAACT   
  
  
- ATATAATCTA AACTTGTGAT TGGACAATTA TATCAAACGG CCACTAAAAA AAAGTTTTAA AACTAAGAAT   
  
  
- CTATACATAT ACCTCATTTT GATTAAAAAT GTCTACGGGT AAAAAAATTA ACTGTGAATA CATTAATTGA   
  
  
- CTTAAGTATT TTGTTCCAAA AAATTTTTTT AAATTGAGTA TAGTTACTTT GTTTCTGCTC TTGCTTTCGT   
  
  
- GTTAAGATAC CTTTACCTTT TACTTCAGTG AATACCTCTA AGTTATCATT CGTTTAGCTC TCGTCTTTTA   
  
  
- GATTAGAGGA AAGCAGTAAT ATGTTAGTTA TAACGGATAA ACCAAAATCA CTAAACAAAG TCGGCGTTTT   
  
  
- GAATTAAAAG AAACCACGTA TACTTTAGAA TTTTTCTTAA TTTTATTCTA TATTATGCAA TTGAGAAGTT   
  
  
- GTTGTACTAT ATAAATTCTA TGGTTATAAA CATAAGTAGT ATATTTTTAA CATCACAACG TTTTATAATT   
  
  
- TTAATAAAGT ATTTATTTGT AATAATATTC TTGACTGAGG CGCCTAATAG TATACCATAT CTAATGTCTC   
  
  
- ACCCACCCAA ATTTATTAAA GGGGCTGTGT AATATGAAAA TCTTCTCGAT AATCGAGGTT TAATAAATTT   
  
  
- GTTACAAGAT TTCTCGTTTT TTTAGTTACA AAACCTAAAA CTAGGCTGGC TCTCCTGAAC AGGCTGGTAA   
  
  
- GGTATAATTT TACAAGGATC AAGACTAATC GATTATGGT

+     G-Box

| Site Name | Organism | Position | Strand | Matrix score. | sequence | function |
| --- | --- | --- | --- | --- | --- | --- |
| G-Box | Pisum sativum | 634 | + | 6 | CACGTT | cis-acting regulatory element involved in light responsiveness |

>PlantCARE\_4926   
+ TAAAGTTGTC GTGATTTCTT GCCCTTTATT TTTTATTTAT ATTAACATAT ATTTTTAAAA AAAAATTAAA   
  
  
+ GACGTTCCTA ACGGAAACTC ATGTATTTTT TGATTATGAA TATGAATATC CCCTCTATAT TAATCATGGA   
  
  
+ GCATTACAAC ATGTTTTCGT AGCCATATGT CATCACGAGA ATGATTTTTA GAATTGTTAG AAAAATAAAT   
  
  
+ TGATTCATAT AAACATATAC TATGTTTTTT ATTAAACTAA CTATCAAATT AATTAATAGT GTACAAAAAA   
  
  
+ ATATTTTTTT CTTTCCTTAA ATAAAAACTA CGGAATTACC TAATATGGCT AACATATATA TGACAATTAA   
  
  
+ TGATTATGAA TAATACATAT TTGATAAAAA AATTTCTAAC CTCTCTCTTT TTTGTTTAAT TTTATATTAT   
  
  
+ TAAAGGAAAT TTAACAATCA CATTAATCAT ATAATAAAAA CAATTAGATT TTTTCTTATA TGTTATATTT   
  
  
+ TGAATTTTTA AAAACGACTA TAAATTACTA AAAATGATAA GAGTCCCACA TTAAAAAATT TGTGATCAAC   
  
  
+ CGTTTAACTT TTTTTTTAGT TCAAGCAAGA TACAAATGAT CATATATCTG ATATAGACGT GGGCGTTCGG   
  
  
+ ATACACGTTC GGGTTTGTAT CAGATATTTC AGTATAAAGG TATAGAACCC GTTCGGGTAT TTCTACACTC   
  
  
+ CGAGTCGGGT TCGGGTTCGG ATATTTTGGA TCGGGTTCGA ATATTTAAAT TTTGAAGAAA AAAAGAAATT   
  
  
+ ATTCACTGTT TAAGTTTTTT ATATTTAAAT ATATCTTAAC TTAACTGATT TTTTTTAGTT TTTAAAAGAT   
  
  
+ TAAAATATTA ATATGTTTGG AGATAAAACT TTAAAAATAG AAAGACACTA ATTTAGTTTT TGTTTTGAAA   
  
  
+ ATTTAGATGC AACTTTTGTT AATGCAAGAA ACAAGAACTT GATATGTATT TTAAGTGAGT AACAAATGAT   
  
  
+ TTTGTCTATA GTTATATGTA TATTATCTAA TTTTGAGTAA TAAGAATCAT TAATATAAAT ATTTTGAATA   
  
  
+ AAATTAGATA GATAAACTAT AAATATAGAG TTAAGTATAC TTATGTTTGG TTATCTTCGA ATATTACCCG   
  
  
+ TTCGGATATA TTATCTGAAC TGGTGAAATA AGTAATATGT TTTGTTGTTT TAATTAGATA ATTTTTAGAC   
  
  
+ CGAGCTTGTG AATATATACT AGACAAACAT TTATATTTCG AGTCTGCACT TATATTCTAT AAGAGCTTGA   
  
  
+ TATATTAGAT TTGAACACTA ACCTGTTAAT ATAGTTTGCC GGTGATTTTT TTTCAAAATT TTGATTCTTA   
  
  
+ GATATGTATA TGGAGTAAAA CTAATTTTTA CAGATGCCCA TTTTTTTAAT TGACACTTAT GTAATTAACT   
  
  
+ GAATTCATAA AACAAGGTTT TTTAAAAAAA TTTAACTCAT ATCAATGAAA CAAAGACGAG AACGAAAGCA   
  
  
+ CAATTCTATG GAAATGGAAA ATGAAGTCAC TTATGGAGAT TCAATAGTAA GCAAATCGAG AGCAGAAAAT   
  
  
+ CTAATCTCCT TTCGTCATTA TACAATCAAT ATTGCCTATT TGGTTTTAGT GATTTGTTTC AGCCGCAAAA   
  
  
+ CTTAATTTTC TTTGGTGCAT ATGAAATCTT AAAAAGAATT AAAATAAGAT ATAATACGTT AACTCTTCAA   
  
  
+ CAACATGATA TATTTAAGAT ACCAATATTT GTATTCATCA TATAAAAATT GTAGTGTTGC AAAATATTAA   
  
  
+ AATTATTTCA TAAATAAACA TTATTATAAG AACTGACTCC GCGGATTATC ATATGGTATA GATTACAGAG   
  
  
+ TGGGTGGGTT TAAATAATTT CCCCGACACA TTATACTTTT AGAAGAGCTA TTAGCTCCAA ATTATTTAAA   
  
  
+ CAATGTTCTA AAGAGCAAAA AAATCAATGT TTTGGATTTT GATCCGACCG AGAGGACTTG TCCGACCATT   
  
  
+ CCATATTAAA ATGTTCCTAG TTCTGATTAG CTAATACCA  

- ATTTCAACAG CACTAAAGAA CGGGAAATAA AAAATAAATA TAATTGTATA TAAAAATTTT TTTTTAATTT   
  
  
- CTGCAAGGAT TGCCTTTGAG TACATAAAAA ACTAATACTT ATACTTATAG GGGAGATATA ATTAGTACCT   
  
  
- CGTAATGTTG TACAAAAGCA TCGGTATACA GTAGTGCTCT TACTAAAAAT CTTAACAATC TTTTTATTTA   
  
  
- ACTAAGTATA TTTGTATATG ATACAAAAAA TAATTTGATT GATAGTTTAA TTAATTATCA CATGTTTTTT   
  
  
- TATAAAAAAA GAAAGGAATT TATTTTTGAT GCCTTAATGG ATTATACCGA TTGTATATAT ACTGTTAATT   
  
  
- ACTAATACTT ATTATGTATA AACTATTTTT TTAAAGATTG GAGAGAGAAA AAACAAATTA AAATATAATA   
  
  
- ATTTCCTTTA AATTGTTAGT GTAATTAGTA TATTATTTTT GTTAATCTAA AAAAGAATAT ACAATATAAA   
  
  
- ACTTAAAAAT TTTTGCTGAT ATTTAATGAT TTTTACTATT CTCAGGGTGT AATTTTTTAA ACACTAGTTG   
  
  
- GCAAATTGAA AAAAAAATCA AGTTCGTTCT ATGTTTACTA GTATATAGAC TATATCTGCA CCCGCAAGCC   
  
  
- TATGTGCAAG CCCAAACATA GTCTATAAAG TCATATTTCC ATATCTTGGG CAAGCCCATA AAGATGTGAG   
  
  
- GCTCAGCCCA AGCCCAAGCC TATAAAACCT AGCCCAAGCT TATAAATTTA AAACTTCTTT TTTTCTTTAA   
  
  
- TAAGTGACAA ATTCAAAAAA TATAAATTTA TATAGAATTG AATTGACTAA AAAAAATCAA AAATTTTCTA   
  
  
- ATTTTATAAT TATACAAACC TCTATTTTGA AATTTTTATC TTTCTGTGAT TAAATCAAAA ACAAAACTTT   
  
  
- TAAATCTACG TTGAAAACAA TTACGTTCTT TGTTCTTGAA CTATACATAA AATTCACTCA TTGTTTACTA   
  
  
- AAACAGATAT CAATATACAT ATAATAGATT AAAACTCATT ATTCTTAGTA ATTATATTTA TAAAACTTAT   
  
  
- TTTAATCTAT CTATTTGATA TTTATATCTC AATTCATATG AATACAAACC AATAGAAGCT TATAATGGGC   
  
  
- AAGCCTATAT AATAGACTTG ACCACTTTAT TCATTATACA AAACAACAAA ATTAATCTAT TAAAAATCTG   
  
  
- GCTCGAACAC TTATATATGA TCTGTTTGTA AATATAAAGC TCAGACGTGA ATATAAGATA TTCTCGAACT   
  
  
- ATATAATCTA AACTTGTGAT TGGACAATTA TATCAAACGG CCACTAAAAA AAAGTTTTAA AACTAAGAAT   
  
  
- CTATACATAT ACCTCATTTT GATTAAAAAT GTCTACGGGT AAAAAAATTA ACTGTGAATA CATTAATTGA   
  
  
- CTTAAGTATT TTGTTCCAAA AAATTTTTTT AAATTGAGTA TAGTTACTTT GTTTCTGCTC TTGCTTTCGT   
  
  
- GTTAAGATAC CTTTACCTTT TACTTCAGTG AATACCTCTA AGTTATCATT CGTTTAGCTC TCGTCTTTTA   
  
  
- GATTAGAGGA AAGCAGTAAT ATGTTAGTTA TAACGGATAA ACCAAAATCA CTAAACAAAG TCGGCGTTTT   
  
  
- GAATTAAAAG AAACCACGTA TACTTTAGAA TTTTTCTTAA TTTTATTCTA TATTATGCAA TTGAGAAGTT   
  
  
- GTTGTACTAT ATAAATTCTA TGGTTATAAA CATAAGTAGT ATATTTTTAA CATCACAACG TTTTATAATT   
  
  
- TTAATAAAGT ATTTATTTGT AATAATATTC TTGACTGAGG CGCCTAATAG TATACCATAT CTAATGTCTC   
  
  
- ACCCACCCAA ATTTATTAAA GGGGCTGTGT AATATGAAAA TCTTCTCGAT AATCGAGGTT TAATAAATTT   
  
  
- GTTACAAGAT TTCTCGTTTT TTTAGTTACA AAACCTAAAA CTAGGCTGGC TCTCCTGAAC AGGCTGGTAA   
  
  
- GGTATAATTT TACAAGGATC AAGACTAATC GATTATGGT

+     G-box

| Site Name | Organism | Position | Strand | Matrix score. | sequence | function |
| --- | --- | --- | --- | --- | --- | --- |
| G-box | Zea mays | 8 | - | 6 | CACGAC | cis-acting regulatory element involved in light responsiveness |
| G-box | Zea mays | 616 | - | 6 | CACGTC | cis-acting regulatory element involved in light responsiveness |

>PlantCARE\_4926   
+ TAAAGTTGTC GTGATTTCTT GCCCTTTATT TTTTATTTAT ATTAACATAT ATTTTTAAAA AAAAATTAAA   
  
  
+ GACGTTCCTA ACGGAAACTC ATGTATTTTT TGATTATGAA TATGAATATC CCCTCTATAT TAATCATGGA   
  
  
+ GCATTACAAC ATGTTTTCGT AGCCATATGT CATCACGAGA ATGATTTTTA GAATTGTTAG AAAAATAAAT   
  
  
+ TGATTCATAT AAACATATAC TATGTTTTTT ATTAAACTAA CTATCAAATT AATTAATAGT GTACAAAAAA   
  
  
+ ATATTTTTTT CTTTCCTTAA ATAAAAACTA CGGAATTACC TAATATGGCT AACATATATA TGACAATTAA   
  
  
+ TGATTATGAA TAATACATAT TTGATAAAAA AATTTCTAAC CTCTCTCTTT TTTGTTTAAT TTTATATTAT   
  
  
+ TAAAGGAAAT TTAACAATCA CATTAATCAT ATAATAAAAA CAATTAGATT TTTTCTTATA TGTTATATTT   
  
  
+ TGAATTTTTA AAAACGACTA TAAATTACTA AAAATGATAA GAGTCCCACA TTAAAAAATT TGTGATCAAC   
  
  
+ CGTTTAACTT TTTTTTTAGT TCAAGCAAGA TACAAATGAT CATATATCTG ATATAGACGT GGGCGTTCGG   
  
  
+ ATACACGTTC GGGTTTGTAT CAGATATTTC AGTATAAAGG TATAGAACCC GTTCGGGTAT TTCTACACTC   
  
  
+ CGAGTCGGGT TCGGGTTCGG ATATTTTGGA TCGGGTTCGA ATATTTAAAT TTTGAAGAAA AAAAGAAATT   
  
  
+ ATTCACTGTT TAAGTTTTTT ATATTTAAAT ATATCTTAAC TTAACTGATT TTTTTTAGTT TTTAAAAGAT   
  
  
+ TAAAATATTA ATATGTTTGG AGATAAAACT TTAAAAATAG AAAGACACTA ATTTAGTTTT TGTTTTGAAA   
  
  
+ ATTTAGATGC AACTTTTGTT AATGCAAGAA ACAAGAACTT GATATGTATT TTAAGTGAGT AACAAATGAT   
  
  
+ TTTGTCTATA GTTATATGTA TATTATCTAA TTTTGAGTAA TAAGAATCAT TAATATAAAT ATTTTGAATA   
  
  
+ AAATTAGATA GATAAACTAT AAATATAGAG TTAAGTATAC TTATGTTTGG TTATCTTCGA ATATTACCCG   
  
  
+ TTCGGATATA TTATCTGAAC TGGTGAAATA AGTAATATGT TTTGTTGTTT TAATTAGATA ATTTTTAGAC   
  
  
+ CGAGCTTGTG AATATATACT AGACAAACAT TTATATTTCG AGTCTGCACT TATATTCTAT AAGAGCTTGA   
  
  
+ TATATTAGAT TTGAACACTA ACCTGTTAAT ATAGTTTGCC GGTGATTTTT TTTCAAAATT TTGATTCTTA   
  
  
+ GATATGTATA TGGAGTAAAA CTAATTTTTA CAGATGCCCA TTTTTTTAAT TGACACTTAT GTAATTAACT   
  
  
+ GAATTCATAA AACAAGGTTT TTTAAAAAAA TTTAACTCAT ATCAATGAAA CAAAGACGAG AACGAAAGCA   
  
  
+ CAATTCTATG GAAATGGAAA ATGAAGTCAC TTATGGAGAT TCAATAGTAA GCAAATCGAG AGCAGAAAAT   
  
  
+ CTAATCTCCT TTCGTCATTA TACAATCAAT ATTGCCTATT TGGTTTTAGT GATTTGTTTC AGCCGCAAAA   
  
  
+ CTTAATTTTC TTTGGTGCAT ATGAAATCTT AAAAAGAATT AAAATAAGAT ATAATACGTT AACTCTTCAA   
  
  
+ CAACATGATA TATTTAAGAT ACCAATATTT GTATTCATCA TATAAAAATT GTAGTGTTGC AAAATATTAA   
  
  
+ AATTATTTCA TAAATAAACA TTATTATAAG AACTGACTCC GCGGATTATC ATATGGTATA GATTACAGAG   
  
  
+ TGGGTGGGTT TAAATAATTT CCCCGACACA TTATACTTTT AGAAGAGCTA TTAGCTCCAA ATTATTTAAA   
  
  
+ CAATGTTCTA AAGAGCAAAA AAATCAATGT TTTGGATTTT GATCCGACCG AGAGGACTTG TCCGACCATT   
  
  
+ CCATATTAAA ATGTTCCTAG TTCTGATTAG CTAATACCA  

- ATTTCAACAG CACTAAAGAA CGGGAAATAA AAAATAAATA TAATTGTATA TAAAAATTTT TTTTTAATTT   
  
  
- CTGCAAGGAT TGCCTTTGAG TACATAAAAA ACTAATACTT ATACTTATAG GGGAGATATA ATTAGTACCT   
  
  
- CGTAATGTTG TACAAAAGCA TCGGTATACA GTAGTGCTCT TACTAAAAAT CTTAACAATC TTTTTATTTA   
  
  
- ACTAAGTATA TTTGTATATG ATACAAAAAA TAATTTGATT GATAGTTTAA TTAATTATCA CATGTTTTTT   
  
  
- TATAAAAAAA GAAAGGAATT TATTTTTGAT GCCTTAATGG ATTATACCGA TTGTATATAT ACTGTTAATT   
  
  
- ACTAATACTT ATTATGTATA AACTATTTTT TTAAAGATTG GAGAGAGAAA AAACAAATTA AAATATAATA   
  
  
- ATTTCCTTTA AATTGTTAGT GTAATTAGTA TATTATTTTT GTTAATCTAA AAAAGAATAT ACAATATAAA   
  
  
- ACTTAAAAAT TTTTGCTGAT ATTTAATGAT TTTTACTATT CTCAGGGTGT AATTTTTTAA ACACTAGTTG   
  
  
- GCAAATTGAA AAAAAAATCA AGTTCGTTCT ATGTTTACTA GTATATAGAC TATATCTGCA CCCGCAAGCC   
  
  
- TATGTGCAAG CCCAAACATA GTCTATAAAG TCATATTTCC ATATCTTGGG CAAGCCCATA AAGATGTGAG   
  
  
- GCTCAGCCCA AGCCCAAGCC TATAAAACCT AGCCCAAGCT TATAAATTTA AAACTTCTTT TTTTCTTTAA   
  
  
- TAAGTGACAA ATTCAAAAAA TATAAATTTA TATAGAATTG AATTGACTAA AAAAAATCAA AAATTTTCTA   
  
  
- ATTTTATAAT TATACAAACC TCTATTTTGA AATTTTTATC TTTCTGTGAT TAAATCAAAA ACAAAACTTT   
  
  
- TAAATCTACG TTGAAAACAA TTACGTTCTT TGTTCTTGAA CTATACATAA AATTCACTCA TTGTTTACTA   
  
  
- AAACAGATAT CAATATACAT ATAATAGATT AAAACTCATT ATTCTTAGTA ATTATATTTA TAAAACTTAT   
  
  
- TTTAATCTAT CTATTTGATA TTTATATCTC AATTCATATG AATACAAACC AATAGAAGCT TATAATGGGC   
  
  
- AAGCCTATAT AATAGACTTG ACCACTTTAT TCATTATACA AAACAACAAA ATTAATCTAT TAAAAATCTG   
  
  
- GCTCGAACAC TTATATATGA TCTGTTTGTA AATATAAAGC TCAGACGTGA ATATAAGATA TTCTCGAACT   
  
  
- ATATAATCTA AACTTGTGAT TGGACAATTA TATCAAACGG CCACTAAAAA AAAGTTTTAA AACTAAGAAT   
  
  
- CTATACATAT ACCTCATTTT GATTAAAAAT GTCTACGGGT AAAAAAATTA ACTGTGAATA CATTAATTGA   
  
  
- CTTAAGTATT TTGTTCCAAA AAATTTTTTT AAATTGAGTA TAGTTACTTT GTTTCTGCTC TTGCTTTCGT   
  
  
- GTTAAGATAC CTTTACCTTT TACTTCAGTG AATACCTCTA AGTTATCATT CGTTTAGCTC TCGTCTTTTA   
  
  
- GATTAGAGGA AAGCAGTAAT ATGTTAGTTA TAACGGATAA ACCAAAATCA CTAAACAAAG TCGGCGTTTT   
  
  
- GAATTAAAAG AAACCACGTA TACTTTAGAA TTTTTCTTAA TTTTATTCTA TATTATGCAA TTGAGAAGTT   
  
  
- GTTGTACTAT ATAAATTCTA TGGTTATAAA CATAAGTAGT ATATTTTTAA CATCACAACG TTTTATAATT   
  
  
- TTAATAAAGT ATTTATTTGT AATAATATTC TTGACTGAGG CGCCTAATAG TATACCATAT CTAATGTCTC   
  
  
- ACCCACCCAA ATTTATTAAA GGGGCTGTGT AATATGAAAA TCTTCTCGAT AATCGAGGTT TAATAAATTT   
  
  
- GTTACAAGAT TTCTCGTTTT TTTAGTTACA AAACCTAAAA CTAGGCTGGC TCTCCTGAAC AGGCTGGTAA   
  
  
- GGTATAATTT TACAAGGATC AAGACTAATC GATTATGGT

+     GA-motif

| Site Name | Organism | Position | Strand | Matrix score. | sequence | function |
| --- | --- | --- | --- | --- | --- | --- |
| GA-motif | Arabidopsis thaliana | 1058 | + | 8 | ATAGATAA | part of a light responsive element |

>PlantCARE\_4926   
+ TAAAGTTGTC GTGATTTCTT GCCCTTTATT TTTTATTTAT ATTAACATAT ATTTTTAAAA AAAAATTAAA   
  
  
+ GACGTTCCTA ACGGAAACTC ATGTATTTTT TGATTATGAA TATGAATATC CCCTCTATAT TAATCATGGA   
  
  
+ GCATTACAAC ATGTTTTCGT AGCCATATGT CATCACGAGA ATGATTTTTA GAATTGTTAG AAAAATAAAT   
  
  
+ TGATTCATAT AAACATATAC TATGTTTTTT ATTAAACTAA CTATCAAATT AATTAATAGT GTACAAAAAA   
  
  
+ ATATTTTTTT CTTTCCTTAA ATAAAAACTA CGGAATTACC TAATATGGCT AACATATATA TGACAATTAA   
  
  
+ TGATTATGAA TAATACATAT TTGATAAAAA AATTTCTAAC CTCTCTCTTT TTTGTTTAAT TTTATATTAT   
  
  
+ TAAAGGAAAT TTAACAATCA CATTAATCAT ATAATAAAAA CAATTAGATT TTTTCTTATA TGTTATATTT   
  
  
+ TGAATTTTTA AAAACGACTA TAAATTACTA AAAATGATAA GAGTCCCACA TTAAAAAATT TGTGATCAAC   
  
  
+ CGTTTAACTT TTTTTTTAGT TCAAGCAAGA TACAAATGAT CATATATCTG ATATAGACGT GGGCGTTCGG   
  
  
+ ATACACGTTC GGGTTTGTAT CAGATATTTC AGTATAAAGG TATAGAACCC GTTCGGGTAT TTCTACACTC   
  
  
+ CGAGTCGGGT TCGGGTTCGG ATATTTTGGA TCGGGTTCGA ATATTTAAAT TTTGAAGAAA AAAAGAAATT   
  
  
+ ATTCACTGTT TAAGTTTTTT ATATTTAAAT ATATCTTAAC TTAACTGATT TTTTTTAGTT TTTAAAAGAT   
  
  
+ TAAAATATTA ATATGTTTGG AGATAAAACT TTAAAAATAG AAAGACACTA ATTTAGTTTT TGTTTTGAAA   
  
  
+ ATTTAGATGC AACTTTTGTT AATGCAAGAA ACAAGAACTT GATATGTATT TTAAGTGAGT AACAAATGAT   
  
  
+ TTTGTCTATA GTTATATGTA TATTATCTAA TTTTGAGTAA TAAGAATCAT TAATATAAAT ATTTTGAATA   
  
  
+ AAATTAGATA GATAAACTAT AAATATAGAG TTAAGTATAC TTATGTTTGG TTATCTTCGA ATATTACCCG   
  
  
+ TTCGGATATA TTATCTGAAC TGGTGAAATA AGTAATATGT TTTGTTGTTT TAATTAGATA ATTTTTAGAC   
  
  
+ CGAGCTTGTG AATATATACT AGACAAACAT TTATATTTCG AGTCTGCACT TATATTCTAT AAGAGCTTGA   
  
  
+ TATATTAGAT TTGAACACTA ACCTGTTAAT ATAGTTTGCC GGTGATTTTT TTTCAAAATT TTGATTCTTA   
  
  
+ GATATGTATA TGGAGTAAAA CTAATTTTTA CAGATGCCCA TTTTTTTAAT TGACACTTAT GTAATTAACT   
  
  
+ GAATTCATAA AACAAGGTTT TTTAAAAAAA TTTAACTCAT ATCAATGAAA CAAAGACGAG AACGAAAGCA   
  
  
+ CAATTCTATG GAAATGGAAA ATGAAGTCAC TTATGGAGAT TCAATAGTAA GCAAATCGAG AGCAGAAAAT   
  
  
+ CTAATCTCCT TTCGTCATTA TACAATCAAT ATTGCCTATT TGGTTTTAGT GATTTGTTTC AGCCGCAAAA   
  
  
+ CTTAATTTTC TTTGGTGCAT ATGAAATCTT AAAAAGAATT AAAATAAGAT ATAATACGTT AACTCTTCAA   
  
  
+ CAACATGATA TATTTAAGAT ACCAATATTT GTATTCATCA TATAAAAATT GTAGTGTTGC AAAATATTAA   
  
  
+ AATTATTTCA TAAATAAACA TTATTATAAG AACTGACTCC GCGGATTATC ATATGGTATA GATTACAGAG   
  
  
+ TGGGTGGGTT TAAATAATTT CCCCGACACA TTATACTTTT AGAAGAGCTA TTAGCTCCAA ATTATTTAAA   
  
  
+ CAATGTTCTA AAGAGCAAAA AAATCAATGT TTTGGATTTT GATCCGACCG AGAGGACTTG TCCGACCATT   
  
  
+ CCATATTAAA ATGTTCCTAG TTCTGATTAG CTAATACCA  

- ATTTCAACAG CACTAAAGAA CGGGAAATAA AAAATAAATA TAATTGTATA TAAAAATTTT TTTTTAATTT   
  
  
- CTGCAAGGAT TGCCTTTGAG TACATAAAAA ACTAATACTT ATACTTATAG GGGAGATATA ATTAGTACCT   
  
  
- CGTAATGTTG TACAAAAGCA TCGGTATACA GTAGTGCTCT TACTAAAAAT CTTAACAATC TTTTTATTTA   
  
  
- ACTAAGTATA TTTGTATATG ATACAAAAAA TAATTTGATT GATAGTTTAA TTAATTATCA CATGTTTTTT   
  
  
- TATAAAAAAA GAAAGGAATT TATTTTTGAT GCCTTAATGG ATTATACCGA TTGTATATAT ACTGTTAATT   
  
  
- ACTAATACTT ATTATGTATA AACTATTTTT TTAAAGATTG GAGAGAGAAA AAACAAATTA AAATATAATA   
  
  
- ATTTCCTTTA AATTGTTAGT GTAATTAGTA TATTATTTTT GTTAATCTAA AAAAGAATAT ACAATATAAA   
  
  
- ACTTAAAAAT TTTTGCTGAT ATTTAATGAT TTTTACTATT CTCAGGGTGT AATTTTTTAA ACACTAGTTG   
  
  
- GCAAATTGAA AAAAAAATCA AGTTCGTTCT ATGTTTACTA GTATATAGAC TATATCTGCA CCCGCAAGCC   
  
  
- TATGTGCAAG CCCAAACATA GTCTATAAAG TCATATTTCC ATATCTTGGG CAAGCCCATA AAGATGTGAG   
  
  
- GCTCAGCCCA AGCCCAAGCC TATAAAACCT AGCCCAAGCT TATAAATTTA AAACTTCTTT TTTTCTTTAA   
  
  
- TAAGTGACAA ATTCAAAAAA TATAAATTTA TATAGAATTG AATTGACTAA AAAAAATCAA AAATTTTCTA   
  
  
- ATTTTATAAT TATACAAACC TCTATTTTGA AATTTTTATC TTTCTGTGAT TAAATCAAAA ACAAAACTTT   
  
  
- TAAATCTACG TTGAAAACAA TTACGTTCTT TGTTCTTGAA CTATACATAA AATTCACTCA TTGTTTACTA   
  
  
- AAACAGATAT CAATATACAT ATAATAGATT AAAACTCATT ATTCTTAGTA ATTATATTTA TAAAACTTAT   
  
  
- TTTAATCTAT CTATTTGATA TTTATATCTC AATTCATATG AATACAAACC AATAGAAGCT TATAATGGGC   
  
  
- AAGCCTATAT AATAGACTTG ACCACTTTAT TCATTATACA AAACAACAAA ATTAATCTAT TAAAAATCTG   
  
  
- GCTCGAACAC TTATATATGA TCTGTTTGTA AATATAAAGC TCAGACGTGA ATATAAGATA TTCTCGAACT   
  
  
- ATATAATCTA AACTTGTGAT TGGACAATTA TATCAAACGG CCACTAAAAA AAAGTTTTAA AACTAAGAAT   
  
  
- CTATACATAT ACCTCATTTT GATTAAAAAT GTCTACGGGT AAAAAAATTA ACTGTGAATA CATTAATTGA   
  
  
- CTTAAGTATT TTGTTCCAAA AAATTTTTTT AAATTGAGTA TAGTTACTTT GTTTCTGCTC TTGCTTTCGT   
  
  
- GTTAAGATAC CTTTACCTTT TACTTCAGTG AATACCTCTA AGTTATCATT CGTTTAGCTC TCGTCTTTTA   
  
  
- GATTAGAGGA AAGCAGTAAT ATGTTAGTTA TAACGGATAA ACCAAAATCA CTAAACAAAG TCGGCGTTTT   
  
  
- GAATTAAAAG AAACCACGTA TACTTTAGAA TTTTTCTTAA TTTTATTCTA TATTATGCAA TTGAGAAGTT   
  
  
- GTTGTACTAT ATAAATTCTA TGGTTATAAA CATAAGTAGT ATATTTTTAA CATCACAACG TTTTATAATT   
  
  
- TTAATAAAGT ATTTATTTGT AATAATATTC TTGACTGAGG CGCCTAATAG TATACCATAT CTAATGTCTC   
  
  
- ACCCACCCAA ATTTATTAAA GGGGCTGTGT AATATGAAAA TCTTCTCGAT AATCGAGGTT TAATAAATTT   
  
  
- GTTACAAGAT TTCTCGTTTT TTTAGTTACA AAACCTAAAA CTAGGCTGGC TCTCCTGAAC AGGCTGGTAA   
  
  
- GGTATAATTT TACAAGGATC AAGACTAATC GATTATGGT

+     MYB

| Site Name | Organism | Position | Strand | Matrix score. | sequence | function |
| --- | --- | --- | --- | --- | --- | --- |
| MYB | Arabidopsis thaliana | 1098 | - | 6 | TAACCA |  |

>PlantCARE\_4926   
+ TAAAGTTGTC GTGATTTCTT GCCCTTTATT TTTTATTTAT ATTAACATAT ATTTTTAAAA AAAAATTAAA   
  
  
+ GACGTTCCTA ACGGAAACTC ATGTATTTTT TGATTATGAA TATGAATATC CCCTCTATAT TAATCATGGA   
  
  
+ GCATTACAAC ATGTTTTCGT AGCCATATGT CATCACGAGA ATGATTTTTA GAATTGTTAG AAAAATAAAT   
  
  
+ TGATTCATAT AAACATATAC TATGTTTTTT ATTAAACTAA CTATCAAATT AATTAATAGT GTACAAAAAA   
  
  
+ ATATTTTTTT CTTTCCTTAA ATAAAAACTA CGGAATTACC TAATATGGCT AACATATATA TGACAATTAA   
  
  
+ TGATTATGAA TAATACATAT TTGATAAAAA AATTTCTAAC CTCTCTCTTT TTTGTTTAAT TTTATATTAT   
  
  
+ TAAAGGAAAT TTAACAATCA CATTAATCAT ATAATAAAAA CAATTAGATT TTTTCTTATA TGTTATATTT   
  
  
+ TGAATTTTTA AAAACGACTA TAAATTACTA AAAATGATAA GAGTCCCACA TTAAAAAATT TGTGATCAAC   
  
  
+ CGTTTAACTT TTTTTTTAGT TCAAGCAAGA TACAAATGAT CATATATCTG ATATAGACGT GGGCGTTCGG   
  
  
+ ATACACGTTC GGGTTTGTAT CAGATATTTC AGTATAAAGG TATAGAACCC GTTCGGGTAT TTCTACACTC   
  
  
+ CGAGTCGGGT TCGGGTTCGG ATATTTTGGA TCGGGTTCGA ATATTTAAAT TTTGAAGAAA AAAAGAAATT   
  
  
+ ATTCACTGTT TAAGTTTTTT ATATTTAAAT ATATCTTAAC TTAACTGATT TTTTTTAGTT TTTAAAAGAT   
  
  
+ TAAAATATTA ATATGTTTGG AGATAAAACT TTAAAAATAG AAAGACACTA ATTTAGTTTT TGTTTTGAAA   
  
  
+ ATTTAGATGC AACTTTTGTT AATGCAAGAA ACAAGAACTT GATATGTATT TTAAGTGAGT AACAAATGAT   
  
  
+ TTTGTCTATA GTTATATGTA TATTATCTAA TTTTGAGTAA TAAGAATCAT TAATATAAAT ATTTTGAATA   
  
  
+ AAATTAGATA GATAAACTAT AAATATAGAG TTAAGTATAC TTATGTTTGG TTATCTTCGA ATATTACCCG   
  
  
+ TTCGGATATA TTATCTGAAC TGGTGAAATA AGTAATATGT TTTGTTGTTT TAATTAGATA ATTTTTAGAC   
  
  
+ CGAGCTTGTG AATATATACT AGACAAACAT TTATATTTCG AGTCTGCACT TATATTCTAT AAGAGCTTGA   
  
  
+ TATATTAGAT TTGAACACTA ACCTGTTAAT ATAGTTTGCC GGTGATTTTT TTTCAAAATT TTGATTCTTA   
  
  
+ GATATGTATA TGGAGTAAAA CTAATTTTTA CAGATGCCCA TTTTTTTAAT TGACACTTAT GTAATTAACT   
  
  
+ GAATTCATAA AACAAGGTTT TTTAAAAAAA TTTAACTCAT ATCAATGAAA CAAAGACGAG AACGAAAGCA   
  
  
+ CAATTCTATG GAAATGGAAA ATGAAGTCAC TTATGGAGAT TCAATAGTAA GCAAATCGAG AGCAGAAAAT   
  
  
+ CTAATCTCCT TTCGTCATTA TACAATCAAT ATTGCCTATT TGGTTTTAGT GATTTGTTTC AGCCGCAAAA   
  
  
+ CTTAATTTTC TTTGGTGCAT ATGAAATCTT AAAAAGAATT AAAATAAGAT ATAATACGTT AACTCTTCAA   
  
  
+ CAACATGATA TATTTAAGAT ACCAATATTT GTATTCATCA TATAAAAATT GTAGTGTTGC AAAATATTAA   
  
  
+ AATTATTTCA TAAATAAACA TTATTATAAG AACTGACTCC GCGGATTATC ATATGGTATA GATTACAGAG   
  
  
+ TGGGTGGGTT TAAATAATTT CCCCGACACA TTATACTTTT AGAAGAGCTA TTAGCTCCAA ATTATTTAAA   
  
  
+ CAATGTTCTA AAGAGCAAAA AAATCAATGT TTTGGATTTT GATCCGACCG AGAGGACTTG TCCGACCATT   
  
  
+ CCATATTAAA ATGTTCCTAG TTCTGATTAG CTAATACCA  

- ATTTCAACAG CACTAAAGAA CGGGAAATAA AAAATAAATA TAATTGTATA TAAAAATTTT TTTTTAATTT   
  
  
- CTGCAAGGAT TGCCTTTGAG TACATAAAAA ACTAATACTT ATACTTATAG GGGAGATATA ATTAGTACCT   
  
  
- CGTAATGTTG TACAAAAGCA TCGGTATACA GTAGTGCTCT TACTAAAAAT CTTAACAATC TTTTTATTTA   
  
  
- ACTAAGTATA TTTGTATATG ATACAAAAAA TAATTTGATT GATAGTTTAA TTAATTATCA CATGTTTTTT   
  
  
- TATAAAAAAA GAAAGGAATT TATTTTTGAT GCCTTAATGG ATTATACCGA TTGTATATAT ACTGTTAATT   
  
  
- ACTAATACTT ATTATGTATA AACTATTTTT TTAAAGATTG GAGAGAGAAA AAACAAATTA AAATATAATA   
  
  
- ATTTCCTTTA AATTGTTAGT GTAATTAGTA TATTATTTTT GTTAATCTAA AAAAGAATAT ACAATATAAA   
  
  
- ACTTAAAAAT TTTTGCTGAT ATTTAATGAT TTTTACTATT CTCAGGGTGT AATTTTTTAA ACACTAGTTG   
  
  
- GCAAATTGAA AAAAAAATCA AGTTCGTTCT ATGTTTACTA GTATATAGAC TATATCTGCA CCCGCAAGCC   
  
  
- TATGTGCAAG CCCAAACATA GTCTATAAAG TCATATTTCC ATATCTTGGG CAAGCCCATA AAGATGTGAG   
  
  
- GCTCAGCCCA AGCCCAAGCC TATAAAACCT AGCCCAAGCT TATAAATTTA AAACTTCTTT TTTTCTTTAA   
  
  
- TAAGTGACAA ATTCAAAAAA TATAAATTTA TATAGAATTG AATTGACTAA AAAAAATCAA AAATTTTCTA   
  
  
- ATTTTATAAT TATACAAACC TCTATTTTGA AATTTTTATC TTTCTGTGAT TAAATCAAAA ACAAAACTTT   
  
  
- TAAATCTACG TTGAAAACAA TTACGTTCTT TGTTCTTGAA CTATACATAA AATTCACTCA TTGTTTACTA   
  
  
- AAACAGATAT CAATATACAT ATAATAGATT AAAACTCATT ATTCTTAGTA ATTATATTTA TAAAACTTAT   
  
  
- TTTAATCTAT CTATTTGATA TTTATATCTC AATTCATATG AATACAAACC AATAGAAGCT TATAATGGGC   
  
  
- AAGCCTATAT AATAGACTTG ACCACTTTAT TCATTATACA AAACAACAAA ATTAATCTAT TAAAAATCTG   
  
  
- GCTCGAACAC TTATATATGA TCTGTTTGTA AATATAAAGC TCAGACGTGA ATATAAGATA TTCTCGAACT   
  
  
- ATATAATCTA AACTTGTGAT TGGACAATTA TATCAAACGG CCACTAAAAA AAAGTTTTAA AACTAAGAAT   
  
  
- CTATACATAT ACCTCATTTT GATTAAAAAT GTCTACGGGT AAAAAAATTA ACTGTGAATA CATTAATTGA   
  
  
- CTTAAGTATT TTGTTCCAAA AAATTTTTTT AAATTGAGTA TAGTTACTTT GTTTCTGCTC TTGCTTTCGT   
  
  
- GTTAAGATAC CTTTACCTTT TACTTCAGTG AATACCTCTA AGTTATCATT CGTTTAGCTC TCGTCTTTTA   
  
  
- GATTAGAGGA AAGCAGTAAT ATGTTAGTTA TAACGGATAA ACCAAAATCA CTAAACAAAG TCGGCGTTTT   
  
  
- GAATTAAAAG AAACCACGTA TACTTTAGAA TTTTTCTTAA TTTTATTCTA TATTATGCAA TTGAGAAGTT   
  
  
- GTTGTACTAT ATAAATTCTA TGGTTATAAA CATAAGTAGT ATATTTTTAA CATCACAACG TTTTATAATT   
  
  
- TTAATAAAGT ATTTATTTGT AATAATATTC TTGACTGAGG CGCCTAATAG TATACCATAT CTAATGTCTC   
  
  
- ACCCACCCAA ATTTATTAAA GGGGCTGTGT AATATGAAAA TCTTCTCGAT AATCGAGGTT TAATAAATTT   
  
  
- GTTACAAGAT TTCTCGTTTT TTTAGTTACA AAACCTAAAA CTAGGCTGGC TCTCCTGAAC AGGCTGGTAA   
  
  
- GGTATAATTT TACAAGGATC AAGACTAATC GATTATGGT

+     MYB-like sequence

| Site Name | Organism | Position | Strand | Matrix score. | sequence | function |
| --- | --- | --- | --- | --- | --- | --- |
| MYB-like sequence | Arabidopsis thaliana | 1098 | - | 6 | TAACCA |  |

>PlantCARE\_4926   
+ TAAAGTTGTC GTGATTTCTT GCCCTTTATT TTTTATTTAT ATTAACATAT ATTTTTAAAA AAAAATTAAA   
  
  
+ GACGTTCCTA ACGGAAACTC ATGTATTTTT TGATTATGAA TATGAATATC CCCTCTATAT TAATCATGGA   
  
  
+ GCATTACAAC ATGTTTTCGT AGCCATATGT CATCACGAGA ATGATTTTTA GAATTGTTAG AAAAATAAAT   
  
  
+ TGATTCATAT AAACATATAC TATGTTTTTT ATTAAACTAA CTATCAAATT AATTAATAGT GTACAAAAAA   
  
  
+ ATATTTTTTT CTTTCCTTAA ATAAAAACTA CGGAATTACC TAATATGGCT AACATATATA TGACAATTAA   
  
  
+ TGATTATGAA TAATACATAT TTGATAAAAA AATTTCTAAC CTCTCTCTTT TTTGTTTAAT TTTATATTAT   
  
  
+ TAAAGGAAAT TTAACAATCA CATTAATCAT ATAATAAAAA CAATTAGATT TTTTCTTATA TGTTATATTT   
  
  
+ TGAATTTTTA AAAACGACTA TAAATTACTA AAAATGATAA GAGTCCCACA TTAAAAAATT TGTGATCAAC   
  
  
+ CGTTTAACTT TTTTTTTAGT TCAAGCAAGA TACAAATGAT CATATATCTG ATATAGACGT GGGCGTTCGG   
  
  
+ ATACACGTTC GGGTTTGTAT CAGATATTTC AGTATAAAGG TATAGAACCC GTTCGGGTAT TTCTACACTC   
  
  
+ CGAGTCGGGT TCGGGTTCGG ATATTTTGGA TCGGGTTCGA ATATTTAAAT TTTGAAGAAA AAAAGAAATT   
  
  
+ ATTCACTGTT TAAGTTTTTT ATATTTAAAT ATATCTTAAC TTAACTGATT TTTTTTAGTT TTTAAAAGAT   
  
  
+ TAAAATATTA ATATGTTTGG AGATAAAACT TTAAAAATAG AAAGACACTA ATTTAGTTTT TGTTTTGAAA   
  
  
+ ATTTAGATGC AACTTTTGTT AATGCAAGAA ACAAGAACTT GATATGTATT TTAAGTGAGT AACAAATGAT   
  
  
+ TTTGTCTATA GTTATATGTA TATTATCTAA TTTTGAGTAA TAAGAATCAT TAATATAAAT ATTTTGAATA   
  
  
+ AAATTAGATA GATAAACTAT AAATATAGAG TTAAGTATAC TTATGTTTGG TTATCTTCGA ATATTACCCG   
  
  
+ TTCGGATATA TTATCTGAAC TGGTGAAATA AGTAATATGT TTTGTTGTTT TAATTAGATA ATTTTTAGAC   
  
  
+ CGAGCTTGTG AATATATACT AGACAAACAT TTATATTTCG AGTCTGCACT TATATTCTAT AAGAGCTTGA   
  
  
+ TATATTAGAT TTGAACACTA ACCTGTTAAT ATAGTTTGCC GGTGATTTTT TTTCAAAATT TTGATTCTTA   
  
  
+ GATATGTATA TGGAGTAAAA CTAATTTTTA CAGATGCCCA TTTTTTTAAT TGACACTTAT GTAATTAACT   
  
  
+ GAATTCATAA AACAAGGTTT TTTAAAAAAA TTTAACTCAT ATCAATGAAA CAAAGACGAG AACGAAAGCA   
  
  
+ CAATTCTATG GAAATGGAAA ATGAAGTCAC TTATGGAGAT TCAATAGTAA GCAAATCGAG AGCAGAAAAT   
  
  
+ CTAATCTCCT TTCGTCATTA TACAATCAAT ATTGCCTATT TGGTTTTAGT GATTTGTTTC AGCCGCAAAA   
  
  
+ CTTAATTTTC TTTGGTGCAT ATGAAATCTT AAAAAGAATT AAAATAAGAT ATAATACGTT AACTCTTCAA   
  
  
+ CAACATGATA TATTTAAGAT ACCAATATTT GTATTCATCA TATAAAAATT GTAGTGTTGC AAAATATTAA   
  
  
+ AATTATTTCA TAAATAAACA TTATTATAAG AACTGACTCC GCGGATTATC ATATGGTATA GATTACAGAG   
  
  
+ TGGGTGGGTT TAAATAATTT CCCCGACACA TTATACTTTT AGAAGAGCTA TTAGCTCCAA ATTATTTAAA   
  
  
+ CAATGTTCTA AAGAGCAAAA AAATCAATGT TTTGGATTTT GATCCGACCG AGAGGACTTG TCCGACCATT   
  
  
+ CCATATTAAA ATGTTCCTAG TTCTGATTAG CTAATACCA  

- ATTTCAACAG CACTAAAGAA CGGGAAATAA AAAATAAATA TAATTGTATA TAAAAATTTT TTTTTAATTT   
  
  
- CTGCAAGGAT TGCCTTTGAG TACATAAAAA ACTAATACTT ATACTTATAG GGGAGATATA ATTAGTACCT   
  
  
- CGTAATGTTG TACAAAAGCA TCGGTATACA GTAGTGCTCT TACTAAAAAT CTTAACAATC TTTTTATTTA   
  
  
- ACTAAGTATA TTTGTATATG ATACAAAAAA TAATTTGATT GATAGTTTAA TTAATTATCA CATGTTTTTT   
  
  
- TATAAAAAAA GAAAGGAATT TATTTTTGAT GCCTTAATGG ATTATACCGA TTGTATATAT ACTGTTAATT   
  
  
- ACTAATACTT ATTATGTATA AACTATTTTT TTAAAGATTG GAGAGAGAAA AAACAAATTA AAATATAATA   
  
  
- ATTTCCTTTA AATTGTTAGT GTAATTAGTA TATTATTTTT GTTAATCTAA AAAAGAATAT ACAATATAAA   
  
  
- ACTTAAAAAT TTTTGCTGAT ATTTAATGAT TTTTACTATT CTCAGGGTGT AATTTTTTAA ACACTAGTTG   
  
  
- GCAAATTGAA AAAAAAATCA AGTTCGTTCT ATGTTTACTA GTATATAGAC TATATCTGCA CCCGCAAGCC   
  
  
- TATGTGCAAG CCCAAACATA GTCTATAAAG TCATATTTCC ATATCTTGGG CAAGCCCATA AAGATGTGAG   
  
  
- GCTCAGCCCA AGCCCAAGCC TATAAAACCT AGCCCAAGCT TATAAATTTA AAACTTCTTT TTTTCTTTAA   
  
  
- TAAGTGACAA ATTCAAAAAA TATAAATTTA TATAGAATTG AATTGACTAA AAAAAATCAA AAATTTTCTA   
  
  
- ATTTTATAAT TATACAAACC TCTATTTTGA AATTTTTATC TTTCTGTGAT TAAATCAAAA ACAAAACTTT   
  
  
- TAAATCTACG TTGAAAACAA TTACGTTCTT TGTTCTTGAA CTATACATAA AATTCACTCA TTGTTTACTA   
  
  
- AAACAGATAT CAATATACAT ATAATAGATT AAAACTCATT ATTCTTAGTA ATTATATTTA TAAAACTTAT   
  
  
- TTTAATCTAT CTATTTGATA TTTATATCTC AATTCATATG AATACAAACC AATAGAAGCT TATAATGGGC   
  
  
- AAGCCTATAT AATAGACTTG ACCACTTTAT TCATTATACA AAACAACAAA ATTAATCTAT TAAAAATCTG   
  
  
- GCTCGAACAC TTATATATGA TCTGTTTGTA AATATAAAGC TCAGACGTGA ATATAAGATA TTCTCGAACT   
  
  
- ATATAATCTA AACTTGTGAT TGGACAATTA TATCAAACGG CCACTAAAAA AAAGTTTTAA AACTAAGAAT   
  
  
- CTATACATAT ACCTCATTTT GATTAAAAAT GTCTACGGGT AAAAAAATTA ACTGTGAATA CATTAATTGA   
  
  
- CTTAAGTATT TTGTTCCAAA AAATTTTTTT AAATTGAGTA TAGTTACTTT GTTTCTGCTC TTGCTTTCGT   
  
  
- GTTAAGATAC CTTTACCTTT TACTTCAGTG AATACCTCTA AGTTATCATT CGTTTAGCTC TCGTCTTTTA   
  
  
- GATTAGAGGA AAGCAGTAAT ATGTTAGTTA TAACGGATAA ACCAAAATCA CTAAACAAAG TCGGCGTTTT   
  
  
- GAATTAAAAG AAACCACGTA TACTTTAGAA TTTTTCTTAA TTTTATTCTA TATTATGCAA TTGAGAAGTT   
  
  
- GTTGTACTAT ATAAATTCTA TGGTTATAAA CATAAGTAGT ATATTTTTAA CATCACAACG TTTTATAATT   
  
  
- TTAATAAAGT ATTTATTTGT AATAATATTC TTGACTGAGG CGCCTAATAG TATACCATAT CTAATGTCTC   
  
  
- ACCCACCCAA ATTTATTAAA GGGGCTGTGT AATATGAAAA TCTTCTCGAT AATCGAGGTT TAATAAATTT   
  
  
- GTTACAAGAT TTCTCGTTTT TTTAGTTACA AAACCTAAAA CTAGGCTGGC TCTCCTGAAC AGGCTGGTAA   
  
  
- GGTATAATTT TACAAGGATC AAGACTAATC GATTATGGT

+     MYC

| Site Name | Organism | Position | Strand | Matrix score. | sequence | function |
| --- | --- | --- | --- | --- | --- | --- |
| MYC | Arabidopsis thaliana | 593 | - | 6 | CATTTG |  |
| MYC | Arabidopsis thaliana | 973 | - | 6 | CATTTG |  |

>PlantCARE\_4926   
+ TAAAGTTGTC GTGATTTCTT GCCCTTTATT TTTTATTTAT ATTAACATAT ATTTTTAAAA AAAAATTAAA   
  
  
+ GACGTTCCTA ACGGAAACTC ATGTATTTTT TGATTATGAA TATGAATATC CCCTCTATAT TAATCATGGA   
  
  
+ GCATTACAAC ATGTTTTCGT AGCCATATGT CATCACGAGA ATGATTTTTA GAATTGTTAG AAAAATAAAT   
  
  
+ TGATTCATAT AAACATATAC TATGTTTTTT ATTAAACTAA CTATCAAATT AATTAATAGT GTACAAAAAA   
  
  
+ ATATTTTTTT CTTTCCTTAA ATAAAAACTA CGGAATTACC TAATATGGCT AACATATATA TGACAATTAA   
  
  
+ TGATTATGAA TAATACATAT TTGATAAAAA AATTTCTAAC CTCTCTCTTT TTTGTTTAAT TTTATATTAT   
  
  
+ TAAAGGAAAT TTAACAATCA CATTAATCAT ATAATAAAAA CAATTAGATT TTTTCTTATA TGTTATATTT   
  
  
+ TGAATTTTTA AAAACGACTA TAAATTACTA AAAATGATAA GAGTCCCACA TTAAAAAATT TGTGATCAAC   
  
  
+ CGTTTAACTT TTTTTTTAGT TCAAGCAAGA TACAAATGAT CATATATCTG ATATAGACGT GGGCGTTCGG   
  
  
+ ATACACGTTC GGGTTTGTAT CAGATATTTC AGTATAAAGG TATAGAACCC GTTCGGGTAT TTCTACACTC   
  
  
+ CGAGTCGGGT TCGGGTTCGG ATATTTTGGA TCGGGTTCGA ATATTTAAAT TTTGAAGAAA AAAAGAAATT   
  
  
+ ATTCACTGTT TAAGTTTTTT ATATTTAAAT ATATCTTAAC TTAACTGATT TTTTTTAGTT TTTAAAAGAT   
  
  
+ TAAAATATTA ATATGTTTGG AGATAAAACT TTAAAAATAG AAAGACACTA ATTTAGTTTT TGTTTTGAAA   
  
  
+ ATTTAGATGC AACTTTTGTT AATGCAAGAA ACAAGAACTT GATATGTATT TTAAGTGAGT AACAAATGAT   
  
  
+ TTTGTCTATA GTTATATGTA TATTATCTAA TTTTGAGTAA TAAGAATCAT TAATATAAAT ATTTTGAATA   
  
  
+ AAATTAGATA GATAAACTAT AAATATAGAG TTAAGTATAC TTATGTTTGG TTATCTTCGA ATATTACCCG   
  
  
+ TTCGGATATA TTATCTGAAC TGGTGAAATA AGTAATATGT TTTGTTGTTT TAATTAGATA ATTTTTAGAC   
  
  
+ CGAGCTTGTG AATATATACT AGACAAACAT TTATATTTCG AGTCTGCACT TATATTCTAT AAGAGCTTGA   
  
  
+ TATATTAGAT TTGAACACTA ACCTGTTAAT ATAGTTTGCC GGTGATTTTT TTTCAAAATT TTGATTCTTA   
  
  
+ GATATGTATA TGGAGTAAAA CTAATTTTTA CAGATGCCCA TTTTTTTAAT TGACACTTAT GTAATTAACT   
  
  
+ GAATTCATAA AACAAGGTTT TTTAAAAAAA TTTAACTCAT ATCAATGAAA CAAAGACGAG AACGAAAGCA   
  
  
+ CAATTCTATG GAAATGGAAA ATGAAGTCAC TTATGGAGAT TCAATAGTAA GCAAATCGAG AGCAGAAAAT   
  
  
+ CTAATCTCCT TTCGTCATTA TACAATCAAT ATTGCCTATT TGGTTTTAGT GATTTGTTTC AGCCGCAAAA   
  
  
+ CTTAATTTTC TTTGGTGCAT ATGAAATCTT AAAAAGAATT AAAATAAGAT ATAATACGTT AACTCTTCAA   
  
  
+ CAACATGATA TATTTAAGAT ACCAATATTT GTATTCATCA TATAAAAATT GTAGTGTTGC AAAATATTAA   
  
  
+ AATTATTTCA TAAATAAACA TTATTATAAG AACTGACTCC GCGGATTATC ATATGGTATA GATTACAGAG   
  
  
+ TGGGTGGGTT TAAATAATTT CCCCGACACA TTATACTTTT AGAAGAGCTA TTAGCTCCAA ATTATTTAAA   
  
  
+ CAATGTTCTA AAGAGCAAAA AAATCAATGT TTTGGATTTT GATCCGACCG AGAGGACTTG TCCGACCATT   
  
  
+ CCATATTAAA ATGTTCCTAG TTCTGATTAG CTAATACCA  

- ATTTCAACAG CACTAAAGAA CGGGAAATAA AAAATAAATA TAATTGTATA TAAAAATTTT TTTTTAATTT   
  
  
- CTGCAAGGAT TGCCTTTGAG TACATAAAAA ACTAATACTT ATACTTATAG GGGAGATATA ATTAGTACCT   
  
  
- CGTAATGTTG TACAAAAGCA TCGGTATACA GTAGTGCTCT TACTAAAAAT CTTAACAATC TTTTTATTTA   
  
  
- ACTAAGTATA TTTGTATATG ATACAAAAAA TAATTTGATT GATAGTTTAA TTAATTATCA CATGTTTTTT   
  
  
- TATAAAAAAA GAAAGGAATT TATTTTTGAT GCCTTAATGG ATTATACCGA TTGTATATAT ACTGTTAATT   
  
  
- ACTAATACTT ATTATGTATA AACTATTTTT TTAAAGATTG GAGAGAGAAA AAACAAATTA AAATATAATA   
  
  
- ATTTCCTTTA AATTGTTAGT GTAATTAGTA TATTATTTTT GTTAATCTAA AAAAGAATAT ACAATATAAA   
  
  
- ACTTAAAAAT TTTTGCTGAT ATTTAATGAT TTTTACTATT CTCAGGGTGT AATTTTTTAA ACACTAGTTG   
  
  
- GCAAATTGAA AAAAAAATCA AGTTCGTTCT ATGTTTACTA GTATATAGAC TATATCTGCA CCCGCAAGCC   
  
  
- TATGTGCAAG CCCAAACATA GTCTATAAAG TCATATTTCC ATATCTTGGG CAAGCCCATA AAGATGTGAG   
  
  
- GCTCAGCCCA AGCCCAAGCC TATAAAACCT AGCCCAAGCT TATAAATTTA AAACTTCTTT TTTTCTTTAA   
  
  
- TAAGTGACAA ATTCAAAAAA TATAAATTTA TATAGAATTG AATTGACTAA AAAAAATCAA AAATTTTCTA   
  
  
- ATTTTATAAT TATACAAACC TCTATTTTGA AATTTTTATC TTTCTGTGAT TAAATCAAAA ACAAAACTTT   
  
  
- TAAATCTACG TTGAAAACAA TTACGTTCTT TGTTCTTGAA CTATACATAA AATTCACTCA TTGTTTACTA   
  
  
- AAACAGATAT CAATATACAT ATAATAGATT AAAACTCATT ATTCTTAGTA ATTATATTTA TAAAACTTAT   
  
  
- TTTAATCTAT CTATTTGATA TTTATATCTC AATTCATATG AATACAAACC AATAGAAGCT TATAATGGGC   
  
  
- AAGCCTATAT AATAGACTTG ACCACTTTAT TCATTATACA AAACAACAAA ATTAATCTAT TAAAAATCTG   
  
  
- GCTCGAACAC TTATATATGA TCTGTTTGTA AATATAAAGC TCAGACGTGA ATATAAGATA TTCTCGAACT   
  
  
- ATATAATCTA AACTTGTGAT TGGACAATTA TATCAAACGG CCACTAAAAA AAAGTTTTAA AACTAAGAAT   
  
  
- CTATACATAT ACCTCATTTT GATTAAAAAT GTCTACGGGT AAAAAAATTA ACTGTGAATA CATTAATTGA   
  
  
- CTTAAGTATT TTGTTCCAAA AAATTTTTTT AAATTGAGTA TAGTTACTTT GTTTCTGCTC TTGCTTTCGT   
  
  
- GTTAAGATAC CTTTACCTTT TACTTCAGTG AATACCTCTA AGTTATCATT CGTTTAGCTC TCGTCTTTTA   
  
  
- GATTAGAGGA AAGCAGTAAT ATGTTAGTTA TAACGGATAA ACCAAAATCA CTAAACAAAG TCGGCGTTTT   
  
  
- GAATTAAAAG AAACCACGTA TACTTTAGAA TTTTTCTTAA TTTTATTCTA TATTATGCAA TTGAGAAGTT   
  
  
- GTTGTACTAT ATAAATTCTA TGGTTATAAA CATAAGTAGT ATATTTTTAA CATCACAACG TTTTATAATT   
  
  
- TTAATAAAGT ATTTATTTGT AATAATATTC TTGACTGAGG CGCCTAATAG TATACCATAT CTAATGTCTC   
  
  
- ACCCACCCAA ATTTATTAAA GGGGCTGTGT AATATGAAAA TCTTCTCGAT AATCGAGGTT TAATAAATTT   
  
  
- GTTACAAGAT TTCTCGTTTT TTTAGTTACA AAACCTAAAA CTAGGCTGGC TCTCCTGAAC AGGCTGGTAA   
  
  
- GGTATAATTT TACAAGGATC AAGACTAATC GATTATGGT

+     Myb

| Site Name | Organism | Position | Strand | Matrix score. | sequence | function |
| --- | --- | --- | --- | --- | --- | --- |
| Myb | Arabidopsis thaliana | 812 | + | 6 | TAACTG |  |
| Myb | Arabidopsis thaliana | 1396 | + | 6 | TAACTG |  |

>PlantCARE\_4926   
+ TAAAGTTGTC GTGATTTCTT GCCCTTTATT TTTTATTTAT ATTAACATAT ATTTTTAAAA AAAAATTAAA   
  
  
+ GACGTTCCTA ACGGAAACTC ATGTATTTTT TGATTATGAA TATGAATATC CCCTCTATAT TAATCATGGA   
  
  
+ GCATTACAAC ATGTTTTCGT AGCCATATGT CATCACGAGA ATGATTTTTA GAATTGTTAG AAAAATAAAT   
  
  
+ TGATTCATAT AAACATATAC TATGTTTTTT ATTAAACTAA CTATCAAATT AATTAATAGT GTACAAAAAA   
  
  
+ ATATTTTTTT CTTTCCTTAA ATAAAAACTA CGGAATTACC TAATATGGCT AACATATATA TGACAATTAA   
  
  
+ TGATTATGAA TAATACATAT TTGATAAAAA AATTTCTAAC CTCTCTCTTT TTTGTTTAAT TTTATATTAT   
  
  
+ TAAAGGAAAT TTAACAATCA CATTAATCAT ATAATAAAAA CAATTAGATT TTTTCTTATA TGTTATATTT   
  
  
+ TGAATTTTTA AAAACGACTA TAAATTACTA AAAATGATAA GAGTCCCACA TTAAAAAATT TGTGATCAAC   
  
  
+ CGTTTAACTT TTTTTTTAGT TCAAGCAAGA TACAAATGAT CATATATCTG ATATAGACGT GGGCGTTCGG   
  
  
+ ATACACGTTC GGGTTTGTAT CAGATATTTC AGTATAAAGG TATAGAACCC GTTCGGGTAT TTCTACACTC   
  
  
+ CGAGTCGGGT TCGGGTTCGG ATATTTTGGA TCGGGTTCGA ATATTTAAAT TTTGAAGAAA AAAAGAAATT   
  
  
+ ATTCACTGTT TAAGTTTTTT ATATTTAAAT ATATCTTAAC TTAACTGATT TTTTTTAGTT TTTAAAAGAT   
  
  
+ TAAAATATTA ATATGTTTGG AGATAAAACT TTAAAAATAG AAAGACACTA ATTTAGTTTT TGTTTTGAAA   
  
  
+ ATTTAGATGC AACTTTTGTT AATGCAAGAA ACAAGAACTT GATATGTATT TTAAGTGAGT AACAAATGAT   
  
  
+ TTTGTCTATA GTTATATGTA TATTATCTAA TTTTGAGTAA TAAGAATCAT TAATATAAAT ATTTTGAATA   
  
  
+ AAATTAGATA GATAAACTAT AAATATAGAG TTAAGTATAC TTATGTTTGG TTATCTTCGA ATATTACCCG   
  
  
+ TTCGGATATA TTATCTGAAC TGGTGAAATA AGTAATATGT TTTGTTGTTT TAATTAGATA ATTTTTAGAC   
  
  
+ CGAGCTTGTG AATATATACT AGACAAACAT TTATATTTCG AGTCTGCACT TATATTCTAT AAGAGCTTGA   
  
  
+ TATATTAGAT TTGAACACTA ACCTGTTAAT ATAGTTTGCC GGTGATTTTT TTTCAAAATT TTGATTCTTA   
  
  
+ GATATGTATA TGGAGTAAAA CTAATTTTTA CAGATGCCCA TTTTTTTAAT TGACACTTAT GTAATTAACT   
  
  
+ GAATTCATAA AACAAGGTTT TTTAAAAAAA TTTAACTCAT ATCAATGAAA CAAAGACGAG AACGAAAGCA   
  
  
+ CAATTCTATG GAAATGGAAA ATGAAGTCAC TTATGGAGAT TCAATAGTAA GCAAATCGAG AGCAGAAAAT   
  
  
+ CTAATCTCCT TTCGTCATTA TACAATCAAT ATTGCCTATT TGGTTTTAGT GATTTGTTTC AGCCGCAAAA   
  
  
+ CTTAATTTTC TTTGGTGCAT ATGAAATCTT AAAAAGAATT AAAATAAGAT ATAATACGTT AACTCTTCAA   
  
  
+ CAACATGATA TATTTAAGAT ACCAATATTT GTATTCATCA TATAAAAATT GTAGTGTTGC AAAATATTAA   
  
  
+ AATTATTTCA TAAATAAACA TTATTATAAG AACTGACTCC GCGGATTATC ATATGGTATA GATTACAGAG   
  
  
+ TGGGTGGGTT TAAATAATTT CCCCGACACA TTATACTTTT AGAAGAGCTA TTAGCTCCAA ATTATTTAAA   
  
  
+ CAATGTTCTA AAGAGCAAAA AAATCAATGT TTTGGATTTT GATCCGACCG AGAGGACTTG TCCGACCATT   
  
  
+ CCATATTAAA ATGTTCCTAG TTCTGATTAG CTAATACCA  

- ATTTCAACAG CACTAAAGAA CGGGAAATAA AAAATAAATA TAATTGTATA TAAAAATTTT TTTTTAATTT   
  
  
- CTGCAAGGAT TGCCTTTGAG TACATAAAAA ACTAATACTT ATACTTATAG GGGAGATATA ATTAGTACCT   
  
  
- CGTAATGTTG TACAAAAGCA TCGGTATACA GTAGTGCTCT TACTAAAAAT CTTAACAATC TTTTTATTTA   
  
  
- ACTAAGTATA TTTGTATATG ATACAAAAAA TAATTTGATT GATAGTTTAA TTAATTATCA CATGTTTTTT   
  
  
- TATAAAAAAA GAAAGGAATT TATTTTTGAT GCCTTAATGG ATTATACCGA TTGTATATAT ACTGTTAATT   
  
  
- ACTAATACTT ATTATGTATA AACTATTTTT TTAAAGATTG GAGAGAGAAA AAACAAATTA AAATATAATA   
  
  
- ATTTCCTTTA AATTGTTAGT GTAATTAGTA TATTATTTTT GTTAATCTAA AAAAGAATAT ACAATATAAA   
  
  
- ACTTAAAAAT TTTTGCTGAT ATTTAATGAT TTTTACTATT CTCAGGGTGT AATTTTTTAA ACACTAGTTG   
  
  
- GCAAATTGAA AAAAAAATCA AGTTCGTTCT ATGTTTACTA GTATATAGAC TATATCTGCA CCCGCAAGCC   
  
  
- TATGTGCAAG CCCAAACATA GTCTATAAAG TCATATTTCC ATATCTTGGG CAAGCCCATA AAGATGTGAG   
  
  
- GCTCAGCCCA AGCCCAAGCC TATAAAACCT AGCCCAAGCT TATAAATTTA AAACTTCTTT TTTTCTTTAA   
  
  
- TAAGTGACAA ATTCAAAAAA TATAAATTTA TATAGAATTG AATTGACTAA AAAAAATCAA AAATTTTCTA   
  
  
- ATTTTATAAT TATACAAACC TCTATTTTGA AATTTTTATC TTTCTGTGAT TAAATCAAAA ACAAAACTTT   
  
  
- TAAATCTACG TTGAAAACAA TTACGTTCTT TGTTCTTGAA CTATACATAA AATTCACTCA TTGTTTACTA   
  
  
- AAACAGATAT CAATATACAT ATAATAGATT AAAACTCATT ATTCTTAGTA ATTATATTTA TAAAACTTAT   
  
  
- TTTAATCTAT CTATTTGATA TTTATATCTC AATTCATATG AATACAAACC AATAGAAGCT TATAATGGGC   
  
  
- AAGCCTATAT AATAGACTTG ACCACTTTAT TCATTATACA AAACAACAAA ATTAATCTAT TAAAAATCTG   
  
  
- GCTCGAACAC TTATATATGA TCTGTTTGTA AATATAAAGC TCAGACGTGA ATATAAGATA TTCTCGAACT   
  
  
- ATATAATCTA AACTTGTGAT TGGACAATTA TATCAAACGG CCACTAAAAA AAAGTTTTAA AACTAAGAAT   
  
  
- CTATACATAT ACCTCATTTT GATTAAAAAT GTCTACGGGT AAAAAAATTA ACTGTGAATA CATTAATTGA   
  
  
- CTTAAGTATT TTGTTCCAAA AAATTTTTTT AAATTGAGTA TAGTTACTTT GTTTCTGCTC TTGCTTTCGT   
  
  
- GTTAAGATAC CTTTACCTTT TACTTCAGTG AATACCTCTA AGTTATCATT CGTTTAGCTC TCGTCTTTTA   
  
  
- GATTAGAGGA AAGCAGTAAT ATGTTAGTTA TAACGGATAA ACCAAAATCA CTAAACAAAG TCGGCGTTTT   
  
  
- GAATTAAAAG AAACCACGTA TACTTTAGAA TTTTTCTTAA TTTTATTCTA TATTATGCAA TTGAGAAGTT   
  
  
- GTTGTACTAT ATAAATTCTA TGGTTATAAA CATAAGTAGT ATATTTTTAA CATCACAACG TTTTATAATT   
  
  
- TTAATAAAGT ATTTATTTGT AATAATATTC TTGACTGAGG CGCCTAATAG TATACCATAT CTAATGTCTC   
  
  
- ACCCACCCAA ATTTATTAAA GGGGCTGTGT AATATGAAAA TCTTCTCGAT AATCGAGGTT TAATAAATTT   
  
  
- GTTACAAGAT TTCTCGTTTT TTTAGTTACA AAACCTAAAA CTAGGCTGGC TCTCCTGAAC AGGCTGGTAA   
  
  
- GGTATAATTT TACAAGGATC AAGACTAATC GATTATGGT

+     O2-site

| Site Name | Organism | Position | Strand | Matrix score. | sequence | function |
| --- | --- | --- | --- | --- | --- | --- |
| O2-site | Zea mays | 5 | + | 9 | GTTGACGTGA | cis-acting regulatory element involved in zein metabolism regulation |

>PlantCARE\_4926   
+ TAAAGTTGTC GTGATTTCTT GCCCTTTATT TTTTATTTAT ATTAACATAT ATTTTTAAAA AAAAATTAAA   
  
  
+ GACGTTCCTA ACGGAAACTC ATGTATTTTT TGATTATGAA TATGAATATC CCCTCTATAT TAATCATGGA   
  
  
+ GCATTACAAC ATGTTTTCGT AGCCATATGT CATCACGAGA ATGATTTTTA GAATTGTTAG AAAAATAAAT   
  
  
+ TGATTCATAT AAACATATAC TATGTTTTTT ATTAAACTAA CTATCAAATT AATTAATAGT GTACAAAAAA   
  
  
+ ATATTTTTTT CTTTCCTTAA ATAAAAACTA CGGAATTACC TAATATGGCT AACATATATA TGACAATTAA   
  
  
+ TGATTATGAA TAATACATAT TTGATAAAAA AATTTCTAAC CTCTCTCTTT TTTGTTTAAT TTTATATTAT   
  
  
+ TAAAGGAAAT TTAACAATCA CATTAATCAT ATAATAAAAA CAATTAGATT TTTTCTTATA TGTTATATTT   
  
  
+ TGAATTTTTA AAAACGACTA TAAATTACTA AAAATGATAA GAGTCCCACA TTAAAAAATT TGTGATCAAC   
  
  
+ CGTTTAACTT TTTTTTTAGT TCAAGCAAGA TACAAATGAT CATATATCTG ATATAGACGT GGGCGTTCGG   
  
  
+ ATACACGTTC GGGTTTGTAT CAGATATTTC AGTATAAAGG TATAGAACCC GTTCGGGTAT TTCTACACTC   
  
  
+ CGAGTCGGGT TCGGGTTCGG ATATTTTGGA TCGGGTTCGA ATATTTAAAT TTTGAAGAAA AAAAGAAATT   
  
  
+ ATTCACTGTT TAAGTTTTTT ATATTTAAAT ATATCTTAAC TTAACTGATT TTTTTTAGTT TTTAAAAGAT   
  
  
+ TAAAATATTA ATATGTTTGG AGATAAAACT TTAAAAATAG AAAGACACTA ATTTAGTTTT TGTTTTGAAA   
  
  
+ ATTTAGATGC AACTTTTGTT AATGCAAGAA ACAAGAACTT GATATGTATT TTAAGTGAGT AACAAATGAT   
  
  
+ TTTGTCTATA GTTATATGTA TATTATCTAA TTTTGAGTAA TAAGAATCAT TAATATAAAT ATTTTGAATA   
  
  
+ AAATTAGATA GATAAACTAT AAATATAGAG TTAAGTATAC TTATGTTTGG TTATCTTCGA ATATTACCCG   
  
  
+ TTCGGATATA TTATCTGAAC TGGTGAAATA AGTAATATGT TTTGTTGTTT TAATTAGATA ATTTTTAGAC   
  
  
+ CGAGCTTGTG AATATATACT AGACAAACAT TTATATTTCG AGTCTGCACT TATATTCTAT AAGAGCTTGA   
  
  
+ TATATTAGAT TTGAACACTA ACCTGTTAAT ATAGTTTGCC GGTGATTTTT TTTCAAAATT TTGATTCTTA   
  
  
+ GATATGTATA TGGAGTAAAA CTAATTTTTA CAGATGCCCA TTTTTTTAAT TGACACTTAT GTAATTAACT   
  
  
+ GAATTCATAA AACAAGGTTT TTTAAAAAAA TTTAACTCAT ATCAATGAAA CAAAGACGAG AACGAAAGCA   
  
  
+ CAATTCTATG GAAATGGAAA ATGAAGTCAC TTATGGAGAT TCAATAGTAA GCAAATCGAG AGCAGAAAAT   
  
  
+ CTAATCTCCT TTCGTCATTA TACAATCAAT ATTGCCTATT TGGTTTTAGT GATTTGTTTC AGCCGCAAAA   
  
  
+ CTTAATTTTC TTTGGTGCAT ATGAAATCTT AAAAAGAATT AAAATAAGAT ATAATACGTT AACTCTTCAA   
  
  
+ CAACATGATA TATTTAAGAT ACCAATATTT GTATTCATCA TATAAAAATT GTAGTGTTGC AAAATATTAA   
  
  
+ AATTATTTCA TAAATAAACA TTATTATAAG AACTGACTCC GCGGATTATC ATATGGTATA GATTACAGAG   
  
  
+ TGGGTGGGTT TAAATAATTT CCCCGACACA TTATACTTTT AGAAGAGCTA TTAGCTCCAA ATTATTTAAA   
  
  
+ CAATGTTCTA AAGAGCAAAA AAATCAATGT TTTGGATTTT GATCCGACCG AGAGGACTTG TCCGACCATT   
  
  
+ CCATATTAAA ATGTTCCTAG TTCTGATTAG CTAATACCA  

- ATTTCAACAG CACTAAAGAA CGGGAAATAA AAAATAAATA TAATTGTATA TAAAAATTTT TTTTTAATTT   
  
  
- CTGCAAGGAT TGCCTTTGAG TACATAAAAA ACTAATACTT ATACTTATAG GGGAGATATA ATTAGTACCT   
  
  
- CGTAATGTTG TACAAAAGCA TCGGTATACA GTAGTGCTCT TACTAAAAAT CTTAACAATC TTTTTATTTA   
  
  
- ACTAAGTATA TTTGTATATG ATACAAAAAA TAATTTGATT GATAGTTTAA TTAATTATCA CATGTTTTTT   
  
  
- TATAAAAAAA GAAAGGAATT TATTTTTGAT GCCTTAATGG ATTATACCGA TTGTATATAT ACTGTTAATT   
  
  
- ACTAATACTT ATTATGTATA AACTATTTTT TTAAAGATTG GAGAGAGAAA AAACAAATTA AAATATAATA   
  
  
- ATTTCCTTTA AATTGTTAGT GTAATTAGTA TATTATTTTT GTTAATCTAA AAAAGAATAT ACAATATAAA   
  
  
- ACTTAAAAAT TTTTGCTGAT ATTTAATGAT TTTTACTATT CTCAGGGTGT AATTTTTTAA ACACTAGTTG   
  
  
- GCAAATTGAA AAAAAAATCA AGTTCGTTCT ATGTTTACTA GTATATAGAC TATATCTGCA CCCGCAAGCC   
  
  
- TATGTGCAAG CCCAAACATA GTCTATAAAG TCATATTTCC ATATCTTGGG CAAGCCCATA AAGATGTGAG   
  
  
- GCTCAGCCCA AGCCCAAGCC TATAAAACCT AGCCCAAGCT TATAAATTTA AAACTTCTTT TTTTCTTTAA   
  
  
- TAAGTGACAA ATTCAAAAAA TATAAATTTA TATAGAATTG AATTGACTAA AAAAAATCAA AAATTTTCTA   
  
  
- ATTTTATAAT TATACAAACC TCTATTTTGA AATTTTTATC TTTCTGTGAT TAAATCAAAA ACAAAACTTT   
  
  
- TAAATCTACG TTGAAAACAA TTACGTTCTT TGTTCTTGAA CTATACATAA AATTCACTCA TTGTTTACTA   
  
  
- AAACAGATAT CAATATACAT ATAATAGATT AAAACTCATT ATTCTTAGTA ATTATATTTA TAAAACTTAT   
  
  
- TTTAATCTAT CTATTTGATA TTTATATCTC AATTCATATG AATACAAACC AATAGAAGCT TATAATGGGC   
  
  
- AAGCCTATAT AATAGACTTG ACCACTTTAT TCATTATACA AAACAACAAA ATTAATCTAT TAAAAATCTG   
  
  
- GCTCGAACAC TTATATATGA TCTGTTTGTA AATATAAAGC TCAGACGTGA ATATAAGATA TTCTCGAACT   
  
  
- ATATAATCTA AACTTGTGAT TGGACAATTA TATCAAACGG CCACTAAAAA AAAGTTTTAA AACTAAGAAT   
  
  
- CTATACATAT ACCTCATTTT GATTAAAAAT GTCTACGGGT AAAAAAATTA ACTGTGAATA CATTAATTGA   
  
  
- CTTAAGTATT TTGTTCCAAA AAATTTTTTT AAATTGAGTA TAGTTACTTT GTTTCTGCTC TTGCTTTCGT   
  
  
- GTTAAGATAC CTTTACCTTT TACTTCAGTG AATACCTCTA AGTTATCATT CGTTTAGCTC TCGTCTTTTA   
  
  
- GATTAGAGGA AAGCAGTAAT ATGTTAGTTA TAACGGATAA ACCAAAATCA CTAAACAAAG TCGGCGTTTT   
  
  
- GAATTAAAAG AAACCACGTA TACTTTAGAA TTTTTCTTAA TTTTATTCTA TATTATGCAA TTGAGAAGTT   
  
  
- GTTGTACTAT ATAAATTCTA TGGTTATAAA CATAAGTAGT ATATTTTTAA CATCACAACG TTTTATAATT   
  
  
- TTAATAAAGT ATTTATTTGT AATAATATTC TTGACTGAGG CGCCTAATAG TATACCATAT CTAATGTCTC   
  
  
- ACCCACCCAA ATTTATTAAA GGGGCTGTGT AATATGAAAA TCTTCTCGAT AATCGAGGTT TAATAAATTT   
  
  
- GTTACAAGAT TTCTCGTTTT TTTAGTTACA AAACCTAAAA CTAGGCTGGC TCTCCTGAAC AGGCTGGTAA   
  
  
- GGTATAATTT TACAAGGATC AAGACTAATC GATTATGGT

+     STRE

| Site Name | Organism | Position | Strand | Matrix score. | sequence | function |
| --- | --- | --- | --- | --- | --- | --- |
| STRE | Arabidopsis thaliana | 120 | - | 5 | AGGGG |  |

>PlantCARE\_4926   
+ TAAAGTTGTC GTGATTTCTT GCCCTTTATT TTTTATTTAT ATTAACATAT ATTTTTAAAA AAAAATTAAA   
  
  
+ GACGTTCCTA ACGGAAACTC ATGTATTTTT TGATTATGAA TATGAATATC CCCTCTATAT TAATCATGGA   
  
  
+ GCATTACAAC ATGTTTTCGT AGCCATATGT CATCACGAGA ATGATTTTTA GAATTGTTAG AAAAATAAAT   
  
  
+ TGATTCATAT AAACATATAC TATGTTTTTT ATTAAACTAA CTATCAAATT AATTAATAGT GTACAAAAAA   
  
  
+ ATATTTTTTT CTTTCCTTAA ATAAAAACTA CGGAATTACC TAATATGGCT AACATATATA TGACAATTAA   
  
  
+ TGATTATGAA TAATACATAT TTGATAAAAA AATTTCTAAC CTCTCTCTTT TTTGTTTAAT TTTATATTAT   
  
  
+ TAAAGGAAAT TTAACAATCA CATTAATCAT ATAATAAAAA CAATTAGATT TTTTCTTATA TGTTATATTT   
  
  
+ TGAATTTTTA AAAACGACTA TAAATTACTA AAAATGATAA GAGTCCCACA TTAAAAAATT TGTGATCAAC   
  
  
+ CGTTTAACTT TTTTTTTAGT TCAAGCAAGA TACAAATGAT CATATATCTG ATATAGACGT GGGCGTTCGG   
  
  
+ ATACACGTTC GGGTTTGTAT CAGATATTTC AGTATAAAGG TATAGAACCC GTTCGGGTAT TTCTACACTC   
  
  
+ CGAGTCGGGT TCGGGTTCGG ATATTTTGGA TCGGGTTCGA ATATTTAAAT TTTGAAGAAA AAAAGAAATT   
  
  
+ ATTCACTGTT TAAGTTTTTT ATATTTAAAT ATATCTTAAC TTAACTGATT TTTTTTAGTT TTTAAAAGAT   
  
  
+ TAAAATATTA ATATGTTTGG AGATAAAACT TTAAAAATAG AAAGACACTA ATTTAGTTTT TGTTTTGAAA   
  
  
+ ATTTAGATGC AACTTTTGTT AATGCAAGAA ACAAGAACTT GATATGTATT TTAAGTGAGT AACAAATGAT   
  
  
+ TTTGTCTATA GTTATATGTA TATTATCTAA TTTTGAGTAA TAAGAATCAT TAATATAAAT ATTTTGAATA   
  
  
+ AAATTAGATA GATAAACTAT AAATATAGAG TTAAGTATAC TTATGTTTGG TTATCTTCGA ATATTACCCG   
  
  
+ TTCGGATATA TTATCTGAAC TGGTGAAATA AGTAATATGT TTTGTTGTTT TAATTAGATA ATTTTTAGAC   
  
  
+ CGAGCTTGTG AATATATACT AGACAAACAT TTATATTTCG AGTCTGCACT TATATTCTAT AAGAGCTTGA   
  
  
+ TATATTAGAT TTGAACACTA ACCTGTTAAT ATAGTTTGCC GGTGATTTTT TTTCAAAATT TTGATTCTTA   
  
  
+ GATATGTATA TGGAGTAAAA CTAATTTTTA CAGATGCCCA TTTTTTTAAT TGACACTTAT GTAATTAACT   
  
  
+ GAATTCATAA AACAAGGTTT TTTAAAAAAA TTTAACTCAT ATCAATGAAA CAAAGACGAG AACGAAAGCA   
  
  
+ CAATTCTATG GAAATGGAAA ATGAAGTCAC TTATGGAGAT TCAATAGTAA GCAAATCGAG AGCAGAAAAT   
  
  
+ CTAATCTCCT TTCGTCATTA TACAATCAAT ATTGCCTATT TGGTTTTAGT GATTTGTTTC AGCCGCAAAA   
  
  
+ CTTAATTTTC TTTGGTGCAT ATGAAATCTT AAAAAGAATT AAAATAAGAT ATAATACGTT AACTCTTCAA   
  
  
+ CAACATGATA TATTTAAGAT ACCAATATTT GTATTCATCA TATAAAAATT GTAGTGTTGC AAAATATTAA   
  
  
+ AATTATTTCA TAAATAAACA TTATTATAAG AACTGACTCC GCGGATTATC ATATGGTATA GATTACAGAG   
  
  
+ TGGGTGGGTT TAAATAATTT CCCCGACACA TTATACTTTT AGAAGAGCTA TTAGCTCCAA ATTATTTAAA   
  
  
+ CAATGTTCTA AAGAGCAAAA AAATCAATGT TTTGGATTTT GATCCGACCG AGAGGACTTG TCCGACCATT   
  
  
+ CCATATTAAA ATGTTCCTAG TTCTGATTAG CTAATACCA  

- ATTTCAACAG CACTAAAGAA CGGGAAATAA AAAATAAATA TAATTGTATA TAAAAATTTT TTTTTAATTT   
  
  
- CTGCAAGGAT TGCCTTTGAG TACATAAAAA ACTAATACTT ATACTTATAG GGGAGATATA ATTAGTACCT   
  
  
- CGTAATGTTG TACAAAAGCA TCGGTATACA GTAGTGCTCT TACTAAAAAT CTTAACAATC TTTTTATTTA   
  
  
- ACTAAGTATA TTTGTATATG ATACAAAAAA TAATTTGATT GATAGTTTAA TTAATTATCA CATGTTTTTT   
  
  
- TATAAAAAAA GAAAGGAATT TATTTTTGAT GCCTTAATGG ATTATACCGA TTGTATATAT ACTGTTAATT   
  
  
- ACTAATACTT ATTATGTATA AACTATTTTT TTAAAGATTG GAGAGAGAAA AAACAAATTA AAATATAATA   
  
  
- ATTTCCTTTA AATTGTTAGT GTAATTAGTA TATTATTTTT GTTAATCTAA AAAAGAATAT ACAATATAAA   
  
  
- ACTTAAAAAT TTTTGCTGAT ATTTAATGAT TTTTACTATT CTCAGGGTGT AATTTTTTAA ACACTAGTTG   
  
  
- GCAAATTGAA AAAAAAATCA AGTTCGTTCT ATGTTTACTA GTATATAGAC TATATCTGCA CCCGCAAGCC   
  
  
- TATGTGCAAG CCCAAACATA GTCTATAAAG TCATATTTCC ATATCTTGGG CAAGCCCATA AAGATGTGAG   
  
  
- GCTCAGCCCA AGCCCAAGCC TATAAAACCT AGCCCAAGCT TATAAATTTA AAACTTCTTT TTTTCTTTAA   
  
  
- TAAGTGACAA ATTCAAAAAA TATAAATTTA TATAGAATTG AATTGACTAA AAAAAATCAA AAATTTTCTA   
  
  
- ATTTTATAAT TATACAAACC TCTATTTTGA AATTTTTATC TTTCTGTGAT TAAATCAAAA ACAAAACTTT   
  
  
- TAAATCTACG TTGAAAACAA TTACGTTCTT TGTTCTTGAA CTATACATAA AATTCACTCA TTGTTTACTA   
  
  
- AAACAGATAT CAATATACAT ATAATAGATT AAAACTCATT ATTCTTAGTA ATTATATTTA TAAAACTTAT   
  
  
- TTTAATCTAT CTATTTGATA TTTATATCTC AATTCATATG AATACAAACC AATAGAAGCT TATAATGGGC   
  
  
- AAGCCTATAT AATAGACTTG ACCACTTTAT TCATTATACA AAACAACAAA ATTAATCTAT TAAAAATCTG   
  
  
- GCTCGAACAC TTATATATGA TCTGTTTGTA AATATAAAGC TCAGACGTGA ATATAAGATA TTCTCGAACT   
  
  
- ATATAATCTA AACTTGTGAT TGGACAATTA TATCAAACGG CCACTAAAAA AAAGTTTTAA AACTAAGAAT   
  
  
- CTATACATAT ACCTCATTTT GATTAAAAAT GTCTACGGGT AAAAAAATTA ACTGTGAATA CATTAATTGA   
  
  
- CTTAAGTATT TTGTTCCAAA AAATTTTTTT AAATTGAGTA TAGTTACTTT GTTTCTGCTC TTGCTTTCGT   
  
  
- GTTAAGATAC CTTTACCTTT TACTTCAGTG AATACCTCTA AGTTATCATT CGTTTAGCTC TCGTCTTTTA   
  
  
- GATTAGAGGA AAGCAGTAAT ATGTTAGTTA TAACGGATAA ACCAAAATCA CTAAACAAAG TCGGCGTTTT   
  
  
- GAATTAAAAG AAACCACGTA TACTTTAGAA TTTTTCTTAA TTTTATTCTA TATTATGCAA TTGAGAAGTT   
  
  
- GTTGTACTAT ATAAATTCTA TGGTTATAAA CATAAGTAGT ATATTTTTAA CATCACAACG TTTTATAATT   
  
  
- TTAATAAAGT ATTTATTTGT AATAATATTC TTGACTGAGG CGCCTAATAG TATACCATAT CTAATGTCTC   
  
  
- ACCCACCCAA ATTTATTAAA GGGGCTGTGT AATATGAAAA TCTTCTCGAT AATCGAGGTT TAATAAATTT   
  
  
- GTTACAAGAT TTCTCGTTTT TTTAGTTACA AAACCTAAAA CTAGGCTGGC TCTCCTGAAC AGGCTGGTAA   
  
  
- GGTATAATTT TACAAGGATC AAGACTAATC GATTATGGT

+     TATA

| Site Name | Organism | Position | Strand | Matrix score. | sequence | function |
| --- | --- | --- | --- | --- | --- | --- |
| TATA | Arabidopsis thaliana | 409 | - | 8 | TATAAAAT |  |

>PlantCARE\_4926   
+ TAAAGTTGTC GTGATTTCTT GCCCTTTATT TTTTATTTAT ATTAACATAT ATTTTTAAAA AAAAATTAAA   
  
  
+ GACGTTCCTA ACGGAAACTC ATGTATTTTT TGATTATGAA TATGAATATC CCCTCTATAT TAATCATGGA   
  
  
+ GCATTACAAC ATGTTTTCGT AGCCATATGT CATCACGAGA ATGATTTTTA GAATTGTTAG AAAAATAAAT   
  
  
+ TGATTCATAT AAACATATAC TATGTTTTTT ATTAAACTAA CTATCAAATT AATTAATAGT GTACAAAAAA   
  
  
+ ATATTTTTTT CTTTCCTTAA ATAAAAACTA CGGAATTACC TAATATGGCT AACATATATA TGACAATTAA   
  
  
+ TGATTATGAA TAATACATAT TTGATAAAAA AATTTCTAAC CTCTCTCTTT TTTGTTTAAT TTTATATTAT   
  
  
+ TAAAGGAAAT TTAACAATCA CATTAATCAT ATAATAAAAA CAATTAGATT TTTTCTTATA TGTTATATTT   
  
  
+ TGAATTTTTA AAAACGACTA TAAATTACTA AAAATGATAA GAGTCCCACA TTAAAAAATT TGTGATCAAC   
  
  
+ CGTTTAACTT TTTTTTTAGT TCAAGCAAGA TACAAATGAT CATATATCTG ATATAGACGT GGGCGTTCGG   
  
  
+ ATACACGTTC GGGTTTGTAT CAGATATTTC AGTATAAAGG TATAGAACCC GTTCGGGTAT TTCTACACTC   
  
  
+ CGAGTCGGGT TCGGGTTCGG ATATTTTGGA TCGGGTTCGA ATATTTAAAT TTTGAAGAAA AAAAGAAATT   
  
  
+ ATTCACTGTT TAAGTTTTTT ATATTTAAAT ATATCTTAAC TTAACTGATT TTTTTTAGTT TTTAAAAGAT   
  
  
+ TAAAATATTA ATATGTTTGG AGATAAAACT TTAAAAATAG AAAGACACTA ATTTAGTTTT TGTTTTGAAA   
  
  
+ ATTTAGATGC AACTTTTGTT AATGCAAGAA ACAAGAACTT GATATGTATT TTAAGTGAGT AACAAATGAT   
  
  
+ TTTGTCTATA GTTATATGTA TATTATCTAA TTTTGAGTAA TAAGAATCAT TAATATAAAT ATTTTGAATA   
  
  
+ AAATTAGATA GATAAACTAT AAATATAGAG TTAAGTATAC TTATGTTTGG TTATCTTCGA ATATTACCCG   
  
  
+ TTCGGATATA TTATCTGAAC TGGTGAAATA AGTAATATGT TTTGTTGTTT TAATTAGATA ATTTTTAGAC   
  
  
+ CGAGCTTGTG AATATATACT AGACAAACAT TTATATTTCG AGTCTGCACT TATATTCTAT AAGAGCTTGA   
  
  
+ TATATTAGAT TTGAACACTA ACCTGTTAAT ATAGTTTGCC GGTGATTTTT TTTCAAAATT TTGATTCTTA   
  
  
+ GATATGTATA TGGAGTAAAA CTAATTTTTA CAGATGCCCA TTTTTTTAAT TGACACTTAT GTAATTAACT   
  
  
+ GAATTCATAA AACAAGGTTT TTTAAAAAAA TTTAACTCAT ATCAATGAAA CAAAGACGAG AACGAAAGCA   
  
  
+ CAATTCTATG GAAATGGAAA ATGAAGTCAC TTATGGAGAT TCAATAGTAA GCAAATCGAG AGCAGAAAAT   
  
  
+ CTAATCTCCT TTCGTCATTA TACAATCAAT ATTGCCTATT TGGTTTTAGT GATTTGTTTC AGCCGCAAAA   
  
  
+ CTTAATTTTC TTTGGTGCAT ATGAAATCTT AAAAAGAATT AAAATAAGAT ATAATACGTT AACTCTTCAA   
  
  
+ CAACATGATA TATTTAAGAT ACCAATATTT GTATTCATCA TATAAAAATT GTAGTGTTGC AAAATATTAA   
  
  
+ AATTATTTCA TAAATAAACA TTATTATAAG AACTGACTCC GCGGATTATC ATATGGTATA GATTACAGAG   
  
  
+ TGGGTGGGTT TAAATAATTT CCCCGACACA TTATACTTTT AGAAGAGCTA TTAGCTCCAA ATTATTTAAA   
  
  
+ CAATGTTCTA AAGAGCAAAA AAATCAATGT TTTGGATTTT GATCCGACCG AGAGGACTTG TCCGACCATT   
  
  
+ CCATATTAAA ATGTTCCTAG TTCTGATTAG CTAATACCA  

- ATTTCAACAG CACTAAAGAA CGGGAAATAA AAAATAAATA TAATTGTATA TAAAAATTTT TTTTTAATTT   
  
  
- CTGCAAGGAT TGCCTTTGAG TACATAAAAA ACTAATACTT ATACTTATAG GGGAGATATA ATTAGTACCT   
  
  
- CGTAATGTTG TACAAAAGCA TCGGTATACA GTAGTGCTCT TACTAAAAAT CTTAACAATC TTTTTATTTA   
  
  
- ACTAAGTATA TTTGTATATG ATACAAAAAA TAATTTGATT GATAGTTTAA TTAATTATCA CATGTTTTTT   
  
  
- TATAAAAAAA GAAAGGAATT TATTTTTGAT GCCTTAATGG ATTATACCGA TTGTATATAT ACTGTTAATT   
  
  
- ACTAATACTT ATTATGTATA AACTATTTTT TTAAAGATTG GAGAGAGAAA AAACAAATTA AAATATAATA   
  
  
- ATTTCCTTTA AATTGTTAGT GTAATTAGTA TATTATTTTT GTTAATCTAA AAAAGAATAT ACAATATAAA   
  
  
- ACTTAAAAAT TTTTGCTGAT ATTTAATGAT TTTTACTATT CTCAGGGTGT AATTTTTTAA ACACTAGTTG   
  
  
- GCAAATTGAA AAAAAAATCA AGTTCGTTCT ATGTTTACTA GTATATAGAC TATATCTGCA CCCGCAAGCC   
  
  
- TATGTGCAAG CCCAAACATA GTCTATAAAG TCATATTTCC ATATCTTGGG CAAGCCCATA AAGATGTGAG   
  
  
- GCTCAGCCCA AGCCCAAGCC TATAAAACCT AGCCCAAGCT TATAAATTTA AAACTTCTTT TTTTCTTTAA   
  
  
- TAAGTGACAA ATTCAAAAAA TATAAATTTA TATAGAATTG AATTGACTAA AAAAAATCAA AAATTTTCTA   
  
  
- ATTTTATAAT TATACAAACC TCTATTTTGA AATTTTTATC TTTCTGTGAT TAAATCAAAA ACAAAACTTT   
  
  
- TAAATCTACG TTGAAAACAA TTACGTTCTT TGTTCTTGAA CTATACATAA AATTCACTCA TTGTTTACTA   
  
  
- AAACAGATAT CAATATACAT ATAATAGATT AAAACTCATT ATTCTTAGTA ATTATATTTA TAAAACTTAT   
  
  
- TTTAATCTAT CTATTTGATA TTTATATCTC AATTCATATG AATACAAACC AATAGAAGCT TATAATGGGC   
  
  
- AAGCCTATAT AATAGACTTG ACCACTTTAT TCATTATACA AAACAACAAA ATTAATCTAT TAAAAATCTG   
  
  
- GCTCGAACAC TTATATATGA TCTGTTTGTA AATATAAAGC TCAGACGTGA ATATAAGATA TTCTCGAACT   
  
  
- ATATAATCTA AACTTGTGAT TGGACAATTA TATCAAACGG CCACTAAAAA AAAGTTTTAA AACTAAGAAT   
  
  
- CTATACATAT ACCTCATTTT GATTAAAAAT GTCTACGGGT AAAAAAATTA ACTGTGAATA CATTAATTGA   
  
  
- CTTAAGTATT TTGTTCCAAA AAATTTTTTT AAATTGAGTA TAGTTACTTT GTTTCTGCTC TTGCTTTCGT   
  
  
- GTTAAGATAC CTTTACCTTT TACTTCAGTG AATACCTCTA AGTTATCATT CGTTTAGCTC TCGTCTTTTA   
  
  
- GATTAGAGGA AAGCAGTAAT ATGTTAGTTA TAACGGATAA ACCAAAATCA CTAAACAAAG TCGGCGTTTT   
  
  
- GAATTAAAAG AAACCACGTA TACTTTAGAA TTTTTCTTAA TTTTATTCTA TATTATGCAA TTGAGAAGTT   
  
  
- GTTGTACTAT ATAAATTCTA TGGTTATAAA CATAAGTAGT ATATTTTTAA CATCACAACG TTTTATAATT   
  
  
- TTAATAAAGT ATTTATTTGT AATAATATTC TTGACTGAGG CGCCTAATAG TATACCATAT CTAATGTCTC   
  
  
- ACCCACCCAA ATTTATTAAA GGGGCTGTGT AATATGAAAA TCTTCTCGAT AATCGAGGTT TAATAAATTT   
  
  
- GTTACAAGAT TTCTCGTTTT TTTAGTTACA AAACCTAAAA CTAGGCTGGC TCTCCTGAAC AGGCTGGTAA   
  
  
- GGTATAATTT TACAAGGATC AAGACTAATC GATTATGGT

+     TATA-box

| Site Name | Organism | Position | Strand | Matrix score. | sequence | function |
| --- | --- | --- | --- | --- | --- | --- |
| TATA-box | Arabidopsis thaliana | 1719 | + | 9 | ccTATAAAaa | core promoter element around -30 of transcription start |
| TATA-box | Arabidopsis thaliana | 1721 | - | 4 | TATA | core promoter element around -30 of transcription start |
| TATA-box | Brassica napus | 1688 | - | 6 | ATATAT | core promoter element around -30 of transcription start |
| TATA-box | Brassica napus | 1850 | + | 6 | ATTATA | core promoter element around -30 of transcription start |
| TATA-box | Arabidopsis thaliana | 484 | + | 4 | TATA | core promoter element around -30 of transcription start |
| TATA-box | Arabidopsis thaliana | 1807 | - | 4 | TATA | core promoter element around -30 of transcription start |
| TATA-box | Arabidopsis thaliana | 483 | - | 5 | TATAA | core promoter element around -30 of transcription start |
| TATA-box | Arabidopsis thaliana | 1852 | - | 4 | TATA | core promoter element around -30 of transcription start |
| TATA-box | Arabidopsis thaliana | 1689 | - | 4 | TATA | core promoter element around -30 of transcription start |
| TATA-box | Arabidopsis thaliana | 1774 | - | 5 | TATAA | core promoter element around -30 of transcription start |
| TATA-box | Daucus carota | 34 | - | 8 | TATAAATA | core promoter element around -30 of transcription start |
| TATA-box | Arabidopsis thaliana | 1851 | - | 5 | TATAA | core promoter element around -30 of transcription start |
| TATA-box | Brassica oleracea | 1720 | + | 6 | ATATAA | core promoter element around -30 of transcription start |
| TATA-box | Arabidopsis thaliana | 1829 | - | 8 | TATTTAAA | core promoter element around -30 of transcription start |
| TATA-box | Brassica juncea | 35 | - | 7 | TATAAAT | core promoter element around -30 of transcription start |
| TATA-box | Arabidopsis thaliana | 1883 | + | 8 | TATTTAAA | core promoter element around -30 of transcription start |
| TATA-box | Brassica oleracea | 1659 | + | 6 | ATATAA | core promoter element around -30 of transcription start |
| TATA-box | Brassica napus | 1773 | + | 6 | ATTATA | core promoter element around -30 of transcription start |
| TATA-box | Helianthus annuus | 36 | - | 6 | TATAAA | core promoter element around -30 of transcription start |
| TATA-box | Arabidopsis thaliana | 1775 | - | 4 | TATA | core promoter element around -30 of transcription start |
| TATA-box | Arabidopsis thaliana | 1660 | - | 4 | TATA | core promoter element around -30 of transcription start |
| TATA-box | Arabidopsis thaliana | 37 | - | 5 | TATAA | core promoter element around -30 of transcription start |
| TATA-box | Arabidopsis thaliana | 38 | + | 4 | TATA | core promoter element around -30 of transcription start |
| TATA-box | Brassica napus | 47 | + | 6 | ATATAT | core promoter element around -30 of transcription start |
| TATA-box | Arabidopsis thaliana | 48 | + | 4 | TATA | core promoter element around -30 of transcription start |
| TATA-box | Arabidopsis thaliana | 126 | + | 4 | TATA | core promoter element around -30 of transcription start |
| TATA-box | Arabidopsis thaliana | 212 | - | 9 | taTATAAAtc | core promoter element around -30 of transcription start |
| TATA-box | Brassica oleracea | 217 | + | 6 | ATATAA | core promoter element around -30 of transcription start |
| TATA-box | Arabidopsis thaliana | 218 | + | 4 | TATA | core promoter element around -30 of transcription start |
| TATA-box | Arabidopsis thaliana | 226 | + | 4 | TATA | core promoter element around -30 of transcription start |
| TATA-box | Oryza sativa | 272 | + | 7 | TACAAAA | core promoter element around -30 of transcription start |
| TATA-box | Brassica napus | 334 | + | 6 | ATATAT | core promoter element around -30 of transcription start |
| TATA-box | Arabidopsis thaliana | 335 | + | 6 | TATATA | core promoter element around -30 of transcription start |
| TATA-box | Brassica napus | 336 | + | 6 | ATATAT | core promoter element around -30 of transcription start |
| TATA-box | Arabidopsis thaliana | 337 | + | 4 | TATA | core promoter element around -30 of transcription start |
| TATA-box | Pisum sativum | 410 | - | 7 | TATAAAA | core promoter element around -30 of transcription start |
| TATA-box | Helianthus annuus | 411 | - | 6 | TATAAA | core promoter element around -30 of transcription start |
| TATA-box | Arabidopsis thaliana | 412 | - | 5 | TATAA | core promoter element around -30 of transcription start |
| TATA-box | Arabidopsis thaliana | 413 | + | 4 | TATA | core promoter element around -30 of transcription start |
| TATA-box | Brassica oleracea | 449 | + | 6 | ATATAA | core promoter element around -30 of transcription start |
| TATA-box | Arabidopsis thaliana | 450 | + | 4 | TATA | core promoter element around -30 of transcription start |
| TATA-box | Zea mays | 473 | - | 8 | TATAAGAA | core promoter element around -30 of transcription start |
| TATA-box | Arabidopsis thaliana | 476 | - | 5 | TATAA | core promoter element around -30 of transcription start |
| TATA-box | Arabidopsis thaliana | 477 | + | 4 | TATA | core promoter element around -30 of transcription start |
| TATA-box | Arabidopsis thaliana | 509 | + | 4 | TATA | core promoter element around -30 of transcription start |
| TATA-box | Brassica napus | 602 | + | 6 | ATATAT | core promoter element around -30 of transcription start |
| TATA-box | Arabidopsis thaliana | 603 | + | 4 | TATA | core promoter element around -30 of transcription start |
| TATA-box | Arabidopsis thaliana | 612 | + | 4 | TATA | core promoter element around -30 of transcription start |
| TATA-box | Arabidopsis thaliana | 663 | + | 4 | TATA | core promoter element around -30 of transcription start |
| TATA-box | Arabidopsis thaliana | 671 | + | 4 | TATA | core promoter element around -30 of transcription start |
| TATA-box | Arabidopsis thaliana | 742 | + | 8 | TATTTAAA | core promoter element around -30 of transcription start |
| TATA-box | Pisum sativum | 787 | - | 7 | TATAAAA | core promoter element around -30 of transcription start |
| TATA-box | Helianthus annuus | 788 | - | 6 | TATAAA | core promoter element around -30 of transcription start |
| TATA-box | Arabidopsis thaliana | 789 | - | 5 | TATAA | core promoter element around -30 of transcription start |
| TATA-box | Arabidopsis thaliana | 790 | + | 4 | TATA | core promoter element around -30 of transcription start |
| TATA-box | Arabidopsis thaliana | 792 | + | 8 | TATTTAAA | core promoter element around -30 of transcription start |
| TATA-box | Arabidopsis thaliana | 794 | - | 8 | TATTTAAA | core promoter element around -30 of transcription start |
| TATA-box | Brassica napus | 799 | + | 6 | ATATAT | core promoter element around -30 of transcription start |
| TATA-box | Arabidopsis thaliana | 800 | + | 4 | TATA | core promoter element around -30 of transcription start |
| TATA-box | Arabidopsis thaliana | 987 | + | 4 | TATA | core promoter element around -30 of transcription start |
| TATA-box | Arabidopsis thaliana | 992 | - | 5 | TATAA | core promoter element around -30 of transcription start |
| TATA-box | Arabidopsis thaliana | 993 | + | 4 | TATA | core promoter element around -30 of transcription start |
| TATA-box | Helianthus annuus | 997 | - | 6 | TATACA | core promoter element around -30 of transcription start |
| TATA-box | Arabidopsis thaliana | 999 | + | 4 | TATA | core promoter element around -30 of transcription start |
| TATA-box | Brassica oleracea | 1033 | + | 6 | ATATAA | core promoter element around -30 of transcription start |
| TATA-box | Arabidopsis thaliana | 1034 | - | 4 | TATA | core promoter element around -30 of transcription start |
| TATA-box | Avena sativa | 1065 | - | 12 | TATATTTATATTT | core promoter element around -30 of transcription start |
| TATA-box | Arabidopsis thaliana | 1068 | - | 4 | TATA | core promoter element around -30 of transcription start |
| TATA-box | Arabidopsis thaliana | 1074 | - | 4 | TATA | core promoter element around -30 of transcription start |
| TATA-box | Arabidopsis thaliana | 1086 | - | 4 | TATA | core promoter element around -30 of transcription start |
| TATA-box | Brassica napus | 1126 | - | 6 | ATATAT | core promoter element around -30 of transcription start |
| TATA-box | Arabidopsis thaliana | 1127 | - | 4 | TATA | core promoter element around -30 of transcription start |
| TATA-box | Brassica napus | 1202 | - | 6 | ATATAT | core promoter element around -30 of transcription start |
| TATA-box | Arabidopsis thaliana | 1203 | - | 6 | TATATA | core promoter element around -30 of transcription start |
| TATA-box | Arabidopsis thaliana | 1205 | - | 4 | TATA | core promoter element around -30 of transcription start |
| TATA-box | Brassica juncea | 1219 | - | 7 | TATAAAT | core promoter element around -30 of transcription start |
| TATA-box | Helianthus annuus | 1220 | - | 6 | TATAAA | core promoter element around -30 of transcription start |
| TATA-box | Arabidopsis thaliana | 1221 | - | 5 | TATAA | core promoter element around -30 of transcription start |
| TATA-box | Arabidopsis thaliana | 1222 | - | 4 | TATA | core promoter element around -30 of transcription start |
| TATA-box | Arabidopsis thaliana | 1240 | - | 5 | TATAA | core promoter element around -30 of transcription start |
| TATA-box | Arabidopsis thaliana | 1241 | - | 4 | TATA | core promoter element around -30 of transcription start |
| TATA-box | Arabidopsis thaliana | 1248 | - | 4 | TATA | core promoter element around -30 of transcription start |
| TATA-box | Brassica napus | 1260 | - | 6 | ATATAT | core promoter element around -30 of transcription start |
| TATA-box | Arabidopsis thaliana | 1261 | - | 4 | TATA | core promoter element around -30 of transcription start |
| TATA-box | Arabidopsis thaliana | 1290 | - | 4 | TATA | core promoter element around -30 of transcription start |
| TATA-box | Helianthus annuus | 1335 | - | 6 | TATACA | core promoter element around -30 of transcription start |
| TATA-box | Arabidopsis thaliana | 1337 | - | 4 | TATA | core promoter element around -30 of transcription start |
| TATA-box | Brassica napus | 1557 | + | 6 | ATTATA | core promoter element around -30 of transcription start |
| TATA-box | Arabidopsis thaliana | 1558 | - | 5 | TATAA | core promoter element around -30 of transcription start |
| TATA-box | Arabidopsis thaliana | 1559 | - | 4 | TATA | core promoter element around -30 of transcription start |

>PlantCARE\_4926   
+ TAAAGTTGTC GTGATTTCTT GCCCTTTATT TTTTATTTAT ATTAACATAT ATTTTTAAAA AAAAATTAAA   
  
  
+ GACGTTCCTA ACGGAAACTC ATGTATTTTT TGATTATGAA TATGAATATC CCCTCTATAT TAATCATGGA   
  
  
+ GCATTACAAC ATGTTTTCGT AGCCATATGT CATCACGAGA ATGATTTTTA GAATTGTTAG AAAAATAAAT   
  
  
+ TGATTCATAT AAACATATAC TATGTTTTTT ATTAAACTAA CTATCAAATT AATTAATAGT GTACAAAAAA   
  
  
+ ATATTTTTTT CTTTCCTTAA ATAAAAACTA CGGAATTACC TAATATGGCT AACATATATA TGACAATTAA   
  
  
+ TGATTATGAA TAATACATAT TTGATAAAAA AATTTCTAAC CTCTCTCTTT TTTGTTTAAT TTTATATTAT   
  
  
+ TAAAGGAAAT TTAACAATCA CATTAATCAT ATAATAAAAA CAATTAGATT TTTTCTTATA TGTTATATTT   
  
  
+ TGAATTTTTA AAAACGACTA TAAATTACTA AAAATGATAA GAGTCCCACA TTAAAAAATT TGTGATCAAC   
  
  
+ CGTTTAACTT TTTTTTTAGT TCAAGCAAGA TACAAATGAT CATATATCTG ATATAGACGT GGGCGTTCGG   
  
  
+ ATACACGTTC GGGTTTGTAT CAGATATTTC AGTATAAAGG TATAGAACCC GTTCGGGTAT TTCTACACTC   
  
  
+ CGAGTCGGGT TCGGGTTCGG ATATTTTGGA TCGGGTTCGA ATATTTAAAT TTTGAAGAAA AAAAGAAATT   
  
  
+ ATTCACTGTT TAAGTTTTTT ATATTTAAAT ATATCTTAAC TTAACTGATT TTTTTTAGTT TTTAAAAGAT   
  
  
+ TAAAATATTA ATATGTTTGG AGATAAAACT TTAAAAATAG AAAGACACTA ATTTAGTTTT TGTTTTGAAA   
  
  
+ ATTTAGATGC AACTTTTGTT AATGCAAGAA ACAAGAACTT GATATGTATT TTAAGTGAGT AACAAATGAT   
  
  
+ TTTGTCTATA GTTATATGTA TATTATCTAA TTTTGAGTAA TAAGAATCAT TAATATAAAT ATTTTGAATA   
  
  
+ AAATTAGATA GATAAACTAT AAATATAGAG TTAAGTATAC TTATGTTTGG TTATCTTCGA ATATTACCCG   
  
  
+ TTCGGATATA TTATCTGAAC TGGTGAAATA AGTAATATGT TTTGTTGTTT TAATTAGATA ATTTTTAGAC   
  
  
+ CGAGCTTGTG AATATATACT AGACAAACAT TTATATTTCG AGTCTGCACT TATATTCTAT AAGAGCTTGA   
  
  
+ TATATTAGAT TTGAACACTA ACCTGTTAAT ATAGTTTGCC GGTGATTTTT TTTCAAAATT TTGATTCTTA   
  
  
+ GATATGTATA TGGAGTAAAA CTAATTTTTA CAGATGCCCA TTTTTTTAAT TGACACTTAT GTAATTAACT   
  
  
+ GAATTCATAA AACAAGGTTT TTTAAAAAAA TTTAACTCAT ATCAATGAAA CAAAGACGAG AACGAAAGCA   
  
  
+ CAATTCTATG GAAATGGAAA ATGAAGTCAC TTATGGAGAT TCAATAGTAA GCAAATCGAG AGCAGAAAAT   
  
  
+ CTAATCTCCT TTCGTCATTA TACAATCAAT ATTGCCTATT TGGTTTTAGT GATTTGTTTC AGCCGCAAAA   
  
  
+ CTTAATTTTC TTTGGTGCAT ATGAAATCTT AAAAAGAATT AAAATAAGAT ATAATACGTT AACTCTTCAA   
  
  
+ CAACATGATA TATTTAAGAT ACCAATATTT GTATTCATCA TATAAAAATT GTAGTGTTGC AAAATATTAA   
  
  
+ AATTATTTCA TAAATAAACA TTATTATAAG AACTGACTCC GCGGATTATC ATATGGTATA GATTACAGAG   
  
  
+ TGGGTGGGTT TAAATAATTT CCCCGACACA TTATACTTTT AGAAGAGCTA TTAGCTCCAA ATTATTTAAA   
  
  
+ CAATGTTCTA AAGAGCAAAA AAATCAATGT TTTGGATTTT GATCCGACCG AGAGGACTTG TCCGACCATT   
  
  
+ CCATATTAAA ATGTTCCTAG TTCTGATTAG CTAATACCA  

- ATTTCAACAG CACTAAAGAA CGGGAAATAA AAAATAAATA TAATTGTATA TAAAAATTTT TTTTTAATTT   
  
  
- CTGCAAGGAT TGCCTTTGAG TACATAAAAA ACTAATACTT ATACTTATAG GGGAGATATA ATTAGTACCT   
  
  
- CGTAATGTTG TACAAAAGCA TCGGTATACA GTAGTGCTCT TACTAAAAAT CTTAACAATC TTTTTATTTA   
  
  
- ACTAAGTATA TTTGTATATG ATACAAAAAA TAATTTGATT GATAGTTTAA TTAATTATCA CATGTTTTTT   
  
  
- TATAAAAAAA GAAAGGAATT TATTTTTGAT GCCTTAATGG ATTATACCGA TTGTATATAT ACTGTTAATT   
  
  
- ACTAATACTT ATTATGTATA AACTATTTTT TTAAAGATTG GAGAGAGAAA AAACAAATTA AAATATAATA   
  
  
- ATTTCCTTTA AATTGTTAGT GTAATTAGTA TATTATTTTT GTTAATCTAA AAAAGAATAT ACAATATAAA   
  
  
- ACTTAAAAAT TTTTGCTGAT ATTTAATGAT TTTTACTATT CTCAGGGTGT AATTTTTTAA ACACTAGTTG   
  
  
- GCAAATTGAA AAAAAAATCA AGTTCGTTCT ATGTTTACTA GTATATAGAC TATATCTGCA CCCGCAAGCC   
  
  
- TATGTGCAAG CCCAAACATA GTCTATAAAG TCATATTTCC ATATCTTGGG CAAGCCCATA AAGATGTGAG   
  
  
- GCTCAGCCCA AGCCCAAGCC TATAAAACCT AGCCCAAGCT TATAAATTTA AAACTTCTTT TTTTCTTTAA   
  
  
- TAAGTGACAA ATTCAAAAAA TATAAATTTA TATAGAATTG AATTGACTAA AAAAAATCAA AAATTTTCTA   
  
  
- ATTTTATAAT TATACAAACC TCTATTTTGA AATTTTTATC TTTCTGTGAT TAAATCAAAA ACAAAACTTT   
  
  
- TAAATCTACG TTGAAAACAA TTACGTTCTT TGTTCTTGAA CTATACATAA AATTCACTCA TTGTTTACTA   
  
  
- AAACAGATAT CAATATACAT ATAATAGATT AAAACTCATT ATTCTTAGTA ATTATATTTA TAAAACTTAT   
  
  
- TTTAATCTAT CTATTTGATA TTTATATCTC AATTCATATG AATACAAACC AATAGAAGCT TATAATGGGC   
  
  
- AAGCCTATAT AATAGACTTG ACCACTTTAT TCATTATACA AAACAACAAA ATTAATCTAT TAAAAATCTG   
  
  
- GCTCGAACAC TTATATATGA TCTGTTTGTA AATATAAAGC TCAGACGTGA ATATAAGATA TTCTCGAACT   
  
  
- ATATAATCTA AACTTGTGAT TGGACAATTA TATCAAACGG CCACTAAAAA AAAGTTTTAA AACTAAGAAT   
  
  
- CTATACATAT ACCTCATTTT GATTAAAAAT GTCTACGGGT AAAAAAATTA ACTGTGAATA CATTAATTGA   
  
  
- CTTAAGTATT TTGTTCCAAA AAATTTTTTT AAATTGAGTA TAGTTACTTT GTTTCTGCTC TTGCTTTCGT   
  
  
- GTTAAGATAC CTTTACCTTT TACTTCAGTG AATACCTCTA AGTTATCATT CGTTTAGCTC TCGTCTTTTA   
  
  
- GATTAGAGGA AAGCAGTAAT ATGTTAGTTA TAACGGATAA ACCAAAATCA CTAAACAAAG TCGGCGTTTT   
  
  
- GAATTAAAAG AAACCACGTA TACTTTAGAA TTTTTCTTAA TTTTATTCTA TATTATGCAA TTGAGAAGTT   
  
  
- GTTGTACTAT ATAAATTCTA TGGTTATAAA CATAAGTAGT ATATTTTTAA CATCACAACG TTTTATAATT   
  
  
- TTAATAAAGT ATTTATTTGT AATAATATTC TTGACTGAGG CGCCTAATAG TATACCATAT CTAATGTCTC   
  
  
- ACCCACCCAA ATTTATTAAA GGGGCTGTGT AATATGAAAA TCTTCTCGAT AATCGAGGTT TAATAAATTT   
  
  
- GTTACAAGAT TTCTCGTTTT TTTAGTTACA AAACCTAAAA CTAGGCTGGC TCTCCTGAAC AGGCTGGTAA   
  
  
- GGTATAATTT TACAAGGATC AAGACTAATC GATTATGGT

+     TCA

| Site Name | Organism | Position | Strand | Matrix score. | sequence | function |
| --- | --- | --- | --- | --- | --- | --- |
| TCA | Pisum sativum | 106 | - | 9 | TCATCTTCAT |  |

>PlantCARE\_4926   
+ TAAAGTTGTC GTGATTTCTT GCCCTTTATT TTTTATTTAT ATTAACATAT ATTTTTAAAA AAAAATTAAA   
  
  
+ GACGTTCCTA ACGGAAACTC ATGTATTTTT TGATTATGAA TATGAATATC CCCTCTATAT TAATCATGGA   
  
  
+ GCATTACAAC ATGTTTTCGT AGCCATATGT CATCACGAGA ATGATTTTTA GAATTGTTAG AAAAATAAAT   
  
  
+ TGATTCATAT AAACATATAC TATGTTTTTT ATTAAACTAA CTATCAAATT AATTAATAGT GTACAAAAAA   
  
  
+ ATATTTTTTT CTTTCCTTAA ATAAAAACTA CGGAATTACC TAATATGGCT AACATATATA TGACAATTAA   
  
  
+ TGATTATGAA TAATACATAT TTGATAAAAA AATTTCTAAC CTCTCTCTTT TTTGTTTAAT TTTATATTAT   
  
  
+ TAAAGGAAAT TTAACAATCA CATTAATCAT ATAATAAAAA CAATTAGATT TTTTCTTATA TGTTATATTT   
  
  
+ TGAATTTTTA AAAACGACTA TAAATTACTA AAAATGATAA GAGTCCCACA TTAAAAAATT TGTGATCAAC   
  
  
+ CGTTTAACTT TTTTTTTAGT TCAAGCAAGA TACAAATGAT CATATATCTG ATATAGACGT GGGCGTTCGG   
  
  
+ ATACACGTTC GGGTTTGTAT CAGATATTTC AGTATAAAGG TATAGAACCC GTTCGGGTAT TTCTACACTC   
  
  
+ CGAGTCGGGT TCGGGTTCGG ATATTTTGGA TCGGGTTCGA ATATTTAAAT TTTGAAGAAA AAAAGAAATT   
  
  
+ ATTCACTGTT TAAGTTTTTT ATATTTAAAT ATATCTTAAC TTAACTGATT TTTTTTAGTT TTTAAAAGAT   
  
  
+ TAAAATATTA ATATGTTTGG AGATAAAACT TTAAAAATAG AAAGACACTA ATTTAGTTTT TGTTTTGAAA   
  
  
+ ATTTAGATGC AACTTTTGTT AATGCAAGAA ACAAGAACTT GATATGTATT TTAAGTGAGT AACAAATGAT   
  
  
+ TTTGTCTATA GTTATATGTA TATTATCTAA TTTTGAGTAA TAAGAATCAT TAATATAAAT ATTTTGAATA   
  
  
+ AAATTAGATA GATAAACTAT AAATATAGAG TTAAGTATAC TTATGTTTGG TTATCTTCGA ATATTACCCG   
  
  
+ TTCGGATATA TTATCTGAAC TGGTGAAATA AGTAATATGT TTTGTTGTTT TAATTAGATA ATTTTTAGAC   
  
  
+ CGAGCTTGTG AATATATACT AGACAAACAT TTATATTTCG AGTCTGCACT TATATTCTAT AAGAGCTTGA   
  
  
+ TATATTAGAT TTGAACACTA ACCTGTTAAT ATAGTTTGCC GGTGATTTTT TTTCAAAATT TTGATTCTTA   
  
  
+ GATATGTATA TGGAGTAAAA CTAATTTTTA CAGATGCCCA TTTTTTTAAT TGACACTTAT GTAATTAACT   
  
  
+ GAATTCATAA AACAAGGTTT TTTAAAAAAA TTTAACTCAT ATCAATGAAA CAAAGACGAG AACGAAAGCA   
  
  
+ CAATTCTATG GAAATGGAAA ATGAAGTCAC TTATGGAGAT TCAATAGTAA GCAAATCGAG AGCAGAAAAT   
  
  
+ CTAATCTCCT TTCGTCATTA TACAATCAAT ATTGCCTATT TGGTTTTAGT GATTTGTTTC AGCCGCAAAA   
  
  
+ CTTAATTTTC TTTGGTGCAT ATGAAATCTT AAAAAGAATT AAAATAAGAT ATAATACGTT AACTCTTCAA   
  
  
+ CAACATGATA TATTTAAGAT ACCAATATTT GTATTCATCA TATAAAAATT GTAGTGTTGC AAAATATTAA   
  
  
+ AATTATTTCA TAAATAAACA TTATTATAAG AACTGACTCC GCGGATTATC ATATGGTATA GATTACAGAG   
  
  
+ TGGGTGGGTT TAAATAATTT CCCCGACACA TTATACTTTT AGAAGAGCTA TTAGCTCCAA ATTATTTAAA   
  
  
+ CAATGTTCTA AAGAGCAAAA AAATCAATGT TTTGGATTTT GATCCGACCG AGAGGACTTG TCCGACCATT   
  
  
+ CCATATTAAA ATGTTCCTAG TTCTGATTAG CTAATACCA  

- ATTTCAACAG CACTAAAGAA CGGGAAATAA AAAATAAATA TAATTGTATA TAAAAATTTT TTTTTAATTT   
  
  
- CTGCAAGGAT TGCCTTTGAG TACATAAAAA ACTAATACTT ATACTTATAG GGGAGATATA ATTAGTACCT   
  
  
- CGTAATGTTG TACAAAAGCA TCGGTATACA GTAGTGCTCT TACTAAAAAT CTTAACAATC TTTTTATTTA   
  
  
- ACTAAGTATA TTTGTATATG ATACAAAAAA TAATTTGATT GATAGTTTAA TTAATTATCA CATGTTTTTT   
  
  
- TATAAAAAAA GAAAGGAATT TATTTTTGAT GCCTTAATGG ATTATACCGA TTGTATATAT ACTGTTAATT   
  
  
- ACTAATACTT ATTATGTATA AACTATTTTT TTAAAGATTG GAGAGAGAAA AAACAAATTA AAATATAATA   
  
  
- ATTTCCTTTA AATTGTTAGT GTAATTAGTA TATTATTTTT GTTAATCTAA AAAAGAATAT ACAATATAAA   
  
  
- ACTTAAAAAT TTTTGCTGAT ATTTAATGAT TTTTACTATT CTCAGGGTGT AATTTTTTAA ACACTAGTTG   
  
  
- GCAAATTGAA AAAAAAATCA AGTTCGTTCT ATGTTTACTA GTATATAGAC TATATCTGCA CCCGCAAGCC   
  
  
- TATGTGCAAG CCCAAACATA GTCTATAAAG TCATATTTCC ATATCTTGGG CAAGCCCATA AAGATGTGAG   
  
  
- GCTCAGCCCA AGCCCAAGCC TATAAAACCT AGCCCAAGCT TATAAATTTA AAACTTCTTT TTTTCTTTAA   
  
  
- TAAGTGACAA ATTCAAAAAA TATAAATTTA TATAGAATTG AATTGACTAA AAAAAATCAA AAATTTTCTA   
  
  
- ATTTTATAAT TATACAAACC TCTATTTTGA AATTTTTATC TTTCTGTGAT TAAATCAAAA ACAAAACTTT   
  
  
- TAAATCTACG TTGAAAACAA TTACGTTCTT TGTTCTTGAA CTATACATAA AATTCACTCA TTGTTTACTA   
  
  
- AAACAGATAT CAATATACAT ATAATAGATT AAAACTCATT ATTCTTAGTA ATTATATTTA TAAAACTTAT   
  
  
- TTTAATCTAT CTATTTGATA TTTATATCTC AATTCATATG AATACAAACC AATAGAAGCT TATAATGGGC   
  
  
- AAGCCTATAT AATAGACTTG ACCACTTTAT TCATTATACA AAACAACAAA ATTAATCTAT TAAAAATCTG   
  
  
- GCTCGAACAC TTATATATGA TCTGTTTGTA AATATAAAGC TCAGACGTGA ATATAAGATA TTCTCGAACT   
  
  
- ATATAATCTA AACTTGTGAT TGGACAATTA TATCAAACGG CCACTAAAAA AAAGTTTTAA AACTAAGAAT   
  
  
- CTATACATAT ACCTCATTTT GATTAAAAAT GTCTACGGGT AAAAAAATTA ACTGTGAATA CATTAATTGA   
  
  
- CTTAAGTATT TTGTTCCAAA AAATTTTTTT AAATTGAGTA TAGTTACTTT GTTTCTGCTC TTGCTTTCGT   
  
  
- GTTAAGATAC CTTTACCTTT TACTTCAGTG AATACCTCTA AGTTATCATT CGTTTAGCTC TCGTCTTTTA   
  
  
- GATTAGAGGA AAGCAGTAAT ATGTTAGTTA TAACGGATAA ACCAAAATCA CTAAACAAAG TCGGCGTTTT   
  
  
- GAATTAAAAG AAACCACGTA TACTTTAGAA TTTTTCTTAA TTTTATTCTA TATTATGCAA TTGAGAAGTT   
  
  
- GTTGTACTAT ATAAATTCTA TGGTTATAAA CATAAGTAGT ATATTTTTAA CATCACAACG TTTTATAATT   
  
  
- TTAATAAAGT ATTTATTTGT AATAATATTC TTGACTGAGG CGCCTAATAG TATACCATAT CTAATGTCTC   
  
  
- ACCCACCCAA ATTTATTAAA GGGGCTGTGT AATATGAAAA TCTTCTCGAT AATCGAGGTT TAATAAATTT   
  
  
- GTTACAAGAT TTCTCGTTTT TTTAGTTACA AAACCTAAAA CTAGGCTGGC TCTCCTGAAC AGGCTGGTAA   
  
  
- GGTATAATTT TACAAGGATC AAGACTAATC GATTATGGT

+     TCA-element

| Site Name | Organism | Position | Strand | Matrix score. | sequence | function |
| --- | --- | --- | --- | --- | --- | --- |
| TCA-element | Nicotiana tabacum | 1368 | + | 9 | CCATCTTTTT | cis-acting element involved in salicylic acid responsiveness |

>PlantCARE\_4926   
+ TAAAGTTGTC GTGATTTCTT GCCCTTTATT TTTTATTTAT ATTAACATAT ATTTTTAAAA AAAAATTAAA   
  
  
+ GACGTTCCTA ACGGAAACTC ATGTATTTTT TGATTATGAA TATGAATATC CCCTCTATAT TAATCATGGA   
  
  
+ GCATTACAAC ATGTTTTCGT AGCCATATGT CATCACGAGA ATGATTTTTA GAATTGTTAG AAAAATAAAT   
  
  
+ TGATTCATAT AAACATATAC TATGTTTTTT ATTAAACTAA CTATCAAATT AATTAATAGT GTACAAAAAA   
  
  
+ ATATTTTTTT CTTTCCTTAA ATAAAAACTA CGGAATTACC TAATATGGCT AACATATATA TGACAATTAA   
  
  
+ TGATTATGAA TAATACATAT TTGATAAAAA AATTTCTAAC CTCTCTCTTT TTTGTTTAAT TTTATATTAT   
  
  
+ TAAAGGAAAT TTAACAATCA CATTAATCAT ATAATAAAAA CAATTAGATT TTTTCTTATA TGTTATATTT   
  
  
+ TGAATTTTTA AAAACGACTA TAAATTACTA AAAATGATAA GAGTCCCACA TTAAAAAATT TGTGATCAAC   
  
  
+ CGTTTAACTT TTTTTTTAGT TCAAGCAAGA TACAAATGAT CATATATCTG ATATAGACGT GGGCGTTCGG   
  
  
+ ATACACGTTC GGGTTTGTAT CAGATATTTC AGTATAAAGG TATAGAACCC GTTCGGGTAT TTCTACACTC   
  
  
+ CGAGTCGGGT TCGGGTTCGG ATATTTTGGA TCGGGTTCGA ATATTTAAAT TTTGAAGAAA AAAAGAAATT   
  
  
+ ATTCACTGTT TAAGTTTTTT ATATTTAAAT ATATCTTAAC TTAACTGATT TTTTTTAGTT TTTAAAAGAT   
  
  
+ TAAAATATTA ATATGTTTGG AGATAAAACT TTAAAAATAG AAAGACACTA ATTTAGTTTT TGTTTTGAAA   
  
  
+ ATTTAGATGC AACTTTTGTT AATGCAAGAA ACAAGAACTT GATATGTATT TTAAGTGAGT AACAAATGAT   
  
  
+ TTTGTCTATA GTTATATGTA TATTATCTAA TTTTGAGTAA TAAGAATCAT TAATATAAAT ATTTTGAATA   
  
  
+ AAATTAGATA GATAAACTAT AAATATAGAG TTAAGTATAC TTATGTTTGG TTATCTTCGA ATATTACCCG   
  
  
+ TTCGGATATA TTATCTGAAC TGGTGAAATA AGTAATATGT TTTGTTGTTT TAATTAGATA ATTTTTAGAC   
  
  
+ CGAGCTTGTG AATATATACT AGACAAACAT TTATATTTCG AGTCTGCACT TATATTCTAT AAGAGCTTGA   
  
  
+ TATATTAGAT TTGAACACTA ACCTGTTAAT ATAGTTTGCC GGTGATTTTT TTTCAAAATT TTGATTCTTA   
  
  
+ GATATGTATA TGGAGTAAAA CTAATTTTTA CAGATGCCCA TTTTTTTAAT TGACACTTAT GTAATTAACT   
  
  
+ GAATTCATAA AACAAGGTTT TTTAAAAAAA TTTAACTCAT ATCAATGAAA CAAAGACGAG AACGAAAGCA   
  
  
+ CAATTCTATG GAAATGGAAA ATGAAGTCAC TTATGGAGAT TCAATAGTAA GCAAATCGAG AGCAGAAAAT   
  
  
+ CTAATCTCCT TTCGTCATTA TACAATCAAT ATTGCCTATT TGGTTTTAGT GATTTGTTTC AGCCGCAAAA   
  
  
+ CTTAATTTTC TTTGGTGCAT ATGAAATCTT AAAAAGAATT AAAATAAGAT ATAATACGTT AACTCTTCAA   
  
  
+ CAACATGATA TATTTAAGAT ACCAATATTT GTATTCATCA TATAAAAATT GTAGTGTTGC AAAATATTAA   
  
  
+ AATTATTTCA TAAATAAACA TTATTATAAG AACTGACTCC GCGGATTATC ATATGGTATA GATTACAGAG   
  
  
+ TGGGTGGGTT TAAATAATTT CCCCGACACA TTATACTTTT AGAAGAGCTA TTAGCTCCAA ATTATTTAAA   
  
  
+ CAATGTTCTA AAGAGCAAAA AAATCAATGT TTTGGATTTT GATCCGACCG AGAGGACTTG TCCGACCATT   
  
  
+ CCATATTAAA ATGTTCCTAG TTCTGATTAG CTAATACCA  

- ATTTCAACAG CACTAAAGAA CGGGAAATAA AAAATAAATA TAATTGTATA TAAAAATTTT TTTTTAATTT   
  
  
- CTGCAAGGAT TGCCTTTGAG TACATAAAAA ACTAATACTT ATACTTATAG GGGAGATATA ATTAGTACCT   
  
  
- CGTAATGTTG TACAAAAGCA TCGGTATACA GTAGTGCTCT TACTAAAAAT CTTAACAATC TTTTTATTTA   
  
  
- ACTAAGTATA TTTGTATATG ATACAAAAAA TAATTTGATT GATAGTTTAA TTAATTATCA CATGTTTTTT   
  
  
- TATAAAAAAA GAAAGGAATT TATTTTTGAT GCCTTAATGG ATTATACCGA TTGTATATAT ACTGTTAATT   
  
  
- ACTAATACTT ATTATGTATA AACTATTTTT TTAAAGATTG GAGAGAGAAA AAACAAATTA AAATATAATA   
  
  
- ATTTCCTTTA AATTGTTAGT GTAATTAGTA TATTATTTTT GTTAATCTAA AAAAGAATAT ACAATATAAA   
  
  
- ACTTAAAAAT TTTTGCTGAT ATTTAATGAT TTTTACTATT CTCAGGGTGT AATTTTTTAA ACACTAGTTG   
  
  
- GCAAATTGAA AAAAAAATCA AGTTCGTTCT ATGTTTACTA GTATATAGAC TATATCTGCA CCCGCAAGCC   
  
  
- TATGTGCAAG CCCAAACATA GTCTATAAAG TCATATTTCC ATATCTTGGG CAAGCCCATA AAGATGTGAG   
  
  
- GCTCAGCCCA AGCCCAAGCC TATAAAACCT AGCCCAAGCT TATAAATTTA AAACTTCTTT TTTTCTTTAA   
  
  
- TAAGTGACAA ATTCAAAAAA TATAAATTTA TATAGAATTG AATTGACTAA AAAAAATCAA AAATTTTCTA   
  
  
- ATTTTATAAT TATACAAACC TCTATTTTGA AATTTTTATC TTTCTGTGAT TAAATCAAAA ACAAAACTTT   
  
  
- TAAATCTACG TTGAAAACAA TTACGTTCTT TGTTCTTGAA CTATACATAA AATTCACTCA TTGTTTACTA   
  
  
- AAACAGATAT CAATATACAT ATAATAGATT AAAACTCATT ATTCTTAGTA ATTATATTTA TAAAACTTAT   
  
  
- TTTAATCTAT CTATTTGATA TTTATATCTC AATTCATATG AATACAAACC AATAGAAGCT TATAATGGGC   
  
  
- AAGCCTATAT AATAGACTTG ACCACTTTAT TCATTATACA AAACAACAAA ATTAATCTAT TAAAAATCTG   
  
  
- GCTCGAACAC TTATATATGA TCTGTTTGTA AATATAAAGC TCAGACGTGA ATATAAGATA TTCTCGAACT   
  
  
- ATATAATCTA AACTTGTGAT TGGACAATTA TATCAAACGG CCACTAAAAA AAAGTTTTAA AACTAAGAAT   
  
  
- CTATACATAT ACCTCATTTT GATTAAAAAT GTCTACGGGT AAAAAAATTA ACTGTGAATA CATTAATTGA   
  
  
- CTTAAGTATT TTGTTCCAAA AAATTTTTTT AAATTGAGTA TAGTTACTTT GTTTCTGCTC TTGCTTTCGT   
  
  
- GTTAAGATAC CTTTACCTTT TACTTCAGTG AATACCTCTA AGTTATCATT CGTTTAGCTC TCGTCTTTTA   
  
  
- GATTAGAGGA AAGCAGTAAT ATGTTAGTTA TAACGGATAA ACCAAAATCA CTAAACAAAG TCGGCGTTTT   
  
  
- GAATTAAAAG AAACCACGTA TACTTTAGAA TTTTTCTTAA TTTTATTCTA TATTATGCAA TTGAGAAGTT   
  
  
- GTTGTACTAT ATAAATTCTA TGGTTATAAA CATAAGTAGT ATATTTTTAA CATCACAACG TTTTATAATT   
  
  
- TTAATAAAGT ATTTATTTGT AATAATATTC TTGACTGAGG CGCCTAATAG TATACCATAT CTAATGTCTC   
  
  
- ACCCACCCAA ATTTATTAAA GGGGCTGTGT AATATGAAAA TCTTCTCGAT AATCGAGGTT TAATAAATTT   
  
  
- GTTACAAGAT TTCTCGTTTT TTTAGTTACA AAACCTAAAA CTAGGCTGGC TCTCCTGAAC AGGCTGGTAA   
  
  
- GGTATAATTT TACAAGGATC AAGACTAATC GATTATGGT

+     TGA-element

| Site Name | Organism | Position | Strand | Matrix score. | sequence | function |
| --- | --- | --- | --- | --- | --- | --- |
| TGA-element | Brassica oleracea | 503 | + | 6 | AACGAC | auxin-responsive element |

>PlantCARE\_4926   
+ TAAAGTTGTC GTGATTTCTT GCCCTTTATT TTTTATTTAT ATTAACATAT ATTTTTAAAA AAAAATTAAA   
  
  
+ GACGTTCCTA ACGGAAACTC ATGTATTTTT TGATTATGAA TATGAATATC CCCTCTATAT TAATCATGGA   
  
  
+ GCATTACAAC ATGTTTTCGT AGCCATATGT CATCACGAGA ATGATTTTTA GAATTGTTAG AAAAATAAAT   
  
  
+ TGATTCATAT AAACATATAC TATGTTTTTT ATTAAACTAA CTATCAAATT AATTAATAGT GTACAAAAAA   
  
  
+ ATATTTTTTT CTTTCCTTAA ATAAAAACTA CGGAATTACC TAATATGGCT AACATATATA TGACAATTAA   
  
  
+ TGATTATGAA TAATACATAT TTGATAAAAA AATTTCTAAC CTCTCTCTTT TTTGTTTAAT TTTATATTAT   
  
  
+ TAAAGGAAAT TTAACAATCA CATTAATCAT ATAATAAAAA CAATTAGATT TTTTCTTATA TGTTATATTT   
  
  
+ TGAATTTTTA AAAACGACTA TAAATTACTA AAAATGATAA GAGTCCCACA TTAAAAAATT TGTGATCAAC   
  
  
+ CGTTTAACTT TTTTTTTAGT TCAAGCAAGA TACAAATGAT CATATATCTG ATATAGACGT GGGCGTTCGG   
  
  
+ ATACACGTTC GGGTTTGTAT CAGATATTTC AGTATAAAGG TATAGAACCC GTTCGGGTAT TTCTACACTC   
  
  
+ CGAGTCGGGT TCGGGTTCGG ATATTTTGGA TCGGGTTCGA ATATTTAAAT TTTGAAGAAA AAAAGAAATT   
  
  
+ ATTCACTGTT TAAGTTTTTT ATATTTAAAT ATATCTTAAC TTAACTGATT TTTTTTAGTT TTTAAAAGAT   
  
  
+ TAAAATATTA ATATGTTTGG AGATAAAACT TTAAAAATAG AAAGACACTA ATTTAGTTTT TGTTTTGAAA   
  
  
+ ATTTAGATGC AACTTTTGTT AATGCAAGAA ACAAGAACTT GATATGTATT TTAAGTGAGT AACAAATGAT   
  
  
+ TTTGTCTATA GTTATATGTA TATTATCTAA TTTTGAGTAA TAAGAATCAT TAATATAAAT ATTTTGAATA   
  
  
+ AAATTAGATA GATAAACTAT AAATATAGAG TTAAGTATAC TTATGTTTGG TTATCTTCGA ATATTACCCG   
  
  
+ TTCGGATATA TTATCTGAAC TGGTGAAATA AGTAATATGT TTTGTTGTTT TAATTAGATA ATTTTTAGAC   
  
  
+ CGAGCTTGTG AATATATACT AGACAAACAT TTATATTTCG AGTCTGCACT TATATTCTAT AAGAGCTTGA   
  
  
+ TATATTAGAT TTGAACACTA ACCTGTTAAT ATAGTTTGCC GGTGATTTTT TTTCAAAATT TTGATTCTTA   
  
  
+ GATATGTATA TGGAGTAAAA CTAATTTTTA CAGATGCCCA TTTTTTTAAT TGACACTTAT GTAATTAACT   
  
  
+ GAATTCATAA AACAAGGTTT TTTAAAAAAA TTTAACTCAT ATCAATGAAA CAAAGACGAG AACGAAAGCA   
  
  
+ CAATTCTATG GAAATGGAAA ATGAAGTCAC TTATGGAGAT TCAATAGTAA GCAAATCGAG AGCAGAAAAT   
  
  
+ CTAATCTCCT TTCGTCATTA TACAATCAAT ATTGCCTATT TGGTTTTAGT GATTTGTTTC AGCCGCAAAA   
  
  
+ CTTAATTTTC TTTGGTGCAT ATGAAATCTT AAAAAGAATT AAAATAAGAT ATAATACGTT AACTCTTCAA   
  
  
+ CAACATGATA TATTTAAGAT ACCAATATTT GTATTCATCA TATAAAAATT GTAGTGTTGC AAAATATTAA   
  
  
+ AATTATTTCA TAAATAAACA TTATTATAAG AACTGACTCC GCGGATTATC ATATGGTATA GATTACAGAG   
  
  
+ TGGGTGGGTT TAAATAATTT CCCCGACACA TTATACTTTT AGAAGAGCTA TTAGCTCCAA ATTATTTAAA   
  
  
+ CAATGTTCTA AAGAGCAAAA AAATCAATGT TTTGGATTTT GATCCGACCG AGAGGACTTG TCCGACCATT   
  
  
+ CCATATTAAA ATGTTCCTAG TTCTGATTAG CTAATACCA  

- ATTTCAACAG CACTAAAGAA CGGGAAATAA AAAATAAATA TAATTGTATA TAAAAATTTT TTTTTAATTT   
  
  
- CTGCAAGGAT TGCCTTTGAG TACATAAAAA ACTAATACTT ATACTTATAG GGGAGATATA ATTAGTACCT   
  
  
- CGTAATGTTG TACAAAAGCA TCGGTATACA GTAGTGCTCT TACTAAAAAT CTTAACAATC TTTTTATTTA   
  
  
- ACTAAGTATA TTTGTATATG ATACAAAAAA TAATTTGATT GATAGTTTAA TTAATTATCA CATGTTTTTT   
  
  
- TATAAAAAAA GAAAGGAATT TATTTTTGAT GCCTTAATGG ATTATACCGA TTGTATATAT ACTGTTAATT   
  
  
- ACTAATACTT ATTATGTATA AACTATTTTT TTAAAGATTG GAGAGAGAAA AAACAAATTA AAATATAATA   
  
  
- ATTTCCTTTA AATTGTTAGT GTAATTAGTA TATTATTTTT GTTAATCTAA AAAAGAATAT ACAATATAAA   
  
  
- ACTTAAAAAT TTTTGCTGAT ATTTAATGAT TTTTACTATT CTCAGGGTGT AATTTTTTAA ACACTAGTTG   
  
  
- GCAAATTGAA AAAAAAATCA AGTTCGTTCT ATGTTTACTA GTATATAGAC TATATCTGCA CCCGCAAGCC   
  
  
- TATGTGCAAG CCCAAACATA GTCTATAAAG TCATATTTCC ATATCTTGGG CAAGCCCATA AAGATGTGAG   
  
  
- GCTCAGCCCA AGCCCAAGCC TATAAAACCT AGCCCAAGCT TATAAATTTA AAACTTCTTT TTTTCTTTAA   
  
  
- TAAGTGACAA ATTCAAAAAA TATAAATTTA TATAGAATTG AATTGACTAA AAAAAATCAA AAATTTTCTA   
  
  
- ATTTTATAAT TATACAAACC TCTATTTTGA AATTTTTATC TTTCTGTGAT TAAATCAAAA ACAAAACTTT   
  
  
- TAAATCTACG TTGAAAACAA TTACGTTCTT TGTTCTTGAA CTATACATAA AATTCACTCA TTGTTTACTA   
  
  
- AAACAGATAT CAATATACAT ATAATAGATT AAAACTCATT ATTCTTAGTA ATTATATTTA TAAAACTTAT   
  
  
- TTTAATCTAT CTATTTGATA TTTATATCTC AATTCATATG AATACAAACC AATAGAAGCT TATAATGGGC   
  
  
- AAGCCTATAT AATAGACTTG ACCACTTTAT TCATTATACA AAACAACAAA ATTAATCTAT TAAAAATCTG   
  
  
- GCTCGAACAC TTATATATGA TCTGTTTGTA AATATAAAGC TCAGACGTGA ATATAAGATA TTCTCGAACT   
  
  
- ATATAATCTA AACTTGTGAT TGGACAATTA TATCAAACGG CCACTAAAAA AAAGTTTTAA AACTAAGAAT   
  
  
- CTATACATAT ACCTCATTTT GATTAAAAAT GTCTACGGGT AAAAAAATTA ACTGTGAATA CATTAATTGA   
  
  
- CTTAAGTATT TTGTTCCAAA AAATTTTTTT AAATTGAGTA TAGTTACTTT GTTTCTGCTC TTGCTTTCGT   
  
  
- GTTAAGATAC CTTTACCTTT TACTTCAGTG AATACCTCTA AGTTATCATT CGTTTAGCTC TCGTCTTTTA   
  
  
- GATTAGAGGA AAGCAGTAAT ATGTTAGTTA TAACGGATAA ACCAAAATCA CTAAACAAAG TCGGCGTTTT   
  
  
- GAATTAAAAG AAACCACGTA TACTTTAGAA TTTTTCTTAA TTTTATTCTA TATTATGCAA TTGAGAAGTT   
  
  
- GTTGTACTAT ATAAATTCTA TGGTTATAAA CATAAGTAGT ATATTTTTAA CATCACAACG TTTTATAATT   
  
  
- TTAATAAAGT ATTTATTTGT AATAATATTC TTGACTGAGG CGCCTAATAG TATACCATAT CTAATGTCTC   
  
  
- ACCCACCCAA ATTTATTAAA GGGGCTGTGT AATATGAAAA TCTTCTCGAT AATCGAGGTT TAATAAATTT   
  
  
- GTTACAAGAT TTCTCGTTTT TTTAGTTACA AAACCTAAAA CTAGGCTGGC TCTCCTGAAC AGGCTGGTAA   
  
  
- GGTATAATTT TACAAGGATC AAGACTAATC GATTATGGT

+     TGACG-motif

| Site Name | Organism | Position | Strand | Matrix score. | sequence | function |
| --- | --- | --- | --- | --- | --- | --- |
| TGACG-motif | Hordeum vulgare | 1553 | - | 5 | TGACG | cis-acting regulatory element involved in the MeJA-responsiveness |

>PlantCARE\_4926   
+ TAAAGTTGTC GTGATTTCTT GCCCTTTATT TTTTATTTAT ATTAACATAT ATTTTTAAAA AAAAATTAAA   
  
  
+ GACGTTCCTA ACGGAAACTC ATGTATTTTT TGATTATGAA TATGAATATC CCCTCTATAT TAATCATGGA   
  
  
+ GCATTACAAC ATGTTTTCGT AGCCATATGT CATCACGAGA ATGATTTTTA GAATTGTTAG AAAAATAAAT   
  
  
+ TGATTCATAT AAACATATAC TATGTTTTTT ATTAAACTAA CTATCAAATT AATTAATAGT GTACAAAAAA   
  
  
+ ATATTTTTTT CTTTCCTTAA ATAAAAACTA CGGAATTACC TAATATGGCT AACATATATA TGACAATTAA   
  
  
+ TGATTATGAA TAATACATAT TTGATAAAAA AATTTCTAAC CTCTCTCTTT TTTGTTTAAT TTTATATTAT   
  
  
+ TAAAGGAAAT TTAACAATCA CATTAATCAT ATAATAAAAA CAATTAGATT TTTTCTTATA TGTTATATTT   
  
  
+ TGAATTTTTA AAAACGACTA TAAATTACTA AAAATGATAA GAGTCCCACA TTAAAAAATT TGTGATCAAC   
  
  
+ CGTTTAACTT TTTTTTTAGT TCAAGCAAGA TACAAATGAT CATATATCTG ATATAGACGT GGGCGTTCGG   
  
  
+ ATACACGTTC GGGTTTGTAT CAGATATTTC AGTATAAAGG TATAGAACCC GTTCGGGTAT TTCTACACTC   
  
  
+ CGAGTCGGGT TCGGGTTCGG ATATTTTGGA TCGGGTTCGA ATATTTAAAT TTTGAAGAAA AAAAGAAATT   
  
  
+ ATTCACTGTT TAAGTTTTTT ATATTTAAAT ATATCTTAAC TTAACTGATT TTTTTTAGTT TTTAAAAGAT   
  
  
+ TAAAATATTA ATATGTTTGG AGATAAAACT TTAAAAATAG AAAGACACTA ATTTAGTTTT TGTTTTGAAA   
  
  
+ ATTTAGATGC AACTTTTGTT AATGCAAGAA ACAAGAACTT GATATGTATT TTAAGTGAGT AACAAATGAT   
  
  
+ TTTGTCTATA GTTATATGTA TATTATCTAA TTTTGAGTAA TAAGAATCAT TAATATAAAT ATTTTGAATA   
  
  
+ AAATTAGATA GATAAACTAT AAATATAGAG TTAAGTATAC TTATGTTTGG TTATCTTCGA ATATTACCCG   
  
  
+ TTCGGATATA TTATCTGAAC TGGTGAAATA AGTAATATGT TTTGTTGTTT TAATTAGATA ATTTTTAGAC   
  
  
+ CGAGCTTGTG AATATATACT AGACAAACAT TTATATTTCG AGTCTGCACT TATATTCTAT AAGAGCTTGA   
  
  
+ TATATTAGAT TTGAACACTA ACCTGTTAAT ATAGTTTGCC GGTGATTTTT TTTCAAAATT TTGATTCTTA   
  
  
+ GATATGTATA TGGAGTAAAA CTAATTTTTA CAGATGCCCA TTTTTTTAAT TGACACTTAT GTAATTAACT   
  
  
+ GAATTCATAA AACAAGGTTT TTTAAAAAAA TTTAACTCAT ATCAATGAAA CAAAGACGAG AACGAAAGCA   
  
  
+ CAATTCTATG GAAATGGAAA ATGAAGTCAC TTATGGAGAT TCAATAGTAA GCAAATCGAG AGCAGAAAAT   
  
  
+ CTAATCTCCT TTCGTCATTA TACAATCAAT ATTGCCTATT TGGTTTTAGT GATTTGTTTC AGCCGCAAAA   
  
  
+ CTTAATTTTC TTTGGTGCAT ATGAAATCTT AAAAAGAATT AAAATAAGAT ATAATACGTT AACTCTTCAA   
  
  
+ CAACATGATA TATTTAAGAT ACCAATATTT GTATTCATCA TATAAAAATT GTAGTGTTGC AAAATATTAA   
  
  
+ AATTATTTCA TAAATAAACA TTATTATAAG AACTGACTCC GCGGATTATC ATATGGTATA GATTACAGAG   
  
  
+ TGGGTGGGTT TAAATAATTT CCCCGACACA TTATACTTTT AGAAGAGCTA TTAGCTCCAA ATTATTTAAA   
  
  
+ CAATGTTCTA AAGAGCAAAA AAATCAATGT TTTGGATTTT GATCCGACCG AGAGGACTTG TCCGACCATT   
  
  
+ CCATATTAAA ATGTTCCTAG TTCTGATTAG CTAATACCA  

- ATTTCAACAG CACTAAAGAA CGGGAAATAA AAAATAAATA TAATTGTATA TAAAAATTTT TTTTTAATTT   
  
  
- CTGCAAGGAT TGCCTTTGAG TACATAAAAA ACTAATACTT ATACTTATAG GGGAGATATA ATTAGTACCT   
  
  
- CGTAATGTTG TACAAAAGCA TCGGTATACA GTAGTGCTCT TACTAAAAAT CTTAACAATC TTTTTATTTA   
  
  
- ACTAAGTATA TTTGTATATG ATACAAAAAA TAATTTGATT GATAGTTTAA TTAATTATCA CATGTTTTTT   
  
  
- TATAAAAAAA GAAAGGAATT TATTTTTGAT GCCTTAATGG ATTATACCGA TTGTATATAT ACTGTTAATT   
  
  
- ACTAATACTT ATTATGTATA AACTATTTTT TTAAAGATTG GAGAGAGAAA AAACAAATTA AAATATAATA   
  
  
- ATTTCCTTTA AATTGTTAGT GTAATTAGTA TATTATTTTT GTTAATCTAA AAAAGAATAT ACAATATAAA   
  
  
- ACTTAAAAAT TTTTGCTGAT ATTTAATGAT TTTTACTATT CTCAGGGTGT AATTTTTTAA ACACTAGTTG   
  
  
- GCAAATTGAA AAAAAAATCA AGTTCGTTCT ATGTTTACTA GTATATAGAC TATATCTGCA CCCGCAAGCC   
  
  
- TATGTGCAAG CCCAAACATA GTCTATAAAG TCATATTTCC ATATCTTGGG CAAGCCCATA AAGATGTGAG   
  
  
- GCTCAGCCCA AGCCCAAGCC TATAAAACCT AGCCCAAGCT TATAAATTTA AAACTTCTTT TTTTCTTTAA   
  
  
- TAAGTGACAA ATTCAAAAAA TATAAATTTA TATAGAATTG AATTGACTAA AAAAAATCAA AAATTTTCTA   
  
  
- ATTTTATAAT TATACAAACC TCTATTTTGA AATTTTTATC TTTCTGTGAT TAAATCAAAA ACAAAACTTT   
  
  
- TAAATCTACG TTGAAAACAA TTACGTTCTT TGTTCTTGAA CTATACATAA AATTCACTCA TTGTTTACTA   
  
  
- AAACAGATAT CAATATACAT ATAATAGATT AAAACTCATT ATTCTTAGTA ATTATATTTA TAAAACTTAT   
  
  
- TTTAATCTAT CTATTTGATA TTTATATCTC AATTCATATG AATACAAACC AATAGAAGCT TATAATGGGC   
  
  
- AAGCCTATAT AATAGACTTG ACCACTTTAT TCATTATACA AAACAACAAA ATTAATCTAT TAAAAATCTG   
  
  
- GCTCGAACAC TTATATATGA TCTGTTTGTA AATATAAAGC TCAGACGTGA ATATAAGATA TTCTCGAACT   
  
  
- ATATAATCTA AACTTGTGAT TGGACAATTA TATCAAACGG CCACTAAAAA AAAGTTTTAA AACTAAGAAT   
  
  
- CTATACATAT ACCTCATTTT GATTAAAAAT GTCTACGGGT AAAAAAATTA ACTGTGAATA CATTAATTGA   
  
  
- CTTAAGTATT TTGTTCCAAA AAATTTTTTT AAATTGAGTA TAGTTACTTT GTTTCTGCTC TTGCTTTCGT   
  
  
- GTTAAGATAC CTTTACCTTT TACTTCAGTG AATACCTCTA AGTTATCATT CGTTTAGCTC TCGTCTTTTA   
  
  
- GATTAGAGGA AAGCAGTAAT ATGTTAGTTA TAACGGATAA ACCAAAATCA CTAAACAAAG TCGGCGTTTT   
  
  
- GAATTAAAAG AAACCACGTA TACTTTAGAA TTTTTCTTAA TTTTATTCTA TATTATGCAA TTGAGAAGTT   
  
  
- GTTGTACTAT ATAAATTCTA TGGTTATAAA CATAAGTAGT ATATTTTTAA CATCACAACG TTTTATAATT   
  
  
- TTAATAAAGT ATTTATTTGT AATAATATTC TTGACTGAGG CGCCTAATAG TATACCATAT CTAATGTCTC   
  
  
- ACCCACCCAA ATTTATTAAA GGGGCTGTGT AATATGAAAA TCTTCTCGAT AATCGAGGTT TAATAAATTT   
  
  
- GTTACAAGAT TTCTCGTTTT TTTAGTTACA AAACCTAAAA CTAGGCTGGC TCTCCTGAAC AGGCTGGTAA   
  
  
- GGTATAATTT TACAAGGATC AAGACTAATC GATTATGGT

+     Unnamed\_\_1

| Site Name | Organism | Position | Strand | Matrix score. | sequence | function |
| --- | --- | --- | --- | --- | --- | --- |
| Unnamed\_\_1 | Zea mays | 618 | + | 5 | CGTGG |  |

>PlantCARE\_4926   
+ TAAAGTTGTC GTGATTTCTT GCCCTTTATT TTTTATTTAT ATTAACATAT ATTTTTAAAA AAAAATTAAA   
  
  
+ GACGTTCCTA ACGGAAACTC ATGTATTTTT TGATTATGAA TATGAATATC CCCTCTATAT TAATCATGGA   
  
  
+ GCATTACAAC ATGTTTTCGT AGCCATATGT CATCACGAGA ATGATTTTTA GAATTGTTAG AAAAATAAAT   
  
  
+ TGATTCATAT AAACATATAC TATGTTTTTT ATTAAACTAA CTATCAAATT AATTAATAGT GTACAAAAAA   
  
  
+ ATATTTTTTT CTTTCCTTAA ATAAAAACTA CGGAATTACC TAATATGGCT AACATATATA TGACAATTAA   
  
  
+ TGATTATGAA TAATACATAT TTGATAAAAA AATTTCTAAC CTCTCTCTTT TTTGTTTAAT TTTATATTAT   
  
  
+ TAAAGGAAAT TTAACAATCA CATTAATCAT ATAATAAAAA CAATTAGATT TTTTCTTATA TGTTATATTT   
  
  
+ TGAATTTTTA AAAACGACTA TAAATTACTA AAAATGATAA GAGTCCCACA TTAAAAAATT TGTGATCAAC   
  
  
+ CGTTTAACTT TTTTTTTAGT TCAAGCAAGA TACAAATGAT CATATATCTG ATATAGACGT GGGCGTTCGG   
  
  
+ ATACACGTTC GGGTTTGTAT CAGATATTTC AGTATAAAGG TATAGAACCC GTTCGGGTAT TTCTACACTC   
  
  
+ CGAGTCGGGT TCGGGTTCGG ATATTTTGGA TCGGGTTCGA ATATTTAAAT TTTGAAGAAA AAAAGAAATT   
  
  
+ ATTCACTGTT TAAGTTTTTT ATATTTAAAT ATATCTTAAC TTAACTGATT TTTTTTAGTT TTTAAAAGAT   
  
  
+ TAAAATATTA ATATGTTTGG AGATAAAACT TTAAAAATAG AAAGACACTA ATTTAGTTTT TGTTTTGAAA   
  
  
+ ATTTAGATGC AACTTTTGTT AATGCAAGAA ACAAGAACTT GATATGTATT TTAAGTGAGT AACAAATGAT   
  
  
+ TTTGTCTATA GTTATATGTA TATTATCTAA TTTTGAGTAA TAAGAATCAT TAATATAAAT ATTTTGAATA   
  
  
+ AAATTAGATA GATAAACTAT AAATATAGAG TTAAGTATAC TTATGTTTGG TTATCTTCGA ATATTACCCG   
  
  
+ TTCGGATATA TTATCTGAAC TGGTGAAATA AGTAATATGT TTTGTTGTTT TAATTAGATA ATTTTTAGAC   
  
  
+ CGAGCTTGTG AATATATACT AGACAAACAT TTATATTTCG AGTCTGCACT TATATTCTAT AAGAGCTTGA   
  
  
+ TATATTAGAT TTGAACACTA ACCTGTTAAT ATAGTTTGCC GGTGATTTTT TTTCAAAATT TTGATTCTTA   
  
  
+ GATATGTATA TGGAGTAAAA CTAATTTTTA CAGATGCCCA TTTTTTTAAT TGACACTTAT GTAATTAACT   
  
  
+ GAATTCATAA AACAAGGTTT TTTAAAAAAA TTTAACTCAT ATCAATGAAA CAAAGACGAG AACGAAAGCA   
  
  
+ CAATTCTATG GAAATGGAAA ATGAAGTCAC TTATGGAGAT TCAATAGTAA GCAAATCGAG AGCAGAAAAT   
  
  
+ CTAATCTCCT TTCGTCATTA TACAATCAAT ATTGCCTATT TGGTTTTAGT GATTTGTTTC AGCCGCAAAA   
  
  
+ CTTAATTTTC TTTGGTGCAT ATGAAATCTT AAAAAGAATT AAAATAAGAT ATAATACGTT AACTCTTCAA   
  
  
+ CAACATGATA TATTTAAGAT ACCAATATTT GTATTCATCA TATAAAAATT GTAGTGTTGC AAAATATTAA   
  
  
+ AATTATTTCA TAAATAAACA TTATTATAAG AACTGACTCC GCGGATTATC ATATGGTATA GATTACAGAG   
  
  
+ TGGGTGGGTT TAAATAATTT CCCCGACACA TTATACTTTT AGAAGAGCTA TTAGCTCCAA ATTATTTAAA   
  
  
+ CAATGTTCTA AAGAGCAAAA AAATCAATGT TTTGGATTTT GATCCGACCG AGAGGACTTG TCCGACCATT   
  
  
+ CCATATTAAA ATGTTCCTAG TTCTGATTAG CTAATACCA  

- ATTTCAACAG CACTAAAGAA CGGGAAATAA AAAATAAATA TAATTGTATA TAAAAATTTT TTTTTAATTT   
  
  
- CTGCAAGGAT TGCCTTTGAG TACATAAAAA ACTAATACTT ATACTTATAG GGGAGATATA ATTAGTACCT   
  
  
- CGTAATGTTG TACAAAAGCA TCGGTATACA GTAGTGCTCT TACTAAAAAT CTTAACAATC TTTTTATTTA   
  
  
- ACTAAGTATA TTTGTATATG ATACAAAAAA TAATTTGATT GATAGTTTAA TTAATTATCA CATGTTTTTT   
  
  
- TATAAAAAAA GAAAGGAATT TATTTTTGAT GCCTTAATGG ATTATACCGA TTGTATATAT ACTGTTAATT   
  
  
- ACTAATACTT ATTATGTATA AACTATTTTT TTAAAGATTG GAGAGAGAAA AAACAAATTA AAATATAATA   
  
  
- ATTTCCTTTA AATTGTTAGT GTAATTAGTA TATTATTTTT GTTAATCTAA AAAAGAATAT ACAATATAAA   
  
  
- ACTTAAAAAT TTTTGCTGAT ATTTAATGAT TTTTACTATT CTCAGGGTGT AATTTTTTAA ACACTAGTTG   
  
  
- GCAAATTGAA AAAAAAATCA AGTTCGTTCT ATGTTTACTA GTATATAGAC TATATCTGCA CCCGCAAGCC   
  
  
- TATGTGCAAG CCCAAACATA GTCTATAAAG TCATATTTCC ATATCTTGGG CAAGCCCATA AAGATGTGAG   
  
  
- GCTCAGCCCA AGCCCAAGCC TATAAAACCT AGCCCAAGCT TATAAATTTA AAACTTCTTT TTTTCTTTAA   
  
  
- TAAGTGACAA ATTCAAAAAA TATAAATTTA TATAGAATTG AATTGACTAA AAAAAATCAA AAATTTTCTA   
  
  
- ATTTTATAAT TATACAAACC TCTATTTTGA AATTTTTATC TTTCTGTGAT TAAATCAAAA ACAAAACTTT   
  
  
- TAAATCTACG TTGAAAACAA TTACGTTCTT TGTTCTTGAA CTATACATAA AATTCACTCA TTGTTTACTA   
  
  
- AAACAGATAT CAATATACAT ATAATAGATT AAAACTCATT ATTCTTAGTA ATTATATTTA TAAAACTTAT   
  
  
- TTTAATCTAT CTATTTGATA TTTATATCTC AATTCATATG AATACAAACC AATAGAAGCT TATAATGGGC   
  
  
- AAGCCTATAT AATAGACTTG ACCACTTTAT TCATTATACA AAACAACAAA ATTAATCTAT TAAAAATCTG   
  
  
- GCTCGAACAC TTATATATGA TCTGTTTGTA AATATAAAGC TCAGACGTGA ATATAAGATA TTCTCGAACT   
  
  
- ATATAATCTA AACTTGTGAT TGGACAATTA TATCAAACGG CCACTAAAAA AAAGTTTTAA AACTAAGAAT   
  
  
- CTATACATAT ACCTCATTTT GATTAAAAAT GTCTACGGGT AAAAAAATTA ACTGTGAATA CATTAATTGA   
  
  
- CTTAAGTATT TTGTTCCAAA AAATTTTTTT AAATTGAGTA TAGTTACTTT GTTTCTGCTC TTGCTTTCGT   
  
  
- GTTAAGATAC CTTTACCTTT TACTTCAGTG AATACCTCTA AGTTATCATT CGTTTAGCTC TCGTCTTTTA   
  
  
- GATTAGAGGA AAGCAGTAAT ATGTTAGTTA TAACGGATAA ACCAAAATCA CTAAACAAAG TCGGCGTTTT   
  
  
- GAATTAAAAG AAACCACGTA TACTTTAGAA TTTTTCTTAA TTTTATTCTA TATTATGCAA TTGAGAAGTT   
  
  
- GTTGTACTAT ATAAATTCTA TGGTTATAAA CATAAGTAGT ATATTTTTAA CATCACAACG TTTTATAATT   
  
  
- TTAATAAAGT ATTTATTTGT AATAATATTC TTGACTGAGG CGCCTAATAG TATACCATAT CTAATGTCTC   
  
  
- ACCCACCCAA ATTTATTAAA GGGGCTGTGT AATATGAAAA TCTTCTCGAT AATCGAGGTT TAATAAATTT   
  
  
- GTTACAAGAT TTCTCGTTTT TTTAGTTACA AAACCTAAAA CTAGGCTGGC TCTCCTGAAC AGGCTGGTAA   
  
  
- GGTATAATTT TACAAGGATC AAGACTAATC GATTATGGT

+     Unnamed\_\_4

| Site Name | Organism | Position | Strand | Matrix score. | sequence | function |
| --- | --- | --- | --- | --- | --- | --- |
| Unnamed\_\_4 | Petroselinum hortense | 138 | - | 4 | CTCC |  |
| Unnamed\_\_4 | Petroselinum hortense | 1505 | - | 4 | CTCC |  |
| Unnamed\_\_4 | Petroselinum hortense | 859 | - | 4 | CTCC |  |
| Unnamed\_\_4 | Petroselinum hortense | 1787 | + | 4 | CTCC |  |
| Unnamed\_\_4 | Petroselinum hortense | 698 | + | 4 | CTCC |  |
| Unnamed\_\_4 | Petroselinum hortense | 1546 | + | 4 | CTCC |  |
| Unnamed\_\_4 | Petroselinum hortense | 1342 | - | 4 | CTCC |  |
| Unnamed\_\_4 | Petroselinum hortense | 1875 | + | 4 | CTCC |  |

>PlantCARE\_4926   
+ TAAAGTTGTC GTGATTTCTT GCCCTTTATT TTTTATTTAT ATTAACATAT ATTTTTAAAA AAAAATTAAA   
  
  
+ GACGTTCCTA ACGGAAACTC ATGTATTTTT TGATTATGAA TATGAATATC CCCTCTATAT TAATCATGGA   
  
  
+ GCATTACAAC ATGTTTTCGT AGCCATATGT CATCACGAGA ATGATTTTTA GAATTGTTAG AAAAATAAAT   
  
  
+ TGATTCATAT AAACATATAC TATGTTTTTT ATTAAACTAA CTATCAAATT AATTAATAGT GTACAAAAAA   
  
  
+ ATATTTTTTT CTTTCCTTAA ATAAAAACTA CGGAATTACC TAATATGGCT AACATATATA TGACAATTAA   
  
  
+ TGATTATGAA TAATACATAT TTGATAAAAA AATTTCTAAC CTCTCTCTTT TTTGTTTAAT TTTATATTAT   
  
  
+ TAAAGGAAAT TTAACAATCA CATTAATCAT ATAATAAAAA CAATTAGATT TTTTCTTATA TGTTATATTT   
  
  
+ TGAATTTTTA AAAACGACTA TAAATTACTA AAAATGATAA GAGTCCCACA TTAAAAAATT TGTGATCAAC   
  
  
+ CGTTTAACTT TTTTTTTAGT TCAAGCAAGA TACAAATGAT CATATATCTG ATATAGACGT GGGCGTTCGG   
  
  
+ ATACACGTTC GGGTTTGTAT CAGATATTTC AGTATAAAGG TATAGAACCC GTTCGGGTAT TTCTACACTC   
  
  
+ CGAGTCGGGT TCGGGTTCGG ATATTTTGGA TCGGGTTCGA ATATTTAAAT TTTGAAGAAA AAAAGAAATT   
  
  
+ ATTCACTGTT TAAGTTTTTT ATATTTAAAT ATATCTTAAC TTAACTGATT TTTTTTAGTT TTTAAAAGAT   
  
  
+ TAAAATATTA ATATGTTTGG AGATAAAACT TTAAAAATAG AAAGACACTA ATTTAGTTTT TGTTTTGAAA   
  
  
+ ATTTAGATGC AACTTTTGTT AATGCAAGAA ACAAGAACTT GATATGTATT TTAAGTGAGT AACAAATGAT   
  
  
+ TTTGTCTATA GTTATATGTA TATTATCTAA TTTTGAGTAA TAAGAATCAT TAATATAAAT ATTTTGAATA   
  
  
+ AAATTAGATA GATAAACTAT AAATATAGAG TTAAGTATAC TTATGTTTGG TTATCTTCGA ATATTACCCG   
  
  
+ TTCGGATATA TTATCTGAAC TGGTGAAATA AGTAATATGT TTTGTTGTTT TAATTAGATA ATTTTTAGAC   
  
  
+ CGAGCTTGTG AATATATACT AGACAAACAT TTATATTTCG AGTCTGCACT TATATTCTAT AAGAGCTTGA   
  
  
+ TATATTAGAT TTGAACACTA ACCTGTTAAT ATAGTTTGCC GGTGATTTTT TTTCAAAATT TTGATTCTTA   
  
  
+ GATATGTATA TGGAGTAAAA CTAATTTTTA CAGATGCCCA TTTTTTTAAT TGACACTTAT GTAATTAACT   
  
  
+ GAATTCATAA AACAAGGTTT TTTAAAAAAA TTTAACTCAT ATCAATGAAA CAAAGACGAG AACGAAAGCA   
  
  
+ CAATTCTATG GAAATGGAAA ATGAAGTCAC TTATGGAGAT TCAATAGTAA GCAAATCGAG AGCAGAAAAT   
  
  
+ CTAATCTCCT TTCGTCATTA TACAATCAAT ATTGCCTATT TGGTTTTAGT GATTTGTTTC AGCCGCAAAA   
  
  
+ CTTAATTTTC TTTGGTGCAT ATGAAATCTT AAAAAGAATT AAAATAAGAT ATAATACGTT AACTCTTCAA   
  
  
+ CAACATGATA TATTTAAGAT ACCAATATTT GTATTCATCA TATAAAAATT GTAGTGTTGC AAAATATTAA   
  
  
+ AATTATTTCA TAAATAAACA TTATTATAAG AACTGACTCC GCGGATTATC ATATGGTATA GATTACAGAG   
  
  
+ TGGGTGGGTT TAAATAATTT CCCCGACACA TTATACTTTT AGAAGAGCTA TTAGCTCCAA ATTATTTAAA   
  
  
+ CAATGTTCTA AAGAGCAAAA AAATCAATGT TTTGGATTTT GATCCGACCG AGAGGACTTG TCCGACCATT   
  
  
+ CCATATTAAA ATGTTCCTAG TTCTGATTAG CTAATACCA  

- ATTTCAACAG CACTAAAGAA CGGGAAATAA AAAATAAATA TAATTGTATA TAAAAATTTT TTTTTAATTT   
  
  
- CTGCAAGGAT TGCCTTTGAG TACATAAAAA ACTAATACTT ATACTTATAG GGGAGATATA ATTAGTACCT   
  
  
- CGTAATGTTG TACAAAAGCA TCGGTATACA GTAGTGCTCT TACTAAAAAT CTTAACAATC TTTTTATTTA   
  
  
- ACTAAGTATA TTTGTATATG ATACAAAAAA TAATTTGATT GATAGTTTAA TTAATTATCA CATGTTTTTT   
  
  
- TATAAAAAAA GAAAGGAATT TATTTTTGAT GCCTTAATGG ATTATACCGA TTGTATATAT ACTGTTAATT   
  
  
- ACTAATACTT ATTATGTATA AACTATTTTT TTAAAGATTG GAGAGAGAAA AAACAAATTA AAATATAATA   
  
  
- ATTTCCTTTA AATTGTTAGT GTAATTAGTA TATTATTTTT GTTAATCTAA AAAAGAATAT ACAATATAAA   
  
  
- ACTTAAAAAT TTTTGCTGAT ATTTAATGAT TTTTACTATT CTCAGGGTGT AATTTTTTAA ACACTAGTTG   
  
  
- GCAAATTGAA AAAAAAATCA AGTTCGTTCT ATGTTTACTA GTATATAGAC TATATCTGCA CCCGCAAGCC   
  
  
- TATGTGCAAG CCCAAACATA GTCTATAAAG TCATATTTCC ATATCTTGGG CAAGCCCATA AAGATGTGAG   
  
  
- GCTCAGCCCA AGCCCAAGCC TATAAAACCT AGCCCAAGCT TATAAATTTA AAACTTCTTT TTTTCTTTAA   
  
  
- TAAGTGACAA ATTCAAAAAA TATAAATTTA TATAGAATTG AATTGACTAA AAAAAATCAA AAATTTTCTA   
  
  
- ATTTTATAAT TATACAAACC TCTATTTTGA AATTTTTATC TTTCTGTGAT TAAATCAAAA ACAAAACTTT   
  
  
- TAAATCTACG TTGAAAACAA TTACGTTCTT TGTTCTTGAA CTATACATAA AATTCACTCA TTGTTTACTA   
  
  
- AAACAGATAT CAATATACAT ATAATAGATT AAAACTCATT ATTCTTAGTA ATTATATTTA TAAAACTTAT   
  
  
- TTTAATCTAT CTATTTGATA TTTATATCTC AATTCATATG AATACAAACC AATAGAAGCT TATAATGGGC   
  
  
- AAGCCTATAT AATAGACTTG ACCACTTTAT TCATTATACA AAACAACAAA ATTAATCTAT TAAAAATCTG   
  
  
- GCTCGAACAC TTATATATGA TCTGTTTGTA AATATAAAGC TCAGACGTGA ATATAAGATA TTCTCGAACT   
  
  
- ATATAATCTA AACTTGTGAT TGGACAATTA TATCAAACGG CCACTAAAAA AAAGTTTTAA AACTAAGAAT   
  
  
- CTATACATAT ACCTCATTTT GATTAAAAAT GTCTACGGGT AAAAAAATTA ACTGTGAATA CATTAATTGA   
  
  
- CTTAAGTATT TTGTTCCAAA AAATTTTTTT AAATTGAGTA TAGTTACTTT GTTTCTGCTC TTGCTTTCGT   
  
  
- GTTAAGATAC CTTTACCTTT TACTTCAGTG AATACCTCTA AGTTATCATT CGTTTAGCTC TCGTCTTTTA   
  
  
- GATTAGAGGA AAGCAGTAAT ATGTTAGTTA TAACGGATAA ACCAAAATCA CTAAACAAAG TCGGCGTTTT   
  
  
- GAATTAAAAG AAACCACGTA TACTTTAGAA TTTTTCTTAA TTTTATTCTA TATTATGCAA TTGAGAAGTT   
  
  
- GTTGTACTAT ATAAATTCTA TGGTTATAAA CATAAGTAGT ATATTTTTAA CATCACAACG TTTTATAATT   
  
  
- TTAATAAAGT ATTTATTTGT AATAATATTC TTGACTGAGG CGCCTAATAG TATACCATAT CTAATGTCTC   
  
  
- ACCCACCCAA ATTTATTAAA GGGGCTGTGT AATATGAAAA TCTTCTCGAT AATCGAGGTT TAATAAATTT   
  
  
- GTTACAAGAT TTCTCGTTTT TTTAGTTACA AAACCTAAAA CTAGGCTGGC TCTCCTGAAC AGGCTGGTAA   
  
  
- GGTATAATTT TACAAGGATC AAGACTAATC GATTATGGT

+     Unnamed\_\_6

| Site Name | Organism | Position | Strand | Matrix score. | sequence | function |
| --- | --- | --- | --- | --- | --- | --- |
| Unnamed\_\_6 | Zea mays | 1034 | + | 10 | taTAAATATct |  |
| Unnamed\_\_6 | Zea mays | 1330 | - | 10 | taTAAATATct |  |

>PlantCARE\_4926   
+ TAAAGTTGTC GTGATTTCTT GCCCTTTATT TTTTATTTAT ATTAACATAT ATTTTTAAAA AAAAATTAAA   
  
  
+ GACGTTCCTA ACGGAAACTC ATGTATTTTT TGATTATGAA TATGAATATC CCCTCTATAT TAATCATGGA   
  
  
+ GCATTACAAC ATGTTTTCGT AGCCATATGT CATCACGAGA ATGATTTTTA GAATTGTTAG AAAAATAAAT   
  
  
+ TGATTCATAT AAACATATAC TATGTTTTTT ATTAAACTAA CTATCAAATT AATTAATAGT GTACAAAAAA   
  
  
+ ATATTTTTTT CTTTCCTTAA ATAAAAACTA CGGAATTACC TAATATGGCT AACATATATA TGACAATTAA   
  
  
+ TGATTATGAA TAATACATAT TTGATAAAAA AATTTCTAAC CTCTCTCTTT TTTGTTTAAT TTTATATTAT   
  
  
+ TAAAGGAAAT TTAACAATCA CATTAATCAT ATAATAAAAA CAATTAGATT TTTTCTTATA TGTTATATTT   
  
  
+ TGAATTTTTA AAAACGACTA TAAATTACTA AAAATGATAA GAGTCCCACA TTAAAAAATT TGTGATCAAC   
  
  
+ CGTTTAACTT TTTTTTTAGT TCAAGCAAGA TACAAATGAT CATATATCTG ATATAGACGT GGGCGTTCGG   
  
  
+ ATACACGTTC GGGTTTGTAT CAGATATTTC AGTATAAAGG TATAGAACCC GTTCGGGTAT TTCTACACTC   
  
  
+ CGAGTCGGGT TCGGGTTCGG ATATTTTGGA TCGGGTTCGA ATATTTAAAT TTTGAAGAAA AAAAGAAATT   
  
  
+ ATTCACTGTT TAAGTTTTTT ATATTTAAAT ATATCTTAAC TTAACTGATT TTTTTTAGTT TTTAAAAGAT   
  
  
+ TAAAATATTA ATATGTTTGG AGATAAAACT TTAAAAATAG AAAGACACTA ATTTAGTTTT TGTTTTGAAA   
  
  
+ ATTTAGATGC AACTTTTGTT AATGCAAGAA ACAAGAACTT GATATGTATT TTAAGTGAGT AACAAATGAT   
  
  
+ TTTGTCTATA GTTATATGTA TATTATCTAA TTTTGAGTAA TAAGAATCAT TAATATAAAT ATTTTGAATA   
  
  
+ AAATTAGATA GATAAACTAT AAATATAGAG TTAAGTATAC TTATGTTTGG TTATCTTCGA ATATTACCCG   
  
  
+ TTCGGATATA TTATCTGAAC TGGTGAAATA AGTAATATGT TTTGTTGTTT TAATTAGATA ATTTTTAGAC   
  
  
+ CGAGCTTGTG AATATATACT AGACAAACAT TTATATTTCG AGTCTGCACT TATATTCTAT AAGAGCTTGA   
  
  
+ TATATTAGAT TTGAACACTA ACCTGTTAAT ATAGTTTGCC GGTGATTTTT TTTCAAAATT TTGATTCTTA   
  
  
+ GATATGTATA TGGAGTAAAA CTAATTTTTA CAGATGCCCA TTTTTTTAAT TGACACTTAT GTAATTAACT   
  
  
+ GAATTCATAA AACAAGGTTT TTTAAAAAAA TTTAACTCAT ATCAATGAAA CAAAGACGAG AACGAAAGCA   
  
  
+ CAATTCTATG GAAATGGAAA ATGAAGTCAC TTATGGAGAT TCAATAGTAA GCAAATCGAG AGCAGAAAAT   
  
  
+ CTAATCTCCT TTCGTCATTA TACAATCAAT ATTGCCTATT TGGTTTTAGT GATTTGTTTC AGCCGCAAAA   
  
  
+ CTTAATTTTC TTTGGTGCAT ATGAAATCTT AAAAAGAATT AAAATAAGAT ATAATACGTT AACTCTTCAA   
  
  
+ CAACATGATA TATTTAAGAT ACCAATATTT GTATTCATCA TATAAAAATT GTAGTGTTGC AAAATATTAA   
  
  
+ AATTATTTCA TAAATAAACA TTATTATAAG AACTGACTCC GCGGATTATC ATATGGTATA GATTACAGAG   
  
  
+ TGGGTGGGTT TAAATAATTT CCCCGACACA TTATACTTTT AGAAGAGCTA TTAGCTCCAA ATTATTTAAA   
  
  
+ CAATGTTCTA AAGAGCAAAA AAATCAATGT TTTGGATTTT GATCCGACCG AGAGGACTTG TCCGACCATT   
  
  
+ CCATATTAAA ATGTTCCTAG TTCTGATTAG CTAATACCA  

- ATTTCAACAG CACTAAAGAA CGGGAAATAA AAAATAAATA TAATTGTATA TAAAAATTTT TTTTTAATTT   
  
  
- CTGCAAGGAT TGCCTTTGAG TACATAAAAA ACTAATACTT ATACTTATAG GGGAGATATA ATTAGTACCT   
  
  
- CGTAATGTTG TACAAAAGCA TCGGTATACA GTAGTGCTCT TACTAAAAAT CTTAACAATC TTTTTATTTA   
  
  
- ACTAAGTATA TTTGTATATG ATACAAAAAA TAATTTGATT GATAGTTTAA TTAATTATCA CATGTTTTTT   
  
  
- TATAAAAAAA GAAAGGAATT TATTTTTGAT GCCTTAATGG ATTATACCGA TTGTATATAT ACTGTTAATT   
  
  
- ACTAATACTT ATTATGTATA AACTATTTTT TTAAAGATTG GAGAGAGAAA AAACAAATTA AAATATAATA   
  
  
- ATTTCCTTTA AATTGTTAGT GTAATTAGTA TATTATTTTT GTTAATCTAA AAAAGAATAT ACAATATAAA   
  
  
- ACTTAAAAAT TTTTGCTGAT ATTTAATGAT TTTTACTATT CTCAGGGTGT AATTTTTTAA ACACTAGTTG   
  
  
- GCAAATTGAA AAAAAAATCA AGTTCGTTCT ATGTTTACTA GTATATAGAC TATATCTGCA CCCGCAAGCC   
  
  
- TATGTGCAAG CCCAAACATA GTCTATAAAG TCATATTTCC ATATCTTGGG CAAGCCCATA AAGATGTGAG   
  
  
- GCTCAGCCCA AGCCCAAGCC TATAAAACCT AGCCCAAGCT TATAAATTTA AAACTTCTTT TTTTCTTTAA   
  
  
- TAAGTGACAA ATTCAAAAAA TATAAATTTA TATAGAATTG AATTGACTAA AAAAAATCAA AAATTTTCTA   
  
  
- ATTTTATAAT TATACAAACC TCTATTTTGA AATTTTTATC TTTCTGTGAT TAAATCAAAA ACAAAACTTT   
  
  
- TAAATCTACG TTGAAAACAA TTACGTTCTT TGTTCTTGAA CTATACATAA AATTCACTCA TTGTTTACTA   
  
  
- AAACAGATAT CAATATACAT ATAATAGATT AAAACTCATT ATTCTTAGTA ATTATATTTA TAAAACTTAT   
  
  
- TTTAATCTAT CTATTTGATA TTTATATCTC AATTCATATG AATACAAACC AATAGAAGCT TATAATGGGC   
  
  
- AAGCCTATAT AATAGACTTG ACCACTTTAT TCATTATACA AAACAACAAA ATTAATCTAT TAAAAATCTG   
  
  
- GCTCGAACAC TTATATATGA TCTGTTTGTA AATATAAAGC TCAGACGTGA ATATAAGATA TTCTCGAACT   
  
  
- ATATAATCTA AACTTGTGAT TGGACAATTA TATCAAACGG CCACTAAAAA AAAGTTTTAA AACTAAGAAT   
  
  
- CTATACATAT ACCTCATTTT GATTAAAAAT GTCTACGGGT AAAAAAATTA ACTGTGAATA CATTAATTGA   
  
  
- CTTAAGTATT TTGTTCCAAA AAATTTTTTT AAATTGAGTA TAGTTACTTT GTTTCTGCTC TTGCTTTCGT   
  
  
- GTTAAGATAC CTTTACCTTT TACTTCAGTG AATACCTCTA AGTTATCATT CGTTTAGCTC TCGTCTTTTA   
  
  
- GATTAGAGGA AAGCAGTAAT ATGTTAGTTA TAACGGATAA ACCAAAATCA CTAAACAAAG TCGGCGTTTT   
  
  
- GAATTAAAAG AAACCACGTA TACTTTAGAA TTTTTCTTAA TTTTATTCTA TATTATGCAA TTGAGAAGTT   
  
  
- GTTGTACTAT ATAAATTCTA TGGTTATAAA CATAAGTAGT ATATTTTTAA CATCACAACG TTTTATAATT   
  
  
- TTAATAAAGT ATTTATTTGT AATAATATTC TTGACTGAGG CGCCTAATAG TATACCATAT CTAATGTCTC   
  
  
- ACCCACCCAA ATTTATTAAA GGGGCTGTGT AATATGAAAA TCTTCTCGAT AATCGAGGTT TAATAAATTT   
  
  
- GTTACAAGAT TTCTCGTTTT TTTAGTTACA AAACCTAAAA CTAGGCTGGC TCTCCTGAAC AGGCTGGTAA   
  
  
- GGTATAATTT TACAAGGATC AAGACTAATC GATTATGGT

+     WUN-motif

| Site Name | Organism | Position | Strand | Matrix score. | sequence | function |
| --- | --- | --- | --- | --- | --- | --- |
| WUN-motif | Brassica oleracea | 424 | - | 9 | AAATTTCCT | wound-responsive element |
| WUN-motif | Nicotiana glutinosa | 512 | + | 9 | AAATTACTA |  |

>PlantCARE\_4926   
+ TAAAGTTGTC GTGATTTCTT GCCCTTTATT TTTTATTTAT ATTAACATAT ATTTTTAAAA AAAAATTAAA   
  
  
+ GACGTTCCTA ACGGAAACTC ATGTATTTTT TGATTATGAA TATGAATATC CCCTCTATAT TAATCATGGA   
  
  
+ GCATTACAAC ATGTTTTCGT AGCCATATGT CATCACGAGA ATGATTTTTA GAATTGTTAG AAAAATAAAT   
  
  
+ TGATTCATAT AAACATATAC TATGTTTTTT ATTAAACTAA CTATCAAATT AATTAATAGT GTACAAAAAA   
  
  
+ ATATTTTTTT CTTTCCTTAA ATAAAAACTA CGGAATTACC TAATATGGCT AACATATATA TGACAATTAA   
  
  
+ TGATTATGAA TAATACATAT TTGATAAAAA AATTTCTAAC CTCTCTCTTT TTTGTTTAAT TTTATATTAT   
  
  
+ TAAAGGAAAT TTAACAATCA CATTAATCAT ATAATAAAAA CAATTAGATT TTTTCTTATA TGTTATATTT   
  
  
+ TGAATTTTTA AAAACGACTA TAAATTACTA AAAATGATAA GAGTCCCACA TTAAAAAATT TGTGATCAAC   
  
  
+ CGTTTAACTT TTTTTTTAGT TCAAGCAAGA TACAAATGAT CATATATCTG ATATAGACGT GGGCGTTCGG   
  
  
+ ATACACGTTC GGGTTTGTAT CAGATATTTC AGTATAAAGG TATAGAACCC GTTCGGGTAT TTCTACACTC   
  
  
+ CGAGTCGGGT TCGGGTTCGG ATATTTTGGA TCGGGTTCGA ATATTTAAAT TTTGAAGAAA AAAAGAAATT   
  
  
+ ATTCACTGTT TAAGTTTTTT ATATTTAAAT ATATCTTAAC TTAACTGATT TTTTTTAGTT TTTAAAAGAT   
  
  
+ TAAAATATTA ATATGTTTGG AGATAAAACT TTAAAAATAG AAAGACACTA ATTTAGTTTT TGTTTTGAAA   
  
  
+ ATTTAGATGC AACTTTTGTT AATGCAAGAA ACAAGAACTT GATATGTATT TTAAGTGAGT AACAAATGAT   
  
  
+ TTTGTCTATA GTTATATGTA TATTATCTAA TTTTGAGTAA TAAGAATCAT TAATATAAAT ATTTTGAATA   
  
  
+ AAATTAGATA GATAAACTAT AAATATAGAG TTAAGTATAC TTATGTTTGG TTATCTTCGA ATATTACCCG   
  
  
+ TTCGGATATA TTATCTGAAC TGGTGAAATA AGTAATATGT TTTGTTGTTT TAATTAGATA ATTTTTAGAC   
  
  
+ CGAGCTTGTG AATATATACT AGACAAACAT TTATATTTCG AGTCTGCACT TATATTCTAT AAGAGCTTGA   
  
  
+ TATATTAGAT TTGAACACTA ACCTGTTAAT ATAGTTTGCC GGTGATTTTT TTTCAAAATT TTGATTCTTA   
  
  
+ GATATGTATA TGGAGTAAAA CTAATTTTTA CAGATGCCCA TTTTTTTAAT TGACACTTAT GTAATTAACT   
  
  
+ GAATTCATAA AACAAGGTTT TTTAAAAAAA TTTAACTCAT ATCAATGAAA CAAAGACGAG AACGAAAGCA   
  
  
+ CAATTCTATG GAAATGGAAA ATGAAGTCAC TTATGGAGAT TCAATAGTAA GCAAATCGAG AGCAGAAAAT   
  
  
+ CTAATCTCCT TTCGTCATTA TACAATCAAT ATTGCCTATT TGGTTTTAGT GATTTGTTTC AGCCGCAAAA   
  
  
+ CTTAATTTTC TTTGGTGCAT ATGAAATCTT AAAAAGAATT AAAATAAGAT ATAATACGTT AACTCTTCAA   
  
  
+ CAACATGATA TATTTAAGAT ACCAATATTT GTATTCATCA TATAAAAATT GTAGTGTTGC AAAATATTAA   
  
  
+ AATTATTTCA TAAATAAACA TTATTATAAG AACTGACTCC GCGGATTATC ATATGGTATA GATTACAGAG   
  
  
+ TGGGTGGGTT TAAATAATTT CCCCGACACA TTATACTTTT AGAAGAGCTA TTAGCTCCAA ATTATTTAAA   
  
  
+ CAATGTTCTA AAGAGCAAAA AAATCAATGT TTTGGATTTT GATCCGACCG AGAGGACTTG TCCGACCATT   
  
  
+ CCATATTAAA ATGTTCCTAG TTCTGATTAG CTAATACCA  

- ATTTCAACAG CACTAAAGAA CGGGAAATAA AAAATAAATA TAATTGTATA TAAAAATTTT TTTTTAATTT   
  
  
- CTGCAAGGAT TGCCTTTGAG TACATAAAAA ACTAATACTT ATACTTATAG GGGAGATATA ATTAGTACCT   
  
  
- CGTAATGTTG TACAAAAGCA TCGGTATACA GTAGTGCTCT TACTAAAAAT CTTAACAATC TTTTTATTTA   
  
  
- ACTAAGTATA TTTGTATATG ATACAAAAAA TAATTTGATT GATAGTTTAA TTAATTATCA CATGTTTTTT   
  
  
- TATAAAAAAA GAAAGGAATT TATTTTTGAT GCCTTAATGG ATTATACCGA TTGTATATAT ACTGTTAATT   
  
  
- ACTAATACTT ATTATGTATA AACTATTTTT TTAAAGATTG GAGAGAGAAA AAACAAATTA AAATATAATA   
  
  
- ATTTCCTTTA AATTGTTAGT GTAATTAGTA TATTATTTTT GTTAATCTAA AAAAGAATAT ACAATATAAA   
  
  
- ACTTAAAAAT TTTTGCTGAT ATTTAATGAT TTTTACTATT CTCAGGGTGT AATTTTTTAA ACACTAGTTG   
  
  
- GCAAATTGAA AAAAAAATCA AGTTCGTTCT ATGTTTACTA GTATATAGAC TATATCTGCA CCCGCAAGCC   
  
  
- TATGTGCAAG CCCAAACATA GTCTATAAAG TCATATTTCC ATATCTTGGG CAAGCCCATA AAGATGTGAG   
  
  
- GCTCAGCCCA AGCCCAAGCC TATAAAACCT AGCCCAAGCT TATAAATTTA AAACTTCTTT TTTTCTTTAA   
  
  
- TAAGTGACAA ATTCAAAAAA TATAAATTTA TATAGAATTG AATTGACTAA AAAAAATCAA AAATTTTCTA   
  
  
- ATTTTATAAT TATACAAACC TCTATTTTGA AATTTTTATC TTTCTGTGAT TAAATCAAAA ACAAAACTTT   
  
  
- TAAATCTACG TTGAAAACAA TTACGTTCTT TGTTCTTGAA CTATACATAA AATTCACTCA TTGTTTACTA   
  
  
- AAACAGATAT CAATATACAT ATAATAGATT AAAACTCATT ATTCTTAGTA ATTATATTTA TAAAACTTAT   
  
  
- TTTAATCTAT CTATTTGATA TTTATATCTC AATTCATATG AATACAAACC AATAGAAGCT TATAATGGGC   
  
  
- AAGCCTATAT AATAGACTTG ACCACTTTAT TCATTATACA AAACAACAAA ATTAATCTAT TAAAAATCTG   
  
  
- GCTCGAACAC TTATATATGA TCTGTTTGTA AATATAAAGC TCAGACGTGA ATATAAGATA TTCTCGAACT   
  
  
- ATATAATCTA AACTTGTGAT TGGACAATTA TATCAAACGG CCACTAAAAA AAAGTTTTAA AACTAAGAAT   
  
  
- CTATACATAT ACCTCATTTT GATTAAAAAT GTCTACGGGT AAAAAAATTA ACTGTGAATA CATTAATTGA   
  
  
- CTTAAGTATT TTGTTCCAAA AAATTTTTTT AAATTGAGTA TAGTTACTTT GTTTCTGCTC TTGCTTTCGT   
  
  
- GTTAAGATAC CTTTACCTTT TACTTCAGTG AATACCTCTA AGTTATCATT CGTTTAGCTC TCGTCTTTTA   
  
  
- GATTAGAGGA AAGCAGTAAT ATGTTAGTTA TAACGGATAA ACCAAAATCA CTAAACAAAG TCGGCGTTTT   
  
  
- GAATTAAAAG AAACCACGTA TACTTTAGAA TTTTTCTTAA TTTTATTCTA TATTATGCAA TTGAGAAGTT   
  
  
- GTTGTACTAT ATAAATTCTA TGGTTATAAA CATAAGTAGT ATATTTTTAA CATCACAACG TTTTATAATT   
  
  
- TTAATAAAGT ATTTATTTGT AATAATATTC TTGACTGAGG CGCCTAATAG TATACCATAT CTAATGTCTC   
  
  
- ACCCACCCAA ATTTATTAAA GGGGCTGTGT AATATGAAAA TCTTCTCGAT AATCGAGGTT TAATAAATTT   
  
  
- GTTACAAGAT TTCTCGTTTT TTTAGTTACA AAACCTAAAA CTAGGCTGGC TCTCCTGAAC AGGCTGGTAA   
  
  
- GGTATAATTT TACAAGGATC AAGACTAATC GATTATGGT

+     as-1

| Site Name | Organism | Position | Strand | Matrix score. | sequence | function |
| --- | --- | --- | --- | --- | --- | --- |
| as-1 | Arabidopsis thaliana | 1553 | - | 5 | TGACG |  |

>PlantCARE\_4926   
+ TAAAGTTGTC GTGATTTCTT GCCCTTTATT TTTTATTTAT ATTAACATAT ATTTTTAAAA AAAAATTAAA   
  
  
+ GACGTTCCTA ACGGAAACTC ATGTATTTTT TGATTATGAA TATGAATATC CCCTCTATAT TAATCATGGA   
  
  
+ GCATTACAAC ATGTTTTCGT AGCCATATGT CATCACGAGA ATGATTTTTA GAATTGTTAG AAAAATAAAT   
  
  
+ TGATTCATAT AAACATATAC TATGTTTTTT ATTAAACTAA CTATCAAATT AATTAATAGT GTACAAAAAA   
  
  
+ ATATTTTTTT CTTTCCTTAA ATAAAAACTA CGGAATTACC TAATATGGCT AACATATATA TGACAATTAA   
  
  
+ TGATTATGAA TAATACATAT TTGATAAAAA AATTTCTAAC CTCTCTCTTT TTTGTTTAAT TTTATATTAT   
  
  
+ TAAAGGAAAT TTAACAATCA CATTAATCAT ATAATAAAAA CAATTAGATT TTTTCTTATA TGTTATATTT   
  
  
+ TGAATTTTTA AAAACGACTA TAAATTACTA AAAATGATAA GAGTCCCACA TTAAAAAATT TGTGATCAAC   
  
  
+ CGTTTAACTT TTTTTTTAGT TCAAGCAAGA TACAAATGAT CATATATCTG ATATAGACGT GGGCGTTCGG   
  
  
+ ATACACGTTC GGGTTTGTAT CAGATATTTC AGTATAAAGG TATAGAACCC GTTCGGGTAT TTCTACACTC   
  
  
+ CGAGTCGGGT TCGGGTTCGG ATATTTTGGA TCGGGTTCGA ATATTTAAAT TTTGAAGAAA AAAAGAAATT   
  
  
+ ATTCACTGTT TAAGTTTTTT ATATTTAAAT ATATCTTAAC TTAACTGATT TTTTTTAGTT TTTAAAAGAT   
  
  
+ TAAAATATTA ATATGTTTGG AGATAAAACT TTAAAAATAG AAAGACACTA ATTTAGTTTT TGTTTTGAAA   
  
  
+ ATTTAGATGC AACTTTTGTT AATGCAAGAA ACAAGAACTT GATATGTATT TTAAGTGAGT AACAAATGAT   
  
  
+ TTTGTCTATA GTTATATGTA TATTATCTAA TTTTGAGTAA TAAGAATCAT TAATATAAAT ATTTTGAATA   
  
  
+ AAATTAGATA GATAAACTAT AAATATAGAG TTAAGTATAC TTATGTTTGG TTATCTTCGA ATATTACCCG   
  
  
+ TTCGGATATA TTATCTGAAC TGGTGAAATA AGTAATATGT TTTGTTGTTT TAATTAGATA ATTTTTAGAC   
  
  
+ CGAGCTTGTG AATATATACT AGACAAACAT TTATATTTCG AGTCTGCACT TATATTCTAT AAGAGCTTGA   
  
  
+ TATATTAGAT TTGAACACTA ACCTGTTAAT ATAGTTTGCC GGTGATTTTT TTTCAAAATT TTGATTCTTA   
  
  
+ GATATGTATA TGGAGTAAAA CTAATTTTTA CAGATGCCCA TTTTTTTAAT TGACACTTAT GTAATTAACT   
  
  
+ GAATTCATAA AACAAGGTTT TTTAAAAAAA TTTAACTCAT ATCAATGAAA CAAAGACGAG AACGAAAGCA   
  
  
+ CAATTCTATG GAAATGGAAA ATGAAGTCAC TTATGGAGAT TCAATAGTAA GCAAATCGAG AGCAGAAAAT   
  
  
+ CTAATCTCCT TTCGTCATTA TACAATCAAT ATTGCCTATT TGGTTTTAGT GATTTGTTTC AGCCGCAAAA   
  
  
+ CTTAATTTTC TTTGGTGCAT ATGAAATCTT AAAAAGAATT AAAATAAGAT ATAATACGTT AACTCTTCAA   
  
  
+ CAACATGATA TATTTAAGAT ACCAATATTT GTATTCATCA TATAAAAATT GTAGTGTTGC AAAATATTAA   
  
  
+ AATTATTTCA TAAATAAACA TTATTATAAG AACTGACTCC GCGGATTATC ATATGGTATA GATTACAGAG   
  
  
+ TGGGTGGGTT TAAATAATTT CCCCGACACA TTATACTTTT AGAAGAGCTA TTAGCTCCAA ATTATTTAAA   
  
  
+ CAATGTTCTA AAGAGCAAAA AAATCAATGT TTTGGATTTT GATCCGACCG AGAGGACTTG TCCGACCATT   
  
  
+ CCATATTAAA ATGTTCCTAG TTCTGATTAG CTAATACCA  

- ATTTCAACAG CACTAAAGAA CGGGAAATAA AAAATAAATA TAATTGTATA TAAAAATTTT TTTTTAATTT   
  
  
- CTGCAAGGAT TGCCTTTGAG TACATAAAAA ACTAATACTT ATACTTATAG GGGAGATATA ATTAGTACCT   
  
  
- CGTAATGTTG TACAAAAGCA TCGGTATACA GTAGTGCTCT TACTAAAAAT CTTAACAATC TTTTTATTTA   
  
  
- ACTAAGTATA TTTGTATATG ATACAAAAAA TAATTTGATT GATAGTTTAA TTAATTATCA CATGTTTTTT   
  
  
- TATAAAAAAA GAAAGGAATT TATTTTTGAT GCCTTAATGG ATTATACCGA TTGTATATAT ACTGTTAATT   
  
  
- ACTAATACTT ATTATGTATA AACTATTTTT TTAAAGATTG GAGAGAGAAA AAACAAATTA AAATATAATA   
  
  
- ATTTCCTTTA AATTGTTAGT GTAATTAGTA TATTATTTTT GTTAATCTAA AAAAGAATAT ACAATATAAA   
  
  
- ACTTAAAAAT TTTTGCTGAT ATTTAATGAT TTTTACTATT CTCAGGGTGT AATTTTTTAA ACACTAGTTG   
  
  
- GCAAATTGAA AAAAAAATCA AGTTCGTTCT ATGTTTACTA GTATATAGAC TATATCTGCA CCCGCAAGCC   
  
  
- TATGTGCAAG CCCAAACATA GTCTATAAAG TCATATTTCC ATATCTTGGG CAAGCCCATA AAGATGTGAG   
  
  
- GCTCAGCCCA AGCCCAAGCC TATAAAACCT AGCCCAAGCT TATAAATTTA AAACTTCTTT TTTTCTTTAA   
  
  
- TAAGTGACAA ATTCAAAAAA TATAAATTTA TATAGAATTG AATTGACTAA AAAAAATCAA AAATTTTCTA   
  
  
- ATTTTATAAT TATACAAACC TCTATTTTGA AATTTTTATC TTTCTGTGAT TAAATCAAAA ACAAAACTTT   
  
  
- TAAATCTACG TTGAAAACAA TTACGTTCTT TGTTCTTGAA CTATACATAA AATTCACTCA TTGTTTACTA   
  
  
- AAACAGATAT CAATATACAT ATAATAGATT AAAACTCATT ATTCTTAGTA ATTATATTTA TAAAACTTAT   
  
  
- TTTAATCTAT CTATTTGATA TTTATATCTC AATTCATATG AATACAAACC AATAGAAGCT TATAATGGGC   
  
  
- AAGCCTATAT AATAGACTTG ACCACTTTAT TCATTATACA AAACAACAAA ATTAATCTAT TAAAAATCTG   
  
  
- GCTCGAACAC TTATATATGA TCTGTTTGTA AATATAAAGC TCAGACGTGA ATATAAGATA TTCTCGAACT   
  
  
- ATATAATCTA AACTTGTGAT TGGACAATTA TATCAAACGG CCACTAAAAA AAAGTTTTAA AACTAAGAAT   
  
  
- CTATACATAT ACCTCATTTT GATTAAAAAT GTCTACGGGT AAAAAAATTA ACTGTGAATA CATTAATTGA   
  
  
- CTTAAGTATT TTGTTCCAAA AAATTTTTTT AAATTGAGTA TAGTTACTTT GTTTCTGCTC TTGCTTTCGT   
  
  
- GTTAAGATAC CTTTACCTTT TACTTCAGTG AATACCTCTA AGTTATCATT CGTTTAGCTC TCGTCTTTTA   
  
  
- GATTAGAGGA AAGCAGTAAT ATGTTAGTTA TAACGGATAA ACCAAAATCA CTAAACAAAG TCGGCGTTTT   
  
  
- GAATTAAAAG AAACCACGTA TACTTTAGAA TTTTTCTTAA TTTTATTCTA TATTATGCAA TTGAGAAGTT   
  
  
- GTTGTACTAT ATAAATTCTA TGGTTATAAA CATAAGTAGT ATATTTTTAA CATCACAACG TTTTATAATT   
  
  
- TTAATAAAGT ATTTATTTGT AATAATATTC TTGACTGAGG CGCCTAATAG TATACCATAT CTAATGTCTC   
  
  
- ACCCACCCAA ATTTATTAAA GGGGCTGTGT AATATGAAAA TCTTCTCGAT AATCGAGGTT TAATAAATTT   
  
  
- GTTACAAGAT TTCTCGTTTT TTTAGTTACA AAACCTAAAA CTAGGCTGGC TCTCCTGAAC AGGCTGGTAA   
  
  
- GGTATAATTT TACAAGGATC AAGACTAATC GATTATGGT
