# Supplementary material for: Effects of Low Nighttime Temperature on Fatty Acid Content in Developing Seeds from Brassica napus L. Based on RNA-Seq and Metabolome
Source: Plants (Basel). 2023 Jan 10;12(2):325. doi: 10.3390/plants12020325 (PMC9862530; doi:10.3390/plants12020325)
Supplement: Supplementary file 1 [file plants-12-00325-s001.zip › File S3.html]

Content-Type: text/html; charset=ISO-8859-1


PlantCARE


Webmaster Firefox specific output  
To save the result:
click on the frame with the right mouse button and save the source code as a text file with extension .html  
REFERENCE:PlantCARE: a database of plant cis-acting regulatory elements and a portal to tools for in silico analysis of promoter sequences.  
Lescot, M., Déhais, P., Moreau, Y., De Moor, B., Rouzé ,P.,and Rombauts, S.  
Nucleic Acids Res., Database issue(2002), 30(1):325-327.   


---

>PlantCARE\_8972   
+ GAACTGTTAA AGCCAATCAT TTAAAATAAA AAGTCAAACC GGTTTAAACC GGTTTTCAAT AAGTTAATAA   
  
  
+ CCGGACTCGG TGTTAAAGAG ATTAAATTTA ACGGTTTAAC TCAGTGAGAT CGGTTTCGCT TTCTCTCTGT   
  
  
+ CGTGTTTGAG AAGTTGAAAG AGAGGAGGAT GAGCGCTGAA GATTTCCAGA AGAAGGTTTC GATCAGAGAC   
  
  
+ TCCTCCGTCG CCGGAGAAAT GGAAATCGAA TGCGGCGGGT CTACTAGCTC CGCGGTTGGT TCGTCTCGAA   
  
  
+ CCTTGGTTTT GCTTCGGAGA CTGCTCGAGA TTCAGGAGCG TCGAGCACAG GCCTACGCCA AACTCAAAAG   
  
  
+ GTTCGATCTT TGGAGTGTTT ATGCTATTAG TTACTGTTTT TGATTCAGTT ACTTGGTGAT TGTTACTGAG   
  
  
+ GGATGTTGGA GTTAAGTAAC TGATAGTGTT GTTGAGGAAG CGATGTCTCA TAAAGTTTAG ATTTTTATCT   
  
  
+ GTTGATCTCT TGCTATGCTT TTGGAATTTG ATTAATCAGA GGGTTCTCAG AGTATGTGGA GACTAGTGGT   
  
  
+ GAAGCGCTTT ATCAGAAGCT CTGCAGCGAG ATAACTGCTG AGTTCAACGA GTGTTCCAAA CAAGTAACTG   
  
  
+ AGTTTTTTTT TCTTCTTTTG ATTGATTACC TTTATGATGT TGTTGAAGAT ACGGTGTTAA AGATTGCGTC   
  
  
+ TTTGTTTGAT GGCAATGCAA GGTACGCGAA ATGGAAACTC TGTTTCTGAA TCCTGACGTT GGAAGATCGG   
  
  
+ ATCTTGCTCA ACTGCTCAGT GACATTCAAA CTCAGGAGAA GCAGAAACTG CATCTGGTCT GATTCCTATA   
  
  
+ TACTCAATTG TTGAACATAA CTGTTAACTT GTTGGACAGA ACAAAAGTTT CTTACTTTAG TTAGTTTTTT   
  
  
+ TTTTTTTTTG CAGACGGTTA CAATACAGGT ACTGAAGAAG GCAGGGAGGC CGTCAGAACG AATGCTGACA   
  
  
+ CACGAGAACT GCAAGTTCAA GAAACCGATG CAGCACGAGT GTGTGCATCT TCATGAGATT ACAGAAGCTG   
  
  
+ AAGGAACAGA GGAAGCAGAG GCGGATGCAG AGTTTGACAA CGCTTTGAAG GAAGCAATCA GAGGAGTGCA   
  
  
+ AGACGCTGTG ACTTGCATCA ATGAGTATTT GGAAGACGTT AGGTACGAGA TTGCAGCTCT TGAAGCTGAT   
  
  
+ TAGTTGTTCT CAATCTCATC ACTTCTTTAC CCCTTTCGAC TGTTAACTTG CTATGTTGCT AATTAGCTTA   
  
  
+ AGATATAAGA TATTTAGAAA CTCAAAAGTT ATGACATTTC TTGTTGTCAA AGTTATCTAT GTTGATTACT   
  
  
+ GTGGGTAGTG GCTACAAATA CAATAGCAAA TTCCAAATTA TTTAAAACAC AATATTTGAT TTGGAAAACA   
  
  
+ ATTGATTTAA AGAGGAACTT TGTTACAAAT ATAAAAGTTA TTAGCATTTT CGGTTCAAAA TGCATAAGAT   
  
  
+ GAGTTGTGTT TTTTTTGTCG TGTACACATC AAAAACTGTC ATACGGACAA TAAGCTAGTC GCTTTGACCC   
  
  
+ ATTCACCGTA TGTCCCAATG TCCTTGACCC TGCCAAATGA TAAGAACGCC AGCACAAAGG TTTTATAAGT   
  
  
+ TTCTCAAAGA GAAACAACAC AATGGGAAAA CACACCATTG CTTGACTTAC AAATGGAGAT CGGTTGGATC   
  
  
+ AATCTCTGGA AACAGGTTGA AGTAGAATCT AAGCCCGTCT GGGCTCATGT CTCTACACAG CAGACCTGAT   
  
  
+ CAAAACAAAT GGGAGAATGA GAATCACATA ACTCTGAAAG AGTCTATCTT TCTACGAGGT GGATTCTGTT   
  
  
+ TACCTTTGGT CTCTGCAGAT AGTAGATGTT CCTTGGCCAT GGCTGGAGCG ATTTTAAGCG TCAAAGCTGC   
  
  
+ ATCACTAGCA GTGACTCCAG TTCTTAAAGT TTCGGTTTTA GTCACAAGCG TTCT  

- CTTGACAATT TCGGTTAGTA AATTTTATTT TTCAGTTTGG CCAAATTTGG CCAAAAGTTA TTCAATTATT   
  
  
- GGCCTGAGCC ACAATTTCTC TAATTTAAAT TGCCAAATTG AGTCACTCTA GCCAAAGCGA AAGAGAGACA   
  
  
- GCACAAACTC TTCAACTTTC TCTCCTCCTA CTCGCGACTT CTAAAGGTCT TCTTCCAAAG CTAGTCTCTG   
  
  
- AGGAGGCAGC GGCCTCTTTA CCTTTAGCTT ACGCCGCCCA GATGATCGAG GCGCCAACCA AGCAGAGCTT   
  
  
- GGAACCAAAA CGAAGCCTCT GACGAGCTCT AAGTCCTCGC AGCTCGTGTC CGGATGCGGT TTGAGTTTTC   
  
  
- CAAGCTAGAA ACCTCACAAA TACGATAATC AATGACAAAA ACTAAGTCAA TGAACCACTA ACAATGACTC   
  
  
- CCTACAACCT CAATTCATTG ACTATCACAA CAACTCCTTC GCTACAGAGT ATTTCAAATC TAAAAATAGA   
  
  
- CAACTAGAGA ACGATACGAA AACCTTAAAC TAATTAGTCT CCCAAGAGTC TCATACACCT CTGATCACCA   
  
  
- CTTCGCGAAA TAGTCTTCGA GACGTCGCTC TATTGACGAC TCAAGTTGCT CACAAGGTTT GTTCATTGAC   
  
  
- TCAAAAAAAA AGAAGAAAAC TAACTAATGG AAATACTACA ACAACTTCTA TGCCACAATT TCTAACGCAG   
  
  
- AAACAAACTA CCGTTACGTT CCATGCGCTT TACCTTTGAG ACAAAGACTT AGGACTGCAA CCTTCTAGCC   
  
  
- TAGAACGAGT TGACGAGTCA CTGTAAGTTT GAGTCCTCTT CGTCTTTGAC GTAGACCAGA CTAAGGATAT   
  
  
- ATGAGTTAAC AACTTGTATT GACAATTGAA CAACCTGTCT TGTTTTCAAA GAATGAAATC AATCAAAAAA   
  
  
- AAAAAAAAAC GTCTGCCAAT GTTATGTCCA TGACTTCTTC CGTCCCTCCG GCAGTCTTGC TTACGACTGT   
  
  
- GTGCTCTTGA CGTTCAAGTT CTTTGGCTAC GTCGTGCTCA CACACGTAGA AGTACTCTAA TGTCTTCGAC   
  
  
- TTCCTTGTCT CCTTCGTCTC CGCCTACGTC TCAAACTGTT GCGAAACTTC CTTCGTTAGT CTCCTCACGT   
  
  
- TCTGCGACAC TGAACGTAGT TACTCATAAA CCTTCTGCAA TCCATGCTCT AACGTCGAGA ACTTCGACTA   
  
  
- ATCAACAAGA GTTAGAGTAG TGAAGAAATG GGGAAAGCTG ACAATTGAAC GATACAACGA TTAATCGAAT   
  
  
- TCTATATTCT ATAAATCTTT GAGTTTTCAA TACTGTAAAG AACAACAGTT TCAATAGATA CAACTAATGA   
  
  
- CACCCATCAC CGATGTTTAT GTTATCGTTT AAGGTTTAAT AAATTTTGTG TTATAAACTA AACCTTTTGT   
  
  
- TAACTAAATT TCTCCTTGAA ACAATGTTTA TATTTTCAAT AATCGTAAAA GCCAAGTTTT ACGTATTCTA   
  
  
- CTCAACACAA AAAAAACAGC ACATGTGTAG TTTTTGACAG TATGCCTGTT ATTCGATCAG CGAAACTGGG   
  
  
- TAAGTGGCAT ACAGGGTTAC AGGAACTGGG ACGGTTTACT ATTCTTGCGG TCGTGTTTCC AAAATATTCA   
  
  
- AAGAGTTTCT CTTTGTTGTG TTACCCTTTT GTGTGGTAAC GAACTGAATG TTTACCTCTA GCCAACCTAG   
  
  
- TTAGAGACCT TTGTCCAACT TCATCTTAGA TTCGGGCAGA CCCGAGTACA GAGATGTGTC GTCTGGACTA   
  
  
- GTTTTGTTTA CCCTCTTACT CTTAGTGTAT TGAGACTTTC TCAGATAGAA AGATGCTCCA CCTAAGACAA   
  
  
- ATGGAAACCA GAGACGTCTA TCATCTACAA GGAACCGGTA CCGACCTCGC TAAAATTCGC AGTTTCGACG   
  
  
- TAGTGATCGT CACTGAGGTC AAGAATTTCA AAGCCAAAAT CAGTGTTCGC AAGA

  
  
Motifs Found  

+   

| Site Name | Organism | Position | Strand | Matrix score. | sequence | function |
| --- | --- | --- | --- | --- | --- | --- |
|  | organism | 325 | - | 4 | motif\_sequence | short\_function |
|  | organism | 1077 | - | 4 | motif\_sequence | short\_function |
|  | organism | 874 | - | 4 | motif\_sequence | short\_function |
|  | organism | 1835 | - | 4 | motif\_sequence | short\_function |
|  | organism | 762 | - | 4 | motif\_sequence | short\_function |
|  | organism | 1731 | + | 4 | motif\_sequence | short\_function |
|  | organism | 1054 | - | 4 | motif\_sequence | short\_function |
|  | organism | 1846 | + | 4 | motif\_sequence | short\_function |
|  | organism | 579 | + | 4 | motif\_sequence | short\_function |
|  | organism | 1558 | + | 4 | motif\_sequence | short\_function |
|  | organism | 1027 | + | 4 | motif\_sequence | short\_function |
|  | organism | 1842 | - | 4 | motif\_sequence | short\_function |
|  | organism | 853 | - | 4 | motif\_sequence | short\_function |
|  | organism | 1831 | + | 4 | motif\_sequence | short\_function |
|  | organism | 1065 | - | 4 | motif\_sequence | short\_function |
|  | organism | 1550 | + | 4 | motif\_sequence | short\_function |
|  | organism | 178 | - | 4 | motif\_sequence | short\_function |
|  | organism | 1665 | - | 4 | motif\_sequence | short\_function |
|  | organism | 675 | - | 4 | motif\_sequence | short\_function |

>PlantCARE\_8972   
+ GAACTGTTAA AGCCAATCAT TTAAAATAAA AAGTCAAACC GGTTTAAACC GGTTTTCAAT AAGTTAATAA   
  
  
+ CCGGACTCGG TGTTAAAGAG ATTAAATTTA ACGGTTTAAC TCAGTGAGAT CGGTTTCGCT TTCTCTCTGT   
  
  
+ CGTGTTTGAG AAGTTGAAAG AGAGGAGGAT GAGCGCTGAA GATTTCCAGA AGAAGGTTTC GATCAGAGAC   
  
  
+ TCCTCCGTCG CCGGAGAAAT GGAAATCGAA TGCGGCGGGT CTACTAGCTC CGCGGTTGGT TCGTCTCGAA   
  
  
+ CCTTGGTTTT GCTTCGGAGA CTGCTCGAGA TTCAGGAGCG TCGAGCACAG GCCTACGCCA AACTCAAAAG   
  
  
+ GTTCGATCTT TGGAGTGTTT ATGCTATTAG TTACTGTTTT TGATTCAGTT ACTTGGTGAT TGTTACTGAG   
  
  
+ GGATGTTGGA GTTAAGTAAC TGATAGTGTT GTTGAGGAAG CGATGTCTCA TAAAGTTTAG ATTTTTATCT   
  
  
+ GTTGATCTCT TGCTATGCTT TTGGAATTTG ATTAATCAGA GGGTTCTCAG AGTATGTGGA GACTAGTGGT   
  
  
+ GAAGCGCTTT ATCAGAAGCT CTGCAGCGAG ATAACTGCTG AGTTCAACGA GTGTTCCAAA CAAGTAACTG   
  
  
+ AGTTTTTTTT TCTTCTTTTG ATTGATTACC TTTATGATGT TGTTGAAGAT ACGGTGTTAA AGATTGCGTC   
  
  
+ TTTGTTTGAT GGCAATGCAA GGTACGCGAA ATGGAAACTC TGTTTCTGAA TCCTGACGTT GGAAGATCGG   
  
  
+ ATCTTGCTCA ACTGCTCAGT GACATTCAAA CTCAGGAGAA GCAGAAACTG CATCTGGTCT GATTCCTATA   
  
  
+ TACTCAATTG TTGAACATAA CTGTTAACTT GTTGGACAGA ACAAAAGTTT CTTACTTTAG TTAGTTTTTT   
  
  
+ TTTTTTTTTG CAGACGGTTA CAATACAGGT ACTGAAGAAG GCAGGGAGGC CGTCAGAACG AATGCTGACA   
  
  
+ CACGAGAACT GCAAGTTCAA GAAACCGATG CAGCACGAGT GTGTGCATCT TCATGAGATT ACAGAAGCTG   
  
  
+ AAGGAACAGA GGAAGCAGAG GCGGATGCAG AGTTTGACAA CGCTTTGAAG GAAGCAATCA GAGGAGTGCA   
  
  
+ AGACGCTGTG ACTTGCATCA ATGAGTATTT GGAAGACGTT AGGTACGAGA TTGCAGCTCT TGAAGCTGAT   
  
  
+ TAGTTGTTCT CAATCTCATC ACTTCTTTAC CCCTTTCGAC TGTTAACTTG CTATGTTGCT AATTAGCTTA   
  
  
+ AGATATAAGA TATTTAGAAA CTCAAAAGTT ATGACATTTC TTGTTGTCAA AGTTATCTAT GTTGATTACT   
  
  
+ GTGGGTAGTG GCTACAAATA CAATAGCAAA TTCCAAATTA TTTAAAACAC AATATTTGAT TTGGAAAACA   
  
  
+ ATTGATTTAA AGAGGAACTT TGTTACAAAT ATAAAAGTTA TTAGCATTTT CGGTTCAAAA TGCATAAGAT   
  
  
+ GAGTTGTGTT TTTTTTGTCG TGTACACATC AAAAACTGTC ATACGGACAA TAAGCTAGTC GCTTTGACCC   
  
  
+ ATTCACCGTA TGTCCCAATG TCCTTGACCC TGCCAAATGA TAAGAACGCC AGCACAAAGG TTTTATAAGT   
  
  
+ TTCTCAAAGA GAAACAACAC AATGGGAAAA CACACCATTG CTTGACTTAC AAATGGAGAT CGGTTGGATC   
  
  
+ AATCTCTGGA AACAGGTTGA AGTAGAATCT AAGCCCGTCT GGGCTCATGT CTCTACACAG CAGACCTGAT   
  
  
+ CAAAACAAAT GGGAGAATGA GAATCACATA ACTCTGAAAG AGTCTATCTT TCTACGAGGT GGATTCTGTT   
  
  
+ TACCTTTGGT CTCTGCAGAT AGTAGATGTT CCTTGGCCAT GGCTGGAGCG ATTTTAAGCG TCAAAGCTGC   
  
  
+ ATCACTAGCA GTGACTCCAG TTCTTAAAGT TTCGGTTTTA GTCACAAGCG TTCT  

- CTTGACAATT TCGGTTAGTA AATTTTATTT TTCAGTTTGG CCAAATTTGG CCAAAAGTTA TTCAATTATT   
  
  
- GGCCTGAGCC ACAATTTCTC TAATTTAAAT TGCCAAATTG AGTCACTCTA GCCAAAGCGA AAGAGAGACA   
  
  
- GCACAAACTC TTCAACTTTC TCTCCTCCTA CTCGCGACTT CTAAAGGTCT TCTTCCAAAG CTAGTCTCTG   
  
  
- AGGAGGCAGC GGCCTCTTTA CCTTTAGCTT ACGCCGCCCA GATGATCGAG GCGCCAACCA AGCAGAGCTT   
  
  
- GGAACCAAAA CGAAGCCTCT GACGAGCTCT AAGTCCTCGC AGCTCGTGTC CGGATGCGGT TTGAGTTTTC   
  
  
- CAAGCTAGAA ACCTCACAAA TACGATAATC AATGACAAAA ACTAAGTCAA TGAACCACTA ACAATGACTC   
  
  
- CCTACAACCT CAATTCATTG ACTATCACAA CAACTCCTTC GCTACAGAGT ATTTCAAATC TAAAAATAGA   
  
  
- CAACTAGAGA ACGATACGAA AACCTTAAAC TAATTAGTCT CCCAAGAGTC TCATACACCT CTGATCACCA   
  
  
- CTTCGCGAAA TAGTCTTCGA GACGTCGCTC TATTGACGAC TCAAGTTGCT CACAAGGTTT GTTCATTGAC   
  
  
- TCAAAAAAAA AGAAGAAAAC TAACTAATGG AAATACTACA ACAACTTCTA TGCCACAATT TCTAACGCAG   
  
  
- AAACAAACTA CCGTTACGTT CCATGCGCTT TACCTTTGAG ACAAAGACTT AGGACTGCAA CCTTCTAGCC   
  
  
- TAGAACGAGT TGACGAGTCA CTGTAAGTTT GAGTCCTCTT CGTCTTTGAC GTAGACCAGA CTAAGGATAT   
  
  
- ATGAGTTAAC AACTTGTATT GACAATTGAA CAACCTGTCT TGTTTTCAAA GAATGAAATC AATCAAAAAA   
  
  
- AAAAAAAAAC GTCTGCCAAT GTTATGTCCA TGACTTCTTC CGTCCCTCCG GCAGTCTTGC TTACGACTGT   
  
  
- GTGCTCTTGA CGTTCAAGTT CTTTGGCTAC GTCGTGCTCA CACACGTAGA AGTACTCTAA TGTCTTCGAC   
  
  
- TTCCTTGTCT CCTTCGTCTC CGCCTACGTC TCAAACTGTT GCGAAACTTC CTTCGTTAGT CTCCTCACGT   
  
  
- TCTGCGACAC TGAACGTAGT TACTCATAAA CCTTCTGCAA TCCATGCTCT AACGTCGAGA ACTTCGACTA   
  
  
- ATCAACAAGA GTTAGAGTAG TGAAGAAATG GGGAAAGCTG ACAATTGAAC GATACAACGA TTAATCGAAT   
  
  
- TCTATATTCT ATAAATCTTT GAGTTTTCAA TACTGTAAAG AACAACAGTT TCAATAGATA CAACTAATGA   
  
  
- CACCCATCAC CGATGTTTAT GTTATCGTTT AAGGTTTAAT AAATTTTGTG TTATAAACTA AACCTTTTGT   
  
  
- TAACTAAATT TCTCCTTGAA ACAATGTTTA TATTTTCAAT AATCGTAAAA GCCAAGTTTT ACGTATTCTA   
  
  
- CTCAACACAA AAAAAACAGC ACATGTGTAG TTTTTGACAG TATGCCTGTT ATTCGATCAG CGAAACTGGG   
  
  
- TAAGTGGCAT ACAGGGTTAC AGGAACTGGG ACGGTTTACT ATTCTTGCGG TCGTGTTTCC AAAATATTCA   
  
  
- AAGAGTTTCT CTTTGTTGTG TTACCCTTTT GTGTGGTAAC GAACTGAATG TTTACCTCTA GCCAACCTAG   
  
  
- TTAGAGACCT TTGTCCAACT TCATCTTAGA TTCGGGCAGA CCCGAGTACA GAGATGTGTC GTCTGGACTA   
  
  
- GTTTTGTTTA CCCTCTTACT CTTAGTGTAT TGAGACTTTC TCAGATAGAA AGATGCTCCA CCTAAGACAA   
  
  
- ATGGAAACCA GAGACGTCTA TCATCTACAA GGAACCGGTA CCGACCTCGC TAAAATTCGC AGTTTCGACG   
  
  
- TAGTGATCGT CACTGAGGTC AAGAATTTCA AAGCCAAAAT CAGTGTTCGC AAGA

+     AAGAA-motif

| Site Name | Organism | Position | Strand | Matrix score. | sequence | function |
| --- | --- | --- | --- | --- | --- | --- |
| AAGAA-motif | Avena sativa | 1212 | - | 9 | gGTAAAGAAA |  |

>PlantCARE\_8972   
+ GAACTGTTAA AGCCAATCAT TTAAAATAAA AAGTCAAACC GGTTTAAACC GGTTTTCAAT AAGTTAATAA   
  
  
+ CCGGACTCGG TGTTAAAGAG ATTAAATTTA ACGGTTTAAC TCAGTGAGAT CGGTTTCGCT TTCTCTCTGT   
  
  
+ CGTGTTTGAG AAGTTGAAAG AGAGGAGGAT GAGCGCTGAA GATTTCCAGA AGAAGGTTTC GATCAGAGAC   
  
  
+ TCCTCCGTCG CCGGAGAAAT GGAAATCGAA TGCGGCGGGT CTACTAGCTC CGCGGTTGGT TCGTCTCGAA   
  
  
+ CCTTGGTTTT GCTTCGGAGA CTGCTCGAGA TTCAGGAGCG TCGAGCACAG GCCTACGCCA AACTCAAAAG   
  
  
+ GTTCGATCTT TGGAGTGTTT ATGCTATTAG TTACTGTTTT TGATTCAGTT ACTTGGTGAT TGTTACTGAG   
  
  
+ GGATGTTGGA GTTAAGTAAC TGATAGTGTT GTTGAGGAAG CGATGTCTCA TAAAGTTTAG ATTTTTATCT   
  
  
+ GTTGATCTCT TGCTATGCTT TTGGAATTTG ATTAATCAGA GGGTTCTCAG AGTATGTGGA GACTAGTGGT   
  
  
+ GAAGCGCTTT ATCAGAAGCT CTGCAGCGAG ATAACTGCTG AGTTCAACGA GTGTTCCAAA CAAGTAACTG   
  
  
+ AGTTTTTTTT TCTTCTTTTG ATTGATTACC TTTATGATGT TGTTGAAGAT ACGGTGTTAA AGATTGCGTC   
  
  
+ TTTGTTTGAT GGCAATGCAA GGTACGCGAA ATGGAAACTC TGTTTCTGAA TCCTGACGTT GGAAGATCGG   
  
  
+ ATCTTGCTCA ACTGCTCAGT GACATTCAAA CTCAGGAGAA GCAGAAACTG CATCTGGTCT GATTCCTATA   
  
  
+ TACTCAATTG TTGAACATAA CTGTTAACTT GTTGGACAGA ACAAAAGTTT CTTACTTTAG TTAGTTTTTT   
  
  
+ TTTTTTTTTG CAGACGGTTA CAATACAGGT ACTGAAGAAG GCAGGGAGGC CGTCAGAACG AATGCTGACA   
  
  
+ CACGAGAACT GCAAGTTCAA GAAACCGATG CAGCACGAGT GTGTGCATCT TCATGAGATT ACAGAAGCTG   
  
  
+ AAGGAACAGA GGAAGCAGAG GCGGATGCAG AGTTTGACAA CGCTTTGAAG GAAGCAATCA GAGGAGTGCA   
  
  
+ AGACGCTGTG ACTTGCATCA ATGAGTATTT GGAAGACGTT AGGTACGAGA TTGCAGCTCT TGAAGCTGAT   
  
  
+ TAGTTGTTCT CAATCTCATC ACTTCTTTAC CCCTTTCGAC TGTTAACTTG CTATGTTGCT AATTAGCTTA   
  
  
+ AGATATAAGA TATTTAGAAA CTCAAAAGTT ATGACATTTC TTGTTGTCAA AGTTATCTAT GTTGATTACT   
  
  
+ GTGGGTAGTG GCTACAAATA CAATAGCAAA TTCCAAATTA TTTAAAACAC AATATTTGAT TTGGAAAACA   
  
  
+ ATTGATTTAA AGAGGAACTT TGTTACAAAT ATAAAAGTTA TTAGCATTTT CGGTTCAAAA TGCATAAGAT   
  
  
+ GAGTTGTGTT TTTTTTGTCG TGTACACATC AAAAACTGTC ATACGGACAA TAAGCTAGTC GCTTTGACCC   
  
  
+ ATTCACCGTA TGTCCCAATG TCCTTGACCC TGCCAAATGA TAAGAACGCC AGCACAAAGG TTTTATAAGT   
  
  
+ TTCTCAAAGA GAAACAACAC AATGGGAAAA CACACCATTG CTTGACTTAC AAATGGAGAT CGGTTGGATC   
  
  
+ AATCTCTGGA AACAGGTTGA AGTAGAATCT AAGCCCGTCT GGGCTCATGT CTCTACACAG CAGACCTGAT   
  
  
+ CAAAACAAAT GGGAGAATGA GAATCACATA ACTCTGAAAG AGTCTATCTT TCTACGAGGT GGATTCTGTT   
  
  
+ TACCTTTGGT CTCTGCAGAT AGTAGATGTT CCTTGGCCAT GGCTGGAGCG ATTTTAAGCG TCAAAGCTGC   
  
  
+ ATCACTAGCA GTGACTCCAG TTCTTAAAGT TTCGGTTTTA GTCACAAGCG TTCT  

- CTTGACAATT TCGGTTAGTA AATTTTATTT TTCAGTTTGG CCAAATTTGG CCAAAAGTTA TTCAATTATT   
  
  
- GGCCTGAGCC ACAATTTCTC TAATTTAAAT TGCCAAATTG AGTCACTCTA GCCAAAGCGA AAGAGAGACA   
  
  
- GCACAAACTC TTCAACTTTC TCTCCTCCTA CTCGCGACTT CTAAAGGTCT TCTTCCAAAG CTAGTCTCTG   
  
  
- AGGAGGCAGC GGCCTCTTTA CCTTTAGCTT ACGCCGCCCA GATGATCGAG GCGCCAACCA AGCAGAGCTT   
  
  
- GGAACCAAAA CGAAGCCTCT GACGAGCTCT AAGTCCTCGC AGCTCGTGTC CGGATGCGGT TTGAGTTTTC   
  
  
- CAAGCTAGAA ACCTCACAAA TACGATAATC AATGACAAAA ACTAAGTCAA TGAACCACTA ACAATGACTC   
  
  
- CCTACAACCT CAATTCATTG ACTATCACAA CAACTCCTTC GCTACAGAGT ATTTCAAATC TAAAAATAGA   
  
  
- CAACTAGAGA ACGATACGAA AACCTTAAAC TAATTAGTCT CCCAAGAGTC TCATACACCT CTGATCACCA   
  
  
- CTTCGCGAAA TAGTCTTCGA GACGTCGCTC TATTGACGAC TCAAGTTGCT CACAAGGTTT GTTCATTGAC   
  
  
- TCAAAAAAAA AGAAGAAAAC TAACTAATGG AAATACTACA ACAACTTCTA TGCCACAATT TCTAACGCAG   
  
  
- AAACAAACTA CCGTTACGTT CCATGCGCTT TACCTTTGAG ACAAAGACTT AGGACTGCAA CCTTCTAGCC   
  
  
- TAGAACGAGT TGACGAGTCA CTGTAAGTTT GAGTCCTCTT CGTCTTTGAC GTAGACCAGA CTAAGGATAT   
  
  
- ATGAGTTAAC AACTTGTATT GACAATTGAA CAACCTGTCT TGTTTTCAAA GAATGAAATC AATCAAAAAA   
  
  
- AAAAAAAAAC GTCTGCCAAT GTTATGTCCA TGACTTCTTC CGTCCCTCCG GCAGTCTTGC TTACGACTGT   
  
  
- GTGCTCTTGA CGTTCAAGTT CTTTGGCTAC GTCGTGCTCA CACACGTAGA AGTACTCTAA TGTCTTCGAC   
  
  
- TTCCTTGTCT CCTTCGTCTC CGCCTACGTC TCAAACTGTT GCGAAACTTC CTTCGTTAGT CTCCTCACGT   
  
  
- TCTGCGACAC TGAACGTAGT TACTCATAAA CCTTCTGCAA TCCATGCTCT AACGTCGAGA ACTTCGACTA   
  
  
- ATCAACAAGA GTTAGAGTAG TGAAGAAATG GGGAAAGCTG ACAATTGAAC GATACAACGA TTAATCGAAT   
  
  
- TCTATATTCT ATAAATCTTT GAGTTTTCAA TACTGTAAAG AACAACAGTT TCAATAGATA CAACTAATGA   
  
  
- CACCCATCAC CGATGTTTAT GTTATCGTTT AAGGTTTAAT AAATTTTGTG TTATAAACTA AACCTTTTGT   
  
  
- TAACTAAATT TCTCCTTGAA ACAATGTTTA TATTTTCAAT AATCGTAAAA GCCAAGTTTT ACGTATTCTA   
  
  
- CTCAACACAA AAAAAACAGC ACATGTGTAG TTTTTGACAG TATGCCTGTT ATTCGATCAG CGAAACTGGG   
  
  
- TAAGTGGCAT ACAGGGTTAC AGGAACTGGG ACGGTTTACT ATTCTTGCGG TCGTGTTTCC AAAATATTCA   
  
  
- AAGAGTTTCT CTTTGTTGTG TTACCCTTTT GTGTGGTAAC GAACTGAATG TTTACCTCTA GCCAACCTAG   
  
  
- TTAGAGACCT TTGTCCAACT TCATCTTAGA TTCGGGCAGA CCCGAGTACA GAGATGTGTC GTCTGGACTA   
  
  
- GTTTTGTTTA CCCTCTTACT CTTAGTGTAT TGAGACTTTC TCAGATAGAA AGATGCTCCA CCTAAGACAA   
  
  
- ATGGAAACCA GAGACGTCTA TCATCTACAA GGAACCGGTA CCGACCTCGC TAAAATTCGC AGTTTCGACG   
  
  
- TAGTGATCGT CACTGAGGTC AAGAATTTCA AAGCCAAAAT CAGTGTTCGC AAGA

+     ACE

| Site Name | Organism | Position | Strand | Matrix score. | sequence | function |
| --- | --- | --- | --- | --- | --- | --- |
| ACE | Petroselinum crispum | 1153 | - | 9 | CTAACGTATT | cis-acting element involved in light responsiveness |

>PlantCARE\_8972   
+ GAACTGTTAA AGCCAATCAT TTAAAATAAA AAGTCAAACC GGTTTAAACC GGTTTTCAAT AAGTTAATAA   
  
  
+ CCGGACTCGG TGTTAAAGAG ATTAAATTTA ACGGTTTAAC TCAGTGAGAT CGGTTTCGCT TTCTCTCTGT   
  
  
+ CGTGTTTGAG AAGTTGAAAG AGAGGAGGAT GAGCGCTGAA GATTTCCAGA AGAAGGTTTC GATCAGAGAC   
  
  
+ TCCTCCGTCG CCGGAGAAAT GGAAATCGAA TGCGGCGGGT CTACTAGCTC CGCGGTTGGT TCGTCTCGAA   
  
  
+ CCTTGGTTTT GCTTCGGAGA CTGCTCGAGA TTCAGGAGCG TCGAGCACAG GCCTACGCCA AACTCAAAAG   
  
  
+ GTTCGATCTT TGGAGTGTTT ATGCTATTAG TTACTGTTTT TGATTCAGTT ACTTGGTGAT TGTTACTGAG   
  
  
+ GGATGTTGGA GTTAAGTAAC TGATAGTGTT GTTGAGGAAG CGATGTCTCA TAAAGTTTAG ATTTTTATCT   
  
  
+ GTTGATCTCT TGCTATGCTT TTGGAATTTG ATTAATCAGA GGGTTCTCAG AGTATGTGGA GACTAGTGGT   
  
  
+ GAAGCGCTTT ATCAGAAGCT CTGCAGCGAG ATAACTGCTG AGTTCAACGA GTGTTCCAAA CAAGTAACTG   
  
  
+ AGTTTTTTTT TCTTCTTTTG ATTGATTACC TTTATGATGT TGTTGAAGAT ACGGTGTTAA AGATTGCGTC   
  
  
+ TTTGTTTGAT GGCAATGCAA GGTACGCGAA ATGGAAACTC TGTTTCTGAA TCCTGACGTT GGAAGATCGG   
  
  
+ ATCTTGCTCA ACTGCTCAGT GACATTCAAA CTCAGGAGAA GCAGAAACTG CATCTGGTCT GATTCCTATA   
  
  
+ TACTCAATTG TTGAACATAA CTGTTAACTT GTTGGACAGA ACAAAAGTTT CTTACTTTAG TTAGTTTTTT   
  
  
+ TTTTTTTTTG CAGACGGTTA CAATACAGGT ACTGAAGAAG GCAGGGAGGC CGTCAGAACG AATGCTGACA   
  
  
+ CACGAGAACT GCAAGTTCAA GAAACCGATG CAGCACGAGT GTGTGCATCT TCATGAGATT ACAGAAGCTG   
  
  
+ AAGGAACAGA GGAAGCAGAG GCGGATGCAG AGTTTGACAA CGCTTTGAAG GAAGCAATCA GAGGAGTGCA   
  
  
+ AGACGCTGTG ACTTGCATCA ATGAGTATTT GGAAGACGTT AGGTACGAGA TTGCAGCTCT TGAAGCTGAT   
  
  
+ TAGTTGTTCT CAATCTCATC ACTTCTTTAC CCCTTTCGAC TGTTAACTTG CTATGTTGCT AATTAGCTTA   
  
  
+ AGATATAAGA TATTTAGAAA CTCAAAAGTT ATGACATTTC TTGTTGTCAA AGTTATCTAT GTTGATTACT   
  
  
+ GTGGGTAGTG GCTACAAATA CAATAGCAAA TTCCAAATTA TTTAAAACAC AATATTTGAT TTGGAAAACA   
  
  
+ ATTGATTTAA AGAGGAACTT TGTTACAAAT ATAAAAGTTA TTAGCATTTT CGGTTCAAAA TGCATAAGAT   
  
  
+ GAGTTGTGTT TTTTTTGTCG TGTACACATC AAAAACTGTC ATACGGACAA TAAGCTAGTC GCTTTGACCC   
  
  
+ ATTCACCGTA TGTCCCAATG TCCTTGACCC TGCCAAATGA TAAGAACGCC AGCACAAAGG TTTTATAAGT   
  
  
+ TTCTCAAAGA GAAACAACAC AATGGGAAAA CACACCATTG CTTGACTTAC AAATGGAGAT CGGTTGGATC   
  
  
+ AATCTCTGGA AACAGGTTGA AGTAGAATCT AAGCCCGTCT GGGCTCATGT CTCTACACAG CAGACCTGAT   
  
  
+ CAAAACAAAT GGGAGAATGA GAATCACATA ACTCTGAAAG AGTCTATCTT TCTACGAGGT GGATTCTGTT   
  
  
+ TACCTTTGGT CTCTGCAGAT AGTAGATGTT CCTTGGCCAT GGCTGGAGCG ATTTTAAGCG TCAAAGCTGC   
  
  
+ ATCACTAGCA GTGACTCCAG TTCTTAAAGT TTCGGTTTTA GTCACAAGCG TTCT  

- CTTGACAATT TCGGTTAGTA AATTTTATTT TTCAGTTTGG CCAAATTTGG CCAAAAGTTA TTCAATTATT   
  
  
- GGCCTGAGCC ACAATTTCTC TAATTTAAAT TGCCAAATTG AGTCACTCTA GCCAAAGCGA AAGAGAGACA   
  
  
- GCACAAACTC TTCAACTTTC TCTCCTCCTA CTCGCGACTT CTAAAGGTCT TCTTCCAAAG CTAGTCTCTG   
  
  
- AGGAGGCAGC GGCCTCTTTA CCTTTAGCTT ACGCCGCCCA GATGATCGAG GCGCCAACCA AGCAGAGCTT   
  
  
- GGAACCAAAA CGAAGCCTCT GACGAGCTCT AAGTCCTCGC AGCTCGTGTC CGGATGCGGT TTGAGTTTTC   
  
  
- CAAGCTAGAA ACCTCACAAA TACGATAATC AATGACAAAA ACTAAGTCAA TGAACCACTA ACAATGACTC   
  
  
- CCTACAACCT CAATTCATTG ACTATCACAA CAACTCCTTC GCTACAGAGT ATTTCAAATC TAAAAATAGA   
  
  
- CAACTAGAGA ACGATACGAA AACCTTAAAC TAATTAGTCT CCCAAGAGTC TCATACACCT CTGATCACCA   
  
  
- CTTCGCGAAA TAGTCTTCGA GACGTCGCTC TATTGACGAC TCAAGTTGCT CACAAGGTTT GTTCATTGAC   
  
  
- TCAAAAAAAA AGAAGAAAAC TAACTAATGG AAATACTACA ACAACTTCTA TGCCACAATT TCTAACGCAG   
  
  
- AAACAAACTA CCGTTACGTT CCATGCGCTT TACCTTTGAG ACAAAGACTT AGGACTGCAA CCTTCTAGCC   
  
  
- TAGAACGAGT TGACGAGTCA CTGTAAGTTT GAGTCCTCTT CGTCTTTGAC GTAGACCAGA CTAAGGATAT   
  
  
- ATGAGTTAAC AACTTGTATT GACAATTGAA CAACCTGTCT TGTTTTCAAA GAATGAAATC AATCAAAAAA   
  
  
- AAAAAAAAAC GTCTGCCAAT GTTATGTCCA TGACTTCTTC CGTCCCTCCG GCAGTCTTGC TTACGACTGT   
  
  
- GTGCTCTTGA CGTTCAAGTT CTTTGGCTAC GTCGTGCTCA CACACGTAGA AGTACTCTAA TGTCTTCGAC   
  
  
- TTCCTTGTCT CCTTCGTCTC CGCCTACGTC TCAAACTGTT GCGAAACTTC CTTCGTTAGT CTCCTCACGT   
  
  
- TCTGCGACAC TGAACGTAGT TACTCATAAA CCTTCTGCAA TCCATGCTCT AACGTCGAGA ACTTCGACTA   
  
  
- ATCAACAAGA GTTAGAGTAG TGAAGAAATG GGGAAAGCTG ACAATTGAAC GATACAACGA TTAATCGAAT   
  
  
- TCTATATTCT ATAAATCTTT GAGTTTTCAA TACTGTAAAG AACAACAGTT TCAATAGATA CAACTAATGA   
  
  
- CACCCATCAC CGATGTTTAT GTTATCGTTT AAGGTTTAAT AAATTTTGTG TTATAAACTA AACCTTTTGT   
  
  
- TAACTAAATT TCTCCTTGAA ACAATGTTTA TATTTTCAAT AATCGTAAAA GCCAAGTTTT ACGTATTCTA   
  
  
- CTCAACACAA AAAAAACAGC ACATGTGTAG TTTTTGACAG TATGCCTGTT ATTCGATCAG CGAAACTGGG   
  
  
- TAAGTGGCAT ACAGGGTTAC AGGAACTGGG ACGGTTTACT ATTCTTGCGG TCGTGTTTCC AAAATATTCA   
  
  
- AAGAGTTTCT CTTTGTTGTG TTACCCTTTT GTGTGGTAAC GAACTGAATG TTTACCTCTA GCCAACCTAG   
  
  
- TTAGAGACCT TTGTCCAACT TCATCTTAGA TTCGGGCAGA CCCGAGTACA GAGATGTGTC GTCTGGACTA   
  
  
- GTTTTGTTTA CCCTCTTACT CTTAGTGTAT TGAGACTTTC TCAGATAGAA AGATGCTCCA CCTAAGACAA   
  
  
- ATGGAAACCA GAGACGTCTA TCATCTACAA GGAACCGGTA CCGACCTCGC TAAAATTCGC AGTTTCGACG   
  
  
- TAGTGATCGT CACTGAGGTC AAGAATTTCA AAGCCAAAAT CAGTGTTCGC AAGA

+     AE-box

| Site Name | Organism | Position | Strand | Matrix score. | sequence | function |
| --- | --- | --- | --- | --- | --- | --- |
| AE-box | Arabidopsis thaliana | 885 | - | 8 | AGAAACTT | part of a module for light response |
| AE-box | Arabidopsis thaliana | 1620 | + | 8 | AGAAACAA | part of a module for light response |
| AE-box | Arabidopsis thaliana | 1607 | - | 8 | AGAAACTT | part of a module for light response |

>PlantCARE\_8972   
+ GAACTGTTAA AGCCAATCAT TTAAAATAAA AAGTCAAACC GGTTTAAACC GGTTTTCAAT AAGTTAATAA   
  
  
+ CCGGACTCGG TGTTAAAGAG ATTAAATTTA ACGGTTTAAC TCAGTGAGAT CGGTTTCGCT TTCTCTCTGT   
  
  
+ CGTGTTTGAG AAGTTGAAAG AGAGGAGGAT GAGCGCTGAA GATTTCCAGA AGAAGGTTTC GATCAGAGAC   
  
  
+ TCCTCCGTCG CCGGAGAAAT GGAAATCGAA TGCGGCGGGT CTACTAGCTC CGCGGTTGGT TCGTCTCGAA   
  
  
+ CCTTGGTTTT GCTTCGGAGA CTGCTCGAGA TTCAGGAGCG TCGAGCACAG GCCTACGCCA AACTCAAAAG   
  
  
+ GTTCGATCTT TGGAGTGTTT ATGCTATTAG TTACTGTTTT TGATTCAGTT ACTTGGTGAT TGTTACTGAG   
  
  
+ GGATGTTGGA GTTAAGTAAC TGATAGTGTT GTTGAGGAAG CGATGTCTCA TAAAGTTTAG ATTTTTATCT   
  
  
+ GTTGATCTCT TGCTATGCTT TTGGAATTTG ATTAATCAGA GGGTTCTCAG AGTATGTGGA GACTAGTGGT   
  
  
+ GAAGCGCTTT ATCAGAAGCT CTGCAGCGAG ATAACTGCTG AGTTCAACGA GTGTTCCAAA CAAGTAACTG   
  
  
+ AGTTTTTTTT TCTTCTTTTG ATTGATTACC TTTATGATGT TGTTGAAGAT ACGGTGTTAA AGATTGCGTC   
  
  
+ TTTGTTTGAT GGCAATGCAA GGTACGCGAA ATGGAAACTC TGTTTCTGAA TCCTGACGTT GGAAGATCGG   
  
  
+ ATCTTGCTCA ACTGCTCAGT GACATTCAAA CTCAGGAGAA GCAGAAACTG CATCTGGTCT GATTCCTATA   
  
  
+ TACTCAATTG TTGAACATAA CTGTTAACTT GTTGGACAGA ACAAAAGTTT CTTACTTTAG TTAGTTTTTT   
  
  
+ TTTTTTTTTG CAGACGGTTA CAATACAGGT ACTGAAGAAG GCAGGGAGGC CGTCAGAACG AATGCTGACA   
  
  
+ CACGAGAACT GCAAGTTCAA GAAACCGATG CAGCACGAGT GTGTGCATCT TCATGAGATT ACAGAAGCTG   
  
  
+ AAGGAACAGA GGAAGCAGAG GCGGATGCAG AGTTTGACAA CGCTTTGAAG GAAGCAATCA GAGGAGTGCA   
  
  
+ AGACGCTGTG ACTTGCATCA ATGAGTATTT GGAAGACGTT AGGTACGAGA TTGCAGCTCT TGAAGCTGAT   
  
  
+ TAGTTGTTCT CAATCTCATC ACTTCTTTAC CCCTTTCGAC TGTTAACTTG CTATGTTGCT AATTAGCTTA   
  
  
+ AGATATAAGA TATTTAGAAA CTCAAAAGTT ATGACATTTC TTGTTGTCAA AGTTATCTAT GTTGATTACT   
  
  
+ GTGGGTAGTG GCTACAAATA CAATAGCAAA TTCCAAATTA TTTAAAACAC AATATTTGAT TTGGAAAACA   
  
  
+ ATTGATTTAA AGAGGAACTT TGTTACAAAT ATAAAAGTTA TTAGCATTTT CGGTTCAAAA TGCATAAGAT   
  
  
+ GAGTTGTGTT TTTTTTGTCG TGTACACATC AAAAACTGTC ATACGGACAA TAAGCTAGTC GCTTTGACCC   
  
  
+ ATTCACCGTA TGTCCCAATG TCCTTGACCC TGCCAAATGA TAAGAACGCC AGCACAAAGG TTTTATAAGT   
  
  
+ TTCTCAAAGA GAAACAACAC AATGGGAAAA CACACCATTG CTTGACTTAC AAATGGAGAT CGGTTGGATC   
  
  
+ AATCTCTGGA AACAGGTTGA AGTAGAATCT AAGCCCGTCT GGGCTCATGT CTCTACACAG CAGACCTGAT   
  
  
+ CAAAACAAAT GGGAGAATGA GAATCACATA ACTCTGAAAG AGTCTATCTT TCTACGAGGT GGATTCTGTT   
  
  
+ TACCTTTGGT CTCTGCAGAT AGTAGATGTT CCTTGGCCAT GGCTGGAGCG ATTTTAAGCG TCAAAGCTGC   
  
  
+ ATCACTAGCA GTGACTCCAG TTCTTAAAGT TTCGGTTTTA GTCACAAGCG TTCT  

- CTTGACAATT TCGGTTAGTA AATTTTATTT TTCAGTTTGG CCAAATTTGG CCAAAAGTTA TTCAATTATT   
  
  
- GGCCTGAGCC ACAATTTCTC TAATTTAAAT TGCCAAATTG AGTCACTCTA GCCAAAGCGA AAGAGAGACA   
  
  
- GCACAAACTC TTCAACTTTC TCTCCTCCTA CTCGCGACTT CTAAAGGTCT TCTTCCAAAG CTAGTCTCTG   
  
  
- AGGAGGCAGC GGCCTCTTTA CCTTTAGCTT ACGCCGCCCA GATGATCGAG GCGCCAACCA AGCAGAGCTT   
  
  
- GGAACCAAAA CGAAGCCTCT GACGAGCTCT AAGTCCTCGC AGCTCGTGTC CGGATGCGGT TTGAGTTTTC   
  
  
- CAAGCTAGAA ACCTCACAAA TACGATAATC AATGACAAAA ACTAAGTCAA TGAACCACTA ACAATGACTC   
  
  
- CCTACAACCT CAATTCATTG ACTATCACAA CAACTCCTTC GCTACAGAGT ATTTCAAATC TAAAAATAGA   
  
  
- CAACTAGAGA ACGATACGAA AACCTTAAAC TAATTAGTCT CCCAAGAGTC TCATACACCT CTGATCACCA   
  
  
- CTTCGCGAAA TAGTCTTCGA GACGTCGCTC TATTGACGAC TCAAGTTGCT CACAAGGTTT GTTCATTGAC   
  
  
- TCAAAAAAAA AGAAGAAAAC TAACTAATGG AAATACTACA ACAACTTCTA TGCCACAATT TCTAACGCAG   
  
  
- AAACAAACTA CCGTTACGTT CCATGCGCTT TACCTTTGAG ACAAAGACTT AGGACTGCAA CCTTCTAGCC   
  
  
- TAGAACGAGT TGACGAGTCA CTGTAAGTTT GAGTCCTCTT CGTCTTTGAC GTAGACCAGA CTAAGGATAT   
  
  
- ATGAGTTAAC AACTTGTATT GACAATTGAA CAACCTGTCT TGTTTTCAAA GAATGAAATC AATCAAAAAA   
  
  
- AAAAAAAAAC GTCTGCCAAT GTTATGTCCA TGACTTCTTC CGTCCCTCCG GCAGTCTTGC TTACGACTGT   
  
  
- GTGCTCTTGA CGTTCAAGTT CTTTGGCTAC GTCGTGCTCA CACACGTAGA AGTACTCTAA TGTCTTCGAC   
  
  
- TTCCTTGTCT CCTTCGTCTC CGCCTACGTC TCAAACTGTT GCGAAACTTC CTTCGTTAGT CTCCTCACGT   
  
  
- TCTGCGACAC TGAACGTAGT TACTCATAAA CCTTCTGCAA TCCATGCTCT AACGTCGAGA ACTTCGACTA   
  
  
- ATCAACAAGA GTTAGAGTAG TGAAGAAATG GGGAAAGCTG ACAATTGAAC GATACAACGA TTAATCGAAT   
  
  
- TCTATATTCT ATAAATCTTT GAGTTTTCAA TACTGTAAAG AACAACAGTT TCAATAGATA CAACTAATGA   
  
  
- CACCCATCAC CGATGTTTAT GTTATCGTTT AAGGTTTAAT AAATTTTGTG TTATAAACTA AACCTTTTGT   
  
  
- TAACTAAATT TCTCCTTGAA ACAATGTTTA TATTTTCAAT AATCGTAAAA GCCAAGTTTT ACGTATTCTA   
  
  
- CTCAACACAA AAAAAACAGC ACATGTGTAG TTTTTGACAG TATGCCTGTT ATTCGATCAG CGAAACTGGG   
  
  
- TAAGTGGCAT ACAGGGTTAC AGGAACTGGG ACGGTTTACT ATTCTTGCGG TCGTGTTTCC AAAATATTCA   
  
  
- AAGAGTTTCT CTTTGTTGTG TTACCCTTTT GTGTGGTAAC GAACTGAATG TTTACCTCTA GCCAACCTAG   
  
  
- TTAGAGACCT TTGTCCAACT TCATCTTAGA TTCGGGCAGA CCCGAGTACA GAGATGTGTC GTCTGGACTA   
  
  
- GTTTTGTTTA CCCTCTTACT CTTAGTGTAT TGAGACTTTC TCAGATAGAA AGATGCTCCA CCTAAGACAA   
  
  
- ATGGAAACCA GAGACGTCTA TCATCTACAA GGAACCGGTA CCGACCTCGC TAAAATTCGC AGTTTCGACG   
  
  
- TAGTGATCGT CACTGAGGTC AAGAATTTCA AAGCCAAAAT CAGTGTTCGC AAGA

+     ARE

| Site Name | Organism | Position | Strand | Matrix score. | sequence | function |
| --- | --- | --- | --- | --- | --- | --- |
| ARE | Zea mays | 284 | - | 6 | AAACCA | cis-acting regulatory element essential for the anaerobic induction |

>PlantCARE\_8972   
+ GAACTGTTAA AGCCAATCAT TTAAAATAAA AAGTCAAACC GGTTTAAACC GGTTTTCAAT AAGTTAATAA   
  
  
+ CCGGACTCGG TGTTAAAGAG ATTAAATTTA ACGGTTTAAC TCAGTGAGAT CGGTTTCGCT TTCTCTCTGT   
  
  
+ CGTGTTTGAG AAGTTGAAAG AGAGGAGGAT GAGCGCTGAA GATTTCCAGA AGAAGGTTTC GATCAGAGAC   
  
  
+ TCCTCCGTCG CCGGAGAAAT GGAAATCGAA TGCGGCGGGT CTACTAGCTC CGCGGTTGGT TCGTCTCGAA   
  
  
+ CCTTGGTTTT GCTTCGGAGA CTGCTCGAGA TTCAGGAGCG TCGAGCACAG GCCTACGCCA AACTCAAAAG   
  
  
+ GTTCGATCTT TGGAGTGTTT ATGCTATTAG TTACTGTTTT TGATTCAGTT ACTTGGTGAT TGTTACTGAG   
  
  
+ GGATGTTGGA GTTAAGTAAC TGATAGTGTT GTTGAGGAAG CGATGTCTCA TAAAGTTTAG ATTTTTATCT   
  
  
+ GTTGATCTCT TGCTATGCTT TTGGAATTTG ATTAATCAGA GGGTTCTCAG AGTATGTGGA GACTAGTGGT   
  
  
+ GAAGCGCTTT ATCAGAAGCT CTGCAGCGAG ATAACTGCTG AGTTCAACGA GTGTTCCAAA CAAGTAACTG   
  
  
+ AGTTTTTTTT TCTTCTTTTG ATTGATTACC TTTATGATGT TGTTGAAGAT ACGGTGTTAA AGATTGCGTC   
  
  
+ TTTGTTTGAT GGCAATGCAA GGTACGCGAA ATGGAAACTC TGTTTCTGAA TCCTGACGTT GGAAGATCGG   
  
  
+ ATCTTGCTCA ACTGCTCAGT GACATTCAAA CTCAGGAGAA GCAGAAACTG CATCTGGTCT GATTCCTATA   
  
  
+ TACTCAATTG TTGAACATAA CTGTTAACTT GTTGGACAGA ACAAAAGTTT CTTACTTTAG TTAGTTTTTT   
  
  
+ TTTTTTTTTG CAGACGGTTA CAATACAGGT ACTGAAGAAG GCAGGGAGGC CGTCAGAACG AATGCTGACA   
  
  
+ CACGAGAACT GCAAGTTCAA GAAACCGATG CAGCACGAGT GTGTGCATCT TCATGAGATT ACAGAAGCTG   
  
  
+ AAGGAACAGA GGAAGCAGAG GCGGATGCAG AGTTTGACAA CGCTTTGAAG GAAGCAATCA GAGGAGTGCA   
  
  
+ AGACGCTGTG ACTTGCATCA ATGAGTATTT GGAAGACGTT AGGTACGAGA TTGCAGCTCT TGAAGCTGAT   
  
  
+ TAGTTGTTCT CAATCTCATC ACTTCTTTAC CCCTTTCGAC TGTTAACTTG CTATGTTGCT AATTAGCTTA   
  
  
+ AGATATAAGA TATTTAGAAA CTCAAAAGTT ATGACATTTC TTGTTGTCAA AGTTATCTAT GTTGATTACT   
  
  
+ GTGGGTAGTG GCTACAAATA CAATAGCAAA TTCCAAATTA TTTAAAACAC AATATTTGAT TTGGAAAACA   
  
  
+ ATTGATTTAA AGAGGAACTT TGTTACAAAT ATAAAAGTTA TTAGCATTTT CGGTTCAAAA TGCATAAGAT   
  
  
+ GAGTTGTGTT TTTTTTGTCG TGTACACATC AAAAACTGTC ATACGGACAA TAAGCTAGTC GCTTTGACCC   
  
  
+ ATTCACCGTA TGTCCCAATG TCCTTGACCC TGCCAAATGA TAAGAACGCC AGCACAAAGG TTTTATAAGT   
  
  
+ TTCTCAAAGA GAAACAACAC AATGGGAAAA CACACCATTG CTTGACTTAC AAATGGAGAT CGGTTGGATC   
  
  
+ AATCTCTGGA AACAGGTTGA AGTAGAATCT AAGCCCGTCT GGGCTCATGT CTCTACACAG CAGACCTGAT   
  
  
+ CAAAACAAAT GGGAGAATGA GAATCACATA ACTCTGAAAG AGTCTATCTT TCTACGAGGT GGATTCTGTT   
  
  
+ TACCTTTGGT CTCTGCAGAT AGTAGATGTT CCTTGGCCAT GGCTGGAGCG ATTTTAAGCG TCAAAGCTGC   
  
  
+ ATCACTAGCA GTGACTCCAG TTCTTAAAGT TTCGGTTTTA GTCACAAGCG TTCT  

- CTTGACAATT TCGGTTAGTA AATTTTATTT TTCAGTTTGG CCAAATTTGG CCAAAAGTTA TTCAATTATT   
  
  
- GGCCTGAGCC ACAATTTCTC TAATTTAAAT TGCCAAATTG AGTCACTCTA GCCAAAGCGA AAGAGAGACA   
  
  
- GCACAAACTC TTCAACTTTC TCTCCTCCTA CTCGCGACTT CTAAAGGTCT TCTTCCAAAG CTAGTCTCTG   
  
  
- AGGAGGCAGC GGCCTCTTTA CCTTTAGCTT ACGCCGCCCA GATGATCGAG GCGCCAACCA AGCAGAGCTT   
  
  
- GGAACCAAAA CGAAGCCTCT GACGAGCTCT AAGTCCTCGC AGCTCGTGTC CGGATGCGGT TTGAGTTTTC   
  
  
- CAAGCTAGAA ACCTCACAAA TACGATAATC AATGACAAAA ACTAAGTCAA TGAACCACTA ACAATGACTC   
  
  
- CCTACAACCT CAATTCATTG ACTATCACAA CAACTCCTTC GCTACAGAGT ATTTCAAATC TAAAAATAGA   
  
  
- CAACTAGAGA ACGATACGAA AACCTTAAAC TAATTAGTCT CCCAAGAGTC TCATACACCT CTGATCACCA   
  
  
- CTTCGCGAAA TAGTCTTCGA GACGTCGCTC TATTGACGAC TCAAGTTGCT CACAAGGTTT GTTCATTGAC   
  
  
- TCAAAAAAAA AGAAGAAAAC TAACTAATGG AAATACTACA ACAACTTCTA TGCCACAATT TCTAACGCAG   
  
  
- AAACAAACTA CCGTTACGTT CCATGCGCTT TACCTTTGAG ACAAAGACTT AGGACTGCAA CCTTCTAGCC   
  
  
- TAGAACGAGT TGACGAGTCA CTGTAAGTTT GAGTCCTCTT CGTCTTTGAC GTAGACCAGA CTAAGGATAT   
  
  
- ATGAGTTAAC AACTTGTATT GACAATTGAA CAACCTGTCT TGTTTTCAAA GAATGAAATC AATCAAAAAA   
  
  
- AAAAAAAAAC GTCTGCCAAT GTTATGTCCA TGACTTCTTC CGTCCCTCCG GCAGTCTTGC TTACGACTGT   
  
  
- GTGCTCTTGA CGTTCAAGTT CTTTGGCTAC GTCGTGCTCA CACACGTAGA AGTACTCTAA TGTCTTCGAC   
  
  
- TTCCTTGTCT CCTTCGTCTC CGCCTACGTC TCAAACTGTT GCGAAACTTC CTTCGTTAGT CTCCTCACGT   
  
  
- TCTGCGACAC TGAACGTAGT TACTCATAAA CCTTCTGCAA TCCATGCTCT AACGTCGAGA ACTTCGACTA   
  
  
- ATCAACAAGA GTTAGAGTAG TGAAGAAATG GGGAAAGCTG ACAATTGAAC GATACAACGA TTAATCGAAT   
  
  
- TCTATATTCT ATAAATCTTT GAGTTTTCAA TACTGTAAAG AACAACAGTT TCAATAGATA CAACTAATGA   
  
  
- CACCCATCAC CGATGTTTAT GTTATCGTTT AAGGTTTAAT AAATTTTGTG TTATAAACTA AACCTTTTGT   
  
  
- TAACTAAATT TCTCCTTGAA ACAATGTTTA TATTTTCAAT AATCGTAAAA GCCAAGTTTT ACGTATTCTA   
  
  
- CTCAACACAA AAAAAACAGC ACATGTGTAG TTTTTGACAG TATGCCTGTT ATTCGATCAG CGAAACTGGG   
  
  
- TAAGTGGCAT ACAGGGTTAC AGGAACTGGG ACGGTTTACT ATTCTTGCGG TCGTGTTTCC AAAATATTCA   
  
  
- AAGAGTTTCT CTTTGTTGTG TTACCCTTTT GTGTGGTAAC GAACTGAATG TTTACCTCTA GCCAACCTAG   
  
  
- TTAGAGACCT TTGTCCAACT TCATCTTAGA TTCGGGCAGA CCCGAGTACA GAGATGTGTC GTCTGGACTA   
  
  
- GTTTTGTTTA CCCTCTTACT CTTAGTGTAT TGAGACTTTC TCAGATAGAA AGATGCTCCA CCTAAGACAA   
  
  
- ATGGAAACCA GAGACGTCTA TCATCTACAA GGAACCGGTA CCGACCTCGC TAAAATTCGC AGTTTCGACG   
  
  
- TAGTGATCGT CACTGAGGTC AAGAATTTCA AAGCCAAAAT CAGTGTTCGC AAGA

+     ATCT-motif

| Site Name | Organism | Position | Strand | Matrix score. | sequence | function |
| --- | --- | --- | --- | --- | --- | --- |
| ATCT-motif | Pisum sativum | 1706 | + | 9 | AATCTAATCC | part of a conserved DNA module involved in light responsiveness |

>PlantCARE\_8972   
+ GAACTGTTAA AGCCAATCAT TTAAAATAAA AAGTCAAACC GGTTTAAACC GGTTTTCAAT AAGTTAATAA   
  
  
+ CCGGACTCGG TGTTAAAGAG ATTAAATTTA ACGGTTTAAC TCAGTGAGAT CGGTTTCGCT TTCTCTCTGT   
  
  
+ CGTGTTTGAG AAGTTGAAAG AGAGGAGGAT GAGCGCTGAA GATTTCCAGA AGAAGGTTTC GATCAGAGAC   
  
  
+ TCCTCCGTCG CCGGAGAAAT GGAAATCGAA TGCGGCGGGT CTACTAGCTC CGCGGTTGGT TCGTCTCGAA   
  
  
+ CCTTGGTTTT GCTTCGGAGA CTGCTCGAGA TTCAGGAGCG TCGAGCACAG GCCTACGCCA AACTCAAAAG   
  
  
+ GTTCGATCTT TGGAGTGTTT ATGCTATTAG TTACTGTTTT TGATTCAGTT ACTTGGTGAT TGTTACTGAG   
  
  
+ GGATGTTGGA GTTAAGTAAC TGATAGTGTT GTTGAGGAAG CGATGTCTCA TAAAGTTTAG ATTTTTATCT   
  
  
+ GTTGATCTCT TGCTATGCTT TTGGAATTTG ATTAATCAGA GGGTTCTCAG AGTATGTGGA GACTAGTGGT   
  
  
+ GAAGCGCTTT ATCAGAAGCT CTGCAGCGAG ATAACTGCTG AGTTCAACGA GTGTTCCAAA CAAGTAACTG   
  
  
+ AGTTTTTTTT TCTTCTTTTG ATTGATTACC TTTATGATGT TGTTGAAGAT ACGGTGTTAA AGATTGCGTC   
  
  
+ TTTGTTTGAT GGCAATGCAA GGTACGCGAA ATGGAAACTC TGTTTCTGAA TCCTGACGTT GGAAGATCGG   
  
  
+ ATCTTGCTCA ACTGCTCAGT GACATTCAAA CTCAGGAGAA GCAGAAACTG CATCTGGTCT GATTCCTATA   
  
  
+ TACTCAATTG TTGAACATAA CTGTTAACTT GTTGGACAGA ACAAAAGTTT CTTACTTTAG TTAGTTTTTT   
  
  
+ TTTTTTTTTG CAGACGGTTA CAATACAGGT ACTGAAGAAG GCAGGGAGGC CGTCAGAACG AATGCTGACA   
  
  
+ CACGAGAACT GCAAGTTCAA GAAACCGATG CAGCACGAGT GTGTGCATCT TCATGAGATT ACAGAAGCTG   
  
  
+ AAGGAACAGA GGAAGCAGAG GCGGATGCAG AGTTTGACAA CGCTTTGAAG GAAGCAATCA GAGGAGTGCA   
  
  
+ AGACGCTGTG ACTTGCATCA ATGAGTATTT GGAAGACGTT AGGTACGAGA TTGCAGCTCT TGAAGCTGAT   
  
  
+ TAGTTGTTCT CAATCTCATC ACTTCTTTAC CCCTTTCGAC TGTTAACTTG CTATGTTGCT AATTAGCTTA   
  
  
+ AGATATAAGA TATTTAGAAA CTCAAAAGTT ATGACATTTC TTGTTGTCAA AGTTATCTAT GTTGATTACT   
  
  
+ GTGGGTAGTG GCTACAAATA CAATAGCAAA TTCCAAATTA TTTAAAACAC AATATTTGAT TTGGAAAACA   
  
  
+ ATTGATTTAA AGAGGAACTT TGTTACAAAT ATAAAAGTTA TTAGCATTTT CGGTTCAAAA TGCATAAGAT   
  
  
+ GAGTTGTGTT TTTTTTGTCG TGTACACATC AAAAACTGTC ATACGGACAA TAAGCTAGTC GCTTTGACCC   
  
  
+ ATTCACCGTA TGTCCCAATG TCCTTGACCC TGCCAAATGA TAAGAACGCC AGCACAAAGG TTTTATAAGT   
  
  
+ TTCTCAAAGA GAAACAACAC AATGGGAAAA CACACCATTG CTTGACTTAC AAATGGAGAT CGGTTGGATC   
  
  
+ AATCTCTGGA AACAGGTTGA AGTAGAATCT AAGCCCGTCT GGGCTCATGT CTCTACACAG CAGACCTGAT   
  
  
+ CAAAACAAAT GGGAGAATGA GAATCACATA ACTCTGAAAG AGTCTATCTT TCTACGAGGT GGATTCTGTT   
  
  
+ TACCTTTGGT CTCTGCAGAT AGTAGATGTT CCTTGGCCAT GGCTGGAGCG ATTTTAAGCG TCAAAGCTGC   
  
  
+ ATCACTAGCA GTGACTCCAG TTCTTAAAGT TTCGGTTTTA GTCACAAGCG TTCT  

- CTTGACAATT TCGGTTAGTA AATTTTATTT TTCAGTTTGG CCAAATTTGG CCAAAAGTTA TTCAATTATT   
  
  
- GGCCTGAGCC ACAATTTCTC TAATTTAAAT TGCCAAATTG AGTCACTCTA GCCAAAGCGA AAGAGAGACA   
  
  
- GCACAAACTC TTCAACTTTC TCTCCTCCTA CTCGCGACTT CTAAAGGTCT TCTTCCAAAG CTAGTCTCTG   
  
  
- AGGAGGCAGC GGCCTCTTTA CCTTTAGCTT ACGCCGCCCA GATGATCGAG GCGCCAACCA AGCAGAGCTT   
  
  
- GGAACCAAAA CGAAGCCTCT GACGAGCTCT AAGTCCTCGC AGCTCGTGTC CGGATGCGGT TTGAGTTTTC   
  
  
- CAAGCTAGAA ACCTCACAAA TACGATAATC AATGACAAAA ACTAAGTCAA TGAACCACTA ACAATGACTC   
  
  
- CCTACAACCT CAATTCATTG ACTATCACAA CAACTCCTTC GCTACAGAGT ATTTCAAATC TAAAAATAGA   
  
  
- CAACTAGAGA ACGATACGAA AACCTTAAAC TAATTAGTCT CCCAAGAGTC TCATACACCT CTGATCACCA   
  
  
- CTTCGCGAAA TAGTCTTCGA GACGTCGCTC TATTGACGAC TCAAGTTGCT CACAAGGTTT GTTCATTGAC   
  
  
- TCAAAAAAAA AGAAGAAAAC TAACTAATGG AAATACTACA ACAACTTCTA TGCCACAATT TCTAACGCAG   
  
  
- AAACAAACTA CCGTTACGTT CCATGCGCTT TACCTTTGAG ACAAAGACTT AGGACTGCAA CCTTCTAGCC   
  
  
- TAGAACGAGT TGACGAGTCA CTGTAAGTTT GAGTCCTCTT CGTCTTTGAC GTAGACCAGA CTAAGGATAT   
  
  
- ATGAGTTAAC AACTTGTATT GACAATTGAA CAACCTGTCT TGTTTTCAAA GAATGAAATC AATCAAAAAA   
  
  
- AAAAAAAAAC GTCTGCCAAT GTTATGTCCA TGACTTCTTC CGTCCCTCCG GCAGTCTTGC TTACGACTGT   
  
  
- GTGCTCTTGA CGTTCAAGTT CTTTGGCTAC GTCGTGCTCA CACACGTAGA AGTACTCTAA TGTCTTCGAC   
  
  
- TTCCTTGTCT CCTTCGTCTC CGCCTACGTC TCAAACTGTT GCGAAACTTC CTTCGTTAGT CTCCTCACGT   
  
  
- TCTGCGACAC TGAACGTAGT TACTCATAAA CCTTCTGCAA TCCATGCTCT AACGTCGAGA ACTTCGACTA   
  
  
- ATCAACAAGA GTTAGAGTAG TGAAGAAATG GGGAAAGCTG ACAATTGAAC GATACAACGA TTAATCGAAT   
  
  
- TCTATATTCT ATAAATCTTT GAGTTTTCAA TACTGTAAAG AACAACAGTT TCAATAGATA CAACTAATGA   
  
  
- CACCCATCAC CGATGTTTAT GTTATCGTTT AAGGTTTAAT AAATTTTGTG TTATAAACTA AACCTTTTGT   
  
  
- TAACTAAATT TCTCCTTGAA ACAATGTTTA TATTTTCAAT AATCGTAAAA GCCAAGTTTT ACGTATTCTA   
  
  
- CTCAACACAA AAAAAACAGC ACATGTGTAG TTTTTGACAG TATGCCTGTT ATTCGATCAG CGAAACTGGG   
  
  
- TAAGTGGCAT ACAGGGTTAC AGGAACTGGG ACGGTTTACT ATTCTTGCGG TCGTGTTTCC AAAATATTCA   
  
  
- AAGAGTTTCT CTTTGTTGTG TTACCCTTTT GTGTGGTAAC GAACTGAATG TTTACCTCTA GCCAACCTAG   
  
  
- TTAGAGACCT TTGTCCAACT TCATCTTAGA TTCGGGCAGA CCCGAGTACA GAGATGTGTC GTCTGGACTA   
  
  
- GTTTTGTTTA CCCTCTTACT CTTAGTGTAT TGAGACTTTC TCAGATAGAA AGATGCTCCA CCTAAGACAA   
  
  
- ATGGAAACCA GAGACGTCTA TCATCTACAA GGAACCGGTA CCGACCTCGC TAAAATTCGC AGTTTCGACG   
  
  
- TAGTGATCGT CACTGAGGTC AAGAATTTCA AAGCCAAAAT CAGTGTTCGC AAGA

+     AT~TATA-box

| Site Name | Organism | Position | Strand | Matrix score. | sequence | function |
| --- | --- | --- | --- | --- | --- | --- |
| AT~TATA-box | Arabidopsis thaliana | 837 | + | 6 | TATATA |  |

>PlantCARE\_8972   
+ GAACTGTTAA AGCCAATCAT TTAAAATAAA AAGTCAAACC GGTTTAAACC GGTTTTCAAT AAGTTAATAA   
  
  
+ CCGGACTCGG TGTTAAAGAG ATTAAATTTA ACGGTTTAAC TCAGTGAGAT CGGTTTCGCT TTCTCTCTGT   
  
  
+ CGTGTTTGAG AAGTTGAAAG AGAGGAGGAT GAGCGCTGAA GATTTCCAGA AGAAGGTTTC GATCAGAGAC   
  
  
+ TCCTCCGTCG CCGGAGAAAT GGAAATCGAA TGCGGCGGGT CTACTAGCTC CGCGGTTGGT TCGTCTCGAA   
  
  
+ CCTTGGTTTT GCTTCGGAGA CTGCTCGAGA TTCAGGAGCG TCGAGCACAG GCCTACGCCA AACTCAAAAG   
  
  
+ GTTCGATCTT TGGAGTGTTT ATGCTATTAG TTACTGTTTT TGATTCAGTT ACTTGGTGAT TGTTACTGAG   
  
  
+ GGATGTTGGA GTTAAGTAAC TGATAGTGTT GTTGAGGAAG CGATGTCTCA TAAAGTTTAG ATTTTTATCT   
  
  
+ GTTGATCTCT TGCTATGCTT TTGGAATTTG ATTAATCAGA GGGTTCTCAG AGTATGTGGA GACTAGTGGT   
  
  
+ GAAGCGCTTT ATCAGAAGCT CTGCAGCGAG ATAACTGCTG AGTTCAACGA GTGTTCCAAA CAAGTAACTG   
  
  
+ AGTTTTTTTT TCTTCTTTTG ATTGATTACC TTTATGATGT TGTTGAAGAT ACGGTGTTAA AGATTGCGTC   
  
  
+ TTTGTTTGAT GGCAATGCAA GGTACGCGAA ATGGAAACTC TGTTTCTGAA TCCTGACGTT GGAAGATCGG   
  
  
+ ATCTTGCTCA ACTGCTCAGT GACATTCAAA CTCAGGAGAA GCAGAAACTG CATCTGGTCT GATTCCTATA   
  
  
+ TACTCAATTG TTGAACATAA CTGTTAACTT GTTGGACAGA ACAAAAGTTT CTTACTTTAG TTAGTTTTTT   
  
  
+ TTTTTTTTTG CAGACGGTTA CAATACAGGT ACTGAAGAAG GCAGGGAGGC CGTCAGAACG AATGCTGACA   
  
  
+ CACGAGAACT GCAAGTTCAA GAAACCGATG CAGCACGAGT GTGTGCATCT TCATGAGATT ACAGAAGCTG   
  
  
+ AAGGAACAGA GGAAGCAGAG GCGGATGCAG AGTTTGACAA CGCTTTGAAG GAAGCAATCA GAGGAGTGCA   
  
  
+ AGACGCTGTG ACTTGCATCA ATGAGTATTT GGAAGACGTT AGGTACGAGA TTGCAGCTCT TGAAGCTGAT   
  
  
+ TAGTTGTTCT CAATCTCATC ACTTCTTTAC CCCTTTCGAC TGTTAACTTG CTATGTTGCT AATTAGCTTA   
  
  
+ AGATATAAGA TATTTAGAAA CTCAAAAGTT ATGACATTTC TTGTTGTCAA AGTTATCTAT GTTGATTACT   
  
  
+ GTGGGTAGTG GCTACAAATA CAATAGCAAA TTCCAAATTA TTTAAAACAC AATATTTGAT TTGGAAAACA   
  
  
+ ATTGATTTAA AGAGGAACTT TGTTACAAAT ATAAAAGTTA TTAGCATTTT CGGTTCAAAA TGCATAAGAT   
  
  
+ GAGTTGTGTT TTTTTTGTCG TGTACACATC AAAAACTGTC ATACGGACAA TAAGCTAGTC GCTTTGACCC   
  
  
+ ATTCACCGTA TGTCCCAATG TCCTTGACCC TGCCAAATGA TAAGAACGCC AGCACAAAGG TTTTATAAGT   
  
  
+ TTCTCAAAGA GAAACAACAC AATGGGAAAA CACACCATTG CTTGACTTAC AAATGGAGAT CGGTTGGATC   
  
  
+ AATCTCTGGA AACAGGTTGA AGTAGAATCT AAGCCCGTCT GGGCTCATGT CTCTACACAG CAGACCTGAT   
  
  
+ CAAAACAAAT GGGAGAATGA GAATCACATA ACTCTGAAAG AGTCTATCTT TCTACGAGGT GGATTCTGTT   
  
  
+ TACCTTTGGT CTCTGCAGAT AGTAGATGTT CCTTGGCCAT GGCTGGAGCG ATTTTAAGCG TCAAAGCTGC   
  
  
+ ATCACTAGCA GTGACTCCAG TTCTTAAAGT TTCGGTTTTA GTCACAAGCG TTCT  

- CTTGACAATT TCGGTTAGTA AATTTTATTT TTCAGTTTGG CCAAATTTGG CCAAAAGTTA TTCAATTATT   
  
  
- GGCCTGAGCC ACAATTTCTC TAATTTAAAT TGCCAAATTG AGTCACTCTA GCCAAAGCGA AAGAGAGACA   
  
  
- GCACAAACTC TTCAACTTTC TCTCCTCCTA CTCGCGACTT CTAAAGGTCT TCTTCCAAAG CTAGTCTCTG   
  
  
- AGGAGGCAGC GGCCTCTTTA CCTTTAGCTT ACGCCGCCCA GATGATCGAG GCGCCAACCA AGCAGAGCTT   
  
  
- GGAACCAAAA CGAAGCCTCT GACGAGCTCT AAGTCCTCGC AGCTCGTGTC CGGATGCGGT TTGAGTTTTC   
  
  
- CAAGCTAGAA ACCTCACAAA TACGATAATC AATGACAAAA ACTAAGTCAA TGAACCACTA ACAATGACTC   
  
  
- CCTACAACCT CAATTCATTG ACTATCACAA CAACTCCTTC GCTACAGAGT ATTTCAAATC TAAAAATAGA   
  
  
- CAACTAGAGA ACGATACGAA AACCTTAAAC TAATTAGTCT CCCAAGAGTC TCATACACCT CTGATCACCA   
  
  
- CTTCGCGAAA TAGTCTTCGA GACGTCGCTC TATTGACGAC TCAAGTTGCT CACAAGGTTT GTTCATTGAC   
  
  
- TCAAAAAAAA AGAAGAAAAC TAACTAATGG AAATACTACA ACAACTTCTA TGCCACAATT TCTAACGCAG   
  
  
- AAACAAACTA CCGTTACGTT CCATGCGCTT TACCTTTGAG ACAAAGACTT AGGACTGCAA CCTTCTAGCC   
  
  
- TAGAACGAGT TGACGAGTCA CTGTAAGTTT GAGTCCTCTT CGTCTTTGAC GTAGACCAGA CTAAGGATAT   
  
  
- ATGAGTTAAC AACTTGTATT GACAATTGAA CAACCTGTCT TGTTTTCAAA GAATGAAATC AATCAAAAAA   
  
  
- AAAAAAAAAC GTCTGCCAAT GTTATGTCCA TGACTTCTTC CGTCCCTCCG GCAGTCTTGC TTACGACTGT   
  
  
- GTGCTCTTGA CGTTCAAGTT CTTTGGCTAC GTCGTGCTCA CACACGTAGA AGTACTCTAA TGTCTTCGAC   
  
  
- TTCCTTGTCT CCTTCGTCTC CGCCTACGTC TCAAACTGTT GCGAAACTTC CTTCGTTAGT CTCCTCACGT   
  
  
- TCTGCGACAC TGAACGTAGT TACTCATAAA CCTTCTGCAA TCCATGCTCT AACGTCGAGA ACTTCGACTA   
  
  
- ATCAACAAGA GTTAGAGTAG TGAAGAAATG GGGAAAGCTG ACAATTGAAC GATACAACGA TTAATCGAAT   
  
  
- TCTATATTCT ATAAATCTTT GAGTTTTCAA TACTGTAAAG AACAACAGTT TCAATAGATA CAACTAATGA   
  
  
- CACCCATCAC CGATGTTTAT GTTATCGTTT AAGGTTTAAT AAATTTTGTG TTATAAACTA AACCTTTTGT   
  
  
- TAACTAAATT TCTCCTTGAA ACAATGTTTA TATTTTCAAT AATCGTAAAA GCCAAGTTTT ACGTATTCTA   
  
  
- CTCAACACAA AAAAAACAGC ACATGTGTAG TTTTTGACAG TATGCCTGTT ATTCGATCAG CGAAACTGGG   
  
  
- TAAGTGGCAT ACAGGGTTAC AGGAACTGGG ACGGTTTACT ATTCTTGCGG TCGTGTTTCC AAAATATTCA   
  
  
- AAGAGTTTCT CTTTGTTGTG TTACCCTTTT GTGTGGTAAC GAACTGAATG TTTACCTCTA GCCAACCTAG   
  
  
- TTAGAGACCT TTGTCCAACT TCATCTTAGA TTCGGGCAGA CCCGAGTACA GAGATGTGTC GTCTGGACTA   
  
  
- GTTTTGTTTA CCCTCTTACT CTTAGTGTAT TGAGACTTTC TCAGATAGAA AGATGCTCCA CCTAAGACAA   
  
  
- ATGGAAACCA GAGACGTCTA TCATCTACAA GGAACCGGTA CCGACCTCGC TAAAATTCGC AGTTTCGACG   
  
  
- TAGTGATCGT CACTGAGGTC AAGAATTTCA AAGCCAAAAT CAGTGTTCGC AAGA

+     Box 4

| Site Name | Organism | Position | Strand | Matrix score. | sequence | function |
| --- | --- | --- | --- | --- | --- | --- |
| Box 4 | Petroselinum crispum | 521 | + | 6 | ATTAAT | part of a conserved DNA module involved in light responsiveness |

>PlantCARE\_8972   
+ GAACTGTTAA AGCCAATCAT TTAAAATAAA AAGTCAAACC GGTTTAAACC GGTTTTCAAT AAGTTAATAA   
  
  
+ CCGGACTCGG TGTTAAAGAG ATTAAATTTA ACGGTTTAAC TCAGTGAGAT CGGTTTCGCT TTCTCTCTGT   
  
  
+ CGTGTTTGAG AAGTTGAAAG AGAGGAGGAT GAGCGCTGAA GATTTCCAGA AGAAGGTTTC GATCAGAGAC   
  
  
+ TCCTCCGTCG CCGGAGAAAT GGAAATCGAA TGCGGCGGGT CTACTAGCTC CGCGGTTGGT TCGTCTCGAA   
  
  
+ CCTTGGTTTT GCTTCGGAGA CTGCTCGAGA TTCAGGAGCG TCGAGCACAG GCCTACGCCA AACTCAAAAG   
  
  
+ GTTCGATCTT TGGAGTGTTT ATGCTATTAG TTACTGTTTT TGATTCAGTT ACTTGGTGAT TGTTACTGAG   
  
  
+ GGATGTTGGA GTTAAGTAAC TGATAGTGTT GTTGAGGAAG CGATGTCTCA TAAAGTTTAG ATTTTTATCT   
  
  
+ GTTGATCTCT TGCTATGCTT TTGGAATTTG ATTAATCAGA GGGTTCTCAG AGTATGTGGA GACTAGTGGT   
  
  
+ GAAGCGCTTT ATCAGAAGCT CTGCAGCGAG ATAACTGCTG AGTTCAACGA GTGTTCCAAA CAAGTAACTG   
  
  
+ AGTTTTTTTT TCTTCTTTTG ATTGATTACC TTTATGATGT TGTTGAAGAT ACGGTGTTAA AGATTGCGTC   
  
  
+ TTTGTTTGAT GGCAATGCAA GGTACGCGAA ATGGAAACTC TGTTTCTGAA TCCTGACGTT GGAAGATCGG   
  
  
+ ATCTTGCTCA ACTGCTCAGT GACATTCAAA CTCAGGAGAA GCAGAAACTG CATCTGGTCT GATTCCTATA   
  
  
+ TACTCAATTG TTGAACATAA CTGTTAACTT GTTGGACAGA ACAAAAGTTT CTTACTTTAG TTAGTTTTTT   
  
  
+ TTTTTTTTTG CAGACGGTTA CAATACAGGT ACTGAAGAAG GCAGGGAGGC CGTCAGAACG AATGCTGACA   
  
  
+ CACGAGAACT GCAAGTTCAA GAAACCGATG CAGCACGAGT GTGTGCATCT TCATGAGATT ACAGAAGCTG   
  
  
+ AAGGAACAGA GGAAGCAGAG GCGGATGCAG AGTTTGACAA CGCTTTGAAG GAAGCAATCA GAGGAGTGCA   
  
  
+ AGACGCTGTG ACTTGCATCA ATGAGTATTT GGAAGACGTT AGGTACGAGA TTGCAGCTCT TGAAGCTGAT   
  
  
+ TAGTTGTTCT CAATCTCATC ACTTCTTTAC CCCTTTCGAC TGTTAACTTG CTATGTTGCT AATTAGCTTA   
  
  
+ AGATATAAGA TATTTAGAAA CTCAAAAGTT ATGACATTTC TTGTTGTCAA AGTTATCTAT GTTGATTACT   
  
  
+ GTGGGTAGTG GCTACAAATA CAATAGCAAA TTCCAAATTA TTTAAAACAC AATATTTGAT TTGGAAAACA   
  
  
+ ATTGATTTAA AGAGGAACTT TGTTACAAAT ATAAAAGTTA TTAGCATTTT CGGTTCAAAA TGCATAAGAT   
  
  
+ GAGTTGTGTT TTTTTTGTCG TGTACACATC AAAAACTGTC ATACGGACAA TAAGCTAGTC GCTTTGACCC   
  
  
+ ATTCACCGTA TGTCCCAATG TCCTTGACCC TGCCAAATGA TAAGAACGCC AGCACAAAGG TTTTATAAGT   
  
  
+ TTCTCAAAGA GAAACAACAC AATGGGAAAA CACACCATTG CTTGACTTAC AAATGGAGAT CGGTTGGATC   
  
  
+ AATCTCTGGA AACAGGTTGA AGTAGAATCT AAGCCCGTCT GGGCTCATGT CTCTACACAG CAGACCTGAT   
  
  
+ CAAAACAAAT GGGAGAATGA GAATCACATA ACTCTGAAAG AGTCTATCTT TCTACGAGGT GGATTCTGTT   
  
  
+ TACCTTTGGT CTCTGCAGAT AGTAGATGTT CCTTGGCCAT GGCTGGAGCG ATTTTAAGCG TCAAAGCTGC   
  
  
+ ATCACTAGCA GTGACTCCAG TTCTTAAAGT TTCGGTTTTA GTCACAAGCG TTCT  

- CTTGACAATT TCGGTTAGTA AATTTTATTT TTCAGTTTGG CCAAATTTGG CCAAAAGTTA TTCAATTATT   
  
  
- GGCCTGAGCC ACAATTTCTC TAATTTAAAT TGCCAAATTG AGTCACTCTA GCCAAAGCGA AAGAGAGACA   
  
  
- GCACAAACTC TTCAACTTTC TCTCCTCCTA CTCGCGACTT CTAAAGGTCT TCTTCCAAAG CTAGTCTCTG   
  
  
- AGGAGGCAGC GGCCTCTTTA CCTTTAGCTT ACGCCGCCCA GATGATCGAG GCGCCAACCA AGCAGAGCTT   
  
  
- GGAACCAAAA CGAAGCCTCT GACGAGCTCT AAGTCCTCGC AGCTCGTGTC CGGATGCGGT TTGAGTTTTC   
  
  
- CAAGCTAGAA ACCTCACAAA TACGATAATC AATGACAAAA ACTAAGTCAA TGAACCACTA ACAATGACTC   
  
  
- CCTACAACCT CAATTCATTG ACTATCACAA CAACTCCTTC GCTACAGAGT ATTTCAAATC TAAAAATAGA   
  
  
- CAACTAGAGA ACGATACGAA AACCTTAAAC TAATTAGTCT CCCAAGAGTC TCATACACCT CTGATCACCA   
  
  
- CTTCGCGAAA TAGTCTTCGA GACGTCGCTC TATTGACGAC TCAAGTTGCT CACAAGGTTT GTTCATTGAC   
  
  
- TCAAAAAAAA AGAAGAAAAC TAACTAATGG AAATACTACA ACAACTTCTA TGCCACAATT TCTAACGCAG   
  
  
- AAACAAACTA CCGTTACGTT CCATGCGCTT TACCTTTGAG ACAAAGACTT AGGACTGCAA CCTTCTAGCC   
  
  
- TAGAACGAGT TGACGAGTCA CTGTAAGTTT GAGTCCTCTT CGTCTTTGAC GTAGACCAGA CTAAGGATAT   
  
  
- ATGAGTTAAC AACTTGTATT GACAATTGAA CAACCTGTCT TGTTTTCAAA GAATGAAATC AATCAAAAAA   
  
  
- AAAAAAAAAC GTCTGCCAAT GTTATGTCCA TGACTTCTTC CGTCCCTCCG GCAGTCTTGC TTACGACTGT   
  
  
- GTGCTCTTGA CGTTCAAGTT CTTTGGCTAC GTCGTGCTCA CACACGTAGA AGTACTCTAA TGTCTTCGAC   
  
  
- TTCCTTGTCT CCTTCGTCTC CGCCTACGTC TCAAACTGTT GCGAAACTTC CTTCGTTAGT CTCCTCACGT   
  
  
- TCTGCGACAC TGAACGTAGT TACTCATAAA CCTTCTGCAA TCCATGCTCT AACGTCGAGA ACTTCGACTA   
  
  
- ATCAACAAGA GTTAGAGTAG TGAAGAAATG GGGAAAGCTG ACAATTGAAC GATACAACGA TTAATCGAAT   
  
  
- TCTATATTCT ATAAATCTTT GAGTTTTCAA TACTGTAAAG AACAACAGTT TCAATAGATA CAACTAATGA   
  
  
- CACCCATCAC CGATGTTTAT GTTATCGTTT AAGGTTTAAT AAATTTTGTG TTATAAACTA AACCTTTTGT   
  
  
- TAACTAAATT TCTCCTTGAA ACAATGTTTA TATTTTCAAT AATCGTAAAA GCCAAGTTTT ACGTATTCTA   
  
  
- CTCAACACAA AAAAAACAGC ACATGTGTAG TTTTTGACAG TATGCCTGTT ATTCGATCAG CGAAACTGGG   
  
  
- TAAGTGGCAT ACAGGGTTAC AGGAACTGGG ACGGTTTACT ATTCTTGCGG TCGTGTTTCC AAAATATTCA   
  
  
- AAGAGTTTCT CTTTGTTGTG TTACCCTTTT GTGTGGTAAC GAACTGAATG TTTACCTCTA GCCAACCTAG   
  
  
- TTAGAGACCT TTGTCCAACT TCATCTTAGA TTCGGGCAGA CCCGAGTACA GAGATGTGTC GTCTGGACTA   
  
  
- GTTTTGTTTA CCCTCTTACT CTTAGTGTAT TGAGACTTTC TCAGATAGAA AGATGCTCCA CCTAAGACAA   
  
  
- ATGGAAACCA GAGACGTCTA TCATCTACAA GGAACCGGTA CCGACCTCGC TAAAATTCGC AGTTTCGACG   
  
  
- TAGTGATCGT CACTGAGGTC AAGAATTTCA AAGCCAAAAT CAGTGTTCGC AAGA

+     CAAT-box

| Site Name | Organism | Position | Strand | Matrix score. | sequence | function |
| --- | --- | --- | --- | --- | --- | --- |
| CAAT-box | Arabidopsis thaliana | 13 | + | 5 | CCAAT | common cis-acting element in promoter and enhancer regions |
| CAAT-box | Nicotiana glutinosa | 14 | + | 4 | CAAT |  |
| CAAT-box | Nicotiana glutinosa | 57 | + | 4 | CAAT |  |
| CAAT-box | Nicotiana glutinosa | 409 | - | 4 | CAAT |  |
| CAAT-box | Pisum sativum | 516 | - | 5 | CAAAT | common cis-acting element in promoter and enhancer regions |
| CAAT-box | Nicotiana glutinosa | 651 | - | 4 | CAAT |  |
| CAAT-box | Nicotiana glutinosa | 693 | - | 4 | CAAT |  |
| CAAT-box | Nicotiana glutinosa | 713 | + | 4 | CAAT |  |
| CAAT-box | Nicotiana glutinosa | 845 | + | 4 | CAAT |  |
| CAAT-box | Nicotiana glutinosa | 847 | - | 4 | CAAT |  |
| CAAT-box | Nicotiana glutinosa | 931 | + | 4 | CAAT |  |
| CAAT-box | Nicotiana glutinosa | 1105 | + | 4 | CAAT |  |
| CAAT-box | Nicotiana glutinosa | 1139 | + | 4 | CAAT |  |
| CAAT-box | Pisum sativum | 1147 | - | 5 | CAAAT | common cis-acting element in promoter and enhancer regions |
| CAAT-box | Nicotiana glutinosa | 1170 | - | 4 | CAAT |  |
| CAAT-box | Nicotiana glutinosa | 1201 | + | 4 | CAAT |  |
| CAAT-box | Pisum sativum | 1345 | + | 5 | CAAAT | common cis-acting element in promoter and enhancer regions |
| CAAT-box | Nicotiana glutinosa | 1351 | + | 4 | CAAT |  |
| CAAT-box | Pisum sativum | 1357 | + | 5 | CAAAT | common cis-acting element in promoter and enhancer regions |
| CAAT-box | Pisum sativum | 1364 | + | 5 | CAAAT | common cis-acting element in promoter and enhancer regions |
| CAAT-box | Nicotiana glutinosa | 1380 | + | 4 | CAAT |  |
| CAAT-box | Pisum sativum | 1384 | - | 5 | CAAAT | common cis-acting element in promoter and enhancer regions |
| CAAT-box | Pisum sativum | 1389 | - | 5 | CAAAT | common cis-acting element in promoter and enhancer regions |
| CAAT-box | Nicotiana glutinosa | 1399 | + | 4 | CAAT |  |
| CAAT-box | Nicotiana glutinosa | 1401 | - | 4 | CAAT |  |
| CAAT-box | Pisum sativum | 1426 | + | 5 | CAAAT | common cis-acting element in promoter and enhancer regions |
| CAAT-box | Nicotiana glutinosa | 1518 | + | 4 | CAAT |  |
| CAAT-box | Arabidopsis thaliana | 1555 | + | 5 | CCAAT | common cis-acting element in promoter and enhancer regions |
| CAAT-box | Nicotiana glutinosa | 1556 | + | 4 | CAAT |  |
| CAAT-box | Pisum sativum | 1574 | + | 5 | CAAAT | common cis-acting element in promoter and enhancer regions |
| CAAT-box | Nicotiana glutinosa | 1630 | + | 4 | CAAT |  |
| CAAT-box | Nicotiana glutinosa | 1647 | - | 4 | CAAT |  |
| CAAT-box | Pisum sativum | 1660 | + | 5 | CAAAT | common cis-acting element in promoter and enhancer regions |
| CAAT-box | Nicotiana glutinosa | 1680 | + | 4 | CAAT |  |
| CAAT-box | Pisum sativum | 1756 | + | 5 | CAAAT | common cis-acting element in promoter and enhancer regions |

>PlantCARE\_8972   
+ GAACTGTTAA AGCCAATCAT TTAAAATAAA AAGTCAAACC GGTTTAAACC GGTTTTCAAT AAGTTAATAA   
  
  
+ CCGGACTCGG TGTTAAAGAG ATTAAATTTA ACGGTTTAAC TCAGTGAGAT CGGTTTCGCT TTCTCTCTGT   
  
  
+ CGTGTTTGAG AAGTTGAAAG AGAGGAGGAT GAGCGCTGAA GATTTCCAGA AGAAGGTTTC GATCAGAGAC   
  
  
+ TCCTCCGTCG CCGGAGAAAT GGAAATCGAA TGCGGCGGGT CTACTAGCTC CGCGGTTGGT TCGTCTCGAA   
  
  
+ CCTTGGTTTT GCTTCGGAGA CTGCTCGAGA TTCAGGAGCG TCGAGCACAG GCCTACGCCA AACTCAAAAG   
  
  
+ GTTCGATCTT TGGAGTGTTT ATGCTATTAG TTACTGTTTT TGATTCAGTT ACTTGGTGAT TGTTACTGAG   
  
  
+ GGATGTTGGA GTTAAGTAAC TGATAGTGTT GTTGAGGAAG CGATGTCTCA TAAAGTTTAG ATTTTTATCT   
  
  
+ GTTGATCTCT TGCTATGCTT TTGGAATTTG ATTAATCAGA GGGTTCTCAG AGTATGTGGA GACTAGTGGT   
  
  
+ GAAGCGCTTT ATCAGAAGCT CTGCAGCGAG ATAACTGCTG AGTTCAACGA GTGTTCCAAA CAAGTAACTG   
  
  
+ AGTTTTTTTT TCTTCTTTTG ATTGATTACC TTTATGATGT TGTTGAAGAT ACGGTGTTAA AGATTGCGTC   
  
  
+ TTTGTTTGAT GGCAATGCAA GGTACGCGAA ATGGAAACTC TGTTTCTGAA TCCTGACGTT GGAAGATCGG   
  
  
+ ATCTTGCTCA ACTGCTCAGT GACATTCAAA CTCAGGAGAA GCAGAAACTG CATCTGGTCT GATTCCTATA   
  
  
+ TACTCAATTG TTGAACATAA CTGTTAACTT GTTGGACAGA ACAAAAGTTT CTTACTTTAG TTAGTTTTTT   
  
  
+ TTTTTTTTTG CAGACGGTTA CAATACAGGT ACTGAAGAAG GCAGGGAGGC CGTCAGAACG AATGCTGACA   
  
  
+ CACGAGAACT GCAAGTTCAA GAAACCGATG CAGCACGAGT GTGTGCATCT TCATGAGATT ACAGAAGCTG   
  
  
+ AAGGAACAGA GGAAGCAGAG GCGGATGCAG AGTTTGACAA CGCTTTGAAG GAAGCAATCA GAGGAGTGCA   
  
  
+ AGACGCTGTG ACTTGCATCA ATGAGTATTT GGAAGACGTT AGGTACGAGA TTGCAGCTCT TGAAGCTGAT   
  
  
+ TAGTTGTTCT CAATCTCATC ACTTCTTTAC CCCTTTCGAC TGTTAACTTG CTATGTTGCT AATTAGCTTA   
  
  
+ AGATATAAGA TATTTAGAAA CTCAAAAGTT ATGACATTTC TTGTTGTCAA AGTTATCTAT GTTGATTACT   
  
  
+ GTGGGTAGTG GCTACAAATA CAATAGCAAA TTCCAAATTA TTTAAAACAC AATATTTGAT TTGGAAAACA   
  
  
+ ATTGATTTAA AGAGGAACTT TGTTACAAAT ATAAAAGTTA TTAGCATTTT CGGTTCAAAA TGCATAAGAT   
  
  
+ GAGTTGTGTT TTTTTTGTCG TGTACACATC AAAAACTGTC ATACGGACAA TAAGCTAGTC GCTTTGACCC   
  
  
+ ATTCACCGTA TGTCCCAATG TCCTTGACCC TGCCAAATGA TAAGAACGCC AGCACAAAGG TTTTATAAGT   
  
  
+ TTCTCAAAGA GAAACAACAC AATGGGAAAA CACACCATTG CTTGACTTAC AAATGGAGAT CGGTTGGATC   
  
  
+ AATCTCTGGA AACAGGTTGA AGTAGAATCT AAGCCCGTCT GGGCTCATGT CTCTACACAG CAGACCTGAT   
  
  
+ CAAAACAAAT GGGAGAATGA GAATCACATA ACTCTGAAAG AGTCTATCTT TCTACGAGGT GGATTCTGTT   
  
  
+ TACCTTTGGT CTCTGCAGAT AGTAGATGTT CCTTGGCCAT GGCTGGAGCG ATTTTAAGCG TCAAAGCTGC   
  
  
+ ATCACTAGCA GTGACTCCAG TTCTTAAAGT TTCGGTTTTA GTCACAAGCG TTCT  

- CTTGACAATT TCGGTTAGTA AATTTTATTT TTCAGTTTGG CCAAATTTGG CCAAAAGTTA TTCAATTATT   
  
  
- GGCCTGAGCC ACAATTTCTC TAATTTAAAT TGCCAAATTG AGTCACTCTA GCCAAAGCGA AAGAGAGACA   
  
  
- GCACAAACTC TTCAACTTTC TCTCCTCCTA CTCGCGACTT CTAAAGGTCT TCTTCCAAAG CTAGTCTCTG   
  
  
- AGGAGGCAGC GGCCTCTTTA CCTTTAGCTT ACGCCGCCCA GATGATCGAG GCGCCAACCA AGCAGAGCTT   
  
  
- GGAACCAAAA CGAAGCCTCT GACGAGCTCT AAGTCCTCGC AGCTCGTGTC CGGATGCGGT TTGAGTTTTC   
  
  
- CAAGCTAGAA ACCTCACAAA TACGATAATC AATGACAAAA ACTAAGTCAA TGAACCACTA ACAATGACTC   
  
  
- CCTACAACCT CAATTCATTG ACTATCACAA CAACTCCTTC GCTACAGAGT ATTTCAAATC TAAAAATAGA   
  
  
- CAACTAGAGA ACGATACGAA AACCTTAAAC TAATTAGTCT CCCAAGAGTC TCATACACCT CTGATCACCA   
  
  
- CTTCGCGAAA TAGTCTTCGA GACGTCGCTC TATTGACGAC TCAAGTTGCT CACAAGGTTT GTTCATTGAC   
  
  
- TCAAAAAAAA AGAAGAAAAC TAACTAATGG AAATACTACA ACAACTTCTA TGCCACAATT TCTAACGCAG   
  
  
- AAACAAACTA CCGTTACGTT CCATGCGCTT TACCTTTGAG ACAAAGACTT AGGACTGCAA CCTTCTAGCC   
  
  
- TAGAACGAGT TGACGAGTCA CTGTAAGTTT GAGTCCTCTT CGTCTTTGAC GTAGACCAGA CTAAGGATAT   
  
  
- ATGAGTTAAC AACTTGTATT GACAATTGAA CAACCTGTCT TGTTTTCAAA GAATGAAATC AATCAAAAAA   
  
  
- AAAAAAAAAC GTCTGCCAAT GTTATGTCCA TGACTTCTTC CGTCCCTCCG GCAGTCTTGC TTACGACTGT   
  
  
- GTGCTCTTGA CGTTCAAGTT CTTTGGCTAC GTCGTGCTCA CACACGTAGA AGTACTCTAA TGTCTTCGAC   
  
  
- TTCCTTGTCT CCTTCGTCTC CGCCTACGTC TCAAACTGTT GCGAAACTTC CTTCGTTAGT CTCCTCACGT   
  
  
- TCTGCGACAC TGAACGTAGT TACTCATAAA CCTTCTGCAA TCCATGCTCT AACGTCGAGA ACTTCGACTA   
  
  
- ATCAACAAGA GTTAGAGTAG TGAAGAAATG GGGAAAGCTG ACAATTGAAC GATACAACGA TTAATCGAAT   
  
  
- TCTATATTCT ATAAATCTTT GAGTTTTCAA TACTGTAAAG AACAACAGTT TCAATAGATA CAACTAATGA   
  
  
- CACCCATCAC CGATGTTTAT GTTATCGTTT AAGGTTTAAT AAATTTTGTG TTATAAACTA AACCTTTTGT   
  
  
- TAACTAAATT TCTCCTTGAA ACAATGTTTA TATTTTCAAT AATCGTAAAA GCCAAGTTTT ACGTATTCTA   
  
  
- CTCAACACAA AAAAAACAGC ACATGTGTAG TTTTTGACAG TATGCCTGTT ATTCGATCAG CGAAACTGGG   
  
  
- TAAGTGGCAT ACAGGGTTAC AGGAACTGGG ACGGTTTACT ATTCTTGCGG TCGTGTTTCC AAAATATTCA   
  
  
- AAGAGTTTCT CTTTGTTGTG TTACCCTTTT GTGTGGTAAC GAACTGAATG TTTACCTCTA GCCAACCTAG   
  
  
- TTAGAGACCT TTGTCCAACT TCATCTTAGA TTCGGGCAGA CCCGAGTACA GAGATGTGTC GTCTGGACTA   
  
  
- GTTTTGTTTA CCCTCTTACT CTTAGTGTAT TGAGACTTTC TCAGATAGAA AGATGCTCCA CCTAAGACAA   
  
  
- ATGGAAACCA GAGACGTCTA TCATCTACAA GGAACCGGTA CCGACCTCGC TAAAATTCGC AGTTTCGACG   
  
  
- TAGTGATCGT CACTGAGGTC AAGAATTTCA AAGCCAAAAT CAGTGTTCGC AAGA

+     CAT-box

| Site Name | Organism | Position | Strand | Matrix score. | sequence | function |
| --- | --- | --- | --- | --- | --- | --- |
| CAT-box | Arabidopsis thaliana | 1337 | - | 6 | GCCACT | cis-acting regulatory element related to meristem expression |

>PlantCARE\_8972   
+ GAACTGTTAA AGCCAATCAT TTAAAATAAA AAGTCAAACC GGTTTAAACC GGTTTTCAAT AAGTTAATAA   
  
  
+ CCGGACTCGG TGTTAAAGAG ATTAAATTTA ACGGTTTAAC TCAGTGAGAT CGGTTTCGCT TTCTCTCTGT   
  
  
+ CGTGTTTGAG AAGTTGAAAG AGAGGAGGAT GAGCGCTGAA GATTTCCAGA AGAAGGTTTC GATCAGAGAC   
  
  
+ TCCTCCGTCG CCGGAGAAAT GGAAATCGAA TGCGGCGGGT CTACTAGCTC CGCGGTTGGT TCGTCTCGAA   
  
  
+ CCTTGGTTTT GCTTCGGAGA CTGCTCGAGA TTCAGGAGCG TCGAGCACAG GCCTACGCCA AACTCAAAAG   
  
  
+ GTTCGATCTT TGGAGTGTTT ATGCTATTAG TTACTGTTTT TGATTCAGTT ACTTGGTGAT TGTTACTGAG   
  
  
+ GGATGTTGGA GTTAAGTAAC TGATAGTGTT GTTGAGGAAG CGATGTCTCA TAAAGTTTAG ATTTTTATCT   
  
  
+ GTTGATCTCT TGCTATGCTT TTGGAATTTG ATTAATCAGA GGGTTCTCAG AGTATGTGGA GACTAGTGGT   
  
  
+ GAAGCGCTTT ATCAGAAGCT CTGCAGCGAG ATAACTGCTG AGTTCAACGA GTGTTCCAAA CAAGTAACTG   
  
  
+ AGTTTTTTTT TCTTCTTTTG ATTGATTACC TTTATGATGT TGTTGAAGAT ACGGTGTTAA AGATTGCGTC   
  
  
+ TTTGTTTGAT GGCAATGCAA GGTACGCGAA ATGGAAACTC TGTTTCTGAA TCCTGACGTT GGAAGATCGG   
  
  
+ ATCTTGCTCA ACTGCTCAGT GACATTCAAA CTCAGGAGAA GCAGAAACTG CATCTGGTCT GATTCCTATA   
  
  
+ TACTCAATTG TTGAACATAA CTGTTAACTT GTTGGACAGA ACAAAAGTTT CTTACTTTAG TTAGTTTTTT   
  
  
+ TTTTTTTTTG CAGACGGTTA CAATACAGGT ACTGAAGAAG GCAGGGAGGC CGTCAGAACG AATGCTGACA   
  
  
+ CACGAGAACT GCAAGTTCAA GAAACCGATG CAGCACGAGT GTGTGCATCT TCATGAGATT ACAGAAGCTG   
  
  
+ AAGGAACAGA GGAAGCAGAG GCGGATGCAG AGTTTGACAA CGCTTTGAAG GAAGCAATCA GAGGAGTGCA   
  
  
+ AGACGCTGTG ACTTGCATCA ATGAGTATTT GGAAGACGTT AGGTACGAGA TTGCAGCTCT TGAAGCTGAT   
  
  
+ TAGTTGTTCT CAATCTCATC ACTTCTTTAC CCCTTTCGAC TGTTAACTTG CTATGTTGCT AATTAGCTTA   
  
  
+ AGATATAAGA TATTTAGAAA CTCAAAAGTT ATGACATTTC TTGTTGTCAA AGTTATCTAT GTTGATTACT   
  
  
+ GTGGGTAGTG GCTACAAATA CAATAGCAAA TTCCAAATTA TTTAAAACAC AATATTTGAT TTGGAAAACA   
  
  
+ ATTGATTTAA AGAGGAACTT TGTTACAAAT ATAAAAGTTA TTAGCATTTT CGGTTCAAAA TGCATAAGAT   
  
  
+ GAGTTGTGTT TTTTTTGTCG TGTACACATC AAAAACTGTC ATACGGACAA TAAGCTAGTC GCTTTGACCC   
  
  
+ ATTCACCGTA TGTCCCAATG TCCTTGACCC TGCCAAATGA TAAGAACGCC AGCACAAAGG TTTTATAAGT   
  
  
+ TTCTCAAAGA GAAACAACAC AATGGGAAAA CACACCATTG CTTGACTTAC AAATGGAGAT CGGTTGGATC   
  
  
+ AATCTCTGGA AACAGGTTGA AGTAGAATCT AAGCCCGTCT GGGCTCATGT CTCTACACAG CAGACCTGAT   
  
  
+ CAAAACAAAT GGGAGAATGA GAATCACATA ACTCTGAAAG AGTCTATCTT TCTACGAGGT GGATTCTGTT   
  
  
+ TACCTTTGGT CTCTGCAGAT AGTAGATGTT CCTTGGCCAT GGCTGGAGCG ATTTTAAGCG TCAAAGCTGC   
  
  
+ ATCACTAGCA GTGACTCCAG TTCTTAAAGT TTCGGTTTTA GTCACAAGCG TTCT  

- CTTGACAATT TCGGTTAGTA AATTTTATTT TTCAGTTTGG CCAAATTTGG CCAAAAGTTA TTCAATTATT   
  
  
- GGCCTGAGCC ACAATTTCTC TAATTTAAAT TGCCAAATTG AGTCACTCTA GCCAAAGCGA AAGAGAGACA   
  
  
- GCACAAACTC TTCAACTTTC TCTCCTCCTA CTCGCGACTT CTAAAGGTCT TCTTCCAAAG CTAGTCTCTG   
  
  
- AGGAGGCAGC GGCCTCTTTA CCTTTAGCTT ACGCCGCCCA GATGATCGAG GCGCCAACCA AGCAGAGCTT   
  
  
- GGAACCAAAA CGAAGCCTCT GACGAGCTCT AAGTCCTCGC AGCTCGTGTC CGGATGCGGT TTGAGTTTTC   
  
  
- CAAGCTAGAA ACCTCACAAA TACGATAATC AATGACAAAA ACTAAGTCAA TGAACCACTA ACAATGACTC   
  
  
- CCTACAACCT CAATTCATTG ACTATCACAA CAACTCCTTC GCTACAGAGT ATTTCAAATC TAAAAATAGA   
  
  
- CAACTAGAGA ACGATACGAA AACCTTAAAC TAATTAGTCT CCCAAGAGTC TCATACACCT CTGATCACCA   
  
  
- CTTCGCGAAA TAGTCTTCGA GACGTCGCTC TATTGACGAC TCAAGTTGCT CACAAGGTTT GTTCATTGAC   
  
  
- TCAAAAAAAA AGAAGAAAAC TAACTAATGG AAATACTACA ACAACTTCTA TGCCACAATT TCTAACGCAG   
  
  
- AAACAAACTA CCGTTACGTT CCATGCGCTT TACCTTTGAG ACAAAGACTT AGGACTGCAA CCTTCTAGCC   
  
  
- TAGAACGAGT TGACGAGTCA CTGTAAGTTT GAGTCCTCTT CGTCTTTGAC GTAGACCAGA CTAAGGATAT   
  
  
- ATGAGTTAAC AACTTGTATT GACAATTGAA CAACCTGTCT TGTTTTCAAA GAATGAAATC AATCAAAAAA   
  
  
- AAAAAAAAAC GTCTGCCAAT GTTATGTCCA TGACTTCTTC CGTCCCTCCG GCAGTCTTGC TTACGACTGT   
  
  
- GTGCTCTTGA CGTTCAAGTT CTTTGGCTAC GTCGTGCTCA CACACGTAGA AGTACTCTAA TGTCTTCGAC   
  
  
- TTCCTTGTCT CCTTCGTCTC CGCCTACGTC TCAAACTGTT GCGAAACTTC CTTCGTTAGT CTCCTCACGT   
  
  
- TCTGCGACAC TGAACGTAGT TACTCATAAA CCTTCTGCAA TCCATGCTCT AACGTCGAGA ACTTCGACTA   
  
  
- ATCAACAAGA GTTAGAGTAG TGAAGAAATG GGGAAAGCTG ACAATTGAAC GATACAACGA TTAATCGAAT   
  
  
- TCTATATTCT ATAAATCTTT GAGTTTTCAA TACTGTAAAG AACAACAGTT TCAATAGATA CAACTAATGA   
  
  
- CACCCATCAC CGATGTTTAT GTTATCGTTT AAGGTTTAAT AAATTTTGTG TTATAAACTA AACCTTTTGT   
  
  
- TAACTAAATT TCTCCTTGAA ACAATGTTTA TATTTTCAAT AATCGTAAAA GCCAAGTTTT ACGTATTCTA   
  
  
- CTCAACACAA AAAAAACAGC ACATGTGTAG TTTTTGACAG TATGCCTGTT ATTCGATCAG CGAAACTGGG   
  
  
- TAAGTGGCAT ACAGGGTTAC AGGAACTGGG ACGGTTTACT ATTCTTGCGG TCGTGTTTCC AAAATATTCA   
  
  
- AAGAGTTTCT CTTTGTTGTG TTACCCTTTT GTGTGGTAAC GAACTGAATG TTTACCTCTA GCCAACCTAG   
  
  
- TTAGAGACCT TTGTCCAACT TCATCTTAGA TTCGGGCAGA CCCGAGTACA GAGATGTGTC GTCTGGACTA   
  
  
- GTTTTGTTTA CCCTCTTACT CTTAGTGTAT TGAGACTTTC TCAGATAGAA AGATGCTCCA CCTAAGACAA   
  
  
- ATGGAAACCA GAGACGTCTA TCATCTACAA GGAACCGGTA CCGACCTCGC TAAAATTCGC AGTTTCGACG   
  
  
- TAGTGATCGT CACTGAGGTC AAGAATTTCA AAGCCAAAAT CAGTGTTCGC AAGA

+     CGTCA-motif

| Site Name | Organism | Position | Strand | Matrix score. | sequence | function |
| --- | --- | --- | --- | --- | --- | --- |
| CGTCA-motif | Hordeum vulgare | 754 | - | 5 | CGTCA | cis-acting regulatory element involved in the MeJA-responsiveness |
| CGTCA-motif | Hordeum vulgare | 1879 | + | 5 | CGTCA | cis-acting regulatory element involved in the MeJA-responsiveness |
| CGTCA-motif | Hordeum vulgare | 961 | + | 5 | CGTCA | cis-acting regulatory element involved in the MeJA-responsiveness |

>PlantCARE\_8972   
+ GAACTGTTAA AGCCAATCAT TTAAAATAAA AAGTCAAACC GGTTTAAACC GGTTTTCAAT AAGTTAATAA   
  
  
+ CCGGACTCGG TGTTAAAGAG ATTAAATTTA ACGGTTTAAC TCAGTGAGAT CGGTTTCGCT TTCTCTCTGT   
  
  
+ CGTGTTTGAG AAGTTGAAAG AGAGGAGGAT GAGCGCTGAA GATTTCCAGA AGAAGGTTTC GATCAGAGAC   
  
  
+ TCCTCCGTCG CCGGAGAAAT GGAAATCGAA TGCGGCGGGT CTACTAGCTC CGCGGTTGGT TCGTCTCGAA   
  
  
+ CCTTGGTTTT GCTTCGGAGA CTGCTCGAGA TTCAGGAGCG TCGAGCACAG GCCTACGCCA AACTCAAAAG   
  
  
+ GTTCGATCTT TGGAGTGTTT ATGCTATTAG TTACTGTTTT TGATTCAGTT ACTTGGTGAT TGTTACTGAG   
  
  
+ GGATGTTGGA GTTAAGTAAC TGATAGTGTT GTTGAGGAAG CGATGTCTCA TAAAGTTTAG ATTTTTATCT   
  
  
+ GTTGATCTCT TGCTATGCTT TTGGAATTTG ATTAATCAGA GGGTTCTCAG AGTATGTGGA GACTAGTGGT   
  
  
+ GAAGCGCTTT ATCAGAAGCT CTGCAGCGAG ATAACTGCTG AGTTCAACGA GTGTTCCAAA CAAGTAACTG   
  
  
+ AGTTTTTTTT TCTTCTTTTG ATTGATTACC TTTATGATGT TGTTGAAGAT ACGGTGTTAA AGATTGCGTC   
  
  
+ TTTGTTTGAT GGCAATGCAA GGTACGCGAA ATGGAAACTC TGTTTCTGAA TCCTGACGTT GGAAGATCGG   
  
  
+ ATCTTGCTCA ACTGCTCAGT GACATTCAAA CTCAGGAGAA GCAGAAACTG CATCTGGTCT GATTCCTATA   
  
  
+ TACTCAATTG TTGAACATAA CTGTTAACTT GTTGGACAGA ACAAAAGTTT CTTACTTTAG TTAGTTTTTT   
  
  
+ TTTTTTTTTG CAGACGGTTA CAATACAGGT ACTGAAGAAG GCAGGGAGGC CGTCAGAACG AATGCTGACA   
  
  
+ CACGAGAACT GCAAGTTCAA GAAACCGATG CAGCACGAGT GTGTGCATCT TCATGAGATT ACAGAAGCTG   
  
  
+ AAGGAACAGA GGAAGCAGAG GCGGATGCAG AGTTTGACAA CGCTTTGAAG GAAGCAATCA GAGGAGTGCA   
  
  
+ AGACGCTGTG ACTTGCATCA ATGAGTATTT GGAAGACGTT AGGTACGAGA TTGCAGCTCT TGAAGCTGAT   
  
  
+ TAGTTGTTCT CAATCTCATC ACTTCTTTAC CCCTTTCGAC TGTTAACTTG CTATGTTGCT AATTAGCTTA   
  
  
+ AGATATAAGA TATTTAGAAA CTCAAAAGTT ATGACATTTC TTGTTGTCAA AGTTATCTAT GTTGATTACT   
  
  
+ GTGGGTAGTG GCTACAAATA CAATAGCAAA TTCCAAATTA TTTAAAACAC AATATTTGAT TTGGAAAACA   
  
  
+ ATTGATTTAA AGAGGAACTT TGTTACAAAT ATAAAAGTTA TTAGCATTTT CGGTTCAAAA TGCATAAGAT   
  
  
+ GAGTTGTGTT TTTTTTGTCG TGTACACATC AAAAACTGTC ATACGGACAA TAAGCTAGTC GCTTTGACCC   
  
  
+ ATTCACCGTA TGTCCCAATG TCCTTGACCC TGCCAAATGA TAAGAACGCC AGCACAAAGG TTTTATAAGT   
  
  
+ TTCTCAAAGA GAAACAACAC AATGGGAAAA CACACCATTG CTTGACTTAC AAATGGAGAT CGGTTGGATC   
  
  
+ AATCTCTGGA AACAGGTTGA AGTAGAATCT AAGCCCGTCT GGGCTCATGT CTCTACACAG CAGACCTGAT   
  
  
+ CAAAACAAAT GGGAGAATGA GAATCACATA ACTCTGAAAG AGTCTATCTT TCTACGAGGT GGATTCTGTT   
  
  
+ TACCTTTGGT CTCTGCAGAT AGTAGATGTT CCTTGGCCAT GGCTGGAGCG ATTTTAAGCG TCAAAGCTGC   
  
  
+ ATCACTAGCA GTGACTCCAG TTCTTAAAGT TTCGGTTTTA GTCACAAGCG TTCT  

- CTTGACAATT TCGGTTAGTA AATTTTATTT TTCAGTTTGG CCAAATTTGG CCAAAAGTTA TTCAATTATT   
  
  
- GGCCTGAGCC ACAATTTCTC TAATTTAAAT TGCCAAATTG AGTCACTCTA GCCAAAGCGA AAGAGAGACA   
  
  
- GCACAAACTC TTCAACTTTC TCTCCTCCTA CTCGCGACTT CTAAAGGTCT TCTTCCAAAG CTAGTCTCTG   
  
  
- AGGAGGCAGC GGCCTCTTTA CCTTTAGCTT ACGCCGCCCA GATGATCGAG GCGCCAACCA AGCAGAGCTT   
  
  
- GGAACCAAAA CGAAGCCTCT GACGAGCTCT AAGTCCTCGC AGCTCGTGTC CGGATGCGGT TTGAGTTTTC   
  
  
- CAAGCTAGAA ACCTCACAAA TACGATAATC AATGACAAAA ACTAAGTCAA TGAACCACTA ACAATGACTC   
  
  
- CCTACAACCT CAATTCATTG ACTATCACAA CAACTCCTTC GCTACAGAGT ATTTCAAATC TAAAAATAGA   
  
  
- CAACTAGAGA ACGATACGAA AACCTTAAAC TAATTAGTCT CCCAAGAGTC TCATACACCT CTGATCACCA   
  
  
- CTTCGCGAAA TAGTCTTCGA GACGTCGCTC TATTGACGAC TCAAGTTGCT CACAAGGTTT GTTCATTGAC   
  
  
- TCAAAAAAAA AGAAGAAAAC TAACTAATGG AAATACTACA ACAACTTCTA TGCCACAATT TCTAACGCAG   
  
  
- AAACAAACTA CCGTTACGTT CCATGCGCTT TACCTTTGAG ACAAAGACTT AGGACTGCAA CCTTCTAGCC   
  
  
- TAGAACGAGT TGACGAGTCA CTGTAAGTTT GAGTCCTCTT CGTCTTTGAC GTAGACCAGA CTAAGGATAT   
  
  
- ATGAGTTAAC AACTTGTATT GACAATTGAA CAACCTGTCT TGTTTTCAAA GAATGAAATC AATCAAAAAA   
  
  
- AAAAAAAAAC GTCTGCCAAT GTTATGTCCA TGACTTCTTC CGTCCCTCCG GCAGTCTTGC TTACGACTGT   
  
  
- GTGCTCTTGA CGTTCAAGTT CTTTGGCTAC GTCGTGCTCA CACACGTAGA AGTACTCTAA TGTCTTCGAC   
  
  
- TTCCTTGTCT CCTTCGTCTC CGCCTACGTC TCAAACTGTT GCGAAACTTC CTTCGTTAGT CTCCTCACGT   
  
  
- TCTGCGACAC TGAACGTAGT TACTCATAAA CCTTCTGCAA TCCATGCTCT AACGTCGAGA ACTTCGACTA   
  
  
- ATCAACAAGA GTTAGAGTAG TGAAGAAATG GGGAAAGCTG ACAATTGAAC GATACAACGA TTAATCGAAT   
  
  
- TCTATATTCT ATAAATCTTT GAGTTTTCAA TACTGTAAAG AACAACAGTT TCAATAGATA CAACTAATGA   
  
  
- CACCCATCAC CGATGTTTAT GTTATCGTTT AAGGTTTAAT AAATTTTGTG TTATAAACTA AACCTTTTGT   
  
  
- TAACTAAATT TCTCCTTGAA ACAATGTTTA TATTTTCAAT AATCGTAAAA GCCAAGTTTT ACGTATTCTA   
  
  
- CTCAACACAA AAAAAACAGC ACATGTGTAG TTTTTGACAG TATGCCTGTT ATTCGATCAG CGAAACTGGG   
  
  
- TAAGTGGCAT ACAGGGTTAC AGGAACTGGG ACGGTTTACT ATTCTTGCGG TCGTGTTTCC AAAATATTCA   
  
  
- AAGAGTTTCT CTTTGTTGTG TTACCCTTTT GTGTGGTAAC GAACTGAATG TTTACCTCTA GCCAACCTAG   
  
  
- TTAGAGACCT TTGTCCAACT TCATCTTAGA TTCGGGCAGA CCCGAGTACA GAGATGTGTC GTCTGGACTA   
  
  
- GTTTTGTTTA CCCTCTTACT CTTAGTGTAT TGAGACTTTC TCAGATAGAA AGATGCTCCA CCTAAGACAA   
  
  
- ATGGAAACCA GAGACGTCTA TCATCTACAA GGAACCGGTA CCGACCTCGC TAAAATTCGC AGTTTCGACG   
  
  
- TAGTGATCGT CACTGAGGTC AAGAATTTCA AAGCCAAAAT CAGTGTTCGC AAGA

+     ERE

| Site Name | Organism | Position | Strand | Matrix score. | sequence | function |
| --- | --- | --- | --- | --- | --- | --- |
| ERE | Nicotiana glutinos | 20 | - | 8 | ATTTTAAA |  |

>PlantCARE\_8972   
+ GAACTGTTAA AGCCAATCAT TTAAAATAAA AAGTCAAACC GGTTTAAACC GGTTTTCAAT AAGTTAATAA   
  
  
+ CCGGACTCGG TGTTAAAGAG ATTAAATTTA ACGGTTTAAC TCAGTGAGAT CGGTTTCGCT TTCTCTCTGT   
  
  
+ CGTGTTTGAG AAGTTGAAAG AGAGGAGGAT GAGCGCTGAA GATTTCCAGA AGAAGGTTTC GATCAGAGAC   
  
  
+ TCCTCCGTCG CCGGAGAAAT GGAAATCGAA TGCGGCGGGT CTACTAGCTC CGCGGTTGGT TCGTCTCGAA   
  
  
+ CCTTGGTTTT GCTTCGGAGA CTGCTCGAGA TTCAGGAGCG TCGAGCACAG GCCTACGCCA AACTCAAAAG   
  
  
+ GTTCGATCTT TGGAGTGTTT ATGCTATTAG TTACTGTTTT TGATTCAGTT ACTTGGTGAT TGTTACTGAG   
  
  
+ GGATGTTGGA GTTAAGTAAC TGATAGTGTT GTTGAGGAAG CGATGTCTCA TAAAGTTTAG ATTTTTATCT   
  
  
+ GTTGATCTCT TGCTATGCTT TTGGAATTTG ATTAATCAGA GGGTTCTCAG AGTATGTGGA GACTAGTGGT   
  
  
+ GAAGCGCTTT ATCAGAAGCT CTGCAGCGAG ATAACTGCTG AGTTCAACGA GTGTTCCAAA CAAGTAACTG   
  
  
+ AGTTTTTTTT TCTTCTTTTG ATTGATTACC TTTATGATGT TGTTGAAGAT ACGGTGTTAA AGATTGCGTC   
  
  
+ TTTGTTTGAT GGCAATGCAA GGTACGCGAA ATGGAAACTC TGTTTCTGAA TCCTGACGTT GGAAGATCGG   
  
  
+ ATCTTGCTCA ACTGCTCAGT GACATTCAAA CTCAGGAGAA GCAGAAACTG CATCTGGTCT GATTCCTATA   
  
  
+ TACTCAATTG TTGAACATAA CTGTTAACTT GTTGGACAGA ACAAAAGTTT CTTACTTTAG TTAGTTTTTT   
  
  
+ TTTTTTTTTG CAGACGGTTA CAATACAGGT ACTGAAGAAG GCAGGGAGGC CGTCAGAACG AATGCTGACA   
  
  
+ CACGAGAACT GCAAGTTCAA GAAACCGATG CAGCACGAGT GTGTGCATCT TCATGAGATT ACAGAAGCTG   
  
  
+ AAGGAACAGA GGAAGCAGAG GCGGATGCAG AGTTTGACAA CGCTTTGAAG GAAGCAATCA GAGGAGTGCA   
  
  
+ AGACGCTGTG ACTTGCATCA ATGAGTATTT GGAAGACGTT AGGTACGAGA TTGCAGCTCT TGAAGCTGAT   
  
  
+ TAGTTGTTCT CAATCTCATC ACTTCTTTAC CCCTTTCGAC TGTTAACTTG CTATGTTGCT AATTAGCTTA   
  
  
+ AGATATAAGA TATTTAGAAA CTCAAAAGTT ATGACATTTC TTGTTGTCAA AGTTATCTAT GTTGATTACT   
  
  
+ GTGGGTAGTG GCTACAAATA CAATAGCAAA TTCCAAATTA TTTAAAACAC AATATTTGAT TTGGAAAACA   
  
  
+ ATTGATTTAA AGAGGAACTT TGTTACAAAT ATAAAAGTTA TTAGCATTTT CGGTTCAAAA TGCATAAGAT   
  
  
+ GAGTTGTGTT TTTTTTGTCG TGTACACATC AAAAACTGTC ATACGGACAA TAAGCTAGTC GCTTTGACCC   
  
  
+ ATTCACCGTA TGTCCCAATG TCCTTGACCC TGCCAAATGA TAAGAACGCC AGCACAAAGG TTTTATAAGT   
  
  
+ TTCTCAAAGA GAAACAACAC AATGGGAAAA CACACCATTG CTTGACTTAC AAATGGAGAT CGGTTGGATC   
  
  
+ AATCTCTGGA AACAGGTTGA AGTAGAATCT AAGCCCGTCT GGGCTCATGT CTCTACACAG CAGACCTGAT   
  
  
+ CAAAACAAAT GGGAGAATGA GAATCACATA ACTCTGAAAG AGTCTATCTT TCTACGAGGT GGATTCTGTT   
  
  
+ TACCTTTGGT CTCTGCAGAT AGTAGATGTT CCTTGGCCAT GGCTGGAGCG ATTTTAAGCG TCAAAGCTGC   
  
  
+ ATCACTAGCA GTGACTCCAG TTCTTAAAGT TTCGGTTTTA GTCACAAGCG TTCT  

- CTTGACAATT TCGGTTAGTA AATTTTATTT TTCAGTTTGG CCAAATTTGG CCAAAAGTTA TTCAATTATT   
  
  
- GGCCTGAGCC ACAATTTCTC TAATTTAAAT TGCCAAATTG AGTCACTCTA GCCAAAGCGA AAGAGAGACA   
  
  
- GCACAAACTC TTCAACTTTC TCTCCTCCTA CTCGCGACTT CTAAAGGTCT TCTTCCAAAG CTAGTCTCTG   
  
  
- AGGAGGCAGC GGCCTCTTTA CCTTTAGCTT ACGCCGCCCA GATGATCGAG GCGCCAACCA AGCAGAGCTT   
  
  
- GGAACCAAAA CGAAGCCTCT GACGAGCTCT AAGTCCTCGC AGCTCGTGTC CGGATGCGGT TTGAGTTTTC   
  
  
- CAAGCTAGAA ACCTCACAAA TACGATAATC AATGACAAAA ACTAAGTCAA TGAACCACTA ACAATGACTC   
  
  
- CCTACAACCT CAATTCATTG ACTATCACAA CAACTCCTTC GCTACAGAGT ATTTCAAATC TAAAAATAGA   
  
  
- CAACTAGAGA ACGATACGAA AACCTTAAAC TAATTAGTCT CCCAAGAGTC TCATACACCT CTGATCACCA   
  
  
- CTTCGCGAAA TAGTCTTCGA GACGTCGCTC TATTGACGAC TCAAGTTGCT CACAAGGTTT GTTCATTGAC   
  
  
- TCAAAAAAAA AGAAGAAAAC TAACTAATGG AAATACTACA ACAACTTCTA TGCCACAATT TCTAACGCAG   
  
  
- AAACAAACTA CCGTTACGTT CCATGCGCTT TACCTTTGAG ACAAAGACTT AGGACTGCAA CCTTCTAGCC   
  
  
- TAGAACGAGT TGACGAGTCA CTGTAAGTTT GAGTCCTCTT CGTCTTTGAC GTAGACCAGA CTAAGGATAT   
  
  
- ATGAGTTAAC AACTTGTATT GACAATTGAA CAACCTGTCT TGTTTTCAAA GAATGAAATC AATCAAAAAA   
  
  
- AAAAAAAAAC GTCTGCCAAT GTTATGTCCA TGACTTCTTC CGTCCCTCCG GCAGTCTTGC TTACGACTGT   
  
  
- GTGCTCTTGA CGTTCAAGTT CTTTGGCTAC GTCGTGCTCA CACACGTAGA AGTACTCTAA TGTCTTCGAC   
  
  
- TTCCTTGTCT CCTTCGTCTC CGCCTACGTC TCAAACTGTT GCGAAACTTC CTTCGTTAGT CTCCTCACGT   
  
  
- TCTGCGACAC TGAACGTAGT TACTCATAAA CCTTCTGCAA TCCATGCTCT AACGTCGAGA ACTTCGACTA   
  
  
- ATCAACAAGA GTTAGAGTAG TGAAGAAATG GGGAAAGCTG ACAATTGAAC GATACAACGA TTAATCGAAT   
  
  
- TCTATATTCT ATAAATCTTT GAGTTTTCAA TACTGTAAAG AACAACAGTT TCAATAGATA CAACTAATGA   
  
  
- CACCCATCAC CGATGTTTAT GTTATCGTTT AAGGTTTAAT AAATTTTGTG TTATAAACTA AACCTTTTGT   
  
  
- TAACTAAATT TCTCCTTGAA ACAATGTTTA TATTTTCAAT AATCGTAAAA GCCAAGTTTT ACGTATTCTA   
  
  
- CTCAACACAA AAAAAACAGC ACATGTGTAG TTTTTGACAG TATGCCTGTT ATTCGATCAG CGAAACTGGG   
  
  
- TAAGTGGCAT ACAGGGTTAC AGGAACTGGG ACGGTTTACT ATTCTTGCGG TCGTGTTTCC AAAATATTCA   
  
  
- AAGAGTTTCT CTTTGTTGTG TTACCCTTTT GTGTGGTAAC GAACTGAATG TTTACCTCTA GCCAACCTAG   
  
  
- TTAGAGACCT TTGTCCAACT TCATCTTAGA TTCGGGCAGA CCCGAGTACA GAGATGTGTC GTCTGGACTA   
  
  
- GTTTTGTTTA CCCTCTTACT CTTAGTGTAT TGAGACTTTC TCAGATAGAA AGATGCTCCA CCTAAGACAA   
  
  
- ATGGAAACCA GAGACGTCTA TCATCTACAA GGAACCGGTA CCGACCTCGC TAAAATTCGC AGTTTCGACG   
  
  
- TAGTGATCGT CACTGAGGTC AAGAATTTCA AAGCCAAAAT CAGTGTTCGC AAGA

+     G-box

| Site Name | Organism | Position | Strand | Matrix score. | sequence | function |
| --- | --- | --- | --- | --- | --- | --- |
| G-box | Zea mays | 139 | - | 6 | CACGAC | cis-acting regulatory element involved in light responsiveness |
| G-box | Zea mays | 1487 | - | 6 | CACGAC | cis-acting regulatory element involved in light responsiveness |

>PlantCARE\_8972   
+ GAACTGTTAA AGCCAATCAT TTAAAATAAA AAGTCAAACC GGTTTAAACC GGTTTTCAAT AAGTTAATAA   
  
  
+ CCGGACTCGG TGTTAAAGAG ATTAAATTTA ACGGTTTAAC TCAGTGAGAT CGGTTTCGCT TTCTCTCTGT   
  
  
+ CGTGTTTGAG AAGTTGAAAG AGAGGAGGAT GAGCGCTGAA GATTTCCAGA AGAAGGTTTC GATCAGAGAC   
  
  
+ TCCTCCGTCG CCGGAGAAAT GGAAATCGAA TGCGGCGGGT CTACTAGCTC CGCGGTTGGT TCGTCTCGAA   
  
  
+ CCTTGGTTTT GCTTCGGAGA CTGCTCGAGA TTCAGGAGCG TCGAGCACAG GCCTACGCCA AACTCAAAAG   
  
  
+ GTTCGATCTT TGGAGTGTTT ATGCTATTAG TTACTGTTTT TGATTCAGTT ACTTGGTGAT TGTTACTGAG   
  
  
+ GGATGTTGGA GTTAAGTAAC TGATAGTGTT GTTGAGGAAG CGATGTCTCA TAAAGTTTAG ATTTTTATCT   
  
  
+ GTTGATCTCT TGCTATGCTT TTGGAATTTG ATTAATCAGA GGGTTCTCAG AGTATGTGGA GACTAGTGGT   
  
  
+ GAAGCGCTTT ATCAGAAGCT CTGCAGCGAG ATAACTGCTG AGTTCAACGA GTGTTCCAAA CAAGTAACTG   
  
  
+ AGTTTTTTTT TCTTCTTTTG ATTGATTACC TTTATGATGT TGTTGAAGAT ACGGTGTTAA AGATTGCGTC   
  
  
+ TTTGTTTGAT GGCAATGCAA GGTACGCGAA ATGGAAACTC TGTTTCTGAA TCCTGACGTT GGAAGATCGG   
  
  
+ ATCTTGCTCA ACTGCTCAGT GACATTCAAA CTCAGGAGAA GCAGAAACTG CATCTGGTCT GATTCCTATA   
  
  
+ TACTCAATTG TTGAACATAA CTGTTAACTT GTTGGACAGA ACAAAAGTTT CTTACTTTAG TTAGTTTTTT   
  
  
+ TTTTTTTTTG CAGACGGTTA CAATACAGGT ACTGAAGAAG GCAGGGAGGC CGTCAGAACG AATGCTGACA   
  
  
+ CACGAGAACT GCAAGTTCAA GAAACCGATG CAGCACGAGT GTGTGCATCT TCATGAGATT ACAGAAGCTG   
  
  
+ AAGGAACAGA GGAAGCAGAG GCGGATGCAG AGTTTGACAA CGCTTTGAAG GAAGCAATCA GAGGAGTGCA   
  
  
+ AGACGCTGTG ACTTGCATCA ATGAGTATTT GGAAGACGTT AGGTACGAGA TTGCAGCTCT TGAAGCTGAT   
  
  
+ TAGTTGTTCT CAATCTCATC ACTTCTTTAC CCCTTTCGAC TGTTAACTTG CTATGTTGCT AATTAGCTTA   
  
  
+ AGATATAAGA TATTTAGAAA CTCAAAAGTT ATGACATTTC TTGTTGTCAA AGTTATCTAT GTTGATTACT   
  
  
+ GTGGGTAGTG GCTACAAATA CAATAGCAAA TTCCAAATTA TTTAAAACAC AATATTTGAT TTGGAAAACA   
  
  
+ ATTGATTTAA AGAGGAACTT TGTTACAAAT ATAAAAGTTA TTAGCATTTT CGGTTCAAAA TGCATAAGAT   
  
  
+ GAGTTGTGTT TTTTTTGTCG TGTACACATC AAAAACTGTC ATACGGACAA TAAGCTAGTC GCTTTGACCC   
  
  
+ ATTCACCGTA TGTCCCAATG TCCTTGACCC TGCCAAATGA TAAGAACGCC AGCACAAAGG TTTTATAAGT   
  
  
+ TTCTCAAAGA GAAACAACAC AATGGGAAAA CACACCATTG CTTGACTTAC AAATGGAGAT CGGTTGGATC   
  
  
+ AATCTCTGGA AACAGGTTGA AGTAGAATCT AAGCCCGTCT GGGCTCATGT CTCTACACAG CAGACCTGAT   
  
  
+ CAAAACAAAT GGGAGAATGA GAATCACATA ACTCTGAAAG AGTCTATCTT TCTACGAGGT GGATTCTGTT   
  
  
+ TACCTTTGGT CTCTGCAGAT AGTAGATGTT CCTTGGCCAT GGCTGGAGCG ATTTTAAGCG TCAAAGCTGC   
  
  
+ ATCACTAGCA GTGACTCCAG TTCTTAAAGT TTCGGTTTTA GTCACAAGCG TTCT  

- CTTGACAATT TCGGTTAGTA AATTTTATTT TTCAGTTTGG CCAAATTTGG CCAAAAGTTA TTCAATTATT   
  
  
- GGCCTGAGCC ACAATTTCTC TAATTTAAAT TGCCAAATTG AGTCACTCTA GCCAAAGCGA AAGAGAGACA   
  
  
- GCACAAACTC TTCAACTTTC TCTCCTCCTA CTCGCGACTT CTAAAGGTCT TCTTCCAAAG CTAGTCTCTG   
  
  
- AGGAGGCAGC GGCCTCTTTA CCTTTAGCTT ACGCCGCCCA GATGATCGAG GCGCCAACCA AGCAGAGCTT   
  
  
- GGAACCAAAA CGAAGCCTCT GACGAGCTCT AAGTCCTCGC AGCTCGTGTC CGGATGCGGT TTGAGTTTTC   
  
  
- CAAGCTAGAA ACCTCACAAA TACGATAATC AATGACAAAA ACTAAGTCAA TGAACCACTA ACAATGACTC   
  
  
- CCTACAACCT CAATTCATTG ACTATCACAA CAACTCCTTC GCTACAGAGT ATTTCAAATC TAAAAATAGA   
  
  
- CAACTAGAGA ACGATACGAA AACCTTAAAC TAATTAGTCT CCCAAGAGTC TCATACACCT CTGATCACCA   
  
  
- CTTCGCGAAA TAGTCTTCGA GACGTCGCTC TATTGACGAC TCAAGTTGCT CACAAGGTTT GTTCATTGAC   
  
  
- TCAAAAAAAA AGAAGAAAAC TAACTAATGG AAATACTACA ACAACTTCTA TGCCACAATT TCTAACGCAG   
  
  
- AAACAAACTA CCGTTACGTT CCATGCGCTT TACCTTTGAG ACAAAGACTT AGGACTGCAA CCTTCTAGCC   
  
  
- TAGAACGAGT TGACGAGTCA CTGTAAGTTT GAGTCCTCTT CGTCTTTGAC GTAGACCAGA CTAAGGATAT   
  
  
- ATGAGTTAAC AACTTGTATT GACAATTGAA CAACCTGTCT TGTTTTCAAA GAATGAAATC AATCAAAAAA   
  
  
- AAAAAAAAAC GTCTGCCAAT GTTATGTCCA TGACTTCTTC CGTCCCTCCG GCAGTCTTGC TTACGACTGT   
  
  
- GTGCTCTTGA CGTTCAAGTT CTTTGGCTAC GTCGTGCTCA CACACGTAGA AGTACTCTAA TGTCTTCGAC   
  
  
- TTCCTTGTCT CCTTCGTCTC CGCCTACGTC TCAAACTGTT GCGAAACTTC CTTCGTTAGT CTCCTCACGT   
  
  
- TCTGCGACAC TGAACGTAGT TACTCATAAA CCTTCTGCAA TCCATGCTCT AACGTCGAGA ACTTCGACTA   
  
  
- ATCAACAAGA GTTAGAGTAG TGAAGAAATG GGGAAAGCTG ACAATTGAAC GATACAACGA TTAATCGAAT   
  
  
- TCTATATTCT ATAAATCTTT GAGTTTTCAA TACTGTAAAG AACAACAGTT TCAATAGATA CAACTAATGA   
  
  
- CACCCATCAC CGATGTTTAT GTTATCGTTT AAGGTTTAAT AAATTTTGTG TTATAAACTA AACCTTTTGT   
  
  
- TAACTAAATT TCTCCTTGAA ACAATGTTTA TATTTTCAAT AATCGTAAAA GCCAAGTTTT ACGTATTCTA   
  
  
- CTCAACACAA AAAAAACAGC ACATGTGTAG TTTTTGACAG TATGCCTGTT ATTCGATCAG CGAAACTGGG   
  
  
- TAAGTGGCAT ACAGGGTTAC AGGAACTGGG ACGGTTTACT ATTCTTGCGG TCGTGTTTCC AAAATATTCA   
  
  
- AAGAGTTTCT CTTTGTTGTG TTACCCTTTT GTGTGGTAAC GAACTGAATG TTTACCTCTA GCCAACCTAG   
  
  
- TTAGAGACCT TTGTCCAACT TCATCTTAGA TTCGGGCAGA CCCGAGTACA GAGATGTGTC GTCTGGACTA   
  
  
- GTTTTGTTTA CCCTCTTACT CTTAGTGTAT TGAGACTTTC TCAGATAGAA AGATGCTCCA CCTAAGACAA   
  
  
- ATGGAAACCA GAGACGTCTA TCATCTACAA GGAACCGGTA CCGACCTCGC TAAAATTCGC AGTTTCGACG   
  
  
- TAGTGATCGT CACTGAGGTC AAGAATTTCA AAGCCAAAAT CAGTGTTCGC AAGA

+     GA-motif

| Site Name | Organism | Position | Strand | Matrix score. | sequence | function |
| --- | --- | --- | --- | --- | --- | --- |
| GA-motif | Arabidopsis thaliana | 1313 | - | 8 | ATAGATAA | part of a light responsive element |

>PlantCARE\_8972   
+ GAACTGTTAA AGCCAATCAT TTAAAATAAA AAGTCAAACC GGTTTAAACC GGTTTTCAAT AAGTTAATAA   
  
  
+ CCGGACTCGG TGTTAAAGAG ATTAAATTTA ACGGTTTAAC TCAGTGAGAT CGGTTTCGCT TTCTCTCTGT   
  
  
+ CGTGTTTGAG AAGTTGAAAG AGAGGAGGAT GAGCGCTGAA GATTTCCAGA AGAAGGTTTC GATCAGAGAC   
  
  
+ TCCTCCGTCG CCGGAGAAAT GGAAATCGAA TGCGGCGGGT CTACTAGCTC CGCGGTTGGT TCGTCTCGAA   
  
  
+ CCTTGGTTTT GCTTCGGAGA CTGCTCGAGA TTCAGGAGCG TCGAGCACAG GCCTACGCCA AACTCAAAAG   
  
  
+ GTTCGATCTT TGGAGTGTTT ATGCTATTAG TTACTGTTTT TGATTCAGTT ACTTGGTGAT TGTTACTGAG   
  
  
+ GGATGTTGGA GTTAAGTAAC TGATAGTGTT GTTGAGGAAG CGATGTCTCA TAAAGTTTAG ATTTTTATCT   
  
  
+ GTTGATCTCT TGCTATGCTT TTGGAATTTG ATTAATCAGA GGGTTCTCAG AGTATGTGGA GACTAGTGGT   
  
  
+ GAAGCGCTTT ATCAGAAGCT CTGCAGCGAG ATAACTGCTG AGTTCAACGA GTGTTCCAAA CAAGTAACTG   
  
  
+ AGTTTTTTTT TCTTCTTTTG ATTGATTACC TTTATGATGT TGTTGAAGAT ACGGTGTTAA AGATTGCGTC   
  
  
+ TTTGTTTGAT GGCAATGCAA GGTACGCGAA ATGGAAACTC TGTTTCTGAA TCCTGACGTT GGAAGATCGG   
  
  
+ ATCTTGCTCA ACTGCTCAGT GACATTCAAA CTCAGGAGAA GCAGAAACTG CATCTGGTCT GATTCCTATA   
  
  
+ TACTCAATTG TTGAACATAA CTGTTAACTT GTTGGACAGA ACAAAAGTTT CTTACTTTAG TTAGTTTTTT   
  
  
+ TTTTTTTTTG CAGACGGTTA CAATACAGGT ACTGAAGAAG GCAGGGAGGC CGTCAGAACG AATGCTGACA   
  
  
+ CACGAGAACT GCAAGTTCAA GAAACCGATG CAGCACGAGT GTGTGCATCT TCATGAGATT ACAGAAGCTG   
  
  
+ AAGGAACAGA GGAAGCAGAG GCGGATGCAG AGTTTGACAA CGCTTTGAAG GAAGCAATCA GAGGAGTGCA   
  
  
+ AGACGCTGTG ACTTGCATCA ATGAGTATTT GGAAGACGTT AGGTACGAGA TTGCAGCTCT TGAAGCTGAT   
  
  
+ TAGTTGTTCT CAATCTCATC ACTTCTTTAC CCCTTTCGAC TGTTAACTTG CTATGTTGCT AATTAGCTTA   
  
  
+ AGATATAAGA TATTTAGAAA CTCAAAAGTT ATGACATTTC TTGTTGTCAA AGTTATCTAT GTTGATTACT   
  
  
+ GTGGGTAGTG GCTACAAATA CAATAGCAAA TTCCAAATTA TTTAAAACAC AATATTTGAT TTGGAAAACA   
  
  
+ ATTGATTTAA AGAGGAACTT TGTTACAAAT ATAAAAGTTA TTAGCATTTT CGGTTCAAAA TGCATAAGAT   
  
  
+ GAGTTGTGTT TTTTTTGTCG TGTACACATC AAAAACTGTC ATACGGACAA TAAGCTAGTC GCTTTGACCC   
  
  
+ ATTCACCGTA TGTCCCAATG TCCTTGACCC TGCCAAATGA TAAGAACGCC AGCACAAAGG TTTTATAAGT   
  
  
+ TTCTCAAAGA GAAACAACAC AATGGGAAAA CACACCATTG CTTGACTTAC AAATGGAGAT CGGTTGGATC   
  
  
+ AATCTCTGGA AACAGGTTGA AGTAGAATCT AAGCCCGTCT GGGCTCATGT CTCTACACAG CAGACCTGAT   
  
  
+ CAAAACAAAT GGGAGAATGA GAATCACATA ACTCTGAAAG AGTCTATCTT TCTACGAGGT GGATTCTGTT   
  
  
+ TACCTTTGGT CTCTGCAGAT AGTAGATGTT CCTTGGCCAT GGCTGGAGCG ATTTTAAGCG TCAAAGCTGC   
  
  
+ ATCACTAGCA GTGACTCCAG TTCTTAAAGT TTCGGTTTTA GTCACAAGCG TTCT  

- CTTGACAATT TCGGTTAGTA AATTTTATTT TTCAGTTTGG CCAAATTTGG CCAAAAGTTA TTCAATTATT   
  
  
- GGCCTGAGCC ACAATTTCTC TAATTTAAAT TGCCAAATTG AGTCACTCTA GCCAAAGCGA AAGAGAGACA   
  
  
- GCACAAACTC TTCAACTTTC TCTCCTCCTA CTCGCGACTT CTAAAGGTCT TCTTCCAAAG CTAGTCTCTG   
  
  
- AGGAGGCAGC GGCCTCTTTA CCTTTAGCTT ACGCCGCCCA GATGATCGAG GCGCCAACCA AGCAGAGCTT   
  
  
- GGAACCAAAA CGAAGCCTCT GACGAGCTCT AAGTCCTCGC AGCTCGTGTC CGGATGCGGT TTGAGTTTTC   
  
  
- CAAGCTAGAA ACCTCACAAA TACGATAATC AATGACAAAA ACTAAGTCAA TGAACCACTA ACAATGACTC   
  
  
- CCTACAACCT CAATTCATTG ACTATCACAA CAACTCCTTC GCTACAGAGT ATTTCAAATC TAAAAATAGA   
  
  
- CAACTAGAGA ACGATACGAA AACCTTAAAC TAATTAGTCT CCCAAGAGTC TCATACACCT CTGATCACCA   
  
  
- CTTCGCGAAA TAGTCTTCGA GACGTCGCTC TATTGACGAC TCAAGTTGCT CACAAGGTTT GTTCATTGAC   
  
  
- TCAAAAAAAA AGAAGAAAAC TAACTAATGG AAATACTACA ACAACTTCTA TGCCACAATT TCTAACGCAG   
  
  
- AAACAAACTA CCGTTACGTT CCATGCGCTT TACCTTTGAG ACAAAGACTT AGGACTGCAA CCTTCTAGCC   
  
  
- TAGAACGAGT TGACGAGTCA CTGTAAGTTT GAGTCCTCTT CGTCTTTGAC GTAGACCAGA CTAAGGATAT   
  
  
- ATGAGTTAAC AACTTGTATT GACAATTGAA CAACCTGTCT TGTTTTCAAA GAATGAAATC AATCAAAAAA   
  
  
- AAAAAAAAAC GTCTGCCAAT GTTATGTCCA TGACTTCTTC CGTCCCTCCG GCAGTCTTGC TTACGACTGT   
  
  
- GTGCTCTTGA CGTTCAAGTT CTTTGGCTAC GTCGTGCTCA CACACGTAGA AGTACTCTAA TGTCTTCGAC   
  
  
- TTCCTTGTCT CCTTCGTCTC CGCCTACGTC TCAAACTGTT GCGAAACTTC CTTCGTTAGT CTCCTCACGT   
  
  
- TCTGCGACAC TGAACGTAGT TACTCATAAA CCTTCTGCAA TCCATGCTCT AACGTCGAGA ACTTCGACTA   
  
  
- ATCAACAAGA GTTAGAGTAG TGAAGAAATG GGGAAAGCTG ACAATTGAAC GATACAACGA TTAATCGAAT   
  
  
- TCTATATTCT ATAAATCTTT GAGTTTTCAA TACTGTAAAG AACAACAGTT TCAATAGATA CAACTAATGA   
  
  
- CACCCATCAC CGATGTTTAT GTTATCGTTT AAGGTTTAAT AAATTTTGTG TTATAAACTA AACCTTTTGT   
  
  
- TAACTAAATT TCTCCTTGAA ACAATGTTTA TATTTTCAAT AATCGTAAAA GCCAAGTTTT ACGTATTCTA   
  
  
- CTCAACACAA AAAAAACAGC ACATGTGTAG TTTTTGACAG TATGCCTGTT ATTCGATCAG CGAAACTGGG   
  
  
- TAAGTGGCAT ACAGGGTTAC AGGAACTGGG ACGGTTTACT ATTCTTGCGG TCGTGTTTCC AAAATATTCA   
  
  
- AAGAGTTTCT CTTTGTTGTG TTACCCTTTT GTGTGGTAAC GAACTGAATG TTTACCTCTA GCCAACCTAG   
  
  
- TTAGAGACCT TTGTCCAACT TCATCTTAGA TTCGGGCAGA CCCGAGTACA GAGATGTGTC GTCTGGACTA   
  
  
- GTTTTGTTTA CCCTCTTACT CTTAGTGTAT TGAGACTTTC TCAGATAGAA AGATGCTCCA CCTAAGACAA   
  
  
- ATGGAAACCA GAGACGTCTA TCATCTACAA GGAACCGGTA CCGACCTCGC TAAAATTCGC AGTTTCGACG   
  
  
- TAGTGATCGT CACTGAGGTC AAGAATTTCA AAGCCAAAAT CAGTGTTCGC AAGA

+     GARE-motif

| Site Name | Organism | Position | Strand | Matrix score. | sequence | function |
| --- | --- | --- | --- | --- | --- | --- |
| GARE-motif | Brassica oleracea | 488 | + | 7 | TCTGTTG | gibberellin-responsive element |

>PlantCARE\_8972   
+ GAACTGTTAA AGCCAATCAT TTAAAATAAA AAGTCAAACC GGTTTAAACC GGTTTTCAAT AAGTTAATAA   
  
  
+ CCGGACTCGG TGTTAAAGAG ATTAAATTTA ACGGTTTAAC TCAGTGAGAT CGGTTTCGCT TTCTCTCTGT   
  
  
+ CGTGTTTGAG AAGTTGAAAG AGAGGAGGAT GAGCGCTGAA GATTTCCAGA AGAAGGTTTC GATCAGAGAC   
  
  
+ TCCTCCGTCG CCGGAGAAAT GGAAATCGAA TGCGGCGGGT CTACTAGCTC CGCGGTTGGT TCGTCTCGAA   
  
  
+ CCTTGGTTTT GCTTCGGAGA CTGCTCGAGA TTCAGGAGCG TCGAGCACAG GCCTACGCCA AACTCAAAAG   
  
  
+ GTTCGATCTT TGGAGTGTTT ATGCTATTAG TTACTGTTTT TGATTCAGTT ACTTGGTGAT TGTTACTGAG   
  
  
+ GGATGTTGGA GTTAAGTAAC TGATAGTGTT GTTGAGGAAG CGATGTCTCA TAAAGTTTAG ATTTTTATCT   
  
  
+ GTTGATCTCT TGCTATGCTT TTGGAATTTG ATTAATCAGA GGGTTCTCAG AGTATGTGGA GACTAGTGGT   
  
  
+ GAAGCGCTTT ATCAGAAGCT CTGCAGCGAG ATAACTGCTG AGTTCAACGA GTGTTCCAAA CAAGTAACTG   
  
  
+ AGTTTTTTTT TCTTCTTTTG ATTGATTACC TTTATGATGT TGTTGAAGAT ACGGTGTTAA AGATTGCGTC   
  
  
+ TTTGTTTGAT GGCAATGCAA GGTACGCGAA ATGGAAACTC TGTTTCTGAA TCCTGACGTT GGAAGATCGG   
  
  
+ ATCTTGCTCA ACTGCTCAGT GACATTCAAA CTCAGGAGAA GCAGAAACTG CATCTGGTCT GATTCCTATA   
  
  
+ TACTCAATTG TTGAACATAA CTGTTAACTT GTTGGACAGA ACAAAAGTTT CTTACTTTAG TTAGTTTTTT   
  
  
+ TTTTTTTTTG CAGACGGTTA CAATACAGGT ACTGAAGAAG GCAGGGAGGC CGTCAGAACG AATGCTGACA   
  
  
+ CACGAGAACT GCAAGTTCAA GAAACCGATG CAGCACGAGT GTGTGCATCT TCATGAGATT ACAGAAGCTG   
  
  
+ AAGGAACAGA GGAAGCAGAG GCGGATGCAG AGTTTGACAA CGCTTTGAAG GAAGCAATCA GAGGAGTGCA   
  
  
+ AGACGCTGTG ACTTGCATCA ATGAGTATTT GGAAGACGTT AGGTACGAGA TTGCAGCTCT TGAAGCTGAT   
  
  
+ TAGTTGTTCT CAATCTCATC ACTTCTTTAC CCCTTTCGAC TGTTAACTTG CTATGTTGCT AATTAGCTTA   
  
  
+ AGATATAAGA TATTTAGAAA CTCAAAAGTT ATGACATTTC TTGTTGTCAA AGTTATCTAT GTTGATTACT   
  
  
+ GTGGGTAGTG GCTACAAATA CAATAGCAAA TTCCAAATTA TTTAAAACAC AATATTTGAT TTGGAAAACA   
  
  
+ ATTGATTTAA AGAGGAACTT TGTTACAAAT ATAAAAGTTA TTAGCATTTT CGGTTCAAAA TGCATAAGAT   
  
  
+ GAGTTGTGTT TTTTTTGTCG TGTACACATC AAAAACTGTC ATACGGACAA TAAGCTAGTC GCTTTGACCC   
  
  
+ ATTCACCGTA TGTCCCAATG TCCTTGACCC TGCCAAATGA TAAGAACGCC AGCACAAAGG TTTTATAAGT   
  
  
+ TTCTCAAAGA GAAACAACAC AATGGGAAAA CACACCATTG CTTGACTTAC AAATGGAGAT CGGTTGGATC   
  
  
+ AATCTCTGGA AACAGGTTGA AGTAGAATCT AAGCCCGTCT GGGCTCATGT CTCTACACAG CAGACCTGAT   
  
  
+ CAAAACAAAT GGGAGAATGA GAATCACATA ACTCTGAAAG AGTCTATCTT TCTACGAGGT GGATTCTGTT   
  
  
+ TACCTTTGGT CTCTGCAGAT AGTAGATGTT CCTTGGCCAT GGCTGGAGCG ATTTTAAGCG TCAAAGCTGC   
  
  
+ ATCACTAGCA GTGACTCCAG TTCTTAAAGT TTCGGTTTTA GTCACAAGCG TTCT  

- CTTGACAATT TCGGTTAGTA AATTTTATTT TTCAGTTTGG CCAAATTTGG CCAAAAGTTA TTCAATTATT   
  
  
- GGCCTGAGCC ACAATTTCTC TAATTTAAAT TGCCAAATTG AGTCACTCTA GCCAAAGCGA AAGAGAGACA   
  
  
- GCACAAACTC TTCAACTTTC TCTCCTCCTA CTCGCGACTT CTAAAGGTCT TCTTCCAAAG CTAGTCTCTG   
  
  
- AGGAGGCAGC GGCCTCTTTA CCTTTAGCTT ACGCCGCCCA GATGATCGAG GCGCCAACCA AGCAGAGCTT   
  
  
- GGAACCAAAA CGAAGCCTCT GACGAGCTCT AAGTCCTCGC AGCTCGTGTC CGGATGCGGT TTGAGTTTTC   
  
  
- CAAGCTAGAA ACCTCACAAA TACGATAATC AATGACAAAA ACTAAGTCAA TGAACCACTA ACAATGACTC   
  
  
- CCTACAACCT CAATTCATTG ACTATCACAA CAACTCCTTC GCTACAGAGT ATTTCAAATC TAAAAATAGA   
  
  
- CAACTAGAGA ACGATACGAA AACCTTAAAC TAATTAGTCT CCCAAGAGTC TCATACACCT CTGATCACCA   
  
  
- CTTCGCGAAA TAGTCTTCGA GACGTCGCTC TATTGACGAC TCAAGTTGCT CACAAGGTTT GTTCATTGAC   
  
  
- TCAAAAAAAA AGAAGAAAAC TAACTAATGG AAATACTACA ACAACTTCTA TGCCACAATT TCTAACGCAG   
  
  
- AAACAAACTA CCGTTACGTT CCATGCGCTT TACCTTTGAG ACAAAGACTT AGGACTGCAA CCTTCTAGCC   
  
  
- TAGAACGAGT TGACGAGTCA CTGTAAGTTT GAGTCCTCTT CGTCTTTGAC GTAGACCAGA CTAAGGATAT   
  
  
- ATGAGTTAAC AACTTGTATT GACAATTGAA CAACCTGTCT TGTTTTCAAA GAATGAAATC AATCAAAAAA   
  
  
- AAAAAAAAAC GTCTGCCAAT GTTATGTCCA TGACTTCTTC CGTCCCTCCG GCAGTCTTGC TTACGACTGT   
  
  
- GTGCTCTTGA CGTTCAAGTT CTTTGGCTAC GTCGTGCTCA CACACGTAGA AGTACTCTAA TGTCTTCGAC   
  
  
- TTCCTTGTCT CCTTCGTCTC CGCCTACGTC TCAAACTGTT GCGAAACTTC CTTCGTTAGT CTCCTCACGT   
  
  
- TCTGCGACAC TGAACGTAGT TACTCATAAA CCTTCTGCAA TCCATGCTCT AACGTCGAGA ACTTCGACTA   
  
  
- ATCAACAAGA GTTAGAGTAG TGAAGAAATG GGGAAAGCTG ACAATTGAAC GATACAACGA TTAATCGAAT   
  
  
- TCTATATTCT ATAAATCTTT GAGTTTTCAA TACTGTAAAG AACAACAGTT TCAATAGATA CAACTAATGA   
  
  
- CACCCATCAC CGATGTTTAT GTTATCGTTT AAGGTTTAAT AAATTTTGTG TTATAAACTA AACCTTTTGT   
  
  
- TAACTAAATT TCTCCTTGAA ACAATGTTTA TATTTTCAAT AATCGTAAAA GCCAAGTTTT ACGTATTCTA   
  
  
- CTCAACACAA AAAAAACAGC ACATGTGTAG TTTTTGACAG TATGCCTGTT ATTCGATCAG CGAAACTGGG   
  
  
- TAAGTGGCAT ACAGGGTTAC AGGAACTGGG ACGGTTTACT ATTCTTGCGG TCGTGTTTCC AAAATATTCA   
  
  
- AAGAGTTTCT CTTTGTTGTG TTACCCTTTT GTGTGGTAAC GAACTGAATG TTTACCTCTA GCCAACCTAG   
  
  
- TTAGAGACCT TTGTCCAACT TCATCTTAGA TTCGGGCAGA CCCGAGTACA GAGATGTGTC GTCTGGACTA   
  
  
- GTTTTGTTTA CCCTCTTACT CTTAGTGTAT TGAGACTTTC TCAGATAGAA AGATGCTCCA CCTAAGACAA   
  
  
- ATGGAAACCA GAGACGTCTA TCATCTACAA GGAACCGGTA CCGACCTCGC TAAAATTCGC AGTTTCGACG   
  
  
- TAGTGATCGT CACTGAGGTC AAGAATTTCA AAGCCAAAAT CAGTGTTCGC AAGA

+     LAMP-element

| Site Name | Organism | Position | Strand | Matrix score. | sequence | function |
| --- | --- | --- | --- | --- | --- | --- |
| LAMP-element | Pisum sativum | 567 | + | 8 | CTTTATCA | part of a light responsive element |

>PlantCARE\_8972   
+ GAACTGTTAA AGCCAATCAT TTAAAATAAA AAGTCAAACC GGTTTAAACC GGTTTTCAAT AAGTTAATAA   
  
  
+ CCGGACTCGG TGTTAAAGAG ATTAAATTTA ACGGTTTAAC TCAGTGAGAT CGGTTTCGCT TTCTCTCTGT   
  
  
+ CGTGTTTGAG AAGTTGAAAG AGAGGAGGAT GAGCGCTGAA GATTTCCAGA AGAAGGTTTC GATCAGAGAC   
  
  
+ TCCTCCGTCG CCGGAGAAAT GGAAATCGAA TGCGGCGGGT CTACTAGCTC CGCGGTTGGT TCGTCTCGAA   
  
  
+ CCTTGGTTTT GCTTCGGAGA CTGCTCGAGA TTCAGGAGCG TCGAGCACAG GCCTACGCCA AACTCAAAAG   
  
  
+ GTTCGATCTT TGGAGTGTTT ATGCTATTAG TTACTGTTTT TGATTCAGTT ACTTGGTGAT TGTTACTGAG   
  
  
+ GGATGTTGGA GTTAAGTAAC TGATAGTGTT GTTGAGGAAG CGATGTCTCA TAAAGTTTAG ATTTTTATCT   
  
  
+ GTTGATCTCT TGCTATGCTT TTGGAATTTG ATTAATCAGA GGGTTCTCAG AGTATGTGGA GACTAGTGGT   
  
  
+ GAAGCGCTTT ATCAGAAGCT CTGCAGCGAG ATAACTGCTG AGTTCAACGA GTGTTCCAAA CAAGTAACTG   
  
  
+ AGTTTTTTTT TCTTCTTTTG ATTGATTACC TTTATGATGT TGTTGAAGAT ACGGTGTTAA AGATTGCGTC   
  
  
+ TTTGTTTGAT GGCAATGCAA GGTACGCGAA ATGGAAACTC TGTTTCTGAA TCCTGACGTT GGAAGATCGG   
  
  
+ ATCTTGCTCA ACTGCTCAGT GACATTCAAA CTCAGGAGAA GCAGAAACTG CATCTGGTCT GATTCCTATA   
  
  
+ TACTCAATTG TTGAACATAA CTGTTAACTT GTTGGACAGA ACAAAAGTTT CTTACTTTAG TTAGTTTTTT   
  
  
+ TTTTTTTTTG CAGACGGTTA CAATACAGGT ACTGAAGAAG GCAGGGAGGC CGTCAGAACG AATGCTGACA   
  
  
+ CACGAGAACT GCAAGTTCAA GAAACCGATG CAGCACGAGT GTGTGCATCT TCATGAGATT ACAGAAGCTG   
  
  
+ AAGGAACAGA GGAAGCAGAG GCGGATGCAG AGTTTGACAA CGCTTTGAAG GAAGCAATCA GAGGAGTGCA   
  
  
+ AGACGCTGTG ACTTGCATCA ATGAGTATTT GGAAGACGTT AGGTACGAGA TTGCAGCTCT TGAAGCTGAT   
  
  
+ TAGTTGTTCT CAATCTCATC ACTTCTTTAC CCCTTTCGAC TGTTAACTTG CTATGTTGCT AATTAGCTTA   
  
  
+ AGATATAAGA TATTTAGAAA CTCAAAAGTT ATGACATTTC TTGTTGTCAA AGTTATCTAT GTTGATTACT   
  
  
+ GTGGGTAGTG GCTACAAATA CAATAGCAAA TTCCAAATTA TTTAAAACAC AATATTTGAT TTGGAAAACA   
  
  
+ ATTGATTTAA AGAGGAACTT TGTTACAAAT ATAAAAGTTA TTAGCATTTT CGGTTCAAAA TGCATAAGAT   
  
  
+ GAGTTGTGTT TTTTTTGTCG TGTACACATC AAAAACTGTC ATACGGACAA TAAGCTAGTC GCTTTGACCC   
  
  
+ ATTCACCGTA TGTCCCAATG TCCTTGACCC TGCCAAATGA TAAGAACGCC AGCACAAAGG TTTTATAAGT   
  
  
+ TTCTCAAAGA GAAACAACAC AATGGGAAAA CACACCATTG CTTGACTTAC AAATGGAGAT CGGTTGGATC   
  
  
+ AATCTCTGGA AACAGGTTGA AGTAGAATCT AAGCCCGTCT GGGCTCATGT CTCTACACAG CAGACCTGAT   
  
  
+ CAAAACAAAT GGGAGAATGA GAATCACATA ACTCTGAAAG AGTCTATCTT TCTACGAGGT GGATTCTGTT   
  
  
+ TACCTTTGGT CTCTGCAGAT AGTAGATGTT CCTTGGCCAT GGCTGGAGCG ATTTTAAGCG TCAAAGCTGC   
  
  
+ ATCACTAGCA GTGACTCCAG TTCTTAAAGT TTCGGTTTTA GTCACAAGCG TTCT  

- CTTGACAATT TCGGTTAGTA AATTTTATTT TTCAGTTTGG CCAAATTTGG CCAAAAGTTA TTCAATTATT   
  
  
- GGCCTGAGCC ACAATTTCTC TAATTTAAAT TGCCAAATTG AGTCACTCTA GCCAAAGCGA AAGAGAGACA   
  
  
- GCACAAACTC TTCAACTTTC TCTCCTCCTA CTCGCGACTT CTAAAGGTCT TCTTCCAAAG CTAGTCTCTG   
  
  
- AGGAGGCAGC GGCCTCTTTA CCTTTAGCTT ACGCCGCCCA GATGATCGAG GCGCCAACCA AGCAGAGCTT   
  
  
- GGAACCAAAA CGAAGCCTCT GACGAGCTCT AAGTCCTCGC AGCTCGTGTC CGGATGCGGT TTGAGTTTTC   
  
  
- CAAGCTAGAA ACCTCACAAA TACGATAATC AATGACAAAA ACTAAGTCAA TGAACCACTA ACAATGACTC   
  
  
- CCTACAACCT CAATTCATTG ACTATCACAA CAACTCCTTC GCTACAGAGT ATTTCAAATC TAAAAATAGA   
  
  
- CAACTAGAGA ACGATACGAA AACCTTAAAC TAATTAGTCT CCCAAGAGTC TCATACACCT CTGATCACCA   
  
  
- CTTCGCGAAA TAGTCTTCGA GACGTCGCTC TATTGACGAC TCAAGTTGCT CACAAGGTTT GTTCATTGAC   
  
  
- TCAAAAAAAA AGAAGAAAAC TAACTAATGG AAATACTACA ACAACTTCTA TGCCACAATT TCTAACGCAG   
  
  
- AAACAAACTA CCGTTACGTT CCATGCGCTT TACCTTTGAG ACAAAGACTT AGGACTGCAA CCTTCTAGCC   
  
  
- TAGAACGAGT TGACGAGTCA CTGTAAGTTT GAGTCCTCTT CGTCTTTGAC GTAGACCAGA CTAAGGATAT   
  
  
- ATGAGTTAAC AACTTGTATT GACAATTGAA CAACCTGTCT TGTTTTCAAA GAATGAAATC AATCAAAAAA   
  
  
- AAAAAAAAAC GTCTGCCAAT GTTATGTCCA TGACTTCTTC CGTCCCTCCG GCAGTCTTGC TTACGACTGT   
  
  
- GTGCTCTTGA CGTTCAAGTT CTTTGGCTAC GTCGTGCTCA CACACGTAGA AGTACTCTAA TGTCTTCGAC   
  
  
- TTCCTTGTCT CCTTCGTCTC CGCCTACGTC TCAAACTGTT GCGAAACTTC CTTCGTTAGT CTCCTCACGT   
  
  
- TCTGCGACAC TGAACGTAGT TACTCATAAA CCTTCTGCAA TCCATGCTCT AACGTCGAGA ACTTCGACTA   
  
  
- ATCAACAAGA GTTAGAGTAG TGAAGAAATG GGGAAAGCTG ACAATTGAAC GATACAACGA TTAATCGAAT   
  
  
- TCTATATTCT ATAAATCTTT GAGTTTTCAA TACTGTAAAG AACAACAGTT TCAATAGATA CAACTAATGA   
  
  
- CACCCATCAC CGATGTTTAT GTTATCGTTT AAGGTTTAAT AAATTTTGTG TTATAAACTA AACCTTTTGT   
  
  
- TAACTAAATT TCTCCTTGAA ACAATGTTTA TATTTTCAAT AATCGTAAAA GCCAAGTTTT ACGTATTCTA   
  
  
- CTCAACACAA AAAAAACAGC ACATGTGTAG TTTTTGACAG TATGCCTGTT ATTCGATCAG CGAAACTGGG   
  
  
- TAAGTGGCAT ACAGGGTTAC AGGAACTGGG ACGGTTTACT ATTCTTGCGG TCGTGTTTCC AAAATATTCA   
  
  
- AAGAGTTTCT CTTTGTTGTG TTACCCTTTT GTGTGGTAAC GAACTGAATG TTTACCTCTA GCCAACCTAG   
  
  
- TTAGAGACCT TTGTCCAACT TCATCTTAGA TTCGGGCAGA CCCGAGTACA GAGATGTGTC GTCTGGACTA   
  
  
- GTTTTGTTTA CCCTCTTACT CTTAGTGTAT TGAGACTTTC TCAGATAGAA AGATGCTCCA CCTAAGACAA   
  
  
- ATGGAAACCA GAGACGTCTA TCATCTACAA GGAACCGGTA CCGACCTCGC TAAAATTCGC AGTTTCGACG   
  
  
- TAGTGATCGT CACTGAGGTC AAGAATTTCA AAGCCAAAAT CAGTGTTCGC AAGA

+     LTR

| Site Name | Organism | Position | Strand | Matrix score. | sequence | function |
| --- | --- | --- | --- | --- | --- | --- |
| LTR | Hordeum vulgare | 1448 | - | 6 | CCGAAA | cis-acting element involved in low-temperature responsiveness |
| LTR | Hordeum vulgare | 1920 | - | 6 | CCGAAA | cis-acting element involved in low-temperature responsiveness |

>PlantCARE\_8972   
+ GAACTGTTAA AGCCAATCAT TTAAAATAAA AAGTCAAACC GGTTTAAACC GGTTTTCAAT AAGTTAATAA   
  
  
+ CCGGACTCGG TGTTAAAGAG ATTAAATTTA ACGGTTTAAC TCAGTGAGAT CGGTTTCGCT TTCTCTCTGT   
  
  
+ CGTGTTTGAG AAGTTGAAAG AGAGGAGGAT GAGCGCTGAA GATTTCCAGA AGAAGGTTTC GATCAGAGAC   
  
  
+ TCCTCCGTCG CCGGAGAAAT GGAAATCGAA TGCGGCGGGT CTACTAGCTC CGCGGTTGGT TCGTCTCGAA   
  
  
+ CCTTGGTTTT GCTTCGGAGA CTGCTCGAGA TTCAGGAGCG TCGAGCACAG GCCTACGCCA AACTCAAAAG   
  
  
+ GTTCGATCTT TGGAGTGTTT ATGCTATTAG TTACTGTTTT TGATTCAGTT ACTTGGTGAT TGTTACTGAG   
  
  
+ GGATGTTGGA GTTAAGTAAC TGATAGTGTT GTTGAGGAAG CGATGTCTCA TAAAGTTTAG ATTTTTATCT   
  
  
+ GTTGATCTCT TGCTATGCTT TTGGAATTTG ATTAATCAGA GGGTTCTCAG AGTATGTGGA GACTAGTGGT   
  
  
+ GAAGCGCTTT ATCAGAAGCT CTGCAGCGAG ATAACTGCTG AGTTCAACGA GTGTTCCAAA CAAGTAACTG   
  
  
+ AGTTTTTTTT TCTTCTTTTG ATTGATTACC TTTATGATGT TGTTGAAGAT ACGGTGTTAA AGATTGCGTC   
  
  
+ TTTGTTTGAT GGCAATGCAA GGTACGCGAA ATGGAAACTC TGTTTCTGAA TCCTGACGTT GGAAGATCGG   
  
  
+ ATCTTGCTCA ACTGCTCAGT GACATTCAAA CTCAGGAGAA GCAGAAACTG CATCTGGTCT GATTCCTATA   
  
  
+ TACTCAATTG TTGAACATAA CTGTTAACTT GTTGGACAGA ACAAAAGTTT CTTACTTTAG TTAGTTTTTT   
  
  
+ TTTTTTTTTG CAGACGGTTA CAATACAGGT ACTGAAGAAG GCAGGGAGGC CGTCAGAACG AATGCTGACA   
  
  
+ CACGAGAACT GCAAGTTCAA GAAACCGATG CAGCACGAGT GTGTGCATCT TCATGAGATT ACAGAAGCTG   
  
  
+ AAGGAACAGA GGAAGCAGAG GCGGATGCAG AGTTTGACAA CGCTTTGAAG GAAGCAATCA GAGGAGTGCA   
  
  
+ AGACGCTGTG ACTTGCATCA ATGAGTATTT GGAAGACGTT AGGTACGAGA TTGCAGCTCT TGAAGCTGAT   
  
  
+ TAGTTGTTCT CAATCTCATC ACTTCTTTAC CCCTTTCGAC TGTTAACTTG CTATGTTGCT AATTAGCTTA   
  
  
+ AGATATAAGA TATTTAGAAA CTCAAAAGTT ATGACATTTC TTGTTGTCAA AGTTATCTAT GTTGATTACT   
  
  
+ GTGGGTAGTG GCTACAAATA CAATAGCAAA TTCCAAATTA TTTAAAACAC AATATTTGAT TTGGAAAACA   
  
  
+ ATTGATTTAA AGAGGAACTT TGTTACAAAT ATAAAAGTTA TTAGCATTTT CGGTTCAAAA TGCATAAGAT   
  
  
+ GAGTTGTGTT TTTTTTGTCG TGTACACATC AAAAACTGTC ATACGGACAA TAAGCTAGTC GCTTTGACCC   
  
  
+ ATTCACCGTA TGTCCCAATG TCCTTGACCC TGCCAAATGA TAAGAACGCC AGCACAAAGG TTTTATAAGT   
  
  
+ TTCTCAAAGA GAAACAACAC AATGGGAAAA CACACCATTG CTTGACTTAC AAATGGAGAT CGGTTGGATC   
  
  
+ AATCTCTGGA AACAGGTTGA AGTAGAATCT AAGCCCGTCT GGGCTCATGT CTCTACACAG CAGACCTGAT   
  
  
+ CAAAACAAAT GGGAGAATGA GAATCACATA ACTCTGAAAG AGTCTATCTT TCTACGAGGT GGATTCTGTT   
  
  
+ TACCTTTGGT CTCTGCAGAT AGTAGATGTT CCTTGGCCAT GGCTGGAGCG ATTTTAAGCG TCAAAGCTGC   
  
  
+ ATCACTAGCA GTGACTCCAG TTCTTAAAGT TTCGGTTTTA GTCACAAGCG TTCT  

- CTTGACAATT TCGGTTAGTA AATTTTATTT TTCAGTTTGG CCAAATTTGG CCAAAAGTTA TTCAATTATT   
  
  
- GGCCTGAGCC ACAATTTCTC TAATTTAAAT TGCCAAATTG AGTCACTCTA GCCAAAGCGA AAGAGAGACA   
  
  
- GCACAAACTC TTCAACTTTC TCTCCTCCTA CTCGCGACTT CTAAAGGTCT TCTTCCAAAG CTAGTCTCTG   
  
  
- AGGAGGCAGC GGCCTCTTTA CCTTTAGCTT ACGCCGCCCA GATGATCGAG GCGCCAACCA AGCAGAGCTT   
  
  
- GGAACCAAAA CGAAGCCTCT GACGAGCTCT AAGTCCTCGC AGCTCGTGTC CGGATGCGGT TTGAGTTTTC   
  
  
- CAAGCTAGAA ACCTCACAAA TACGATAATC AATGACAAAA ACTAAGTCAA TGAACCACTA ACAATGACTC   
  
  
- CCTACAACCT CAATTCATTG ACTATCACAA CAACTCCTTC GCTACAGAGT ATTTCAAATC TAAAAATAGA   
  
  
- CAACTAGAGA ACGATACGAA AACCTTAAAC TAATTAGTCT CCCAAGAGTC TCATACACCT CTGATCACCA   
  
  
- CTTCGCGAAA TAGTCTTCGA GACGTCGCTC TATTGACGAC TCAAGTTGCT CACAAGGTTT GTTCATTGAC   
  
  
- TCAAAAAAAA AGAAGAAAAC TAACTAATGG AAATACTACA ACAACTTCTA TGCCACAATT TCTAACGCAG   
  
  
- AAACAAACTA CCGTTACGTT CCATGCGCTT TACCTTTGAG ACAAAGACTT AGGACTGCAA CCTTCTAGCC   
  
  
- TAGAACGAGT TGACGAGTCA CTGTAAGTTT GAGTCCTCTT CGTCTTTGAC GTAGACCAGA CTAAGGATAT   
  
  
- ATGAGTTAAC AACTTGTATT GACAATTGAA CAACCTGTCT TGTTTTCAAA GAATGAAATC AATCAAAAAA   
  
  
- AAAAAAAAAC GTCTGCCAAT GTTATGTCCA TGACTTCTTC CGTCCCTCCG GCAGTCTTGC TTACGACTGT   
  
  
- GTGCTCTTGA CGTTCAAGTT CTTTGGCTAC GTCGTGCTCA CACACGTAGA AGTACTCTAA TGTCTTCGAC   
  
  
- TTCCTTGTCT CCTTCGTCTC CGCCTACGTC TCAAACTGTT GCGAAACTTC CTTCGTTAGT CTCCTCACGT   
  
  
- TCTGCGACAC TGAACGTAGT TACTCATAAA CCTTCTGCAA TCCATGCTCT AACGTCGAGA ACTTCGACTA   
  
  
- ATCAACAAGA GTTAGAGTAG TGAAGAAATG GGGAAAGCTG ACAATTGAAC GATACAACGA TTAATCGAAT   
  
  
- TCTATATTCT ATAAATCTTT GAGTTTTCAA TACTGTAAAG AACAACAGTT TCAATAGATA CAACTAATGA   
  
  
- CACCCATCAC CGATGTTTAT GTTATCGTTT AAGGTTTAAT AAATTTTGTG TTATAAACTA AACCTTTTGT   
  
  
- TAACTAAATT TCTCCTTGAA ACAATGTTTA TATTTTCAAT AATCGTAAAA GCCAAGTTTT ACGTATTCTA   
  
  
- CTCAACACAA AAAAAACAGC ACATGTGTAG TTTTTGACAG TATGCCTGTT ATTCGATCAG CGAAACTGGG   
  
  
- TAAGTGGCAT ACAGGGTTAC AGGAACTGGG ACGGTTTACT ATTCTTGCGG TCGTGTTTCC AAAATATTCA   
  
  
- AAGAGTTTCT CTTTGTTGTG TTACCCTTTT GTGTGGTAAC GAACTGAATG TTTACCTCTA GCCAACCTAG   
  
  
- TTAGAGACCT TTGTCCAACT TCATCTTAGA TTCGGGCAGA CCCGAGTACA GAGATGTGTC GTCTGGACTA   
  
  
- GTTTTGTTTA CCCTCTTACT CTTAGTGTAT TGAGACTTTC TCAGATAGAA AGATGCTCCA CCTAAGACAA   
  
  
- ATGGAAACCA GAGACGTCTA TCATCTACAA GGAACCGGTA CCGACCTCGC TAAAATTCGC AGTTTCGACG   
  
  
- TAGTGATCGT CACTGAGGTC AAGAATTTCA AAGCCAAAAT CAGTGTTCGC AAGA

+     MBS

| Site Name | Organism | Position | Strand | Matrix score. | sequence | function |
| --- | --- | --- | --- | --- | --- | --- |
| MBS | Arabidopsis thaliana | 779 | + | 6 | CAACTG | MYB binding site involved in drought-inducibility |

>PlantCARE\_8972   
+ GAACTGTTAA AGCCAATCAT TTAAAATAAA AAGTCAAACC GGTTTAAACC GGTTTTCAAT AAGTTAATAA   
  
  
+ CCGGACTCGG TGTTAAAGAG ATTAAATTTA ACGGTTTAAC TCAGTGAGAT CGGTTTCGCT TTCTCTCTGT   
  
  
+ CGTGTTTGAG AAGTTGAAAG AGAGGAGGAT GAGCGCTGAA GATTTCCAGA AGAAGGTTTC GATCAGAGAC   
  
  
+ TCCTCCGTCG CCGGAGAAAT GGAAATCGAA TGCGGCGGGT CTACTAGCTC CGCGGTTGGT TCGTCTCGAA   
  
  
+ CCTTGGTTTT GCTTCGGAGA CTGCTCGAGA TTCAGGAGCG TCGAGCACAG GCCTACGCCA AACTCAAAAG   
  
  
+ GTTCGATCTT TGGAGTGTTT ATGCTATTAG TTACTGTTTT TGATTCAGTT ACTTGGTGAT TGTTACTGAG   
  
  
+ GGATGTTGGA GTTAAGTAAC TGATAGTGTT GTTGAGGAAG CGATGTCTCA TAAAGTTTAG ATTTTTATCT   
  
  
+ GTTGATCTCT TGCTATGCTT TTGGAATTTG ATTAATCAGA GGGTTCTCAG AGTATGTGGA GACTAGTGGT   
  
  
+ GAAGCGCTTT ATCAGAAGCT CTGCAGCGAG ATAACTGCTG AGTTCAACGA GTGTTCCAAA CAAGTAACTG   
  
  
+ AGTTTTTTTT TCTTCTTTTG ATTGATTACC TTTATGATGT TGTTGAAGAT ACGGTGTTAA AGATTGCGTC   
  
  
+ TTTGTTTGAT GGCAATGCAA GGTACGCGAA ATGGAAACTC TGTTTCTGAA TCCTGACGTT GGAAGATCGG   
  
  
+ ATCTTGCTCA ACTGCTCAGT GACATTCAAA CTCAGGAGAA GCAGAAACTG CATCTGGTCT GATTCCTATA   
  
  
+ TACTCAATTG TTGAACATAA CTGTTAACTT GTTGGACAGA ACAAAAGTTT CTTACTTTAG TTAGTTTTTT   
  
  
+ TTTTTTTTTG CAGACGGTTA CAATACAGGT ACTGAAGAAG GCAGGGAGGC CGTCAGAACG AATGCTGACA   
  
  
+ CACGAGAACT GCAAGTTCAA GAAACCGATG CAGCACGAGT GTGTGCATCT TCATGAGATT ACAGAAGCTG   
  
  
+ AAGGAACAGA GGAAGCAGAG GCGGATGCAG AGTTTGACAA CGCTTTGAAG GAAGCAATCA GAGGAGTGCA   
  
  
+ AGACGCTGTG ACTTGCATCA ATGAGTATTT GGAAGACGTT AGGTACGAGA TTGCAGCTCT TGAAGCTGAT   
  
  
+ TAGTTGTTCT CAATCTCATC ACTTCTTTAC CCCTTTCGAC TGTTAACTTG CTATGTTGCT AATTAGCTTA   
  
  
+ AGATATAAGA TATTTAGAAA CTCAAAAGTT ATGACATTTC TTGTTGTCAA AGTTATCTAT GTTGATTACT   
  
  
+ GTGGGTAGTG GCTACAAATA CAATAGCAAA TTCCAAATTA TTTAAAACAC AATATTTGAT TTGGAAAACA   
  
  
+ ATTGATTTAA AGAGGAACTT TGTTACAAAT ATAAAAGTTA TTAGCATTTT CGGTTCAAAA TGCATAAGAT   
  
  
+ GAGTTGTGTT TTTTTTGTCG TGTACACATC AAAAACTGTC ATACGGACAA TAAGCTAGTC GCTTTGACCC   
  
  
+ ATTCACCGTA TGTCCCAATG TCCTTGACCC TGCCAAATGA TAAGAACGCC AGCACAAAGG TTTTATAAGT   
  
  
+ TTCTCAAAGA GAAACAACAC AATGGGAAAA CACACCATTG CTTGACTTAC AAATGGAGAT CGGTTGGATC   
  
  
+ AATCTCTGGA AACAGGTTGA AGTAGAATCT AAGCCCGTCT GGGCTCATGT CTCTACACAG CAGACCTGAT   
  
  
+ CAAAACAAAT GGGAGAATGA GAATCACATA ACTCTGAAAG AGTCTATCTT TCTACGAGGT GGATTCTGTT   
  
  
+ TACCTTTGGT CTCTGCAGAT AGTAGATGTT CCTTGGCCAT GGCTGGAGCG ATTTTAAGCG TCAAAGCTGC   
  
  
+ ATCACTAGCA GTGACTCCAG TTCTTAAAGT TTCGGTTTTA GTCACAAGCG TTCT  

- CTTGACAATT TCGGTTAGTA AATTTTATTT TTCAGTTTGG CCAAATTTGG CCAAAAGTTA TTCAATTATT   
  
  
- GGCCTGAGCC ACAATTTCTC TAATTTAAAT TGCCAAATTG AGTCACTCTA GCCAAAGCGA AAGAGAGACA   
  
  
- GCACAAACTC TTCAACTTTC TCTCCTCCTA CTCGCGACTT CTAAAGGTCT TCTTCCAAAG CTAGTCTCTG   
  
  
- AGGAGGCAGC GGCCTCTTTA CCTTTAGCTT ACGCCGCCCA GATGATCGAG GCGCCAACCA AGCAGAGCTT   
  
  
- GGAACCAAAA CGAAGCCTCT GACGAGCTCT AAGTCCTCGC AGCTCGTGTC CGGATGCGGT TTGAGTTTTC   
  
  
- CAAGCTAGAA ACCTCACAAA TACGATAATC AATGACAAAA ACTAAGTCAA TGAACCACTA ACAATGACTC   
  
  
- CCTACAACCT CAATTCATTG ACTATCACAA CAACTCCTTC GCTACAGAGT ATTTCAAATC TAAAAATAGA   
  
  
- CAACTAGAGA ACGATACGAA AACCTTAAAC TAATTAGTCT CCCAAGAGTC TCATACACCT CTGATCACCA   
  
  
- CTTCGCGAAA TAGTCTTCGA GACGTCGCTC TATTGACGAC TCAAGTTGCT CACAAGGTTT GTTCATTGAC   
  
  
- TCAAAAAAAA AGAAGAAAAC TAACTAATGG AAATACTACA ACAACTTCTA TGCCACAATT TCTAACGCAG   
  
  
- AAACAAACTA CCGTTACGTT CCATGCGCTT TACCTTTGAG ACAAAGACTT AGGACTGCAA CCTTCTAGCC   
  
  
- TAGAACGAGT TGACGAGTCA CTGTAAGTTT GAGTCCTCTT CGTCTTTGAC GTAGACCAGA CTAAGGATAT   
  
  
- ATGAGTTAAC AACTTGTATT GACAATTGAA CAACCTGTCT TGTTTTCAAA GAATGAAATC AATCAAAAAA   
  
  
- AAAAAAAAAC GTCTGCCAAT GTTATGTCCA TGACTTCTTC CGTCCCTCCG GCAGTCTTGC TTACGACTGT   
  
  
- GTGCTCTTGA CGTTCAAGTT CTTTGGCTAC GTCGTGCTCA CACACGTAGA AGTACTCTAA TGTCTTCGAC   
  
  
- TTCCTTGTCT CCTTCGTCTC CGCCTACGTC TCAAACTGTT GCGAAACTTC CTTCGTTAGT CTCCTCACGT   
  
  
- TCTGCGACAC TGAACGTAGT TACTCATAAA CCTTCTGCAA TCCATGCTCT AACGTCGAGA ACTTCGACTA   
  
  
- ATCAACAAGA GTTAGAGTAG TGAAGAAATG GGGAAAGCTG ACAATTGAAC GATACAACGA TTAATCGAAT   
  
  
- TCTATATTCT ATAAATCTTT GAGTTTTCAA TACTGTAAAG AACAACAGTT TCAATAGATA CAACTAATGA   
  
  
- CACCCATCAC CGATGTTTAT GTTATCGTTT AAGGTTTAAT AAATTTTGTG TTATAAACTA AACCTTTTGT   
  
  
- TAACTAAATT TCTCCTTGAA ACAATGTTTA TATTTTCAAT AATCGTAAAA GCCAAGTTTT ACGTATTCTA   
  
  
- CTCAACACAA AAAAAACAGC ACATGTGTAG TTTTTGACAG TATGCCTGTT ATTCGATCAG CGAAACTGGG   
  
  
- TAAGTGGCAT ACAGGGTTAC AGGAACTGGG ACGGTTTACT ATTCTTGCGG TCGTGTTTCC AAAATATTCA   
  
  
- AAGAGTTTCT CTTTGTTGTG TTACCCTTTT GTGTGGTAAC GAACTGAATG TTTACCTCTA GCCAACCTAG   
  
  
- TTAGAGACCT TTGTCCAACT TCATCTTAGA TTCGGGCAGA CCCGAGTACA GAGATGTGTC GTCTGGACTA   
  
  
- GTTTTGTTTA CCCTCTTACT CTTAGTGTAT TGAGACTTTC TCAGATAGAA AGATGCTCCA CCTAAGACAA   
  
  
- ATGGAAACCA GAGACGTCTA TCATCTACAA GGAACCGGTA CCGACCTCGC TAAAATTCGC AGTTTCGACG   
  
  
- TAGTGATCGT CACTGAGGTC AAGAATTTCA AAGCCAAAAT CAGTGTTCGC AAGA

+     MYB

| Site Name | Organism | Position | Strand | Matrix score. | sequence | function |
| --- | --- | --- | --- | --- | --- | --- |
| MYB | Arabidopsis thaliana | 489 | - | 6 | CAACAG |  |

>PlantCARE\_8972   
+ GAACTGTTAA AGCCAATCAT TTAAAATAAA AAGTCAAACC GGTTTAAACC GGTTTTCAAT AAGTTAATAA   
  
  
+ CCGGACTCGG TGTTAAAGAG ATTAAATTTA ACGGTTTAAC TCAGTGAGAT CGGTTTCGCT TTCTCTCTGT   
  
  
+ CGTGTTTGAG AAGTTGAAAG AGAGGAGGAT GAGCGCTGAA GATTTCCAGA AGAAGGTTTC GATCAGAGAC   
  
  
+ TCCTCCGTCG CCGGAGAAAT GGAAATCGAA TGCGGCGGGT CTACTAGCTC CGCGGTTGGT TCGTCTCGAA   
  
  
+ CCTTGGTTTT GCTTCGGAGA CTGCTCGAGA TTCAGGAGCG TCGAGCACAG GCCTACGCCA AACTCAAAAG   
  
  
+ GTTCGATCTT TGGAGTGTTT ATGCTATTAG TTACTGTTTT TGATTCAGTT ACTTGGTGAT TGTTACTGAG   
  
  
+ GGATGTTGGA GTTAAGTAAC TGATAGTGTT GTTGAGGAAG CGATGTCTCA TAAAGTTTAG ATTTTTATCT   
  
  
+ GTTGATCTCT TGCTATGCTT TTGGAATTTG ATTAATCAGA GGGTTCTCAG AGTATGTGGA GACTAGTGGT   
  
  
+ GAAGCGCTTT ATCAGAAGCT CTGCAGCGAG ATAACTGCTG AGTTCAACGA GTGTTCCAAA CAAGTAACTG   
  
  
+ AGTTTTTTTT TCTTCTTTTG ATTGATTACC TTTATGATGT TGTTGAAGAT ACGGTGTTAA AGATTGCGTC   
  
  
+ TTTGTTTGAT GGCAATGCAA GGTACGCGAA ATGGAAACTC TGTTTCTGAA TCCTGACGTT GGAAGATCGG   
  
  
+ ATCTTGCTCA ACTGCTCAGT GACATTCAAA CTCAGGAGAA GCAGAAACTG CATCTGGTCT GATTCCTATA   
  
  
+ TACTCAATTG TTGAACATAA CTGTTAACTT GTTGGACAGA ACAAAAGTTT CTTACTTTAG TTAGTTTTTT   
  
  
+ TTTTTTTTTG CAGACGGTTA CAATACAGGT ACTGAAGAAG GCAGGGAGGC CGTCAGAACG AATGCTGACA   
  
  
+ CACGAGAACT GCAAGTTCAA GAAACCGATG CAGCACGAGT GTGTGCATCT TCATGAGATT ACAGAAGCTG   
  
  
+ AAGGAACAGA GGAAGCAGAG GCGGATGCAG AGTTTGACAA CGCTTTGAAG GAAGCAATCA GAGGAGTGCA   
  
  
+ AGACGCTGTG ACTTGCATCA ATGAGTATTT GGAAGACGTT AGGTACGAGA TTGCAGCTCT TGAAGCTGAT   
  
  
+ TAGTTGTTCT CAATCTCATC ACTTCTTTAC CCCTTTCGAC TGTTAACTTG CTATGTTGCT AATTAGCTTA   
  
  
+ AGATATAAGA TATTTAGAAA CTCAAAAGTT ATGACATTTC TTGTTGTCAA AGTTATCTAT GTTGATTACT   
  
  
+ GTGGGTAGTG GCTACAAATA CAATAGCAAA TTCCAAATTA TTTAAAACAC AATATTTGAT TTGGAAAACA   
  
  
+ ATTGATTTAA AGAGGAACTT TGTTACAAAT ATAAAAGTTA TTAGCATTTT CGGTTCAAAA TGCATAAGAT   
  
  
+ GAGTTGTGTT TTTTTTGTCG TGTACACATC AAAAACTGTC ATACGGACAA TAAGCTAGTC GCTTTGACCC   
  
  
+ ATTCACCGTA TGTCCCAATG TCCTTGACCC TGCCAAATGA TAAGAACGCC AGCACAAAGG TTTTATAAGT   
  
  
+ TTCTCAAAGA GAAACAACAC AATGGGAAAA CACACCATTG CTTGACTTAC AAATGGAGAT CGGTTGGATC   
  
  
+ AATCTCTGGA AACAGGTTGA AGTAGAATCT AAGCCCGTCT GGGCTCATGT CTCTACACAG CAGACCTGAT   
  
  
+ CAAAACAAAT GGGAGAATGA GAATCACATA ACTCTGAAAG AGTCTATCTT TCTACGAGGT GGATTCTGTT   
  
  
+ TACCTTTGGT CTCTGCAGAT AGTAGATGTT CCTTGGCCAT GGCTGGAGCG ATTTTAAGCG TCAAAGCTGC   
  
  
+ ATCACTAGCA GTGACTCCAG TTCTTAAAGT TTCGGTTTTA GTCACAAGCG TTCT  

- CTTGACAATT TCGGTTAGTA AATTTTATTT TTCAGTTTGG CCAAATTTGG CCAAAAGTTA TTCAATTATT   
  
  
- GGCCTGAGCC ACAATTTCTC TAATTTAAAT TGCCAAATTG AGTCACTCTA GCCAAAGCGA AAGAGAGACA   
  
  
- GCACAAACTC TTCAACTTTC TCTCCTCCTA CTCGCGACTT CTAAAGGTCT TCTTCCAAAG CTAGTCTCTG   
  
  
- AGGAGGCAGC GGCCTCTTTA CCTTTAGCTT ACGCCGCCCA GATGATCGAG GCGCCAACCA AGCAGAGCTT   
  
  
- GGAACCAAAA CGAAGCCTCT GACGAGCTCT AAGTCCTCGC AGCTCGTGTC CGGATGCGGT TTGAGTTTTC   
  
  
- CAAGCTAGAA ACCTCACAAA TACGATAATC AATGACAAAA ACTAAGTCAA TGAACCACTA ACAATGACTC   
  
  
- CCTACAACCT CAATTCATTG ACTATCACAA CAACTCCTTC GCTACAGAGT ATTTCAAATC TAAAAATAGA   
  
  
- CAACTAGAGA ACGATACGAA AACCTTAAAC TAATTAGTCT CCCAAGAGTC TCATACACCT CTGATCACCA   
  
  
- CTTCGCGAAA TAGTCTTCGA GACGTCGCTC TATTGACGAC TCAAGTTGCT CACAAGGTTT GTTCATTGAC   
  
  
- TCAAAAAAAA AGAAGAAAAC TAACTAATGG AAATACTACA ACAACTTCTA TGCCACAATT TCTAACGCAG   
  
  
- AAACAAACTA CCGTTACGTT CCATGCGCTT TACCTTTGAG ACAAAGACTT AGGACTGCAA CCTTCTAGCC   
  
  
- TAGAACGAGT TGACGAGTCA CTGTAAGTTT GAGTCCTCTT CGTCTTTGAC GTAGACCAGA CTAAGGATAT   
  
  
- ATGAGTTAAC AACTTGTATT GACAATTGAA CAACCTGTCT TGTTTTCAAA GAATGAAATC AATCAAAAAA   
  
  
- AAAAAAAAAC GTCTGCCAAT GTTATGTCCA TGACTTCTTC CGTCCCTCCG GCAGTCTTGC TTACGACTGT   
  
  
- GTGCTCTTGA CGTTCAAGTT CTTTGGCTAC GTCGTGCTCA CACACGTAGA AGTACTCTAA TGTCTTCGAC   
  
  
- TTCCTTGTCT CCTTCGTCTC CGCCTACGTC TCAAACTGTT GCGAAACTTC CTTCGTTAGT CTCCTCACGT   
  
  
- TCTGCGACAC TGAACGTAGT TACTCATAAA CCTTCTGCAA TCCATGCTCT AACGTCGAGA ACTTCGACTA   
  
  
- ATCAACAAGA GTTAGAGTAG TGAAGAAATG GGGAAAGCTG ACAATTGAAC GATACAACGA TTAATCGAAT   
  
  
- TCTATATTCT ATAAATCTTT GAGTTTTCAA TACTGTAAAG AACAACAGTT TCAATAGATA CAACTAATGA   
  
  
- CACCCATCAC CGATGTTTAT GTTATCGTTT AAGGTTTAAT AAATTTTGTG TTATAAACTA AACCTTTTGT   
  
  
- TAACTAAATT TCTCCTTGAA ACAATGTTTA TATTTTCAAT AATCGTAAAA GCCAAGTTTT ACGTATTCTA   
  
  
- CTCAACACAA AAAAAACAGC ACATGTGTAG TTTTTGACAG TATGCCTGTT ATTCGATCAG CGAAACTGGG   
  
  
- TAAGTGGCAT ACAGGGTTAC AGGAACTGGG ACGGTTTACT ATTCTTGCGG TCGTGTTTCC AAAATATTCA   
  
  
- AAGAGTTTCT CTTTGTTGTG TTACCCTTTT GTGTGGTAAC GAACTGAATG TTTACCTCTA GCCAACCTAG   
  
  
- TTAGAGACCT TTGTCCAACT TCATCTTAGA TTCGGGCAGA CCCGAGTACA GAGATGTGTC GTCTGGACTA   
  
  
- GTTTTGTTTA CCCTCTTACT CTTAGTGTAT TGAGACTTTC TCAGATAGAA AGATGCTCCA CCTAAGACAA   
  
  
- ATGGAAACCA GAGACGTCTA TCATCTACAA GGAACCGGTA CCGACCTCGC TAAAATTCGC AGTTTCGACG   
  
  
- TAGTGATCGT CACTGAGGTC AAGAATTTCA AAGCCAAAAT CAGTGTTCGC AAGA

+     MYC

| Site Name | Organism | Position | Strand | Matrix score. | sequence | function |
| --- | --- | --- | --- | --- | --- | --- |
| MYC | Arabidopsis thaliana | 845 | + | 6 | CAATTG |  |
| MYC | Arabidopsis thaliana | 1756 | - | 6 | CATTTG |  |
| MYC | Arabidopsis thaliana | 1574 | - | 6 | CATTTG |  |
| MYC | Arabidopsis thaliana | 1399 | - | 6 | CAATTG |  |
| MYC | Arabidopsis thaliana | 1660 | - | 6 | CATTTG |  |

>PlantCARE\_8972   
+ GAACTGTTAA AGCCAATCAT TTAAAATAAA AAGTCAAACC GGTTTAAACC GGTTTTCAAT AAGTTAATAA   
  
  
+ CCGGACTCGG TGTTAAAGAG ATTAAATTTA ACGGTTTAAC TCAGTGAGAT CGGTTTCGCT TTCTCTCTGT   
  
  
+ CGTGTTTGAG AAGTTGAAAG AGAGGAGGAT GAGCGCTGAA GATTTCCAGA AGAAGGTTTC GATCAGAGAC   
  
  
+ TCCTCCGTCG CCGGAGAAAT GGAAATCGAA TGCGGCGGGT CTACTAGCTC CGCGGTTGGT TCGTCTCGAA   
  
  
+ CCTTGGTTTT GCTTCGGAGA CTGCTCGAGA TTCAGGAGCG TCGAGCACAG GCCTACGCCA AACTCAAAAG   
  
  
+ GTTCGATCTT TGGAGTGTTT ATGCTATTAG TTACTGTTTT TGATTCAGTT ACTTGGTGAT TGTTACTGAG   
  
  
+ GGATGTTGGA GTTAAGTAAC TGATAGTGTT GTTGAGGAAG CGATGTCTCA TAAAGTTTAG ATTTTTATCT   
  
  
+ GTTGATCTCT TGCTATGCTT TTGGAATTTG ATTAATCAGA GGGTTCTCAG AGTATGTGGA GACTAGTGGT   
  
  
+ GAAGCGCTTT ATCAGAAGCT CTGCAGCGAG ATAACTGCTG AGTTCAACGA GTGTTCCAAA CAAGTAACTG   
  
  
+ AGTTTTTTTT TCTTCTTTTG ATTGATTACC TTTATGATGT TGTTGAAGAT ACGGTGTTAA AGATTGCGTC   
  
  
+ TTTGTTTGAT GGCAATGCAA GGTACGCGAA ATGGAAACTC TGTTTCTGAA TCCTGACGTT GGAAGATCGG   
  
  
+ ATCTTGCTCA ACTGCTCAGT GACATTCAAA CTCAGGAGAA GCAGAAACTG CATCTGGTCT GATTCCTATA   
  
  
+ TACTCAATTG TTGAACATAA CTGTTAACTT GTTGGACAGA ACAAAAGTTT CTTACTTTAG TTAGTTTTTT   
  
  
+ TTTTTTTTTG CAGACGGTTA CAATACAGGT ACTGAAGAAG GCAGGGAGGC CGTCAGAACG AATGCTGACA   
  
  
+ CACGAGAACT GCAAGTTCAA GAAACCGATG CAGCACGAGT GTGTGCATCT TCATGAGATT ACAGAAGCTG   
  
  
+ AAGGAACAGA GGAAGCAGAG GCGGATGCAG AGTTTGACAA CGCTTTGAAG GAAGCAATCA GAGGAGTGCA   
  
  
+ AGACGCTGTG ACTTGCATCA ATGAGTATTT GGAAGACGTT AGGTACGAGA TTGCAGCTCT TGAAGCTGAT   
  
  
+ TAGTTGTTCT CAATCTCATC ACTTCTTTAC CCCTTTCGAC TGTTAACTTG CTATGTTGCT AATTAGCTTA   
  
  
+ AGATATAAGA TATTTAGAAA CTCAAAAGTT ATGACATTTC TTGTTGTCAA AGTTATCTAT GTTGATTACT   
  
  
+ GTGGGTAGTG GCTACAAATA CAATAGCAAA TTCCAAATTA TTTAAAACAC AATATTTGAT TTGGAAAACA   
  
  
+ ATTGATTTAA AGAGGAACTT TGTTACAAAT ATAAAAGTTA TTAGCATTTT CGGTTCAAAA TGCATAAGAT   
  
  
+ GAGTTGTGTT TTTTTTGTCG TGTACACATC AAAAACTGTC ATACGGACAA TAAGCTAGTC GCTTTGACCC   
  
  
+ ATTCACCGTA TGTCCCAATG TCCTTGACCC TGCCAAATGA TAAGAACGCC AGCACAAAGG TTTTATAAGT   
  
  
+ TTCTCAAAGA GAAACAACAC AATGGGAAAA CACACCATTG CTTGACTTAC AAATGGAGAT CGGTTGGATC   
  
  
+ AATCTCTGGA AACAGGTTGA AGTAGAATCT AAGCCCGTCT GGGCTCATGT CTCTACACAG CAGACCTGAT   
  
  
+ CAAAACAAAT GGGAGAATGA GAATCACATA ACTCTGAAAG AGTCTATCTT TCTACGAGGT GGATTCTGTT   
  
  
+ TACCTTTGGT CTCTGCAGAT AGTAGATGTT CCTTGGCCAT GGCTGGAGCG ATTTTAAGCG TCAAAGCTGC   
  
  
+ ATCACTAGCA GTGACTCCAG TTCTTAAAGT TTCGGTTTTA GTCACAAGCG TTCT  

- CTTGACAATT TCGGTTAGTA AATTTTATTT TTCAGTTTGG CCAAATTTGG CCAAAAGTTA TTCAATTATT   
  
  
- GGCCTGAGCC ACAATTTCTC TAATTTAAAT TGCCAAATTG AGTCACTCTA GCCAAAGCGA AAGAGAGACA   
  
  
- GCACAAACTC TTCAACTTTC TCTCCTCCTA CTCGCGACTT CTAAAGGTCT TCTTCCAAAG CTAGTCTCTG   
  
  
- AGGAGGCAGC GGCCTCTTTA CCTTTAGCTT ACGCCGCCCA GATGATCGAG GCGCCAACCA AGCAGAGCTT   
  
  
- GGAACCAAAA CGAAGCCTCT GACGAGCTCT AAGTCCTCGC AGCTCGTGTC CGGATGCGGT TTGAGTTTTC   
  
  
- CAAGCTAGAA ACCTCACAAA TACGATAATC AATGACAAAA ACTAAGTCAA TGAACCACTA ACAATGACTC   
  
  
- CCTACAACCT CAATTCATTG ACTATCACAA CAACTCCTTC GCTACAGAGT ATTTCAAATC TAAAAATAGA   
  
  
- CAACTAGAGA ACGATACGAA AACCTTAAAC TAATTAGTCT CCCAAGAGTC TCATACACCT CTGATCACCA   
  
  
- CTTCGCGAAA TAGTCTTCGA GACGTCGCTC TATTGACGAC TCAAGTTGCT CACAAGGTTT GTTCATTGAC   
  
  
- TCAAAAAAAA AGAAGAAAAC TAACTAATGG AAATACTACA ACAACTTCTA TGCCACAATT TCTAACGCAG   
  
  
- AAACAAACTA CCGTTACGTT CCATGCGCTT TACCTTTGAG ACAAAGACTT AGGACTGCAA CCTTCTAGCC   
  
  
- TAGAACGAGT TGACGAGTCA CTGTAAGTTT GAGTCCTCTT CGTCTTTGAC GTAGACCAGA CTAAGGATAT   
  
  
- ATGAGTTAAC AACTTGTATT GACAATTGAA CAACCTGTCT TGTTTTCAAA GAATGAAATC AATCAAAAAA   
  
  
- AAAAAAAAAC GTCTGCCAAT GTTATGTCCA TGACTTCTTC CGTCCCTCCG GCAGTCTTGC TTACGACTGT   
  
  
- GTGCTCTTGA CGTTCAAGTT CTTTGGCTAC GTCGTGCTCA CACACGTAGA AGTACTCTAA TGTCTTCGAC   
  
  
- TTCCTTGTCT CCTTCGTCTC CGCCTACGTC TCAAACTGTT GCGAAACTTC CTTCGTTAGT CTCCTCACGT   
  
  
- TCTGCGACAC TGAACGTAGT TACTCATAAA CCTTCTGCAA TCCATGCTCT AACGTCGAGA ACTTCGACTA   
  
  
- ATCAACAAGA GTTAGAGTAG TGAAGAAATG GGGAAAGCTG ACAATTGAAC GATACAACGA TTAATCGAAT   
  
  
- TCTATATTCT ATAAATCTTT GAGTTTTCAA TACTGTAAAG AACAACAGTT TCAATAGATA CAACTAATGA   
  
  
- CACCCATCAC CGATGTTTAT GTTATCGTTT AAGGTTTAAT AAATTTTGTG TTATAAACTA AACCTTTTGT   
  
  
- TAACTAAATT TCTCCTTGAA ACAATGTTTA TATTTTCAAT AATCGTAAAA GCCAAGTTTT ACGTATTCTA   
  
  
- CTCAACACAA AAAAAACAGC ACATGTGTAG TTTTTGACAG TATGCCTGTT ATTCGATCAG CGAAACTGGG   
  
  
- TAAGTGGCAT ACAGGGTTAC AGGAACTGGG ACGGTTTACT ATTCTTGCGG TCGTGTTTCC AAAATATTCA   
  
  
- AAGAGTTTCT CTTTGTTGTG TTACCCTTTT GTGTGGTAAC GAACTGAATG TTTACCTCTA GCCAACCTAG   
  
  
- TTAGAGACCT TTGTCCAACT TCATCTTAGA TTCGGGCAGA CCCGAGTACA GAGATGTGTC GTCTGGACTA   
  
  
- GTTTTGTTTA CCCTCTTACT CTTAGTGTAT TGAGACTTTC TCAGATAGAA AGATGCTCCA CCTAAGACAA   
  
  
- ATGGAAACCA GAGACGTCTA TCATCTACAA GGAACCGGTA CCGACCTCGC TAAAATTCGC AGTTTCGACG   
  
  
- TAGTGATCGT CACTGAGGTC AAGAATTTCA AAGCCAAAAT CAGTGTTCGC AAGA

+     Myb

| Site Name | Organism | Position | Strand | Matrix score. | sequence | function |
| --- | --- | --- | --- | --- | --- | --- |
| Myb | Arabidopsis thaliana | 396 | - | 6 | TAACTG |  |
| Myb | Arabidopsis thaliana | 779 | + | 6 | CAACTG |  |
| Myb | Arabidopsis thaliana | 592 | + | 6 | TAACTG |  |
| Myb | Arabidopsis thaliana | 858 | + | 6 | TAACTG |  |
| Myb | Arabidopsis thaliana | 437 | + | 6 | TAACTG |  |
| Myb | Arabidopsis thaliana | 625 | + | 6 | TAACTG |  |

>PlantCARE\_8972   
+ GAACTGTTAA AGCCAATCAT TTAAAATAAA AAGTCAAACC GGTTTAAACC GGTTTTCAAT AAGTTAATAA   
  
  
+ CCGGACTCGG TGTTAAAGAG ATTAAATTTA ACGGTTTAAC TCAGTGAGAT CGGTTTCGCT TTCTCTCTGT   
  
  
+ CGTGTTTGAG AAGTTGAAAG AGAGGAGGAT GAGCGCTGAA GATTTCCAGA AGAAGGTTTC GATCAGAGAC   
  
  
+ TCCTCCGTCG CCGGAGAAAT GGAAATCGAA TGCGGCGGGT CTACTAGCTC CGCGGTTGGT TCGTCTCGAA   
  
  
+ CCTTGGTTTT GCTTCGGAGA CTGCTCGAGA TTCAGGAGCG TCGAGCACAG GCCTACGCCA AACTCAAAAG   
  
  
+ GTTCGATCTT TGGAGTGTTT ATGCTATTAG TTACTGTTTT TGATTCAGTT ACTTGGTGAT TGTTACTGAG   
  
  
+ GGATGTTGGA GTTAAGTAAC TGATAGTGTT GTTGAGGAAG CGATGTCTCA TAAAGTTTAG ATTTTTATCT   
  
  
+ GTTGATCTCT TGCTATGCTT TTGGAATTTG ATTAATCAGA GGGTTCTCAG AGTATGTGGA GACTAGTGGT   
  
  
+ GAAGCGCTTT ATCAGAAGCT CTGCAGCGAG ATAACTGCTG AGTTCAACGA GTGTTCCAAA CAAGTAACTG   
  
  
+ AGTTTTTTTT TCTTCTTTTG ATTGATTACC TTTATGATGT TGTTGAAGAT ACGGTGTTAA AGATTGCGTC   
  
  
+ TTTGTTTGAT GGCAATGCAA GGTACGCGAA ATGGAAACTC TGTTTCTGAA TCCTGACGTT GGAAGATCGG   
  
  
+ ATCTTGCTCA ACTGCTCAGT GACATTCAAA CTCAGGAGAA GCAGAAACTG CATCTGGTCT GATTCCTATA   
  
  
+ TACTCAATTG TTGAACATAA CTGTTAACTT GTTGGACAGA ACAAAAGTTT CTTACTTTAG TTAGTTTTTT   
  
  
+ TTTTTTTTTG CAGACGGTTA CAATACAGGT ACTGAAGAAG GCAGGGAGGC CGTCAGAACG AATGCTGACA   
  
  
+ CACGAGAACT GCAAGTTCAA GAAACCGATG CAGCACGAGT GTGTGCATCT TCATGAGATT ACAGAAGCTG   
  
  
+ AAGGAACAGA GGAAGCAGAG GCGGATGCAG AGTTTGACAA CGCTTTGAAG GAAGCAATCA GAGGAGTGCA   
  
  
+ AGACGCTGTG ACTTGCATCA ATGAGTATTT GGAAGACGTT AGGTACGAGA TTGCAGCTCT TGAAGCTGAT   
  
  
+ TAGTTGTTCT CAATCTCATC ACTTCTTTAC CCCTTTCGAC TGTTAACTTG CTATGTTGCT AATTAGCTTA   
  
  
+ AGATATAAGA TATTTAGAAA CTCAAAAGTT ATGACATTTC TTGTTGTCAA AGTTATCTAT GTTGATTACT   
  
  
+ GTGGGTAGTG GCTACAAATA CAATAGCAAA TTCCAAATTA TTTAAAACAC AATATTTGAT TTGGAAAACA   
  
  
+ ATTGATTTAA AGAGGAACTT TGTTACAAAT ATAAAAGTTA TTAGCATTTT CGGTTCAAAA TGCATAAGAT   
  
  
+ GAGTTGTGTT TTTTTTGTCG TGTACACATC AAAAACTGTC ATACGGACAA TAAGCTAGTC GCTTTGACCC   
  
  
+ ATTCACCGTA TGTCCCAATG TCCTTGACCC TGCCAAATGA TAAGAACGCC AGCACAAAGG TTTTATAAGT   
  
  
+ TTCTCAAAGA GAAACAACAC AATGGGAAAA CACACCATTG CTTGACTTAC AAATGGAGAT CGGTTGGATC   
  
  
+ AATCTCTGGA AACAGGTTGA AGTAGAATCT AAGCCCGTCT GGGCTCATGT CTCTACACAG CAGACCTGAT   
  
  
+ CAAAACAAAT GGGAGAATGA GAATCACATA ACTCTGAAAG AGTCTATCTT TCTACGAGGT GGATTCTGTT   
  
  
+ TACCTTTGGT CTCTGCAGAT AGTAGATGTT CCTTGGCCAT GGCTGGAGCG ATTTTAAGCG TCAAAGCTGC   
  
  
+ ATCACTAGCA GTGACTCCAG TTCTTAAAGT TTCGGTTTTA GTCACAAGCG TTCT  

- CTTGACAATT TCGGTTAGTA AATTTTATTT TTCAGTTTGG CCAAATTTGG CCAAAAGTTA TTCAATTATT   
  
  
- GGCCTGAGCC ACAATTTCTC TAATTTAAAT TGCCAAATTG AGTCACTCTA GCCAAAGCGA AAGAGAGACA   
  
  
- GCACAAACTC TTCAACTTTC TCTCCTCCTA CTCGCGACTT CTAAAGGTCT TCTTCCAAAG CTAGTCTCTG   
  
  
- AGGAGGCAGC GGCCTCTTTA CCTTTAGCTT ACGCCGCCCA GATGATCGAG GCGCCAACCA AGCAGAGCTT   
  
  
- GGAACCAAAA CGAAGCCTCT GACGAGCTCT AAGTCCTCGC AGCTCGTGTC CGGATGCGGT TTGAGTTTTC   
  
  
- CAAGCTAGAA ACCTCACAAA TACGATAATC AATGACAAAA ACTAAGTCAA TGAACCACTA ACAATGACTC   
  
  
- CCTACAACCT CAATTCATTG ACTATCACAA CAACTCCTTC GCTACAGAGT ATTTCAAATC TAAAAATAGA   
  
  
- CAACTAGAGA ACGATACGAA AACCTTAAAC TAATTAGTCT CCCAAGAGTC TCATACACCT CTGATCACCA   
  
  
- CTTCGCGAAA TAGTCTTCGA GACGTCGCTC TATTGACGAC TCAAGTTGCT CACAAGGTTT GTTCATTGAC   
  
  
- TCAAAAAAAA AGAAGAAAAC TAACTAATGG AAATACTACA ACAACTTCTA TGCCACAATT TCTAACGCAG   
  
  
- AAACAAACTA CCGTTACGTT CCATGCGCTT TACCTTTGAG ACAAAGACTT AGGACTGCAA CCTTCTAGCC   
  
  
- TAGAACGAGT TGACGAGTCA CTGTAAGTTT GAGTCCTCTT CGTCTTTGAC GTAGACCAGA CTAAGGATAT   
  
  
- ATGAGTTAAC AACTTGTATT GACAATTGAA CAACCTGTCT TGTTTTCAAA GAATGAAATC AATCAAAAAA   
  
  
- AAAAAAAAAC GTCTGCCAAT GTTATGTCCA TGACTTCTTC CGTCCCTCCG GCAGTCTTGC TTACGACTGT   
  
  
- GTGCTCTTGA CGTTCAAGTT CTTTGGCTAC GTCGTGCTCA CACACGTAGA AGTACTCTAA TGTCTTCGAC   
  
  
- TTCCTTGTCT CCTTCGTCTC CGCCTACGTC TCAAACTGTT GCGAAACTTC CTTCGTTAGT CTCCTCACGT   
  
  
- TCTGCGACAC TGAACGTAGT TACTCATAAA CCTTCTGCAA TCCATGCTCT AACGTCGAGA ACTTCGACTA   
  
  
- ATCAACAAGA GTTAGAGTAG TGAAGAAATG GGGAAAGCTG ACAATTGAAC GATACAACGA TTAATCGAAT   
  
  
- TCTATATTCT ATAAATCTTT GAGTTTTCAA TACTGTAAAG AACAACAGTT TCAATAGATA CAACTAATGA   
  
  
- CACCCATCAC CGATGTTTAT GTTATCGTTT AAGGTTTAAT AAATTTTGTG TTATAAACTA AACCTTTTGT   
  
  
- TAACTAAATT TCTCCTTGAA ACAATGTTTA TATTTTCAAT AATCGTAAAA GCCAAGTTTT ACGTATTCTA   
  
  
- CTCAACACAA AAAAAACAGC ACATGTGTAG TTTTTGACAG TATGCCTGTT ATTCGATCAG CGAAACTGGG   
  
  
- TAAGTGGCAT ACAGGGTTAC AGGAACTGGG ACGGTTTACT ATTCTTGCGG TCGTGTTTCC AAAATATTCA   
  
  
- AAGAGTTTCT CTTTGTTGTG TTACCCTTTT GTGTGGTAAC GAACTGAATG TTTACCTCTA GCCAACCTAG   
  
  
- TTAGAGACCT TTGTCCAACT TCATCTTAGA TTCGGGCAGA CCCGAGTACA GAGATGTGTC GTCTGGACTA   
  
  
- GTTTTGTTTA CCCTCTTACT CTTAGTGTAT TGAGACTTTC TCAGATAGAA AGATGCTCCA CCTAAGACAA   
  
  
- ATGGAAACCA GAGACGTCTA TCATCTACAA GGAACCGGTA CCGACCTCGC TAAAATTCGC AGTTTCGACG   
  
  
- TAGTGATCGT CACTGAGGTC AAGAATTTCA AAGCCAAAAT CAGTGTTCGC AAGA

+     Myb-binding site

| Site Name | Organism | Position | Strand | Matrix score. | sequence | function |
| --- | --- | --- | --- | --- | --- | --- |
| Myb-binding site | Nicotiana tabacum | 489 | - | 6 | CAACAG |  |

>PlantCARE\_8972   
+ GAACTGTTAA AGCCAATCAT TTAAAATAAA AAGTCAAACC GGTTTAAACC GGTTTTCAAT AAGTTAATAA   
  
  
+ CCGGACTCGG TGTTAAAGAG ATTAAATTTA ACGGTTTAAC TCAGTGAGAT CGGTTTCGCT TTCTCTCTGT   
  
  
+ CGTGTTTGAG AAGTTGAAAG AGAGGAGGAT GAGCGCTGAA GATTTCCAGA AGAAGGTTTC GATCAGAGAC   
  
  
+ TCCTCCGTCG CCGGAGAAAT GGAAATCGAA TGCGGCGGGT CTACTAGCTC CGCGGTTGGT TCGTCTCGAA   
  
  
+ CCTTGGTTTT GCTTCGGAGA CTGCTCGAGA TTCAGGAGCG TCGAGCACAG GCCTACGCCA AACTCAAAAG   
  
  
+ GTTCGATCTT TGGAGTGTTT ATGCTATTAG TTACTGTTTT TGATTCAGTT ACTTGGTGAT TGTTACTGAG   
  
  
+ GGATGTTGGA GTTAAGTAAC TGATAGTGTT GTTGAGGAAG CGATGTCTCA TAAAGTTTAG ATTTTTATCT   
  
  
+ GTTGATCTCT TGCTATGCTT TTGGAATTTG ATTAATCAGA GGGTTCTCAG AGTATGTGGA GACTAGTGGT   
  
  
+ GAAGCGCTTT ATCAGAAGCT CTGCAGCGAG ATAACTGCTG AGTTCAACGA GTGTTCCAAA CAAGTAACTG   
  
  
+ AGTTTTTTTT TCTTCTTTTG ATTGATTACC TTTATGATGT TGTTGAAGAT ACGGTGTTAA AGATTGCGTC   
  
  
+ TTTGTTTGAT GGCAATGCAA GGTACGCGAA ATGGAAACTC TGTTTCTGAA TCCTGACGTT GGAAGATCGG   
  
  
+ ATCTTGCTCA ACTGCTCAGT GACATTCAAA CTCAGGAGAA GCAGAAACTG CATCTGGTCT GATTCCTATA   
  
  
+ TACTCAATTG TTGAACATAA CTGTTAACTT GTTGGACAGA ACAAAAGTTT CTTACTTTAG TTAGTTTTTT   
  
  
+ TTTTTTTTTG CAGACGGTTA CAATACAGGT ACTGAAGAAG GCAGGGAGGC CGTCAGAACG AATGCTGACA   
  
  
+ CACGAGAACT GCAAGTTCAA GAAACCGATG CAGCACGAGT GTGTGCATCT TCATGAGATT ACAGAAGCTG   
  
  
+ AAGGAACAGA GGAAGCAGAG GCGGATGCAG AGTTTGACAA CGCTTTGAAG GAAGCAATCA GAGGAGTGCA   
  
  
+ AGACGCTGTG ACTTGCATCA ATGAGTATTT GGAAGACGTT AGGTACGAGA TTGCAGCTCT TGAAGCTGAT   
  
  
+ TAGTTGTTCT CAATCTCATC ACTTCTTTAC CCCTTTCGAC TGTTAACTTG CTATGTTGCT AATTAGCTTA   
  
  
+ AGATATAAGA TATTTAGAAA CTCAAAAGTT ATGACATTTC TTGTTGTCAA AGTTATCTAT GTTGATTACT   
  
  
+ GTGGGTAGTG GCTACAAATA CAATAGCAAA TTCCAAATTA TTTAAAACAC AATATTTGAT TTGGAAAACA   
  
  
+ ATTGATTTAA AGAGGAACTT TGTTACAAAT ATAAAAGTTA TTAGCATTTT CGGTTCAAAA TGCATAAGAT   
  
  
+ GAGTTGTGTT TTTTTTGTCG TGTACACATC AAAAACTGTC ATACGGACAA TAAGCTAGTC GCTTTGACCC   
  
  
+ ATTCACCGTA TGTCCCAATG TCCTTGACCC TGCCAAATGA TAAGAACGCC AGCACAAAGG TTTTATAAGT   
  
  
+ TTCTCAAAGA GAAACAACAC AATGGGAAAA CACACCATTG CTTGACTTAC AAATGGAGAT CGGTTGGATC   
  
  
+ AATCTCTGGA AACAGGTTGA AGTAGAATCT AAGCCCGTCT GGGCTCATGT CTCTACACAG CAGACCTGAT   
  
  
+ CAAAACAAAT GGGAGAATGA GAATCACATA ACTCTGAAAG AGTCTATCTT TCTACGAGGT GGATTCTGTT   
  
  
+ TACCTTTGGT CTCTGCAGAT AGTAGATGTT CCTTGGCCAT GGCTGGAGCG ATTTTAAGCG TCAAAGCTGC   
  
  
+ ATCACTAGCA GTGACTCCAG TTCTTAAAGT TTCGGTTTTA GTCACAAGCG TTCT  

- CTTGACAATT TCGGTTAGTA AATTTTATTT TTCAGTTTGG CCAAATTTGG CCAAAAGTTA TTCAATTATT   
  
  
- GGCCTGAGCC ACAATTTCTC TAATTTAAAT TGCCAAATTG AGTCACTCTA GCCAAAGCGA AAGAGAGACA   
  
  
- GCACAAACTC TTCAACTTTC TCTCCTCCTA CTCGCGACTT CTAAAGGTCT TCTTCCAAAG CTAGTCTCTG   
  
  
- AGGAGGCAGC GGCCTCTTTA CCTTTAGCTT ACGCCGCCCA GATGATCGAG GCGCCAACCA AGCAGAGCTT   
  
  
- GGAACCAAAA CGAAGCCTCT GACGAGCTCT AAGTCCTCGC AGCTCGTGTC CGGATGCGGT TTGAGTTTTC   
  
  
- CAAGCTAGAA ACCTCACAAA TACGATAATC AATGACAAAA ACTAAGTCAA TGAACCACTA ACAATGACTC   
  
  
- CCTACAACCT CAATTCATTG ACTATCACAA CAACTCCTTC GCTACAGAGT ATTTCAAATC TAAAAATAGA   
  
  
- CAACTAGAGA ACGATACGAA AACCTTAAAC TAATTAGTCT CCCAAGAGTC TCATACACCT CTGATCACCA   
  
  
- CTTCGCGAAA TAGTCTTCGA GACGTCGCTC TATTGACGAC TCAAGTTGCT CACAAGGTTT GTTCATTGAC   
  
  
- TCAAAAAAAA AGAAGAAAAC TAACTAATGG AAATACTACA ACAACTTCTA TGCCACAATT TCTAACGCAG   
  
  
- AAACAAACTA CCGTTACGTT CCATGCGCTT TACCTTTGAG ACAAAGACTT AGGACTGCAA CCTTCTAGCC   
  
  
- TAGAACGAGT TGACGAGTCA CTGTAAGTTT GAGTCCTCTT CGTCTTTGAC GTAGACCAGA CTAAGGATAT   
  
  
- ATGAGTTAAC AACTTGTATT GACAATTGAA CAACCTGTCT TGTTTTCAAA GAATGAAATC AATCAAAAAA   
  
  
- AAAAAAAAAC GTCTGCCAAT GTTATGTCCA TGACTTCTTC CGTCCCTCCG GCAGTCTTGC TTACGACTGT   
  
  
- GTGCTCTTGA CGTTCAAGTT CTTTGGCTAC GTCGTGCTCA CACACGTAGA AGTACTCTAA TGTCTTCGAC   
  
  
- TTCCTTGTCT CCTTCGTCTC CGCCTACGTC TCAAACTGTT GCGAAACTTC CTTCGTTAGT CTCCTCACGT   
  
  
- TCTGCGACAC TGAACGTAGT TACTCATAAA CCTTCTGCAA TCCATGCTCT AACGTCGAGA ACTTCGACTA   
  
  
- ATCAACAAGA GTTAGAGTAG TGAAGAAATG GGGAAAGCTG ACAATTGAAC GATACAACGA TTAATCGAAT   
  
  
- TCTATATTCT ATAAATCTTT GAGTTTTCAA TACTGTAAAG AACAACAGTT TCAATAGATA CAACTAATGA   
  
  
- CACCCATCAC CGATGTTTAT GTTATCGTTT AAGGTTTAAT AAATTTTGTG TTATAAACTA AACCTTTTGT   
  
  
- TAACTAAATT TCTCCTTGAA ACAATGTTTA TATTTTCAAT AATCGTAAAA GCCAAGTTTT ACGTATTCTA   
  
  
- CTCAACACAA AAAAAACAGC ACATGTGTAG TTTTTGACAG TATGCCTGTT ATTCGATCAG CGAAACTGGG   
  
  
- TAAGTGGCAT ACAGGGTTAC AGGAACTGGG ACGGTTTACT ATTCTTGCGG TCGTGTTTCC AAAATATTCA   
  
  
- AAGAGTTTCT CTTTGTTGTG TTACCCTTTT GTGTGGTAAC GAACTGAATG TTTACCTCTA GCCAACCTAG   
  
  
- TTAGAGACCT TTGTCCAACT TCATCTTAGA TTCGGGCAGA CCCGAGTACA GAGATGTGTC GTCTGGACTA   
  
  
- GTTTTGTTTA CCCTCTTACT CTTAGTGTAT TGAGACTTTC TCAGATAGAA AGATGCTCCA CCTAAGACAA   
  
  
- ATGGAAACCA GAGACGTCTA TCATCTACAA GGAACCGGTA CCGACCTCGC TAAAATTCGC AGTTTCGACG   
  
  
- TAGTGATCGT CACTGAGGTC AAGAATTTCA AAGCCAAAAT CAGTGTTCGC AAGA

+     P-box

| Site Name | Organism | Position | Strand | Matrix score. | sequence | function |
| --- | --- | --- | --- | --- | --- | --- |
| P-box | Oryza sativa | 345 | - | 7 | CCTTTTG | gibberellin-responsive element |

>PlantCARE\_8972   
+ GAACTGTTAA AGCCAATCAT TTAAAATAAA AAGTCAAACC GGTTTAAACC GGTTTTCAAT AAGTTAATAA   
  
  
+ CCGGACTCGG TGTTAAAGAG ATTAAATTTA ACGGTTTAAC TCAGTGAGAT CGGTTTCGCT TTCTCTCTGT   
  
  
+ CGTGTTTGAG AAGTTGAAAG AGAGGAGGAT GAGCGCTGAA GATTTCCAGA AGAAGGTTTC GATCAGAGAC   
  
  
+ TCCTCCGTCG CCGGAGAAAT GGAAATCGAA TGCGGCGGGT CTACTAGCTC CGCGGTTGGT TCGTCTCGAA   
  
  
+ CCTTGGTTTT GCTTCGGAGA CTGCTCGAGA TTCAGGAGCG TCGAGCACAG GCCTACGCCA AACTCAAAAG   
  
  
+ GTTCGATCTT TGGAGTGTTT ATGCTATTAG TTACTGTTTT TGATTCAGTT ACTTGGTGAT TGTTACTGAG   
  
  
+ GGATGTTGGA GTTAAGTAAC TGATAGTGTT GTTGAGGAAG CGATGTCTCA TAAAGTTTAG ATTTTTATCT   
  
  
+ GTTGATCTCT TGCTATGCTT TTGGAATTTG ATTAATCAGA GGGTTCTCAG AGTATGTGGA GACTAGTGGT   
  
  
+ GAAGCGCTTT ATCAGAAGCT CTGCAGCGAG ATAACTGCTG AGTTCAACGA GTGTTCCAAA CAAGTAACTG   
  
  
+ AGTTTTTTTT TCTTCTTTTG ATTGATTACC TTTATGATGT TGTTGAAGAT ACGGTGTTAA AGATTGCGTC   
  
  
+ TTTGTTTGAT GGCAATGCAA GGTACGCGAA ATGGAAACTC TGTTTCTGAA TCCTGACGTT GGAAGATCGG   
  
  
+ ATCTTGCTCA ACTGCTCAGT GACATTCAAA CTCAGGAGAA GCAGAAACTG CATCTGGTCT GATTCCTATA   
  
  
+ TACTCAATTG TTGAACATAA CTGTTAACTT GTTGGACAGA ACAAAAGTTT CTTACTTTAG TTAGTTTTTT   
  
  
+ TTTTTTTTTG CAGACGGTTA CAATACAGGT ACTGAAGAAG GCAGGGAGGC CGTCAGAACG AATGCTGACA   
  
  
+ CACGAGAACT GCAAGTTCAA GAAACCGATG CAGCACGAGT GTGTGCATCT TCATGAGATT ACAGAAGCTG   
  
  
+ AAGGAACAGA GGAAGCAGAG GCGGATGCAG AGTTTGACAA CGCTTTGAAG GAAGCAATCA GAGGAGTGCA   
  
  
+ AGACGCTGTG ACTTGCATCA ATGAGTATTT GGAAGACGTT AGGTACGAGA TTGCAGCTCT TGAAGCTGAT   
  
  
+ TAGTTGTTCT CAATCTCATC ACTTCTTTAC CCCTTTCGAC TGTTAACTTG CTATGTTGCT AATTAGCTTA   
  
  
+ AGATATAAGA TATTTAGAAA CTCAAAAGTT ATGACATTTC TTGTTGTCAA AGTTATCTAT GTTGATTACT   
  
  
+ GTGGGTAGTG GCTACAAATA CAATAGCAAA TTCCAAATTA TTTAAAACAC AATATTTGAT TTGGAAAACA   
  
  
+ ATTGATTTAA AGAGGAACTT TGTTACAAAT ATAAAAGTTA TTAGCATTTT CGGTTCAAAA TGCATAAGAT   
  
  
+ GAGTTGTGTT TTTTTTGTCG TGTACACATC AAAAACTGTC ATACGGACAA TAAGCTAGTC GCTTTGACCC   
  
  
+ ATTCACCGTA TGTCCCAATG TCCTTGACCC TGCCAAATGA TAAGAACGCC AGCACAAAGG TTTTATAAGT   
  
  
+ TTCTCAAAGA GAAACAACAC AATGGGAAAA CACACCATTG CTTGACTTAC AAATGGAGAT CGGTTGGATC   
  
  
+ AATCTCTGGA AACAGGTTGA AGTAGAATCT AAGCCCGTCT GGGCTCATGT CTCTACACAG CAGACCTGAT   
  
  
+ CAAAACAAAT GGGAGAATGA GAATCACATA ACTCTGAAAG AGTCTATCTT TCTACGAGGT GGATTCTGTT   
  
  
+ TACCTTTGGT CTCTGCAGAT AGTAGATGTT CCTTGGCCAT GGCTGGAGCG ATTTTAAGCG TCAAAGCTGC   
  
  
+ ATCACTAGCA GTGACTCCAG TTCTTAAAGT TTCGGTTTTA GTCACAAGCG TTCT  

- CTTGACAATT TCGGTTAGTA AATTTTATTT TTCAGTTTGG CCAAATTTGG CCAAAAGTTA TTCAATTATT   
  
  
- GGCCTGAGCC ACAATTTCTC TAATTTAAAT TGCCAAATTG AGTCACTCTA GCCAAAGCGA AAGAGAGACA   
  
  
- GCACAAACTC TTCAACTTTC TCTCCTCCTA CTCGCGACTT CTAAAGGTCT TCTTCCAAAG CTAGTCTCTG   
  
  
- AGGAGGCAGC GGCCTCTTTA CCTTTAGCTT ACGCCGCCCA GATGATCGAG GCGCCAACCA AGCAGAGCTT   
  
  
- GGAACCAAAA CGAAGCCTCT GACGAGCTCT AAGTCCTCGC AGCTCGTGTC CGGATGCGGT TTGAGTTTTC   
  
  
- CAAGCTAGAA ACCTCACAAA TACGATAATC AATGACAAAA ACTAAGTCAA TGAACCACTA ACAATGACTC   
  
  
- CCTACAACCT CAATTCATTG ACTATCACAA CAACTCCTTC GCTACAGAGT ATTTCAAATC TAAAAATAGA   
  
  
- CAACTAGAGA ACGATACGAA AACCTTAAAC TAATTAGTCT CCCAAGAGTC TCATACACCT CTGATCACCA   
  
  
- CTTCGCGAAA TAGTCTTCGA GACGTCGCTC TATTGACGAC TCAAGTTGCT CACAAGGTTT GTTCATTGAC   
  
  
- TCAAAAAAAA AGAAGAAAAC TAACTAATGG AAATACTACA ACAACTTCTA TGCCACAATT TCTAACGCAG   
  
  
- AAACAAACTA CCGTTACGTT CCATGCGCTT TACCTTTGAG ACAAAGACTT AGGACTGCAA CCTTCTAGCC   
  
  
- TAGAACGAGT TGACGAGTCA CTGTAAGTTT GAGTCCTCTT CGTCTTTGAC GTAGACCAGA CTAAGGATAT   
  
  
- ATGAGTTAAC AACTTGTATT GACAATTGAA CAACCTGTCT TGTTTTCAAA GAATGAAATC AATCAAAAAA   
  
  
- AAAAAAAAAC GTCTGCCAAT GTTATGTCCA TGACTTCTTC CGTCCCTCCG GCAGTCTTGC TTACGACTGT   
  
  
- GTGCTCTTGA CGTTCAAGTT CTTTGGCTAC GTCGTGCTCA CACACGTAGA AGTACTCTAA TGTCTTCGAC   
  
  
- TTCCTTGTCT CCTTCGTCTC CGCCTACGTC TCAAACTGTT GCGAAACTTC CTTCGTTAGT CTCCTCACGT   
  
  
- TCTGCGACAC TGAACGTAGT TACTCATAAA CCTTCTGCAA TCCATGCTCT AACGTCGAGA ACTTCGACTA   
  
  
- ATCAACAAGA GTTAGAGTAG TGAAGAAATG GGGAAAGCTG ACAATTGAAC GATACAACGA TTAATCGAAT   
  
  
- TCTATATTCT ATAAATCTTT GAGTTTTCAA TACTGTAAAG AACAACAGTT TCAATAGATA CAACTAATGA   
  
  
- CACCCATCAC CGATGTTTAT GTTATCGTTT AAGGTTTAAT AAATTTTGTG TTATAAACTA AACCTTTTGT   
  
  
- TAACTAAATT TCTCCTTGAA ACAATGTTTA TATTTTCAAT AATCGTAAAA GCCAAGTTTT ACGTATTCTA   
  
  
- CTCAACACAA AAAAAACAGC ACATGTGTAG TTTTTGACAG TATGCCTGTT ATTCGATCAG CGAAACTGGG   
  
  
- TAAGTGGCAT ACAGGGTTAC AGGAACTGGG ACGGTTTACT ATTCTTGCGG TCGTGTTTCC AAAATATTCA   
  
  
- AAGAGTTTCT CTTTGTTGTG TTACCCTTTT GTGTGGTAAC GAACTGAATG TTTACCTCTA GCCAACCTAG   
  
  
- TTAGAGACCT TTGTCCAACT TCATCTTAGA TTCGGGCAGA CCCGAGTACA GAGATGTGTC GTCTGGACTA   
  
  
- GTTTTGTTTA CCCTCTTACT CTTAGTGTAT TGAGACTTTC TCAGATAGAA AGATGCTCCA CCTAAGACAA   
  
  
- ATGGAAACCA GAGACGTCTA TCATCTACAA GGAACCGGTA CCGACCTCGC TAAAATTCGC AGTTTCGACG   
  
  
- TAGTGATCGT CACTGAGGTC AAGAATTTCA AAGCCAAAAT CAGTGTTCGC AAGA

+     STRE

| Site Name | Organism | Position | Strand | Matrix score. | sequence | function |
| --- | --- | --- | --- | --- | --- | --- |
| STRE | Arabidopsis thaliana | 1220 | - | 5 | AGGGG |  |

>PlantCARE\_8972   
+ GAACTGTTAA AGCCAATCAT TTAAAATAAA AAGTCAAACC GGTTTAAACC GGTTTTCAAT AAGTTAATAA   
  
  
+ CCGGACTCGG TGTTAAAGAG ATTAAATTTA ACGGTTTAAC TCAGTGAGAT CGGTTTCGCT TTCTCTCTGT   
  
  
+ CGTGTTTGAG AAGTTGAAAG AGAGGAGGAT GAGCGCTGAA GATTTCCAGA AGAAGGTTTC GATCAGAGAC   
  
  
+ TCCTCCGTCG CCGGAGAAAT GGAAATCGAA TGCGGCGGGT CTACTAGCTC CGCGGTTGGT TCGTCTCGAA   
  
  
+ CCTTGGTTTT GCTTCGGAGA CTGCTCGAGA TTCAGGAGCG TCGAGCACAG GCCTACGCCA AACTCAAAAG   
  
  
+ GTTCGATCTT TGGAGTGTTT ATGCTATTAG TTACTGTTTT TGATTCAGTT ACTTGGTGAT TGTTACTGAG   
  
  
+ GGATGTTGGA GTTAAGTAAC TGATAGTGTT GTTGAGGAAG CGATGTCTCA TAAAGTTTAG ATTTTTATCT   
  
  
+ GTTGATCTCT TGCTATGCTT TTGGAATTTG ATTAATCAGA GGGTTCTCAG AGTATGTGGA GACTAGTGGT   
  
  
+ GAAGCGCTTT ATCAGAAGCT CTGCAGCGAG ATAACTGCTG AGTTCAACGA GTGTTCCAAA CAAGTAACTG   
  
  
+ AGTTTTTTTT TCTTCTTTTG ATTGATTACC TTTATGATGT TGTTGAAGAT ACGGTGTTAA AGATTGCGTC   
  
  
+ TTTGTTTGAT GGCAATGCAA GGTACGCGAA ATGGAAACTC TGTTTCTGAA TCCTGACGTT GGAAGATCGG   
  
  
+ ATCTTGCTCA ACTGCTCAGT GACATTCAAA CTCAGGAGAA GCAGAAACTG CATCTGGTCT GATTCCTATA   
  
  
+ TACTCAATTG TTGAACATAA CTGTTAACTT GTTGGACAGA ACAAAAGTTT CTTACTTTAG TTAGTTTTTT   
  
  
+ TTTTTTTTTG CAGACGGTTA CAATACAGGT ACTGAAGAAG GCAGGGAGGC CGTCAGAACG AATGCTGACA   
  
  
+ CACGAGAACT GCAAGTTCAA GAAACCGATG CAGCACGAGT GTGTGCATCT TCATGAGATT ACAGAAGCTG   
  
  
+ AAGGAACAGA GGAAGCAGAG GCGGATGCAG AGTTTGACAA CGCTTTGAAG GAAGCAATCA GAGGAGTGCA   
  
  
+ AGACGCTGTG ACTTGCATCA ATGAGTATTT GGAAGACGTT AGGTACGAGA TTGCAGCTCT TGAAGCTGAT   
  
  
+ TAGTTGTTCT CAATCTCATC ACTTCTTTAC CCCTTTCGAC TGTTAACTTG CTATGTTGCT AATTAGCTTA   
  
  
+ AGATATAAGA TATTTAGAAA CTCAAAAGTT ATGACATTTC TTGTTGTCAA AGTTATCTAT GTTGATTACT   
  
  
+ GTGGGTAGTG GCTACAAATA CAATAGCAAA TTCCAAATTA TTTAAAACAC AATATTTGAT TTGGAAAACA   
  
  
+ ATTGATTTAA AGAGGAACTT TGTTACAAAT ATAAAAGTTA TTAGCATTTT CGGTTCAAAA TGCATAAGAT   
  
  
+ GAGTTGTGTT TTTTTTGTCG TGTACACATC AAAAACTGTC ATACGGACAA TAAGCTAGTC GCTTTGACCC   
  
  
+ ATTCACCGTA TGTCCCAATG TCCTTGACCC TGCCAAATGA TAAGAACGCC AGCACAAAGG TTTTATAAGT   
  
  
+ TTCTCAAAGA GAAACAACAC AATGGGAAAA CACACCATTG CTTGACTTAC AAATGGAGAT CGGTTGGATC   
  
  
+ AATCTCTGGA AACAGGTTGA AGTAGAATCT AAGCCCGTCT GGGCTCATGT CTCTACACAG CAGACCTGAT   
  
  
+ CAAAACAAAT GGGAGAATGA GAATCACATA ACTCTGAAAG AGTCTATCTT TCTACGAGGT GGATTCTGTT   
  
  
+ TACCTTTGGT CTCTGCAGAT AGTAGATGTT CCTTGGCCAT GGCTGGAGCG ATTTTAAGCG TCAAAGCTGC   
  
  
+ ATCACTAGCA GTGACTCCAG TTCTTAAAGT TTCGGTTTTA GTCACAAGCG TTCT  

- CTTGACAATT TCGGTTAGTA AATTTTATTT TTCAGTTTGG CCAAATTTGG CCAAAAGTTA TTCAATTATT   
  
  
- GGCCTGAGCC ACAATTTCTC TAATTTAAAT TGCCAAATTG AGTCACTCTA GCCAAAGCGA AAGAGAGACA   
  
  
- GCACAAACTC TTCAACTTTC TCTCCTCCTA CTCGCGACTT CTAAAGGTCT TCTTCCAAAG CTAGTCTCTG   
  
  
- AGGAGGCAGC GGCCTCTTTA CCTTTAGCTT ACGCCGCCCA GATGATCGAG GCGCCAACCA AGCAGAGCTT   
  
  
- GGAACCAAAA CGAAGCCTCT GACGAGCTCT AAGTCCTCGC AGCTCGTGTC CGGATGCGGT TTGAGTTTTC   
  
  
- CAAGCTAGAA ACCTCACAAA TACGATAATC AATGACAAAA ACTAAGTCAA TGAACCACTA ACAATGACTC   
  
  
- CCTACAACCT CAATTCATTG ACTATCACAA CAACTCCTTC GCTACAGAGT ATTTCAAATC TAAAAATAGA   
  
  
- CAACTAGAGA ACGATACGAA AACCTTAAAC TAATTAGTCT CCCAAGAGTC TCATACACCT CTGATCACCA   
  
  
- CTTCGCGAAA TAGTCTTCGA GACGTCGCTC TATTGACGAC TCAAGTTGCT CACAAGGTTT GTTCATTGAC   
  
  
- TCAAAAAAAA AGAAGAAAAC TAACTAATGG AAATACTACA ACAACTTCTA TGCCACAATT TCTAACGCAG   
  
  
- AAACAAACTA CCGTTACGTT CCATGCGCTT TACCTTTGAG ACAAAGACTT AGGACTGCAA CCTTCTAGCC   
  
  
- TAGAACGAGT TGACGAGTCA CTGTAAGTTT GAGTCCTCTT CGTCTTTGAC GTAGACCAGA CTAAGGATAT   
  
  
- ATGAGTTAAC AACTTGTATT GACAATTGAA CAACCTGTCT TGTTTTCAAA GAATGAAATC AATCAAAAAA   
  
  
- AAAAAAAAAC GTCTGCCAAT GTTATGTCCA TGACTTCTTC CGTCCCTCCG GCAGTCTTGC TTACGACTGT   
  
  
- GTGCTCTTGA CGTTCAAGTT CTTTGGCTAC GTCGTGCTCA CACACGTAGA AGTACTCTAA TGTCTTCGAC   
  
  
- TTCCTTGTCT CCTTCGTCTC CGCCTACGTC TCAAACTGTT GCGAAACTTC CTTCGTTAGT CTCCTCACGT   
  
  
- TCTGCGACAC TGAACGTAGT TACTCATAAA CCTTCTGCAA TCCATGCTCT AACGTCGAGA ACTTCGACTA   
  
  
- ATCAACAAGA GTTAGAGTAG TGAAGAAATG GGGAAAGCTG ACAATTGAAC GATACAACGA TTAATCGAAT   
  
  
- TCTATATTCT ATAAATCTTT GAGTTTTCAA TACTGTAAAG AACAACAGTT TCAATAGATA CAACTAATGA   
  
  
- CACCCATCAC CGATGTTTAT GTTATCGTTT AAGGTTTAAT AAATTTTGTG TTATAAACTA AACCTTTTGT   
  
  
- TAACTAAATT TCTCCTTGAA ACAATGTTTA TATTTTCAAT AATCGTAAAA GCCAAGTTTT ACGTATTCTA   
  
  
- CTCAACACAA AAAAAACAGC ACATGTGTAG TTTTTGACAG TATGCCTGTT ATTCGATCAG CGAAACTGGG   
  
  
- TAAGTGGCAT ACAGGGTTAC AGGAACTGGG ACGGTTTACT ATTCTTGCGG TCGTGTTTCC AAAATATTCA   
  
  
- AAGAGTTTCT CTTTGTTGTG TTACCCTTTT GTGTGGTAAC GAACTGAATG TTTACCTCTA GCCAACCTAG   
  
  
- TTAGAGACCT TTGTCCAACT TCATCTTAGA TTCGGGCAGA CCCGAGTACA GAGATGTGTC GTCTGGACTA   
  
  
- GTTTTGTTTA CCCTCTTACT CTTAGTGTAT TGAGACTTTC TCAGATAGAA AGATGCTCCA CCTAAGACAA   
  
  
- ATGGAAACCA GAGACGTCTA TCATCTACAA GGAACCGGTA CCGACCTCGC TAAAATTCGC AGTTTCGACG   
  
  
- TAGTGATCGT CACTGAGGTC AAGAATTTCA AAGCCAAAAT CAGTGTTCGC AAGA

+     TATA-box

| Site Name | Organism | Position | Strand | Matrix score. | sequence | function |
| --- | --- | --- | --- | --- | --- | --- |
| TATA-box | Arabidopsis thaliana | 688 | + | 8 | TAAAGATT | core promoter element around -30 of transcription start |
| TATA-box | Brassica oleracea | 1429 | + | 6 | ATATAA | core promoter element around -30 of transcription start |
| TATA-box | Arabidopsis thaliana | 1264 | - | 4 | TATA | core promoter element around -30 of transcription start |
| TATA-box | Arabidopsis thaliana | 1603 | - | 5 | TATAA | core promoter element around -30 of transcription start |
| TATA-box | Arabidopsis thaliana | 839 | + | 4 | TATA | core promoter element around -30 of transcription start |
| TATA-box | Pisum sativum | 1601 | - | 7 | TATAAAA | core promoter element around -30 of transcription start |
| TATA-box | Arabidopsis thaliana | 1369 | + | 8 | TATTTAAA | core promoter element around -30 of transcription start |
| TATA-box | Arabidopsis thaliana | 1604 | - | 4 | TATA | core promoter element around -30 of transcription start |
| TATA-box | Arabidopsis thaliana | 837 | + | 6 | TATATA | core promoter element around -30 of transcription start |
| TATA-box | Arabidopsis thaliana | 1430 | - | 4 | TATA | core promoter element around -30 of transcription start |
| TATA-box | Brassica oleracea | 1263 | + | 6 | ATATAA | core promoter element around -30 of transcription start |
| TATA-box | Helianthus annuus | 1602 | - | 6 | TATAAA | core promoter element around -30 of transcription start |

>PlantCARE\_8972   
+ GAACTGTTAA AGCCAATCAT TTAAAATAAA AAGTCAAACC GGTTTAAACC GGTTTTCAAT AAGTTAATAA   
  
  
+ CCGGACTCGG TGTTAAAGAG ATTAAATTTA ACGGTTTAAC TCAGTGAGAT CGGTTTCGCT TTCTCTCTGT   
  
  
+ CGTGTTTGAG AAGTTGAAAG AGAGGAGGAT GAGCGCTGAA GATTTCCAGA AGAAGGTTTC GATCAGAGAC   
  
  
+ TCCTCCGTCG CCGGAGAAAT GGAAATCGAA TGCGGCGGGT CTACTAGCTC CGCGGTTGGT TCGTCTCGAA   
  
  
+ CCTTGGTTTT GCTTCGGAGA CTGCTCGAGA TTCAGGAGCG TCGAGCACAG GCCTACGCCA AACTCAAAAG   
  
  
+ GTTCGATCTT TGGAGTGTTT ATGCTATTAG TTACTGTTTT TGATTCAGTT ACTTGGTGAT TGTTACTGAG   
  
  
+ GGATGTTGGA GTTAAGTAAC TGATAGTGTT GTTGAGGAAG CGATGTCTCA TAAAGTTTAG ATTTTTATCT   
  
  
+ GTTGATCTCT TGCTATGCTT TTGGAATTTG ATTAATCAGA GGGTTCTCAG AGTATGTGGA GACTAGTGGT   
  
  
+ GAAGCGCTTT ATCAGAAGCT CTGCAGCGAG ATAACTGCTG AGTTCAACGA GTGTTCCAAA CAAGTAACTG   
  
  
+ AGTTTTTTTT TCTTCTTTTG ATTGATTACC TTTATGATGT TGTTGAAGAT ACGGTGTTAA AGATTGCGTC   
  
  
+ TTTGTTTGAT GGCAATGCAA GGTACGCGAA ATGGAAACTC TGTTTCTGAA TCCTGACGTT GGAAGATCGG   
  
  
+ ATCTTGCTCA ACTGCTCAGT GACATTCAAA CTCAGGAGAA GCAGAAACTG CATCTGGTCT GATTCCTATA   
  
  
+ TACTCAATTG TTGAACATAA CTGTTAACTT GTTGGACAGA ACAAAAGTTT CTTACTTTAG TTAGTTTTTT   
  
  
+ TTTTTTTTTG CAGACGGTTA CAATACAGGT ACTGAAGAAG GCAGGGAGGC CGTCAGAACG AATGCTGACA   
  
  
+ CACGAGAACT GCAAGTTCAA GAAACCGATG CAGCACGAGT GTGTGCATCT TCATGAGATT ACAGAAGCTG   
  
  
+ AAGGAACAGA GGAAGCAGAG GCGGATGCAG AGTTTGACAA CGCTTTGAAG GAAGCAATCA GAGGAGTGCA   
  
  
+ AGACGCTGTG ACTTGCATCA ATGAGTATTT GGAAGACGTT AGGTACGAGA TTGCAGCTCT TGAAGCTGAT   
  
  
+ TAGTTGTTCT CAATCTCATC ACTTCTTTAC CCCTTTCGAC TGTTAACTTG CTATGTTGCT AATTAGCTTA   
  
  
+ AGATATAAGA TATTTAGAAA CTCAAAAGTT ATGACATTTC TTGTTGTCAA AGTTATCTAT GTTGATTACT   
  
  
+ GTGGGTAGTG GCTACAAATA CAATAGCAAA TTCCAAATTA TTTAAAACAC AATATTTGAT TTGGAAAACA   
  
  
+ ATTGATTTAA AGAGGAACTT TGTTACAAAT ATAAAAGTTA TTAGCATTTT CGGTTCAAAA TGCATAAGAT   
  
  
+ GAGTTGTGTT TTTTTTGTCG TGTACACATC AAAAACTGTC ATACGGACAA TAAGCTAGTC GCTTTGACCC   
  
  
+ ATTCACCGTA TGTCCCAATG TCCTTGACCC TGCCAAATGA TAAGAACGCC AGCACAAAGG TTTTATAAGT   
  
  
+ TTCTCAAAGA GAAACAACAC AATGGGAAAA CACACCATTG CTTGACTTAC AAATGGAGAT CGGTTGGATC   
  
  
+ AATCTCTGGA AACAGGTTGA AGTAGAATCT AAGCCCGTCT GGGCTCATGT CTCTACACAG CAGACCTGAT   
  
  
+ CAAAACAAAT GGGAGAATGA GAATCACATA ACTCTGAAAG AGTCTATCTT TCTACGAGGT GGATTCTGTT   
  
  
+ TACCTTTGGT CTCTGCAGAT AGTAGATGTT CCTTGGCCAT GGCTGGAGCG ATTTTAAGCG TCAAAGCTGC   
  
  
+ ATCACTAGCA GTGACTCCAG TTCTTAAAGT TTCGGTTTTA GTCACAAGCG TTCT  

- CTTGACAATT TCGGTTAGTA AATTTTATTT TTCAGTTTGG CCAAATTTGG CCAAAAGTTA TTCAATTATT   
  
  
- GGCCTGAGCC ACAATTTCTC TAATTTAAAT TGCCAAATTG AGTCACTCTA GCCAAAGCGA AAGAGAGACA   
  
  
- GCACAAACTC TTCAACTTTC TCTCCTCCTA CTCGCGACTT CTAAAGGTCT TCTTCCAAAG CTAGTCTCTG   
  
  
- AGGAGGCAGC GGCCTCTTTA CCTTTAGCTT ACGCCGCCCA GATGATCGAG GCGCCAACCA AGCAGAGCTT   
  
  
- GGAACCAAAA CGAAGCCTCT GACGAGCTCT AAGTCCTCGC AGCTCGTGTC CGGATGCGGT TTGAGTTTTC   
  
  
- CAAGCTAGAA ACCTCACAAA TACGATAATC AATGACAAAA ACTAAGTCAA TGAACCACTA ACAATGACTC   
  
  
- CCTACAACCT CAATTCATTG ACTATCACAA CAACTCCTTC GCTACAGAGT ATTTCAAATC TAAAAATAGA   
  
  
- CAACTAGAGA ACGATACGAA AACCTTAAAC TAATTAGTCT CCCAAGAGTC TCATACACCT CTGATCACCA   
  
  
- CTTCGCGAAA TAGTCTTCGA GACGTCGCTC TATTGACGAC TCAAGTTGCT CACAAGGTTT GTTCATTGAC   
  
  
- TCAAAAAAAA AGAAGAAAAC TAACTAATGG AAATACTACA ACAACTTCTA TGCCACAATT TCTAACGCAG   
  
  
- AAACAAACTA CCGTTACGTT CCATGCGCTT TACCTTTGAG ACAAAGACTT AGGACTGCAA CCTTCTAGCC   
  
  
- TAGAACGAGT TGACGAGTCA CTGTAAGTTT GAGTCCTCTT CGTCTTTGAC GTAGACCAGA CTAAGGATAT   
  
  
- ATGAGTTAAC AACTTGTATT GACAATTGAA CAACCTGTCT TGTTTTCAAA GAATGAAATC AATCAAAAAA   
  
  
- AAAAAAAAAC GTCTGCCAAT GTTATGTCCA TGACTTCTTC CGTCCCTCCG GCAGTCTTGC TTACGACTGT   
  
  
- GTGCTCTTGA CGTTCAAGTT CTTTGGCTAC GTCGTGCTCA CACACGTAGA AGTACTCTAA TGTCTTCGAC   
  
  
- TTCCTTGTCT CCTTCGTCTC CGCCTACGTC TCAAACTGTT GCGAAACTTC CTTCGTTAGT CTCCTCACGT   
  
  
- TCTGCGACAC TGAACGTAGT TACTCATAAA CCTTCTGCAA TCCATGCTCT AACGTCGAGA ACTTCGACTA   
  
  
- ATCAACAAGA GTTAGAGTAG TGAAGAAATG GGGAAAGCTG ACAATTGAAC GATACAACGA TTAATCGAAT   
  
  
- TCTATATTCT ATAAATCTTT GAGTTTTCAA TACTGTAAAG AACAACAGTT TCAATAGATA CAACTAATGA   
  
  
- CACCCATCAC CGATGTTTAT GTTATCGTTT AAGGTTTAAT AAATTTTGTG TTATAAACTA AACCTTTTGT   
  
  
- TAACTAAATT TCTCCTTGAA ACAATGTTTA TATTTTCAAT AATCGTAAAA GCCAAGTTTT ACGTATTCTA   
  
  
- CTCAACACAA AAAAAACAGC ACATGTGTAG TTTTTGACAG TATGCCTGTT ATTCGATCAG CGAAACTGGG   
  
  
- TAAGTGGCAT ACAGGGTTAC AGGAACTGGG ACGGTTTACT ATTCTTGCGG TCGTGTTTCC AAAATATTCA   
  
  
- AAGAGTTTCT CTTTGTTGTG TTACCCTTTT GTGTGGTAAC GAACTGAATG TTTACCTCTA GCCAACCTAG   
  
  
- TTAGAGACCT TTGTCCAACT TCATCTTAGA TTCGGGCAGA CCCGAGTACA GAGATGTGTC GTCTGGACTA   
  
  
- GTTTTGTTTA CCCTCTTACT CTTAGTGTAT TGAGACTTTC TCAGATAGAA AGATGCTCCA CCTAAGACAA   
  
  
- ATGGAAACCA GAGACGTCTA TCATCTACAA GGAACCGGTA CCGACCTCGC TAAAATTCGC AGTTTCGACG   
  
  
- TAGTGATCGT CACTGAGGTC AAGAATTTCA AAGCCAAAAT CAGTGTTCGC AAGA

+     TCA

| Site Name | Organism | Position | Strand | Matrix score. | sequence | function |
| --- | --- | --- | --- | --- | --- | --- |
| TCA | Pisum sativum | 1025 | + | 9 | TCATCTTCAT |  |

>PlantCARE\_8972   
+ GAACTGTTAA AGCCAATCAT TTAAAATAAA AAGTCAAACC GGTTTAAACC GGTTTTCAAT AAGTTAATAA   
  
  
+ CCGGACTCGG TGTTAAAGAG ATTAAATTTA ACGGTTTAAC TCAGTGAGAT CGGTTTCGCT TTCTCTCTGT   
  
  
+ CGTGTTTGAG AAGTTGAAAG AGAGGAGGAT GAGCGCTGAA GATTTCCAGA AGAAGGTTTC GATCAGAGAC   
  
  
+ TCCTCCGTCG CCGGAGAAAT GGAAATCGAA TGCGGCGGGT CTACTAGCTC CGCGGTTGGT TCGTCTCGAA   
  
  
+ CCTTGGTTTT GCTTCGGAGA CTGCTCGAGA TTCAGGAGCG TCGAGCACAG GCCTACGCCA AACTCAAAAG   
  
  
+ GTTCGATCTT TGGAGTGTTT ATGCTATTAG TTACTGTTTT TGATTCAGTT ACTTGGTGAT TGTTACTGAG   
  
  
+ GGATGTTGGA GTTAAGTAAC TGATAGTGTT GTTGAGGAAG CGATGTCTCA TAAAGTTTAG ATTTTTATCT   
  
  
+ GTTGATCTCT TGCTATGCTT TTGGAATTTG ATTAATCAGA GGGTTCTCAG AGTATGTGGA GACTAGTGGT   
  
  
+ GAAGCGCTTT ATCAGAAGCT CTGCAGCGAG ATAACTGCTG AGTTCAACGA GTGTTCCAAA CAAGTAACTG   
  
  
+ AGTTTTTTTT TCTTCTTTTG ATTGATTACC TTTATGATGT TGTTGAAGAT ACGGTGTTAA AGATTGCGTC   
  
  
+ TTTGTTTGAT GGCAATGCAA GGTACGCGAA ATGGAAACTC TGTTTCTGAA TCCTGACGTT GGAAGATCGG   
  
  
+ ATCTTGCTCA ACTGCTCAGT GACATTCAAA CTCAGGAGAA GCAGAAACTG CATCTGGTCT GATTCCTATA   
  
  
+ TACTCAATTG TTGAACATAA CTGTTAACTT GTTGGACAGA ACAAAAGTTT CTTACTTTAG TTAGTTTTTT   
  
  
+ TTTTTTTTTG CAGACGGTTA CAATACAGGT ACTGAAGAAG GCAGGGAGGC CGTCAGAACG AATGCTGACA   
  
  
+ CACGAGAACT GCAAGTTCAA GAAACCGATG CAGCACGAGT GTGTGCATCT TCATGAGATT ACAGAAGCTG   
  
  
+ AAGGAACAGA GGAAGCAGAG GCGGATGCAG AGTTTGACAA CGCTTTGAAG GAAGCAATCA GAGGAGTGCA   
  
  
+ AGACGCTGTG ACTTGCATCA ATGAGTATTT GGAAGACGTT AGGTACGAGA TTGCAGCTCT TGAAGCTGAT   
  
  
+ TAGTTGTTCT CAATCTCATC ACTTCTTTAC CCCTTTCGAC TGTTAACTTG CTATGTTGCT AATTAGCTTA   
  
  
+ AGATATAAGA TATTTAGAAA CTCAAAAGTT ATGACATTTC TTGTTGTCAA AGTTATCTAT GTTGATTACT   
  
  
+ GTGGGTAGTG GCTACAAATA CAATAGCAAA TTCCAAATTA TTTAAAACAC AATATTTGAT TTGGAAAACA   
  
  
+ ATTGATTTAA AGAGGAACTT TGTTACAAAT ATAAAAGTTA TTAGCATTTT CGGTTCAAAA TGCATAAGAT   
  
  
+ GAGTTGTGTT TTTTTTGTCG TGTACACATC AAAAACTGTC ATACGGACAA TAAGCTAGTC GCTTTGACCC   
  
  
+ ATTCACCGTA TGTCCCAATG TCCTTGACCC TGCCAAATGA TAAGAACGCC AGCACAAAGG TTTTATAAGT   
  
  
+ TTCTCAAAGA GAAACAACAC AATGGGAAAA CACACCATTG CTTGACTTAC AAATGGAGAT CGGTTGGATC   
  
  
+ AATCTCTGGA AACAGGTTGA AGTAGAATCT AAGCCCGTCT GGGCTCATGT CTCTACACAG CAGACCTGAT   
  
  
+ CAAAACAAAT GGGAGAATGA GAATCACATA ACTCTGAAAG AGTCTATCTT TCTACGAGGT GGATTCTGTT   
  
  
+ TACCTTTGGT CTCTGCAGAT AGTAGATGTT CCTTGGCCAT GGCTGGAGCG ATTTTAAGCG TCAAAGCTGC   
  
  
+ ATCACTAGCA GTGACTCCAG TTCTTAAAGT TTCGGTTTTA GTCACAAGCG TTCT  

- CTTGACAATT TCGGTTAGTA AATTTTATTT TTCAGTTTGG CCAAATTTGG CCAAAAGTTA TTCAATTATT   
  
  
- GGCCTGAGCC ACAATTTCTC TAATTTAAAT TGCCAAATTG AGTCACTCTA GCCAAAGCGA AAGAGAGACA   
  
  
- GCACAAACTC TTCAACTTTC TCTCCTCCTA CTCGCGACTT CTAAAGGTCT TCTTCCAAAG CTAGTCTCTG   
  
  
- AGGAGGCAGC GGCCTCTTTA CCTTTAGCTT ACGCCGCCCA GATGATCGAG GCGCCAACCA AGCAGAGCTT   
  
  
- GGAACCAAAA CGAAGCCTCT GACGAGCTCT AAGTCCTCGC AGCTCGTGTC CGGATGCGGT TTGAGTTTTC   
  
  
- CAAGCTAGAA ACCTCACAAA TACGATAATC AATGACAAAA ACTAAGTCAA TGAACCACTA ACAATGACTC   
  
  
- CCTACAACCT CAATTCATTG ACTATCACAA CAACTCCTTC GCTACAGAGT ATTTCAAATC TAAAAATAGA   
  
  
- CAACTAGAGA ACGATACGAA AACCTTAAAC TAATTAGTCT CCCAAGAGTC TCATACACCT CTGATCACCA   
  
  
- CTTCGCGAAA TAGTCTTCGA GACGTCGCTC TATTGACGAC TCAAGTTGCT CACAAGGTTT GTTCATTGAC   
  
  
- TCAAAAAAAA AGAAGAAAAC TAACTAATGG AAATACTACA ACAACTTCTA TGCCACAATT TCTAACGCAG   
  
  
- AAACAAACTA CCGTTACGTT CCATGCGCTT TACCTTTGAG ACAAAGACTT AGGACTGCAA CCTTCTAGCC   
  
  
- TAGAACGAGT TGACGAGTCA CTGTAAGTTT GAGTCCTCTT CGTCTTTGAC GTAGACCAGA CTAAGGATAT   
  
  
- ATGAGTTAAC AACTTGTATT GACAATTGAA CAACCTGTCT TGTTTTCAAA GAATGAAATC AATCAAAAAA   
  
  
- AAAAAAAAAC GTCTGCCAAT GTTATGTCCA TGACTTCTTC CGTCCCTCCG GCAGTCTTGC TTACGACTGT   
  
  
- GTGCTCTTGA CGTTCAAGTT CTTTGGCTAC GTCGTGCTCA CACACGTAGA AGTACTCTAA TGTCTTCGAC   
  
  
- TTCCTTGTCT CCTTCGTCTC CGCCTACGTC TCAAACTGTT GCGAAACTTC CTTCGTTAGT CTCCTCACGT   
  
  
- TCTGCGACAC TGAACGTAGT TACTCATAAA CCTTCTGCAA TCCATGCTCT AACGTCGAGA ACTTCGACTA   
  
  
- ATCAACAAGA GTTAGAGTAG TGAAGAAATG GGGAAAGCTG ACAATTGAAC GATACAACGA TTAATCGAAT   
  
  
- TCTATATTCT ATAAATCTTT GAGTTTTCAA TACTGTAAAG AACAACAGTT TCAATAGATA CAACTAATGA   
  
  
- CACCCATCAC CGATGTTTAT GTTATCGTTT AAGGTTTAAT AAATTTTGTG TTATAAACTA AACCTTTTGT   
  
  
- TAACTAAATT TCTCCTTGAA ACAATGTTTA TATTTTCAAT AATCGTAAAA GCCAAGTTTT ACGTATTCTA   
  
  
- CTCAACACAA AAAAAACAGC ACATGTGTAG TTTTTGACAG TATGCCTGTT ATTCGATCAG CGAAACTGGG   
  
  
- TAAGTGGCAT ACAGGGTTAC AGGAACTGGG ACGGTTTACT ATTCTTGCGG TCGTGTTTCC AAAATATTCA   
  
  
- AAGAGTTTCT CTTTGTTGTG TTACCCTTTT GTGTGGTAAC GAACTGAATG TTTACCTCTA GCCAACCTAG   
  
  
- TTAGAGACCT TTGTCCAACT TCATCTTAGA TTCGGGCAGA CCCGAGTACA GAGATGTGTC GTCTGGACTA   
  
  
- GTTTTGTTTA CCCTCTTACT CTTAGTGTAT TGAGACTTTC TCAGATAGAA AGATGCTCCA CCTAAGACAA   
  
  
- ATGGAAACCA GAGACGTCTA TCATCTACAA GGAACCGGTA CCGACCTCGC TAAAATTCGC AGTTTCGACG   
  
  
- TAGTGATCGT CACTGAGGTC AAGAATTTCA AAGCCAAAAT CAGTGTTCGC AAGA

+     TCT-motif

| Site Name | Organism | Position | Strand | Matrix score. | sequence | function |
| --- | --- | --- | --- | --- | --- | --- |
| TCT-motif | Arabidopsis thaliana | 890 | + | 6 | TCTTAC | part of a light responsive element |

>PlantCARE\_8972   
+ GAACTGTTAA AGCCAATCAT TTAAAATAAA AAGTCAAACC GGTTTAAACC GGTTTTCAAT AAGTTAATAA   
  
  
+ CCGGACTCGG TGTTAAAGAG ATTAAATTTA ACGGTTTAAC TCAGTGAGAT CGGTTTCGCT TTCTCTCTGT   
  
  
+ CGTGTTTGAG AAGTTGAAAG AGAGGAGGAT GAGCGCTGAA GATTTCCAGA AGAAGGTTTC GATCAGAGAC   
  
  
+ TCCTCCGTCG CCGGAGAAAT GGAAATCGAA TGCGGCGGGT CTACTAGCTC CGCGGTTGGT TCGTCTCGAA   
  
  
+ CCTTGGTTTT GCTTCGGAGA CTGCTCGAGA TTCAGGAGCG TCGAGCACAG GCCTACGCCA AACTCAAAAG   
  
  
+ GTTCGATCTT TGGAGTGTTT ATGCTATTAG TTACTGTTTT TGATTCAGTT ACTTGGTGAT TGTTACTGAG   
  
  
+ GGATGTTGGA GTTAAGTAAC TGATAGTGTT GTTGAGGAAG CGATGTCTCA TAAAGTTTAG ATTTTTATCT   
  
  
+ GTTGATCTCT TGCTATGCTT TTGGAATTTG ATTAATCAGA GGGTTCTCAG AGTATGTGGA GACTAGTGGT   
  
  
+ GAAGCGCTTT ATCAGAAGCT CTGCAGCGAG ATAACTGCTG AGTTCAACGA GTGTTCCAAA CAAGTAACTG   
  
  
+ AGTTTTTTTT TCTTCTTTTG ATTGATTACC TTTATGATGT TGTTGAAGAT ACGGTGTTAA AGATTGCGTC   
  
  
+ TTTGTTTGAT GGCAATGCAA GGTACGCGAA ATGGAAACTC TGTTTCTGAA TCCTGACGTT GGAAGATCGG   
  
  
+ ATCTTGCTCA ACTGCTCAGT GACATTCAAA CTCAGGAGAA GCAGAAACTG CATCTGGTCT GATTCCTATA   
  
  
+ TACTCAATTG TTGAACATAA CTGTTAACTT GTTGGACAGA ACAAAAGTTT CTTACTTTAG TTAGTTTTTT   
  
  
+ TTTTTTTTTG CAGACGGTTA CAATACAGGT ACTGAAGAAG GCAGGGAGGC CGTCAGAACG AATGCTGACA   
  
  
+ CACGAGAACT GCAAGTTCAA GAAACCGATG CAGCACGAGT GTGTGCATCT TCATGAGATT ACAGAAGCTG   
  
  
+ AAGGAACAGA GGAAGCAGAG GCGGATGCAG AGTTTGACAA CGCTTTGAAG GAAGCAATCA GAGGAGTGCA   
  
  
+ AGACGCTGTG ACTTGCATCA ATGAGTATTT GGAAGACGTT AGGTACGAGA TTGCAGCTCT TGAAGCTGAT   
  
  
+ TAGTTGTTCT CAATCTCATC ACTTCTTTAC CCCTTTCGAC TGTTAACTTG CTATGTTGCT AATTAGCTTA   
  
  
+ AGATATAAGA TATTTAGAAA CTCAAAAGTT ATGACATTTC TTGTTGTCAA AGTTATCTAT GTTGATTACT   
  
  
+ GTGGGTAGTG GCTACAAATA CAATAGCAAA TTCCAAATTA TTTAAAACAC AATATTTGAT TTGGAAAACA   
  
  
+ ATTGATTTAA AGAGGAACTT TGTTACAAAT ATAAAAGTTA TTAGCATTTT CGGTTCAAAA TGCATAAGAT   
  
  
+ GAGTTGTGTT TTTTTTGTCG TGTACACATC AAAAACTGTC ATACGGACAA TAAGCTAGTC GCTTTGACCC   
  
  
+ ATTCACCGTA TGTCCCAATG TCCTTGACCC TGCCAAATGA TAAGAACGCC AGCACAAAGG TTTTATAAGT   
  
  
+ TTCTCAAAGA GAAACAACAC AATGGGAAAA CACACCATTG CTTGACTTAC AAATGGAGAT CGGTTGGATC   
  
  
+ AATCTCTGGA AACAGGTTGA AGTAGAATCT AAGCCCGTCT GGGCTCATGT CTCTACACAG CAGACCTGAT   
  
  
+ CAAAACAAAT GGGAGAATGA GAATCACATA ACTCTGAAAG AGTCTATCTT TCTACGAGGT GGATTCTGTT   
  
  
+ TACCTTTGGT CTCTGCAGAT AGTAGATGTT CCTTGGCCAT GGCTGGAGCG ATTTTAAGCG TCAAAGCTGC   
  
  
+ ATCACTAGCA GTGACTCCAG TTCTTAAAGT TTCGGTTTTA GTCACAAGCG TTCT  

- CTTGACAATT TCGGTTAGTA AATTTTATTT TTCAGTTTGG CCAAATTTGG CCAAAAGTTA TTCAATTATT   
  
  
- GGCCTGAGCC ACAATTTCTC TAATTTAAAT TGCCAAATTG AGTCACTCTA GCCAAAGCGA AAGAGAGACA   
  
  
- GCACAAACTC TTCAACTTTC TCTCCTCCTA CTCGCGACTT CTAAAGGTCT TCTTCCAAAG CTAGTCTCTG   
  
  
- AGGAGGCAGC GGCCTCTTTA CCTTTAGCTT ACGCCGCCCA GATGATCGAG GCGCCAACCA AGCAGAGCTT   
  
  
- GGAACCAAAA CGAAGCCTCT GACGAGCTCT AAGTCCTCGC AGCTCGTGTC CGGATGCGGT TTGAGTTTTC   
  
  
- CAAGCTAGAA ACCTCACAAA TACGATAATC AATGACAAAA ACTAAGTCAA TGAACCACTA ACAATGACTC   
  
  
- CCTACAACCT CAATTCATTG ACTATCACAA CAACTCCTTC GCTACAGAGT ATTTCAAATC TAAAAATAGA   
  
  
- CAACTAGAGA ACGATACGAA AACCTTAAAC TAATTAGTCT CCCAAGAGTC TCATACACCT CTGATCACCA   
  
  
- CTTCGCGAAA TAGTCTTCGA GACGTCGCTC TATTGACGAC TCAAGTTGCT CACAAGGTTT GTTCATTGAC   
  
  
- TCAAAAAAAA AGAAGAAAAC TAACTAATGG AAATACTACA ACAACTTCTA TGCCACAATT TCTAACGCAG   
  
  
- AAACAAACTA CCGTTACGTT CCATGCGCTT TACCTTTGAG ACAAAGACTT AGGACTGCAA CCTTCTAGCC   
  
  
- TAGAACGAGT TGACGAGTCA CTGTAAGTTT GAGTCCTCTT CGTCTTTGAC GTAGACCAGA CTAAGGATAT   
  
  
- ATGAGTTAAC AACTTGTATT GACAATTGAA CAACCTGTCT TGTTTTCAAA GAATGAAATC AATCAAAAAA   
  
  
- AAAAAAAAAC GTCTGCCAAT GTTATGTCCA TGACTTCTTC CGTCCCTCCG GCAGTCTTGC TTACGACTGT   
  
  
- GTGCTCTTGA CGTTCAAGTT CTTTGGCTAC GTCGTGCTCA CACACGTAGA AGTACTCTAA TGTCTTCGAC   
  
  
- TTCCTTGTCT CCTTCGTCTC CGCCTACGTC TCAAACTGTT GCGAAACTTC CTTCGTTAGT CTCCTCACGT   
  
  
- TCTGCGACAC TGAACGTAGT TACTCATAAA CCTTCTGCAA TCCATGCTCT AACGTCGAGA ACTTCGACTA   
  
  
- ATCAACAAGA GTTAGAGTAG TGAAGAAATG GGGAAAGCTG ACAATTGAAC GATACAACGA TTAATCGAAT   
  
  
- TCTATATTCT ATAAATCTTT GAGTTTTCAA TACTGTAAAG AACAACAGTT TCAATAGATA CAACTAATGA   
  
  
- CACCCATCAC CGATGTTTAT GTTATCGTTT AAGGTTTAAT AAATTTTGTG TTATAAACTA AACCTTTTGT   
  
  
- TAACTAAATT TCTCCTTGAA ACAATGTTTA TATTTTCAAT AATCGTAAAA GCCAAGTTTT ACGTATTCTA   
  
  
- CTCAACACAA AAAAAACAGC ACATGTGTAG TTTTTGACAG TATGCCTGTT ATTCGATCAG CGAAACTGGG   
  
  
- TAAGTGGCAT ACAGGGTTAC AGGAACTGGG ACGGTTTACT ATTCTTGCGG TCGTGTTTCC AAAATATTCA   
  
  
- AAGAGTTTCT CTTTGTTGTG TTACCCTTTT GTGTGGTAAC GAACTGAATG TTTACCTCTA GCCAACCTAG   
  
  
- TTAGAGACCT TTGTCCAACT TCATCTTAGA TTCGGGCAGA CCCGAGTACA GAGATGTGTC GTCTGGACTA   
  
  
- GTTTTGTTTA CCCTCTTACT CTTAGTGTAT TGAGACTTTC TCAGATAGAA AGATGCTCCA CCTAAGACAA   
  
  
- ATGGAAACCA GAGACGTCTA TCATCTACAA GGAACCGGTA CCGACCTCGC TAAAATTCGC AGTTTCGACG   
  
  
- TAGTGATCGT CACTGAGGTC AAGAATTTCA AAGCCAAAAT CAGTGTTCGC AAGA

+     TGACG-motif

| Site Name | Organism | Position | Strand | Matrix score. | sequence | function |
| --- | --- | --- | --- | --- | --- | --- |
| TGACG-motif | Hordeum vulgare | 754 | + | 5 | TGACG | cis-acting regulatory element involved in the MeJA-responsiveness |
| TGACG-motif | Hordeum vulgare | 1879 | - | 5 | TGACG | cis-acting regulatory element involved in the MeJA-responsiveness |
| TGACG-motif | Hordeum vulgare | 961 | - | 5 | TGACG | cis-acting regulatory element involved in the MeJA-responsiveness |

>PlantCARE\_8972   
+ GAACTGTTAA AGCCAATCAT TTAAAATAAA AAGTCAAACC GGTTTAAACC GGTTTTCAAT AAGTTAATAA   
  
  
+ CCGGACTCGG TGTTAAAGAG ATTAAATTTA ACGGTTTAAC TCAGTGAGAT CGGTTTCGCT TTCTCTCTGT   
  
  
+ CGTGTTTGAG AAGTTGAAAG AGAGGAGGAT GAGCGCTGAA GATTTCCAGA AGAAGGTTTC GATCAGAGAC   
  
  
+ TCCTCCGTCG CCGGAGAAAT GGAAATCGAA TGCGGCGGGT CTACTAGCTC CGCGGTTGGT TCGTCTCGAA   
  
  
+ CCTTGGTTTT GCTTCGGAGA CTGCTCGAGA TTCAGGAGCG TCGAGCACAG GCCTACGCCA AACTCAAAAG   
  
  
+ GTTCGATCTT TGGAGTGTTT ATGCTATTAG TTACTGTTTT TGATTCAGTT ACTTGGTGAT TGTTACTGAG   
  
  
+ GGATGTTGGA GTTAAGTAAC TGATAGTGTT GTTGAGGAAG CGATGTCTCA TAAAGTTTAG ATTTTTATCT   
  
  
+ GTTGATCTCT TGCTATGCTT TTGGAATTTG ATTAATCAGA GGGTTCTCAG AGTATGTGGA GACTAGTGGT   
  
  
+ GAAGCGCTTT ATCAGAAGCT CTGCAGCGAG ATAACTGCTG AGTTCAACGA GTGTTCCAAA CAAGTAACTG   
  
  
+ AGTTTTTTTT TCTTCTTTTG ATTGATTACC TTTATGATGT TGTTGAAGAT ACGGTGTTAA AGATTGCGTC   
  
  
+ TTTGTTTGAT GGCAATGCAA GGTACGCGAA ATGGAAACTC TGTTTCTGAA TCCTGACGTT GGAAGATCGG   
  
  
+ ATCTTGCTCA ACTGCTCAGT GACATTCAAA CTCAGGAGAA GCAGAAACTG CATCTGGTCT GATTCCTATA   
  
  
+ TACTCAATTG TTGAACATAA CTGTTAACTT GTTGGACAGA ACAAAAGTTT CTTACTTTAG TTAGTTTTTT   
  
  
+ TTTTTTTTTG CAGACGGTTA CAATACAGGT ACTGAAGAAG GCAGGGAGGC CGTCAGAACG AATGCTGACA   
  
  
+ CACGAGAACT GCAAGTTCAA GAAACCGATG CAGCACGAGT GTGTGCATCT TCATGAGATT ACAGAAGCTG   
  
  
+ AAGGAACAGA GGAAGCAGAG GCGGATGCAG AGTTTGACAA CGCTTTGAAG GAAGCAATCA GAGGAGTGCA   
  
  
+ AGACGCTGTG ACTTGCATCA ATGAGTATTT GGAAGACGTT AGGTACGAGA TTGCAGCTCT TGAAGCTGAT   
  
  
+ TAGTTGTTCT CAATCTCATC ACTTCTTTAC CCCTTTCGAC TGTTAACTTG CTATGTTGCT AATTAGCTTA   
  
  
+ AGATATAAGA TATTTAGAAA CTCAAAAGTT ATGACATTTC TTGTTGTCAA AGTTATCTAT GTTGATTACT   
  
  
+ GTGGGTAGTG GCTACAAATA CAATAGCAAA TTCCAAATTA TTTAAAACAC AATATTTGAT TTGGAAAACA   
  
  
+ ATTGATTTAA AGAGGAACTT TGTTACAAAT ATAAAAGTTA TTAGCATTTT CGGTTCAAAA TGCATAAGAT   
  
  
+ GAGTTGTGTT TTTTTTGTCG TGTACACATC AAAAACTGTC ATACGGACAA TAAGCTAGTC GCTTTGACCC   
  
  
+ ATTCACCGTA TGTCCCAATG TCCTTGACCC TGCCAAATGA TAAGAACGCC AGCACAAAGG TTTTATAAGT   
  
  
+ TTCTCAAAGA GAAACAACAC AATGGGAAAA CACACCATTG CTTGACTTAC AAATGGAGAT CGGTTGGATC   
  
  
+ AATCTCTGGA AACAGGTTGA AGTAGAATCT AAGCCCGTCT GGGCTCATGT CTCTACACAG CAGACCTGAT   
  
  
+ CAAAACAAAT GGGAGAATGA GAATCACATA ACTCTGAAAG AGTCTATCTT TCTACGAGGT GGATTCTGTT   
  
  
+ TACCTTTGGT CTCTGCAGAT AGTAGATGTT CCTTGGCCAT GGCTGGAGCG ATTTTAAGCG TCAAAGCTGC   
  
  
+ ATCACTAGCA GTGACTCCAG TTCTTAAAGT TTCGGTTTTA GTCACAAGCG TTCT  

- CTTGACAATT TCGGTTAGTA AATTTTATTT TTCAGTTTGG CCAAATTTGG CCAAAAGTTA TTCAATTATT   
  
  
- GGCCTGAGCC ACAATTTCTC TAATTTAAAT TGCCAAATTG AGTCACTCTA GCCAAAGCGA AAGAGAGACA   
  
  
- GCACAAACTC TTCAACTTTC TCTCCTCCTA CTCGCGACTT CTAAAGGTCT TCTTCCAAAG CTAGTCTCTG   
  
  
- AGGAGGCAGC GGCCTCTTTA CCTTTAGCTT ACGCCGCCCA GATGATCGAG GCGCCAACCA AGCAGAGCTT   
  
  
- GGAACCAAAA CGAAGCCTCT GACGAGCTCT AAGTCCTCGC AGCTCGTGTC CGGATGCGGT TTGAGTTTTC   
  
  
- CAAGCTAGAA ACCTCACAAA TACGATAATC AATGACAAAA ACTAAGTCAA TGAACCACTA ACAATGACTC   
  
  
- CCTACAACCT CAATTCATTG ACTATCACAA CAACTCCTTC GCTACAGAGT ATTTCAAATC TAAAAATAGA   
  
  
- CAACTAGAGA ACGATACGAA AACCTTAAAC TAATTAGTCT CCCAAGAGTC TCATACACCT CTGATCACCA   
  
  
- CTTCGCGAAA TAGTCTTCGA GACGTCGCTC TATTGACGAC TCAAGTTGCT CACAAGGTTT GTTCATTGAC   
  
  
- TCAAAAAAAA AGAAGAAAAC TAACTAATGG AAATACTACA ACAACTTCTA TGCCACAATT TCTAACGCAG   
  
  
- AAACAAACTA CCGTTACGTT CCATGCGCTT TACCTTTGAG ACAAAGACTT AGGACTGCAA CCTTCTAGCC   
  
  
- TAGAACGAGT TGACGAGTCA CTGTAAGTTT GAGTCCTCTT CGTCTTTGAC GTAGACCAGA CTAAGGATAT   
  
  
- ATGAGTTAAC AACTTGTATT GACAATTGAA CAACCTGTCT TGTTTTCAAA GAATGAAATC AATCAAAAAA   
  
  
- AAAAAAAAAC GTCTGCCAAT GTTATGTCCA TGACTTCTTC CGTCCCTCCG GCAGTCTTGC TTACGACTGT   
  
  
- GTGCTCTTGA CGTTCAAGTT CTTTGGCTAC GTCGTGCTCA CACACGTAGA AGTACTCTAA TGTCTTCGAC   
  
  
- TTCCTTGTCT CCTTCGTCTC CGCCTACGTC TCAAACTGTT GCGAAACTTC CTTCGTTAGT CTCCTCACGT   
  
  
- TCTGCGACAC TGAACGTAGT TACTCATAAA CCTTCTGCAA TCCATGCTCT AACGTCGAGA ACTTCGACTA   
  
  
- ATCAACAAGA GTTAGAGTAG TGAAGAAATG GGGAAAGCTG ACAATTGAAC GATACAACGA TTAATCGAAT   
  
  
- TCTATATTCT ATAAATCTTT GAGTTTTCAA TACTGTAAAG AACAACAGTT TCAATAGATA CAACTAATGA   
  
  
- CACCCATCAC CGATGTTTAT GTTATCGTTT AAGGTTTAAT AAATTTTGTG TTATAAACTA AACCTTTTGT   
  
  
- TAACTAAATT TCTCCTTGAA ACAATGTTTA TATTTTCAAT AATCGTAAAA GCCAAGTTTT ACGTATTCTA   
  
  
- CTCAACACAA AAAAAACAGC ACATGTGTAG TTTTTGACAG TATGCCTGTT ATTCGATCAG CGAAACTGGG   
  
  
- TAAGTGGCAT ACAGGGTTAC AGGAACTGGG ACGGTTTACT ATTCTTGCGG TCGTGTTTCC AAAATATTCA   
  
  
- AAGAGTTTCT CTTTGTTGTG TTACCCTTTT GTGTGGTAAC GAACTGAATG TTTACCTCTA GCCAACCTAG   
  
  
- TTAGAGACCT TTGTCCAACT TCATCTTAGA TTCGGGCAGA CCCGAGTACA GAGATGTGTC GTCTGGACTA   
  
  
- GTTTTGTTTA CCCTCTTACT CTTAGTGTAT TGAGACTTTC TCAGATAGAA AGATGCTCCA CCTAAGACAA   
  
  
- ATGGAAACCA GAGACGTCTA TCATCTACAA GGAACCGGTA CCGACCTCGC TAAAATTCGC AGTTTCGACG   
  
  
- TAGTGATCGT CACTGAGGTC AAGAATTTCA AAGCCAAAAT CAGTGTTCGC AAGA

+     Unnamed\_\_1

| Site Name | Organism | Position | Strand | Matrix score. | sequence | function |
| --- | --- | --- | --- | --- | --- | --- |
| Unnamed\_\_1 | Glycine max | 514 | + | 11 | GAATTTAATTAA | 60K protein binding site |

>PlantCARE\_8972   
+ GAACTGTTAA AGCCAATCAT TTAAAATAAA AAGTCAAACC GGTTTAAACC GGTTTTCAAT AAGTTAATAA   
  
  
+ CCGGACTCGG TGTTAAAGAG ATTAAATTTA ACGGTTTAAC TCAGTGAGAT CGGTTTCGCT TTCTCTCTGT   
  
  
+ CGTGTTTGAG AAGTTGAAAG AGAGGAGGAT GAGCGCTGAA GATTTCCAGA AGAAGGTTTC GATCAGAGAC   
  
  
+ TCCTCCGTCG CCGGAGAAAT GGAAATCGAA TGCGGCGGGT CTACTAGCTC CGCGGTTGGT TCGTCTCGAA   
  
  
+ CCTTGGTTTT GCTTCGGAGA CTGCTCGAGA TTCAGGAGCG TCGAGCACAG GCCTACGCCA AACTCAAAAG   
  
  
+ GTTCGATCTT TGGAGTGTTT ATGCTATTAG TTACTGTTTT TGATTCAGTT ACTTGGTGAT TGTTACTGAG   
  
  
+ GGATGTTGGA GTTAAGTAAC TGATAGTGTT GTTGAGGAAG CGATGTCTCA TAAAGTTTAG ATTTTTATCT   
  
  
+ GTTGATCTCT TGCTATGCTT TTGGAATTTG ATTAATCAGA GGGTTCTCAG AGTATGTGGA GACTAGTGGT   
  
  
+ GAAGCGCTTT ATCAGAAGCT CTGCAGCGAG ATAACTGCTG AGTTCAACGA GTGTTCCAAA CAAGTAACTG   
  
  
+ AGTTTTTTTT TCTTCTTTTG ATTGATTACC TTTATGATGT TGTTGAAGAT ACGGTGTTAA AGATTGCGTC   
  
  
+ TTTGTTTGAT GGCAATGCAA GGTACGCGAA ATGGAAACTC TGTTTCTGAA TCCTGACGTT GGAAGATCGG   
  
  
+ ATCTTGCTCA ACTGCTCAGT GACATTCAAA CTCAGGAGAA GCAGAAACTG CATCTGGTCT GATTCCTATA   
  
  
+ TACTCAATTG TTGAACATAA CTGTTAACTT GTTGGACAGA ACAAAAGTTT CTTACTTTAG TTAGTTTTTT   
  
  
+ TTTTTTTTTG CAGACGGTTA CAATACAGGT ACTGAAGAAG GCAGGGAGGC CGTCAGAACG AATGCTGACA   
  
  
+ CACGAGAACT GCAAGTTCAA GAAACCGATG CAGCACGAGT GTGTGCATCT TCATGAGATT ACAGAAGCTG   
  
  
+ AAGGAACAGA GGAAGCAGAG GCGGATGCAG AGTTTGACAA CGCTTTGAAG GAAGCAATCA GAGGAGTGCA   
  
  
+ AGACGCTGTG ACTTGCATCA ATGAGTATTT GGAAGACGTT AGGTACGAGA TTGCAGCTCT TGAAGCTGAT   
  
  
+ TAGTTGTTCT CAATCTCATC ACTTCTTTAC CCCTTTCGAC TGTTAACTTG CTATGTTGCT AATTAGCTTA   
  
  
+ AGATATAAGA TATTTAGAAA CTCAAAAGTT ATGACATTTC TTGTTGTCAA AGTTATCTAT GTTGATTACT   
  
  
+ GTGGGTAGTG GCTACAAATA CAATAGCAAA TTCCAAATTA TTTAAAACAC AATATTTGAT TTGGAAAACA   
  
  
+ ATTGATTTAA AGAGGAACTT TGTTACAAAT ATAAAAGTTA TTAGCATTTT CGGTTCAAAA TGCATAAGAT   
  
  
+ GAGTTGTGTT TTTTTTGTCG TGTACACATC AAAAACTGTC ATACGGACAA TAAGCTAGTC GCTTTGACCC   
  
  
+ ATTCACCGTA TGTCCCAATG TCCTTGACCC TGCCAAATGA TAAGAACGCC AGCACAAAGG TTTTATAAGT   
  
  
+ TTCTCAAAGA GAAACAACAC AATGGGAAAA CACACCATTG CTTGACTTAC AAATGGAGAT CGGTTGGATC   
  
  
+ AATCTCTGGA AACAGGTTGA AGTAGAATCT AAGCCCGTCT GGGCTCATGT CTCTACACAG CAGACCTGAT   
  
  
+ CAAAACAAAT GGGAGAATGA GAATCACATA ACTCTGAAAG AGTCTATCTT TCTACGAGGT GGATTCTGTT   
  
  
+ TACCTTTGGT CTCTGCAGAT AGTAGATGTT CCTTGGCCAT GGCTGGAGCG ATTTTAAGCG TCAAAGCTGC   
  
  
+ ATCACTAGCA GTGACTCCAG TTCTTAAAGT TTCGGTTTTA GTCACAAGCG TTCT  

- CTTGACAATT TCGGTTAGTA AATTTTATTT TTCAGTTTGG CCAAATTTGG CCAAAAGTTA TTCAATTATT   
  
  
- GGCCTGAGCC ACAATTTCTC TAATTTAAAT TGCCAAATTG AGTCACTCTA GCCAAAGCGA AAGAGAGACA   
  
  
- GCACAAACTC TTCAACTTTC TCTCCTCCTA CTCGCGACTT CTAAAGGTCT TCTTCCAAAG CTAGTCTCTG   
  
  
- AGGAGGCAGC GGCCTCTTTA CCTTTAGCTT ACGCCGCCCA GATGATCGAG GCGCCAACCA AGCAGAGCTT   
  
  
- GGAACCAAAA CGAAGCCTCT GACGAGCTCT AAGTCCTCGC AGCTCGTGTC CGGATGCGGT TTGAGTTTTC   
  
  
- CAAGCTAGAA ACCTCACAAA TACGATAATC AATGACAAAA ACTAAGTCAA TGAACCACTA ACAATGACTC   
  
  
- CCTACAACCT CAATTCATTG ACTATCACAA CAACTCCTTC GCTACAGAGT ATTTCAAATC TAAAAATAGA   
  
  
- CAACTAGAGA ACGATACGAA AACCTTAAAC TAATTAGTCT CCCAAGAGTC TCATACACCT CTGATCACCA   
  
  
- CTTCGCGAAA TAGTCTTCGA GACGTCGCTC TATTGACGAC TCAAGTTGCT CACAAGGTTT GTTCATTGAC   
  
  
- TCAAAAAAAA AGAAGAAAAC TAACTAATGG AAATACTACA ACAACTTCTA TGCCACAATT TCTAACGCAG   
  
  
- AAACAAACTA CCGTTACGTT CCATGCGCTT TACCTTTGAG ACAAAGACTT AGGACTGCAA CCTTCTAGCC   
  
  
- TAGAACGAGT TGACGAGTCA CTGTAAGTTT GAGTCCTCTT CGTCTTTGAC GTAGACCAGA CTAAGGATAT   
  
  
- ATGAGTTAAC AACTTGTATT GACAATTGAA CAACCTGTCT TGTTTTCAAA GAATGAAATC AATCAAAAAA   
  
  
- AAAAAAAAAC GTCTGCCAAT GTTATGTCCA TGACTTCTTC CGTCCCTCCG GCAGTCTTGC TTACGACTGT   
  
  
- GTGCTCTTGA CGTTCAAGTT CTTTGGCTAC GTCGTGCTCA CACACGTAGA AGTACTCTAA TGTCTTCGAC   
  
  
- TTCCTTGTCT CCTTCGTCTC CGCCTACGTC TCAAACTGTT GCGAAACTTC CTTCGTTAGT CTCCTCACGT   
  
  
- TCTGCGACAC TGAACGTAGT TACTCATAAA CCTTCTGCAA TCCATGCTCT AACGTCGAGA ACTTCGACTA   
  
  
- ATCAACAAGA GTTAGAGTAG TGAAGAAATG GGGAAAGCTG ACAATTGAAC GATACAACGA TTAATCGAAT   
  
  
- TCTATATTCT ATAAATCTTT GAGTTTTCAA TACTGTAAAG AACAACAGTT TCAATAGATA CAACTAATGA   
  
  
- CACCCATCAC CGATGTTTAT GTTATCGTTT AAGGTTTAAT AAATTTTGTG TTATAAACTA AACCTTTTGT   
  
  
- TAACTAAATT TCTCCTTGAA ACAATGTTTA TATTTTCAAT AATCGTAAAA GCCAAGTTTT ACGTATTCTA   
  
  
- CTCAACACAA AAAAAACAGC ACATGTGTAG TTTTTGACAG TATGCCTGTT ATTCGATCAG CGAAACTGGG   
  
  
- TAAGTGGCAT ACAGGGTTAC AGGAACTGGG ACGGTTTACT ATTCTTGCGG TCGTGTTTCC AAAATATTCA   
  
  
- AAGAGTTTCT CTTTGTTGTG TTACCCTTTT GTGTGGTAAC GAACTGAATG TTTACCTCTA GCCAACCTAG   
  
  
- TTAGAGACCT TTGTCCAACT TCATCTTAGA TTCGGGCAGA CCCGAGTACA GAGATGTGTC GTCTGGACTA   
  
  
- GTTTTGTTTA CCCTCTTACT CTTAGTGTAT TGAGACTTTC TCAGATAGAA AGATGCTCCA CCTAAGACAA   
  
  
- ATGGAAACCA GAGACGTCTA TCATCTACAA GGAACCGGTA CCGACCTCGC TAAAATTCGC AGTTTCGACG   
  
  
- TAGTGATCGT CACTGAGGTC AAGAATTTCA AAGCCAAAAT CAGTGTTCGC AAGA

+     Unnamed\_\_4

| Site Name | Organism | Position | Strand | Matrix score. | sequence | function |
| --- | --- | --- | --- | --- | --- | --- |
| Unnamed\_\_4 | Petroselinum hortense | 164 | - | 4 | CTCC |  |
| Unnamed\_\_4 | Petroselinum hortense | 805 | - | 4 | CTCC |  |
| Unnamed\_\_4 | Petroselinum hortense | 315 | - | 4 | CTCC |  |
| Unnamed\_\_4 | Petroselinum hortense | 1762 | - | 4 | CTCC |  |
| Unnamed\_\_4 | Petroselinum hortense | 258 | + | 4 | CTCC |  |
| Unnamed\_\_4 | Petroselinum hortense | 1113 | - | 4 | CTCC |  |
| Unnamed\_\_4 | Petroselinum hortense | 428 | - | 4 | CTCC |  |
| Unnamed\_\_4 | Petroselinum hortense | 1905 | + | 4 | CTCC |  |
| Unnamed\_\_4 | Petroselinum hortense | 213 | + | 4 | CTCC |  |
| Unnamed\_\_4 | Petroselinum hortense | 955 | - | 4 | CTCC |  |
| Unnamed\_\_4 | Petroselinum hortense | 362 | - | 4 | CTCC |  |
| Unnamed\_\_4 | Petroselinum hortense | 1865 | - | 4 | CTCC |  |
| Unnamed\_\_4 | Petroselinum hortense | 296 | - | 4 | CTCC |  |
| Unnamed\_\_4 | Petroselinum hortense | 1665 | - | 4 | CTCC |  |
| Unnamed\_\_4 | Petroselinum hortense | 548 | - | 4 | CTCC |  |
| Unnamed\_\_4 | Petroselinum hortense | 210 | + | 4 | CTCC |  |
| Unnamed\_\_4 | Petroselinum hortense | 223 | - | 4 | CTCC |  |

>PlantCARE\_8972   
+ GAACTGTTAA AGCCAATCAT TTAAAATAAA AAGTCAAACC GGTTTAAACC GGTTTTCAAT AAGTTAATAA   
  
  
+ CCGGACTCGG TGTTAAAGAG ATTAAATTTA ACGGTTTAAC TCAGTGAGAT CGGTTTCGCT TTCTCTCTGT   
  
  
+ CGTGTTTGAG AAGTTGAAAG AGAGGAGGAT GAGCGCTGAA GATTTCCAGA AGAAGGTTTC GATCAGAGAC   
  
  
+ TCCTCCGTCG CCGGAGAAAT GGAAATCGAA TGCGGCGGGT CTACTAGCTC CGCGGTTGGT TCGTCTCGAA   
  
  
+ CCTTGGTTTT GCTTCGGAGA CTGCTCGAGA TTCAGGAGCG TCGAGCACAG GCCTACGCCA AACTCAAAAG   
  
  
+ GTTCGATCTT TGGAGTGTTT ATGCTATTAG TTACTGTTTT TGATTCAGTT ACTTGGTGAT TGTTACTGAG   
  
  
+ GGATGTTGGA GTTAAGTAAC TGATAGTGTT GTTGAGGAAG CGATGTCTCA TAAAGTTTAG ATTTTTATCT   
  
  
+ GTTGATCTCT TGCTATGCTT TTGGAATTTG ATTAATCAGA GGGTTCTCAG AGTATGTGGA GACTAGTGGT   
  
  
+ GAAGCGCTTT ATCAGAAGCT CTGCAGCGAG ATAACTGCTG AGTTCAACGA GTGTTCCAAA CAAGTAACTG   
  
  
+ AGTTTTTTTT TCTTCTTTTG ATTGATTACC TTTATGATGT TGTTGAAGAT ACGGTGTTAA AGATTGCGTC   
  
  
+ TTTGTTTGAT GGCAATGCAA GGTACGCGAA ATGGAAACTC TGTTTCTGAA TCCTGACGTT GGAAGATCGG   
  
  
+ ATCTTGCTCA ACTGCTCAGT GACATTCAAA CTCAGGAGAA GCAGAAACTG CATCTGGTCT GATTCCTATA   
  
  
+ TACTCAATTG TTGAACATAA CTGTTAACTT GTTGGACAGA ACAAAAGTTT CTTACTTTAG TTAGTTTTTT   
  
  
+ TTTTTTTTTG CAGACGGTTA CAATACAGGT ACTGAAGAAG GCAGGGAGGC CGTCAGAACG AATGCTGACA   
  
  
+ CACGAGAACT GCAAGTTCAA GAAACCGATG CAGCACGAGT GTGTGCATCT TCATGAGATT ACAGAAGCTG   
  
  
+ AAGGAACAGA GGAAGCAGAG GCGGATGCAG AGTTTGACAA CGCTTTGAAG GAAGCAATCA GAGGAGTGCA   
  
  
+ AGACGCTGTG ACTTGCATCA ATGAGTATTT GGAAGACGTT AGGTACGAGA TTGCAGCTCT TGAAGCTGAT   
  
  
+ TAGTTGTTCT CAATCTCATC ACTTCTTTAC CCCTTTCGAC TGTTAACTTG CTATGTTGCT AATTAGCTTA   
  
  
+ AGATATAAGA TATTTAGAAA CTCAAAAGTT ATGACATTTC TTGTTGTCAA AGTTATCTAT GTTGATTACT   
  
  
+ GTGGGTAGTG GCTACAAATA CAATAGCAAA TTCCAAATTA TTTAAAACAC AATATTTGAT TTGGAAAACA   
  
  
+ ATTGATTTAA AGAGGAACTT TGTTACAAAT ATAAAAGTTA TTAGCATTTT CGGTTCAAAA TGCATAAGAT   
  
  
+ GAGTTGTGTT TTTTTTGTCG TGTACACATC AAAAACTGTC ATACGGACAA TAAGCTAGTC GCTTTGACCC   
  
  
+ ATTCACCGTA TGTCCCAATG TCCTTGACCC TGCCAAATGA TAAGAACGCC AGCACAAAGG TTTTATAAGT   
  
  
+ TTCTCAAAGA GAAACAACAC AATGGGAAAA CACACCATTG CTTGACTTAC AAATGGAGAT CGGTTGGATC   
  
  
+ AATCTCTGGA AACAGGTTGA AGTAGAATCT AAGCCCGTCT GGGCTCATGT CTCTACACAG CAGACCTGAT   
  
  
+ CAAAACAAAT GGGAGAATGA GAATCACATA ACTCTGAAAG AGTCTATCTT TCTACGAGGT GGATTCTGTT   
  
  
+ TACCTTTGGT CTCTGCAGAT AGTAGATGTT CCTTGGCCAT GGCTGGAGCG ATTTTAAGCG TCAAAGCTGC   
  
  
+ ATCACTAGCA GTGACTCCAG TTCTTAAAGT TTCGGTTTTA GTCACAAGCG TTCT  

- CTTGACAATT TCGGTTAGTA AATTTTATTT TTCAGTTTGG CCAAATTTGG CCAAAAGTTA TTCAATTATT   
  
  
- GGCCTGAGCC ACAATTTCTC TAATTTAAAT TGCCAAATTG AGTCACTCTA GCCAAAGCGA AAGAGAGACA   
  
  
- GCACAAACTC TTCAACTTTC TCTCCTCCTA CTCGCGACTT CTAAAGGTCT TCTTCCAAAG CTAGTCTCTG   
  
  
- AGGAGGCAGC GGCCTCTTTA CCTTTAGCTT ACGCCGCCCA GATGATCGAG GCGCCAACCA AGCAGAGCTT   
  
  
- GGAACCAAAA CGAAGCCTCT GACGAGCTCT AAGTCCTCGC AGCTCGTGTC CGGATGCGGT TTGAGTTTTC   
  
  
- CAAGCTAGAA ACCTCACAAA TACGATAATC AATGACAAAA ACTAAGTCAA TGAACCACTA ACAATGACTC   
  
  
- CCTACAACCT CAATTCATTG ACTATCACAA CAACTCCTTC GCTACAGAGT ATTTCAAATC TAAAAATAGA   
  
  
- CAACTAGAGA ACGATACGAA AACCTTAAAC TAATTAGTCT CCCAAGAGTC TCATACACCT CTGATCACCA   
  
  
- CTTCGCGAAA TAGTCTTCGA GACGTCGCTC TATTGACGAC TCAAGTTGCT CACAAGGTTT GTTCATTGAC   
  
  
- TCAAAAAAAA AGAAGAAAAC TAACTAATGG AAATACTACA ACAACTTCTA TGCCACAATT TCTAACGCAG   
  
  
- AAACAAACTA CCGTTACGTT CCATGCGCTT TACCTTTGAG ACAAAGACTT AGGACTGCAA CCTTCTAGCC   
  
  
- TAGAACGAGT TGACGAGTCA CTGTAAGTTT GAGTCCTCTT CGTCTTTGAC GTAGACCAGA CTAAGGATAT   
  
  
- ATGAGTTAAC AACTTGTATT GACAATTGAA CAACCTGTCT TGTTTTCAAA GAATGAAATC AATCAAAAAA   
  
  
- AAAAAAAAAC GTCTGCCAAT GTTATGTCCA TGACTTCTTC CGTCCCTCCG GCAGTCTTGC TTACGACTGT   
  
  
- GTGCTCTTGA CGTTCAAGTT CTTTGGCTAC GTCGTGCTCA CACACGTAGA AGTACTCTAA TGTCTTCGAC   
  
  
- TTCCTTGTCT CCTTCGTCTC CGCCTACGTC TCAAACTGTT GCGAAACTTC CTTCGTTAGT CTCCTCACGT   
  
  
- TCTGCGACAC TGAACGTAGT TACTCATAAA CCTTCTGCAA TCCATGCTCT AACGTCGAGA ACTTCGACTA   
  
  
- ATCAACAAGA GTTAGAGTAG TGAAGAAATG GGGAAAGCTG ACAATTGAAC GATACAACGA TTAATCGAAT   
  
  
- TCTATATTCT ATAAATCTTT GAGTTTTCAA TACTGTAAAG AACAACAGTT TCAATAGATA CAACTAATGA   
  
  
- CACCCATCAC CGATGTTTAT GTTATCGTTT AAGGTTTAAT AAATTTTGTG TTATAAACTA AACCTTTTGT   
  
  
- TAACTAAATT TCTCCTTGAA ACAATGTTTA TATTTTCAAT AATCGTAAAA GCCAAGTTTT ACGTATTCTA   
  
  
- CTCAACACAA AAAAAACAGC ACATGTGTAG TTTTTGACAG TATGCCTGTT ATTCGATCAG CGAAACTGGG   
  
  
- TAAGTGGCAT ACAGGGTTAC AGGAACTGGG ACGGTTTACT ATTCTTGCGG TCGTGTTTCC AAAATATTCA   
  
  
- AAGAGTTTCT CTTTGTTGTG TTACCCTTTT GTGTGGTAAC GAACTGAATG TTTACCTCTA GCCAACCTAG   
  
  
- TTAGAGACCT TTGTCCAACT TCATCTTAGA TTCGGGCAGA CCCGAGTACA GAGATGTGTC GTCTGGACTA   
  
  
- GTTTTGTTTA CCCTCTTACT CTTAGTGTAT TGAGACTTTC TCAGATAGAA AGATGCTCCA CCTAAGACAA   
  
  
- ATGGAAACCA GAGACGTCTA TCATCTACAA GGAACCGGTA CCGACCTCGC TAAAATTCGC AGTTTCGACG   
  
  
- TAGTGATCGT CACTGAGGTC AAGAATTTCA AAGCCAAAAT CAGTGTTCGC AAGA

+     Unnamed\_\_6

| Site Name | Organism | Position | Strand | Matrix score. | sequence | function |
| --- | --- | --- | --- | --- | --- | --- |
| Unnamed\_\_6 | Zea mays | 1268 | - | 10 | taTAAATATct |  |

>PlantCARE\_8972   
+ GAACTGTTAA AGCCAATCAT TTAAAATAAA AAGTCAAACC GGTTTAAACC GGTTTTCAAT AAGTTAATAA   
  
  
+ CCGGACTCGG TGTTAAAGAG ATTAAATTTA ACGGTTTAAC TCAGTGAGAT CGGTTTCGCT TTCTCTCTGT   
  
  
+ CGTGTTTGAG AAGTTGAAAG AGAGGAGGAT GAGCGCTGAA GATTTCCAGA AGAAGGTTTC GATCAGAGAC   
  
  
+ TCCTCCGTCG CCGGAGAAAT GGAAATCGAA TGCGGCGGGT CTACTAGCTC CGCGGTTGGT TCGTCTCGAA   
  
  
+ CCTTGGTTTT GCTTCGGAGA CTGCTCGAGA TTCAGGAGCG TCGAGCACAG GCCTACGCCA AACTCAAAAG   
  
  
+ GTTCGATCTT TGGAGTGTTT ATGCTATTAG TTACTGTTTT TGATTCAGTT ACTTGGTGAT TGTTACTGAG   
  
  
+ GGATGTTGGA GTTAAGTAAC TGATAGTGTT GTTGAGGAAG CGATGTCTCA TAAAGTTTAG ATTTTTATCT   
  
  
+ GTTGATCTCT TGCTATGCTT TTGGAATTTG ATTAATCAGA GGGTTCTCAG AGTATGTGGA GACTAGTGGT   
  
  
+ GAAGCGCTTT ATCAGAAGCT CTGCAGCGAG ATAACTGCTG AGTTCAACGA GTGTTCCAAA CAAGTAACTG   
  
  
+ AGTTTTTTTT TCTTCTTTTG ATTGATTACC TTTATGATGT TGTTGAAGAT ACGGTGTTAA AGATTGCGTC   
  
  
+ TTTGTTTGAT GGCAATGCAA GGTACGCGAA ATGGAAACTC TGTTTCTGAA TCCTGACGTT GGAAGATCGG   
  
  
+ ATCTTGCTCA ACTGCTCAGT GACATTCAAA CTCAGGAGAA GCAGAAACTG CATCTGGTCT GATTCCTATA   
  
  
+ TACTCAATTG TTGAACATAA CTGTTAACTT GTTGGACAGA ACAAAAGTTT CTTACTTTAG TTAGTTTTTT   
  
  
+ TTTTTTTTTG CAGACGGTTA CAATACAGGT ACTGAAGAAG GCAGGGAGGC CGTCAGAACG AATGCTGACA   
  
  
+ CACGAGAACT GCAAGTTCAA GAAACCGATG CAGCACGAGT GTGTGCATCT TCATGAGATT ACAGAAGCTG   
  
  
+ AAGGAACAGA GGAAGCAGAG GCGGATGCAG AGTTTGACAA CGCTTTGAAG GAAGCAATCA GAGGAGTGCA   
  
  
+ AGACGCTGTG ACTTGCATCA ATGAGTATTT GGAAGACGTT AGGTACGAGA TTGCAGCTCT TGAAGCTGAT   
  
  
+ TAGTTGTTCT CAATCTCATC ACTTCTTTAC CCCTTTCGAC TGTTAACTTG CTATGTTGCT AATTAGCTTA   
  
  
+ AGATATAAGA TATTTAGAAA CTCAAAAGTT ATGACATTTC TTGTTGTCAA AGTTATCTAT GTTGATTACT   
  
  
+ GTGGGTAGTG GCTACAAATA CAATAGCAAA TTCCAAATTA TTTAAAACAC AATATTTGAT TTGGAAAACA   
  
  
+ ATTGATTTAA AGAGGAACTT TGTTACAAAT ATAAAAGTTA TTAGCATTTT CGGTTCAAAA TGCATAAGAT   
  
  
+ GAGTTGTGTT TTTTTTGTCG TGTACACATC AAAAACTGTC ATACGGACAA TAAGCTAGTC GCTTTGACCC   
  
  
+ ATTCACCGTA TGTCCCAATG TCCTTGACCC TGCCAAATGA TAAGAACGCC AGCACAAAGG TTTTATAAGT   
  
  
+ TTCTCAAAGA GAAACAACAC AATGGGAAAA CACACCATTG CTTGACTTAC AAATGGAGAT CGGTTGGATC   
  
  
+ AATCTCTGGA AACAGGTTGA AGTAGAATCT AAGCCCGTCT GGGCTCATGT CTCTACACAG CAGACCTGAT   
  
  
+ CAAAACAAAT GGGAGAATGA GAATCACATA ACTCTGAAAG AGTCTATCTT TCTACGAGGT GGATTCTGTT   
  
  
+ TACCTTTGGT CTCTGCAGAT AGTAGATGTT CCTTGGCCAT GGCTGGAGCG ATTTTAAGCG TCAAAGCTGC   
  
  
+ ATCACTAGCA GTGACTCCAG TTCTTAAAGT TTCGGTTTTA GTCACAAGCG TTCT  

- CTTGACAATT TCGGTTAGTA AATTTTATTT TTCAGTTTGG CCAAATTTGG CCAAAAGTTA TTCAATTATT   
  
  
- GGCCTGAGCC ACAATTTCTC TAATTTAAAT TGCCAAATTG AGTCACTCTA GCCAAAGCGA AAGAGAGACA   
  
  
- GCACAAACTC TTCAACTTTC TCTCCTCCTA CTCGCGACTT CTAAAGGTCT TCTTCCAAAG CTAGTCTCTG   
  
  
- AGGAGGCAGC GGCCTCTTTA CCTTTAGCTT ACGCCGCCCA GATGATCGAG GCGCCAACCA AGCAGAGCTT   
  
  
- GGAACCAAAA CGAAGCCTCT GACGAGCTCT AAGTCCTCGC AGCTCGTGTC CGGATGCGGT TTGAGTTTTC   
  
  
- CAAGCTAGAA ACCTCACAAA TACGATAATC AATGACAAAA ACTAAGTCAA TGAACCACTA ACAATGACTC   
  
  
- CCTACAACCT CAATTCATTG ACTATCACAA CAACTCCTTC GCTACAGAGT ATTTCAAATC TAAAAATAGA   
  
  
- CAACTAGAGA ACGATACGAA AACCTTAAAC TAATTAGTCT CCCAAGAGTC TCATACACCT CTGATCACCA   
  
  
- CTTCGCGAAA TAGTCTTCGA GACGTCGCTC TATTGACGAC TCAAGTTGCT CACAAGGTTT GTTCATTGAC   
  
  
- TCAAAAAAAA AGAAGAAAAC TAACTAATGG AAATACTACA ACAACTTCTA TGCCACAATT TCTAACGCAG   
  
  
- AAACAAACTA CCGTTACGTT CCATGCGCTT TACCTTTGAG ACAAAGACTT AGGACTGCAA CCTTCTAGCC   
  
  
- TAGAACGAGT TGACGAGTCA CTGTAAGTTT GAGTCCTCTT CGTCTTTGAC GTAGACCAGA CTAAGGATAT   
  
  
- ATGAGTTAAC AACTTGTATT GACAATTGAA CAACCTGTCT TGTTTTCAAA GAATGAAATC AATCAAAAAA   
  
  
- AAAAAAAAAC GTCTGCCAAT GTTATGTCCA TGACTTCTTC CGTCCCTCCG GCAGTCTTGC TTACGACTGT   
  
  
- GTGCTCTTGA CGTTCAAGTT CTTTGGCTAC GTCGTGCTCA CACACGTAGA AGTACTCTAA TGTCTTCGAC   
  
  
- TTCCTTGTCT CCTTCGTCTC CGCCTACGTC TCAAACTGTT GCGAAACTTC CTTCGTTAGT CTCCTCACGT   
  
  
- TCTGCGACAC TGAACGTAGT TACTCATAAA CCTTCTGCAA TCCATGCTCT AACGTCGAGA ACTTCGACTA   
  
  
- ATCAACAAGA GTTAGAGTAG TGAAGAAATG GGGAAAGCTG ACAATTGAAC GATACAACGA TTAATCGAAT   
  
  
- TCTATATTCT ATAAATCTTT GAGTTTTCAA TACTGTAAAG AACAACAGTT TCAATAGATA CAACTAATGA   
  
  
- CACCCATCAC CGATGTTTAT GTTATCGTTT AAGGTTTAAT AAATTTTGTG TTATAAACTA AACCTTTTGT   
  
  
- TAACTAAATT TCTCCTTGAA ACAATGTTTA TATTTTCAAT AATCGTAAAA GCCAAGTTTT ACGTATTCTA   
  
  
- CTCAACACAA AAAAAACAGC ACATGTGTAG TTTTTGACAG TATGCCTGTT ATTCGATCAG CGAAACTGGG   
  
  
- TAAGTGGCAT ACAGGGTTAC AGGAACTGGG ACGGTTTACT ATTCTTGCGG TCGTGTTTCC AAAATATTCA   
  
  
- AAGAGTTTCT CTTTGTTGTG TTACCCTTTT GTGTGGTAAC GAACTGAATG TTTACCTCTA GCCAACCTAG   
  
  
- TTAGAGACCT TTGTCCAACT TCATCTTAGA TTCGGGCAGA CCCGAGTACA GAGATGTGTC GTCTGGACTA   
  
  
- GTTTTGTTTA CCCTCTTACT CTTAGTGTAT TGAGACTTTC TCAGATAGAA AGATGCTCCA CCTAAGACAA   
  
  
- ATGGAAACCA GAGACGTCTA TCATCTACAA GGAACCGGTA CCGACCTCGC TAAAATTCGC AGTTTCGACG   
  
  
- TAGTGATCGT CACTGAGGTC AAGAATTTCA AAGCCAAAAT CAGTGTTCGC AAGA

+     W box

| Site Name | Organism | Position | Strand | Matrix score. | sequence | function |
| --- | --- | --- | --- | --- | --- | --- |
| W box | Arabidopsis thaliana | 1534 | + | 6 | TTGACC |  |
| W box | Arabidopsis thaliana | 1564 | + | 6 | TTGACC |  |

>PlantCARE\_8972   
+ GAACTGTTAA AGCCAATCAT TTAAAATAAA AAGTCAAACC GGTTTAAACC GGTTTTCAAT AAGTTAATAA   
  
  
+ CCGGACTCGG TGTTAAAGAG ATTAAATTTA ACGGTTTAAC TCAGTGAGAT CGGTTTCGCT TTCTCTCTGT   
  
  
+ CGTGTTTGAG AAGTTGAAAG AGAGGAGGAT GAGCGCTGAA GATTTCCAGA AGAAGGTTTC GATCAGAGAC   
  
  
+ TCCTCCGTCG CCGGAGAAAT GGAAATCGAA TGCGGCGGGT CTACTAGCTC CGCGGTTGGT TCGTCTCGAA   
  
  
+ CCTTGGTTTT GCTTCGGAGA CTGCTCGAGA TTCAGGAGCG TCGAGCACAG GCCTACGCCA AACTCAAAAG   
  
  
+ GTTCGATCTT TGGAGTGTTT ATGCTATTAG TTACTGTTTT TGATTCAGTT ACTTGGTGAT TGTTACTGAG   
  
  
+ GGATGTTGGA GTTAAGTAAC TGATAGTGTT GTTGAGGAAG CGATGTCTCA TAAAGTTTAG ATTTTTATCT   
  
  
+ GTTGATCTCT TGCTATGCTT TTGGAATTTG ATTAATCAGA GGGTTCTCAG AGTATGTGGA GACTAGTGGT   
  
  
+ GAAGCGCTTT ATCAGAAGCT CTGCAGCGAG ATAACTGCTG AGTTCAACGA GTGTTCCAAA CAAGTAACTG   
  
  
+ AGTTTTTTTT TCTTCTTTTG ATTGATTACC TTTATGATGT TGTTGAAGAT ACGGTGTTAA AGATTGCGTC   
  
  
+ TTTGTTTGAT GGCAATGCAA GGTACGCGAA ATGGAAACTC TGTTTCTGAA TCCTGACGTT GGAAGATCGG   
  
  
+ ATCTTGCTCA ACTGCTCAGT GACATTCAAA CTCAGGAGAA GCAGAAACTG CATCTGGTCT GATTCCTATA   
  
  
+ TACTCAATTG TTGAACATAA CTGTTAACTT GTTGGACAGA ACAAAAGTTT CTTACTTTAG TTAGTTTTTT   
  
  
+ TTTTTTTTTG CAGACGGTTA CAATACAGGT ACTGAAGAAG GCAGGGAGGC CGTCAGAACG AATGCTGACA   
  
  
+ CACGAGAACT GCAAGTTCAA GAAACCGATG CAGCACGAGT GTGTGCATCT TCATGAGATT ACAGAAGCTG   
  
  
+ AAGGAACAGA GGAAGCAGAG GCGGATGCAG AGTTTGACAA CGCTTTGAAG GAAGCAATCA GAGGAGTGCA   
  
  
+ AGACGCTGTG ACTTGCATCA ATGAGTATTT GGAAGACGTT AGGTACGAGA TTGCAGCTCT TGAAGCTGAT   
  
  
+ TAGTTGTTCT CAATCTCATC ACTTCTTTAC CCCTTTCGAC TGTTAACTTG CTATGTTGCT AATTAGCTTA   
  
  
+ AGATATAAGA TATTTAGAAA CTCAAAAGTT ATGACATTTC TTGTTGTCAA AGTTATCTAT GTTGATTACT   
  
  
+ GTGGGTAGTG GCTACAAATA CAATAGCAAA TTCCAAATTA TTTAAAACAC AATATTTGAT TTGGAAAACA   
  
  
+ ATTGATTTAA AGAGGAACTT TGTTACAAAT ATAAAAGTTA TTAGCATTTT CGGTTCAAAA TGCATAAGAT   
  
  
+ GAGTTGTGTT TTTTTTGTCG TGTACACATC AAAAACTGTC ATACGGACAA TAAGCTAGTC GCTTTGACCC   
  
  
+ ATTCACCGTA TGTCCCAATG TCCTTGACCC TGCCAAATGA TAAGAACGCC AGCACAAAGG TTTTATAAGT   
  
  
+ TTCTCAAAGA GAAACAACAC AATGGGAAAA CACACCATTG CTTGACTTAC AAATGGAGAT CGGTTGGATC   
  
  
+ AATCTCTGGA AACAGGTTGA AGTAGAATCT AAGCCCGTCT GGGCTCATGT CTCTACACAG CAGACCTGAT   
  
  
+ CAAAACAAAT GGGAGAATGA GAATCACATA ACTCTGAAAG AGTCTATCTT TCTACGAGGT GGATTCTGTT   
  
  
+ TACCTTTGGT CTCTGCAGAT AGTAGATGTT CCTTGGCCAT GGCTGGAGCG ATTTTAAGCG TCAAAGCTGC   
  
  
+ ATCACTAGCA GTGACTCCAG TTCTTAAAGT TTCGGTTTTA GTCACAAGCG TTCT  

- CTTGACAATT TCGGTTAGTA AATTTTATTT TTCAGTTTGG CCAAATTTGG CCAAAAGTTA TTCAATTATT   
  
  
- GGCCTGAGCC ACAATTTCTC TAATTTAAAT TGCCAAATTG AGTCACTCTA GCCAAAGCGA AAGAGAGACA   
  
  
- GCACAAACTC TTCAACTTTC TCTCCTCCTA CTCGCGACTT CTAAAGGTCT TCTTCCAAAG CTAGTCTCTG   
  
  
- AGGAGGCAGC GGCCTCTTTA CCTTTAGCTT ACGCCGCCCA GATGATCGAG GCGCCAACCA AGCAGAGCTT   
  
  
- GGAACCAAAA CGAAGCCTCT GACGAGCTCT AAGTCCTCGC AGCTCGTGTC CGGATGCGGT TTGAGTTTTC   
  
  
- CAAGCTAGAA ACCTCACAAA TACGATAATC AATGACAAAA ACTAAGTCAA TGAACCACTA ACAATGACTC   
  
  
- CCTACAACCT CAATTCATTG ACTATCACAA CAACTCCTTC GCTACAGAGT ATTTCAAATC TAAAAATAGA   
  
  
- CAACTAGAGA ACGATACGAA AACCTTAAAC TAATTAGTCT CCCAAGAGTC TCATACACCT CTGATCACCA   
  
  
- CTTCGCGAAA TAGTCTTCGA GACGTCGCTC TATTGACGAC TCAAGTTGCT CACAAGGTTT GTTCATTGAC   
  
  
- TCAAAAAAAA AGAAGAAAAC TAACTAATGG AAATACTACA ACAACTTCTA TGCCACAATT TCTAACGCAG   
  
  
- AAACAAACTA CCGTTACGTT CCATGCGCTT TACCTTTGAG ACAAAGACTT AGGACTGCAA CCTTCTAGCC   
  
  
- TAGAACGAGT TGACGAGTCA CTGTAAGTTT GAGTCCTCTT CGTCTTTGAC GTAGACCAGA CTAAGGATAT   
  
  
- ATGAGTTAAC AACTTGTATT GACAATTGAA CAACCTGTCT TGTTTTCAAA GAATGAAATC AATCAAAAAA   
  
  
- AAAAAAAAAC GTCTGCCAAT GTTATGTCCA TGACTTCTTC CGTCCCTCCG GCAGTCTTGC TTACGACTGT   
  
  
- GTGCTCTTGA CGTTCAAGTT CTTTGGCTAC GTCGTGCTCA CACACGTAGA AGTACTCTAA TGTCTTCGAC   
  
  
- TTCCTTGTCT CCTTCGTCTC CGCCTACGTC TCAAACTGTT GCGAAACTTC CTTCGTTAGT CTCCTCACGT   
  
  
- TCTGCGACAC TGAACGTAGT TACTCATAAA CCTTCTGCAA TCCATGCTCT AACGTCGAGA ACTTCGACTA   
  
  
- ATCAACAAGA GTTAGAGTAG TGAAGAAATG GGGAAAGCTG ACAATTGAAC GATACAACGA TTAATCGAAT   
  
  
- TCTATATTCT ATAAATCTTT GAGTTTTCAA TACTGTAAAG AACAACAGTT TCAATAGATA CAACTAATGA   
  
  
- CACCCATCAC CGATGTTTAT GTTATCGTTT AAGGTTTAAT AAATTTTGTG TTATAAACTA AACCTTTTGT   
  
  
- TAACTAAATT TCTCCTTGAA ACAATGTTTA TATTTTCAAT AATCGTAAAA GCCAAGTTTT ACGTATTCTA   
  
  
- CTCAACACAA AAAAAACAGC ACATGTGTAG TTTTTGACAG TATGCCTGTT ATTCGATCAG CGAAACTGGG   
  
  
- TAAGTGGCAT ACAGGGTTAC AGGAACTGGG ACGGTTTACT ATTCTTGCGG TCGTGTTTCC AAAATATTCA   
  
  
- AAGAGTTTCT CTTTGTTGTG TTACCCTTTT GTGTGGTAAC GAACTGAATG TTTACCTCTA GCCAACCTAG   
  
  
- TTAGAGACCT TTGTCCAACT TCATCTTAGA TTCGGGCAGA CCCGAGTACA GAGATGTGTC GTCTGGACTA   
  
  
- GTTTTGTTTA CCCTCTTACT CTTAGTGTAT TGAGACTTTC TCAGATAGAA AGATGCTCCA CCTAAGACAA   
  
  
- ATGGAAACCA GAGACGTCTA TCATCTACAA GGAACCGGTA CCGACCTCGC TAAAATTCGC AGTTTCGACG   
  
  
- TAGTGATCGT CACTGAGGTC AAGAATTTCA AAGCCAAAAT CAGTGTTCGC AAGA

+     WRE3

| Site Name | Organism | Position | Strand | Matrix score. | sequence | function |
| --- | --- | --- | --- | --- | --- | --- |
| WRE3 | Pisum sativum | 1807 | - | 6 | CCACCT |  |

>PlantCARE\_8972   
+ GAACTGTTAA AGCCAATCAT TTAAAATAAA AAGTCAAACC GGTTTAAACC GGTTTTCAAT AAGTTAATAA   
  
  
+ CCGGACTCGG TGTTAAAGAG ATTAAATTTA ACGGTTTAAC TCAGTGAGAT CGGTTTCGCT TTCTCTCTGT   
  
  
+ CGTGTTTGAG AAGTTGAAAG AGAGGAGGAT GAGCGCTGAA GATTTCCAGA AGAAGGTTTC GATCAGAGAC   
  
  
+ TCCTCCGTCG CCGGAGAAAT GGAAATCGAA TGCGGCGGGT CTACTAGCTC CGCGGTTGGT TCGTCTCGAA   
  
  
+ CCTTGGTTTT GCTTCGGAGA CTGCTCGAGA TTCAGGAGCG TCGAGCACAG GCCTACGCCA AACTCAAAAG   
  
  
+ GTTCGATCTT TGGAGTGTTT ATGCTATTAG TTACTGTTTT TGATTCAGTT ACTTGGTGAT TGTTACTGAG   
  
  
+ GGATGTTGGA GTTAAGTAAC TGATAGTGTT GTTGAGGAAG CGATGTCTCA TAAAGTTTAG ATTTTTATCT   
  
  
+ GTTGATCTCT TGCTATGCTT TTGGAATTTG ATTAATCAGA GGGTTCTCAG AGTATGTGGA GACTAGTGGT   
  
  
+ GAAGCGCTTT ATCAGAAGCT CTGCAGCGAG ATAACTGCTG AGTTCAACGA GTGTTCCAAA CAAGTAACTG   
  
  
+ AGTTTTTTTT TCTTCTTTTG ATTGATTACC TTTATGATGT TGTTGAAGAT ACGGTGTTAA AGATTGCGTC   
  
  
+ TTTGTTTGAT GGCAATGCAA GGTACGCGAA ATGGAAACTC TGTTTCTGAA TCCTGACGTT GGAAGATCGG   
  
  
+ ATCTTGCTCA ACTGCTCAGT GACATTCAAA CTCAGGAGAA GCAGAAACTG CATCTGGTCT GATTCCTATA   
  
  
+ TACTCAATTG TTGAACATAA CTGTTAACTT GTTGGACAGA ACAAAAGTTT CTTACTTTAG TTAGTTTTTT   
  
  
+ TTTTTTTTTG CAGACGGTTA CAATACAGGT ACTGAAGAAG GCAGGGAGGC CGTCAGAACG AATGCTGACA   
  
  
+ CACGAGAACT GCAAGTTCAA GAAACCGATG CAGCACGAGT GTGTGCATCT TCATGAGATT ACAGAAGCTG   
  
  
+ AAGGAACAGA GGAAGCAGAG GCGGATGCAG AGTTTGACAA CGCTTTGAAG GAAGCAATCA GAGGAGTGCA   
  
  
+ AGACGCTGTG ACTTGCATCA ATGAGTATTT GGAAGACGTT AGGTACGAGA TTGCAGCTCT TGAAGCTGAT   
  
  
+ TAGTTGTTCT CAATCTCATC ACTTCTTTAC CCCTTTCGAC TGTTAACTTG CTATGTTGCT AATTAGCTTA   
  
  
+ AGATATAAGA TATTTAGAAA CTCAAAAGTT ATGACATTTC TTGTTGTCAA AGTTATCTAT GTTGATTACT   
  
  
+ GTGGGTAGTG GCTACAAATA CAATAGCAAA TTCCAAATTA TTTAAAACAC AATATTTGAT TTGGAAAACA   
  
  
+ ATTGATTTAA AGAGGAACTT TGTTACAAAT ATAAAAGTTA TTAGCATTTT CGGTTCAAAA TGCATAAGAT   
  
  
+ GAGTTGTGTT TTTTTTGTCG TGTACACATC AAAAACTGTC ATACGGACAA TAAGCTAGTC GCTTTGACCC   
  
  
+ ATTCACCGTA TGTCCCAATG TCCTTGACCC TGCCAAATGA TAAGAACGCC AGCACAAAGG TTTTATAAGT   
  
  
+ TTCTCAAAGA GAAACAACAC AATGGGAAAA CACACCATTG CTTGACTTAC AAATGGAGAT CGGTTGGATC   
  
  
+ AATCTCTGGA AACAGGTTGA AGTAGAATCT AAGCCCGTCT GGGCTCATGT CTCTACACAG CAGACCTGAT   
  
  
+ CAAAACAAAT GGGAGAATGA GAATCACATA ACTCTGAAAG AGTCTATCTT TCTACGAGGT GGATTCTGTT   
  
  
+ TACCTTTGGT CTCTGCAGAT AGTAGATGTT CCTTGGCCAT GGCTGGAGCG ATTTTAAGCG TCAAAGCTGC   
  
  
+ ATCACTAGCA GTGACTCCAG TTCTTAAAGT TTCGGTTTTA GTCACAAGCG TTCT  

- CTTGACAATT TCGGTTAGTA AATTTTATTT TTCAGTTTGG CCAAATTTGG CCAAAAGTTA TTCAATTATT   
  
  
- GGCCTGAGCC ACAATTTCTC TAATTTAAAT TGCCAAATTG AGTCACTCTA GCCAAAGCGA AAGAGAGACA   
  
  
- GCACAAACTC TTCAACTTTC TCTCCTCCTA CTCGCGACTT CTAAAGGTCT TCTTCCAAAG CTAGTCTCTG   
  
  
- AGGAGGCAGC GGCCTCTTTA CCTTTAGCTT ACGCCGCCCA GATGATCGAG GCGCCAACCA AGCAGAGCTT   
  
  
- GGAACCAAAA CGAAGCCTCT GACGAGCTCT AAGTCCTCGC AGCTCGTGTC CGGATGCGGT TTGAGTTTTC   
  
  
- CAAGCTAGAA ACCTCACAAA TACGATAATC AATGACAAAA ACTAAGTCAA TGAACCACTA ACAATGACTC   
  
  
- CCTACAACCT CAATTCATTG ACTATCACAA CAACTCCTTC GCTACAGAGT ATTTCAAATC TAAAAATAGA   
  
  
- CAACTAGAGA ACGATACGAA AACCTTAAAC TAATTAGTCT CCCAAGAGTC TCATACACCT CTGATCACCA   
  
  
- CTTCGCGAAA TAGTCTTCGA GACGTCGCTC TATTGACGAC TCAAGTTGCT CACAAGGTTT GTTCATTGAC   
  
  
- TCAAAAAAAA AGAAGAAAAC TAACTAATGG AAATACTACA ACAACTTCTA TGCCACAATT TCTAACGCAG   
  
  
- AAACAAACTA CCGTTACGTT CCATGCGCTT TACCTTTGAG ACAAAGACTT AGGACTGCAA CCTTCTAGCC   
  
  
- TAGAACGAGT TGACGAGTCA CTGTAAGTTT GAGTCCTCTT CGTCTTTGAC GTAGACCAGA CTAAGGATAT   
  
  
- ATGAGTTAAC AACTTGTATT GACAATTGAA CAACCTGTCT TGTTTTCAAA GAATGAAATC AATCAAAAAA   
  
  
- AAAAAAAAAC GTCTGCCAAT GTTATGTCCA TGACTTCTTC CGTCCCTCCG GCAGTCTTGC TTACGACTGT   
  
  
- GTGCTCTTGA CGTTCAAGTT CTTTGGCTAC GTCGTGCTCA CACACGTAGA AGTACTCTAA TGTCTTCGAC   
  
  
- TTCCTTGTCT CCTTCGTCTC CGCCTACGTC TCAAACTGTT GCGAAACTTC CTTCGTTAGT CTCCTCACGT   
  
  
- TCTGCGACAC TGAACGTAGT TACTCATAAA CCTTCTGCAA TCCATGCTCT AACGTCGAGA ACTTCGACTA   
  
  
- ATCAACAAGA GTTAGAGTAG TGAAGAAATG GGGAAAGCTG ACAATTGAAC GATACAACGA TTAATCGAAT   
  
  
- TCTATATTCT ATAAATCTTT GAGTTTTCAA TACTGTAAAG AACAACAGTT TCAATAGATA CAACTAATGA   
  
  
- CACCCATCAC CGATGTTTAT GTTATCGTTT AAGGTTTAAT AAATTTTGTG TTATAAACTA AACCTTTTGT   
  
  
- TAACTAAATT TCTCCTTGAA ACAATGTTTA TATTTTCAAT AATCGTAAAA GCCAAGTTTT ACGTATTCTA   
  
  
- CTCAACACAA AAAAAACAGC ACATGTGTAG TTTTTGACAG TATGCCTGTT ATTCGATCAG CGAAACTGGG   
  
  
- TAAGTGGCAT ACAGGGTTAC AGGAACTGGG ACGGTTTACT ATTCTTGCGG TCGTGTTTCC AAAATATTCA   
  
  
- AAGAGTTTCT CTTTGTTGTG TTACCCTTTT GTGTGGTAAC GAACTGAATG TTTACCTCTA GCCAACCTAG   
  
  
- TTAGAGACCT TTGTCCAACT TCATCTTAGA TTCGGGCAGA CCCGAGTACA GAGATGTGTC GTCTGGACTA   
  
  
- GTTTTGTTTA CCCTCTTACT CTTAGTGTAT TGAGACTTTC TCAGATAGAA AGATGCTCCA CCTAAGACAA   
  
  
- ATGGAAACCA GAGACGTCTA TCATCTACAA GGAACCGGTA CCGACCTCGC TAAAATTCGC AGTTTCGACG   
  
  
- TAGTGATCGT CACTGAGGTC AAGAATTTCA AAGCCAAAAT CAGTGTTCGC AAGA

+     as-1

| Site Name | Organism | Position | Strand | Matrix score. | sequence | function |
| --- | --- | --- | --- | --- | --- | --- |
| as-1 | Arabidopsis thaliana | 754 | + | 5 | TGACG |  |
| as-1 | Arabidopsis thaliana | 1879 | - | 5 | TGACG |  |
| as-1 | Arabidopsis thaliana | 961 | - | 5 | TGACG |  |

>PlantCARE\_8972   
+ GAACTGTTAA AGCCAATCAT TTAAAATAAA AAGTCAAACC GGTTTAAACC GGTTTTCAAT AAGTTAATAA   
  
  
+ CCGGACTCGG TGTTAAAGAG ATTAAATTTA ACGGTTTAAC TCAGTGAGAT CGGTTTCGCT TTCTCTCTGT   
  
  
+ CGTGTTTGAG AAGTTGAAAG AGAGGAGGAT GAGCGCTGAA GATTTCCAGA AGAAGGTTTC GATCAGAGAC   
  
  
+ TCCTCCGTCG CCGGAGAAAT GGAAATCGAA TGCGGCGGGT CTACTAGCTC CGCGGTTGGT TCGTCTCGAA   
  
  
+ CCTTGGTTTT GCTTCGGAGA CTGCTCGAGA TTCAGGAGCG TCGAGCACAG GCCTACGCCA AACTCAAAAG   
  
  
+ GTTCGATCTT TGGAGTGTTT ATGCTATTAG TTACTGTTTT TGATTCAGTT ACTTGGTGAT TGTTACTGAG   
  
  
+ GGATGTTGGA GTTAAGTAAC TGATAGTGTT GTTGAGGAAG CGATGTCTCA TAAAGTTTAG ATTTTTATCT   
  
  
+ GTTGATCTCT TGCTATGCTT TTGGAATTTG ATTAATCAGA GGGTTCTCAG AGTATGTGGA GACTAGTGGT   
  
  
+ GAAGCGCTTT ATCAGAAGCT CTGCAGCGAG ATAACTGCTG AGTTCAACGA GTGTTCCAAA CAAGTAACTG   
  
  
+ AGTTTTTTTT TCTTCTTTTG ATTGATTACC TTTATGATGT TGTTGAAGAT ACGGTGTTAA AGATTGCGTC   
  
  
+ TTTGTTTGAT GGCAATGCAA GGTACGCGAA ATGGAAACTC TGTTTCTGAA TCCTGACGTT GGAAGATCGG   
  
  
+ ATCTTGCTCA ACTGCTCAGT GACATTCAAA CTCAGGAGAA GCAGAAACTG CATCTGGTCT GATTCCTATA   
  
  
+ TACTCAATTG TTGAACATAA CTGTTAACTT GTTGGACAGA ACAAAAGTTT CTTACTTTAG TTAGTTTTTT   
  
  
+ TTTTTTTTTG CAGACGGTTA CAATACAGGT ACTGAAGAAG GCAGGGAGGC CGTCAGAACG AATGCTGACA   
  
  
+ CACGAGAACT GCAAGTTCAA GAAACCGATG CAGCACGAGT GTGTGCATCT TCATGAGATT ACAGAAGCTG   
  
  
+ AAGGAACAGA GGAAGCAGAG GCGGATGCAG AGTTTGACAA CGCTTTGAAG GAAGCAATCA GAGGAGTGCA   
  
  
+ AGACGCTGTG ACTTGCATCA ATGAGTATTT GGAAGACGTT AGGTACGAGA TTGCAGCTCT TGAAGCTGAT   
  
  
+ TAGTTGTTCT CAATCTCATC ACTTCTTTAC CCCTTTCGAC TGTTAACTTG CTATGTTGCT AATTAGCTTA   
  
  
+ AGATATAAGA TATTTAGAAA CTCAAAAGTT ATGACATTTC TTGTTGTCAA AGTTATCTAT GTTGATTACT   
  
  
+ GTGGGTAGTG GCTACAAATA CAATAGCAAA TTCCAAATTA TTTAAAACAC AATATTTGAT TTGGAAAACA   
  
  
+ ATTGATTTAA AGAGGAACTT TGTTACAAAT ATAAAAGTTA TTAGCATTTT CGGTTCAAAA TGCATAAGAT   
  
  
+ GAGTTGTGTT TTTTTTGTCG TGTACACATC AAAAACTGTC ATACGGACAA TAAGCTAGTC GCTTTGACCC   
  
  
+ ATTCACCGTA TGTCCCAATG TCCTTGACCC TGCCAAATGA TAAGAACGCC AGCACAAAGG TTTTATAAGT   
  
  
+ TTCTCAAAGA GAAACAACAC AATGGGAAAA CACACCATTG CTTGACTTAC AAATGGAGAT CGGTTGGATC   
  
  
+ AATCTCTGGA AACAGGTTGA AGTAGAATCT AAGCCCGTCT GGGCTCATGT CTCTACACAG CAGACCTGAT   
  
  
+ CAAAACAAAT GGGAGAATGA GAATCACATA ACTCTGAAAG AGTCTATCTT TCTACGAGGT GGATTCTGTT   
  
  
+ TACCTTTGGT CTCTGCAGAT AGTAGATGTT CCTTGGCCAT GGCTGGAGCG ATTTTAAGCG TCAAAGCTGC   
  
  
+ ATCACTAGCA GTGACTCCAG TTCTTAAAGT TTCGGTTTTA GTCACAAGCG TTCT  

- CTTGACAATT TCGGTTAGTA AATTTTATTT TTCAGTTTGG CCAAATTTGG CCAAAAGTTA TTCAATTATT   
  
  
- GGCCTGAGCC ACAATTTCTC TAATTTAAAT TGCCAAATTG AGTCACTCTA GCCAAAGCGA AAGAGAGACA   
  
  
- GCACAAACTC TTCAACTTTC TCTCCTCCTA CTCGCGACTT CTAAAGGTCT TCTTCCAAAG CTAGTCTCTG   
  
  
- AGGAGGCAGC GGCCTCTTTA CCTTTAGCTT ACGCCGCCCA GATGATCGAG GCGCCAACCA AGCAGAGCTT   
  
  
- GGAACCAAAA CGAAGCCTCT GACGAGCTCT AAGTCCTCGC AGCTCGTGTC CGGATGCGGT TTGAGTTTTC   
  
  
- CAAGCTAGAA ACCTCACAAA TACGATAATC AATGACAAAA ACTAAGTCAA TGAACCACTA ACAATGACTC   
  
  
- CCTACAACCT CAATTCATTG ACTATCACAA CAACTCCTTC GCTACAGAGT ATTTCAAATC TAAAAATAGA   
  
  
- CAACTAGAGA ACGATACGAA AACCTTAAAC TAATTAGTCT CCCAAGAGTC TCATACACCT CTGATCACCA   
  
  
- CTTCGCGAAA TAGTCTTCGA GACGTCGCTC TATTGACGAC TCAAGTTGCT CACAAGGTTT GTTCATTGAC   
  
  
- TCAAAAAAAA AGAAGAAAAC TAACTAATGG AAATACTACA ACAACTTCTA TGCCACAATT TCTAACGCAG   
  
  
- AAACAAACTA CCGTTACGTT CCATGCGCTT TACCTTTGAG ACAAAGACTT AGGACTGCAA CCTTCTAGCC   
  
  
- TAGAACGAGT TGACGAGTCA CTGTAAGTTT GAGTCCTCTT CGTCTTTGAC GTAGACCAGA CTAAGGATAT   
  
  
- ATGAGTTAAC AACTTGTATT GACAATTGAA CAACCTGTCT TGTTTTCAAA GAATGAAATC AATCAAAAAA   
  
  
- AAAAAAAAAC GTCTGCCAAT GTTATGTCCA TGACTTCTTC CGTCCCTCCG GCAGTCTTGC TTACGACTGT   
  
  
- GTGCTCTTGA CGTTCAAGTT CTTTGGCTAC GTCGTGCTCA CACACGTAGA AGTACTCTAA TGTCTTCGAC   
  
  
- TTCCTTGTCT CCTTCGTCTC CGCCTACGTC TCAAACTGTT GCGAAACTTC CTTCGTTAGT CTCCTCACGT   
  
  
- TCTGCGACAC TGAACGTAGT TACTCATAAA CCTTCTGCAA TCCATGCTCT AACGTCGAGA ACTTCGACTA   
  
  
- ATCAACAAGA GTTAGAGTAG TGAAGAAATG GGGAAAGCTG ACAATTGAAC GATACAACGA TTAATCGAAT   
  
  
- TCTATATTCT ATAAATCTTT GAGTTTTCAA TACTGTAAAG AACAACAGTT TCAATAGATA CAACTAATGA   
  
  
- CACCCATCAC CGATGTTTAT GTTATCGTTT AAGGTTTAAT AAATTTTGTG TTATAAACTA AACCTTTTGT   
  
  
- TAACTAAATT TCTCCTTGAA ACAATGTTTA TATTTTCAAT AATCGTAAAA GCCAAGTTTT ACGTATTCTA   
  
  
- CTCAACACAA AAAAAACAGC ACATGTGTAG TTTTTGACAG TATGCCTGTT ATTCGATCAG CGAAACTGGG   
  
  
- TAAGTGGCAT ACAGGGTTAC AGGAACTGGG ACGGTTTACT ATTCTTGCGG TCGTGTTTCC AAAATATTCA   
  
  
- AAGAGTTTCT CTTTGTTGTG TTACCCTTTT GTGTGGTAAC GAACTGAATG TTTACCTCTA GCCAACCTAG   
  
  
- TTAGAGACCT TTGTCCAACT TCATCTTAGA TTCGGGCAGA CCCGAGTACA GAGATGTGTC GTCTGGACTA   
  
  
- GTTTTGTTTA CCCTCTTACT CTTAGTGTAT TGAGACTTTC TCAGATAGAA AGATGCTCCA CCTAAGACAA   
  
  
- ATGGAAACCA GAGACGTCTA TCATCTACAA GGAACCGGTA CCGACCTCGC TAAAATTCGC AGTTTCGACG   
  
  
- TAGTGATCGT CACTGAGGTC AAGAATTTCA AAGCCAAAAT CAGTGTTCGC AAGA

+     chs-CMA1a

| Site Name | Organism | Position | Strand | Matrix score. | sequence | function |
| --- | --- | --- | --- | --- | --- | --- |
| chs-CMA1a | Daucus carota | 432 | - | 8 | TTACTTAA | part of a light responsive element |

>PlantCARE\_8972   
+ GAACTGTTAA AGCCAATCAT TTAAAATAAA AAGTCAAACC GGTTTAAACC GGTTTTCAAT AAGTTAATAA   
  
  
+ CCGGACTCGG TGTTAAAGAG ATTAAATTTA ACGGTTTAAC TCAGTGAGAT CGGTTTCGCT TTCTCTCTGT   
  
  
+ CGTGTTTGAG AAGTTGAAAG AGAGGAGGAT GAGCGCTGAA GATTTCCAGA AGAAGGTTTC GATCAGAGAC   
  
  
+ TCCTCCGTCG CCGGAGAAAT GGAAATCGAA TGCGGCGGGT CTACTAGCTC CGCGGTTGGT TCGTCTCGAA   
  
  
+ CCTTGGTTTT GCTTCGGAGA CTGCTCGAGA TTCAGGAGCG TCGAGCACAG GCCTACGCCA AACTCAAAAG   
  
  
+ GTTCGATCTT TGGAGTGTTT ATGCTATTAG TTACTGTTTT TGATTCAGTT ACTTGGTGAT TGTTACTGAG   
  
  
+ GGATGTTGGA GTTAAGTAAC TGATAGTGTT GTTGAGGAAG CGATGTCTCA TAAAGTTTAG ATTTTTATCT   
  
  
+ GTTGATCTCT TGCTATGCTT TTGGAATTTG ATTAATCAGA GGGTTCTCAG AGTATGTGGA GACTAGTGGT   
  
  
+ GAAGCGCTTT ATCAGAAGCT CTGCAGCGAG ATAACTGCTG AGTTCAACGA GTGTTCCAAA CAAGTAACTG   
  
  
+ AGTTTTTTTT TCTTCTTTTG ATTGATTACC TTTATGATGT TGTTGAAGAT ACGGTGTTAA AGATTGCGTC   
  
  
+ TTTGTTTGAT GGCAATGCAA GGTACGCGAA ATGGAAACTC TGTTTCTGAA TCCTGACGTT GGAAGATCGG   
  
  
+ ATCTTGCTCA ACTGCTCAGT GACATTCAAA CTCAGGAGAA GCAGAAACTG CATCTGGTCT GATTCCTATA   
  
  
+ TACTCAATTG TTGAACATAA CTGTTAACTT GTTGGACAGA ACAAAAGTTT CTTACTTTAG TTAGTTTTTT   
  
  
+ TTTTTTTTTG CAGACGGTTA CAATACAGGT ACTGAAGAAG GCAGGGAGGC CGTCAGAACG AATGCTGACA   
  
  
+ CACGAGAACT GCAAGTTCAA GAAACCGATG CAGCACGAGT GTGTGCATCT TCATGAGATT ACAGAAGCTG   
  
  
+ AAGGAACAGA GGAAGCAGAG GCGGATGCAG AGTTTGACAA CGCTTTGAAG GAAGCAATCA GAGGAGTGCA   
  
  
+ AGACGCTGTG ACTTGCATCA ATGAGTATTT GGAAGACGTT AGGTACGAGA TTGCAGCTCT TGAAGCTGAT   
  
  
+ TAGTTGTTCT CAATCTCATC ACTTCTTTAC CCCTTTCGAC TGTTAACTTG CTATGTTGCT AATTAGCTTA   
  
  
+ AGATATAAGA TATTTAGAAA CTCAAAAGTT ATGACATTTC TTGTTGTCAA AGTTATCTAT GTTGATTACT   
  
  
+ GTGGGTAGTG GCTACAAATA CAATAGCAAA TTCCAAATTA TTTAAAACAC AATATTTGAT TTGGAAAACA   
  
  
+ ATTGATTTAA AGAGGAACTT TGTTACAAAT ATAAAAGTTA TTAGCATTTT CGGTTCAAAA TGCATAAGAT   
  
  
+ GAGTTGTGTT TTTTTTGTCG TGTACACATC AAAAACTGTC ATACGGACAA TAAGCTAGTC GCTTTGACCC   
  
  
+ ATTCACCGTA TGTCCCAATG TCCTTGACCC TGCCAAATGA TAAGAACGCC AGCACAAAGG TTTTATAAGT   
  
  
+ TTCTCAAAGA GAAACAACAC AATGGGAAAA CACACCATTG CTTGACTTAC AAATGGAGAT CGGTTGGATC   
  
  
+ AATCTCTGGA AACAGGTTGA AGTAGAATCT AAGCCCGTCT GGGCTCATGT CTCTACACAG CAGACCTGAT   
  
  
+ CAAAACAAAT GGGAGAATGA GAATCACATA ACTCTGAAAG AGTCTATCTT TCTACGAGGT GGATTCTGTT   
  
  
+ TACCTTTGGT CTCTGCAGAT AGTAGATGTT CCTTGGCCAT GGCTGGAGCG ATTTTAAGCG TCAAAGCTGC   
  
  
+ ATCACTAGCA GTGACTCCAG TTCTTAAAGT TTCGGTTTTA GTCACAAGCG TTCT  

- CTTGACAATT TCGGTTAGTA AATTTTATTT TTCAGTTTGG CCAAATTTGG CCAAAAGTTA TTCAATTATT   
  
  
- GGCCTGAGCC ACAATTTCTC TAATTTAAAT TGCCAAATTG AGTCACTCTA GCCAAAGCGA AAGAGAGACA   
  
  
- GCACAAACTC TTCAACTTTC TCTCCTCCTA CTCGCGACTT CTAAAGGTCT TCTTCCAAAG CTAGTCTCTG   
  
  
- AGGAGGCAGC GGCCTCTTTA CCTTTAGCTT ACGCCGCCCA GATGATCGAG GCGCCAACCA AGCAGAGCTT   
  
  
- GGAACCAAAA CGAAGCCTCT GACGAGCTCT AAGTCCTCGC AGCTCGTGTC CGGATGCGGT TTGAGTTTTC   
  
  
- CAAGCTAGAA ACCTCACAAA TACGATAATC AATGACAAAA ACTAAGTCAA TGAACCACTA ACAATGACTC   
  
  
- CCTACAACCT CAATTCATTG ACTATCACAA CAACTCCTTC GCTACAGAGT ATTTCAAATC TAAAAATAGA   
  
  
- CAACTAGAGA ACGATACGAA AACCTTAAAC TAATTAGTCT CCCAAGAGTC TCATACACCT CTGATCACCA   
  
  
- CTTCGCGAAA TAGTCTTCGA GACGTCGCTC TATTGACGAC TCAAGTTGCT CACAAGGTTT GTTCATTGAC   
  
  
- TCAAAAAAAA AGAAGAAAAC TAACTAATGG AAATACTACA ACAACTTCTA TGCCACAATT TCTAACGCAG   
  
  
- AAACAAACTA CCGTTACGTT CCATGCGCTT TACCTTTGAG ACAAAGACTT AGGACTGCAA CCTTCTAGCC   
  
  
- TAGAACGAGT TGACGAGTCA CTGTAAGTTT GAGTCCTCTT CGTCTTTGAC GTAGACCAGA CTAAGGATAT   
  
  
- ATGAGTTAAC AACTTGTATT GACAATTGAA CAACCTGTCT TGTTTTCAAA GAATGAAATC AATCAAAAAA   
  
  
- AAAAAAAAAC GTCTGCCAAT GTTATGTCCA TGACTTCTTC CGTCCCTCCG GCAGTCTTGC TTACGACTGT   
  
  
- GTGCTCTTGA CGTTCAAGTT CTTTGGCTAC GTCGTGCTCA CACACGTAGA AGTACTCTAA TGTCTTCGAC   
  
  
- TTCCTTGTCT CCTTCGTCTC CGCCTACGTC TCAAACTGTT GCGAAACTTC CTTCGTTAGT CTCCTCACGT   
  
  
- TCTGCGACAC TGAACGTAGT TACTCATAAA CCTTCTGCAA TCCATGCTCT AACGTCGAGA ACTTCGACTA   
  
  
- ATCAACAAGA GTTAGAGTAG TGAAGAAATG GGGAAAGCTG ACAATTGAAC GATACAACGA TTAATCGAAT   
  
  
- TCTATATTCT ATAAATCTTT GAGTTTTCAA TACTGTAAAG AACAACAGTT TCAATAGATA CAACTAATGA   
  
  
- CACCCATCAC CGATGTTTAT GTTATCGTTT AAGGTTTAAT AAATTTTGTG TTATAAACTA AACCTTTTGT   
  
  
- TAACTAAATT TCTCCTTGAA ACAATGTTTA TATTTTCAAT AATCGTAAAA GCCAAGTTTT ACGTATTCTA   
  
  
- CTCAACACAA AAAAAACAGC ACATGTGTAG TTTTTGACAG TATGCCTGTT ATTCGATCAG CGAAACTGGG   
  
  
- TAAGTGGCAT ACAGGGTTAC AGGAACTGGG ACGGTTTACT ATTCTTGCGG TCGTGTTTCC AAAATATTCA   
  
  
- AAGAGTTTCT CTTTGTTGTG TTACCCTTTT GTGTGGTAAC GAACTGAATG TTTACCTCTA GCCAACCTAG   
  
  
- TTAGAGACCT TTGTCCAACT TCATCTTAGA TTCGGGCAGA CCCGAGTACA GAGATGTGTC GTCTGGACTA   
  
  
- GTTTTGTTTA CCCTCTTACT CTTAGTGTAT TGAGACTTTC TCAGATAGAA AGATGCTCCA CCTAAGACAA   
  
  
- ATGGAAACCA GAGACGTCTA TCATCTACAA GGAACCGGTA CCGACCTCGC TAAAATTCGC AGTTTCGACG   
  
  
- TAGTGATCGT CACTGAGGTC AAGAATTTCA AAGCCAAAAT CAGTGTTCGC AAGA

+     circadian

| Site Name | Organism | Position | Strand | Matrix score. | sequence | function |
| --- | --- | --- | --- | --- | --- | --- |
| circadian | Lycopersicon esculentum | 1308 | + | 9 | CAAAGATATC | cis-acting regulatory element involved in circadian control |

>PlantCARE\_8972   
+ GAACTGTTAA AGCCAATCAT TTAAAATAAA AAGTCAAACC GGTTTAAACC GGTTTTCAAT AAGTTAATAA   
  
  
+ CCGGACTCGG TGTTAAAGAG ATTAAATTTA ACGGTTTAAC TCAGTGAGAT CGGTTTCGCT TTCTCTCTGT   
  
  
+ CGTGTTTGAG AAGTTGAAAG AGAGGAGGAT GAGCGCTGAA GATTTCCAGA AGAAGGTTTC GATCAGAGAC   
  
  
+ TCCTCCGTCG CCGGAGAAAT GGAAATCGAA TGCGGCGGGT CTACTAGCTC CGCGGTTGGT TCGTCTCGAA   
  
  
+ CCTTGGTTTT GCTTCGGAGA CTGCTCGAGA TTCAGGAGCG TCGAGCACAG GCCTACGCCA AACTCAAAAG   
  
  
+ GTTCGATCTT TGGAGTGTTT ATGCTATTAG TTACTGTTTT TGATTCAGTT ACTTGGTGAT TGTTACTGAG   
  
  
+ GGATGTTGGA GTTAAGTAAC TGATAGTGTT GTTGAGGAAG CGATGTCTCA TAAAGTTTAG ATTTTTATCT   
  
  
+ GTTGATCTCT TGCTATGCTT TTGGAATTTG ATTAATCAGA GGGTTCTCAG AGTATGTGGA GACTAGTGGT   
  
  
+ GAAGCGCTTT ATCAGAAGCT CTGCAGCGAG ATAACTGCTG AGTTCAACGA GTGTTCCAAA CAAGTAACTG   
  
  
+ AGTTTTTTTT TCTTCTTTTG ATTGATTACC TTTATGATGT TGTTGAAGAT ACGGTGTTAA AGATTGCGTC   
  
  
+ TTTGTTTGAT GGCAATGCAA GGTACGCGAA ATGGAAACTC TGTTTCTGAA TCCTGACGTT GGAAGATCGG   
  
  
+ ATCTTGCTCA ACTGCTCAGT GACATTCAAA CTCAGGAGAA GCAGAAACTG CATCTGGTCT GATTCCTATA   
  
  
+ TACTCAATTG TTGAACATAA CTGTTAACTT GTTGGACAGA ACAAAAGTTT CTTACTTTAG TTAGTTTTTT   
  
  
+ TTTTTTTTTG CAGACGGTTA CAATACAGGT ACTGAAGAAG GCAGGGAGGC CGTCAGAACG AATGCTGACA   
  
  
+ CACGAGAACT GCAAGTTCAA GAAACCGATG CAGCACGAGT GTGTGCATCT TCATGAGATT ACAGAAGCTG   
  
  
+ AAGGAACAGA GGAAGCAGAG GCGGATGCAG AGTTTGACAA CGCTTTGAAG GAAGCAATCA GAGGAGTGCA   
  
  
+ AGACGCTGTG ACTTGCATCA ATGAGTATTT GGAAGACGTT AGGTACGAGA TTGCAGCTCT TGAAGCTGAT   
  
  
+ TAGTTGTTCT CAATCTCATC ACTTCTTTAC CCCTTTCGAC TGTTAACTTG CTATGTTGCT AATTAGCTTA   
  
  
+ AGATATAAGA TATTTAGAAA CTCAAAAGTT ATGACATTTC TTGTTGTCAA AGTTATCTAT GTTGATTACT   
  
  
+ GTGGGTAGTG GCTACAAATA CAATAGCAAA TTCCAAATTA TTTAAAACAC AATATTTGAT TTGGAAAACA   
  
  
+ ATTGATTTAA AGAGGAACTT TGTTACAAAT ATAAAAGTTA TTAGCATTTT CGGTTCAAAA TGCATAAGAT   
  
  
+ GAGTTGTGTT TTTTTTGTCG TGTACACATC AAAAACTGTC ATACGGACAA TAAGCTAGTC GCTTTGACCC   
  
  
+ ATTCACCGTA TGTCCCAATG TCCTTGACCC TGCCAAATGA TAAGAACGCC AGCACAAAGG TTTTATAAGT   
  
  
+ TTCTCAAAGA GAAACAACAC AATGGGAAAA CACACCATTG CTTGACTTAC AAATGGAGAT CGGTTGGATC   
  
  
+ AATCTCTGGA AACAGGTTGA AGTAGAATCT AAGCCCGTCT GGGCTCATGT CTCTACACAG CAGACCTGAT   
  
  
+ CAAAACAAAT GGGAGAATGA GAATCACATA ACTCTGAAAG AGTCTATCTT TCTACGAGGT GGATTCTGTT   
  
  
+ TACCTTTGGT CTCTGCAGAT AGTAGATGTT CCTTGGCCAT GGCTGGAGCG ATTTTAAGCG TCAAAGCTGC   
  
  
+ ATCACTAGCA GTGACTCCAG TTCTTAAAGT TTCGGTTTTA GTCACAAGCG TTCT  

- CTTGACAATT TCGGTTAGTA AATTTTATTT TTCAGTTTGG CCAAATTTGG CCAAAAGTTA TTCAATTATT   
  
  
- GGCCTGAGCC ACAATTTCTC TAATTTAAAT TGCCAAATTG AGTCACTCTA GCCAAAGCGA AAGAGAGACA   
  
  
- GCACAAACTC TTCAACTTTC TCTCCTCCTA CTCGCGACTT CTAAAGGTCT TCTTCCAAAG CTAGTCTCTG   
  
  
- AGGAGGCAGC GGCCTCTTTA CCTTTAGCTT ACGCCGCCCA GATGATCGAG GCGCCAACCA AGCAGAGCTT   
  
  
- GGAACCAAAA CGAAGCCTCT GACGAGCTCT AAGTCCTCGC AGCTCGTGTC CGGATGCGGT TTGAGTTTTC   
  
  
- CAAGCTAGAA ACCTCACAAA TACGATAATC AATGACAAAA ACTAAGTCAA TGAACCACTA ACAATGACTC   
  
  
- CCTACAACCT CAATTCATTG ACTATCACAA CAACTCCTTC GCTACAGAGT ATTTCAAATC TAAAAATAGA   
  
  
- CAACTAGAGA ACGATACGAA AACCTTAAAC TAATTAGTCT CCCAAGAGTC TCATACACCT CTGATCACCA   
  
  
- CTTCGCGAAA TAGTCTTCGA GACGTCGCTC TATTGACGAC TCAAGTTGCT CACAAGGTTT GTTCATTGAC   
  
  
- TCAAAAAAAA AGAAGAAAAC TAACTAATGG AAATACTACA ACAACTTCTA TGCCACAATT TCTAACGCAG   
  
  
- AAACAAACTA CCGTTACGTT CCATGCGCTT TACCTTTGAG ACAAAGACTT AGGACTGCAA CCTTCTAGCC   
  
  
- TAGAACGAGT TGACGAGTCA CTGTAAGTTT GAGTCCTCTT CGTCTTTGAC GTAGACCAGA CTAAGGATAT   
  
  
- ATGAGTTAAC AACTTGTATT GACAATTGAA CAACCTGTCT TGTTTTCAAA GAATGAAATC AATCAAAAAA   
  
  
- AAAAAAAAAC GTCTGCCAAT GTTATGTCCA TGACTTCTTC CGTCCCTCCG GCAGTCTTGC TTACGACTGT   
  
  
- GTGCTCTTGA CGTTCAAGTT CTTTGGCTAC GTCGTGCTCA CACACGTAGA AGTACTCTAA TGTCTTCGAC   
  
  
- TTCCTTGTCT CCTTCGTCTC CGCCTACGTC TCAAACTGTT GCGAAACTTC CTTCGTTAGT CTCCTCACGT   
  
  
- TCTGCGACAC TGAACGTAGT TACTCATAAA CCTTCTGCAA TCCATGCTCT AACGTCGAGA ACTTCGACTA   
  
  
- ATCAACAAGA GTTAGAGTAG TGAAGAAATG GGGAAAGCTG ACAATTGAAC GATACAACGA TTAATCGAAT   
  
  
- TCTATATTCT ATAAATCTTT GAGTTTTCAA TACTGTAAAG AACAACAGTT TCAATAGATA CAACTAATGA   
  
  
- CACCCATCAC CGATGTTTAT GTTATCGTTT AAGGTTTAAT AAATTTTGTG TTATAAACTA AACCTTTTGT   
  
  
- TAACTAAATT TCTCCTTGAA ACAATGTTTA TATTTTCAAT AATCGTAAAA GCCAAGTTTT ACGTATTCTA   
  
  
- CTCAACACAA AAAAAACAGC ACATGTGTAG TTTTTGACAG TATGCCTGTT ATTCGATCAG CGAAACTGGG   
  
  
- TAAGTGGCAT ACAGGGTTAC AGGAACTGGG ACGGTTTACT ATTCTTGCGG TCGTGTTTCC AAAATATTCA   
  
  
- AAGAGTTTCT CTTTGTTGTG TTACCCTTTT GTGTGGTAAC GAACTGAATG TTTACCTCTA GCCAACCTAG   
  
  
- TTAGAGACCT TTGTCCAACT TCATCTTAGA TTCGGGCAGA CCCGAGTACA GAGATGTGTC GTCTGGACTA   
  
  
- GTTTTGTTTA CCCTCTTACT CTTAGTGTAT TGAGACTTTC TCAGATAGAA AGATGCTCCA CCTAAGACAA   
  
  
- ATGGAAACCA GAGACGTCTA TCATCTACAA GGAACCGGTA CCGACCTCGC TAAAATTCGC AGTTTCGACG   
  
  
- TAGTGATCGT CACTGAGGTC AAGAATTTCA AAGCCAAAAT CAGTGTTCGC AAGA
