# Supplementary figures and images for: Effects of Low Nighttime Temperature on Fatty Acid Content in Developing Seeds from Brassica napus L. Based on RNA-Seq and Metabolome
Source: Plants (Basel). 2023 Jan 10;12(2):325. doi: 10.3390/plants12020325 (PMC9862530; doi:10.3390/plants12020325)

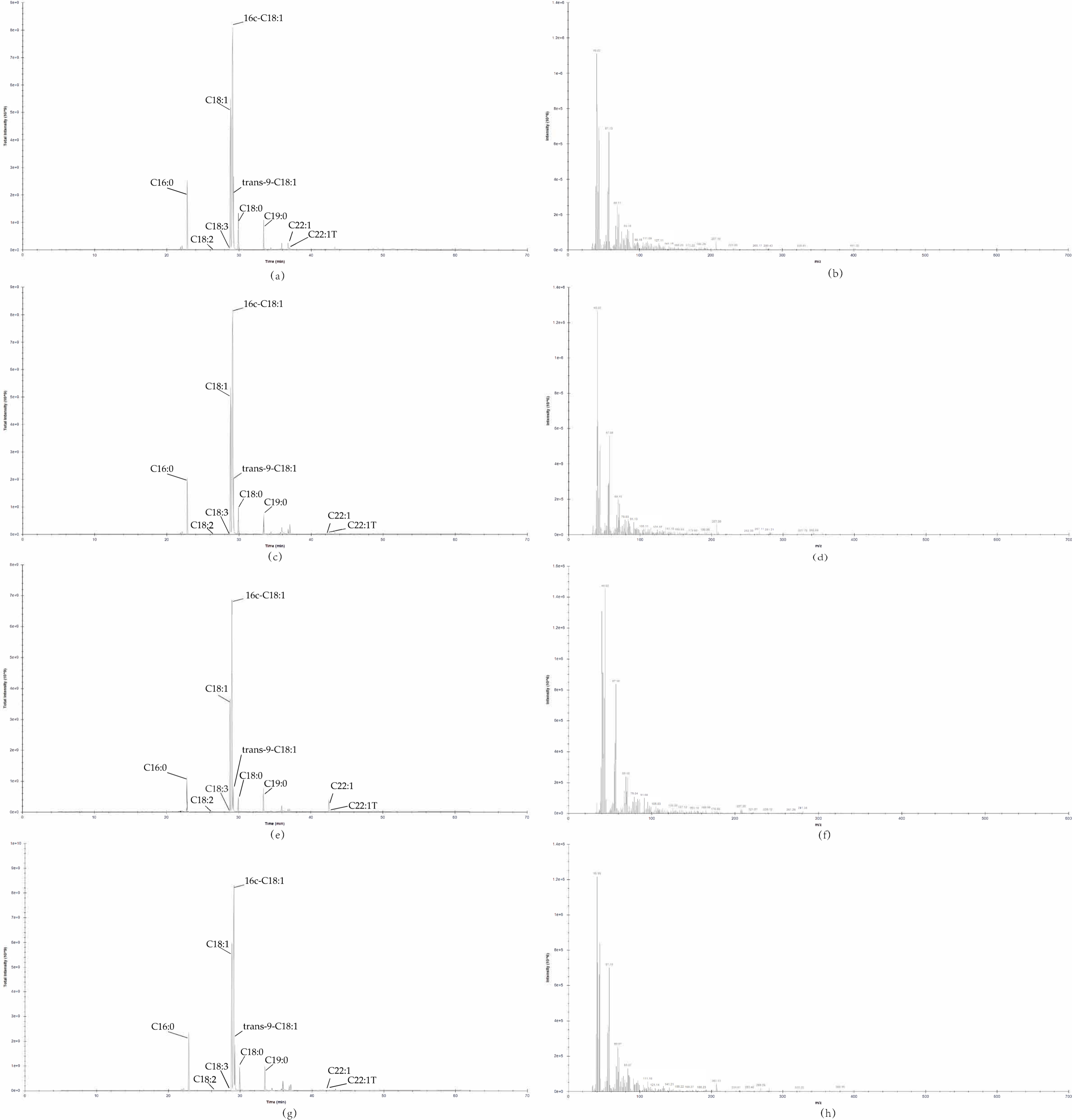

Supplement: Supplementary file 1 [file plants-12-00325-s001.zip › Figure S1.pdf]

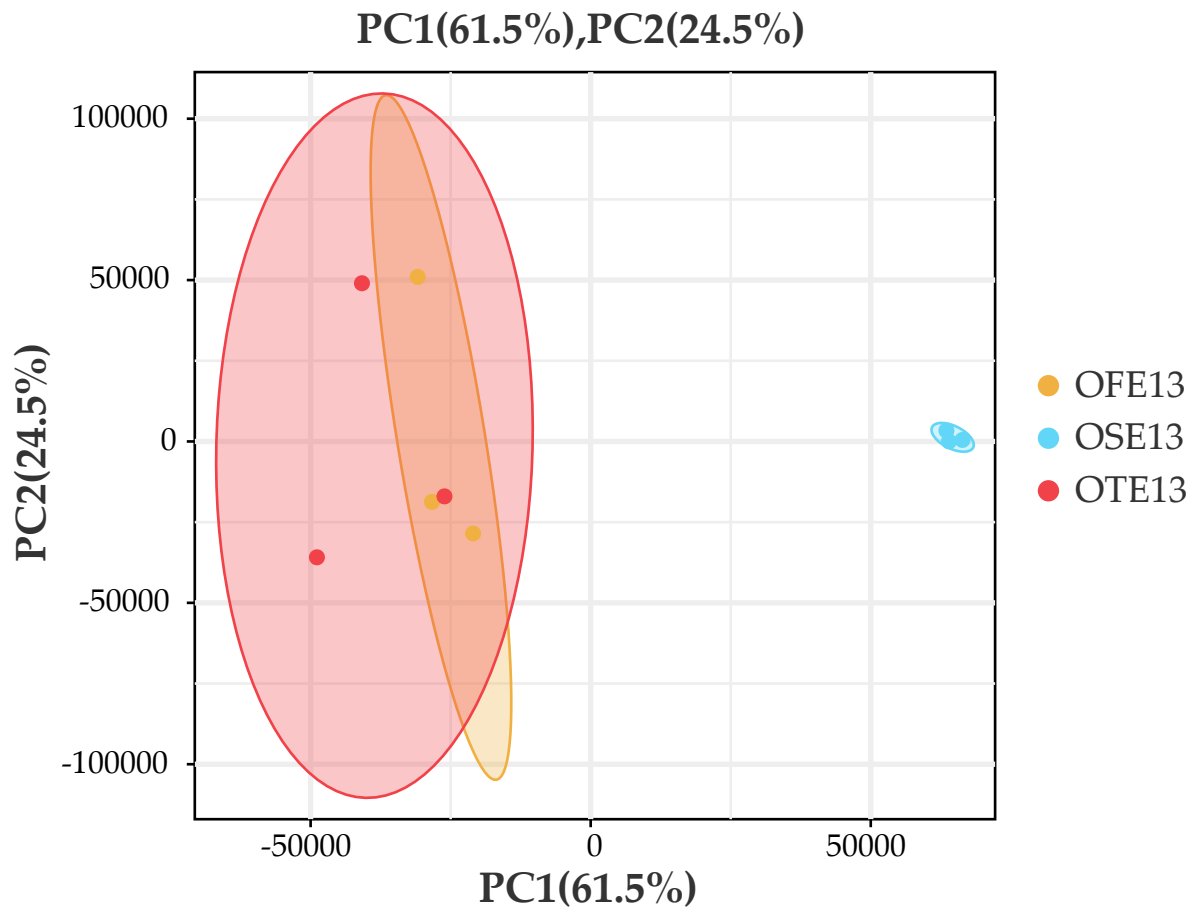

Figure S4. Principal component analysis (PCA) score chart of the RNA-seq data of STSL in LNT.

Supplement: Supplementary file 1 [file plants-12-00325-s001.zip › Figure S4.pdf]

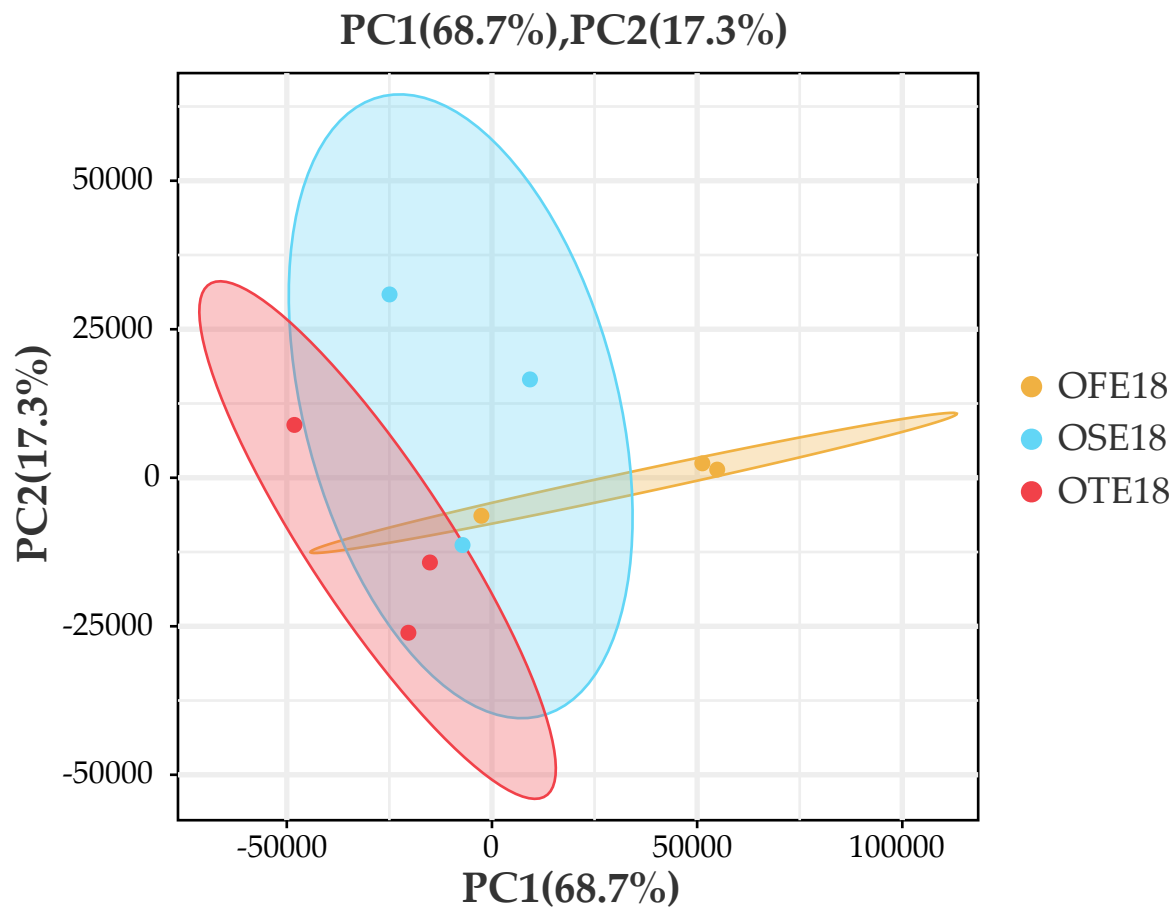

Figure S5. Principal component analysis (PCA) score chart of the RNA-seq data of STSL in HNT.

Supplement: Supplementary file 1 [file plants-12-00325-s001.zip › Figure S5.pdf]

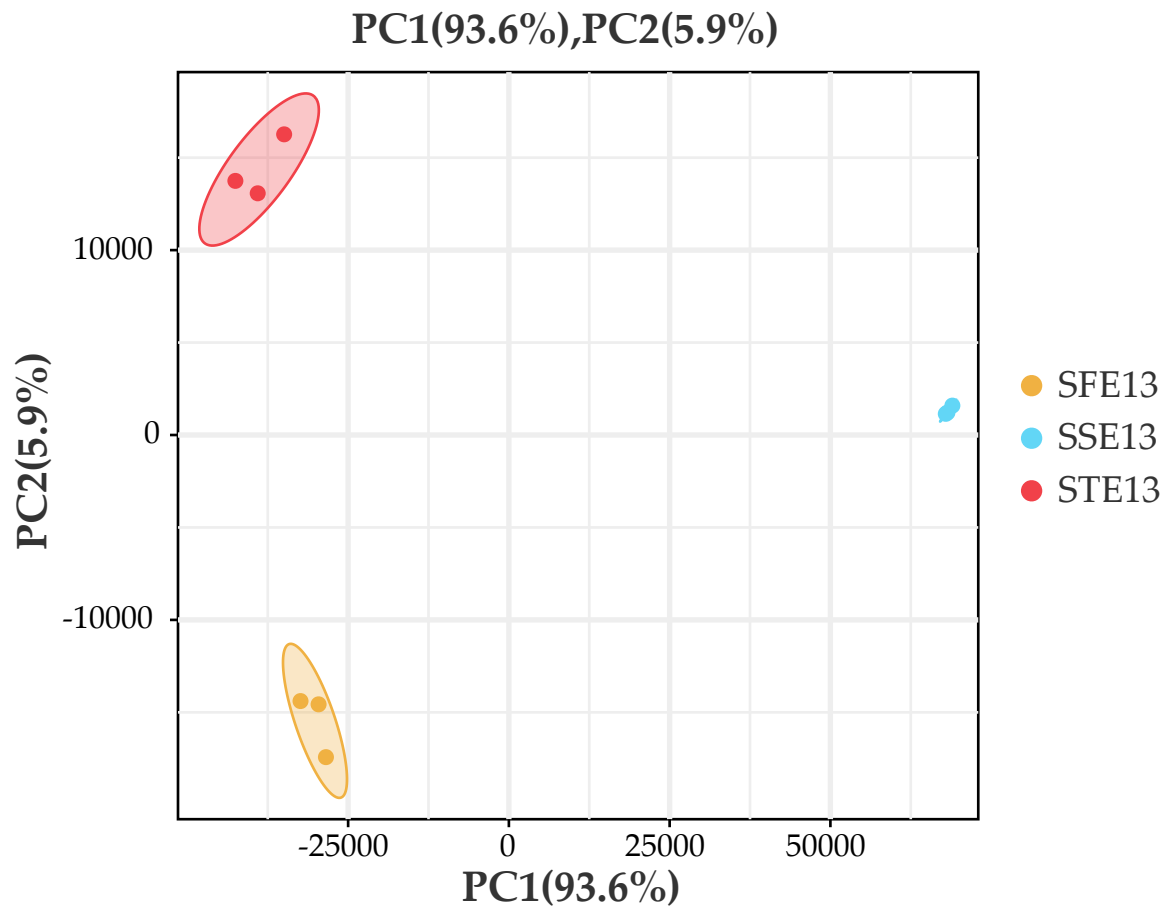

Figure S6. Principal component analysis (PCA) score chart of the RNA-seq data of WTS in LNT.

Supplement: Supplementary file 1 [file plants-12-00325-s001.zip › Figure S6.pdf]

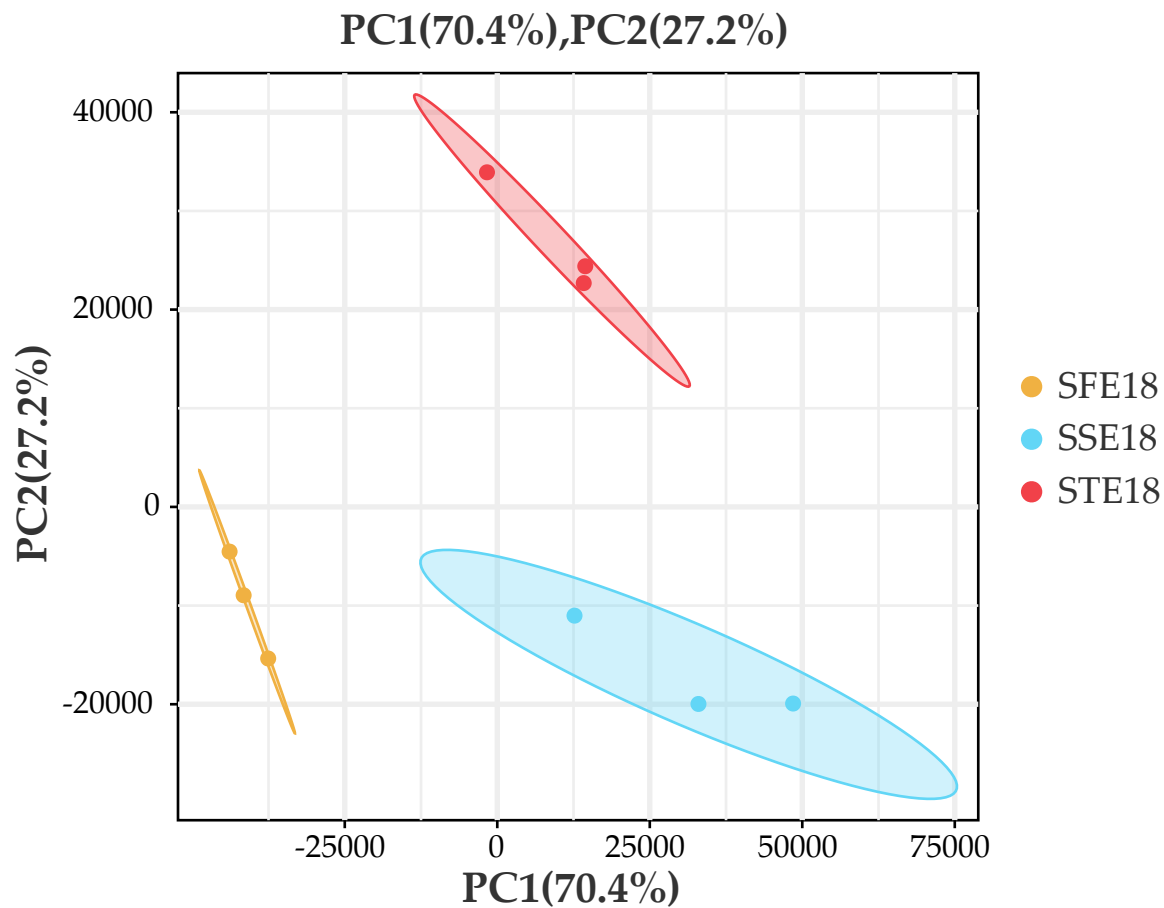

Figure S7. Principal component analysis (PCA) score chart of the RNA-seq data of WTSL in HNT.

Supplement: Supplementary file 1 [file plants-12-00325-s001.zip › Figure S7.pdf]
