# Supplementary material for: Effects of Low Nighttime Temperature on Fatty Acid Content in Developing Seeds from Brassica napus L. Based on RNA-Seq and Metabolome
Source: Plants (Basel). 2023 Jan 10;12(2):325. doi: 10.3390/plants12020325 (PMC9862530; doi:10.3390/plants12020325)
Supplement: Supplementary file 1 [file Figure S8.pdf › Figure S8.pdf]

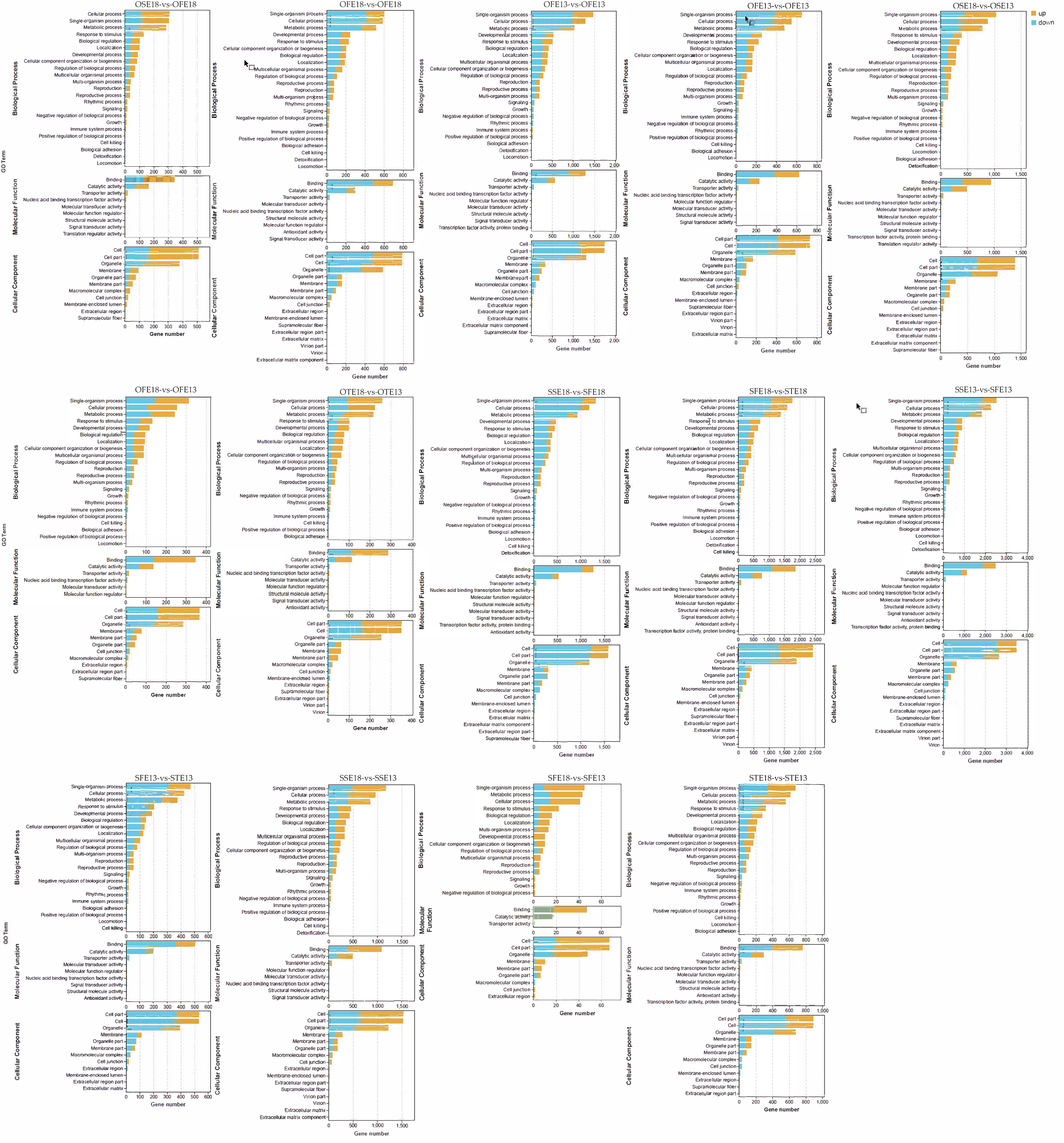

Figure S8. GO functional annotation of DEGs of in 27, 35 and 43 DAF seeds in WTSL and STSL in LNT and
